# Prediction and characterization of prophages of *Stenotrophomonas maltophilia* reveals a remarkable phylogenetic diversity of prophages

**DOI:** 10.1038/s41598-023-50449-x

**Published:** 2023-12-22

**Authors:** Zheng Fang, Man Xu, Shan Shen, Weiwei Sun, Qing Yu, Qingshan Wu, Lan Xiang, Qingbei Weng

**Affiliations:** 1 School of Life Sciences, Guizhou Normal University, Guiyang, 550025 Guizhou People’s Republic of China; 2 Qiannan Normal University for Nationalities, Duyun, 558000 Guizhou People’s Republic of China

**Keywords:** Cell biology, Microbiology, Molecular biology

## Abstract

Prophages, which enables bacterial hosts to acquire novel traits, and increase genetic variation and evolutionary innovation, are considered to be one of the greatest drivers of bacterial diversity and evolution. *Stenotrophomonas maltophilia* is widely distributed and one of the most important multidrug resistant bacteria in hospitals. However, the distribution and genetic diversity of *S. maltophilia* prophages have not been elucidated. In this study, putative prophages were predicted in *S. maltophilia* genomes by using virus prediction tools, and the genetic diversity and phylogeny of *S. maltophilia* and the prophages they harbor were further analyzed. A total of 356 prophage regions were predicted from 88 *S. maltophilia* genomes. Among them, 144 were intact prophages, but 77.09% of the intact prophages did not match any known phage sequences in the public database. The number of prophage carried by *S. maltophilia* is related to its host habitat and is an important factor affecting the size of the host genome, but it is not related to the genetic diversity of the prophage. The prediction of auxiliary genes encoded by prophage showed that antibiotic resistance genes was not predicted for any of the prophages except for one questionable prophage, while 53 virulence genes and 169 carbohydrate active enzymes were predicted from 11.24 and 44.1% prophages, respectively. Most of the prophages (72.29%) mediated horizontal gene transfer of *S. maltophilia* genome, but only involved in 6.25% of the horizontal gene transfer events. In addition, CRISPR prediction indicated 97.75% *S. maltophilia* strains contained the CRISPR-Cas system containing 818 spacer sequences. However, these spacer sequences did not match any known *S. maltophilia* phages, and only a few *S. maltophilia* prophages. Comparative genomic analysis revealed a highly conserved and syntenic organization with genomic rearrangement between the prophages and the known related *S. maltophilia* phages. Our results indicate a high prevalence and genetic diversity of prophages in the genome of *S. maltophilia*, as well as the presence of a large number of uncharacterized phages. It provides an important complement to understanding the diversity and biological characteristics of phages, as well as the interactions and evolution between bacteria and phages.

## Introduction

As natural predators of bacteria, bacteriophages (phages) play a crucial role in microbiota diversity, bacterial ecology and evolution^1–4^. Lytic phages can cause bacterial cell death and release of new phage progeny after infection (lytic life cycle), whilst temperate phages are defined by their characteristics to integrate their genome into the host genome without causing disruption (lysogenic life cycle). These latent phages, known as prophages, usually remain dormant and replicate their genomes along with the host genome^5^. During long-term evolution, some key genes of the intact prophages, such as some structural genes, are deleted by the host bacteria and become incomplete or questionable prophages. Through this domestication mechanism, the host bacteria reduce the risk of being lysed and death while some genes in prophages that provide a range of fitness benefits to host bacteria are retained^6^. Therefore, prophages are considered to be one of the main drivers of bacterial diversity and evolution^5,6^.

Prophages are one of the important mobile genetic elements (MGEs) that widely present in bacteria, and are involved in diverse bacteria life processes, including the acquisition of novel traits by through horizontal gene transfer (HGT), increased genetic variation and evolutionary innovation^5^.Expression of functional genes can confer a survival advantage to lysogens in adverse environments^6,7^, such as, the integrating antibiotic resistance genes (ARGs) for kanamycin, chloramphenicol and ampicillin^8^, carbohydrate activity enzymes (CAZys), and virulence genes (VGs) carried by prophages, which can not only increase the virulence of the host bacteria, but can even transfer non-virulent strains to pathogenic strains^9,10^. Moreover, it is important to note that intact phages can be activated by specific induction, leading to DNA excision, resumption of lytic cycling of the lysogens, and subsequent lytic release of the activated phages^7^. Given the ability of phages to switch modes of infection, prophage activation is a strategy to induce the killing of competitors after prophage release^11^. Therefore, the induction and activation of prophages is also a viable approach for the treatment of infections of bacterial diseases such as Shiga toxin-producing *Escherichia coli* infection^10^.

Bacterial CRISPR (clustered regularly interspaced short palindromic repeats)—Cas immunity is also widely recognized as an important player in phage evolution^12^. CRISPR loci consist of short DNA repeats separated by sequences, known as spacers, that match exogenous MGEs from invaders such as phages and plasmids. When the protospacer region of prophages in bacterial genomes are deleted, this may lead to interesting eco-evolutionary dynamics, suggesting an ongoing battle between phage and CRISPR-Cas systems even after the integration of a prophage into the host chromosome^13,14^.Indeed, the diversity, universality and richness of prophages have been revealed in studies of bacterial genomes of different species^11,15^, with approximately 40–50% of microbial genomes identified as carry prophages^16^. The prophages carried are variable between bacteria, with some bacteria carrying none, whilst others are polylysogenic and can carry over a dozen prophages^6^. However, due to the uncertainty of prophage induction, prophages and temperate phages have been studied far less than virulent phages^17^. In recent years, with the rapid development of large-scale bacterial genome sequencing projects and the development of prophage finding software^9,10^, our ability to detect prophages and to understand their distribution in across a range of bacteria has improved.

*Stenotrophomonas maltophilia*, a member of the *Xanthomonadaceae* family, is a Gram-negative aerobic bacterium widely distributed in hospitals, water, soil, plants, animals and humans^17,18^. It is causing concerns as a potential opportunistic pathogen with low virulence and high mortality, which can cause a variety of infections, such as pneumonia, bacteremia, meningitis, endocarditis and catheter-associated bacteremia/septicemia^18^. With the increase of nosocomial and community acquired *S. maltophilia* infections and resistance to a variety of antibiotics including cephalosporin and carbapenems, it has been identified by the World Health Organization (WHO) as one of the most underrated and important multi-drug resistant bacteria in hospitals^19,20^.

Despite the recognition of the importance of (pro)phages for the genetic diversity and evolution of host bacteria, only a few temperate and virulent phages of *S. maltophilia* have been completely sequenced to date (accessed on March 10, 2023). The 58 known *S. maltophilia* phages are circular ssDNA (single-stranded DNA) and typically linear dsDNA (double-stranded DNA) viruses with genomic sizes ranging from 5 to 168 kb. Most of these isolated viruses have been described as tailed-phages belonging to *Siphoviridae*, *Podoviridae*, *Myoviridae* and *Autographiviridae* viral family. Currently, little is known about the distribution, genetic structure, and influence of *S. maltophilia* prophage on host diversity and evolution. We used a viral prediction tools to scan the publicly available genome of *S. maltophilia* and analyzed the predicted prophage characteristics, auxiliary genes and HGT events carried by *S. maltophilia* prophages. Understanding the prevalence and characteristics of prophages from a broader genomic perspective is of great significance for exploring the extensive genetic diversity and evolution of *S. maltophilia* strains, as well as for contributing to a deeper study of viral diversity, evolution and interactions between viruses and host bacteria.

## Results

### Predicting prophages in genomes of *S*. *maltophilia* species

**Figure 1:**
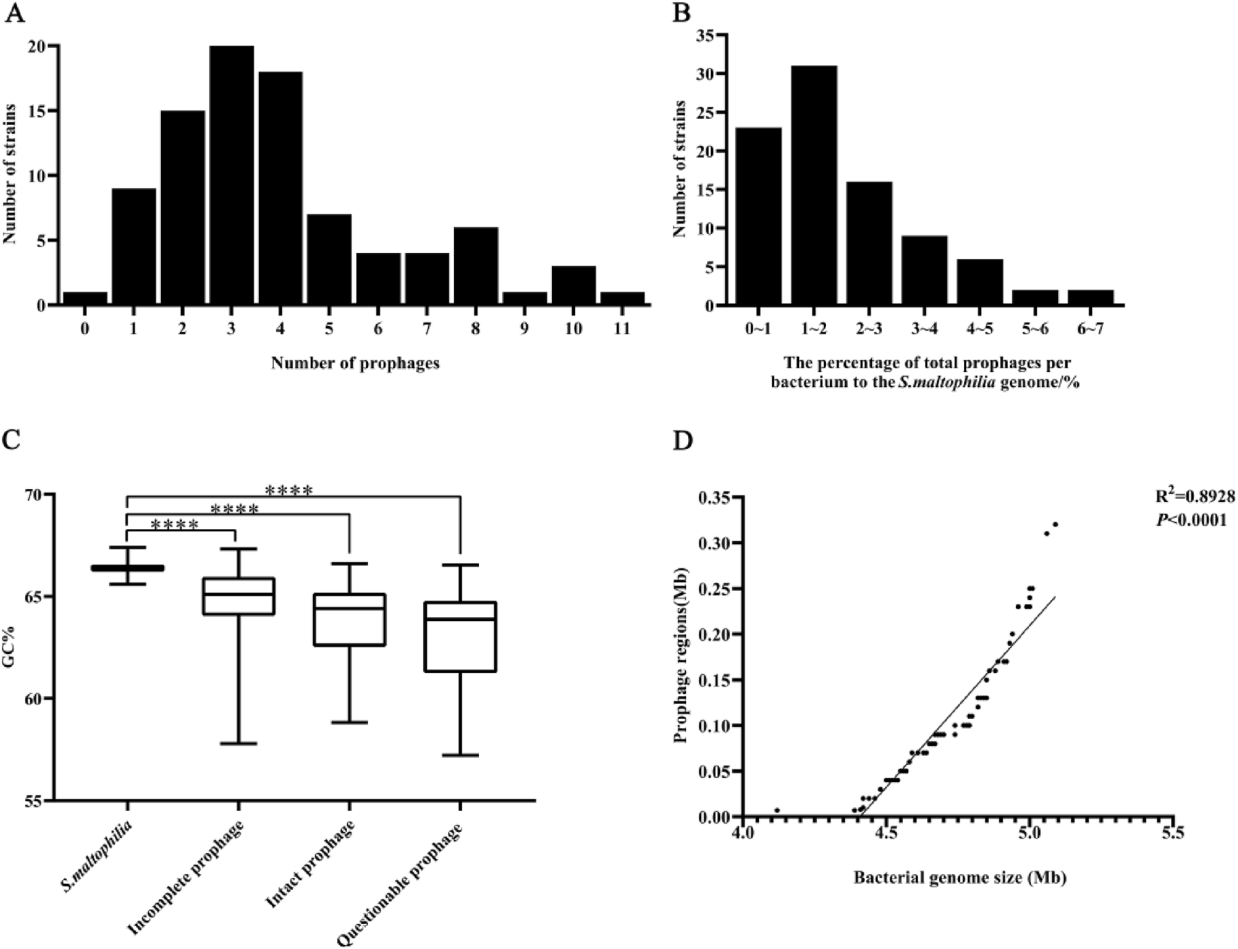
Distribution characteristics of prophages in *S. maltophilia* genome. (**A**) Number of prophages carried in *S. maltophilia* genome; (**B**) Proportional distribution of prophages carried in *S. maltophilia* genome; (**C**) GC% content of *S. maltophilia* and prophages, ****, *p* ≤ 0.0001; (**D**) Correlation between genome size of *S. maltophilia* and prophage regions.

To search for prophages, a data set of 89 publicly available complete genome assemblies of *S. maltophilia* was compiled (Supplementary Table S1), and a total of 356 prophage regions were predicted from the 88 *S. maltophilia* genomes (except that the prophage could not be predicted from strain AA1), including 144 intact prophages, 174 incomplete prophages, and 38 questionable prophages (Supplementary Table S1). The number of prophages varied extremely among strains, with each strain harboring 1 to 11 prophages (Fig. 1A), among which strain FDAARGOS_1044 isolated from American (source missing) carried the largest number of prophages (11). The 144 intact prophages were distributed in 78.7% (70/89) of *S. maltophilia* strains, with 1 to 5 intact prophages per strain (Fig. 1B). Four strains (ICU331, UHH_PC239, UHH_PC240 and NCTC10257) isolated from humans carried the highest number (5) of intact prophages. These intact prophages were used as primary subjects for subsequent analyses. The results revealed that prophage was highly prevalent and wide spread in *S. maltophilia* strains.

Among 144 putative intact *S. maltophilia* prophages, the average genome size was 25.76 ± 20.32 kb (median ± interquartile range), ranging from 5.9 kb (prophage NZ_LT906480.1-4 of strain NCTC10257) to 109 kb (prophage NZ_CP060026.1-3 of strain UHH_ICU331). The length of total intact prophage genome accounted for 0.16 to 6.29% of the total bacterial host genome. The mean guanine and cytosine content (GC content) was 63.88%, which was significantly lower than that of the host (66.14%) (Fig. 1C). The number of prophages carried was positively correlated with the host genome size (R^2^ = 0.8929) (Fig. 1D), suggesting that the strains with bigger genomic sizes allow more prophage integration events.

All 144 intact prophage genomes were aligned with publicly available phage sequences in the NCBI NR database, of which only 22.91% (33/144) matched to the eight known phages genomes to varying degrees. Of these, 30 intact prophages matched to *Stenotrophomonas* phages (including 1 PSH1, 12 phiSHP2, 3 phiSHP3, 7 phiSMA7 and 7 phiSMA6), two intact prophages matched to *Pseudomonas* phages (1 persinger and 1 phiAH14a) and one intact prophage matched to the *Rhizobium* phage RHEph01 (Supplementary Table S2). However, the remaining 111 intact prophages could not be matched to any known viral sequences, implying that most of the intact prophages harbored in *S. maltophilia* predicted in this study were unrelated to known phages and may be novel. In the subsequent diversity analysis, the 12 prophages had identical sequences in pairs, so the redundant prophages (6 prophages) were removed, and then the remaining 138 intact prophages were used.

### Diversity and phylogenetic analysis of *S*. *maltophilia* and their carrying prophages

**Figure 2:**
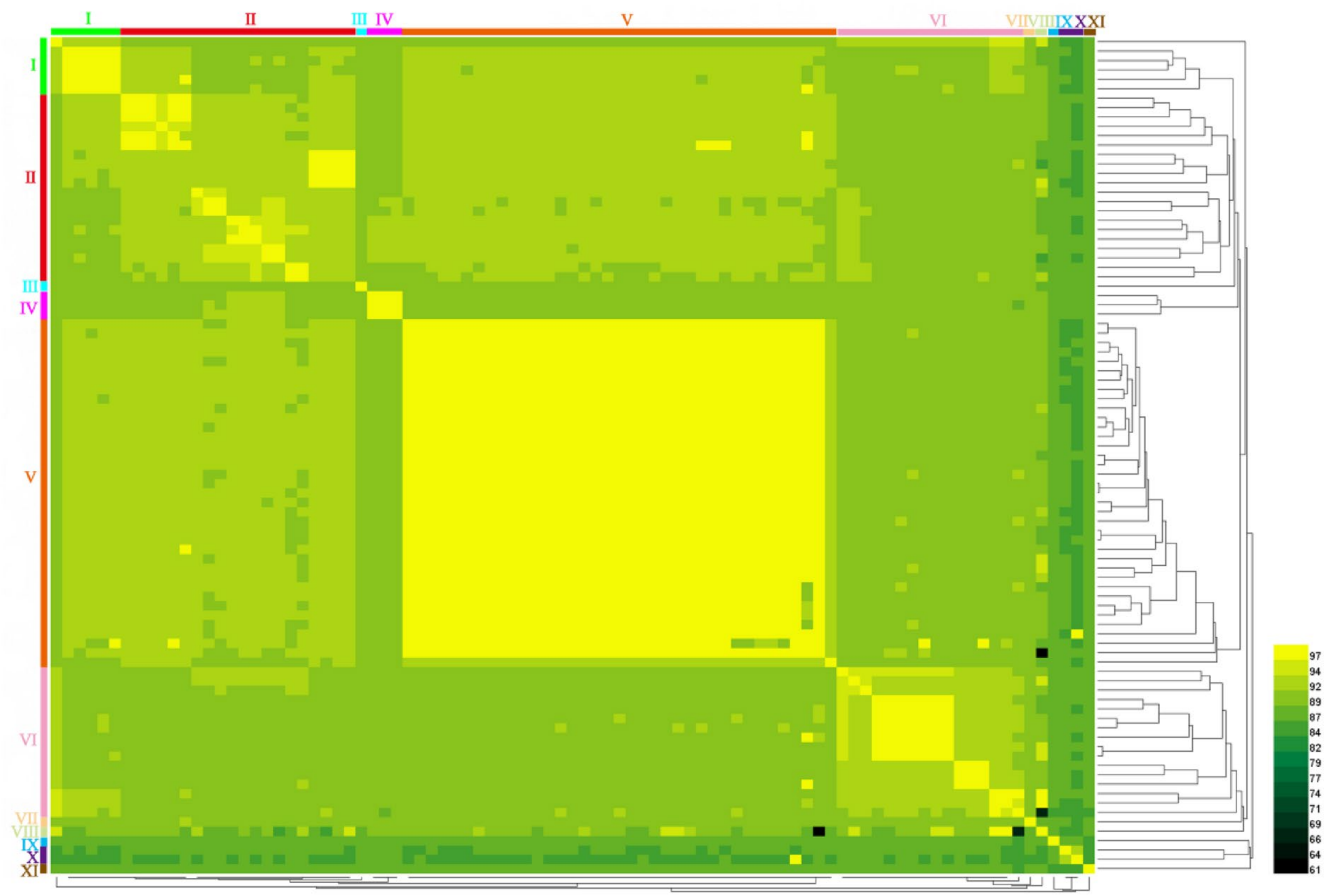
Heatmap of pairwise ANI values across 89 *S. maltophilia* genomes. The 89 *S. maltophilia* were clustered at the genomic level using ANI analysis with default parameters. Using EzBioCloud (https://www.ezbiocloud.net/) to calculate the ANI value of any two *S. maltophilias*. The resulting heatmap was drawn using HemI (Heatmap Illustrator v1.0). Colors represent ANI values with a gradient from dark black (low nucleotide similarity) to yellow (high nucleotide similarity). Gropus of strains with similar sequences were labeled as cluster I–XI.

To analyze the effect of the genetic diversity of *S. maltophilia* on the types of prophages they carry, we first established the evolutionary relationships of *S. maltophilia*. The average nucleotide identity (ANI) values between each pair of genomes were predicted to construct an ANI heatmap comprising the genomes of 89 strains of *S. maltophilia* were constructed and drawn using HemI (heatmap Illustrator v1.0)^21^ (Fig. 2). Based on ANI values, these strains could be grouped into 11 clusters (I—XI), and the ANI values of each cluster were below the threshold of new species classification (95%). This result indicates a high genetic diversity among the strains within *S. maltophilia* species.

**Figure 3:**
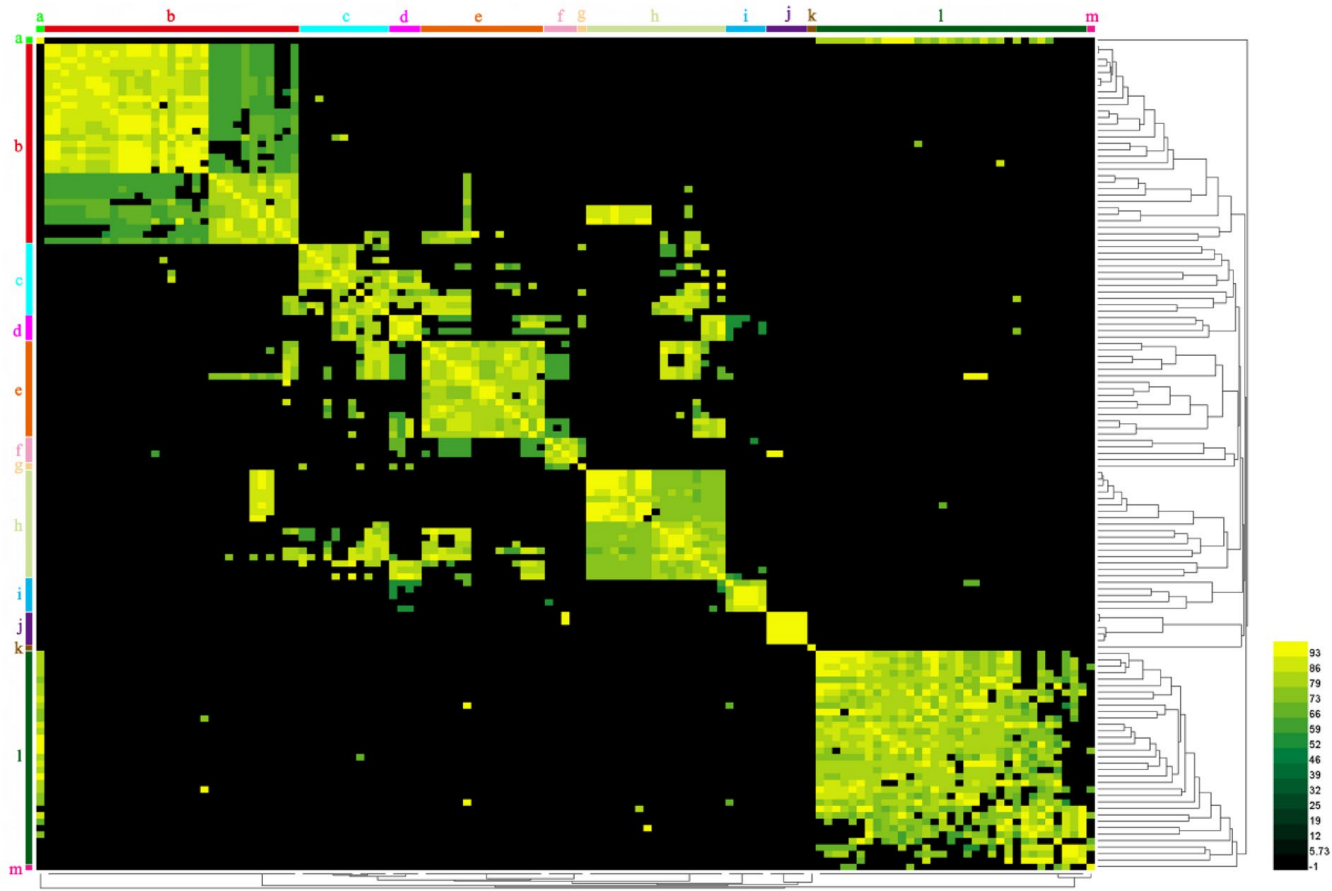
ANI heatmap of the *S. maltophilia* prophages. *S. maltophilia* prophages were clustered at the genomic level using ANI analysis with default parameters (95% ANI). Using EzBioCloud to calculate the ANI value of any two prophages. The resulting heatmap was drawn using HemI (Heatmap Illustrator v1.0). Colors represent ANI values with a gradient from dark black (low nucleotide similarity) to yellow (high nucleotide similarity). Groups of prophages with similar sequences were labeled cluster a–m.

To investigate the diversity of *S. maltophilia* prophage at the genomic level, ANI values of 138 intact prophage sequences were calculated using EZbioCloud, which showed generally low ANI values, suggesting a generally low genomic similarity among *S. maltophilia* prophage. The pairwise ANI values were arranged into a matrix and visualized in a bidirectional hierarchical clustering heatmap for graphical representation of species similarity, resulting in 13 clusters of potential interest for prophages (clusters a–m) (Fig. 3). However, for prophages that clustered in the same cluster, the bacterial host strains carrying them do not necessarily clustered together. Similarly, strains clustered in the same cluster do not necessarily carry prophage in the same cluster, indicating that the type of prophage is independent of the similarity of the host genome (Supplementary Table S3).

**Figure 4:**
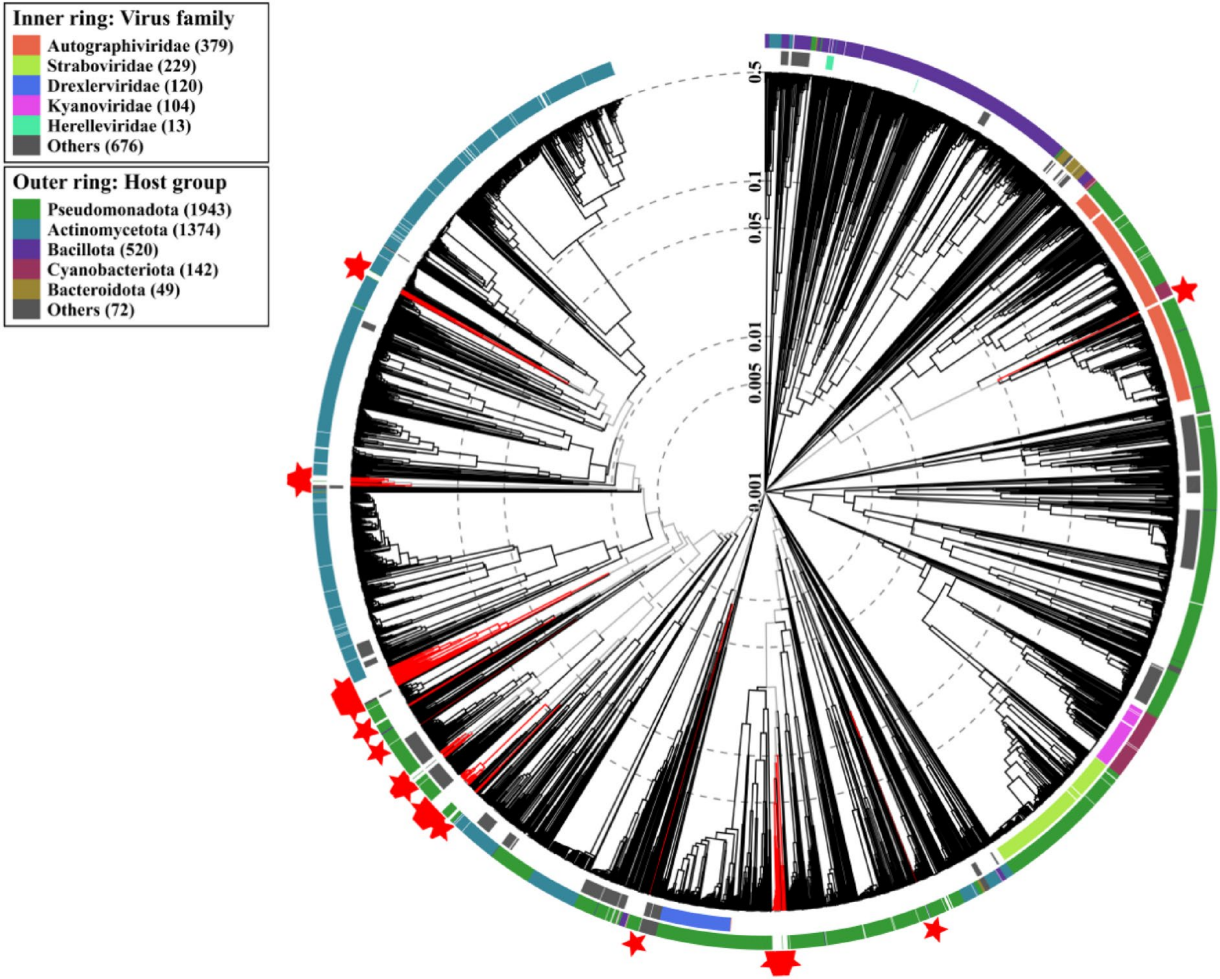
Phylogenetic analysis of *S. maltophilia* intact prophages. The 138 predicted *S. maltophilia* intact prophages were selected. The “proteomic tree” was constructed with the prophages and the known phages associated with prokaryotic hosts in public database using Viptree (https://www.genome.jp/viptree/) by genome-wide protein levels. *S. maltophilia* prophages are highlighted with red stars.

The genome sequences of 138 predicted intact prophages identified from *S. maltophilia* were compared with all known phage genomes in public databases using Viral Proteomic Tree (ViPtree, https://www.genome.jp/viptree/)^22^ (Fig. 4). The predicted *S. maltophilia* intact prophages clustered into 42 clusters, of which 41 clusters were including *Siphoviridae*, *Myoviridae* and other 18 viral families, and one was unknown viral family (Supplementary Table S4). However, only 21.01% (29/138) of the intact *S. maltophilia* prophage sequences were fallen within 3/42 clusters with known *S. maltophilia* phages. The remaining 78.99% (109/138) of the intact prophage sequences formed clusters with other known phages other than the *S. maltophilia* host. Among them, 64 sequences formed clusters with phages infecting *Pseudomonas* taxa (e.g. *Xanthomonas*, *Ralstonia*), 43 formed clusters with the phages infecting *Actinomycetota* taxa, and one sequences formed clusters with phages infecting *Euryarchaeota* taxa. One sequence clustered with the phages infecting unknown host taxa (Fig. 4 and Supplementary Table S4). These results highlight the great diversity of prophages in the genome of *S. maltophilia*.

Taken together, the results demonstrate the great genetic diversity of prophages in the genome of *S. maltophilia*.

### Correlation between habitat of *S*. *maltophilia* and carrying prophage

**Figure 5:**
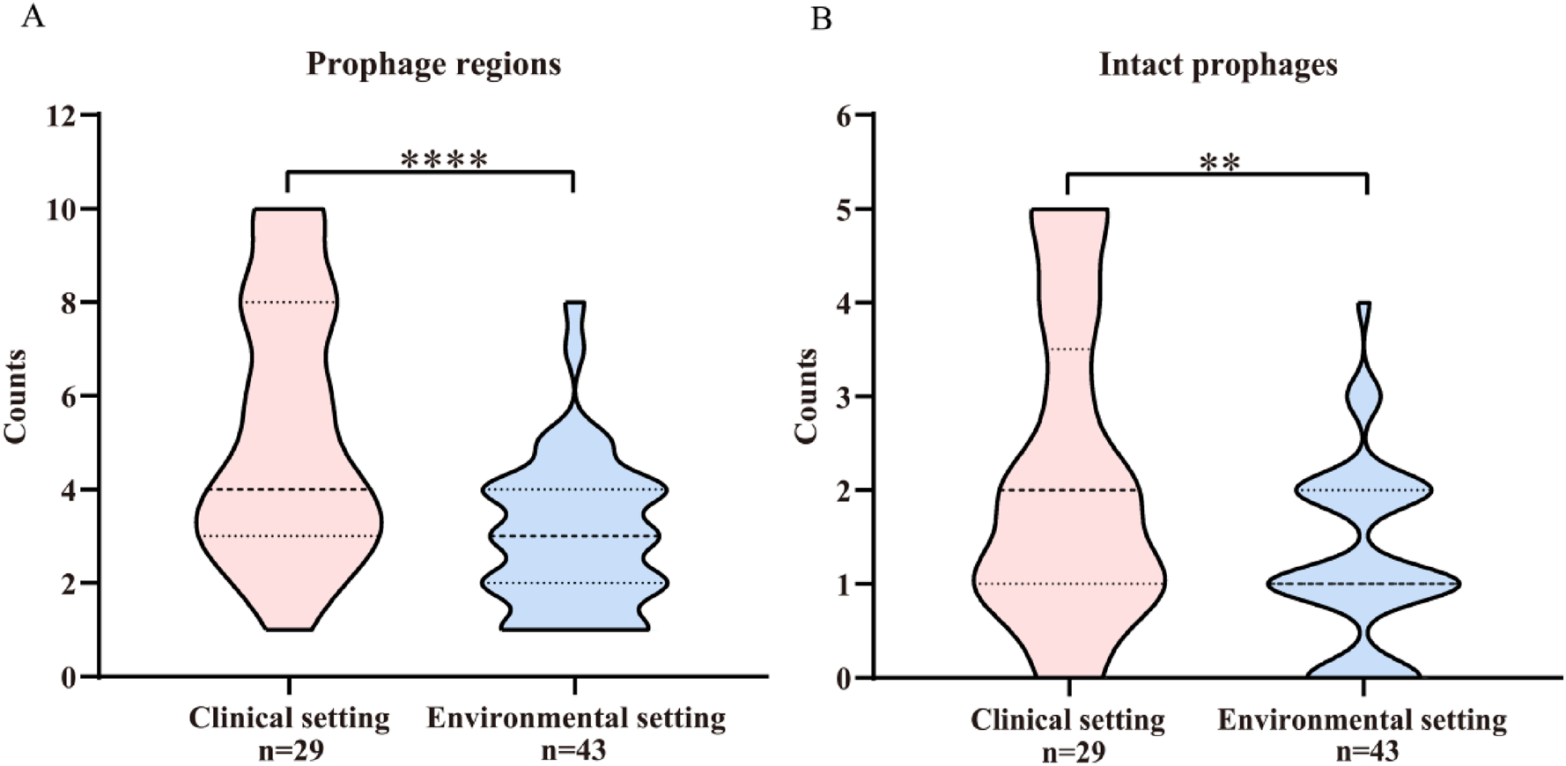
Distribution of prophages in *S. maltophilia* strains from different habitats. The 72 *S. maltophilia* strains were divided into “clinical setting” group (n = 29) and “environmental setting” group (n = 43), and the number of prophages (**A**) and the number of intact prophages (**B**) harbored by the two groups were compared. **p ≤ 0.01; ****p ≤ 0.0001.

To investigate whether the number of prophages carried by *S. maltophilia* was related to the host habitat, 72 *S. maltophilia* strains with definite isolation source were selected and divided into two groups according to their habitats: “clinical setting” group (isolated from human and hospital, n = 29) and “environmental setting” group (isolated from other habitats, n = 43). The results showed that strains from the clinical setting (151 prophage regions, including 62 intact prophages) harbored significantly greater numbers of prophages than strains from the environmental setting (130 prophage regions, including 54 intact prophages). This was obviously observed in both the predicted prophages (Fig. 5A, p < 0.0001) and intact prophages (Fig. 5B, p < 0.01).

The ANI of *S. maltophilia* showed that strains from clinical setting could be clustered into 5 clusters, most of which (68.97%, 20/29) were clustered into cluster V (Fig. 2 and Supplementary Table S3), and the intact prophages carried by these strains could be clustered into 10 clusters, of which 30.65% (19/62) were clustered in cluster b (Fig. 3 and Supplementary Table S3). The strains from the environmental setting could be clustered into nine clusters, of which 30.23% (13/43) were clustered in cluster II (Fig. 2 and Supplementary Table S3), and the intact prophages they carried could be clustered into nine clusters, among which 31.48% (17/54) were clustered into cluster l (Fig. 3 and Supplementary Table S3). Although most strains from the clinical setting clustered together (cluster V), the intact prophages they carried did not clustertogether, but instead cross-clusered with the intact prophages carried by strains from the environmental setting (Supplementary Table S3). The findings indicate that the abundance of prophages may be associated with host habitats.

### Auxiliary genes and HGTs mediated by prophages in *S*. *maltophilia*

ARGs was not predicted for any of the prophages except for a questionable prophage carried by strain HW002Y isolated from an ICU ward in Malaysia. The prophage predicted by HW002Y contained 11 ARGs, which involved drug resistance to aminoglycoside antibiotic, rifamycin antibiotic, carbapenem, cephalosporin, penam, macrolide antibiotic, disinfecting agents and antiseptics, sulfonamide antibiotic and tetracycline antibiotic (Supplementary Table S5).

A total of 53 VGs were predicted in 11.24% (40/356) of the prophages, which were involved in regulation, stress survival, adherence, immune modulation, biofilm, effector delivery system and motility (Supplementary Fig. S1). Among them, *Pilz* genes associated to adherence were the most (16 genes), followed by *Fur* genes related to the regulation of iron uptake (9 genes). There biofilm-related genes were predicted in the incomplete phages carried by SCAID WND1-2022 and FDAARGOS isolates that were isolated from humans. Nine VGs were predicted in only 6.25% (9/144) of the intact prophages, and none of the remaining intact prophages were predicted (Supplementary Table S5).

The enzymes involved in the regulation of complex carbohydrates assembly and breakdown are collectively designated as CAZys^23^. A total of 169 CAZys were predicted from 44.1% (78 intact prophage, 62 incomplete prophages and 17 questionable prophage, 157/356) of the prophages, including 159 glycoside hydrolases (GHs), four glycosyl transferases (GTs), three carbohydrate esterases (CEs), two carbohydrate-binding modules (CBMs) and one auxiliary activities (AAs) (Supplementary Table S5). Only 4.73% (8/169) of CAZys were predicted to contain signal peptides, indicating that CAZys encoded by *S.maltophilia* prophages were mainly intracellular.

**Figure 6:**
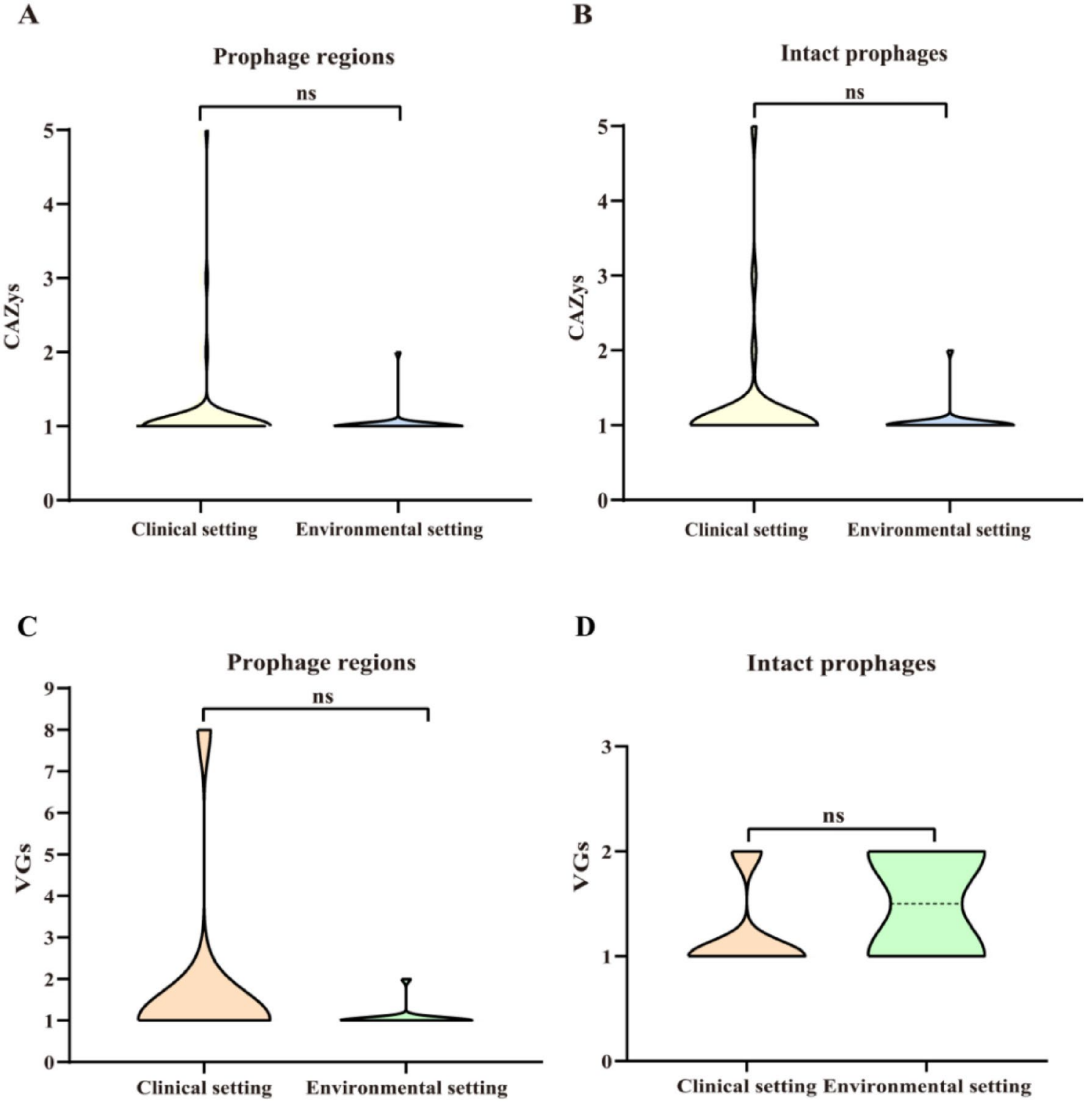
Amount of CAZys and VGs encoded by prophages harbored in *S. maltophilia* strains from different habitats. The amount of CAZys encoded by prophage regions (**A**) and intact prophages (**B**); The amount of VGs encoded by prophage regions (**C**) and intact prophages (**D**). ns, *p* > 0.05.

The amounts of CAZys (Fig. 6A,B) and VGs (Fig. 6C,D) encoded by the prophages harbored in *S. maltophilia* in the clinical setting group did not show significant difference from those in the environmental setting group (*p* > 0.05). The results showed that the habitats of *S. maltophilia* did not affect the amount of CAZys and VGs encoded by the prophage they harbored.

In order to evaluate the contribution of prophages to HGTs events in *S. maltophilia*, HGTector software (https://github.com/qiyunlab/HGTector)^24^ was used to predict HGT events in 89 strains of *S. maltophilia*, and a total of 6520 HGT events were predicted. Of these, 6.25% (425/6520) HGTs match perfectly to the sequences of *S. maltophilia* prophage, including 253 intact prophages, 108 incomplete prophages and 27 questionable prophages (Supplementary Table S6), suggesting that the most of prophages (72.29%) might be involved mediating these HGT events in *S. maltophilia.* These HGT genes involved heat or acid-resistance protein, heavy metal translocating P-type ATPase, recombinase family protein, tetracycline resistance transcriptional repressor TetR (A), etc.

### CRISPR-Cas and spacer prediction

**Figure 7:**
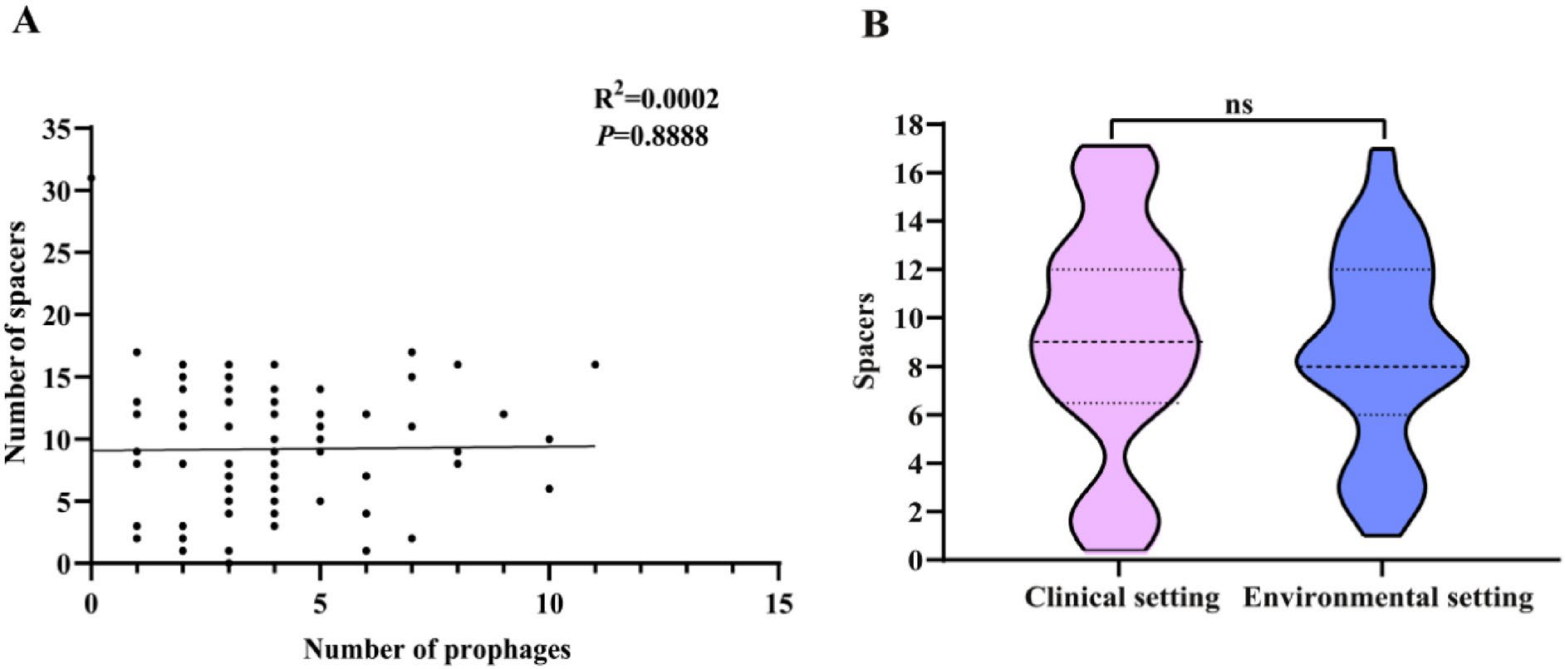
The CRISPR spacers within *S. maltophilia* genomes. (**A**) The distribution relationship between spacers and number of *S. maltophilia* prophages; (**B**) The number of spacers of *S. maltophilia* prophages between clinical setting group and environmental setting group, ns, *p* > 0.05.

The genomes of 89 *S. maltophilia* strains were predicted for the presence of CRISPR arrays, and 97.75% strains (87/89, except UHH_SKK55 and SKK55) contained CRISPR-Cas system. A total of 818 spacers were obtained from the CRISPR locus (Supplementary Table S7), of which strain AA1 (containing 31 spacers) was found to contain the most spacers of *S. maltophilia*. Analysis by GraphPad Prism 8.0.1 revealed that there was no significant correlation between the number of *S. maltophilia* prophage and the number of spacers (R^2^ = 0.0002) (Fig. 7A), indicating that the CRISPR spacer had little effect on the number of *S. maltophilia* prophage. Furthermore, the number of spacers in the clinical setting group was comparable to that of in the environmental setting group (*p* > 0.05) (Fig. 7B).

According to the similarity of spacer sequences, the 818 CRISPR spacers sequences of *S. maltophilia* were divided into 514 operational taxonomic units (OTUs), of which 71.20% (366/514) OTUs contained only one sequence each, and the remaining 148 OTUs contained more than two sequences (Supplementary Table S7). CRISPR spacer sequences were aligned to the genome sequences of the predicted *S. maltophilia* prophages and known phages in NCBI public database. The spacer sequences of only 12.84% OTUs (66/514) matched to 20.79% (74/356) of the *S. maltophilia* prophages sequences, of which 31 OTUs spacers matched to 26 intact prophage sequences. Moreover, spacer sequences from all but six strains UHH, ICU331, NCTC10498, FZD2, X28 and FDAARGOS, failed to target predicted prophage sequences in their respective genomes. In addition, except for the spacer sequence of *S. maltophilia* FDAARGOS_649 and SVIA2, which target *Synechococcus* phage syn9 (NC_008296.2) and *Arthrobacter* phage wildwest (OR521060.1), respectively, none of the remaining spacer sequences were predicted to target any known phage in the NCBI database including *S. maltophilia* phage.

### Comparative genomic analysis of *S*. *maltophilia* prophage

**Figure 8:**
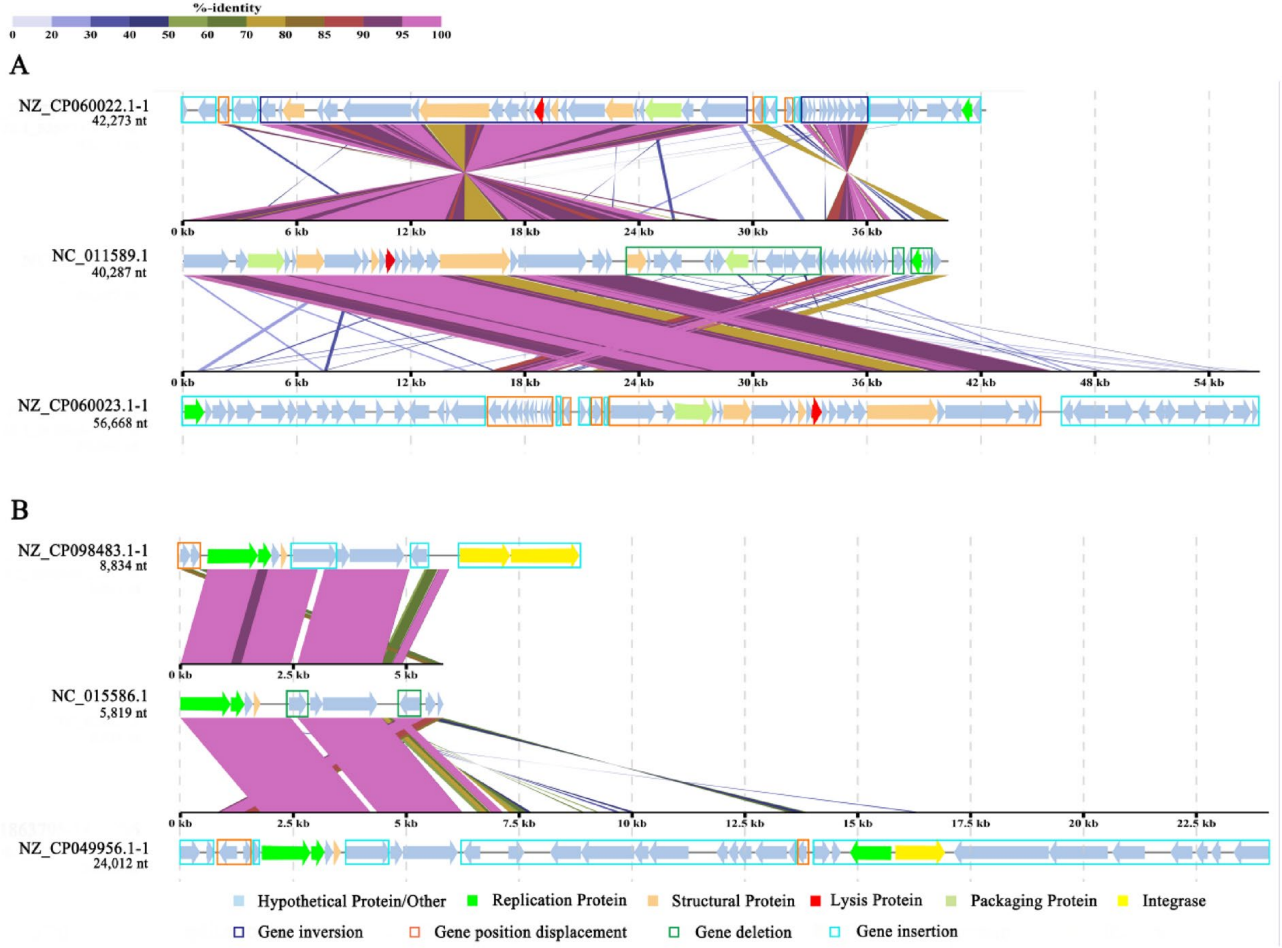
Comparative genomic analysis of *S. maltophilia* prophage and *S. maltophilia* phage by Viptree. (**A**) *S. maltophilia* prophage and *S. maltophilia* phage S1. (**B**) *S. maltophilia* prophage and *S. maltophilia* phage phiSHP2. Each arrow indicates an encoding gene, genes predicted to have similar functions are labeled with the same color. The colour spectrum between genomes reflect amino acid identity as shown in the percentage based legend. Regions marked with different clour boxes mainly enumerated the genes positions and transcription directions of similar genes among phages. blue box: gene inversion, orange box: gene position substitution, green box: gene deletion, cyan box: gene insertion.

The selected prophages was further looked into and with known *S. maltophilia* phages with similar sequences (Fig. 8, Supplementary Fig. S2 and Table S4). In most cases, we observed a highly conserved and syntenic organization between the phage and prophage genomes with a certain rearrangement of gene blocks. As shown in Fig. 8, prophage (NZ_CP060022.1-1) carried by strain UHH_PC240 and prophage (NZ_CP060023.1-1) carried by strain UHH_PC239 shared a highest sequence similarity (over 70%) with *S. maltophilia* phage S1 (NC_011589.1), and contain most of the genes of phage S1. Similarly, prophage (NZ_CP098483.1-1) carried by strain NCTC10498 and prophage (NZ_CP049956.1-1) of strain 142 shared a highest sequence similarity with *S. maltophilia* phage phiSHP2 (NC_015586.1). They contain at least 80% of phage phiSHP2 genes with more than 95% similarity. Besides that, genomic rearrangements were observed between the predicted prophages NZ_CP060022.1-1 and NZ_CP060023.1-1 and phage S1 (Fig. 8A), as well as the predicted prophages NZ_CP098483.1-1 and NZ_CP049956.1-1 and phage phiSHP2 (Fig. 8B), including gene inversion (blue boxes), gene position substitution (orange boxes), gene deletion (green boxes), and gene insertion (cyan boxes). In addition, integrase genes were predicted in both prophages NZ_CP098483.1-1 and NZ_CP049956.1-1, but not in phage phiSHP2. These results suggest that a variety of gene recombinations may occur after phage infection of different *S. maltophilia*.

## Discussion

Prophages are the major biological drivers of bacterial ecology and evolution through strategies such as symbiosis, dependency, and dormancy^25,26^. *S. maltophilia* exists widely in various environments. In this study, prophage prediction analysis showed there was a high prevalence and diversity of prophages in *S. maltophilia*, and further demonstrated the contribution of prophages to genetic diversity and plasticity of *S. maltophilia*. The diversity of prophages in *S. maltophilia* provides a new perspective on the genetic diversity of *S. maltophilia*.

Genomic prediction analysis revealed the prevalence of prophage in *S. maltophilia*, suggesting that almost all strains have been attacked by phages before^27^. Except for one strain, almost all *S. maltophilia* strains in this study were predicted to carry prophages, with a total of 356 predicted prophages, including 40.45% of intact prophage and 59.55% questionable and incomplete prophages (Supplementary Table S1). Under selection pressure, prophage genes are rapidly deleted from bacterial genomes, but mutations in one or more of the genetic elements required for excision result in the failure of the prophage excision from the host genome rendering them ‘grounded’ or defective prophages^6,28,29^. The identification of intact prophages remains to be further confirmed by induction and formation infectious phage particle. The prevalence of prophages in bacterial genomes may reflect a combination of direct competitive benefits in certain environments and their role in the exchange of genetic material^8,30^.

The number of prophages carried by *S. maltophilia* is related to their host habitat and is an important factor affecting the size of the host genome. Prophage is carried in large number in a variety of bacteria^31–33^, similar to bacteria such as *E. coli* and *Salmonella enterica*^7^, *Acinetobacter baumannii*^33^, host genome size of *S. maltophilia* was positive correlation with the number of prophages carried by the strains (Fig. 1). In addition, *S. maltophilia* from clinical setting carried more predicted and intact prophages (Fig. 5), indicating that *S. maltophilia* from clinical setting tended to integrate more prophages in response to various environmental stresses than strains from environmental setting^31^. Environmental stress can induce bacteria to develop a range of mechanisms for resistance against adverse environments, and phages are often considered to confer certain advantages for host adaptation to the environment^34^. The high prevalence and carriage number of prophage may account for the ubiquity of *S. maltophilia* in the environment^35^.

*S. maltophilia* exhibits high genetic diversity within species. The 89 strains of *S. maltophilia* can be divided into 11 clusters based on the ANI value. The genetic diversity of the strains of *S. maltophilia* may be related to the diversity of the prophage genomes carried by these strains. The 138 complete prophage sequences harbored in *S. maltophilia* were distributed in 13 clusters and exhibited generally low ANI values and genome similarity (Fig. 3). Moreover, genetic recombination events may drive the diversity of the host bacterial populations at the level of the prophage genome, promoting their continuous evolution^36^, due to the presence of various degrees of insertions, deletions, and inversions in the prophage of *S. maltophilia* (Fig. 8 and Supplementary Fig. S2). Furthermore, the genetic diversity of the strains of *S. maltophilia* may also be related to their carrying a large number of HGT events (Supplementary Table S6). Previous studies have reported that prophage-mediated HGT promotes *S. maltophilia* acquiring antibiotic resistance^37^. Most (72.29%) of the *S. maltophilia* prophages mediate HGT events, including recombinase family protein and resistance proteins. It was implied that *S. maltophilia* prophage not only directly mediates host HGT events, but also promotes the occurrence of host HGT events through related HGT events genes such as recombinase family protein^24,38^. However, prophage was predicted to mediate only 6.25% of HGT events in *S. maltophilia*, implying that more HGT events in *S. maltophilia* may be mediated by transformation or plasmid conjugation^39^. Taken together, these results suggest that genetic recombination and HGT events may be driving the diversity of *S. maltophilia* within species populations at the level of the prophage genome.

The predicted *S. maltophilia* prophages showed highly novel genome. Previous studies have shown that most of the predicted prophages harbored by *Lactobacillus* match to *Lactobacillus* phages, and most of the prophages are known and have sequences consistent with the phage sequences in public database^31^. However, majority of the predicted *S. maltophilia* prophages (77.09%) did not match any known phage sequences. The lack of known phage information indicates that there are still few known isolated and characterized phages, including *S. maltophilia* phages. In addition, some of the predicted prophages shared higher genomic similarity with the phages that infect *Rhizobium* and *Actinobacteria* (Supplementary Table S4), suggesting that *S. maltophilia* phages can not only spread across genera, but also potentially across families and even orders.

The transmission of ARGs by *S. maltophilia* may not be predominantly mediated by prophages. No ARGs were predicted for *S. maltophilia* prophages except for one questionable prophage carrying 11 ARGs. Intact prophage is generally considered to be an important carry and repository of ARGs, leading to the widespread dissemination of antibiotic resistance in host bacteria^31,37^. For example, multiple ARGs were predicted in intact prophage analysis of various bacteria including *A. baumannii*, *E. coli*, *Klebsiella pneumoniae,* and *Lactobacillus*^31,33,40,41^. However, Wendling et al. demonstrated that the predicted prophages encoding ARG typically cannot confer resistance^8^. They suggested that there are 30% fewer ARGs encoded on prophages than previously thought, indicating that prophage-mediated ARGs was significantly overestimated.

The prophages harbored numerous auxiliary genes related to the metabolism and virulence of both the phage and the bacteria^42^. However, only a small number of *S. maltophilia* prophages (11.24%) were predicted to contain VGs, which is significantly lower than VGs carriage propotion of many other bacteria prophages, such as 70.4% for *E. cloacae*, 72.3% for *S. aureus*^43^. *pilZ* (adhesion) and *Fur* (regulation) were the two most abundant VGs carried by *S. maltophilia* prophages. *Fur* is a global regulator that integrates multiple biological signals and regulates several potential pathways to contribute to the virulence of bacterial pathogens^44^, indicating that the prophage may contribute to the virulence of *S. maltophilia*. According to prediction, 44.1% of *S. maltophilia* prophages encode 169 CAZys. Of these, glycoside hydrolase (GH) with glycoprotein hydrolase enzyme activity was the most abundant auxiliary gene. GH has been reported to be critical for the growth of *Streptococcus gordonii* under the limited availability of fermentable carbohydrates^45^. CAZys encoded by prophages may enable *S. maltophilia* to utilize a wide range of carbon sources and tolerate oligotrophic conditions, thus allowing it to survive and persist under many adverse conditions^35^.

Multiple putative CRISPR-Cas systems were identified in almost all *S. maltophilia* genomes (Supplementary Table S7). Analysis of CRISPR spacer sequences provided evidence of historical interactions between *S. maltophilia* and phage^32,46^. However, most CRISPR spacer sequences could not target (pro)phage, only 12.84% of spacer sequences matching 20.79% of the *S. maltophilia* prophage, and one spacer sequence from *S. maltophilia* could target two known phages. Similarly, only a small number of CRISPR spacer sequences in *Salmonella* and *E. coli* were predicted to target their prophages^7^. It is speculated that extensive genetic recombination of *S. maltophilia* prophage (Fig. 8) may lead to mutations in (pro)phage sequences that prevent the alignment of CRISPR spacer sequences to their targets^12,47,48^ thereby impeding their function and spacer recognition^49,50^. In addition, the scarcity of sequenced prophages in public databases may also account for the the lack of targeting of spacer sequences. To date, the NCBI genome database contains only 22,029 complete phage genome sequences (including 58 *S. maltophilia* phages). Thus, there are still a significant number of uncharacterized (pro)phages that need to be further identified in the environmental microbiomes. Genomic analysis of the prophage will provide important complementary data to understand the diversity and biological properties of the phage in this species.

## Methods

### Prophage identification

To determine the prevalence of the prophage sequences within *S. maltophilia* genomes, all *S. maltophilia* genomes uploaded to the NCBI genome database (accessed on 01 December 2022) with assembly levels of “chromosome” and “complete” were included in this study and screened using PHASTER (http://phaster.ca/) with default parameters. It mostly includes strain genomes isolated from clinical environments (human or hospital environments), water (drink water or freshwater stream) and soil (rhizosphere), as well as missing environmental information. A total of 89 *S. maltophilia* genomes were considered, albeit one genome (NZ_CP011306.1) was excluded because of nucleotide duplication with another (NZ_CP011305.1).

PHASTER hits were automatically classified as intact prophage (score > 90), questionable prophage (score 70–90), and incomplete prophage (score < 70) based on phage size, similarity to known phages, and presence of phage-like and phage cornerstone genes (such as ‘capsid’, ‘head’, ‘plate’, ‘tail’, ‘coat’, ‘portal’ and ‘holin’)^40^. All predicted intact prophages were further identified by BLASTn alignment against the NCBI NR database, and subsequent analyses were performed after redundancy removal.

### ANI analysis and heatmap visualization

Analysis of *S. maltophilia* and *S. maltophilia* prophage were performed at the genomic level using ANI analysis with default parameters (95% ANI). EzBioCloud (https://www.ezbiocloud.net/) was used to calculate the ANI value of any two *S. maltophilias* or any two intact prophages. Heatmap visualization and hierarchical clustering were performed using HemI (Heatmap Illustrator v1.0).

### Phylogenetic analysis of prophage

Intact *S. maltophilia* prophages were selected for phylogenetic analysis, and Viral Proteomic Tree (ViPtree) server (https://www.genome.jp/viptree/, accessed on 10 March 2023) was used to generate a “proteomic tree” of the predicted prophages and to infer their position within the tree of viral life.

### Auxiliary genes encoded by prophage

ARGs and VGs were scanned within the prophage sequences using ResFinder 4.1 (https://cge.cbs.dtu.dk/services/ResFinder/) and VFanalyzer tools from the VF database (http://www.mgc.ac.cn/VFs/), respectively. CAZys were identified within the prophage sequences by dbCAN2 (http://bcb.unl.edu/dbCAN2/). All prediction parameters of the target genes were set as e-value ≤ 0.0001 and coverage ≥ 50%.

### Prediction of HGT mediated by prophages

HGTector v2.0b3 was used to identify putative HGT^22^. A pre-built default reference database (dated Jan 02, 2023) was downloaded (https://github.com/qiyunlab/HGTector) and the database was compiled using DIAMOND. Installation and running code for HGTector can be found at the following website (https://github.com/qiyunlab/HGTector). The parameter settings of “hgtector analyze” followed previous studies^14,16^ and were slightly modified to meet stricter criteria. Set “evalue” to “1e-20”, “–identity” to “50%”, “–coverage” to “75%”, “–self-tax” to “40,323” (NCBI taxon ID for genus *Stenotrophomonas*), “–self-rank” to “genus”, “–self-low” flag to “yes”, “–close-tax” to “32,033” (NCBI taxon ID for family *Xanthomonadaceae*), and “–bandwidth” method to “grid”. The predicted HGTs were aligned with the sequences of *S. maltophilia* prophages to identify the HGTs mediated by the prophage (100% coverage and similarity) and to assess the contribution proportion of *S. maltophilia* prophage to HGT.

### CRISPR analysis

The putative CRISPR-Cas system was identified with CRISPRFinder program (https://crisprcas.i2bc.paris-saclay.fr/) using default parameters. A spacer OTU is defined as a group of the same spacer. All OTUs sequences were mapped against the prophage sequences predicted in this study and the known phage sequences with complete genomes in the NCBI database (dated October 19, 2023, a total of 22,029 phage sequences, including 58 *S. maltophilia* phage sequences) using nucleotide BLAST searches^29^. Spacers must match the target with 100% identity over the entire length of the spacer (i.e. zero mismatches)^48^ with an e-value ≤ 0.0001.

### Comparative genomic analysis

Based on the BLASTn alignment results, the intact phages with the highest sequence similarity to the public phage data in NCBI were selected for comparative genomics analysis by ViPtree.

### Statistical analysis and visualization of data

All statistical data were analyzed by unpaired *t*-test, and the correlation was analyzed by linear regression. GraphPad Prism 8.0.1 software was used for data visualization analysis.

### Supplementary Information

Supplementary Information.

## Data Availability

Data used to support the findings of this study are available from the NCBI GeneBank database and Supplementary Materials.
