# Supplementary material for: Prediction and characterization of prophages of Stenotrophomonas maltophilia reveals a remarkable phylogenetic diversity of prophages
Source: Sci Rep. 2023 Dec 22;13:22941. doi: 10.1038/s41598-023-50449-x (PMC10746704; doi:10.1038/s41598-023-50449-x)
Supplement: Supplementary file 1 — Supplementary Information. [file 41598_2023_50449_MOESM1_ESM.pdf]

Supplementary information to:
Prediction and characterization of prophages of *Stenotrophomonas maltophilia* reveals a remarkable phylogenetic diversity of prophages

Zheng Fang[1#], Man Xu[1#], Shan Shen[1], Weiwei Sun[1], Qing Yu[1], Qingshan Wu[1], Lan Xiang[2] and Qingbei Weng[1, 2]

[1]School of Life Sciences, Guizhou Normal University, Guiyang, Guizhou, PR China, 550025

[2]Qiannan Normal University for Nationalities， Duyun, Guizhou, PR China,558000,

*Corresponding author:

Qingbei Weng, wengqb@126.com, ORCiD: wengqb@126.com

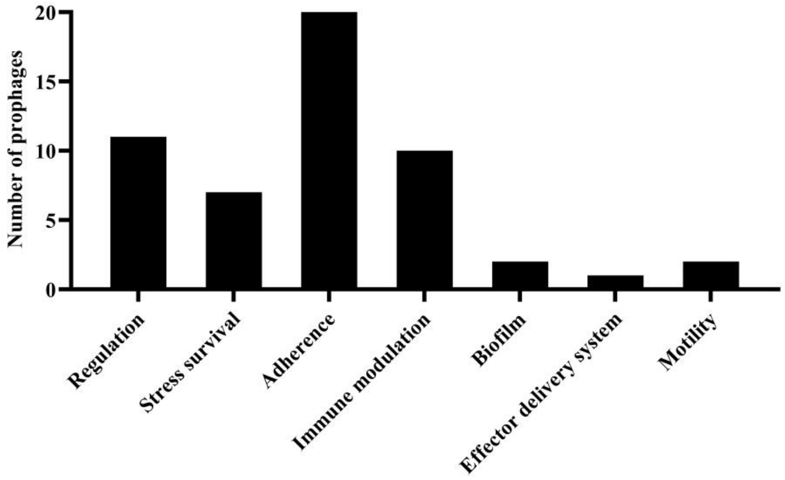

Fig. S1 The classification of VGs in *S. maltophilia* prophage.

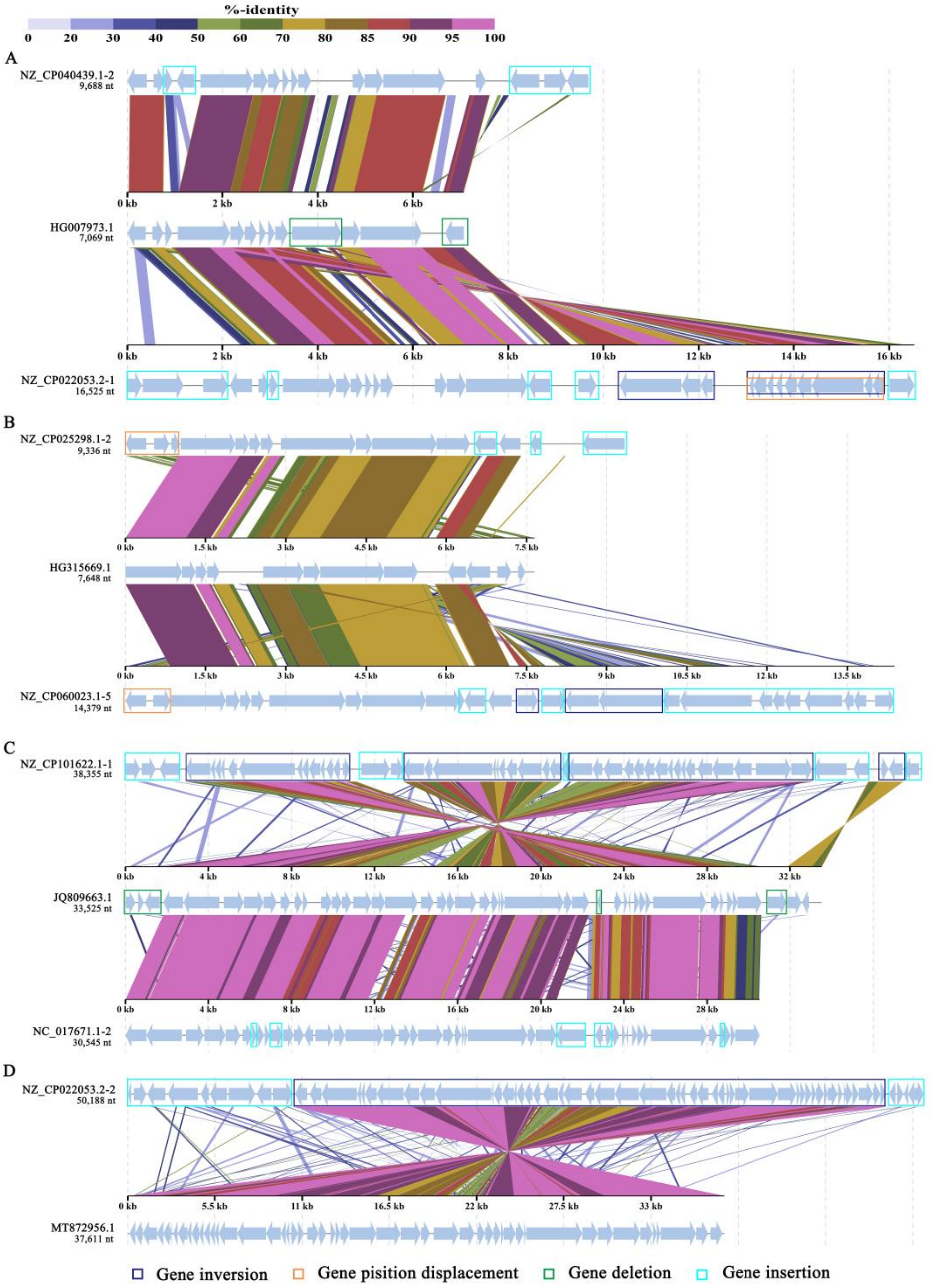

Fig. S2 Comparative genomic analysis of between *S. maltophilia* prophage and *S. maltophilia* phage by Viptree. (A) *S. maltophilia* prophage and *S. maltophilia* phage phiSMA7. (B) *S. maltophilia* prophage and *S. maltophilia* phage phiSMA6. (C) *S. maltophilia* prophage and *S. maltophilia* phage Smp131. (D) *S. maltophilia* prophage and *S. maltophilia* phage phiSHP3. Each arrow indicates an encoding gene, genes predicted to have similar functions are labeled with the same color. The colour spectrum between genomes reflect amino acid identity as shown in the percentage based legend. Regions marked with different clour boxes mainly enumerated the

genes positions and transcription directions of similar genes among phages. blue box**:** gene inversion, orange box: gene position displacement, green box: gene deletion and cyan box: gene insertion.

Table S1: The information of *S. maltophilia* genome and predicted *S. maltophilia* prophages used in this study, N stands for no.

Genome GenBank	GC%	Size (Mb)	*S. maltophilia* strain	Source	Area of isolation	Intact prophage	Genome location	GC %	Size（Kb）	Incomplete prophage	Genome location	GC%	Size（Kb）	Questionable prophage	Genome location	GC%	Size（Kb）
CP050452.1	66.8	4.42	SoD9b	Antarctic soil	Antarctica	CP050452.1-1	2228855-2235236	62.11	6.3	N	N	N	N	CP050452.1-2	1615410-1677436	63.63	62
CP071784.1	66.5	4.39	ZT1	Cholelithiasis patient bile acid	China	N	N	N	N	CP071784.1-1	2946067-2976554	65.53	30.4	N	N	N	N
CP078102.1	66.5	4.52	O1	Bos taurus feces	South Korea	CP078102.1-1	246619-269988	66.27	23.3	CP078102.1-2	551632-566615	59.46	14.9	N	N	N	N
										CP078102.1-3	4311483-4322730	65.22	11.2				
CP091781.1	66.48	4.57	PSKL2	Storm drain water	China	CP091781.1-1	2729380-2754123	64.53	24.7	CP091781.1-2	1036506-1059075	66.45	22.5	N	N	N	N
NC_010943.1	66.3	4.85	K279a	Male patient blood	Britain	NC_010943.1-1	1896567-1926321	29.7	63.32	NC_010943.1 -3	55179-78328	64.53	23.1	NC_010943.1-6	1091933-1113529	66.54	21.5
						NC_010943.1-2	299939-335333	35.3	63.54	NC_010943.1 -4	846502-868954	67.33	22.4				
						N	N	N	N	NC_010943.1 -5	1966393-1983120	65.70	16.7	N	N	N	N
NC_011071.1	66.3	4.57	R551-3	Populus trichocarpa	America	NC_011071.1-1	1024520-1046742	22.2	65.83	N	N	N	N	NC_011071.1-2	2795124-2816171	64.66	21
NC_015947.1	66.9	4.54	JV3	Rhizosphere soil	Brazil	N	N	N	N	N	N	N	N	NC_015947.1-1	255387-270324	62.95	14.9
NC_017671.1	66.8	4.77	D457	Homo	Spain	NC_017671.1-1	286699-306148	19.4	64.85	N	N	N	N	NC_017671.1-3	1767255-17931	62.68	30.5

				clinical											77		
						NC_017671.1-2	3646341-3676885	25.9	64.88					N	N	N	N
NZ_AP021867.1	66.2	4.58	KMM 349	Philippine Sea sponge	Japan	N	N	N	N	NZ_AP021867.1-1	387263-394664	66.59	7.4	N	N	N	N
										NZ_AP021908.1-1	971457-1019511	64.97	48				
NZ_AP021908.1	66.2	4.92	WP1-W18-CRE-01	Wastewater	Japan	N	N	N	N	NZ_AP021908.1-2	1088047-1110855	66.32	22.8	N	N	N	N
										NZ_AP021908.1-3	3749374-3761458	64.83	12				
NZ_CM001824.1	66.4	4.79	EPM1	Homo giardia duodenalis	Portugal	NZ_CM001824.1-1	905086-944750	39.6	65.57	NZ_CM001824.1-5	1604199-1622841	18.6	64.91	N	N	N	N
						NZ_CM001824.1-2	1003361-1026134	22.7	66.27								
						NZ_CM001824.1-3	2352623-2362648	10	61.07								
						NZ_CM001824.1-4	2444582-2467138	22.5	62.55								
						NZ_CP008838.1-1	435158-486726	51.5	62.96	NZ_CP008838.1-5	1028850-1061814	32.9	66.64				
NZ_CP008838.1	66.1	4.99	ATCC 13637	Missing	America	NZ_CP008838.1-2	940494-1020287	79.7	64.83	NZ_CP008838.1-6	4460133-4467327	7.1	65.32	NZ_CP008838.1-7	2008142-2025542	64.61	17.4
						NZ_CP008838.1-3	2474213-2484703	10.4	62.80	N	N	N	N				

						NZ_CP008838.1-4	3606237-3642185	35.9	64.39								
NZ_CP011010.1	66.7	4.8	ISMMS3	Homo Patient1 blood	America	NZ_CP011010.1-1	970762-1014681	43.9	64.70	N	N	N	N	N	N	N	N
						NZ_CP011010.1-2	1070725-1103088	32.3	65.45								
						NZ_CP011010.1-3	1835742-1886513	50.7	64.88								
						NZ_CP011010.1-4	2908945-2937005	28	64.41								
NZ_CP011305.1	66.4	4.51	ISMMS2	Homo Patient1 blood	America	NZ_CP011305.1-1	968646-1000562	31.9	66.6	N	N	N	N	NZ_CP011305.1-2	2216278-2233653	60.04	17.3
NZ_CP014014.1	66.3	4.82	FDAARGOS_92	Homo Respiratory Cx	America	NZ_CP014014.1-1	2651172-2689899	38.7	62.53	NZ_CP014014.1-3	75599-95347	19.7	66.376	NZ_CP014014.1-8	58811-72113	64.61	13.1
						NZ_CP014014.1-2	4780938-4817759	36.8	65.6	NZ_CP014014.1-4	736180-758382	22.2	65.07				
										NZ_CP014014.1-5	920328-947663	27.3	63.92				
						N	N	N	N	NZ_CP014014.1-6	954402-971032	16.6	65.9				
										NZ_CP014014.1-7	4764337-4778926	14.5	64.38				
NZ_CP015612.1	66.3	4.67	OUC_Est10	Soil	China	NZ_CP015612.1-1	1046357-1065439	19	65.73	N	N	N	N	N	N	N	N
						NZ_CP015612.1-	2845460-28717	26.2	64.42								

						2	20										
NZ_CP018756.1	67.4	4.66	AA1	Zea mays root	America	N	N	N	N	N	N	N	N	N	N	N	N
						NZ_CP022053.2-1	628235-644759	16.6	61.83	NZ_CP022053.2-5	1131962-1152240	20.2	65.07				
NZ_CP022053.2	66.3	4.85	FDAARGOS_325	Homo eye	America	NZ_CP022053.2-2	1060294-1110481	50.1	65.68	NZ_CP022053.2-6	1149193-1183247	34	63.08	NZ_CP022053.2-8	4232129-4281236	65.86	49.1
						NZ_CP022053.2-3	1953699-1996271	42.5	66.15	NZ_CP022053.2-7	1359364-1367090	7.7	59.17				
						NZ_CP022053.2-4	4017408-4052113	34.7	65.68	N	N	N	N				
NZ_CP025298.1	66.6	4.74	CSM2	Laboratory sink	Mexico	NZ_CP025298.1-1	287852-301983	14.1	65.30	N	N	N	N	N	N	N	N
						NZ_CP025298.1-2	2308027-2317362	9.3	61.92								
NZ_CP027562.1	65.9	4.93	SJTH1	Wastewater	China	NZ_CP027562.1-1	1031275-1050337	19	66.11	N	N	N	N	N	N	N	N
						NZ_CP028358.1-1	318410-369235	50.8	65.09								
NZ_CP028358.1	66.1	4.74	W18	Oil contaminated soil	China	NZ_CP028358.1-2	1094663-1114599	19.9	65.74	NZ_CP028358.1-5	1867339-1876217	8.8	62.61	N	N	N	N
						NZ_CP028358.1-3	3607285-3651679	44.3	64.07								
						NZ_CP028358.1-4	4162008-4210377	48.3	63.97								
NZ_CP02889	66.5	4.94	AB550	Wastewater	Australia	NZ_CP028899.1-	314025-337509	23.4	64.81	NZ_CP028899.1-	988053-101067	22.6	63.61	N	N	N	N

9.1						1				4	4						
						NZ_CP028899.1-2	2297516-2322084	24.5	63.38	NZ_CP028899.1-5	3652385-3687550	35.1	66.54				
						NZ_CP028899.1-3	4237609-4288145	50.5	64.54	NZ_CP028899.1-6	3690086-3702630	12.5	66.79				
										NZ_CP028899.1-7	4277531-4290990	13.4	64.44				
										NZ_CP028899.1-8	4486109-4492516	6.4	64.90				
NZ_CP029773.1	66.3	4.89	SJTL3	Wastewater	China	NZ_CP029773.1-1	999327-1018357	19	66.30	NZ_CP029773.1-3	954673-974537	19.8	61.43	NZ_CP029773.1-4	1804434-1832920	60.58	24.4
						NZ_CP029773.1-2	2327392-2351873	28.4	59.98								
										NZ_CP031058.1-2	384435-422365	37.9	65.43				
										NZ_CP031058.1-3	3200882-3223760	22.8	65.79				
NZ_CP031058.1	66	5.09	SM 866	Homo organism	India	NZ_CP031058.1-1	2010692-2059957	49.2	63.13	NZ_CP031058.1-4	3228696-3243643	14.9	65.87	N	N	N	N
										NZ_CP031058.1-5	3643531-3664688	21.1	64.62				
										NZ_CP031058.1-6	3915010-3925896	10.8	64.17				
NZ_CP033586.1	66.6	4.5	SVIA2	Soil	Mexico	NZ_CP033586.1-1	2123514-2136946	13.4	60.31	NZ_CP033586.1-2	3558452-3581223	22.7	66.22	N	N	N	N

NZ_CP033829.1	66.6	4.58	FDAARGOS_507	Homo sputum	America	NZ_CP033829.1-1	722098-744561	22.4	66.23	NZ_CP033829.1-2<br>NZ_CP033829.1-3	1474443-1488853<br>3212707-3240213	14.4<br>27.5	64.32<br>65.88	N	N	N	N
NZ_CP033877.1	66.7	4.55	291	Calibrachoa elegans rhizosphere	Brazil	NZ_CP033877.1-1	272584-293101	20.5	64.35	N	N	N	N	N	N	N	N
NZ_CP037858.1	66.5	4.55	X28	Aerobic granular sludge	China	N	N	N	N	NZ_CP037858.1-1<br>NZ_CP037858.1-2	3114967-3137626<br>3870099-3886632	22.6<br>16.5	66.25<br>65.16	NZ_CP037858.1-3	195583-202022	60.65	6.4
NZ_CP040429.1	66.4	4.54	U5	Missing	Germany	NZ_CP040429.1-1<br>NZ_CP040429.1-2<br>NZ_CP040429.1-3	276658-294509<br>1637170-1678714<br>2270713-2276624	17.8<br>41.5<br>5.9	63.63<br>61.76<br>61.16	N	N	N	N	N	N	N	N
NZ_CP040430.1	66.3	4.68	Sm53	Missing	Germany	N	N	N	N	NZ_CP040430.1-1	1097048-1119855	22.8	66.30	NZ_CP040430.1-2	959894-1030045	64.45	70.1
NZ_CP040431.1	4.69	66.3	Sm454	Missing	Germany	NZ_CP040431.1-1	276802-314369	37.5	64.25	NZ_CP040431.1-2<br>NZ_CP040431.1-3<br>NZ_CP040431.1-	1022762-1070816<br>1131574-1161473<br>2341463-23504	48<br>29.9<br>9	64.96<br>66.13<br>63.20	N	N	N	N

										4	88						
NZ_CP040432.1	5.01	65.6	Sm-RA9	Missing	Germany	NZ_CP040432.1-1	1834361-1875001	40.6	64.01	NZ_CP040432.1-2	2890925-2897770	6.8	64.08	NZ_CP040432.1-4	1072495-1098541	59.36	26
										NZ_CP040432.1-3	4587376-4596674	9.2	62.22				
NZ_CP040433.1	66.3	4.68	SKK55	Missing	Germany	N	N	N	N	NZ_CP040433.1-1	934240-956225	21.9	62.85	NZ_CP040433.1-3	2322708-2332723	59.72	10
										NZ_CP040433.1-2	2357276-2367739	10.4	62.37				
NZ_CP040434.1	66.7	4.64	PEG-68	Missing	Germany	NZ_CP040434.1-1	280702-304300	23.5	65.28	N	N	N	N	NZ_CP040434.1-2	3170508-3195381	65.74	24.8
NZ_CP040435.1	66.1	4.85	PEG-42	Missing	Germany	NZ_CP040435.1-1	1053958-1073211	19.2	65.92	NZ_CP040435.1-5	1920811-1938097	17.2	65.05	N	N	N	N
						NZ_CP040435.1-2	1701691-1742460	40.7	63.70	NZ_CP040435.1-6	4257126-4271937	14.8	63.31				
						NZ_CP040435.1-3	2418156-2451960	33.8	60.96	N	N	N	N				
						NZ_CP040435.1-4	4277687-4300979	23.2	65.28								
NZ_CP040436.1	66.4	4.55	PEG-390	Missing	Germany	N	N	N	N	NZ_CP040436.1-1	926796-975063	48.2	64.39	N	N	N	N
										NZ_CP040436.1-2	1726724-1765452	38.7	65.69				
										NZ_CP040436.1-	1827493-18477	20.2	65.14				

										3	44						
NZ_CP040437.1	67.2	4.5	PEG-305	Missing	Germany	N	N	N	N	NZ_CP040437.1-1	179811-189708	9.8	66.33	N	N	N	N
										NZ_CP040437.1-2	799764-806510	6.7	65.66				
										NZ_CP040437.1-3	1019355-1038105	18.7	65.19				
										NZ_CP040437.1-4	2590898-2597820	6.9	65.2				
NZ_CP040438.1	66.1	4.77	PEG-173	Missing	Germany	N	N	N	N	NZ_CP040438.1-1	1821314-1841902	20.5	64.87	NZ_CP040438.1-3	2350211-2357794	61.33	7.5
										NZ_CP040438.1-2	1899546-1912069	12.5	66.42				
NZ_CP040439.1	66.1	5	PEG-141	Missing	Germany	NZ_CP040439.1-1	1765759-1785698	19.9	64.90	NZ_CP040439.1-4	937727-985752	48	64.97	N	N	N	N
						NZ_CP040439.1-2	2559412-2569099	9.6	61.66	NZ_CP040439.1-5	1937250-1948426	11.1	63.99				
						NZ_CP040439.1-3	4367626-4395396	27.7	64.99	NZ_CP040439.1-6	1956673-1979782	23.1	65.80				
						N	N	N	N	NZ_CP040439.1-7	2089874-2109443	19.5	64.97				
										NZ_CP040439.1-8	2159545-2172855	13.3	65.72				
NZ_CP040440.1	66.2	5	ICU331	Clinical	Germany	NZ_CP040440.1-1	246639-299394	52.7	65.17	NZ_CP040440.1-6	1890191-1906918	16.7	65.61	N	N	N	N
						NZ_CP040440.1-	968282-101243	44.1	64.77	NZ_CP040440.1-		30.3	61.22				

						2	7			7	2547533-2577844						
						NZ_CP040440.1-3	1106475-1129063	22.5	66.35	NZ_CP040440.1-8	4517287-4537781	20.4	61.03				
						NZ_CP040440.1-4	2634960-2644615	9.6	61.66	N	N	N	N				
						NZ_CP040440.1-5	3722196-3831252	109	64.78								
NZ_CP043578.1	66	4.78	T50-20	biofilm reactor	China	N	N	N	N	NZ_CP043578.1-1	1006457-1054488	48	64.96	NZ_CP043578.1-2	2281536-2325365	61.11	43.8
NZ_CP044092.1	66.5	4.63	FDAARGOS_649	Homo clinical isolate	America	N	N	N	N	NZ_CP044092.1-1	2227166-2274595	47.4	64.81	N	N	N	N
										NZ_CP044092.1-2	2341070-2370970	29.9	66.09				
										NZ_CP044092.1-3	2951843-2974130	22.2	65.08				
										NZ_CP044092.1-4	2963943-2972239	8.2	66				
										NZ_CP044092.1-5	3147326-3165852	18.5	63.32				
										NZ_CP044092.1-6	3250599-3276138	25.5	63.53				
										NZ_CP044092.1-7	3269056-3276235	7.1	62.67				

NZ_CP047310.1	66.5	4.44	CPBW01	Leptinotarsa decemlineata wings	China	NZ_CP047310.1-1	2146993-2157889	10.8	59.32	N	N	N	N	N	N	N	N
NZ_CP049368.1	66	4.55	MER1	Wastewater	China	NZ_CP049368.1-1	2205753-2228806	23	61.76	NZ_CP049368.1-2	998741-1032555	33.8	65.81	N	N	N	N
										NZ_CP049368.1-3	1776024-1792329	16.3	63.8				
NZ_CP049956.1	66.3	4.93	NCTC10498	Homo feces	China	NZ_CP049956.1-1	1863795-1887896	24.1	59.92	NZ_CP049956.1-4	343379-387519	44.1	64.88	N	N	N	N
						NZ_CP049956.1-2	2360140-2387662	27.5	63.59	NZ_CP049956.1-5	446929-469736	22.8	66.28				
						NZ_CP049956.1-3	3697168-3747691	50.5	64.41	NZ_CP049956.1-6	1444179-1463440	19.2	65.95				
										NZ_CP049956.1-7	2232709-2239477	6.7	64.87				
										NZ_CP049956.1-8	3133618-3150651	17	66.85				
										NZ_CP049956.1-9	3139368-3170771	31.4	66.20				
NZ_CP051467.1	66.4	4.79	NEB515	Missing	America	NZ_CP051467.1-1	1858492-1908635	50.1	66.17	NZ_CP051467.1-5	3332187-3345166	12.9	66.12	NZ_CP051467.1-7	3116263-3138619	64.42	22.3
						NZ_CP051467.1-2	2723171-2740071	16.9	61.40	NZ_CP051467.1-6	4132573-4155346	22.7	66.27				
						NZ_CP051467.1-3	2807210-2834673	27.4	62.19	N	N	N	N				
						NZ_CP051467.1-	3499919-35459	46	65.25								

						4	61										
NZ_CP052863.1	66.5	4.59	CF13	Homo sputum	Australia	NZ_CP052863.1-1	1162525-1204868	42.3	62.53	NZ_CP052863.1-2	3641158-3663729	22.5	66.46	N	N	N	N
										NZ_CP056088.1-2	2549378-2596926	47.5	65.11				
NZ_CP056088.1	66.2	4.82	Oak Ridge strain 02	Nuclear waste	America	NZ_CP056088.1-1	2477880-2496967	19	65.79	NZ_CP056088.1-3	3152281-3165940	13.6	65.83	N	N	N	N
										NZ_CP056088.1-4	3888149-3927146	38.9	64.93				
NZ_CP060021.1	66.1	4.48	UHH_PE13-68-68	Homo respiratory tract	Germany	NZ_CP060021.1-1	2230772-2274537	43.7	61.75	NZ_CP060021.1-2	3629271-3651844	22.5	66.36	NZ_CP060021.1-3	1548263-1570202	62.22	NZ_CP060021.1-3
						NZ_CP060022.1-1	535770-578042	42.2	63.09	NZ_CP060022.1-6	505162-533262	28.1	64.70				
						NZ_CP060022.1-2	752217-771604	19.3	65.18	NZ_CP060022.1-7	1919135-1941011	21.8	65.88				
NZ_CP060022.1	66.7	4.85	UHH_PC240	Homo perineum	Germany	NZ_CP060022.1-3	1326057-1340435	14.3	62.35	NZ_CP060022.1-8	1945006-1968059	23	65.83	N	N	N	N
						NZ_CP060022.1-4	1724501-1763470	38.9	63.36	NZ_CP060022.1-9	4558110-4576099	17.9	65.45				
						NZ_CP060022.1-5	491812-514899	23	64.89	NZ_CP060022.1-10	4844906-4850783	5.8	61.26				
NZ_CP060023.1	66.7	4.86	UHH_PC239	Homo perineum	Germany	NZ_CP060023.1-1	802006-858673	56.6	64.78	NZ_CP060023.1-6	2615424-2638477	23	65.83	N	N	N	N
						NZ_CP060023.1-	1792614-18443	51.7	64.09	NZ_CP060023.1-	2641576-26551	13.6	66.03				

						2	91			7	98						
						NZ_CP060023.1-3	3289283-3312370	23	64.89	NZ_CP060023.1-8	3270920-3296857	25.9	64.63				
						NZ_CP060023.1-4	594433-613820	19.3	65.18	NZ_CP060023.1-9	4576333-4590610	14.2	65.56				
						NZ_CP060023.1-5	1412009-1426387	14.3	62.35	NZ_CP060023.1-10	4843795-4851642	7.8	60.31				
NZ_CP060024.1	66.3	4.79	UHH_677	Homo respiratory tract	Germany	NZ_CP060024.1-1	3722730-3734174	11.4	62.65	NZ_CP060024.1-3	674621-695766	21.1	59.88	N	N	N	N
						NZ_CP060024.1-2	2333248-2378274	45	62.50	NZ_CP060024.1-4	708510-725237	16.7	65.64				
						N	N	N	N	NZ_CP060024.1-5	2547300-2569873	22.5	66.36				
										NZ_CP060024.1-6	4771654-4781081	9.4	59.55				
NZ_CP060025.1	66.3	4.67	UHH_SKK55	Homo respiratory tract	Germany	N	N	N	N	NZ_CP060025.1-1	4653626-4660581	6.9	59.62	NZ_CP060025.1-2	269501-289769	61.51	20.2
														NZ_CP060025.1-3	304517-314532	59.38	10
NZ_CP060026.1	66.3	5.06	UHH_ICU331	ICU	Germany	NZ_CP060026.1-1	790358-843112	52.7	65.16	NZ_CP060026.1-4	1445334-1465828	20.4	61.03	NZ_CP060026.1-9	3301363-3318090	16.7	65.55
						NZ_CP060026.1-2	1016244-1046555	30.3	61.22	NZ_CP060026.1-5	2520427-2564582	44.1	64.77	NZ_CP060026.1-10	4145416-4157245	11.8	65.41
						NZ_CP060026.1-	1744019-18458	101.8	64.79	NZ_CP060026.1-	3244578-32671	22.5	66.35	N	N	N	N

						3	58			6	66						
						N	N	N	N	NZ_CP060026.1-7	3278357-3288675	10.3	61.84				
										NZ_CP060026.1-8	4111527-4119204	7.6	65.41				
NZ_CP060027.1	66.3	4.65	UHH_454	Homo sputum	Germany	NZ_CP060027.1-1	634113-671680	37.5	64.18	NZ_CP060027.1-2	2337167-2367066	29.9	66.13	NZ_CP060027.1-4	3064917-3083747	18.8	61.85
										NZ_CP060027.1-3	2805423-2853359	47.9	64.98				
NZ_CP060259.1	66.3	4.64	JZL8	Clivia soft rot leaves	China	NZ_CP060259.1-1	953089-985543	32.4	64.31	NZ_CP060259.1-3	1988564-2003880	15.3	61.66	N	N	N	N
						NZ_CP060259.1-2	1052774-1072569	19.7	66.47								
NZ_CP065965.1	66.1	5	FDAARGOS_1044	Missing	America	NZ_CP065965.1-1	3135051-3175307	40.2	63.01	NZ_CP065965.1-4	800366-822939	22.5	66.44	NZ_CP065965.1-10	717134-766147	49	64.45
						NZ_CP065965.1-2	4967619-4999409	31.7	65.17	NZ_CP065965.1-5	1193810-1208950	15.1	64.47	NZ_CP065965.1-11	1769569-1783656	14	64.12
						NZ_CP065965.1-3	2240779-2246761	5.9	61.51	NZ_CP065965.1-6	1801337-1822826	21.4	61.62				
										NZ_CP065965.1-7	2643550-2650536	6.9	64.72	N	N	N	N
						N	N	N	N	NZ_CP065965.1-8	4133912-4140814	6.9	65.61				
										NZ_CP065965.1-9	4735464-4761902	26.4	65.60				

NZ_CP067993.1	66.3	4.68	DHHJ	Poultry feathers	China	NZ_CP067993.1-1	2222604-2231964	9.3	60.38	NZ_CP067993.1-3	1909204-1942434	33.2	63.77	N	N	N	N
						NZ_CP067993.1-2	3241035-3288062	47	65.62	NZ_CP067993.1-4	2841271-2852968	11.6	64.07				
NZ_CP077679.1	66.48	4.46	Col1	Pavement soil	South Korea	NZ_CP077679.1-1	996338-1016152	19.8	66.38	N	N	N	N	N	N	N	N
NZ_CP080573.1	66.37	4.82	FZD2	Activated sludge	Poland	NZ_CP080573.1-1	1584878-1627039	42.1	63.67	NZ_CP080573.1-4	998040-1016631	18.5	66.54	N	N	N	N
						NZ_CP080573.1-2	2329255-2340166	10.9	61.24	NZ_CP080573.1-5	1820021-1834204	14.1	66.25				
						NZ_CP080573.1-3	3589858-3614171	24.3	64.21	N	N	N	N				
NZ_CP083454.1	66.4	4.63	XL133	Cucumber rhizosphere	China	NZ_CP083454.1-1	4067671-4109106	41.4	64.30	N	N	N	N	N	N	N	N
NZ_CP088240.1	66.4	4.53	2013-SM24	Drinking water	China	NZ_CP088240.1-1	767650-787448	19.7	66.34	NZ_CP088240.1-2	1496322-1522639	26.3	64.40	NZ_CP088240.1-3	1529378-1546008	16.6	65.90
NZ_CP088241.1	66.3	4.7	2013-SM15	Drinking water	China	NZ_CP088241.1-1	4453178-4478432	25.2	58.83	NZ_CP088241.1-2	261018-273989	12.9	66.07	N	N	N	N
										NZ_CP088241.1-3	1055986-1078793	22.8	66.23				
										NZ_CP088241.1-4	2438269-2445461	7.1	57.79				
										NZ_CP088241.1-5	4114496-4121407	6.9	64.71				
NZ_CP088242.1	67	4.61	2013-SM13	Drinking water	China	NZ_CP088242.1-1	998147-1031698	33.5	64.77	NZ_CP088242.1-3	1791040-1809788	18.7	65.20	N	N	N	N

						NZ_CP088242.1-2	3037718-3047262	9.5	60.36	NZ_CP088242.1-4	2610630-2628005	17.3	62.91				
NZ_CP088243.1	67	4.61	2013-SM12	Drinking water	China	NZ_CP088243.1-1	2143657-2177208	33.5	64.77	NZ_CP088243.1-3	2936632-2955380	18.7	65.20	N	N	N	N
						NZ_CP088243.1-2	4183307-4192851	9.5	60.36	NZ_CP088243.1-4	3756222-3773597	17.3	62.91				
NZ_CP088244.1	66.4	4.58	2013-SM4	Drinking water	China	NZ_CP088244.1-1	2279199-2289059	9.8	60.39	NZ_CP088244.1-3	2293567-2303717	10.1	62.58	N	N	N	N
						NZ_CP088244.1-2	4063857-4099586	35.7	65.47	NZ_CP088244.1-4	2308105-2318594	10.4	62.37				
										NZ_CP088244.1-5	3521058-3528491	7.4	66.85				
						N	N	N	N	NZ_CP088244.1-6	3556883-3565974	9	62.22				
										NZ_CP088244.1-7	4055235-4072527	17.2	63.68				
NZ_CP090418.1	66.03	4.91	WGB211	Shale	China	NZ_CP090418.1-1	1048505-1086056	37.5	66.29	NZ_CP090418.1-2	940906-988958	48	64.97	NZ_CP090418.1-4	2398943-2422570	23.6	60.30
										NZ_CP090418.1-3	1863065-1881415	18.3	64.41	NZ_CP090418.1-5	3930430-3970303	39.8	64.78
NZ_CP090423.1	66.56	4.79	GYH	Biodegradation of	China	NZ_CP090423.1-1	1003319-1015811	12.4	60.49	NZ_CP090423.1-4	2713-27247	24.5	65.88	N	N	N	N

				trichloromethane		NZ_CP090423.1-2<br>NZ_CP090423.1-3	1733223-1764501<br>4286080-4321666	31.2<br>35.5	64.08<br>65.07								
NZ_CP098483.1	66.2	4.83	142	Homo sapiens	China	NZ_CP098483.1-1	2298650-2307483	8.8	59.60	NZ_CP098483.1-2<br>NZ_CP098483.1-3	975917-1023970<br>1100345-1122931	48<br>22.5	64.96<br>66.38	NZ_CP098483.1-4	2322202-2342436	20.2	61.51
NZ_CP101622.1	66.65	4.52	CYZ	Homo sputum	China	NZ_CP101622.1-1<br>NZ_CP101622.1-2	995358-1033712<br>3437368-3457166	38.3<br>19.7	63.6<br>66.34	NZ_CP101622.1-3	2663540-2674387	12.7	66.06	NZ_CP101622.1-4	2693403-2710150	16.7	64.86
NZ_CP102942.1	66.37	4.82	SCAID WND1-2022 (370)	Homo swab from purulent wound	Kazakhstan	NZ_CP102942.1-1<br>NZ_CP102942.1-2	1110850-1137659<br>2381373-2397714	26.8<br>16.3	65.76<br>61.62	NZ_CP102942.1-3	938761-986814	48	64.96	NZ_CP102942.1-4<br>NZ_CP102942.1-5	2451121-2471805<br>3734750-3754016	20.6<br>19.2	61.80<br>64.78
NZ_CP104071.1	66.45	4.42	ACYCb.6H	Freshwater stream	China	N	N	N	N	NZ_CP104071.1-1<br>NZ_CP104071.1-2	369966-378533<br>3304873-3324794	8.5<br>19.9	66.75<br>64.82	N	N	N	N
NZ_CP104169.1	66.46	4.51	HW002Y	Hospital ICU ward	Malaysia	NZ_CP104169.1-1<br>NZ_CP104169.1-2	671102-690926<br>4400889-4436235	19.8<br>35.3	66.43<br>64.79	N	N	N	N	NZ_CP104169.1-3	1895074-1903097	8	61.3

NZ_CP104286.1	66.41	4.67	ACYCa.1J	Freshwater stream	China	N	N	N	N	NZ_CP104286.1-1<br>NZ_CP104286.1-2<br>NZ_CP104286.1-3<br>NZ_CP104286.1-4	819116-825852<br>1040549-1060553<br>1809913-1835557<br>1840549-1857276	6.7<br>20<br>25.6<br>16.7	65.73<br>66.35<br>63.54<br>65.63	N	N	N	N
NZ_CP104287.1	66.48	4.41	ACYCb.1K	Freshwater stream	China	N	N	N	N	NZ_CP104287.1-1	2709954-2727014	17	63.98	N	N	N	N
NZ_CP104288.1	66.51	4.58	ACYCa.2H	Freshwater stream	China	NZ_CP104288.1-1<br>NZ_CP104288.1-2	2260756-2269160<br>3474972-3499933	8.4<br>24.9	62.99<br>64.67	NZ_CP104288.1-3<br>NZ_CP104288.1-4	798020-804849<br>1010891-1045556	6.8<br>34.6	65.61<br>66.16	N	N	N	N
NZ_CP104289.1	66.49	4.44	ACYCd.9D	Freshwater stream	China	NZ_CP104289.1-1<br>NZ_CP104289.1-2	1729177-1755508<br>2261135-2275954	26.3<br>14.8	63.80<br>60.31	NZ_CP104289.1-3	1755396-1764404	9	67.06	N	N	N	N
NZ_CP104290.1	66.57	4.53	ACYCa.6E	Freshwater stream	China	N	N	N	N	NZ_CP104290.1-1	1032485-1055063	22.5	66.44	NZ_CP104290.1-2	3134415-3156064	21.6	64.75
NZ_CP104292.1	66.58	4.51	ACYCe.8N	Freshwater stream	China	N	N	N	N	NZ_CP104292.1-1<br>NZ_CP104292.1-2	999628-1022233<br>2567634-2574622	22.6<br>6.9	66.29<br>64.89	N	N	N	N

										NZ_CP104292.1-3	3924232-3936933	12.7	62.26				
NZ_CP104323.1	66.96	4.42	ACYCc.3B	Freshwater stream	China	NZ_CP104323.1	245975-265349	19.3	65.13	NZ_CP104323.1-2<br>NZ_CP104323.1-3	1892679-1908382<br>1867649-1900750	33.1<br>15.7	65.12<br>65.24	N	N	N	N
NZ_CP104324.1	66.21	4.64	ACYCb.10K	Freshwater stream	China	NZ_CP104324.1-1<br>NZ_CP104324.1-2	2301305-2330715<br>3111887-3125093	29.4<br>13.2	64.51<br>60.14	NZ_CP104324.1-3<br>NZ_CP104324.1-4	2770991-2779727<br>3582840-3600751	8.7<br>17.9	65.24<br>64.39	N	N	N	N
NZ_CP104863.1	66.12	4.96	CW002SM	Clinical waste	Malaysia	NZ_CP104863.1-1	1919878-1963452	43.5	64.57	NZ_CP104863.1-2<br>NZ_CP104863.1-3	3727930-3750651<br>3803855-3851790	22.7<br>47.9	66.24<br>64.99	NZ_CP104863.1-4	3119681-3139742	20	57.22
NZ_CP106759.1	66.94	4.12	SG.Y2	Sempervivum tectorum rhizosphere	China	NZ_CP106759.1-1<br>NZ_CP106759.1-2	2050084-2063158<br>2460397-2487770	13<br>27.3	63.29<br>64.85	N	N	N	N	N	N	N	N
NZ_LR134301.1	66.3	4.59	NCTC13014	Missing	Britain	NZ_LR134301.1-1<br>NZ_LR134301.1-2	1220648-1228169<br>1901756-1933682	7.5<br>31.9	61.41<br>64.27	NZ_LR134301.1-3<br>NZ_LR134301.1-4<br>NZ_LR134301.1-5	1858084-1879923<br>2516674-2535719<br>2579065-2592666	21.8<br>19<br>13.6	65.53<br>65.25<br>60.73	N	N	N	N
NZ_LR13432	66.8	4.56	NCTC10259	Missing	Britain	NZ_LR134324.1-	2764709-28043	39.6	65.61	NZ_LR134324.1-	2756200-27735	17.3	64.42	N	N	N	N

4.1						1	70			3	34						
						NZ_LR134324.1-2	2065829-2085215	19.3	65.16								
NZ_LS483377.1	66.6	4.48	NCTC10258	Homo cerebrospinal fluid	Britain	NZ_LS483377.1-1	2217403-2245413	39.1	62.90	NZ_LS483377.1-2	1014075-1034083	20	66.10	NZ_LS483377.1-3	1911582-1950744	28	64.48
NZ_LS483406.1	66.4	4.66	NCTC10498	Homo oropharyngeal swab	Britain	NZ_LS483406.1-1	1036379-1059106	22.7	66.20	NZ_LS483406.1-2	1648900-1669756	20.8	65.12	N	N	N	N
						NZ_LT906480.1-1	246209-297209	51	64.92	NZ_LT906480.1-6	24797-51235	26.4	65.60				
						NZ_LT906480.1-2	994240-1074033	79.7	64.83	NZ_LT906480.1-7	2063165-2077252	14	64.12				
NZ_LT906480.1	66.1	5	NCTC10257	Homo mouth	Britain	NZ_LT906480.1-3	1093964-1116537	22.5	66.44	NZ_LT906480.1-8	3428647-3453743	25	62.47	N	N	N	N
						NZ_LT906480.1-4	2534375-2540357	5.9	61.51	N	N	N	N				
						NZ_LT906480.1-5	3447458-3468903	21.4	64.19								
NZ_OU943334.1	66.2	4.84	1800	Industrial effluent	Algeria	NZ_OU943334.1-1	1068442-1088261	19.8	66.40	NZ_OU943334.1-2	1952804-1966824	14	63.48	NZ_OU943334.1-4	664913-718129	53.2	65.54
										NZ_OU943334.1-3	4269554-4294454	24.9	62.46	N	N	N	N

Table S2: The blastn result of intact *S. maltophilia* prophages, N stands for no.

Intact prophage	BLASTn results	PHARTER results
CP050452.1-1	*Stenotrophomonas* phage phiSHP2, complete genome	NC_015586 *Stenotrophomonas* phage phiSHP2
CP078102.1-1	N	NC_027995 *Escherichia* phage vB_EcoM_ECO1230-10
CP091781.1-1	N	NC_019708 *Enterobacteria* phage mEp235
NC_010943.1-1	N	NC_018282 *Pseudomonas* phage MP1412
NC_010943.1-2	N	NC_009234 *Burkholderia* phage phiE202 chromosome
NC_011071.1-1	N	NC_019722 *Vibrio* phage vB_VpaM_MAR
NC_017671.1-1	N	NC_013055 *Burkholderia* phage KS9
NC_017671.1-2	N	NC_007145 *Burkholderia* prophage phi52237
NZ_CM001824.1-1	N	NC_025431 *Mesorhizobium* phagevB_MloP_Lo5R7ANS
NZ_CM001824.1-2	N	NC_027995 *Escherichia* phage vB_EcoM_ECO1230-10
NZ_CM001824.1-3	*Stenotrophomonas* phage phiSHP2, complete genome	NC_015586 *Stenotrophomonas* phage phiSHP2
NZ_CM001824.1-4	N	NC_015586 *Stenotrophomonas* phage phiSHP2
NZ_CP008838.1-1	N	NC_016164 *Synechococcus* phage S-CBS1
NZ_CP008838.1-2	N	NC_006548 *Pseudomonas* phage B3
NZ_CP008838.1-3	*Stenotrophomonas* phage phiSHP2, complete genome	NC_015586 *Stenotrophomonas* phage phiSHP2
NZ_CP008838.1-4	N	NC_023588 *Stenotrophomonas* phage Smp131
NZ_CP011010.1-1	N	NC_025431 *Mesorhizobium* phagevB_MloP_Lo5R7ANS
NZ_CP011010.1-2	N	NC_027995 *Escherichia* phage vB_EcoM_ECO1230-10
NZ_CP011010.1-3	N	NC_024365 *Pseudomonas* phage phiPSA1
NZ_CP011010.1-4	N	NC_004902 *Xanthomonas* phage Xp10
NZ_CP011305.1-1	N	NC_027995 *Escherichia* phage vB_EcoM_ECO1230-10

NZ_CP014014.1-1	N	NC_011589 *Stenotrophomonas* phage S1
NZ_CP014014.1-2	*Rhizobium* phage RHEph01, complete genome	NC_025431 *Mesorhizobium* phagevB_MloP_Lo5R7ANS
NZ_CP015612.1-1	*Pseudomonas* phage Persinger, complete genome	NC_027995 *Escherichia* phage vB_EcoM_ECO1230-10
NZ_CP015612.1-2	N	NC_007709 *Xanthomonas* oryzae phage OP1 DNA
NZ_CP022053.2-1	*Stenotrophomonas* phage phiSMA7 proviral complete genome	NC_021569 *Stenotrophomonas* phage phiSMA7
NZ_CP022053.2-2	*Stenotrophomonas* phage phiSHP3, complete genome	NC_028667 *Pseudomonas* phage vB_PaeS_PM105
NZ_CP022053.2-3	N	NC_027995 *Escherichia* phage vB_EcoM_ECO1230-10
NZ_CP022053.2-4	N	NC_028667 *Pseudomonas* phage vB_PaeS_PM105
NZ_CP025298.1-1	N	NC_003309 *Burkholderia* phage phiE125
NZ_CP025298.1-2	*Stenotrophomonas* phage phiSMA6 proviral complete genome	NC_001396 *Xanthomonas* phage Cf1c
NZ_CP027562.1-1	N	NC_027995 *Escherichia* phage vB_EcoM_ECO1230-10
NZ_CP028358.1-1	*Stenotrophomonas* phage phiSHP3, complete genome	NC_028667 *Pseudomonas* phage vB_PaeS_PM105
NZ_CP028358.1-2	N	NC_027995 *Escherichia* phage vB_EcoM_ECO1230-10
NZ_CP028358.1-3	N	NC_011589 *Stenotrophomonas* phage S1
NZ_CP028358.1-4	N	NC_019933 *Xanthomonas* phage CP1
NZ_CP028899.1-1	N	NC_013055 *Burkholderia* phage KS9
NZ_CP028899.1-2	N	NC_021569 *Stenotrophomonas* phage phiSMA7
NZ_CP028899.1-3	N	NC_004902 *Xanthomonas* phage Xp10
NZ_CP029773.1-1	N	NC_027995 *Escherichia* phage vB_EcoM_ECO1230-10
NZ_CP029773.1-2	*Stenotrophomonas* phage phiSHP2, complete genome	NC_019708 *Enterobacteria* phage mEp235
NZ_CP031058.1-1	N	NC_013599 *Xylella* phage Xfas53
NZ_CP033586.1-1	N	NC_015586 *Stenotrophomonas* phage phiSHP2
NZ_CP033829.1-1	N	NC_027995 *Escherichia* phage vB_EcoM_ECO1230-10
NZ_CP033877.1-1	N	NC_027995 Escherichia phage vB_EcoM_ECO1230-10
NZ_CP040429.1-1	N	NC_013055 *Burkholderia* phage KS9
NZ_CP040429.1-2	N	NC_009543 *Xanthomonas* phage Xop411

NZ_CP040429.1-3	*Stenotrophomonas* phage phiSHP2, complete genome	NC_015586 *Stenotrophomonas* phage phiSHP2
NZ_CP040431.1-1	N	NC_023588 *Stenotrophomonas* phage Smp131
NZ_CP040432.1-1	N	NC_011589 *Stenotrophomonas* phage S1
NZ_CP040434.1-1	N	NC_005284 *Burkholderia* Bacteriophage phi1026b
NZ_CP040435.1-1	N	NC_041926 *Escherichia* phage vB_EcoM_ECOO78
NZ_CP040435.1-2	N	NC_011589 *Stenotrophomonas* phage S1
NZ_CP040435.1-3	N	NC_043029 *Stenotrophomonas* phage phiSMA6
NZ_CP040435.1-4	*Pseudomonas* phage phiAH14a endogenous virus, complete genome	NC_004902 *Xanthomonas* phage Xp10
NZ_CP040439.1-1	N	NC_013055 *Burkholderia* phage KS9
NZ_CP040439.1-2	*Stenotrophomonas* phage phiSMA7 proviral complete genome	NC_021569 *Stenotrophomonas* phage phiSMA7
NZ_CP040439.1-3	N	NC_013055 *Burkholderia* phage KS9
NZ_CP040440.1-1	N	NC_023588 *Stenotrophomonas* phage Smp131
NZ_CP040440.1-2	N	NC_025431 *Mesorhizobium* phagevB_MloP_Lo5R7ANS
NZ_CP040440.1-3	N	NC_027995 *Escherichia* phage vB_EcoM_ECO1230-10
NZ_CP040440.1-4	*Stenotrophomonas* phage phiSMA7 proviral complete genome	NC_021569 *Stenotrophomonas* phage phiSMA7
NZ_CP040440.1-5	N	NC_011589 *Stenotrophomonas* phage S1
NZ_CP047310.1-1	*Stenotrophomonas* phage phiSMA7 proviral complete genome	NC_021569 *Stenotrophomonas* phage phiSMA7
NZ_CP049368.1-1	*Stenotrophomonas* phage phiSHP2, complete genome	NC_015586 *Stenotrophomonas* phage phiSHP2
NZ_CP049956.1-1	*Stenotrophomonas* phage phiSHP2, complete genome	NC_015586 *Stenotrophomonas* phage phiSHP2
NZ_CP049956.1-2	N	NC_019708 *Enterobacteria* phage mEp235
NZ_CP049956.1-3	N	NC_003309 *Burkholderia* phage phiE125
NZ_CP051467.1-1	N	NC_005284 *Burkholderia* Bacteriophage phi1026b
NZ_CP051467.1-2	*Stenotrophomonas* phage phiSHP2, complete genome	NC_015586 *Stenotrophomonas* phage phiSHP2
NZ_CP051467.1-3	N	NC_015586 *Stenotrophomonas* phage phiSHP2
NZ_CP051467.1-4	N	NC_011373 *Pseudomonas* phage PAJU2
NZ_CP052863.1-1	N	NC_011589 *Stenotrophomonas* phage S1

NZ_CP056088.1-1	N	NC_027995 *Escherichia* phage vB_EcoM_ECO1230-10
NZ_CP060021.1-1	N	NC_011589 *Stenotrophomonas* phage S1
NZ_CP060022.1-1	N	NC_011589 *Stenotrophomonas* phage S1
NZ_CP060022.1-2	N	NC_013055 *Burkholderia* phage KS9
NZ_CP060022.1-3	*Stenotrophomonas* phage phiSMA6 proviral complete genome	NC_043029 *Stenotrophomonas* phage phiSMA6
NZ_CP060022.1-4	N	NC_011589 *Stenotrophomonas* phage S1
NZ_CP060022.1-5	N	NC_015465 *Synechococcus* phage S-CBS3
NZ_CP060023.1-1	N	NC_011589 *Stenotrophomonas* phage S1
NZ_CP060023.1-2	N	NC_011589 *Stenotrophomonas* phage S1
NZ_CP060023.1-3	N	NC_042115 *Pseudomonas* phage vB_PaeS_PAO1_Ab19
NZ_CP060023.1-4	N	NC_005284 *Burkholderia* Bacteriophage phi1026b
NZ_CP060023.1-5	*Stenotrophomonas* phage phiSMA6 proviral complete genome	NC_043029 *Stenotrophomonas* phage phiSMA6
NZ_CP060024.1-1	N	NC_021569 *Stenotrophomonas* phage phiSMA7
NZ_CP060024.1-2	N	NC_011589 *Stenotrophomonas* phage S1
NZ_CP060026.1-1	N	NC_023588 *Stenotrophomonas* phage Smp131
NZ_CP060026.1-2	N	NC_004813 *Enterobacteria* phage BP-4795
NZ_CP060026.1-3	N	NC_011589 *Stenotrophomonas* phage S1
NZ_CP060027.1-1	N	NC_023588 *Stenotrophomonas* phage Smp131
NZ_CP060259.1-1	N	NC_019710 *Enterobacteria* phage HK140
NZ_CP060259.1-2	N	NC_027995 *Escherichia* phage vB_EcoM_ECO1230-10
NZ_CP065965.1-1	N	NC_016164 *Synechococcus* phage S-CBS1
NZ_CP065965.1-2	N	NC_023588 *Stenotrophomonas* phage Smp131
NZ_CP065965.1-3	*Stenotrophomonas* phage phiSHP2, complete genome	NC_015586 *Stenotrophomonas* phage phiSHP2
NZ_CP067993.1-1	*Stenotrophomonas* phage phiSMA6 proviral complete genome	NC_043029 *Stenotrophomonas* phage phiSMA6
NZ_CP067993.1-2	N	NC_006552 *Pseudomonas* phage F116
NZ_CP077679.1-1	N	NC_027995 *Escherichia* phage vB_EcoM_ECO1230-10

NZ_CP080573.1-1	N	NC_011589 *Stenotrophomonas* phage S1
NZ_CP080573.1-2	*Stenotrophomonas* phage phiSHP2, complete genome	NC_015586 *Stenotrophomonas* phage phiSHP2
NZ_CP080573.1-3	N	NC_011373 *Pseudomonas* phage PAJU2
NZ_CP083454.1-1	N	NC_023588 *Stenotrophomonas* phage Smp131
NZ_CP088240.1-1	N	NC_027995 *Escherichia* phage vB_EcoM_ECO1230-10
NZ_CP088241.1-1	*Stenotrophomonas* phage phiSHP2, complete genome	NC_015586 *Stenotrophomonas* phage phiSHP2
NZ_CP088242.1-1	N	NC_023588 *Stenotrophomonas* phage Smp131
NZ_CP088242.1-2	*Stenotrophomonas* phage phiSMA6 proviral complete genome	NC_043029 *Stenotrophomonas* phage phiSMA6
NZ_CP088243.1-1	N	NC_023588 *Stenotrophomonas* phage Smp131
NZ_CP088243.1-2	*Stenotrophomonas* phage phiSMA6 proviral complete genome	NC_043029 *Stenotrophomonas* phage phiSMA6
NZ_CP088244.1-1	*Stenotrophomonas* phage phiSMA7 proviral complete genome	NC_021569 *Stenotrophomonas* phage phiSMA7
NZ_CP088244.1-2	N	NC_019933 *Xanthomonas* phage CP1
NZ_CP090418.1-1	N	NC_027995 *Escherichia* phage vB_EcoM_ECO1230-10
NZ_CP090423.1-1	*Stenotrophomonas* phage PSH1, complete sequence	NC_043029 *Stenotrophomonas* phage phiSMA6
NZ_CP090423.1-2	N	NC_047909 *Faecalibacterium* phage FP_Brigit
NZ_CP090423.1-3	N	NC_019710 *Enterobacteria* phage HK140
NZ_CP098483.1-1	*Stenotrophomonas* phage phiSHP2, complete genome	NC_015586 *Stenotrophomonas* phage phiSHP2
NZ_CP101622.1-1	N	NC_023588 *Stenotrophomonas* phage Smp131
NZ_CP101622.1-2	N	NC_027995 *Escherichia* phage vB_EcoM_ECO1230-10
NZ_CP102942.1-1	N	NC_041926 *Escherichia* phage vB_EcoM_ECOO78
NZ_CP102942.1-2	*Stenotrophomonas* phage phiSMA7 proviral complete genome	NC_021569 *Stenotrophomonas* phage phiSMA7
NZ_CP104169.1-1	N	NC_027995 *Escherichia* phage vB_EcoM_ECO1230-10
NZ_CP104169.1-2	N	NC_023588 *Stenotrophomonas* phage Smp131
NZ_CP104288.1-1	*Stenotrophomonas* phage phiSMA7 proviral complete genome	NC_021569 *Stenotrophomonas* phage phiSMA7
NZ_CP104288.1-2	N	NC_019708 *Enterobacteria* phage mEp235
NZ_CP104289.1-1	N	NC_019708 *Enterobacteria* phage mEp235

NZ_CP104289.1-2	N	NC_043029 *Stenotrophomonas* phage phiSMA6
NZ_CP104323.1-1	N	NC_005284 *Burkholderia* Bacteriophage phi1026b
NZ_CP104324.1-1	N	NC_019708 *Enterobacteria* phage mEp235
NZ_CP104324.1-2	N	NC_043029 *Stenotrophomonas* phage phiSMA6
NZ_CP104863.1-1	N	NC_011373 *Pseudomonas* phage PAJU2
NZ_CP106759.1-1	N	NC_007189 *Stenotrophomonas* phage phiSMA9
NZ_CP106759.1-2	N	NC_016765 *Pseudomonas* phage PMG1
NZ_LR134301.1-1	*Stenotrophomonas* phage phiSMA6 proviral complete genome	NC_007189 *Stenotrophomonas* phage phiSMA9
NZ_LR134301.1-2	N	NC_011589 *Stenotrophomonas* phage S1
NZ_LR134324.1-1	N	NC_025431 *Mesorhizobium* phagevB_MloP_Lo5R7ANS
NZ_LR134324.1-2	N	NC_005284 *Burkholderia* Bacteriophage phi1026b
NZ_LS483377.1-1	N	NC_015586 *Stenotrophomonas* phage phiSHP2
NZ_LS483406.1-1	N	NC_027995 *Escherichia* phage vB_EcoM_ECO1230-10
NZ_LT906480.1-1	N	NC_023588 *Stenotrophomonas* phage Smp131
NZ_LT906480.1-2	N	NC_028667 *Pseudomonas* phage vB_PaeS_PM105
NZ_LT906480.1-3	N	NC_027995 *Escherichia* phage vB_EcoM_ECO1230-10
NZ_LT906480.1-4	*Stenotrophomonas* phage phiSHP2, complete genome	NC_015586 *Stenotrophomonas* phage phiSHP2
NZ_LT906480.1-5	N	NC_016164 *Synechococcus* phage S-CBS1
NZ_OU943334.1-1	N	NC_027995 *Escherichia* phage vB_EcoM_ECO1230-10

Table S3: The pairwise ANI clustering result statistics of *S. maltophilia* genomes and *S. maltophilia* prophage genomes, respectively.

Genome GenBank	Host ANI results	Intact prophage	Intact prophage ANI results	Sourse
CP050452.1	I	CP050452.1-1	a	Environmental setting
CP071784.1	I	NZ_CP104324.1-1	c	Environmental setting
NZ_CP040436.1	I	NZ_CP104324.1-2	l	Environmental setting
NZ_CP104071.1	I	NZ_CP011305.1-1	b	Clinical setting
NZ_CP104324.1	I	NZ_CP060259.1-1	c	Environmental setting
NZ_CP104287.1	I	NZ_CP060259.1-2	b	Environmental setting
NZ_CP011305.1	II	NZ_CP047310.1-1	l	Environmental setting
NZ_CP060259.1	II	NZ_CP077679.1-1	b	Environmental setting
NZ_CP047310.1	II	NZ_CP104169.1-1	b	Clinical setting
NZ_CP077679.1	II	NZ_CP104169.1-2	b	Clinical setting
NZ_CP104169.1	II	NZ_OU943334.1-1	b	Environmental setting
NZ_OU943334.1	II	NZ_CP015612.1-1	b	Environmental setting
NZ_CP015612.1	II	NZ_CP015612.1-2	c	Environmental setting
NZ_CP027562.1	II	NZ_CP027562.1-1	b	Environmental setting
NZ_CP090418.1	II	NZ_CP090418.1-1	b	Environmental setting
NZ_CP031058.1	II	NZ_CP031058.1-1	k	Clinical setting
NZ_CP067993.1	II	NZ_CP067993.1-1	l	Environmental setting
NZ_CP043578.1	II	NZ_CP067993.1-2	f	Environmental setting
NZ_CP040438.1	II	NZ_CP040439.1-1	c	Unknown
NZ_CP040439.1	II	NZ_CP040439.1-2	l	Unknown
NZ_CP029773.1	II	NZ_CP040439.1-3	h	Unknown
NZ_CP080573.1	II	NZ_CP029773.1-1	b	Environmental setting

NZ_CP028358.1	II	NZ_CP029773.1-2	l	Environmental setting
NZ_CP056088.1	II	NZ_CP080573.1-1	g	Environmental setting
NZ_LR134301.1	II	NZ_CP080573.1-2	l	Environmental setting
NZ_CP040435.1	II	NZ_CP080573.1-3	h	Environmental setting
NZ_CP011010.1	III	NZ_CP028358.1-1	e	Environmental setting
NC_011071.1	IV	NZ_CP028358.1-2	b	Environmental setting
NZ_CP040432.1	IV	NZ_CP028358.1-3	g	Environmental setting
NZ_AP021867.1	IV	NZ_CP028358.1-4	d	Environmental setting
CP078102.1	V	NZ_CP056088.1-1	b	Environmental setting
NZ_AP021908.1	V	NZ_LR134301.1-1	l	Unknown
NZ_CP060021.1	V	NZ_LR134301.1-2	g	Unknown
NZ_CP049956.1	V	NZ_CP040435.1-1	b	Unknown
NZ_CP040430.1	V	NZ_CP040435.1-2	g	Unknown
NZ_LS483377.1	V	NZ_CP040435.1-3	l	Unknown
NZ_CP022053.2	V	NZ_CP040435.1-4	d	Unknown
NZ_CP033586.1	V	NZ_CP011010.1-1	j	Clinical setting
NZ_CP044092.1	V	NZ_CP011010.1-2	b	Clinical setting
NZ_CP104290.1	V	NZ_CP011010.1-3	c	Clinical setting
NZ_CP104286.1	V	NZ_CP011010.1-4	c	Clinical setting
NZ_CP040431.1	V	NC_011071.1-1	b	Environmental setting
NZ_CP060027.1	V	NZ_CP040432.1-1	g	Unknown
NZ_LS483406.1	V	CP078102.1-1	b	Environmental setting
NZ_CP033829.1	V	NZ_CP060021.1-1	g	Clinical setting
NZ_CP052863.1	V	NZ_CP049956.1-1	l	Clinical setting
NZ_CP040440.1	V	NZ_CP049956.1-2	h	Clinical setting
NZ_CP060026.1	V	NZ_CP049956.1-3	h	Clinical setting

NZ_CP014014.1	V	NZ_LS483377.1-1	l	Clinical setting
NZ_CP008838.1	V	NZ_CP022053.2-1	l	Clinical setting
NZ_LT906480.1	V	NZ_CP022053.2-2	e	Clinical setting
NZ_CP065965.1	V	NZ_CP022053.2-3	b	Clinical setting
NC_010943.1	V	NZ_CP022053.2-4	e	Clinical setting
NZ_CM001824.1	V	NZ_CP033586.1-1	l	Environmental setting
NZ_CP051467.1	V	NZ_CP040431.1-1	b	Unknown
NZ_CP088240.1	V	NZ_CP060027.1-1	b	Clinical setting
NZ_CP088241.1	V	NZ_LS483406.1-1	b	Clinical setting
NZ_CP104863.1	V	NZ_CP033829.1-1	b	Clinical setting
NZ_CP098483.1	V	NZ_CP052863.1-1	g	Clinical setting
NZ_CP101622.1	V	NZ_CP040440.1-1	b	Clinical setting
NZ_CP104288.1	V	NZ_CP040440.1-2	j	Clinical setting
NZ_CP104292.1	V	NZ_CP040440.1-3	b	Clinical setting
NZ_CP102942.1	V	NZ_CP040440.1-4	l	Clinical setting
CP091781.1	V	NZ_CP040440.1-5	g	Clinical setting
NZ_CP083454.1	V	NZ_CP060026.1-1	b	Clinical setting
NZ_CP060024.1	V	NZ_CP060026.1-2	m	Clinical setting
NZ_CP037858.1	V	NZ_CP060026.1-3	g	Clinical setting
NC_015947.1	VI	NZ_CP014014.1-1	g	Clinical setting
NZ_CP025298.1	VI	NZ_CP014014.1-2	j	Clinical setting
NZ_CP033877.1	VI	NZ_CP008838.1-1	i	Unknown
NC_017671.1	VI	NZ_CP008838.1-2	e	Unknown
NZ_CP028899.1	VI	NZ_CP008838.1-3	l	Unknown
NZ_CP040434.1	VI	NZ_CP008838.1-4	b	Unknown
NZ_LR134324.1	VI	NZ_LT906480.1-1	b	Clinical setting

NZ_CP104323.1	VI	NZ_LT906480.1-2	e	Clinical setting
NZ_CP060022.1	VI	NZ_LT906480.1-3	b	Clinical setting
NZ_CP060023.1	VI	NZ_LT906480.1-4	l	Clinical setting
NZ_CP040437.1	VI	NZ_LT906480.1-5	i	Clinical setting
NZ_CP088243.1	VI	NZ_CP065965.1-1	i	Unknown
NZ_CP088242.1	VI	NZ_CP065965.1-2	b	Unknown
NZ_CP088244.1	VI	NZ_CP065965.1-3	l	Unknown
NZ_CP104289.1	VI	NC_010943.1-1	i	Clinical setting
NZ_CP060025.1	VI	NC_010943.1-2	b	Clinical setting
NZ_CP090423.1	VII	NZ_CM001824.1-1	j	Clinical setting
NZ_CP040433.1	VIII	NZ_CM001824.1-2	b	Clinical setting
NZ_CP018756.1	IX	NZ_CM001824.1-3	l	Clinical setting
NZ_CP040429.1	X	NZ_CM001824.1-4	l	Clinical setting
NZ_CP049368.1	X	NZ_CP051467.1-1	h	Unknown
NZ_CP106759.1	XI	NZ_CP051467.1-2	l	Unknown
		NZ_CP051467.1-3	l	Unknown
		NZ_CP051467.1-4	h	Unknown
		NZ_CP088240.1-1	b	Environmental setting
		NZ_CP088241.1-1	l	Environmental setting
		NZ_CP104863.1-1	h	Clinical setting
		NZ_CP098483.1-1	l	Clinical setting
		NZ_CP101622.1-1	b	Clinical setting
		NZ_CP101622.1-2	b	Clinical setting
		NZ_CP104288.1-1	l	Environmental setting
		NZ_CP104288.1-2	c	Environmental setting
		NZ_CP102942.1-1	b	Clinical setting

NZ_CP102942.1-2	l	Clinical setting
CP091781.1-1	c	Environmental setting
NZ_CP083454.1-1	b	Environmental setting
NZ_CP060024.1-1	l	Clinical setting
NZ_CP060024.1-2	g	Clinical setting
NZ_CP025298.1-1	h	Environmental setting
NZ_CP025298.1-2	l	Environmental setting
NZ_CP033877.1-1	h	Environmental setting
NC_017671.1-1	h	Clinical setting
NC_017671.1-2	b	Clinical setting
NZ_CP028899.1-1	h	Environmental setting
NZ_CP028899.1-2	l	Environmental setting
NZ_CP028899.1-3	d	Environmental setting
NZ_CP040434.1-1	h	Unknown
NZ_LR134324.1-1	j	Unknown
NZ_LR134324.1-2	h	Unknown
NZ_CP104323.1-1	h	Environmental setting
NZ_CP060022.1-1	g	Clinical setting
NZ_CP060022.1-2	h	Clinical setting
NZ_CP060022.1-3	l	Clinical setting
NZ_CP060022.1-4	g	Clinical setting
NZ_CP060022.1-5	i	Clinical setting
NZ_CP060023.1-1	g	Clinical setting
NZ_CP060023.1-2	g	Clinical setting
NZ_CP060023.1-3	i	Clinical setting
NZ_CP060023.1-4	h	Clinical setting

	NZ_CP060023.1-5	l	Clinical setting
	NZ_CP088243.1-1	b	Environmental setting
	NZ_CP088243.1-2	l	Environmental setting
	NZ_CP088242.1-1	b	Environmental setting
	NZ_CP088242.1-2	l	Environmental setting
	NZ_CP088244.1-1	l	Environmental setting
	NZ_CP088244.1-2	d	Environmental setting
	NZ_CP104289.1-1	h	Environmental setting
	NZ_CP104289.1-2	l	Environmental setting
	NZ_CP090423.1-1	l	Environmental setting
	NZ_CP090423.1-2	c	Environmental setting
	NZ_CP090423.1-3	c	Environmental setting
	NZ_CP040429.1-1	h	Unknown
	NZ_CP040429.1-2	h	Unknown
	NZ_CP040429.1-3	l	Unknown
	NZ_CP049368.1-1	l	Environmental setting
	NZ_CP106759.1-1	l	Environmental setting
	NZ_CP106759.1-2	c	Environmental setting

Table S4: Comparison of whole genome protein levels between *S. maltophilia* prophage and known phage in public database, the yellow marked area indicates that the prophage has the highest similarity to the known phage in public database.

Phage ID	Phage name	Host name	Vfamily	Host group	Numbers	*Stenotrophomonas maltophilia* prophage
NC_049463	*Stenotrophomonas* phage Pokken	*Stenotrophomonas maltophilia*	*Schitoviridae*	*Pseudomonadota*	1	NZ_OU943334.1-1
NC_055838	*Xanthomonas* phage FoX5	*Xanthomonas campestris* pv. campestris	*Myoviridae*	*Pseudomonadota*	6	NZ_CP011305.1-1, NZ_CP022053.2-3, NZ_CP028358.1-2, NZ_CP033829.1-1, NZ_CP102942.1-1, NZ_CP104169.1-1
NC_055836	*Xanthomonas* phage FoX2	*Xanthomonas campestris* pv. campestris	*Myoviridae*	*Pseudomonadota*	9	CP078102.1-1, NZ_CP027562.1-1, NZ_CP029773.1-1, NZ_CP040435.1-1, NZ_CP040440.1-3, NZ_CP060259.1-2, NZ_CP088240.1-1, NZ_CP090418.1-1, NZ_CP101622.1-2
NC_055835	*Xanthomonas* phage FoX1	*Xanthomonas campestris* pv. campestris	*Myoviridae*	*Pseudomonadota*	4	NZ_CM001824.1-2, NZ_CP077679.1-1, NZ_LS483406.1-1, NZ_LT906480.1-3
NC_055837	*Xanthomonas* phage FoX3	*Xanthomonas campestris* pv. campestris	*Myoviridae*	*Pseudomonadota*	4	NC_011071.1-1, NZ_CP011010.1-2, NZ_CP015612.1-1, NZ_CP056088.1-1
NC_047992	*Microbacterium* phage Zeta1847	*Microbacterium paraoxydans*	*Zetavirus*	*Actinomycetota*	6	NZ_CP008838.1-3, NZ_CP011010.1-4, NZ_CP067993.1-2, NZ_CP104288.1-2, NZ_CP104289.1-2, NZ_LR134301.1-1
JX507079	*Acidithiobacillus* phage AcaML1	*Acidithiobacillus caldus* ATCC 51756	*Myoviridae*	*Pseudomonadota*	4	NC_010943.1-1, NZ_CP008838.1-1, NZ_CP065965.1-1, NZ_LT906480.1-5
NC_021347	*Rhodococcus* phage E3	*Rhodococcus*	*Myoviridae*	*Actinomycetota*	2	NZ_CM001824.1-3, NZ_CP051467.1-4
NC_023588	*Stenotrophomonas* phage Smp131	*Stenotrophomonas maltophilia*	*Myoviridae*	*Pseudomonadota*	13	NC_010943.1-2, NC_017671.1-2, NZ_CP008838.1-4, NZ_CP040431.1-1, NZ_CP040440.1-1, NZ_CP060026.1-1, NZ_CP060027.1-1, NZ_CP065965.1-2, NZ_CP083454.1-1, NZ_CP088242.1-1, NZ_CP101622.1-1, NZ_CP104169.1-2, NZ_LT906480.1-1
NC_047793	*Streptomyces* phage PapayaSalad	*Streptomyces venezuelae* ATCC 10712	*Austintatiousvirus*	*Actinomycetota*	1	NZ_CP047310.1-1
NC_054727	*Mycobacterium* phage Phrappuccino	*Mycolicibacterium smegmatis*	*Phrappuccinovirus*	*Actinomycetota*	17	CP050452.1-1, CP091781.1-1, NZ_CP011010.1-3, NZ_CP028899.1-2, NZ_CP028899.1-3, NZ_CP033586.1-1, NZ_CP049368.1-1, NZ_CP049956.1-3, NZ_CP060022.1-5, NZ_CP060026.1-2, NZ_CP060259.1-1, NZ_CP088242.1-2, NZ_CP088244.1-2, NZ_CP090423.1-3, NZ_CP104288.1-1, NZ_CP104324.1-2, NZ_CP106759.1-1
NC_048153	*Streptomyces* phage Austintatious	*Streptomyces venezuelae* ATCC 10712	*Austintatiousvirus*	*Actinomycetota*	1	NZ_CP102942.1-2
NC_047792	*Streptomyces* phage Ididsumtinwong	*Streptomyces venezuelae* ATCC 10712	*Austintatiousvirus*	*Actinomycetota*	1	NZ_CP025298.1-2
NC_018848	*Streptomyces* phage SV1	*Streptomyces venezuelae*	*Picardvirus*	*Actinomycetota*	2	NZ_CP029773.1-2, NZ_CP060023.1-5

NC_015210	*Tsukamurella* phage TPA2	*Tsukamurella paurometabola*	*Siphoviridae*	*Actinomycetota*	1	NZ_CP088244.1-1
NC_051581	*Mycobacterium* phage BirdsNest	*Mycolicibacterium smegmatis* MC2 155	*Bclasvirinae*	*Actinomycetota*	1	NZ_CP080573.1-2
NC_047794	*Streptomyces* phage Picard	*Streptomyces venezuelae* ATCC 10712	*Picardvirus*	*Actinomycetota*	3	NZ_CP040439.1-2, NZ_CP051467.1-3, NZ_CP098483.1-1
NC_019407	*Caulobacter* virus Magneto	*Caulobacter vibrioides*	*Siphoviridae*	*Pseudomonadota*	2	NZ_CP040435.1-3, NZ_CP049956.1-1
NC_021319	*Haloarcula* californiae tailed virus 2	*Haloarcula californiae*	*Saparoviridae*	*Euryarchaeota*	1	NZ_CP022053.2-1
NC_043767	*Mycobacterium* phage TA17A	*Mycolicibacterium smegmatis* MC2 155	*Bclasvirinae*	*Actinomycetota*	1	NZ_CP040429.1-3
NC_048072	*Streptomyces* phage Darolandstone	*Streptomyces platensis*	*Raleighvirus*	*Actinomycetota*	1	NZ_CP090423.1-1
NC_048045	*Caulobacter* phage CcrBL10	*Caulobacter vibrioides* CB15	*Dolichocephalovirina e*	*Pseudomonadota*	1	NZ_CP040439.1-1
NC_019408	*Caulobacter* phage CcrRogue	*Caulobacter vibrioides*	*Siphoviridae*	*Pseudomonadota*	2	NZ_CP051467.1-2, NZ_CP090423.1-2
NC_019405	*Caulobacter* phage phiCbK	*Caulobacter vibrioides*	*Siphoviridae*	*Pseudomonadota*	1	NZ_CP051467.1-1
NC_051583	*Mycobacterium* phage Saguaro	*Mycolicibacterium smegmatis*	*Bclasvirinae*	*Actinomycetota*	1	NZ_LS483377.1-1
NC_008204	*Mycobacterium* phage Qyrzula	-	*Bclasvirinae*	-	1	NZ_CP040440.1-4
NC_019410	*Caulobacter* virus Karma	*Caulobacter vibrioides*	*Siphoviridae*	*Pseudomonadota*	3	NZ_CP015612.1-2, NZ_CP028358.1-4, NZ_CP040435.1-4
NC_054725	*Gordonia* phage Skog	*Gordonia terrae*	*Skogvirus*	*Actinomycetota*	1	NZ_LT906480.1-4
NC_055765	*Gordonia* phage TillyBobJoe	*Gordonia terrae*	*Wizardvirus*	*Actinomycetota*	1	NZ_CP088241.1-1
NC_042035	*Mycobacterium* phage Zemanar	*Mycolicibacterium smegmatis* MC2 155	*Bclasvirinae*	*Actinomycetota*	1	NZ_CM001824.1-4
NC_023686	*Mycobacterium* phage Gadjet	*Mycolicibacterium smegmatis* MC2 155	*Bclasvirinae*	*Actinomycetota*	1	NZ_CP067993.1-1
NC_055026	*Ralstonia* phage Dina	*Ralstonia pseudosolanacearum*	*Dinavirus*	*Pseudomonadota*	14	NC_017671.1-1, NZ_CP025298.1-1, NZ_CP028899.1-1, NZ_CP033877.1-1, NZ_CP040429.1-1, NZ_CP040429.1-2, NZ_CP040434.1-1, NZ_CP040439.1-3, NZ_CP060022.1-2, NZ_CP060023.1-4, NZ_CP080573.1-3, NZ_CP104323.1-1, NZ_CP104863.1-1, NZ_LR134324.1-2
NC_028667	*Pseudomonas* phage vB_PaeS_PM105	*Pseudomonas aeruginosa*	*Siphoviridae*	*Pseudomonadota*	3	NZ_CP022053.2-2, NZ_CP022053.2-4, NZ_CP028358.1-1
NC_002484	*Pseudomonas* phage D3	*Pseudomonas aeruginosa*	*Siphoviridae*	*Pseudomonadota*	1	NZ_CP104324.1-1
NC_016765	*Pseudomonas* phage PMG1	*Pseudomonas aeruginosa*	*Detrevirus*	*Pseudomonadota*	1	NZ_CP106759.1-2
NC_011589	*Stenotrophomonas* phage S1	*Stenotrophomonas maltophilia*	*Siphoviridae*	*Pseudomonadota*	15	NZ_CP014014.1-1, NZ_CP028358.1-3, NZ_CP040432.1-1, NZ_CP040435.1-2, NZ_CP040440.1-5, NZ_CP052863.1-1, NZ_CP060021.1-1, NZ_CP060022.1-1, NZ_CP060022.1-4, NZ_CP060023.1-1, NZ_CP060023.1-2, NZ_CP060024.1-2,

						NZ_CP060026.1-3, NZ_CP080573.1-1, NZ_LR134301.1-2
NC_031129	*Salmonella* phage SJ46	*Salmonella* enterica subsp. enterica serovar Indiana	*Punavirus*	*Pseudomonadota*	1	NZ_CP060024.1-1
NC_006548	*Pseudomonas* phage B3	*Pseudomonas aeruginosa* PAO1	*Beetrevirus*	*Pseudomonadota*	1	NZ_LT906480.1-2
NC_047738	*Rhizobium* phage RHEph01	*Rhizobium etli*	*Autographiviridae*	*Pseudomonadota*	4	NZ_CM001824.1-1, NZ_CP014014.1-2, NZ_CP040440.1-2, NZ_LR134324.1-1
NC_025431	*Mesorhizobium* phage vB_MloP_Lo5R7ANS	*Mesorhizobium loti*	*Autographiviridae*	*Pseudomonadota*	1	NZ_CP011010.1-1
NC_005284	*Burkholderia* phage phi1026b	*Burkholderia pseudomallei* 1026b	*Siphoviridae*	*Pseudomonadota*	2	NZ_CP049956.1-2, NZ_CP104289.1-1
NC_013599	*Xylella* phage Xfas53	*Xylella fastidiosa*	*Unkown*	*Pseudomonadota*	1	NZ_CP031058.1-1

Table S5: Statistics of auxiliary genes encoded by *S. maltophilia* prophage, N stands for no.

ARGs								
Prophage	Genome location	Prophage classification	ARO	Detection criteria	AMR Gene family	Drug class	Resistance mechanism	Identity （%)
NZ_CP104169.1-3	1895074-1903097	Questionable	AAC(6')-Ib9	protein homolog model	AAC(6')	aminoglycoside antibiotic	antibiotic inactivation	100
			AAC(6')-Ib9	protein homolog model	AAC(6')	aminoglycoside antibiotic	antibiotic inactivation	100
			ANT(2'')-Ia	protein homolog model	ANT(2'')	aminoglycoside antibiotic	antibiotic inactivation	100
			APH(6)-Id	protein homolog model	APH(6)	aminoglycoside antibiotic	antibiotic inactivation	99.64
			arr-2	protein homolog model	rifampin ADP-ribosyltransferase (Arr)	rifamycin antibiotic	antibiotic inactivation	78.67
			GES-1	protein homolog model	GES beta-lactamase	carbapenem, cephalosporin, penam	antibiotic inactivation	100
			mphF	protein homolog model	macrolide phosphotransferase (MPH)	macrolide antibiotic	antibiotic inactivation	100
			OXA-10	protein homolog model	OXA beta-lactamase	carbapenem, cephalosporin, penam	antibiotic inactivation	100

			qacEdelta1	protein homolog model	major facilitator superfamily (MFS) antibiotic efflux pump	disinfecting agents and antiseptics	antibiotic efflux	100
			sul1	protein homolog model	sulfonamide resistant sul	sulfonamide antibiotic	antibiotic target replacement	100
			tet(A)	protein homolog model	major facilitator superfamily (MFS) antibiotic efflux pump	tetracycline antibiotic	antibiotic efflux	99.74

VGs

Prophage	Genome location	Prophage classification	Hit	Related VF	VGs type	Source	Area
NZ_CP080573.1-5	1820021-1834204	Incomplete	(fur) ferric iron uptake transcriptional regulator [Fur - Regulation]	Fur	Regulation	Activated sludge	Poland
NZ_CP098483.1-2	975917-1023970	Incomplete	(clpP) ATP-dependent Clp protease proteolytic subunit [ClpP - Stress survival]	ClpP	Stress survival	Homo sapiens	China
NZ_CP098483.1-3	1100345-1122931	Incomplete	(pilZ) type 4 fimbrial biogenesis protein PilZ [Type IV pili - Adherence]	Type IV pili	Adherence	Homo sapiens	China
			(manC) GDP-mannose pyrophosphorylase [Capsule I - Immune modulation]	Capsule I	Immune modulation		
			(algA) phosphomannose isomerase / guanosine 5'-diphospho-D-mannose pyrophosphorylase [Alginate - Biofilm]	Alginate	Biofilm		
			(YE_RS15420) mannose-1-phosphate guanylyltransferase/mannose-6-phosphate isomerase [O-antigen - Immune modulation]	O-antigen	Immune modulation		
NZ_CP102942.1-3	938761-986814	Incomplete	(KP1_RS17280) mannose-1-phosphate guanylyltransferase/mannose-6-phosphate isomerase [Capsule - Immune modulation]	Capsule	Immune modulation	Homo swab from purulent wound	Kazakhstan
			(ACICU_RS00500) phosphomannomutase/phosphoglucomutase [Capsule - Immune modulation]	Capsule	Immune modulation		
			(manCcore) mannose-1-phosphate guanylyltransferase/mannose-6-phosphate isomerase [LPS - Immune modulation]	LPS	Immune modulation		
			(cpsG) phosphomannomutase CpsG [O-antigen - Immune modulation]	O-antigen	Immune modulation		
			(rfbK1) O9 family phosphomannomutase RfbK1 [Capsule - Immune modulation]	Capsule	Immune modulation		
NZ_CP090418.1-2	940906-988958	Incomplete	(clpP) ATP-dependent Clp protease proteolytic subunit [ClpP - Stress survival]	ClpP	Stress survival	Shale	China

NZ_CP090418.1-3	1863065-1881415	Incomplete	(fur) ferric iron uptake transcriptional regulator [Fur - Regulation]	Fur	Regulation	Shale	China
CP091781.1-2	1036506-1059075	Incomplete	(pilZ) type 4 fimbrial biogenesis protein PilZ [Type IV pili - Adherence]	Type IV pili	Adherence	Storm drain water	China
NZ_CP101622.1-3	2663540-2674387	Incomplete	(fur) ferric iron uptake transcriptional regulator [Fur - Regulation]	Fur	Regulation	Homo sputum	China
NZ_CP104071.1-1	369966-378533	Incomplete	(CBU_1594) Coxiella Dot/Icm type IVB secretion system translocated effector [T4SS secreted effectors - Effector delivery system]	T4SS secreted effectors	Effector delivery system	Freshwater stream	China
NZ_CP104288.1-4	1010891-1045556	Incomplete	(pilZ) type 4 fimbrial biogenesis protein PilZ [Type IV pili - Adherence]	Type IV pili	Adherence	Freshwater stream	China
NZ_CP104289.1-3	1755396-1764404	Incomplete	fur) ferric iron uptake transcriptional regulator [Fur - Regulation]	Fur	Regulation	Freshwater stream	China
NZ_CP104290.1-1	1032485-1055063	Incomplete	(pilZ) type 4 fimbrial biogenesis protein PilZ [Type IV pili -Adherence]	Type IV pili	Adherence	Freshwater stream	China
NZ_CP104292.1-1	999628-1022233	Incomplete	(pilZ) type 4 fimbrial biogenesis protein PilZ [Type IV pili -Adherence]	Type IV pili	Adherence	Freshwater stream	China
NZ_CP008838.1-5	1028850-1061814	Incomplete	(flmH) short chain dehydrogenase/reductase family oxidoreductase[Polar flagella - Motility]<br>(pilZ) type 4 fimbrial biogenesis protein PilZ [Type IV pili - Adherence]	Polar flagella<br>Type IV pili	Motility<br>Adherence	Missing	America
NZ_CP008838.1-6	4460133-4467327	Incomplete	(htpB) Hsp60, 60K heat shock protein HtpB [Hsp60 - Adherence]<br>(groEL) chaperonin GroEL [GroEL - Adherence]	Hsp60<br>GroEL	Adherence<br>Adherence	Missing	America
NZ_CP014014.1-5	920328-947663	Incomplete	(adeG) cation/multidrug efflux pump [AdeFGH efflux pump - Biofilm]	AdeFGH efflux pump	Biofilm	Homo Respiratory Cx	America
NZ_CP028899.1-7	4277531-4290990	Incomplete	(sigA/rpoV) RNA polymerase sigma factor SigA [SigA - Regulation]	SigA	Regulation	Wastewater	Australia
NZ_CP033586.1-2	3558452-3581223	Incomplete	(pilZ) type 4 fimbrial biogenesis protein PilZ [Type IV pili -Adherence]	Type IV pili	Adherence	Soil	Mexico
NZ_CP037858.1-1	3114967-3137626	Incomplete	(pilZ) type 4 fimbrial biogenesis protein PilZ [Type IV pili -Adherence]	Type IV pili	Adherence	Aerobic granular sludge	China
NZ_CP037858.1-2	3870099-3886632	Incomplete	(fur) ferric iron uptake transcriptional regulator [Fur - Regulation]	Fur	Regulation	Aerobic granular sludge	China
NZ_CP040430.1-1	1097048-1119855	Incomplete	(pilZ) type 4 fimbrial biogenesis protein PilZ [Type IV pili -Adherence]	Type IV pili	Adherence	Missing	Germany
NZ_CP040431.1-2	1022762-1070816	Incomplete	(clpP) ATP-dependent Clp protease proteolytic subunit [ClpP - Stress survival]	ClpP	Stress survival	Missing	Germany
NZ_CP040433.1-1	934240-956225	Incomplete	(clpP) ATP-dependent Clp protease proteolytic subunit [ClpP - Stress survival]	ClpP	Stress survival	Missing	Germany
NZ_CP040435.1-5	1920811-1938097	Incomplete	(fur) ferric iron uptake transcriptional regulator [Fur - Regulation]	Fur	Regulation	Missing	Germany
NZ_CP040436.1-1	926796-975063	Incomplete	(clpP) ATP-dependent Clp protease proteolytic subunit [ClpP - Stress survival]	ClpP	Stress survival	Missing	Germany
NZ_CP040436.1-3	1827493-1847744	Incomplete	(fur) ferric iron uptake transcriptional regulator [Fur - Regulation]	Fur	Regulation	Missing	Germany
NZ_CP104169.1-2	4400889-4436235	Intact	(flmH) short chain dehydrogenase/reductase family oxidoreductase [Polar flagella - Motility]<br>(pilZ) type 4 fimbrial biogenesis protein PilZ [Type IV pili - Adherence]	Polar flagella<br>Type IV pili	Motility<br>Adherence	Hospital ICU ward	Malaysia

NZ_CP104324.1-2	3111887-3125093	Intact	(clpP) ATP-dependent Clp protease proteolytic subunit [ClpP - Stress survival]	ClpP	Stress survival	Freshwater stream	China
			(pilZ) type 4 fimbrial biogenesis protein PilZ [Type IV pili - Adherence]	Type IV pili	Adherence		
NZ_CP022053.2-2	1060294-1110481	Intact	(fur) ferric iron uptake transcriptional regulator [Fur - Regulation]	Fur	Regulation	Homo eye	America
NZ_CP022053.2-3	1953699-1996271	Intact	(pilZ) type 4 fimbrial biogenesis protein PilZ [Type IV pili -Adherence]	Type IV pili	Adherence	Homo eye	America
NZ_CP033829.1-1	722098-744561	Intact	(pilZ) type 4 fimbrial biogenesis protein PilZ [Type IV pili -Adherence]	Type IV pili	Adherence	Homo sputum	America
NZ_CP040440.1-3	1106475-1129063	Intact	(pilZ) type 4 fimbrial biogenesis protein PilZ [Type IV pili - Adherence]	Type IV pili	Adherence	Clinical	Germany
NZ_CP051467.1-3	2807210-2834673	Intact	(htpB) Hsp60, 60K heat shock protein HtpB [Hsp60 - Adherence]	Hsp60	Adherence	Missing	America
			(groEL) chaperonin GroEL [GroEL - Adherence]	GroEL	Adherence		
NZ_CP051467.1-4	3499919-3545961	Intact	(tviB) Vi polysaccharide biosynthesis UDP-N-acetylglucosamine C-6dehydrogenase TviB [Capsule - Immune modulation]	Capsule	Immune modulation	Missing	America
			(tviB) Vi polysaccharide biosynthesis protein, UDP-glucose/GDP-mannose dehydrogenase TviB [Vi antigen - Immune modulation]	Vi antigen	Immune modulation		
NZ_CP083454.1-1	4067671-4109106	Intact	(sigA/rpoV) RNA polymerase sigma factor SigA [SigA - Regulation]	SigA	Regulation	Cucumber rhizosphere	China
CP050452.1-2	1615410-1677436	Questionable	(tviB) Vi polysaccharide biosynthesis UDP-N-acetylglucosamine C-6dehydrogenase TviB [Capsule - Immune modulation]	Capsule	Immune modulation	Antarctic soil	Antarctica
NZ_CP104290.1-2	3134415-3156064	Questionable	(pilZ) type 4 fimbrial biogenesis protein PilZ [Type IV pili - Adherence]	Type IV pili	Adherence	Freshwater stream	China
NZ_CP014014.1-8	58811-72113	Questionable	(pilZ) type 4 fimbrial biogenesis protein PilZ [Type IV pili - Adherence]	Type IV pili	Adherence	Homo Respiratory Cx	America
NZ_CP040430.1-2	959894-1030045	Questionable	(clpP) ATP-dependent Clp protease proteolytic subunit [ClpP - Stress survival]	ClpP	Stress survival	Missing	Germany
NZ_CP060026.1-9	4145416-4157245	Questionable	(fur) ferric iron uptake transcriptional regulator [Fur - Regulation]	Fur	Regulation	ICU	Germany

CAZy

Prophage	Genome location	Prophage classification	CAZy	Singal peptide
NZ_CP080573.1-4	998040-1016631	Incomplete	GH104	N
NZ_CP098483.1-3	1100345-1122931	Incomplete	GH104	N

CP091781.1-2	1036506-1059075	Incomplete	GH104	N
NZ_CP104071.1-2	3304873-3324794	Incomplete	GH24	Y (1-30)
NZ_CP104286.1-2	1040549-1060553	Incomplete	GH104	N
NZ_CP104286.1-4	1840549-1857276	Incomplete	GH24	N
NZ_CP104287.1-1	2709954-2727014	Incomplete	GH24	N
NZ_CP104288.1-4	1010891-1045556	Incomplete	GH104	N
NZ_CP104290.1-1	1032485-1055063	Incomplete	GH104	N
NZ_CP104292.1-1	999628-1022233	Incomplete	GH104	N
NZ_CP104323.1-3	1867649-1900750	Incomplete	GH104	N
NZ_CP104324.1-4	3582840-3600751	Incomplete	GH24	N
NC_010943.1 -5	1966393-1983120	Incomplete	GH24	N
NZ_AP021908.1-2	1088047-1110855	Incomplete	GH104	N
NZ_CP008838.1-5	1028850-1061814	Incomplete	GH104	N
NZ_CP014014.1-3	75599-95347	Incomplete	GH104	N
NZ_CP014014.1-6	954402-971032	Incomplete	GH24	N
NZ_CP022053.2-5	1131962-1152240	Incomplete	GH24	N
NZ_CP028899.1-5	3652385-3687550	Incomplete	GH24	Y (1-27)
NZ_CP031058.1-2	384435-422365	Incomplete	GH104	N
NZ_CP031058.1-3	3200882-3223760	Incomplete	GH24	N
NZ_CP031058.1-5	3643531-3664688	Incomplete	GH23	Y (1-36)
NZ_CP031058.1-6	3915010-3925896	Incomplete	GH104	N
NZ_CP033586.1-2	3558452-3581223	Incomplete	GH104	N
NZ_CP033829.1-2	1474443-1488853	Incomplete	GH104	N
NZ_CP037858.1-1	3114967-3137626	Incomplete	GH104	N
NZ_CP040430.1-1	1097048-1119855	Incomplete	GH104	N
NZ_CP040431.1-3	1131574-1161473	Incomplete	GH104	N

NZ_CP040433.1-1	934240-956225	Incomplete	GH23	N
NZ_CP040435.1-5	1920811-1938097	Incomplete	GH104	N
NZ_CP040436.1-1	926796-975063	Incomplete	GH24	N
NZ_CP040436.1-3	1827493-1847744	Incomplete	GH19	N
NZ_CP040437.1-3	1019355-1038105	Incomplete	GH104	N
NZ_CP040438.1-1	1821314-1841902	Incomplete	GH23	N
NZ_CP040439.1-6	1956673-1979782	Incomplete	GH24	N
NZ_CP040440.1-6	1890191-1906918	Incomplete	GH24	N
NZ_CP044092.1-1	2227166-2274595	Incomplete	GH24	N
NZ_CP044092.1-2	2341070-2370970	Incomplete	GH104	N
NZ_CP049368.1-2	998741-1032555	Incomplete	GH104	N
			GT83	N
NZ_CP049956.1-4	343379-387519	Incomplete	GH24	N
NZ_CP049956.1-5	446929-469736	Incomplete	GH104	N
NZ_CP051467.1-6	4132573-4155346	Incomplete	GH104	N
NZ_CP052863.1-2	3641158-3663729	Incomplete	GH104	N
NZ_CP056088.1-4	3888149-3927146	Incomplete	GH24	N
NZ_CP060021.1-2	3629271-3651844	Incomplete	GH104	N
NZ_CP060022.1-8	1945006-1968059	Incomplete	GH24	N
NZ_CP060023.1-6	2615424-2638477	Incomplete	GH24	N
NZ_CP060024.1-4	708510-725237	Incomplete	GH24	N
NZ_CP060024.1-5	2547300-2569873	Incomplete	GH104	N
NZ_CP060026.1-5	2520427-2564582	Incomplete	GH24	N
NZ_CP060026.1-6	3244578-3267166	Incomplete	GH104	N
NZ_CP060027.1-2	2337167-2367066	Incomplete	GH104	N
NZ_CP065965.1-4	800366-822939	Incomplete	GH104	N

NZ_CP067993.1-3	1909204-1942434	Incomplete	GH104	N
NZ_CP067993.1-4	2841271-2852968	Incomplete	GH104	N
NZ_CP088241.1-3	1055986-1078793	Incomplete	GH104	N
NZ_CP088242.1-3	1791040-1809788	Incomplete	GH104	N
NZ_CP104863.1-2	3727930-3750651	Incomplete	GH104	N
NZ_LR134301.1-4	2516674-2535719	Incomplete	GH104	N
NZ_LR134301.1-5	2579065-2592666	Incomplete	GH23	N
NZ_LS483377.1-2	1014075-1034083	Incomplete	GH104	N
NZ_LT906480.1-7	2063165-2077252	Incomplete	GH104	N
NZ_CP104169.1-1	671102-690926	Intact	GH104	N
NZ_CP104169.1-2	4400889-4436235	Intact	GH19	N
NZ_CP080573.1-1	1584878-1627039	Intact	GH24	N
NZ_CP080573.1-3	3589858-3614171	Intact	GH104	N
NZ_CP040429.1-2	1637170-1678714	Intact	GH104	N
NZ_CP102942.1-1	1110850-1137659	Intact	GH104	N
NZ_CP011010.1-1	970762-1014681	Intact	GH24	N
NZ_CP011010.1-2	1070725-1103088	Intact	GH104	N
NZ_CP011010.1-3	1835742-1886513	Intact	GH19	N
NZ_CP011010.1-4	2908945-2937005	Intact	GH104	N
NZ_CP011305.1-1	968646-1000562	Intact	GH104	N
CP078102.1-1	246619-269988	Intact	GH104	N
NZ_CP090418.1-1	1048505-1086056	Intact	GH104	N
CP091781.1-1	2729380-2754123	Intact	GH24	N
NZ_CP101622.1-1	995358-1033712	Intact	GH19	N
NZ_CP101622.1-2	3437368-3457166	Intact	GH104	N
NZ_CP104288.1-2	3474972-3499933	Intact	GH104	N

NZ_CP104289.1-1	1729177-1755508	Intact	GH19	N
NZ_CP104324.1-1	2301305-2330715	Intact	GH104	N
NC_010943.1-2	299939-335333	Intact	GH19	N
NC_010943.1-1	1896567-1926321	Intact	GH24	N
NC_011071.1-1	1024520-1046742	Intact	GH104	N
NC_017671.1-2	3646341-3676885	Intact	GH19	N
NZ_CM001824.1-2	1003361-1026134	Intact	GH104	N
NZ_CP008838.1-1	435158-486726	Intact	GH24	N
			GH24	N
NZ_CP008838.1-2	940494-1020287	Intact	GT9	N
			GH23	Y (1-23)
NZ_CP008838.1-4	3606237-3642185	Intact	GH19	N
NZ_CP015612.1-1	1046357-1065439	Intact	GH104	N
NZ_CP015612.1-2	2845460-2871720	Intact	GH104	N
NZ_CP022053.2-3	1953699-1996271	Intact	CBM13+CBM13	N
			GH104	N
NZ_CP022053.2-4	4017408-4052113	Intact	GH24	N
NZ_CP027562.1-1	1031275-1050337	Intact	GH104	N
NZ_CP028358.1-1	318410-369235	Intact	GH24	N
NZ_CP028358.1-2	1094663-1114599	Intact	GH104	N
NZ_CP028358.1-4	4162008-4210377	Intact	GH104	N
NZ_CP028899.1-3	4237609-4288145	Intact	GH104	N
NZ_CP029773.1-1	999327-1018357	Intact	GH104	N
NZ_CP031058.1-1	2010692-2059957	Intact	GH24	N
NZ_CP033829.1-1	722098-744561	Intact	GH104	N
NZ_CP040431.1-1	276802-314369	Intact	GH19	N

NZ_CP040432.1-1	1834361-1875001	Intact	GH24	N
NZ_CP040435.1-1	1053958-1073211	Intact	GH104	N
NZ_CP040435.1-4	4277687-4300979	Intact	GH104	N
NZ_CP040439.1-1	1765759-1785698	Intact	GH104	N
NZ_CP040439.1-3	4367626-4395396	Intact	GH104	N
NZ_CP040440.1-1	246639-299394	Intact	GH19	N
NZ_CP040440.1-2	968282-1012437	Intact	GH24	N
NZ_CP040440.1-3	1106475-1129063	Intact	GH104	N
			GH24	N
			AA1	N
NZ_CP040440.1-5	3722196-3831252	Intact	CE1	Y (1-48)
			CE1	Y (1-31)
			GH24	N
NZ_CP049956.1-2	2360140-2387662	Intact	GH19	N
NZ_CP049956.1-3	3697168-3747691	Intact	GH104	N
NZ_CP051467.1-1	1858492-1908635	Intact	GH19	N
NZ_CP056088.1-1	2477880-2496967	Intact	GH104	N
NZ_CP060022.1-5	491812-514899	Intact	GH24	N
NZ_CP060024.1-1	3722730-3734174	Intact	GH19	N
NZ_CP060026.1-3	1744019-1845858	Intact	GH24	N
NZ_CP060027.1-1	634113-671680	Intact	GH19	N
NZ_CP060259.1-1	953089-985543	Intact	GH104	N
NZ_CP060259.1-2	1052774-1072569	Intact	GH104	N
NZ_CP065965.1-1	3135051-3175307	Intact	GH24	N
NZ_CP065965.1-2	4967619-4999409	Intact	GH19	N
NZ_CP067993.1-2	3241035-3288062	Intact	CBM32	N

NZ_CP077679.1-1	996338-1016152	Intact	GH104	N
NZ_CP083454.1-1	4067671-4109106	Intact	GH19	N
NZ_CP088240.1-1	767650-787448	Intact	GH104	N
NZ_CP088242.1-1	998147-1031698	Intact	GH19	N
NZ_CP088243.1-1	2143657-2177208	Intact	GH19	N
NZ_CP088244.1-2	4063857-4099586	Intact	GH104	N
NZ_CP090423.1-2	1733223-1764501	Intact	GH19	N
NZ_CP090423.1-3	4286080-4321666	Intact	GH24	N
			CE16	N
NZ_CP104863.1-1	1919878-1963452	Intact	GH19	N
NZ_CP106759.1-2	2460397-2487770	Intact	GH19	N
NZ_LS483406.1-1	1036379-1059106	Intact	GH104	N
NZ_LT906480.1-1	246209-297209	Intact	GH19	N
NZ_LT906480.1-2	994240-1074033	Intact	GH24	N
			GT9	N
			GH23	Y (1-23)
NZ_LT906480.1-3	1093964-1116537	Intact	GH104	N
NZ_LT906480.1-5	3447458-3468903	Intact	GH24	N
NZ_OU943334.1-1	1068442-1088261	Intact	GH104	N
NZ_CP102942.1-5	3734750-3754016	Questionable	GH104	N
CP050452.1-2	1615410-1677436	Questionable	GH104	N
NZ_CP090418.1-5	3930430-3970303	Questionable	GH24	N
NZ_CP101622.1-4	2693403-2710150	Questionable	GH104	N
NZ_CP104290.1-2	3134415-3156064	Questionable	GH104	N
NC_010943.1-6	1091933-1113529	Questionable	GH104	N
NC_011071.1-2	2795124-2816171	Questionable	GH104	N

NZ_CP008838.1-7	2008142-2025542	Questionable	GH104	N
NZ_CP022053.2-8	4232129-4281236	Questionable	GH24	N
NZ_CP040430.1-2	959894-1030045	Questionable	GH23	Y (1-23)
NZ_CP051467.1-7	3116263-3138619	Questionable	GH104	N
NZ_CP060026.1-9	3301363-3318090	Questionable	GH24	N
NZ_CP065965.1-10	717134-766147	Questionable	GH24	N
			GT9	N
NZ_CP065965.1-11	1769569-1783656	Questionable	GH104	N
NZ_CP088240.1-3	1529378-1546008	Questionable	GH24	N
NZ_LS483377.1-3	1911582-1950744	Questionable	GH104	N
NZ_OU943334.1-4	664913-718129	Questionable	GH24	N

Table S6: Statistics of HGT events and prophage mediated HGT in *S. maltophilia* strains, N stands for no.

Strain	HGTs result	Function	Intact prophage hit HGTs	Identities(%)	Incomplete prophage hit HGTs	Identities(%)	Questionable prophage hit HGTs	Identities(%)
CP050452	QNA94695.1	ABC transporter ATP-binding protein	N	N	N	N	N	N
	QNA94786.1	hypothetical protein G4G30_03160	N	N	N	N	N	N
	QNA95033.1	AAA family ATPase	N	N	N	N	N	N
	QNA95448.1	phage major capsid protein	N	N	N	N	NZ_CP080573.1-4	100
	QNA95640.1	DUF4391 domain-containing protein	N	N	N	N	N	N
	QNA95864.1	hypothetical protein G4G30_10350	N	N	N	N	N	N
	QNA97245.1	DUF3304 domain-containing protein	N	N	N	N	N	N
	QNA97378.1	terminase large subunit	N	N	N	N	N	N
	QNA97465.1	chromate efflux transporter	N	N	N	N	N	N
CP071784.1	WP_005409726.1	ABC transporter permease	N	N	N	N	N	N
	WP_005409874.1	biopolymer transporter ExbD	N	N	N	N	N	N
	WP_005409898.1	AzlD family protein	N	N	N	N	N	N
	WP_005409984.1	MgtC/SapB family protein	N	N	N	N	N	N
	WP_005411610.1	phosphopantetheine-binding protein	N	N	N	N	N	N
	WP_005413654.1	Lrp/AsnC family transcriptional regulator	N	N	N	N	N	N
	WP_005416613.	ABC transporter ATP-binding protein	N	N	N	N	N	N

1							
WP_005419760.1	hypothetical protein	N	N	N	N	N	N
WP_005420284.1	hypothetical protein	N	N	N	N	N	N
WP_006402206.1	acyl carrier protein	N	N	N	N	N	N
WP_010482545.1	response regulator	N	N	N	N	N	N
WP_010484073.1	type II toxin-antitoxin system RelE/ParE family toxin	N	N	N	N	N	N
WP_010484074.1	HigA family addiction module antitoxin	N	N	N	N	N	N
WP_010484092.1	winged helix-turn-helix domain-containing protein	N	N	N	N	N	N
WP_010485313.1	respiratory nitrate reductase subunit gamma	N	N	N	N	N	N
WP_010485596.1	glucose 1-dehydrogenase	N	N	N	N	N	N
WP_010485668.1	LysR family transcriptional regulator	N	N	N	N	N	N
WP_010486565.1	TetR/AcrR family transcriptional regulator	N	N	N	N	N	N
WP_019658847.1	alpha/beta hydrolase	N	N	N	N	N	N
WP_019659002.	response regulator transcription factor	N	N	N	N	N	N

1							
WP_019659022.1	carboxymuconolactone decarboxylase family protein	N	N	N	N	N	N
WP_019659087.1	response regulator transcription factor	N	N	N	N	N	N
WP_019659189.1	response regulator	N	N	N	N	N	N
WP_019659192.1	EamA family transporter	N	N	N	N	N	N
WP_019659193.1	DMT family transporter	N	N	N	N	N	N
WP_019659196.1	aminotransferase class III-fold pyridoxal phosphate-dependent enzyme	N	N	N	N	N	N
WP_019659257.1	cellulase family glycosylhydrolase	N	N	N	N	N	N
WP_019659276.1	GyrI-like domain-containing protein	N	N	N	N	N	N
WP_019659347.1	alpha/beta hydrolase	N	N	N	N	N	N
WP_019659355.1	helix-turn-helix transcriptional regulator	N	N	N	N	N	N
WP_019659465.1	DUF4287 domain-containing protein	N	N	N	N	N	N
WP_019659488.1	tautomerase family protein	N	N	N	N	N	N
WP_019659639.	type II toxin-antitoxin system RelE/ParE	N	N	N	N	N	N

1	family toxin						
WP_019659640.1	HigA family addiction module antitoxin	N	N	N	N	N	N
WP_019659676.1	response regulator	N	N	N	N	N	N
WP_019659784.1	NAD(P)-dependent oxidoreductase	N	N	N	N	N	N
WP_019659837.1	winged helix-turn-helix domain-containing protein	N	N	N	N	N	N
WP_019659873.1	AzlC family ABC transporter permease	N	N	N	N	N	N
WP_019659912.1	diacylglycerol kinase	N	N	N	N	N	N
WP_019659932.1	tetratricopeptide repeat protein	N	N	N	N	N	N
WP_019659943.1	VOC family protein	N	N	N	N	N	N
WP_019659950.1	MerR family transcriptional regulator	N	N	N	N	N	N
WP_019660234.1	glutamine amidotransferase	N	N	N	N	N	N
WP_019660241.1	RidA family protein	N	N	N	N	N	N
WP_019660307.1	winged helix-turn-helix transcriptional regulator	N	N	N	N	N	N
WP_019660535.	alpha/beta hydrolase	N	N	N	N	N	N

1							
WP_019660551.1	SMR family transporter	N	N	N	N	N	N
WP_019660556.1	metalloregulator ArsR/SmtB family transcription factor	N	N	N	N	N	N
WP_019660561.1	protein-tyrosine-phosphatase	N	N	N	N	N	N
WP_019660562.1	carboxymuconolactone decarboxylase family protein	N	N	N	N	N	N
WP_019660699.1	Gfo/Idh/MocA family oxidoreductase	N	N	N	N	N	N
WP_019660756.1	VOC family protein	N	N	N	N	N	N
WP_019660932.1	class I SAM-dependent methyltransferase	N	N	N	N	N	N
WP_019661026.1	UDP-forming cellulose synthase catalytic subunit	N	N	N	N	N	N
WP_019661429.1	Cu(I)-responsive transcriptional regulator	N	N	N	N	N	N
WP_019661838.1	putative addiction module antidote protein	N	N	N	N	N	N
WP_019662024.1	TonB system transport protein ExbD	N	N	N	N	N	N
WP_021202814.1	DUF2239 family protein	N	N	N	N	N	N
WP_026347179.	low molecular weight	N	N	N	N	N	N

1	protein-tyrosine-phosphatase						
WP_029380003.1	biotin/lipoyl-binding protein	N	N	N	N	N	N
WP_049395317.1	transcriptional regulator BetI	N	N	N	N	N	N
WP_049411723.1	hypothetical protein	N	N	N	N	N	N
WP_049483081.1	protein deglycase HchA	N	N	N	N	N	N
WP_053443160.1	nitrate/nitrite transporter	N	N	N	N	N	N
WP_100445881.1	TIGR03571 family LLM class oxidoreductase	N	N	N	N	N	N
WP_111103887.1	helix-turn-helix domain-containing protein	N	N	N	N	N	N
WP_125438075.1	ABC transporter ATP-binding protein	N	N	N	N	N	N
WP_191850731.1	DMT family transporter	N	N	N	N	N	N
WP_194430608.1	DoxX family protein	N	N	N	N	N	N
WP_233614865.1	helix-turn-helix domain-containing protein	N	N	N	N	N	N
WP_233652748.1	NarK family nitrate/nitrite MFS transporter	N	N	N	N	N	N
WP_233652758.	type II secretion system major	N	N	N	N	N	N

1	pseudopilin GspG						
WP_233652775.1	cyclase family protein	N	N	N	N	N	N
WP_233652789.1	TonB-dependent siderophore receptor	N	N	N	N	N	N
WP_233652794.1	sensory rhodopsin transducer	N	N	N	N	N	N
WP_233652795.1	TIGR03885 family FMN-dependent LLM class oxidoreductase	N	N	N	N	N	N
WP_233652833.1	TetR family transcriptional regulator	N	N	N	N	N	N
WP_233652834.1	nuclear transport factor 2 family protein	N	N	N	N	N	N
WP_233652835.1	aldehyde oxidoreductase molybdenum-binding subunit PaoC	N	N	N	N	N	N
WP_233652844.1	alkyl sulfatase dimerization domain-containing protein	N	N	N	N	N	N
WP_233652886.1	BCCT family transporter	N	N	N	N	N	N
WP_233652930.1	SDR family oxidoreductase	N	N	N	N	N	N
WP_233652942.1	SRPBCC domain-containing protein	N	N	N	N	N	N
WP_233653449.1	VOC family protein	N	N	N	N	N	N
WP_233653538.	DNA polymerase III subunit epsilon	N	N	N	N	N	N

1							
WP_233653621.1	ubiquinol oxidase subunit II	N	N	N	N	N	N
WP_233653622.1	cytochrome o ubiquinol oxidase subunit III	N	N	N	N	N	N
WP_233653707.1	CmlA/FloR family chloramphenicol efflux MFS transporter	N	N	N	N	N	N
WP_233653781.1	selenide, water dikinase SelD	N	N	N	N	N	N
WP_233653782.1	selenocysteine-specific translation elongation factor	N	N	N	N	N	N
WP_233653783.1	L-seryl-tRNA(Sec) selenium transferase	N	N	N	N	N	N
WP_233653785.1	formate dehydrogenase subunit gamma	N	N	N	N	N	N
WP_233653828.1	response regulator transcription factor	N	N	N	N	N	N
WP_233653836.1	aromatic alcohol reductase	N	N	N	N	N	N
WP_233653907.1	Rid family hydrolase	N	N	N	N	N	N
WP_233653913.1	methylated-DNA--[protein]-cysteine S-methyltransferase	N	N	N	N	N	N
WP_233653914.1	SDR family oxidoreductase	N	N	N	N	N	N
WP_233653916.	sugar phosphate isomerase/epimerase	N	N	N	N	N	N

	1	family protein						
	WP_233654110.1	4-oxalomesaconate tautomerase	N	N	N	N	N	N
	WP_272773617.1	helix-turn-helix transcriptional regulator	N	N	N	N	N	N
	WP_005408332.1	GPW/gp25 family protein	N	N	N	N	N	N
	WP_005408904.1	nuclear transport factor 2 family protein	N	N	N	N	N	N
	WP_021203803.1	phage major capsid protein	CP091781.1-1	100	N	N	N	N
	WP_024957746.1	transcriptional regulator	N	N	N	N	N	N
	WP_049449698.1	GNAT family N-acetyltransferase	N	N	N	N	N	N
CP091781.1	WP_049449966.1	ABC transporter ATP-binding protein	N	N	N	N	N	N
	WP_049449972.1	DegT/DnrJ/EryC1/StrS family aminotransferase	N	N	N	N	N	N
	WP_049449986.1	WxcM-like domain-containing protein	N	N	N	N	N	N
	WP_049449988.1	glycosyltransferase family 2 protein	N	N	N	N	N	N
	WP_049449989.1	DegT/DnrJ/EryC1/StrS family aminotransferase	N	N	N	N	N	N
	WP_060381232.	head-tail connector protein	N	N	N	N	N	N

1							
WP_088436955.1	AraC family transcriptional regulator	N	N	N	N	N	N
WP_108269783.1	amidohydrolase	N	N	N	N	N	N
WP_134945719.1	HK97 family phage prohead protease	CP091781.1-1	100	N	N	N	N
WP_149500949.1	abortive infection family protein	N	N	N	N	N	N
WP_149500981.1	DJ-1/PfpI family protein	N	N	N	N	N	N
WP_149501043.1	SDR family oxidoreductase	N	N	N	N	N	N
WP_182680989.1	phage portal protein	CP091781.1-1		N	N	N	N
WP_202151436.1	amidase family protein	N	N	N	N	N	N
WP_227844547.1	cytochrome c	N	N	N	N	N	N
WP_227844548.1	GMC family oxidoreductase	N	N	N	N	N	N
WP_227844549.1	gluconate 2-dehydrogenase subunit 3 family protein	N	N	N	N	N	N
WP_227844553.1	iron chelate uptake ABC transporter family permease subunit	N	N	N	N	N	N
WP_227844557.	fumarylacetoacetate hydrolase family	N	N	N	N	N	N

	1	protein						
	WP_227844562.1	carboxymuconolactone decarboxylase family protein	N	N	N	N	N	N
	WP_227844565.1	cupin domain-containing protein	N	N	N	N	N	N
	WP_227860033.1	ATP-binding cassette domain-containing protein	N	N	N	N	N	N
	WP_237826746.1	hypothetical protein	N	N	N	N	N	N
	WP_237826908.1	CBASS oligonucleotide cyclase	N	N	N	N	N	N
	WP_237826911.1	hypothetical protein	N	N	N	N	N	N
	WP_237826914.1	AAA family ATPase	N	N	N	N	N	N
	WP_237826917.1	SAVED domain-containing protein	N	N	N	N	N	N
	WP_237827068.1	glycosyltransferase family 4 protein	N	N	N	N	N	N
	WP_237827252.1	aldehyde dehydrogenase family protein	N	N	N	N	N	N
	WP_005408112.1	LysR family transcriptional regulator	N	N	N	N	N	N
NZ_010943.1	WP_005408332.1	GPW/gp25 family protein	N	N	N	N	N	N
	WP_005411786.	efflux RND transporter periplasmic	N	N	N	N	N	N

1	adaptor subunit						
WP_005414259.1	hypothetical protein	N	N	N	N	N	N
WP_005414983.1	ATP-binding protein	N	N	N	N	N	N
WP_005414984.1	MFS transporter	N	N	N	N	N	N
WP_005995733.1	DUF305 domain-containing protein	N	N	N	N	N	N
WP_012478642.1	UvrD-helicase domain-containing protein	N	N	N	N	N	N
WP_012478672.1	helix-turn-helix transcriptional regulator	N	N	N	N	N	N
WP_012478840.1	phage tail assembly protein	N	N	N	N	N	N
WP_012478843.1	GPW/gp25 family protein	N	N	N	N	N	N
WP_012478857.1	phage terminase small subunit	NC_010943.1-2	100	N	N	N	N
WP_012478973.1	McrC family protein	N	N	N	N	N	N
WP_012479055.1	ABC transporter permease	N	N	N	N	N	N
WP_012479056.1	ABC transporter ATP-binding protein	N	N	N	N	N	N
WP_012479074.	SMR family transporter	N	N	N	N	N	N

1							
WP_012479075.1	TIM barrel protein	N	N	N	N	N	N
WP_012479077.1	FAD-dependent oxidoreductase	N	N	N	N	N	N
WP_012479078.1	glycosyltransferase	N	N	N	N	N	N
WP_012479459.1	P-type conjugative transfer protein TrbL	N	N	N	N	N	N
WP_012479460.1	P-type conjugative transfer protein TrbJ	N	N	N	N	N	N
WP_012479463.1	TrbC/VirB2 family protein	N	N	N	N	N	N
WP_012479468.1	LysR substrate-binding domain-containing protein	N	N	N	N	N	N
WP_012479470.1	NAD(P)H-dependent oxidoreductase	N	N	N	N	N	N
WP_012479471.1	LysR family transcriptional regulator	N	N	N	N	N	N
WP_012479472.1	EexN family lipoprotein	N	N	N	N	N	N
WP_012479473.1	LysR substrate-binding domain-containing protein	N	N	N	N	N	N
WP_012479476.1	MerR family transcriptional regulator	N	N	N	N	N	N
WP_012479477.	MFS transporter	N	N	N	N	N	N

1							
WP_012479481.1	S26 family signal peptidase	N	N	N	N	N	N
WP_012479482.1	DUF2840 domain-containing protein	N	N	N	N	N	N
WP_012479483.1	chromosome partitioning protein ParB	N	N	N	N	N	N
WP_012479486.1	helix-turn-helix domain-containing protein	N	N	N	N	N	N
WP_012479487.1	DUF2285 domain-containing protein	N	N	N	N	N	N
WP_012479488.1	DUF2958 domain-containing protein	N	N	N	N	N	N
WP_012479489.1	helix-turn-helix transcriptional regulator	N	N	N	N	N	N
WP_012479493.1	IS630-like element ISStma10 family transposase	N	N	N	N	N	N
WP_012479494.1	hypothetical protein	N	N	N	N	N	N
WP_012479495.1	hypothetical protein	N	N	N	N	N	N
WP_012479501.1	hypothetical protein	N	N	N	N	N	N
WP_012479502.1	DNA-binding transcriptional regulator	N	N	N	N	N	N
WP_012479503.	type II toxin-antitoxin system RelE/ParE	N	N	N	N	N	N

1	family toxin						
WP_012479702.<br>1	hypothetical protein, partial	N	N	N	N	N	N
WP_012479808.<br>1	lysozyme	N	N	N	N	N	N
WP_012479809.<br>1	DUF6127 family protein	N	N	N	N	N	N
WP_012479810.<br>1	DUF2793 domain-containing protein	N	N	N	N	N	N
WP_012479811.<br>1	phage tail protein	NC_010943.1-1	100	N	N	N	N
WP_012479815.<br>1	hypothetical protein	NC_010943.1-1	100	N	N	N	N
WP_012479816.<br>1	tail protein	NC_010943.1-1	100	N	N	N	N
WP_012479817.<br>1	DUF6441 family protein	NC_010943.1-1	100	N	N	N	N
WP_012479819.<br>1	hypothetical protein	NC_010943.1-1	100	N	N	N	N
WP_012479822.<br>1	major capsid protein	NC_010943.1-1	100	N	N	N	N
WP_012479823.<br>1	head decoration protein	N	N	N	N	N	N
WP_012479824.<br>1	S49 family peptidase	NC_010943.1-1	100	N	N	N	N
WP_012479826.	hypothetical protein	N	N	N	N	N	N

1							
WP_012479827.1	hypothetical protein	N	N	N	N	N	N
WP_012479828.1	hypothetical protein	N	N	N	N	N	N
WP_012479830.1	hypothetical protein	NC_010943.1-1	100	N	N	N	N
WP_012479833.1	hypothetical protein	N	N	N	N	N	N
WP_012479837.1	site-specific DNA-methyltransferase	N	N	N	N	N	N
WP_012479840.1	hypothetical protein	N	N	N	N	N	N
WP_012479841.1	hypothetical protein	N	N	N	N	N	N
WP_012479842.1	phage/plasmid primase, P4 family	N	N	N	N	N	N
WP_012479844.1	isoleucyl-tRNA synthetase	N	N	N	N	N	N
WP_012479846.1	hypothetical protein	N	N	N	N	N	N
WP_012479848.1	BRO family protein	N	N	N	N	N	N
WP_012479854.1	helix-turn-helix transcriptional regulator	N	N	N	N	N	N
WP_012479855.	DUF2924 domain-containing protein	N	N	N	N	N	N

1							
WP_012479856.1	recombinase family protein	N	N	N	N	N	N
WP_012479857.1	hypothetical protein	N	N	N	N	N	N
WP_012479858.1	hypothetical protein	N	N	N	N	N	N
WP_012479994.1	LysR substrate-binding domain-containing protein	N	N	N	N	N	N
WP_012480184.1	isochorismatase family cysteine hydrolase	N	N	N	N	N	N
WP_012480188.1	Hg(II)-responsive transcriptional regulator	N	N	N	N	N	N
WP_012480190.1	mercury resistance system periplasmic binding protein MerP	N	N	N	N	N	N
WP_012480196.1	NAD(P)/FAD-dependent oxidoreductase	N	N	N	N	N	N
WP_012480197.1	metalloregulator ArsR/SmtB family transcription factor	N	N	N	N	N	N
WP_012480249.1	TM0106 family RecB-like putative nuclease	N	N	N	N	N	N
WP_012480368.1	TIGR03885 family FMN-dependent LLM class oxidoreductase	N	N	N	N	N	N
WP_012481497.1	cytochrome o ubiquinol oxidase subunit IV	N	N	N	N	N	N
WP_024957346.	glycosyltransferase family 2 protein	N	N	N	N	N	N

1							
WP_024957931.1	DUF1629 domain-containing protein	N	N	N	N	N	N
WP_044569661.1	LysR family transcriptional regulator	N	N	N	N	N	N
WP_044569669.1	site-specific integrase	N	N	N	N	N	N
WP_044569892.1	hypothetical protein	N	N	N	N	N	N
WP_044569895.1	hypothetical protein	N	N	N	N	N	N
WP_044569900.1	phage terminase large subunit family protein	NC_010943.1-1	100	N	N	N	N
WP_044569912.1	DUF6362 family protein	N	N	N	N	N	N
WP_044569915.1	ATP-binding protein	N	N	N	N	N	N
WP_044569917.1	hypothetical protein	N	N	N	N	N	N
WP_044571296.1	DUF6511 domain-containing protein	N	N	N	N	N	N
WP_044571374.1	helix-turn-helix domain-containing protein	N	N	N	N	N	N
WP_080047724.1	glycoside hydrolase family protein	N	N	N	N	N	N
WP_087944431.	IS3 family transposase	NC_010943.1-1	100	N	N	N	N

	1							
	WP_197599535.1	NAD(P)-dependent oxidoreductase	N	N	N	N	N	N
	WP_198408047.1	phage portal protein	NC_010943.1-1	100	N	N	N	N
	WP_198408048.1	DUF3489 domain-containing protein	N	N	N	N	N	N
	WP_198408049.1	site-specific DNA-methyltransferase	N	N	N	N	N	N
	WP_229297698.1	SMI1/KNR4 family protein	N	N	N	N	N	N
	WP_231910850.1	type II toxin-antitoxin system RelE/ParE family toxin	N	N	N	N	N	N
	WP_231914718.1	HNH endonuclease signature motif containing protein	N	N	N	N	N	N
	WP_232503886.1	hypothetical protein	N	N	N	N	N	N
	WP_232503891.1	EexN family lipoprotein	N	N	N	N	N	N
	WP_004135208.1	LysR substrate-binding domain-containing protein	N	N	N	N	N	N
NC_011071.1	WP_004135210.1	carboxymuconolactone decarboxylase family protein	N	N	N	N	N	N
	WP_004141029.1	winged helix-turn-helix domain-containing protein	N	N	N	N	N	N
	WP_004150098.	response regulator	N	N	N	N	N	N

1							
WP_005408332.1	GPW/gp25 family protein	N	N	N	N	N	N
WP_005409896.1	Lrp/AsnC family transcriptional regulator	N	N	N	N	N	N
WP_005416651.1	RebB family R body protein	N	N	N	N	N	N
WP_006362647.1	LysR substrate-binding domain-containing protein	N	N	N	N	N	N
WP_006363367.1	Cu(I)-responsive transcriptional regulator	N	N	N	N	N	N
WP_006367009.1	NAD(P)H-dependent oxidoreductase	N	N	N	N	N	N
WP_006367022.1	pirin family protein	N	N	N	N	N	N
WP_006368465.1	hypothetical protein	N	N	N	N	N	N
WP_006368769.1	RebB family R body protein	N	N	N	N	N	N
WP_006370165.1	heavy metal response regulator transcription factor	N	N	N	N	N	N
WP_006370834.1	type II secretion system major pseudopilin GspG	N	N	N	N	N	N
WP_006372781.1	VOC family protein	N	N	N	N	N	N
WP_006375128.	helix-turn-helix domain-containing	N	N	N	N	N	N

1	protein						
WP_006376243.1	LysR substrate-binding domain-containing protein	N	N	N	N	N	N
WP_006395500.1	VOC family protein	N	N	N	N	N	N
WP_006396593.1	glutathione S-transferase family protein	N	N	N	N	N	N
WP_006397453.1	RhuM family protein	N	N	N	N	N	N
WP_006399830.1	cytochrome o ubiquinol oxidase subunit IV	N	N	N	N	N	N
WP_006401882.1	hypothetical protein	N	N	N	N	N	N
WP_006402206.1	acyl carrier protein	N	N	N	N	N	N
WP_010485313.1	respiratory nitrate reductase subunit gamma	N	N	N	N	N	N
WP_012509672.1	type II toxin-antitoxin system HipA family toxin	N	N	N	N	N	N
WP_012509683.1	ShlB/FhaC/HecB family hemolysin secretion/activation protein	N	N	N	N	N	N
WP_012509718.1	tautomerase family protein	N	N	N	N	N	N
WP_012509779.1	type II toxin-antitoxin system RelE/ParE family toxin	N	N	N	N	N	N
WP_012509780.	putative addiction module antidote	N	N	N	N	N	N

1	protein						
WP_012509924.1	MFS transporter	N	N	N	N	N	N
WP_012509968.1	ABC transporter ATP-binding protein	N	N	N	N	N	N
WP_012509972.1	UDP-glucose 4-epimerase GalE	N	N	N	N	N	N
WP_012510078.1	LysR family transcriptional regulator	N	N	N	N	N	N
WP_012510079.1	MFS transporter	N	N	N	N	N	N
WP_012510145.1	VOC family protein	N	N	N	N	N	N
WP_012510190.1	flavin reductase family protein	N	N	N	N	N	N
WP_012510230.1	phage tail sheath subtilisin-like domain-containing protein	NC_011071.1-1	100	N	N	N	N
WP_012510261.1	response regulator transcription factor	N	N	N	N	N	N
WP_012510274.1	GNAT family N-acetyltransferase	N	N	N	N	N	N
WP_012510354.1	class I SAM-dependent DNA methyltransferase	N	N	N	N	N	N
WP_012510357.1	SDR family oxidoreductase	N	N	N	N	N	N
WP_012510358.	AraC family transcriptional regulator	N	N	N	N	N	N

1							
WP_012510368.1	helix-turn-helix domain-containing GNAT family N-acetyltransferase	N	N	N	N	N	N
WP_012510370.1	isocitrate lyase/PEP mutase family protein	N	N	N	N	N	N
WP_012510374.1	GNAT family N-acetyltransferase	N	N	N	N	N	N
WP_012510375.1	LysR family transcriptional regulator	N	N	N	N	N	N
WP_012510376.1	alpha/beta hydrolase	N	N	N	N	N	N
WP_012510380.1	helix-turn-helix transcriptional regulator	N	N	N	N	N	N
WP_012510425.1	GyrI-like domain-containing protein	N	N	N	N	N	N
WP_012510443.1	cellulase family glycosylhydrolase	N	N	N	N	N	N
WP_012510460.1	aminotransferase class III-fold pyridoxal phosphate-dependent enzyme	N	N	N	N	N	N
WP_012510464.1	EamA family transporter	N	N	N	N	N	N
WP_012510599.1	carboxymuconolactone decarboxylase family protein	N	N	N	N	N	N
WP_012510627.1	thioesterase family protein	N	N	N	N	N	N
WP_012510630.	ATP-binding protein	N	N	N	N	N	N

1							
WP_012510673.1	TonB-dependent receptor	N	N	N	N	N	N
WP_012510687.1	alpha/beta hydrolase	N	N	N	N	N	N
WP_012510747.1	GNAT family N-acetyltransferase	N	N	N	N	N	N
WP_012510802.1	LysE family transporter	N	N	N	N	N	N
WP_012510803.1	AraC family transcriptional regulator	N	N	N	N	N	N
WP_012510812.1	DUF2130 domain-containing protein	N	N	N	N	N	N
WP_012510847.1	metalloregulator ArsR/SmtB family transcription factor	N	N	N	N	N	N
WP_012510848.1	DUF2938 domain-containing protein	N	N	N	N	N	N
WP_012510861.1	dihydrodipicolinate synthase family protein	N	N	N	N	N	N
WP_012510863.1	GNAT family N-acetyltransferase	N	N	N	N	N	N
WP_012510944.1	transcriptional regulator BetI	N	N	N	N	N	N
WP_012510945.1	BCCT family transporter	N	N	N	N	N	N
WP_012510950.	alpha/beta fold hydrolase	N	N	N	N	N	N

1							
WP_012511031.1	LysR family transcriptional regulator	N	N	N	N	N	N
WP_012511039.1	chromate efflux transporter	N	N	N	N	N	N
WP_012511045.1	ArsO family NAD(P)H-dependent flavin-containing monooxygenase	N	N	N	N	N	N
WP_012511046.1	arsenate reductase ArsC	N	N	N	N	N	N
WP_012511056.1	Ohr family peroxiredoxin	N	N	N	N	N	N
WP_012511057.1	SDR family oxidoreductase	N	N	N	N	N	N
WP_012511058.1	LysR family transcriptional regulator	N	N	N	N	N	N
WP_012511060.1	carboxymuconolactone decarboxylase family protein	N	N	N	N	N	N
WP_012511061.1	cupin domain-containing protein	N	N	N	N	N	N
WP_012511062.1	RNA polymerase sigma-70 factor	N	N	N	N	N	N
WP_012511063.1	MBL fold metallo-hydrolase	N	N	N	N	N	N
WP_012511067.1	TetR family transcriptional regulator	N	N	N	N	N	N
WP_012511070.	NADP-dependent oxidoreductase	N	N	N	N	N	N

1							
WP_012511072.1	LysR family transcriptional regulator	N	N	N	N	N	N
WP_012511073.1	MFS transporter	N	N	N	N	N	N
WP_012511074.1	aldo/keto reductase	N	N	N	N	N	N
WP_012511111.1	ATP-binding cassette domain-containing protein	N	N	N	N	N	N
WP_012511168.1	ATP-binding protein	N	N	N	N	N	N
WP_012511174.1	PepSY-associated TM helix domain-containing protein	N	N	N	N	N	N
WP_012511219.1	response regulator transcription factor	N	N	N	N	N	N
WP_012511234.1	LysR family transcriptional regulator	N	N	N	N	N	N
WP_012511243.1	biopolymer transporter ExbD	N	N	N	N	N	N
WP_012511262.1	AzlD family protein	N	N	N	N	N	N
WP_012511275.1	NarK family nitrate/nitrite MFS transporter	N	N	N	N	N	N
WP_012511332.1	MgtC/SapB family protein	N	N	N	N	N	N
WP_012511428.	helix-turn-helix domain-containing	N	N	N	N	N	N

1	protein						
WP_012511446.1	MerR family transcriptional regulator	N	N	N	N	N	N
WP_012511447.1	NADH:flavin oxidoreductase/NADH oxidase family protein	N	N	N	N	N	N
WP_012511450.1	FAD-dependent monooxygenase	N	N	N	N	N	N
WP_012511453.1	response regulator transcription factor	N	N	N	N	N	N
WP_012511462.1	site-specific integrase	N	N	N	N	N	N
WP_012511482.1	phage major capsid protein	N	N	N	N	NC_011071.1-2	100
WP_012511483.1	Clp protease ClpP	N	N	N	N	NC_011071.1-2	100
WP_012511493.1	glycoside hydrolase family 104 protein	N	N	N	N	N	N
WP_012511532.1	agmatine deiminase family protein	N	N	N	N	N	N
WP_012511543.1	LysR family transcriptional regulator	N	N	N	N	N	N
WP_012511544.1	zinc-dependent alcohol dehydrogenase family protein	N	N	N	N	N	N
WP_012511668.1	TetR/AcrR family transcriptional regulator	N	N	N	N	N	N
WP_012511670.	alpha/beta hydrolase	N	N	N	N	N	N

1							
WP_012511674.1	SDR family oxidoreductase	N	N	N	N	N	N
WP_012511687.1	SMR family transporter	N	N	N	N	N	N
WP_012511690.1	metalloregulator ArsR/SmtB family transcription factor	N	N	N	N	N	N
WP_012511912.1	response regulator transcription factor	N	N	N	N	N	N
WP_012511986.1	alpha/beta hydrolase	N	N	N	N	N	N
WP_012511987.1	helix-turn-helix transcriptional regulator	N	N	N	N	N	N
WP_012511993.1	formate dehydrogenase subunit beta	N	N	N	N	N	N
WP_012511994.1	formate dehydrogenase subunit gamma	N	N	N	N	N	N
WP_012511996.1	L-seryl-tRNA(Sec) selenium transferase	N	N	N	N	N	N
WP_012512076.1	LysR family transcriptional regulator	N	N	N	N	N	N
WP_012512133.1	LTA synthase family protein	N	N	N	N	N	N
WP_012512134.1	HAD family hydrolase	N	N	N	N	N	N
WP_012512144.	Gfo/Idh/MocA family oxidoreductase	N	N	N	N	N	N

1							
WP_012512188.1	MFS transporter	N	N	N	N	N	N
WP_012512192.1	CmlA/FloR family chloramphenicol efflux MFS transporter	N	N	N	N	N	N
WP_012512247.1	response regulator transcription factor	N	N	N	N	N	N
WP_012512248.1	LLM class oxidoreductase	N	N	N	N	N	N
WP_012512249.1	LysR substrate-binding domain-containing protein	N	N	N	N	N	N
WP_012512293.1	hypothetical protein	N	N	N	N	N	N
WP_012512328.1	PDDEXK nuclease domain-containing protein	N	N	N	N	N	N
WP_012512371.1	cytochrome o ubiquinol oxidase subunit III	N	N	N	N	N	N
WP_041864489.1	DMT family transporter	N	N	N	N	N	N
WP_041864536.1	sigma-70 family RNA polymerase sigma factor	N	N	N	N	N	N
WP_041864643.1	hypothetical protein	N	N	N	N	N	N
WP_157628471.1	DUF1064 domain-containing protein	N	N	N	N	N	N
WP_269634792.	helix-turn-helix transcriptional regulator	N	N	N	N	N	N

	1						
	WP_005409726.1	ABC transporter permease	N	N	N	N	N	N
	WP_005411609.1	acyl carrier protein	N	N	N	N	N	N
	WP_005416648.1	RebB family R body protein	N	N	N	N	N	N
	WP_005416649.1	RebB family R body protein	N	N	N	N	N	N
	WP_005416651.1	RebB family R body protein	N	N	N	N	N	N
	WP_006399830.1	cytochrome o ubiquinol oxidase subunit IV	N	N	N	N	N	N
NC_015947.1	WP_006425769.1	EamA family transporter	N	N	N	N	N	N
	WP_006446865.1	RebB family R body protein	N	N	N	N	N	N
	WP_006471116.1	HigA family addiction module antitoxin	N	N	N	N	N	N
	WP_014035532.1	tail fiber protein	N	N	N	N	N	N
	WP_014035533.1	tail fiber protein	N	N	N	N	N	N
	WP_014035634.1	tail assembly protein	N	N	N	N	NC_015947.1-1	100
	WP_014035777.	AAA family ATPase	N	N	N	N	N	N

1							
WP_014035884.1	ABC transporter permease	N	N	N	N	N	N
WP_014036155.1	helix-turn-helix domain-containing protein	N	N	N	N	N	N
WP_014036325.1	LysR family transcriptional regulator	N	N	N	N	N	N
WP_014036326.1	alpha/beta hydrolase	N	N	N	N	N	N
WP_014036440.1	response regulator	N	N	N	N	N	N
WP_014036641.1	NAD(P)H-dependent oxidoreductase	N	N	N	N	N	N
WP_014036642.1	LysR family transcriptional regulator	N	N	N	N	N	N
WP_014036654.1	tyrosine-type recombinase/integrase	N	N	N	N	N	N
WP_014036690.1	putative metallopeptidase	N	N	N	N	N	N
WP_014036691.1	DUF2280 domain-containing protein	N	N	N	N	N	N
WP_014036913.1	GIY-YIG nuclease family protein	N	N	N	N	N	N
WP_014036942.1	amino acid ABC transporter permease/ATP-binding protein	N	N	N	N	N	N
WP_014036977.	dihydrodipicolinate synthase family	N	N	N	N	N	N

1	protein						
WP_014037146.1	type II toxin-antitoxin system RelE/ParE family toxin	N	N	N	N	N	N
WP_014037147.1	HigA family addiction module antitoxin	N	N	N	N	N	N
WP_014037223.1	aromatic alcohol reductase	N	N	N	N	N	N
WP_014037224.1	helix-turn-helix domain-containing protein	N	N	N	N	N	N
WP_014037235.1	LysR family transcriptional regulator	N	N	N	N	N	N
WP_014037293.1	DUF3649 domain-containing protein	N	N	N	N	N	N
WP_014037294.1	PepSY-associated TM helix domain-containing protein	N	N	N	N	N	N
WP_014037326.1	heavy metal response regulator transcription factor	N	N	N	N	N	N
WP_014037327.1	multidrug efflux RND transporter permease subunit	N	N	N	N	N	N
WP_014037334.1	DUF3224 domain-containing protein	N	N	N	N	N	N
WP_014037391.1	MotA/TolQ/ExbB proton channel family protein	N	N	N	N	N	N
WP_014037392.1	DUF2149 domain-containing protein	N	N	N	N	N	N
WP_014037431.	ABC transporter ATP-binding protein	N	N	N	N	N	N

1							
WP_014037455.1	helix-turn-helix domain-containing protein	N	N	N	N	N	N
WP_014037604.1	efflux transporter outer membrane subunit	N	N	N	N	N	N
WP_014037605.1	efflux RND transporter periplasmic adaptor subunit	N	N	N	N	N	N
WP_014037629.1	helix-turn-helix domain-containing protein	N	N	N	N	N	N
WP_014037777.1	glutathione transferase GstA	N	N	N	N	N	N
WP_014038309.1	HigA family addiction module antitoxin	N	N	N	N	N	N
WP_014038310.1	type II toxin-antitoxin system RelE/ParE family toxin	N	N	N	N	N	N
WP_014038700.1	DNA-binding transcriptional regulator	N	N	N	N	N	N
WP_042358454.1	DinB family protein	N	N	N	N	N	N
WP_042358552.1	beta-ketoacyl-ACP synthase II	N	N	N	N	N	N
WP_042358872.1	metalloregulator ArsR/SmtB family transcription factor	N	N	N	N	N	N
WP_042359232.1	iron ABC transporter permease	N	N	N	N	N	N
WP_198283165.	LysR family transcriptional regulator	N	N	N	N	N	N

	1							
	WP_232289626.1	GFA family protein	N	N	N	N	N	N
	WP_232289656.1	nucleotidyl transferase AbiEii/AbiGii toxin family protein	N	N	N	N	N	N
	WP_269744744.1	NAD-binding protein	N	N	N	N	N	N
	WP_003050225.1	TIGR03758 family integrating conjugative element protein	N	N	N	N	N	N
	WP_003050245.1	helix-turn-helix domain-containing protein	N	N	N	N	N	N
	WP_003050273.1	CBASS effector endonuclease NucC	N	N	N	N	N	N
	WP_003050422.1	hypothetical protein	N	N	N	N	N	N
NC_017671.1	WP_003090093.1	AlpA family transcriptional regulator	N	N	N	N	N	N
	WP_003090097.1	DUF2857 domain-containing protein	N	N	N	N	N	N
	WP_003090159.1	type III CBASS phage resistance system CD-NTase-associated protein Cap7	N	N	N	N	N	N
	WP_003090173.1	TIGR03745 family integrating conjugative element membrane protein	N	N	N	N	N	N
	WP_003090202.1	thioredoxin domain-containing protein	N	N	N	N	N	N
	WP_003090203.	JAB domain-containing protein	N	N	N	N	N	N

1							
WP_003090212.1	hypothetical protein	N	N	N	N	N	N
WP_003090214.1	conjugal transfer protein TraG N-terminal domain-containing protein	N	N	N	N	N	N
WP_003090319.1	copper homeostasis periplasmic binding protein CopC	N	N	N	N	N	N
WP_003097515.1	copper resistance system multicopper oxidase	N	N	N	N	N	N
WP_003097526.1	helix-turn-helix transcriptional regulator	N	N	N	N	N	N
WP_003097544.1	IS21-like element ISPa36 family helper ATPase IstB	N	N	N	N	N	N
WP_003098886.1	TIGR03759 family integrating conjugative element protein	N	N	N	N	N	N
WP_003098890.1	PilL N-terminal domain-containing protein	N	N	N	N	N	N
WP_003098932.1	mercury(II) reductase	N	N	N	N	N	N
WP_003098939.1	Hg(II)-responsive transcriptional regulator	N	N	N	N	N	N
WP_003098941.1	hypothetical protein	N	N	N	N	N	N
WP_003098955.1	signal peptidase II	N	N	N	N	N	N
WP_003098961.	heavy metal translocating P-type ATPase	N	N	N	N	N	N

1							
WP_003098965.1	Cd(II)/Pb(II)-responsive transcriptional regulator	N	N	N	N	N	N
WP_003098972.1	cation transporter	N	N	N	N	N	N
WP_003098976.1	single-stranded DNA-binding protein	N	N	N	N	N	N
WP_003098988.1	ParA family protein	NZ_CP060023.1-2	100	N	N	N	N
WP_003098991.1	hypothetical protein	NZ_CP060023.1-2	100	N	N	N	N
WP_003098996.1	DUF305 domain-containing protein	N	N	N	N	N	N
WP_003105079.1	conjugative transfer ATPase	N	N	N	N	N	N
WP_003105624.1	CBASS oligonucleotide cyclase	N	N	N	N	N	N
WP_003105629.1	AAA family ATPase	N	N	N	N	N	N
WP_003105639.1	TIGR03750 family conjugal transfer protein	N	N	N	N	N	N
WP_003105641.1	TIGR03746 family integrating conjugative element protein	N	N	N	N	N	N
WP_003105643.1	TIGR03749 family integrating conjugative element protein	N	N	N	N	N	N
WP_003107233.	chromate efflux transporter	N	N	N	N	N	N

1							
WP_003107241.<br>1	arsenate reductase ArsC	N	N	N	N	N	N
WP_003107243.<br>1	ArsI/CadI family heavy metal resistance metalloenzyme	N	N	N	N	N	N
WP_003109768.<br>1	MobH family relaxase	N	N	N	N	N	N
WP_003109770.<br>1	integrating conjugative element protein	N	N	N	N	N	N
WP_003109772.<br>1	TIGR03757 family integrating conjugative element protein	N	N	N	N	N	N
WP_003109775.<br>1	TIGR03751 family conjugal transfer lipoprotein	N	N	N	N	N	N
WP_003109779.<br>1	integrating conjugative element protein	N	N	N	N	N	N
WP_003109780.<br>1	transglycosylase SLT domain-containing protein	N	N	N	N	N	N
WP_003116797.<br>1	copper homeostasis membrane protein CopD	N	N	N	N	N	N
WP_003116799.<br>1	helix-turn-helix domain-containing protein	N	N	N	N	N	N
WP_003116819.<br>1	DUF6094 domain-containing protein	N	N	N	N	N	N
WP_003116820.<br>1	hypothetical protein	N	N	N	N	N	N
WP_003116821.	hypothetical protein	N	N	N	N	N	N

1							
WP_003116823.1	DUF3275 family protein	N	N	N	N	N	N
WP_003116824.1	DUF932 domain-containing protein	N	N	N	N	N	N
WP_003116825.1	DUF3577 domain-containing protein	N	N	N	N	N	N
WP_003116826.1	mercury resistance system periplasmic binding protein MerP	N	N	N	N	N	N
WP_003116827.1	mercuric ion transporter MerT	N	N	N	N	N	N
WP_003116832.1	hypothetical protein	N	N	N	N	N	N
WP_003116834.1	DNA topoisomerase III	N	N	N	N	N	N
WP_003116836.1	TIGR03761 family integrating conjugative element protein	N	N	N	N	N	N
WP_003116837.1	STY4528 family pathogenicity island replication protein	N	N	N	N	N	N
WP_004350508.1	ParB family protein	N	N	N	N	N	N
WP_005408883.1	copper resistance protein B	N	N	N	N	N	N
WP_005416648.1	RebB family R body protein	N	N	N	N	N	N
WP_005416649.	RebB family R body protein	N	N	N	N	N	N

1							
WP_005416651.1	RebB family R body protein	N	N	N	N	N	N
WP_006446865.1	RebB family R body protein	N	N	N	N	N	N
WP_008264906.1	HupE/UreJ family protein	N	N	N	N	N	N
WP_014645488.1	NADPH-dependent FMN reductase	N	N	N	N	N	N
WP_014645495.1	IS21-like element ISPa36 family transposase	N	N	N	N	N	N
WP_014645518.1	CusA/CzcA family heavy metal efflux RND transporter	N	N	N	N	N	N
WP_014645845.1	ABC transporter ATP-binding protein	N	N	N	N	N	N
WP_014646102.1	site-specific integrase	N	N	N	N	N	N
WP_014646104.1	helix-turn-helix transcriptional regulator	N	N	N	N	N	N
WP_014646105.1	ABC-three component system protein	N	N	N	N	N	N
WP_014646111.1	DUF932 domain-containing protein	N	N	N	N	N	N
WP_014646112.1	ParB/RepB/Spo0J family partition protein	N	N	N	N	N	N
WP_014646113.	hypothetical protein	N	N	N	N	N	N

1							
WP_014646114.1	DUF736 domain-containing protein	N	N	N	N	N	N
WP_014646117.1	DUF2285 domain-containing protein	N	N	N	N	N	N
WP_014646118.1	helix-turn-helix domain-containing protein	N	N	N	N	N	N
WP_014646121.1	hypothetical protein	N	N	N	N	N	N
WP_014646122.1	DUF2840 domain-containing protein	N	N	N	N	N	N
WP_014646123.1	S26 family signal peptidase	N	N	N	N	N	N
WP_014646132.1	efflux RND transporter permease subunit	N	N	N	N	N	N
WP_014646133.1	efflux RND transporter permease subunit	N	N	N	N	N	N
WP_014646135.1	response regulator	N	N	N	N	N	N
WP_014646139.1	LysR family transcriptional regulator	N	N	N	N	N	N
WP_014646140.1	EexN family lipoprotein	N	N	N	N	N	N
WP_014646142.1	ribbon-helix-helix protein, CopG family	N	N	N	N	N	N
WP_014646144.	TrbC/VirB2 family protein	N	N	N	N	N	N

1							
WP_014646145.1	VirB3 family type IV secretion system protein	N	N	N	N	N	N
WP_014646147.1	P-type conjugative transfer protein TrbJ	N	N	N	N	N	N
WP_014646148.1	hypothetical protein	N	N	N	N	N	N
WP_014646149.1	P-type conjugative transfer protein TrbL	N	N	N	N	N	N
WP_014646150.1	conjugal transfer protein TrbF	N	N	N	N	N	N
WP_014646612.1	IS21-like element ISPa36 family helper ATPase IstB	N	N	N	N	N	N
WP_014646619.1	DUF411 domain-containing protein	N	N	N	N	N	N
WP_014646627.1	TIGR03747 family integrating conjugative element membrane protein	N	N	N	N	N	N
WP_014646628.1	helicase-related protein	N	N	N	N	N	N
WP_014646630.1	hypothetical protein	N	N	N	N	N	N
WP_014646631.1	DUF3158 family protein	N	N	N	N	N	N
WP_014646867.1	DEAD/DEAH box helicase family protein	N	N	N	N	N	N
WP_014646868.	class I SAM-dependent DNA	N	N	N	N	N	N

1	methyltransferase						
WP_014647161.1	SDR family oxidoreductase	N	N	N	N	N	N
WP_014647272.1	TIGR03885 family FMN-dependent LLM class oxidoreductase	N	N	N	N	N	N
WP_014648138.1	GPW/gp25 family protein	N	N	N	N	N	N
WP_014648141.1	phage tail assembly protein	N	N	N	N	N	N
WP_014648144.1	phage tail protein	N	N	N	N	N	N
WP_014648710.1	IS21-like element ISPa36 family transposase	N	N	N	N	N	N
WP_014648728.1	cytochrome o ubiquinol oxidase subunit IV	N	N	N	N	N	N
WP_014747691.1	PIN domain-containing protein	N	N	N	N	N	N
WP_014833982.1	LysR family transcriptional regulator	N	N	N	N	N	N
WP_015014846.1	PadR family transcriptional regulator	N	N	N	N	N	N
WP_026069910.1	TrbI/VirB10 family protein	N	N	N	N	N	N
WP_031629745.1	DUF3085 domain-containing protein	N	N	N	N	N	N
WP_032957905.	glycosyltransferase family 2 protein	N	N	N	N	N	N

1							
WP_032962148.1	type II toxin-antitoxin system RelE/ParE family toxin	N	N	N	N	N	N
WP_033835782.1	hypothetical protein	N	N	N	N	N	N
WP_033836038.1	MFS transporter	N	N	N	N	N	N
WP_034010229.1	type II toxin-antitoxin system RelE/ParE family toxin	N	N	N	N	N	N
WP_034010237.1	DUF2958 domain-containing protein	N	N	N	N	N	N
WP_041863421.1	heavy metal translocating P-type ATPase	N	N	N	N	N	N
WP_051007148.1	helix-turn-helix transcriptional regulator	N	N	N	N	N	N
WP_086009121.1	IS5-like element ISStma16 family transposase	N	N	N	N	N	N
WP_086009122.1	IS3-like element ISPa39 family transposase	N	N	N	N	N	N
WP_087948673.1	replication initiator protein A	N	N	N	N	N	N
WP_087948675.1	IS3-like element ISStma17 family transposase	N	N	N	N	N	N
WP_099475629.1	ABC transporter permease	N	N	N	N	N	N
WP_168356115.	HupE/UreJ family protein	N	N	N	N	N	N

	1							
	WP_228777907.1	heavy metal translocating P-type ATPase	N	N	N	N	N	N
	WP_229653766.1	GlxA family transcriptional regulator	N	N	N	N	N	N
	WP_229766898.1	P-type conjugative transfer protein TrbG	N	N	N	N	N	N
	WP_230374612.1	DUF2326 domain-containing protein	N	N	N	N	N	N
	WP_232503920.1	Rid family hydrolase	N	N	N	N	N	N
	WP_004135208.1	LysR substrate-binding domain-containing protein	N	N	N	N	N	N
	WP_004135210.1	carboxymuconolactone decarboxylase family protein	N	N	N	N	N	N
	WP_005416648.1	RebB family R body protein	N	N	N	N	N	N
NZ_AP021867.1	WP_005416651.1	RebB family R body protein	N	N	N	N	N	N
	WP_006368769.1	RebB family R body protein	N	N	N	N	N	N
	WP_006368771.1	RebB family R body protein	N	N	N	N	N	N
	WP_006389651.1	Fic family protein	N	N	N	N	N	N
	WP_006395487.	M24 family metallopeptidase	N	N	N	N	N	N

1							
WP_012509718.1	tautomerase family protein	N	N	N	N	N	N
WP_012510145.1	VOC family protein	N	N	N	N	N	N
WP_012510368.1	helix-turn-helix domain-containing GNAT family N-acetyltransferase	N	N	N	N	N	N
WP_012510370.1	isocitrate lyase/PEP mutase family protein	N	N	N	N	N	N
WP_032972196.1	GrpB family protein	N	N	N	N	N	N
WP_065722706.1	ABC transporter permease	N	N	N	N	N	N
WP_065722713.1	NAD(P)/FAD-dependent oxidoreductase	N	N	N	N	N	N
WP_080112492.1	helix-turn-helix domain-containing protein	N	N	N	N	N	N
WP_153674910.1	HipA domain-containing protein	N	N	N	N	N	N
WP_153675367.1	AAA family ATPase	N	N	N	N	N	N
WP_153675369.1	ATP-dependent helicase	N	N	N	N	N	N
WP_153675373.1	HsdR family type I site-specific deoxyribonuclease	N	N	N	N	N	N
WP_153675389.	response regulator	N	N	N	N	N	N

	1							
	WP_153675392.1	TlpA disulfide reductase family protein	N	N	N	N	N	N
	WP_153675530.1	alpha/beta fold hydrolase	N	N	N	N	N	N
	WP_153675619.1	LysR family transcriptional regulator	N	N	N	N	N	N
	WP_153675627.1	DHA2 family efflux MFS transporter permease subunit	N	N	N	N	N	N
	WP_153676233.1	NAD(P)H-dependent oxidoreductase	N	N	N	N	N	N
	WP_217491433.1	glycosyltransferase family 2 protein	N	N	N	N	N	N
	WP_232053912.1	MFS transporter	N	N	N	N	N	N
	WP_232053924.1	helix-turn-helix transcriptional regulator	N	N	N	N	N	N
	WP_232053992.1	AAA family ATPase	N	N	N	N	N	N
	WP_232053994.1	S8 family peptidase	N	N	N	N	N	N
	WP_232054050.1	pirin family protein	N	N	N	N	N	N
NZ_AP021908.1	WP_000414383.1	mercury resistance transcriptional regulator MerR	N	N	N	N	N	N
	WP_000732290.	mercury resistance system periplasmic	N	N	N	N	N	N

1	binding protein MerP						
WP_000995360.1	mercury resistance co-regulator MerD	N	N	N	N	N	N
WP_001294666.1	mercuric transport protein MerT	N	N	N	N	N	N
WP_002718009.1	chlorite dismutase family protein	N	N	N	N	N	N
WP_003097546.1	IS21-like element ISPa36 family transposase	N	N	N	N	N	N
WP_003100847.1	recombinase family protein	N	N	N	N	N	N
WP_003100872.1	DUF86 domain-containing protein	N	N	N	N	N	N
WP_003132004.1	broad-spectrum mercury transporter MerE	N	N	N	N	N	N
WP_003141029.1	hypothetical protein	N	N	N	N	N	N
WP_003141085.1	LysR family transcriptional regulator	N	N	N	N	N	N
WP_003282197.1	helix-turn-helix transcriptional regulator	N	N	N	N	N	N
WP_003294141.1	DUF3275 family protein	N	N	N	N	N	N
WP_003294215.1	TIGR03745 family integrating conjugative element membrane protein	N	N	N	N	N	N
WP_003454927.	AlpA family transcriptional regulator	N	N	N	N	N	N

1							
WP_003465043.1	cupin domain-containing protein	N	N	N	N	N	N
WP_004265510.1	ParA family protein	N	N	NZ_AP021908.1-3	100	N	N
WP_005408332.1	GPW/gp25 family protein	N	N	N	N	N	N
WP_005409664.1	heat resistance protein YfdX1	NZ_CP060026.1-2	100	N	N	NZ_CP060025.1-2, NZ_CP060027.1-4, NZ_CP102942.1-4, NZ_CP098483.1-4	100
WP_005409665.1	small heat shock protein sHSP20-GI	N	N	N	N	N	N
WP_008647302.1	sulfite oxidase-like oxidoreductase	N	N	N	N	N	N
WP_009288528.1	TniQ family protein	N	N	N	N	N	N
WP_010792128.1	TIGR03758 family integrating conjugative element protein	N	N	N	N	N	N
WP_010792177.1	DUF2857 domain-containing protein	N	N	N	N	N	N
WP_010890110.1	transcriptional regulator KorA	N	N	N	N	N	N
WP_010890115.1	transcriptional repressor KorC	N	N	N	N	N	N
WP_010890153.	type II toxin-antitoxin system RelE/ParE	N	N	N	N	N	N

1	family toxin						
WP_011494284.1	DDE-type integrase/transposase/recombinase	N	N	N	N	N	N
WP_011494285.1	TniB family NTP-binding protein	N	N	N	N	N	N
WP_011829954.1	hypothetical protein	N	N	N	N	N	N
WP_012761373.1	zinc metalloprotease HtpX	N	N	N	N	N	N
WP_012761383.1	Hsp20/alpha crystallin family protein	N	N	NZ_AP021908.1-3	100	N	N
WP_012761385.1	LysR family transcriptional regulator	N	N	NZ_AP021908.1-3	100	N	N
WP_016446144.1	nucleotidyl transferase AbiEii/AbiGii toxin family protein	N	N	N	N	N	N
WP_016446188.1	IS110 family transposase	N	N	N	N	N	N
WP_016851994.1	hypothetical protein	N	N	N	N	N	N
WP_019185707.1	SLC13 family permease	N	N	N	N	N	N
WP_019484523.1	OST-HTH/LOTUS domain-containing protein	N	N	N	N	N	N
WP_019486762.1	DUF3158 family protein	N	N	N	N	N	N
WP_020309197.	carboxymuconolactone decarboxylase	N	N	N	N	N	N

1	family protein						
WP_021201945.1	hypothetical protein	N	N	N	N	N	N
WP_023083180.1	single-stranded DNA-binding protein	N	N	N	N	N	N
WP_023093118.1	RAQPRD family integrative conjugative element protein	N	N	N	N	N	N
WP_023109284.1	tyrosine-type recombinase/integrase	N	N	N	N	N	N
WP_023109285.1	substrate-binding domain-containing protein	N	N	N	N	N	N
WP_023109290.1	hypothetical protein	N	N	N	N	N	N
WP_023109291.1	integrating conjugative element protein	N	N	N	N	N	N
WP_023109293.1	TIGR03757 family integrating conjugative element protein	N	N	N	N	N	N
WP_023109294.1	DNA repair protein RadC	N	N	N	N	N	N
WP_023109295.1	thioredoxin domain-containing protein	N	N	N	N	N	N
WP_023109297.1	TIGR03751 family conjugal transfer lipoprotein	N	N	N	N	N	N
WP_023109299.1	TIGR03749 family integrating conjugative element protein	N	N	N	N	N	N
WP_023109300.	TIGR03746 family integrating	N	N	N	N	N	N

1	conjugative element protein						
WP_023109301.1	TIGR03750 family conjugal transfer protein	N	N	N	N	N	N
WP_023109303.1	SNF2-related protein	N	N	N	N	N	N
WP_023109307.1	DEAD/DEAH box helicase family protein	N	N	N	N	N	N
WP_023109309.1	TIGR03747 family integrating conjugative element membrane protein	N	N	N	N	N	N
WP_023109311.1	integrating conjugative element protein	N	N	N	N	N	N
WP_023109312.1	hypothetical protein	N	N	N	N	N	N
WP_023109313.1	TIGR03759 family integrating conjugative element protein	N	N	N	N	N	N
WP_023109314.1	hypothetical protein	N	N	N	N	N	N
WP_023109315.1	integrating conjugative element protein PilL, PFGI-1 class	N	N	N	N	N	N
WP_023109318.1	STY4534 family ICE replication protein	N	N	N	N	N	N
WP_023109319.1	DUF3085 domain-containing protein	N	N	N	N	N	N
WP_023109320.1	hypothetical protein	N	N	N	N	N	N
WP_023109321.	hypothetical protein	N	N	N	N	N	N

1							
WP_023109322.1	hypothetical protein	N	N	N	N	N	N
WP_023109323.1	hypothetical protein	N	N	N	N	N	N
WP_023109325.1	STY4528 family pathogenicity island replication protein	N	N	N	N	N	N
WP_023109326.1	ParB family protein	N	N	N	N	N	N
WP_023109327.1	hypothetical protein	N	N	N	N	N	N
WP_023109332.1	heat resistance system K+/H+ antiporter KefB-GI	N	N	N	N	N	N
WP_025298038.1	TIGR03752 family integrating conjugative element protein	N	N	N	N	N	N
WP_031633433.1	DUF3742 family protein	N	N	N	N	N	N
WP_031633437.1	helix-turn-helix domain-containing protein	N	N	N	N	N	N
WP_031633439.1	site-specific DNA-methyltransferase	N	N	N	N	N	N
WP_031754973.1	type IV toxin-antitoxin system AbiEi family antitoxin domain-containing protein	N	N	N	N	N	N
WP_031943937.1	mercury(II) reductase	N	N	N	N	N	N

WP_032961836.1	phage terminase large subunit	NZ_CM001824.1-1, NZ_CP014014.1-2, NZ_LR134324.1-1	100	NZ_AP021908.1-1, NZ_CP040431.1-2, NZ_CP040439.1-4, NZ_CP043578.1-1, NZ_CP056088.1-2, NZ_CP060027.1-3, NZ_LR134324.1-3, NZ_CP090418.1-2, NZ_CP104863.1-3, NZ_CP102942.1-3, NZ_CP098483.1-2	100	NZ_CP040430.1-2	100
WP_034053978.1	redoxin domain-containing protein	N	N	N	N	N	N
WP_034053981.1	DUF427 domain-containing protein	N	N	N	N	N	N
WP_047306875.1	AraC family transcriptional regulator	N	N	N	N	N	N
WP_049259625.1	hypothetical protein	N	N	N	N	N	N
WP_049259627.1	heat resistance protein YfdX2	N	N	N	N	N	N
WP_049419328.1	tRNA-guanine transglycosylase DpdA	N	N	N	N	N	N
WP_049419332.1	7-carboxy-7-deazaguanine synthase	N	N	N	N	N	N
WP_049419335.	7-cyano-7-deazaguanine synthase QueC	N	N	N	N	N	N

1							
WP_065426719.1	hypothetical protein	N	N	N	N	N	N
WP_074423286.1	YdiU family protein	N	N	N	N	N	N
WP_087748587.1	Tn3 family transposase	N	N	N	N	N	N
WP_099421609.1	IS5 family transposase	N	N	N	N	N	N
WP_099497898.1	LuxR C-terminal-related transcriptional regulator	N	N	N	N	N	N
WP_149500783.1	IS3 family transposase	N	N	N	N	N	N
WP_149500785.1	alpha/beta-hydrolase family protein	N	N	N	N	N	N
WP_149501021.1	DUF6088 family protein	N	N	N	N	N	N
WP_164146001.1	patatin-like phospholipase family protein	N	N	N	N	N	N
WP_164146003.1	glutathione S-transferase N-terminal domain-containing protein	N	N	N	N	N	N
WP_164146004.1	magnesium and cobalt transport protein CorA	N	N	N	N	N	N
WP_180869384.1	GntP family permease	N	N	N	N	N	N
WP_183153555.	heat resistance protein PsiE-GI	N	N	N	N	N	N

1							
WP_183153556.1	HdeD family acid-resistance protein	N	N	N	N	N	N
WP_183153558.1	cardiolipin synthase	N	N	N	N	N	N
WP_183153559.1	TIGR03761 family integrating conjugative element protein	N	N	N	N	N	N
WP_183153562.1	IS30 family transposase	N	N	N	N	N	N
WP_183153566.1	IS21 family transposase	N	N	N	N	N	N
WP_183153567.1	IS21-like element helper ATPase IstB	N	N	N	N	N	N
WP_183153569.1	Tn3-like element IS1071 family transposase	N	N	N	N	N	N
WP_183153616.1	VWA domain-containing protein	N	N	N	N	N	N
WP_183153619.1	ATP-binding protein	N	N	N	N	N	N
WP_183153724.1	IS3-like element ISPa39 family transposase	N	N	N	N	N	N
WP_183153825.1	LysR substrate-binding domain-containing protein	N	N	N	N	N	N
WP_183153828.1	transposase	N	N	N	N	N	N
WP_183153913.	AlpA family phage regulatory protein	N	N	N	N	N	N

	1							
	WP_183153949.1	transposase	N	N	N	N	N	N
	WP_197022694.1	ergothioneine biosynthesis protein EgtB	N	N	N	N	N	N
	WP_197599548.1	tyrosine-type recombinase/integrase	N	N	N	N	N	N
	WP_197970337.1	isocitrate lyase/phosphoenolpyruvate mutase family protein	N	N	N	N	N	N
	WP_197970340.1	anti-phage ATPase IteA	N	N	N	N	N	N
	WP_197970341.1	nucleotidyl transferase AbiEii/AbiGii toxin family protein	N	N	N	N	N	N
	WP_227415191.1	nuclear transport factor 2 family protein	N	N	N	N	N	N
	WP_232092420.1	ATP-binding protein	N	N	NZ_AP021908.1-3	100	N	N
NZ_CM001824.1	WP_000761850.1	organomercurial lyase MerB	N	N	N	N	N	N
	WP_000995360.1	mercury resistance co-regulator MerD	N	N	N	N	N	N
	WP_000995361.1	mercury resistance co-regulator MerD	N	N	N	N	N	N
	WP_003131974.1	mercuric ion transporter MerT	N	N	N	N	N	N
	WP_003131987.	mercury resistance system periplasmic	N	N	N	N	N	N

1	binding protein MerP						
WP_003132004.1	broad-spectrum mercury transporter MerE	N	N	N	N	N	N
WP_003830788.1	DDE-type integrase/transposase/recombinase	N	N	N	N	N	N
WP_003830789.1	TniB family NTP-binding protein	N	N	N	N	N	N
WP_005408332.1	GPW/gp25 family protein	N	N	N	N	N	N
WP_005409675.1	AraC family transcriptional regulator	N	N	N	N	N	N
WP_005411473.1	cytochrome o ubiquinol oxidase subunit IV	N	N	N	N	N	N
WP_005412859.1	YqaJ viral recombinase family protein	N	N	NZ_CM001824.1-5	100	N	N
WP_005412860.1	recombinase RecT	N	N	NZ_CM001824.1-5	100	N	N
WP_005412881.1	DUF2280 domain-containing protein	N	N	N	N	N	N
WP_005412900.1	lysozyme	N	N	N	N	N	N
WP_005413068.1	hypothetical protein	N	N	N	N	N	N
WP_005413069.1	hypothetical protein	N	N	N	N	N	N
WP_005413073.	P-type conjugative transfer protein TrbL	N	N	N	N	N	N

1							
WP_005413074.1	hypothetical protein	N	N	N	N	N	N
WP_005413084.1	SDR family oxidoreductase	N	N	N	N	N	N
WP_005413088.1	S26 family signal peptidase	N	N	N	N	N	N
WP_005413093.1	helix-turn-helix domain-containing protein	N	N	N	N	N	N
WP_005413094.1	DUF2285 domain-containing protein	N	N	N	N	N	N
WP_005413097.1	DUF736 family protein	N	N	N	N	N	N
WP_005413100.1	DUF2958 domain-containing protein	N	N	N	N	N	N
WP_005413102.1	hypothetical protein	N	N	N	N	N	N
WP_005413103.1	DEAD/DEAH box helicase	N	N	N	N	N	N
WP_005413104.1	Qat anti-phage system ATPase QatA	N	N	N	N	N	N
WP_005413106.1	Qat anti-phage system QueC-like protein QatC	N	N	N	N	N	N
WP_005413341.1	replication-associated recombination protein A	N	N	N	N	N	N
WP_005413343.	DUF3422 family protein	N	N	N	N	N	N

1							
WP_005413344.1	excinuclease ABC subunit UvrB	N	N	N	N	N	N
WP_005413387.1	Hg(II)-responsive transcriptional regulator	N	N	N	N	N	N
WP_005413389.1	TniQ family protein	N	N	N	N	N	N
WP_005413391.1	DUF3330 domain-containing protein	N	N	N	N	N	N
WP_005413392.1	broad-spectrum mercury transporter MerE	N	N	N	N	N	N
WP_005413394.1	organomercurial transporter MerC	N	N	N	N	N	N
WP_005413396.1	mercury resistance system periplasmic binding protein MerP	N	N	N	N	N	N
WP_005413398.1	Hg(II)-responsive transcriptional regulator	N	N	N	N	N	N
WP_005413453.1	hypothetical protein	N	N	N	N	N	N
WP_005413455.1	SDR family oxidoreductase	N	N	N	N	N	N
WP_005413570.1	heavy metal response regulator transcription factor	N	N	N	N	N	N
WP_005414746.1	HlyD family efflux transporter periplasmic adaptor subunit	N	N	N	N	N	N
WP_005414747.	ATP-binding cassette domain-containing	N	N	N	N	N	N

1	protein						
WP_005414983.1	ATP-binding protein	N	N	N	N	N	N
WP_005414984.1	MFS transporter	N	N	N	N	N	N
WP_012478642.1	UvrD-helicase domain-containing protein	N	N	N	N	N	N
WP_032961836.1	phage terminase large subunit	NZ_CM001824.1-1, NZ_CP014014.1-2, NZ_LR134324.1-1	100	NZ_AP021908.1-1, NZ_CP040431.1-2, NZ_CP040439.1-4, NZ_CP043578.1-1, NZ_CP056088.1-2, NZ_CP060027.1-3, NZ_LR134324.1-3, NZ_CP090418.1-2, NZ_CP104863.1-3, NZ_CP102942.1-3, NZ_CP098483.1-2	100	NZ_CP040430.1-2	100
WP_032966054.1	helix-turn-helix domain-containing protein	N	N	N	N	N	N
WP_032966379.1	DNA cytosine methyltransferase	N	N	N	N	N	N
WP_032966544.1	nucleotide pyrophosphohydrolase	N	N	N	N	N	N
WP_032966653.1	hypothetical protein	N	N	N	N	N	N

WP_032966655.1	GIY-YIG nuclease family protein	N	N	N	N	N	N
WP_032966658.1	hypothetical protein	N	N	N	N	N	N
WP_032966666.1	EexN family lipoprotein	N	N	N	N	N	N
WP_032966671.1	DoxX family protein	N	N	N	N	N	N
WP_032966672.1	DoxX family protein	N	N	N	N	N	N
WP_032966674.1	NAD(P)H-binding protein	N	N	N	N	N	N
WP_032966675.1	helix-turn-helix domain-containing protein	N	N	N	N	N	N
WP_032966679.1	hypothetical protein	N	N	N	N	N	N
WP_032966680.1	Qat anti-phage system associated protein QatB	N	N	N	N	N	N
WP_032966681.1	Qat anti-phage system TatD family nuclease QatD	N	N	N	N	N	N
WP_032966722.1	hypothetical protein	N	N	N	N	N	N
WP_032966740.1	NAD(P)/FAD-dependent oxidoreductase	N	N	N	N	N	N
WP_032966744.1	bestrophin family protein	N	N	N	N	N	N

WP_032966791.1	SDR family oxidoreductase	N	N	N	N	N	N
WP_032967187.1	metalloregulator ArsR/SmtB family transcription factor	N	N	N	N	N	N
WP_032967276.1	hypothetical protein	N	N	N	N	N	N
WP_045890798.1	DUF3330 domain-containing protein	N	N	N	N	N	N
WP_046272807.1	universal stress protein	NZ_CP051467.1-3	100	N	N	N	N
WP_050557873.1	MBL fold metallo-hydrolase	N	N	N	N	N	N
WP_065426719.1	hypothetical protein	N	N	N	N	N	N
WP_080101779.1	very short patch repair endonuclease	N	N	N	N	N	N
WP_080101801.1	helix-turn-helix transcriptional regulator	N	N	N	N	N	N
WP_080101842.1	sigma-54 dependent transcriptional regulator	N	N	N	N	N	N
WP_232418880.1	SDR family oxidoreductase	N	N	N	N	N	N
WP_232418972.1	putative metallopeptidase	N	N	N	N	N	N
WP_232418980.1	AlpA family phage regulatory protein	N	N	N	N	N	N

NZ_CP008838.1	WP_232418992.1	helicase-related protein	N	N	N	N	N	N
	WP_004146420.1	BREX protein BrxB domain-containing protein	N	N	N	N	N	N
	WP_004153231.1	DEAD/DEAH box helicase family protein	N	N	N	N	N	N
	WP_005304583.1	helix-turn-helix transcriptional regulator	N	N	N	N	N	N
	WP_005408112.1	LysR family transcriptional regulator	N	N	N	N	N	N
	WP_005408332.1	GPW/gp25 family protein	N	N	N	N	N	N
	WP_005408904.1	nuclear transport factor 2 family protein	N	N	N	N	N	N
	WP_005411473.1	cytochrome o ubiquinol oxidase subunit IV	N	N	N	N	N	N
	WP_006375900.1	zincin-like metallopeptidase domain-containing protein	N	N	N	N	N	N
	WP_012478672.1	helix-turn-helix transcriptional regulator	N	N	N	N	N	N
	WP_012479056.1	ABC transporter ATP-binding protein	N	N	N	N	N	N
	WP_012479074.1	SMR family transporter	N	N	N	N	N	N
	WP_012479075.1	TIM barrel protein	N	N	N	N	N	N

WP_012479076.1	NAD(P)-dependent oxidoreductase	N	N	N	N	N	N
WP_012479077.1	FAD-dependent oxidoreductase	N	N	N	N	N	N
WP_012479078.1	glycosyltransferase	N	N	N	N	N	N
WP_020200523.1	DUF6127 family protein	N	N	N	N	N	N
WP_020200576.1	helix-turn-helix transcriptional regulator	N	N	N	N	N	N
WP_024956190.1	GPW/gp25 family protein	N	N	N	N	N	N
WP_024956193.1	phage tail assembly protein	N	N	N	N	N	N
WP_024956393.1	Hg(II)-responsive transcriptional regulator	N	N	N	N	N	N
WP_024956394.1	mercuric transporter MerT family protein	N	N	N	N	N	N
WP_024956395.1	mercury resistance system periplasmic binding protein MerP	N	N	N	N	N	N
WP_024956414.1	hypothetical protein	N	N	N	N	N	N
WP_024956557.1	alpha/beta hydrolase	N	N	N	N	N	N
WP_024956711.1	DUF2958 domain-containing protein	N	N	N	N	N	N

WP_024956712.1	DUF2285 domain-containing protein	N	N	NZ_CP060022.1-9, NZ_CP060023.1-9, NZ_CP065965.1-5	100	N	N
WP_024956713.1	helix-turn-helix domain-containing protein	N	N	N	N	N	N
WP_024956864.1	adenylate/guanylate cyclase domain-containing protein	N	N	N	N	N	N
WP_024957346.1	glycosyltransferase family 2 protein	N	N	N	N	N	N
WP_024957400.1	DUF305 domain-containing protein	N	N	N	N	N	N
WP_024957601.1	AAA family ATPase	NZ_CP008838.1-2, NZ_LT906480.1-2	100	N	N	NZ_CP065965.1-10	100
WP_024957611.1	hypothetical protein	N	N	N	N	N	N
WP_024957613.1	hypothetical protein	N	N	N	N	N	N
WP_024957746.1	transcriptional regulator	N	N	N	N	N	N
WP_024957931.1	DUF1629 domain-containing protein	N	N	N	N	N	N
WP_024957933.1	metalloregulator ArsR/SmtB family transcription factor	N	N	N	N	N	N
WP_024957960.1	VOC family protein	N	N	N	N	N	N
WP_024957961.	Na+/H+ antiporter NhaA	N	N	N	N	N	N

1							
WP_024958019.1	XRE family transcriptional regulator	N	N	N	N	N	N
WP_024958021.1	DUF3322 and DUF2220 domain-containing protein	N	N	N	N	N	N
WP_024958026.1	BrxE family protein	N	N	N	N	N	N
WP_024958027.1	DUF1819 family protein	N	N	N	N	N	N
WP_024958028.1	BREX system P-loop protein BrxC	N	N	N	N	N	N
WP_024958029.1	N-6 DNA methylase	N	N	N	N	N	N
WP_024958031.1	DUF488 domain-containing protein	N	N	N	N	N	N
WP_024958032.1	PglZ domain-containing protein	NZ_CP008838.1-1	100	N	N	N	N
WP_024958033.1	BREX system Lon protease-like protein BrxL	NZ_CP008838.1-1	100	N	N	N	N
WP_024958034.1	hypothetical protein	N	N	N	N	N	N
WP_024958035.1	DUF2793 domain-containing protein	N	N	N	N	N	N
WP_024958036.1	glycosyltransferase family 2 protein	NZ_CP008838.1-1, NZ_CP065965.1-1, NZ_LT906480.1-5	100	N	N	N	N

WP_024958037.1	phage tail protein	NZ_CP008838.1-1, NZ_CP065965.1-1, NZ_LT906480.1-5	100	N	N	N	N
WP_024958039.1	DUF2163 domain-containing protein	NZ_CP008838.1-1, NZ_CP065965.1-1, NZ_LT906480.1-5	100	N	N	N	N
WP_024958040.1	hypothetical protein	NZ_CP008838.1-1, NZ_CP065965.1-1, NZ_LT906480.1-5	100	N	N	N	N
WP_024958041.1	hypothetical protein	NZ_CP008838.1-1, NZ_CP065965.1-1, NZ_LT906480.1-5	100	N	N	N	N
WP_024958042.1	tail protein	NZ_CP008838.1-1, NZ_CP065965.1-1, NZ_LT906480.1-5	100	N	N	N	N
WP_024958043.1	DUF6441 family protein	NZ_CP008838.1-1, NZ_CP065965.1-1, NZ_LT906480.1-5	100	N	N	N	N
WP_024958044.1	hypothetical protein	N	N	N	N	N	N
WP_024958045.1	hypothetical protein	NZ_CP008838.1-1, NZ_CP065965.1-1, NZ_LT906480.1-5	100	N	N	N	N
WP_024958046.1	hypothetical protein	N	N	N	N	N	N
WP_024958047.	hypothetical protein	N	N	N	N	N	N

1							
WP_024958049.1	head decoration protein	N	N	N	N	N	N
WP_024958050.1	S49 family peptidase	NZ_CP008838.1-1, NZ_CP065965.1-1, NZ_LT906480.1-5	100	NZ_LT906480.1-8	100	N	N
WP_024958052.1	hypothetical protein	N	N	N	N	N	N
WP_024958053.1	hypothetical protein	N	N	N	N	N	N
WP_024958054.1	hypothetical protein	N	N	N	N	N	N
WP_024958058.1	DUF3489 domain-containing protein	NZ_CP008838.1-1, NZ_CP065965.1-1	100	NZ_LT906480.1-8	100	N	N
WP_024958059.1	hypothetical protein	N	N	N	N	N	N
WP_024958060.1	hypothetical protein	N	N	N	N	N	N
WP_024958063.1	DUF6362 family protein	N	N	N	N	N	N
WP_024958064.1	hypothetical protein	N	N	N	N	N	N
WP_024958066.1	phage/plasmid primase, P4 family	NZ_CP008838.1-1, NZ_CP065965.1-1	100	N	N	N	N
WP_024958069.1	DUF6511 domain-containing protein	N	N	N	N	N	N

WP_024958070.1	hypothetical protein	NZ_CP008838.1-1, NZ_CP065965.1-1	100	N	N	N	N
WP_024958074.1	hypothetical protein	N	N	N	N	N	N
WP_024958075.1	hypothetical protein	N	N	N	N	N	N
WP_024958076.1	recombinase family protein	NZ_CP008838.1-1, NZ_CP065965.1-1	100	N	N	N	N
WP_024958077.1	DUF2924 domain-containing protein	N	N	N	N	N	N
WP_024958080.1	ImmA/IrrE family metallo-endopeptidase	N	N	N	N	N	N
WP_024958081.1	hypothetical protein	NZ_CP008838.1-1	100	N	N	N	N
WP_024958082.1	hypothetical protein	NZ_CP008838.1-1	100	N	N	N	N
WP_024958541.1	YafY family protein	N	N	N	N	N	N
WP_024958791.1	ISL3 family transposase	N	N	N	N	N	N
WP_032963337.1	IS3 family transposase	N	N	N	N	N	N
WP_032964292.1	DUF488 domain-containing protein	N	N	N	N	N	N
WP_032964294.1	lysozyme	N	N	N	N	N	N

WP_032964308.1	site-specific DNA-methyltransferase	N	N	N	N	N	N
WP_076738308.1	ATP-binding protein	N	N	N	N	N	N
WP_076738309.1	3'-5' exonuclease	N	N	N	N	N	N
WP_076738390.1	DNA mismatch endonuclease Vsr	N	N	N	N	N	N
WP_076738391.1	DNA cytosine methyltransferase	N	N	N	N	N	N
WP_076738392.1	DUF262 domain-containing protein	N	N	N	N	N	N
WP_143568593.1	hypothetical protein	N	N	N	N	N	N
WP_197697519.1	DNA adenine methylase	N	N	N	N	N	N
WP_197697522.1	YbhB/YbcL family Raf kinase inhibitor-like protein	N	N	N	N	N	N
WP_197697523.1	CBASS oligonucleotide cyclase	N	N	N	N	N	N
WP_223224752.1	DUF3422 family protein	N	N	N	N	N	N
WP_224119399.1	SMI1/KNR4 family protein	N	N	N	N	N	N
WP_231910752.1	SDR family oxidoreductase	N	N	N	N	N	N

	WP_231910796.1	OST-HTH/LOTUS domain-containing protein	N	N	N	N	N	N
	WP_231910802.1	elements of external origin	N	N	N	N	N	N
	WP_231910804.1	phage portal protein	NZ_CP008838.1-1, NZ_CP065965.1-1, NZ_LT906480.1-5	100	NZ_LT906480.1-8	100	N	N
	WP_231910805.1	major capsid protein	NZ_CP008838.1-1, NZ_CP065965.1-1, NZ_LT906480.1-5	100	N	N	N	N
	WP_231910821.1	SDR family oxidoreductase	N	N	N	N	N	N
	WP_231910850.1	type II toxin-antitoxin system RelE/ParE family toxin	N	N	N	N	N	N
	WP_231910863.1	NgoMIV family type II restriction endonuclease	N	N	N	N	N	N
	WP_231910988.1	substrate binding domain-containing protein	N	N	N	N	N	N
	WP_232622053.1	AbiEi antitoxin N-terminal domain-containing protein	N	N	N	N	N	N
	WP_235219166.1	site-specific DNA-methyltransferase	N	N	N	N	N	N
NZ_CP011010.1	WP_005408332.1	GPW/gp25 family protein	N	N	N	N	N	N
	WP_005416648.1	RebB family R body protein	N	N	N	N	N	N

WP_005416649.1	RebB family R body protein	N	N	N	N	N	N
WP_005416651.1	RebB family R body protein	N	N	N	N	N	N
WP_006446865.1	RebB family R body protein	N	N	N	N	N	N
WP_006474871.1	cytochrome o ubiquinol oxidase subunit IV	N	N	N	N	N	N
WP_006479484.1	hypothetical protein	N	N	N	N	N	N
WP_008264806.1	hypothetical protein	N	N	N	N	N	N
WP_008267712.1	head-tail connector protein	N	N	N	N	N	N
WP_012480190.1	mercury resistance system periplasmic binding protein MerP	N	N	N	N	N	N
WP_019186249.1	metalloregulator ArsR/SmtB family transcription factor	N	N	N	N	N	N
WP_021202108.1	metalloregulator ArsR/SmtB family transcription factor	N	N	N	N	N	N
WP_026070259.1	NAD(P)/FAD-dependent oxidoreductase	N	N	N	N	N	N
WP_040006894.1	efflux RND transporter periplasmic adaptor subunit	N	N	N	N	N	N
WP_049453421.1	UvrD-helicase domain-containing protein	N	N	N	N	N	N

WP_049453423.1	ATP-binding protein	N	N	N	N	N	N
WP_049457237.1	DUF1097 domain-containing protein	N	N	N	N	N	N
WP_053448471.1	TetR/AcrR family transcriptional regulator	N	N	N	N	N	N
WP_053448703.1	ABC transporter ATP-binding protein	N	N	N	N	N	N
WP_053448706.1	DegT/DnrJ/EryC1/StrS family aminotransferase	N	N	N	N	N	N
WP_053448707.1	glycosyltransferase family 4 protein	N	N	N	N	N	N
WP_053448716.1	glycosyltransferase family 2 protein	N	N	N	N	N	N
WP_053448729.1	NAD(P)/FAD-dependent oxidoreductase	N	N	N	N	N	N
WP_053448867.1	transcriptional regulator	N	N	N	N	N	N
WP_053449080.1	DUF2306 domain-containing protein	N	N	N	N	N	N
WP_053449082.1	LytTR family DNA-binding domain-containing protein	N	N	N	N	N	N
WP_053449309.1	Rrf2 family transcriptional regulator	N	N	N	N	N	N
WP_053449431.1	hypothetical protein	NZ_CP011010.1-3	100	N	N	N	N

WP_053449452.1	glycoside hydrolase family 19 protein	N	N	N	N	N	N
WP_053449456.1	phage terminase large subunit	NZ_CP011010.1-3	100	N	N	N	N
WP_053449457.1	DUF1073 domain-containing protein	NZ_CP011010.1-3	100	N	N	N	N
WP_053449460.1	DUF2184 domain-containing protein	NZ_CP011010.1-3	100	N	N	N	N
WP_053449462.1	DnaT-like ssDNA-binding protein	N	N	N	N	N	N
WP_053449529.1	DUF1837 domain-containing protein	N	N	N	N	N	N
WP_053449530.1	DEAD/DEAH box helicase	N	N	N	N	N	N
WP_053449659.1	PLP-dependent aminotransferase family protein	N	N	N	N	N	N
WP_053449747.1	DUF305 domain-containing protein	N	N	N	N	N	N
WP_053449750.1	PstS family phosphate ABC transporter substrate-binding protein	N	N	N	N	N	N
WP_053449782.1	alpha/beta hydrolase	N	N	N	N	N	N
WP_053449784.1	PepSY-associated TM helix domain-containing protein	N	N	N	N	N	N
WP_053449792.1	MFS transporter	N	N	N	N	N	N

WP_053450090.1	phage major capsid protein	NZ_CP011010.1-4	100	N	N	N	N
WP_053450091.1	HK97 family phage prohead protease	NZ_CP011010.1-4	100	N	N	N	N
WP_053450092.1	phage portal protein	NZ_CP011010.1-4	100	N	N	N	N
WP_053450553.1	hypothetical protein	N	N	N	N	N	N
WP_053450937.1	TonB-dependent receptor	N	N	N	N	N	N
WP_053450938.1	alpha-D-ribose 1-methylphosphonate 5-triphosphate diphosphatase	N	N	N	N	N	N
WP_053450940.1	TIGR03364 family FAD-dependent oxidoreductase	N	N	N	N	N	N
WP_053450941.1	DUF5690 family protein	N	N	N	N	N	N
WP_053450942.1	HAD-IA family hydrolase	N	N	N	N	N	N
WP_053451234.1	DegT/DnrJ/EryC1/StrS family aminotransferase	N	N	N	N	N	N
WP_074038356.1	YegP family protein	N	N	N	N	N	N
WP_080374898.1	DCL family protein	N	N	N	N	N	N
WP_080374914.1	phosphate ABC transporter permease PstA	N	N	N	N	N	N

WP_080374983.1	SIR2 family protein	N	N	N	N	N	N
WP_080374987.1	DUF3304 domain-containing protein	N	N	N	N	N	N
WP_086009122.1	IS3-like element ISPa39 family transposase	N	N	N	N	N	N
WP_148565003.1	hypothetical protein	N	N	N	N	N	N
WP_187299795.1	hypothetical protein	N	N	N	N	N	N
WP_187299806.1	phosphate ABC transporter permease subunit PstC	N	N	N	N	N	N
WP_197652506.1	TonB-dependent receptor	N	N	N	N	N	N
WP_202967038.1	McrC family protein	N	N	N	N	N	N
WP_219627573.1	lysozyme	N	N	N	N	N	N
WP_226053492.1	SDR family oxidoreductase	N	N	N	N	N	N
WP_238581791.1	helix-turn-helix transcriptional regulator	N	N	N	N	N	N
WP_238581811.1	terminase TerL endonuclease subunit	NZ_CP011010.1-4	100	N	N	N	N
WP_238581852.1	phage terminase large subunit	NZ_CP011010.1-4	100	N	N	N	N

NZ_CP011305.1	WP_238581858.1	histidine kinase	N	N	N	N	N	N
	WP_004152082.1	carboxymuconolactone decarboxylase family protein	N	N	N	N	N	N
	WP_005408320.1	tautomerase family protein	N	N	N	N	N	N
	WP_005409501.1	transcriptional regulator BetI	N	N	N	N	N	N
	WP_005409726.1	ABC transporter permease	N	N	N	N	N	N
	WP_005409874.1	biopolymer transporter ExbD	N	N	N	N	N	N
	WP_005413654.1	Lrp/AsnC family transcriptional regulator	N	N	N	N	N	N
	WP_005416613.1	ABC transporter ATP-binding protein	N	N	N	N	N	N
	WP_006402206.1	acyl carrier protein	N	N	N	N	N	N
	WP_010481131.1	response regulator transcription factor	N	N	N	N	N	N
	WP_010481265.1	VOC family protein	N	N	N	N	N	N
	WP_010481875.1	LysR family transcriptional regulator	N	N	N	N	N	N
	WP_010482114.1	ubiquinol oxidase subunit II	N	N	N	N	N	N

WP_010482305.1	hypothetical protein	N	N	N	N	N	N
WP_010482807.1	VOC family protein	N	N	N	N	N	N
WP_010483185.1	DMT family transporter	N	N	N	N	N	N
WP_010483441.1	alpha/beta hydrolase	N	N	N	N	N	N
WP_010483459.1	helix-turn-helix transcriptional regulator	N	N	N	N	N	N
WP_010483717.1	response regulator transcription factor	N	N	N	N	N	N
WP_010483737.1	glutathione S-transferase family protein	N	N	N	N	N	N
WP_010483779.1	baseplate J/gp47 family protein	N	N	N	N	N	N
WP_010483787.1	phage tail sheath subtilisin-like domain-containing protein	NZ_CP011305.1-1, NZ_CP011306.1-1, NZ_CP060259.1-2, NZ_CP077679.1-1, NZ_CP104169.1-1, NZ_OU943334.1-1	100	N	N	N	N
WP_010484073.1	type II toxin-antitoxin system RelE/ParE family toxin	N	N	N	N	N	N
WP_010484092.1	winged helix-turn-helix domain-containing protein	N	N	N	N	N	N

WP_010484425.1	protein deglycase HchA	N	N	N	N	N	N
WP_010484895.1	helix-turn-helix domain-containing protein	N	N	N	N	N	N
WP_010484993.1	Rid family hydrolase	N	N	N	N	N	N
WP_010485157.1	MerR family transcriptional regulator	N	N	N	N	N	N
WP_010485159.1	MgtC/SapB family protein	N	N	N	N	N	N
WP_010485178.1	sigma-70 family RNA polymerase sigma factor	N	N	N	N	N	N
WP_010485209.1	hypothetical protein	N	N	N	N	N	N
WP_010485313.1	respiratory nitrate reductase subunit gamma	N	N	N	N	N	N
WP_010485323.1	AzlD family protein	N	N	N	N	N	N
WP_010485465.1	DUF3224 domain-containing protein	N	N	N	N	N	N
WP_010485668.1	LysR family transcriptional regulator	N	N	N	N	N	N
WP_010485778.1	response regulator	N	N	N	N	N	N
WP_010486053.1	thioesterase family protein	N	N	N	N	N	N

WP_010486459.1	VOC family protein	N	N	N	N	N	N
WP_010486561.1	alpha/beta hydrolase	N	N	N	N	N	N
WP_010486565.1	TetR/AcrR family transcriptional regulator	N	N	N	N	N	N
WP_010486984.1	type II toxin-antitoxin system RelE/ParE family toxin	N	N	N	N	N	N
WP_012479558.1	EamA family transporter	N	N	N	N	N	N
WP_012480266.1	Ohr family peroxiredoxin	N	N	N	N	N	N
WP_012510145.1	VOC family protein	N	N	N	N	N	N
WP_019336340.1	DoxX family protein	N	N	N	N	N	N
WP_019336516.1	carboxymuconolactone decarboxylase family protein	N	N	N	N	N	N
WP_019337463.1	GNAT family N-acetyltransferase	N	N	N	N	N	N
WP_019337583.1	response regulator	N	N	N	N	N	N
WP_019337672.1	Gfo/Idh/MocA family oxidoreductase	N	N	N	N	N	N
WP_019337810.1	NarK family nitrate/nitrite MFS transporter	N	N	N	N	N	N

WP_019338001.1	class I SAM-dependent methyltransferase	N	N	N	N	N	N
WP_019338179.1	response regulator transcription factor	N	N	N	N	N	N
WP_019338357.1	alpha/beta hydrolase	N	N	N	N	N	N
WP_019338484.1	GNAT family N-acetyltransferase	N	N	N	N	N	N
WP_019338592.1	PDDEXK nuclease domain-containing protein	N	N	N	N	N	N
WP_019338970.1	DNA-binding transcriptional regulator	N	N	N	N	N	N
WP_019660267.1	DUF2239 family protein	N	N	N	N	N	N
WP_019660556.1	metalloregulator ArsR/SmtB family transcription factor	N	N	N	N	N	N
WP_020424480.1	response regulator	N	N	N	N	N	N
WP_021203687.1	HigA family addiction module antitoxin	N	N	N	N	N	N
WP_029379823.1	BCCT family transporter	N	N	N	N	N	N
WP_029380003.1	biotin/lipoyl-binding protein	N	N	N	N	N	N
WP_029550852.1	low molecular weight protein-tyrosine-phosphatase	N	N	N	N	N	N

WP_043396245.1	type II secretion system major pseudopilin GspG	N	N	N	N	N	N
WP_043397241.1	Cu(I)-responsive transcriptional regulator	N	N	N	N	N	N
WP_043397376.1	hypothetical protein	N	N	N	N	N	N
WP_043400330.1	formate dehydrogenase subunit gamma	N	N	N	N	N	N
WP_043400508.1	response regulator transcription factor	N	N	N	N	N	N
WP_043400702.1	VOC family protein	N	N	N	N	N	N
WP_043400854.1	SDR family oxidoreductase	N	N	N	N	N	N
WP_043401898.1	SMR family transporter	N	N	N	N	N	N
WP_043402402.1	alpha/beta hydrolase	N	N	N	N	N	N
WP_049409128.1	methylated-DNA--[protein]-cysteine S-methyltransferase	N	N	N	N	N	N
WP_049409424.1	helix-turn-helix transcriptional regulator	N	N	N	N	N	N
WP_049410196.1	glycoside hydrolase family 104 protein	N	N	N	N	N	N
WP_049411723.1	hypothetical protein	N	N	N	N	N	N

WP_049413021.1	NADH:flavin oxidoreductase/NADH oxidase family protein	N	N	N	N	N	N
WP_049437620.1	response regulator transcription factor	N	N	N	N	N	N
WP_049455238.1	MBL fold metallo-hydrolase	N	N	N	N	N	N
WP_049455240.1	cupin domain-containing protein	N	N	N	N	N	N
WP_049455305.1	ABC transporter ATP-binding protein	N	N	N	N	N	N
WP_049455405.1	MerR family transcriptional regulator	N	N	N	N	N	N
WP_049480012.1	putative addiction module antidote protein	N	N	N	N	N	N
WP_049480539.1	LysR family transcriptional regulator	N	N	N	N	N	N
WP_049481144.1	ATP-binding cassette domain-containing protein	N	N	N	N	N	N
WP_049482249.1	sugar phosphate isomerase/epimerase family protein	N	N	N	N	N	N
WP_049482965.1	ATP-binding protein	N	N	N	N	N	N
WP_053442379.1	putative addiction module antidote protein	N	N	N	N	N	N
WP_053442503.1	UDP-N-acetylglucosamine 2-epimerase (non-hydrolyzing)	N	N	N	N	N	N

WP_053442597.1	LysR substrate-binding domain-containing protein	N	N	N	N	N	N
WP_053442610.1	GNAT family N-acetyltransferase	N	N	N	N	N	N
WP_053442646.1	LysR family transcriptional regulator	N	N	N	N	N	N
WP_053442679.1	GyrI-like domain-containing protein	N	N	N	N	N	N
WP_053442687.1	cellulase family glycosylhydrolase	N	N	N	N	N	N
WP_053442702.1	aromatic alcohol reductase	N	N	N	N	N	N
WP_053442724.1	aminotransferase class III-fold pyridoxal phosphate-dependent enzyme	N	N	N	N	N	N
WP_053442807.1	TonB-dependent receptor	N	N	N	N	N	N
WP_053442835.1	SRPBCC family protein	N	N	N	N	N	N
WP_053442850.1	GNAT family N-acetyltransferase	N	N	N	N	N	N
WP_053442875.1	hypothetical protein	N	N	N	N	N	N
WP_053442895.1	SDR family oxidoreductase	N	N	N	N	N	N
WP_053443022.1	aldehyde oxidoreductase molybdenum-binding subunit PaoC	N	N	N	N	NZ_CP011305.1-2, NZ_CP011306.1-2	

WP_053443025.1	SDR family oxidoreductase	N	N	N	N	N	N
WP_053443026.1	LysR family transcriptional regulator	N	N	N	N	N	N
WP_053443027.1	carboxymuconolactone decarboxylase family protein	N	N	N	N	N	N
WP_053443028.1	RNA polymerase sigma-70 factor	N	N	N	N	N	N
WP_053443087.1	hypothetical protein	N	N	N	N	N	N
WP_053443100.1	response regulator	N	N	N	N	N	N
WP_053443101.1	ATP-binding protein	N	N	N	N	N	N
WP_053443102.1	ABC transporter six-transmembrane domain-containing protein	N	N	N	N	N	N
WP_053443105.1	TonB-dependent siderophore receptor	N	N	N	N	N	N
WP_053443125.1	heavy metal response regulator transcription factor	N	N	N	N	N	N
WP_053443132.1	response regulator transcription factor	N	N	N	N	N	N
WP_053443160.1	nitrate/nitrite transporter	N	N	N	N	N	N
WP_053443171.1	diacylglycerol kinase	N	N	N	N	N	N

WP_053443191.1	VOC family protein	N	N	N	N	N	N
WP_053443250.1	FAD-dependent monooxygenase	N	N	N	N	N	N
WP_053443264.1	agmatine deiminase family protein	N	N	N	N	N	N
WP_053443265.1	LysR substrate-binding domain-containing protein	N	N	N	N	N	N
WP_053443465.1	L-seryl-tRNA(Sec) selenium transferase	N	N	N	N	N	N
WP_053443466.1	selenide, water dikinase SelD	N	N	N	N	N	N
WP_053443564.1	MFS transporter	N	N	N	N	N	N
WP_053443567.1	CmlA/FloR family chloramphenicol efflux MFS transporter	N	N	N	N	N	N
WP_053443676.1	L-dopachrome tautomerase-related protein	N	N	N	N	N	N
WP_053443739.1	DNA polymerase III subunit epsilon	N	N	N	N	N	N
WP_053443840.1	selenocysteine-specific translation elongation factor	N	N	N	N	N	N
WP_080112492.1	helix-turn-helix domain-containing protein	N	N	N	N	N	N
WP_100050534.1	ATP-binding protein	N	N	N	N	N	N

	WP_100050587.1	cytochrome o ubiquinol oxidase subunit III	N	N	N	N	N	N
	WP_227200592.1	LysR family transcriptional regulator	N	N	N	N	N	N
	WP_238582434.1	HipA N-terminal domain-containing protein	N	N	N	N	N	N
	WP_238582441.1	MFS transporter	N	N	N	N	N	N
	WP_238582468.1	dihydrodipicolinate synthase family protein	N	N	N	N	N	N
	WP_238582477.1	nucleoside triphosphatase NudI	N	N	N	N	N	N
	WP_265349072.1	recombinase family protein	N	N	N	N	N	N
	WP_265349073.1	NAD-binding protein	N	N	N	N	N	N
	WP_005408332.1	GPW/gp25 family protein	N	N	N	N	N	N
	WP_005411473.1	cytochrome o ubiquinol oxidase subunit IV	N	N	N	N	N	N
NZ_CP014014.1	WP_024957933.1	metalloregulator ArsR/SmtB family transcription factor	N	N	N	N	N	N
	WP_024957960.1	VOC family protein	N	N	N	N	N	N
	WP_024957961.1	Na+/H+ antiporter NhaA	N	N	N	N	N	N

WP_032961836.1	phage terminase large subunit	NZ_CM001824.1-1, NZ_CP014014.1-2, NZ_LR134324.1-1	100	NZ_AP021908.1-1, NZ_CP040431.1-2, NZ_CP040439.1-4, NZ_CP043578.1-1, NZ_CP056088.1-2, NZ_CP060027.1-3, NZ_LR134324.1-3, NZ_CP090418.1-2, NZ_CP104863.1-3, NZ_CP102942.1-3, NZ_CP098483.1-2	100	NZ_CP040430.1-2	100
WP_033833415.1	3'-5' exonuclease	N	N	N	N	N	N
WP_033834636.1	restriction endonuclease	N	N	N	N	N	N
WP_033835782.1	hypothetical protein	N	N	N	N	N	N
WP_033835790.1	GNAT family N-acetyltransferase	N	N	N	N	N	N
WP_049397550.1	ABC transporter ATP-binding protein	N	N	N	N	N	N
WP_051187993.1	BPL-N domain-containing protein	N	N	NZ_CP014014.1-7	100	N	N
WP_065426719.1	hypothetical protein	N	N	N	N	N	N
WP_080240930.	ABC transporter permease	N	N	N	N	N	N

	1							
	WP_104920609.1	terminase TerL endonuclease subunit	N	N	N	N	N	N
	WP_104920636.1	zincin-like metallopeptidase domain-containing protein	N	N	N	N	N	N
	WP_104920701.1	YqaJ viral recombinase family protein	N	N	N	N	N	N
	WP_104920739.1	lysozyme	N	N	N	N	N	N
	WP_104920759.1	class I SAM-dependent DNA methyltransferase	N	N	N	N	N	N
	WP_168356115.1	HupE/UreJ family protein	N	N	N	N	N	N
	WP_197628585.1	hypothetical protein	N	N	N	N	N	N
	WP_223224751.1	substrate binding domain-containing protein	N	N	N	N	N	N
	WP_223224752.1	DUF3422 family protein	N	N	N	N	N	N
	WP_223224753.1	SDR family oxidoreductase	N	N	N	N	N	N
	WP_049399935.1	helix-turn-helix transcriptional regulator	N	N	N	N	N	N
NZ_CP015612.1	WP_049444104.1	GPW/gp25 family protein	N	N	N	N	N	N
	WP_049452914.	hypothetical protein	N	N	N	N	N	N

1							
WP_049452916.1	Dyp-type peroxidase	N	N	N	N	N	N
WP_070470375.1	catalase family protein	N	N	N	N	N	N
WP_088024715.1	SNF2-related protein	N	N	N	N	N	N
WP_088024717.1	DNA methyltransferase	N	N	N	N	N	N
WP_088024719.1	DEAD/DEAH box helicase family protein	N	N	N	N	N	N
WP_088024721.1	DUF262 domain-containing protein	N	N	N	N	N	N
WP_088025023.1	LysR family transcriptional regulator	N	N	N	N	N	N
WP_088025239.1	ImmA/IrrE family metallo-endopeptidase	N	N	N	N	N	N
WP_088025245.1	hypothetical protein	N	N	N	N	N	N
WP_088026027.1	helix-turn-helix transcriptional regulator	N	N	N	N	N	N
WP_088026103.1	outer membrane beta-barrel protein	N	N	N	N	N	N
WP_088026115.1	alpha/beta fold hydrolase	N	N	N	N	N	N
WP_088026415.	head-tail connector protein	N	N	N	N	N	N

	1						
	WP_088026416.1	phage major capsid protein	NZ_CP015612.1-2	100	N	N	N	N
	WP_088026417.1	HK97 family phage prohead protease	NZ_CP015612.1-2	100	N	N	N	N
	WP_088026418.1	phage portal protein	NZ_CP015612.1-2	100	N	N	N	N
	WP_088027319.1	M24 family metallopeptidase	N	N	N	N	N	N
	WP_088027681.1	hypothetical protein	N	N	N	N	N	N
	WP_198360839.1	hypothetical protein	N	N	N	N	N	N
	WP_232460611.1	isocitrate lyase/PEP mutase family protein	N	N	N	N	N	N
NZ_CP018756.1	WP_002808376.1	30S ribosomal protein S21	N	N	N	N	N	N
	WP_005409596.1	translation initiation factor IF-1	N	N	N	N	N	N
	WP_010341589.1	30S ribosomal protein S10	N	N	N	N	N	N
	WP_079220429.1	dihydrolipoyl dehydrogenase	N	N	N	N	N	N
	WP_079220531.1	NAD(P)-dependent oxidoreductase	N	N	N	N	N	N
	WP_079220990.	SDR family oxidoreductase	N	N	N	N	N	N

1							
WP_079221084.1	3-methyl-2-oxobutanoate hydroxymethyltransferase	N	N	N	N	N	N
WP_079221085.1	Lrp/AsnC family transcriptional regulator	N	N	N	N	N	N
WP_079221099.1	recombinase family protein	N	N	N	N	N	N
WP_079221148.1	DUF308 domain-containing protein	N	N	N	N	N	N
WP_079221149.1	HD domain-containing protein	N	N	N	N	N	N
WP_079221154.1	SDR family oxidoreductase	N	N	N	N	N	N
WP_079221155.1	galactonate dehydratase	N	N	N	N	N	N
WP_079221159.1	cellulase family glycosylhydrolase	N	N	N	N	N	N
WP_079221400.1	SRPBCC family protein	N	N	N	N	N	N
WP_079221452.1	MFS family transporter	N	N	N	N	N	N
WP_079221638.1	AraC family transcriptional regulator	N	N	N	N	N	N
WP_079221639.1	SDR family oxidoreductase	N	N	N	N	N	N
WP_079221640.	VOC family protein	N	N	N	N	N	N

1							
WP_079221894.1	glucose 1-dehydrogenase	N	N	N	N	N	N
WP_079221929.1	multidrug efflux RND transporter permease subunit	N	N	N	N	N	N
WP_079221931.1	arginine deiminase	N	N	N	N	N	N
WP_079221935.1	basic amino acid/polyamine antiporter	N	N	N	N	N	N
WP_079221936.1	basic amino acid/polyamine antiporter	N	N	N	N	N	N
WP_079221962.1	cysteine hydrolase family protein	N	N	N	N	N	N
WP_079221973.1	SDR family oxidoreductase	N	N	N	N	N	N
WP_079221974.1	helix-turn-helix transcriptional regulator	N	N	N	N	N	N
WP_079222053.1	ketosteroid isomerase-related protein	N	N	N	N	N	N
WP_079222100.1	DUF1330 domain-containing protein	N	N	N	N	N	N
WP_079222103.1	alpha/beta fold hydrolase	N	N	N	N	N	N
WP_079222107.1	cytosine permease	N	N	N	N	N	N
WP_079222108.	NAD-dependent succinate-semialdehyde	N	N	N	N	N	N

1	dehydrogenase						
WP_079222109.1	4-carboxymuconolactone decarboxylase	N	N	N	N	N	N
WP_079222224.1	type VI secretion system contractile sheath small subunit	N	N	N	N	N	N
WP_079222431.1	glutathione transferase GstA	N	N	N	N	N	N
WP_079222584.1	helix-turn-helix domain-containing protein	N	N	N	N	N	N
WP_079222779.1	MerR family transcriptional regulator	N	N	N	N	N	N
WP_079223058.1	type II toxin-antitoxin system RelE/ParE family toxin	N	N	N	N	N	N
WP_079223060.1	NadS family protein	N	N	N	N	N	N
WP_079223610.1	SDR family NAD(P)-dependent oxidoreductase	N	N	N	N	N	N
WP_079224755.1	N-6 DNA methylase	N	N	N	N	N	N
WP_079224760.1	aldehyde reductase	N	N	N	N	N	N
WP_079225202.1	carboxymuconolactone decarboxylase family protein	N	N	N	N	N	N
WP_079225223.1	LysR family transcriptional regulator	N	N	N	N	N	N
WP_192851819.	transcriptional regulator	N	N	N	N	N	N

	1							
	WP_192851837.1	MFS transporter	N	N	N	N	N	N
	WP_202818502.1	EcsC family protein	N	N	N	N	N	N
	WP_202818505.1	AraC family transcriptional regulator	N	N	N	N	N	N
	WP_237771956.1	LysR family transcriptional regulator	N	N	N	N	N	N
	WP_237772019.1	2-dehydro-3-deoxy-6-phosphogalactonate aldolase	N	N	N	N	N	N
	WP_237772020.1	aldose epimerase family protein	N	N	N	N	N	N
	WP_237772081.1	L-histidine N(alpha)-methyltransferase	N	N	N	N	N	N
	WP_237772092.1	histidine kinase famiy protein	N	N	N	N	N	N
	WP_265349656.1	DUF4291 domain-containing protein	N	N	N	N	N	N
NZ_CP022053.2	WP_005408332.1	GPW/gp25 family protein	N	N	N	N	N	N
	WP_021202108.1	metalloregulator ArsR/SmtB family transcription factor	N	N	N	N	N	N
	WP_024957611.1	hypothetical protein	N	N	N	N	N	N
	WP_032966544.	nucleotide pyrophosphohydrolase	N	N	N	N	N	N

1							
WP_049450114.1	DUF1819 family protein	N	N	N	N	N	N
WP_049453423.1	ATP-binding protein	N	N	N	N	N	N
WP_049459799.1	group II intron reverse transcriptase/maturase	N	N	N	N	N	N
WP_049460427.1	DUF3944 domain-containing protein	N	N	N	N	N	N
WP_049460432.1	7-cyano-7-deazaguanine synthase QueC	N	N	N	N	N	N
WP_049460436.1	tRNA-guanine transglycosylase DpdA	N	N	N	N	N	N
WP_049460451.1	tyrosine-type recombinase/integrase	N	N	N	N	N	N
WP_049461407.1	UvrD-helicase domain-containing protein	N	N	N	N	N	N
WP_080356717.1	7-carboxy-7-deazaguanine synthase	N	N	NZ_CP088241.1-4	100	N	N
WP_088610764.1	CoA transferase	N	N	N	N	N	N
WP_088610766.1	3-hydroxyacyl-CoA dehydrogenase NAD-binding domain-containing protein	N	N	N	N	N	N
WP_088610844.1	hypothetical protein	N	N	N	N	N	N
WP_088610919.	MBL fold metallo-hydrolase	N	N	N	N	N	N

1							
WP_088610956.1	BREX system Lon protease-like protein BrxL	N	N	N	N	N	N
WP_088610957.1	PglZ domain-containing protein	N	N	N	N	N	N
WP_088610959.1	SAM-dependent methyltransferase	N	N	N	N	N	N
WP_088610960.1	AIPR family protein	N	N	N	N	N	N
WP_088610961.1	BREX system P-loop protein BrxC	N	N	N	N	N	N
WP_088610962.1	BREX protein BrxB domain-containing protein	N	N	N	N	N	N
WP_088611017.1	ABC transporter ATP-binding protein	N	N	N	N	N	N
WP_088611018.1	ABC transporter permease	N	N	N	N	N	N
WP_088611150.1	hypothetical protein	N	N	N	N	N	N
WP_088611177.1	hypothetical protein	N	N	N	N	N	N
WP_088611178.1	hypothetical protein	N	N	N	N	N	N
WP_088611206.1	terminase	N	N	N	N	N	N
WP_088611259.	enoyl-CoA hydratase-related protein	N	N	N	N	N	N

	1							
	WP_105609235.1	AraC family transcriptional regulator	N	N	N	N	N	N
	WP_105609236.1	acyl-CoA dehydrogenase	N	N	N	N	N	N
	WP_105609246.1	AAA family ATPase	N	N	N	N	N	N
	WP_225792027.1	hypothetical protein	N	N	N	N	N	N
	WP_225792080.1	DUF4423 domain-containing protein	N	N	N	N	N	N
	WP_225792104.1	type II restriction endonuclease subunit M	N	N	N	N	N	N
	WP_225792105.1	BrxE family protein	N	N	N	N	N	N
	WP_239684663.1	substrate binding domain-containing protein	N	N	N	N	N	N
	WP_000995360.1	mercury resistance co-regulator MerD	N	N	N	N	N	N
	WP_003131974.1	mercuric ion transporter MerT	N	N	N	N	N	N
NZ_CP025298.1	WP_003131987.1	mercury resistance system periplasmic binding protein MerP	N	N	N	N	N	N
	WP_003156770.1	mercury(II) reductase	N	N	N	N	N	N
	WP_005413387.	Hg(II)-responsive transcriptional	N	N	N	N	N	N

1	regulator						
WP_005413400.1	Tn3-like element TnAs1 family transposase	N	N	N	N	NZ_CP043578.1-2	100
WP_005414983.1	ATP-binding protein	N	N	N	N	N	N
WP_005414984.1	MFS transporter	N	N	N	N	N	N
WP_005416648.1	RebB family R body protein	N	N	N	N	N	N
WP_005416649.1	RebB family R body protein	N	N	N	N	N	N
WP_005416651.1	RebB family R body protein	N	N	N	N	N	N
WP_006399830.1	cytochrome o ubiquinol oxidase subunit IV	N	N	N	N	N	N
WP_006446865.1	RebB family R body protein	N	N	N	N	N	N
WP_009459572.1	integrase arm-type DNA-binding domain-containing protein	N	N	N	N	N	N
WP_009459580.1	Dyp-type peroxidase	N	N	N	N	N	N
WP_009459591.1	helix-turn-helix transcriptional regulator	N	N	N	N	N	N
WP_009459601.1	DUF488 domain-containing protein	N	N	N	N	N	N
WP_009459603.	excinuclease ABC subunit UvrB	N	N	N	N	N	N

1							
WP_009459624.1	VOC family protein	N	N	N	N	N	N
WP_009459628.1	replication-associated recombination protein A	N	N	N	N	N	N
WP_009459645.1	hypothetical protein	N	N	N	N	N	N
WP_009459646.1	integrating conjugative element protein	N	N	N	N	N	N
WP_009459649.1	TIGR03757 family integrating conjugative element protein	N	N	N	N	N	N
WP_009459650.1	DNA repair protein RadC	N	N	N	N	N	N
WP_009459652.1	DsbA family protein	N	N	N	N	N	N
WP_009459662.1	TIGR03751 family conjugal transfer lipoprotein	N	N	N	N	N	N
WP_009459664.1	TIGR03752 family integrating conjugative element protein	N	N	N	N	N	N
WP_009459665.1	TIGR03749 family integrating conjugative element protein	N	N	N	N	N	N
WP_009459666.1	TIGR03746 family integrating conjugative element protein	N	N	N	N	N	N
WP_009459668.1	TIGR03750 family conjugal transfer protein	N	N	N	N	N	N
WP_009459670.	TIGR03745 family integrating	N	N	N	N	N	N

1	conjugative element membrane protein						
WP_009459672.1	TIGR03758 family integrating conjugative element protein	N	N	N	N	N	N
WP_009459676.1	TIGR03747 family integrating conjugative element membrane protein	N	N	N	N	N	N
WP_009459678.1	integrating conjugative element protein	N	N	N	N	N	N
WP_009459679.1	transglycosylase SLT domain-containing protein	N	N	N	N	N	N
WP_009459680.1	TIGR03759 family integrating conjugative element protein	N	N	N	N	N	N
WP_009459682.1	PilL N-terminal domain-containing protein	N	N	N	N	N	N
WP_009459693.1	hypothetical protein	N	N	N	N	N	N
WP_009459697.1	DUF3275 family protein	N	N	N	N	N	N
WP_009459698.1	DUF3577 domain-containing protein	N	N	N	N	N	N
WP_009459699.1	DUF3085 domain-containing protein	N	N	N	N	N	N
WP_009459707.1	hypothetical protein	N	N	N	N	N	N
WP_009459709.1	hypothetical protein	N	N	N	N	N	N
WP_009459717.	DNA topoisomerase III	N	N	N	N	N	N

1							
WP_009459718.1	DUF3158 family protein	N	N	N	N	N	N
WP_009459719.1	TIGR03761 family integrating conjugative element protein	N	N	N	N	N	N
WP_009459721.1	STY4528 family pathogenicity island replication protein	N	N	N	N	N	N
WP_009459723.1	DUF2857 domain-containing protein	N	N	N	N	N	N
WP_009459725.1	ParB family protein	N	N	N	N	N	N
WP_009459727.1	ParA family protein	N	N	N	N	N	N
WP_009459729.1	AlpA family transcriptional regulator	N	N	N	N	N	N
WP_010921730.1	recombinase family protein	N	N	N	N	N	N
WP_012478642.1	UvrD-helicase domain-containing protein	N	N	N	N	N	N
WP_019726332.1	hypothetical protein	N	N	N	N	N	N
WP_019726333.1	hypothetical protein	N	N	N	N	N	N
WP_019726334.1	single-stranded DNA-binding protein	N	N	N	N	N	N
WP_019726335.	hypothetical protein	N	N	N	N	N	N

1							
WP_019726336.1	hypothetical protein	N	N	N	N	N	N
WP_019726337.1	helicase-related protein	N	N	N	N	N	N
WP_019726341.1	conjugative transfer ATPase	N	N	N	N	N	N
WP_019726345.1	conjugal transfer protein TraG N-terminal domain-containing protein	N	N	N	N	N	N
WP_019726349.1	MobH family relaxase	N	N	N	N	N	N
WP_019726351.1	Na+/H+ antiporter NhaA	N	N	N	N	N	N
WP_019726352.1	NAD(P)/FAD-dependent oxidoreductase	N	N	N	N	N	N
WP_019726353.1	DUF3422 family protein	N	N	N	N	N	N
WP_019726376.1	LysR family transcriptional regulator	N	N	N	N	N	N
WP_024957961.1	Na+/H+ antiporter NhaA	N	N	N	N	N	N
WP_024957965.1	antibiotic biosynthesis monooxygenase	N	N	N	N	N	N
WP_049443984.1	alcohol dehydrogenase catalytic domain-containing protein	N	N	N	N	N	N
WP_049443994.	SDR family oxidoreductase	N	N	N	N	N	N

1							
WP_049445372.1	HigA family addiction module antitoxin	N	N	N	N	N	N
WP_049460453.1	helix-turn-helix domain-containing protein	N	N	N	N	N	N
WP_072167338.1	arsenate reductase ArsC	N	N	N	N	N	N
WP_080347515.1	helix-turn-helix domain-containing protein	N	N	N	N	N	N
WP_080347532.1	LysR family transcriptional regulator	N	N	N	N	N	N
WP_088480608.1	alkene reductase	N	N	N	N	N	N
WP_093999576.1	type II toxin-antitoxin system RelE/ParE family toxin	N	N	N	N	N	N
WP_101764902.1	SDR family oxidoreductase	N	N	N	N	N	N
WP_101765089.1	alpha/beta hydrolase	N	N	N	N	N	N
WP_101765242.1	NAD(P)H-dependent oxidoreductase	N	N	N	N	N	N
WP_101765322.1	type ISP restriction/modification enzyme	N	N	N	N	N	N
WP_101765396.1	alpha/beta fold hydrolase	N	N	N	N	N	N
WP_101765461.	phosphoadenosine phosphosulfate	N	N	N	N	N	N

1	reductase family protein						
WP_101765776.1	glutathione transferase GstA	N	N	N	N	N	N
WP_101766100.1	MepB family protein	N	N	N	N	N	N
WP_101766217.1	hypothetical protein	N	N	N	N	N	N
WP_101766226.1	VOC family protein	N	N	N	N	N	N
WP_101766454.1	sulfite exporter TauE/SafE family protein	N	N	N	N	N	N
WP_219805742.1	DUF6094 domain-containing protein	N	N	N	N	N	N
WP_223224753.1	SDR family oxidoreductase	N	N	N	N	N	N
WP_223224789.1	SRPBCC domain-containing protein	N	N	N	N	N	N
WP_223656181.1	glutathione-independent formaldehyde dehydrogenase	N	N	N	N	N	N
WP_233490792.1	LysR family transcriptional regulator	N	N	N	N	N	N
WP_233490804.1	MFS transporter	N	N	N	N	N	N
WP_233490805.1	transcriptional regulator GcvA	N	N	N	N	N	N
WP_233490806.	DUF3422 family protein	N	N	N	N	N	N

	1							
	WP_233490826.1	DinB family protein	N	N	N	N	N	N
	WP_003131974.1	mercuric ion transporter MerT	N	N	N	N	N	N
	WP_003131987.1	mercury resistance system periplasmic binding protein MerP	N	N	N	N	N	N
	WP_003156770.1	mercury(II) reductase	N	N	N	N	N	N
	WP_003462917.1	VirB3 family type IV secretion system protein	N	N	N	N	N	N
	WP_003830788.1	DDE-type integrase/transposase/recombinase	N	N	N	N	N	N
NZ_CP027562.1	WP_003830789.1	TniB family NTP-binding protein	N	N	N	N	N	N
	WP_004350798.1	P-type conjugative transfer protein TrbL	N	N	N	N	N	N
	WP_004350802.1	P-type conjugative transfer protein TrbJ	N	N	N	N	N	N
	WP_004350822.1	LysR substrate-binding domain-containing protein	N	N	N	N	N	N
	WP_005408332.1	GPW/gp25 family protein	N	N	N	N	N	N
	WP_005413341.1	replication-associated recombination protein A	N	N	N	N	N	N
	WP_005413386.	YeeE/YedE family protein	N	N	N	N	N	N

1							
WP_005413387.1	Hg(II)-responsive transcriptional regulator	N	N	N	N	N	N
WP_005413393.1	mercury(II) reductase	N	N	N	N	N	N
WP_005413394.1	organomercurial transporter MerC	N	N	N	N	N	N
WP_005413396.1	mercury resistance system periplasmic binding protein MerP	N	N	N	N	N	N
WP_005413398.1	Hg(II)-responsive transcriptional regulator	N	N	N	N	N	N
WP_005413400.1	Tn3-like element TnAs1 family transposase	N	N	N	N	NZ_CP043578.1-2	100
WP_005413430.1	cyclopropane fatty acyl phospholipid synthase	NZ_CP051467.1-3	100	N	N	N	N
WP_006399830.1	cytochrome o ubiquinol oxidase subunit IV	N	N	N	N	N	N
WP_006452450.1	helix-turn-helix transcriptional regulator	N	N	N	N	N	N
WP_008264906.1	HupE/UreJ family protein	N	N	N	N	N	N
WP_008294937.1	ribbon-helix-helix protein, CopG family	N	N	N	N	N	N
WP_008786732.1	MFS transporter	N	N	N	N	N	N
WP_008786734.	efflux transporter outer membrane	N	N	N	N	N	N

1	subunit						
WP_008786735.1	efflux RND transporter permease subunit	N	N	N	N	N	N
WP_008786738.1	cation diffusion facilitator family transporter	N	N	N	N	N	N
WP_008786740.1	MFS transporter	N	N	N	N	N	N
WP_008786741.1	metalloregulator ArsR/SmtB family transcription factor	N	N	N	N	N	N
WP_008786745.1	glutaredoxin	N	N	N	N	N	N
WP_008786746.1	Lrp/AsnC ligand binding domain-containing protein	N	N	N	N	N	N
WP_009398074.1	TrbI/VirB10 family protein	N	N	N	N	N	N
WP_009398076.1	P-type conjugative transfer protein TrbG	N	N	N	N	N	N
WP_009586987.1	permease	N	N	N	N	N	N
WP_010921730.1	recombinase family protein	N	N	N	N	N	N
WP_017355006.1	AAA family ATPase	N	N	N	N	N	N
WP_021162752.1	efflux RND transporter periplasmic adaptor subunit	N	N	N	N	N	N
WP_022960598.	conjugal transfer protein TrbF	N	N	N	N	N	N

1							
WP_022960600.1	TrbC/VirB2 family protein	N	N	N	N	N	N
WP_022960607.1	ISL3 family transposase	N	N	N	N	N	N
WP_023047514.1	TetR/AcrR family transcriptional regulator	N	N	N	N	N	N
WP_023081460.1	VirB3 family type IV secretion system protein	N	N	N	N	N	N
WP_023106692.1	DNA repair protein RadC	N	N	N	N	N	N
WP_024718240.1	EexN family lipoprotein	N	N	N	N	N	N
WP_024892197.1	S26 family signal peptidase	N	N	N	N	N	N
WP_026070508.1	NAD(P)/FAD-dependent oxidoreductase	N	N	N	N	N	N
WP_026070509.1	DUF3422 family protein	N	N	N	N	N	N
WP_026070607.1	DUF6602 domain-containing protein	N	N	N	N	N	N
WP_026070609.1	CBASS oligonucleotide cyclase	N	N	N	N	N	N
WP_026346940.1	aromatic alcohol reductase	N	N	N	N	N	N
WP_031623830.	mercuric ion transporter MerT	N	N	N	N	N	N

1							
WP_032962148.1	type II toxin-antitoxin system RelE/ParE family toxin	N	N	N	N	N	N
WP_033835782.1	hypothetical protein	N	N	N	N	N	N
WP_033836038.1	MFS transporter	N	N	N	N	N	N
WP_033938318.1	ribbon-helix-helix protein, CopG family	N	N	N	N	N	N
WP_033939121.1	PDDEXK nuclease domain-containing protein	N	N	N	N	N	N
WP_033939127.1	DNA repair protein RadC	N	N	N	N	N	N
WP_033939129.1	LysE family transporter	N	N	N	N	N	N
WP_033939131.1	LysE family translocator	N	N	N	N	N	N
WP_037590709.1	NAD(P)/FAD-dependent oxidoreductase	N	N	N	N	N	N
WP_043514652.1	site-specific integrase	N	N	N	N	N	N
WP_043514654.1	type II toxin-antitoxin system RelE/ParE family toxin	N	N	N	N	N	N
WP_043514657.1	XRE family transcriptional regulator	N	N	N	N	N	N
WP_043514663.	DUF736 domain-containing protein	N	N	N	N	N	N

1							
WP_043514667.1	ATP-dependent helicase	N	N	N	N	N	N
WP_043514670.1	AAA family ATPase	N	N	N	N	N	N
WP_043514673.1	DUF2285 domain-containing protein	N	N	N	N	N	N
WP_043514675.1	helix-turn-helix domain-containing protein	N	N	N	N	N	N
WP_043514678.1	replication initiator protein A	N	N	N	N	N	N
WP_043514682.1	ParA family partition ATPase	N	N	N	N	N	N
WP_043514684.1	hypothetical protein	N	N	N	N	N	N
WP_043514687.1	DUF2840 domain-containing protein	N	N	N	N	N	N
WP_043514690.1	S26 family signal peptidase	N	N	N	N	N	N
WP_043514729.1	ParB/RepB/Spo0J family partition protein	N	N	N	N	N	N
WP_044316453.1	conjugal transfer protein TrbF	N	N	N	N	N	N
WP_046272807.1	universal stress protein	NZ_CP051467.1-3	100	N	N	N	N
WP_049399935.	helix-turn-helix transcriptional regulator	N	N	N	N	N	N

1							
WP_052150992.1	helix-turn-helix transcriptional regulator	N	N	N	N	N	N
WP_058158353.1	TrbI/VirB10 family protein	N	N	N	N	N	N
WP_058158354.1	P-type conjugative transfer protein TrbG	N	N	N	N	N	N
WP_058158355.1	P-type conjugative transfer protein TrbL	N	N	N	N	N	N
WP_058158357.1	P-type conjugative transfer protein TrbJ	N	N	N	N	N	N
WP_058158359.1	TrbC/VirB2 family protein	N	N	N	N	N	N
WP_071540640.1	caspase family protein	N	N	N	N	N	N
WP_071540641.1	TIR domain-containing protein	N	N	N	N	N	N
WP_080112492.1	helix-turn-helix domain-containing protein	N	N	N	N	N	N
WP_080341470.1	sulfite exporter TauE/SafE family protein	N	N	N	N	N	N
WP_087923870.1	helix-turn-helix domain-containing protein	N	N	N	N	N	N
WP_088025918.1	HigA family addiction module antitoxin	N	N	N	N	N	N
WP_088435694.	AIPR family protein	N	N	N	N	N	N

1							
WP_106466727.1	CusA/CzcA family heavy metal efflux RND transporter	N	N	N	N	N	N
WP_106466886.1	Z1 domain-containing protein	N	N	N	N	N	N
WP_106466985.1	GDP-mannose 4,6-dehydratase	N	N	N	N	N	N
WP_106467224.1	phage tail sheath subtilisin-like domain-containing protein	NZ_CP027562.1-1	100	N	N	N	N
WP_106467617.1	type II toxin-antitoxin system HipA family toxin	N	N	N	N	N	N
WP_106467629.1	NAD(P)H-dependent oxidoreductase	N	N	N	N	N	N
WP_106467630.1	LysR family transcriptional regulator	N	N	N	N	N	N
WP_106467643.1	DNA adenine methylase	N	N	N	N	N	N
WP_106467739.1	type ISP restriction/modification enzyme	N	N	N	N	N	N
WP_106468043.1	flavodoxin	N	N	N	N	N	N
WP_106468047.1	alpha/beta fold hydrolase	N	N	N	N	N	N
WP_106468509.1	EexN family lipoprotein	N	N	N	N	N	N
WP_106468510.	DUF2840 domain-containing protein	N	N	N	N	N	N

1							
WP_106468511.1	ParA family partition ATPase	N	N	N	N	N	N
WP_106468512.1	replication initiator protein A	N	N	N	N	N	N
WP_106468513.1	helix-turn-helix domain-containing protein	N	N	N	N	N	N
WP_106468514.1	DUF2285 domain-containing protein	N	N	N	N	N	N
WP_106468516.1	DUF736 domain-containing protein	N	N	N	N	N	N
WP_106468521.1	ParB/RepB/Spo0J family partition protein	N	N	N	N	N	N
WP_106468522.1	LysR family transcriptional regulator	N	N	N	N	N	N
WP_106468523.1	site-specific integrase	N	N	N	N	N	N
WP_106468708.1	metalloregulator ArsR/SmtB family transcription factor	N	N	N	N	N	N
WP_106469379.1	MBL fold metallo-hydrolase	N	N	N	N	N	N
WP_106469695.1	chromosome partitioning protein ParB	N	N	N	N	N	N
WP_169708473.1	metal-sensing transcriptional repressor	N	N	N	N	N	N
WP_197648563.	MepB family protein	N	N	N	N	N	N

	1						
	WP_199792477.1	DUF2958 domain-containing protein	N	N	N	N	N	N
	WP_226923173.1	TIGR03571 family LLM class oxidoreductase	N	N	N	N	N	N
	WP_234026070.1	AraC family transcriptional regulator	N	N	N	N	N	N
	WP_234026091.1	TIGR03364 family FAD-dependent oxidoreductase	N	N	N	N	N	N
	WP_234026109.1	ATP-binding protein	N	N	N	N	N	N
	WP_234026126.1	isocitrate lyase/PEP mutase family protein	N	N	N	N	N	N
	WP_234026162.1	helicase-related protein	N	N	N	N	N	N
	WP_234026165.1	chromate transporter	N	N	N	N	N	N
	WP_234026176.1	type II toxin-antitoxin system RelE/ParE family toxin	N	N	N	N	N	N
	WP_254919415.1	very short patch repair endonuclease	N	N	N	N	N	N
	WP_265094399.1	recombinase family protein	N	N	N	N	N	N
NZ_CP028358.1	WP_005408332.1	GPW/gp25 family protein	N	N	N	N	N	N
	WP_005411473.	cytochrome o ubiquinol oxidase subunit	N	N	N	N	N	N

1	IV						
WP_019337070.1	nuclear transport factor 2 family protein	N	N	N	N	N	N
WP_021202108.1	metalloregulator ArsR/SmtB family transcription factor	N	N	N	N	N	N
WP_024956864.1	adenylate/guanylate cyclase domain-containing protein	N	N	N	N	N	N
WP_024957611.1	hypothetical protein	N	N	N	N	N	N
WP_046430578.1	sodium:proton exchanger	N	N	N	N	N	N
WP_046430586.1	heavy metal translocating P-type ATPase	N	N	N	N	N	N
WP_046430629.1	MerR family DNA-binding protein	N	N	N	N	N	N
WP_049395165.1	DUF305 domain-containing protein	N	N	N	N	N	N
WP_049410495.1	ABC transporter permease	N	N	N	N	N	N
WP_049429822.1	efflux RND transporter periplasmic adaptor subunit	N	N	N	N	N	N
WP_049429823.1	hypothetical protein	N	N	N	N	N	N
WP_049447908.1	peptidase domain-containing ABC transporter	N	N	N	N	N	N
WP_057503391.	AAA family ATPase	NZ_CP014014.1-2,	100	N	N	N	N

1		NZ_CP028358.1-1					
WP_100442920.1	DNA-processing protein DprA	NZ_CP028358.1-4	100	N	N	N	N
WP_107378969.1	hypothetical protein	N	N	N	N	N	N
WP_107379071.1	ABC transporter ATP-binding protein	N	N	N	N	N	N
WP_107379458.1	DUF4145 domain-containing protein	N	N	N	N	N	N
WP_107379496.1	DUF1972 domain-containing protein	N	N	N	N	N	N
WP_107379589.1	Tn3 family transposase	N	N	N	N	N	N
WP_107379596.1	alcohol dehydrogenase catalytic domain-containing protein	N	N	N	N	N	N
WP_107379599.1	NTP transferase domain-containing protein	N	N	N	N	N	N
WP_107379600.1	XdhC family protein	N	N	N	N	N	N
WP_107379603.1	nuclear transport factor 2 family protein	N	N	N	N	N	N
WP_107380114.1	hypothetical protein	N	N	N	N	N	N
WP_107380556.1	site-specific DNA-methyltransferase	NZ_CP028358.1-4	100	N	N	N	N
WP_180895277.	metallohydrolase	N	N	N	N	N	N

	1							
	WP_197596969.1	vitamin K epoxide reductase family protein	N	N	N	N	N	N
	WP_199849297.1	multidrug effflux MFS transporter	N	N	N	N	N	N
	WP_199849316.1	restriction endonuclease	N	N	N	N	N	N
	WP_003107109.1	helix-turn-helix domain-containing protein	N	N	N	N	N	N
	WP_005416649.1	RebB family R body protein	N	N	N	N	N	N
	WP_005416651.1	RebB family R body protein	N	N	N	N	N	N
	WP_005996621.1	arsenic transporter	N	N	N	N	N	N
NZ_CP028899.1	WP_006399830.1	cytochrome o ubiquinol oxidase subunit IV	N	N	N	N	N	N
	WP_006472389.1	VirB3 family type IV secretion system protein	N	N	N	N	N	N
	WP_008264806.1	hypothetical protein	N	N	N	N	N	N
	WP_008264906.1	HupE/UreJ family protein	N	N	N	N	N	N
	WP_008266162.1	CusA/CzcA family heavy metal efflux RND transporter	N	N	N	N	N	N
	WP_008266446.	SDR family oxidoreductase	N	N	N	N	N	N

1							
WP_008267238.1	MFS transporter	N	N	N	N	N	N
WP_010481480.1	DUF2188 domain-containing protein	N	N	N	N	N	N
WP_011516230.1	helix-turn-helix transcriptional regulator	N	N	N	N	N	N
WP_012204563.1	TrbC/VirB2 family protein	N	N	N	N	N	N
WP_012204568.1	LysR family transcriptional regulator	N	N	N	N	N	N
WP_012204575.1	cytochrome b	N	N	N	N	N	N
WP_012204576.1	VIT family protein	N	N	N	N	N	N
WP_014159627.1	metalloregulator ArsR/SmtB family transcription factor	N	N	N	N	N	N
WP_014995090.1	helix-turn-helix domain-containing protein	N	N	N	N	N	N
WP_017357195.1	isoprenylcysteine carboxylmethyltransferase family protein	N	N	N	N	N	N
WP_020230337.1	VirB3 family type IV secretion system protein	N	N	N	N	N	N
WP_027457565.1	P-type conjugative transfer protein TrbJ	N	N	N	N	N	N
WP_027457577.	helix-turn-helix domain-containing	N	N	N	N	N	N

1	protein						
WP_027457578.1	ArsI/CadI family heavy metal resistance metalloenzyme	N	N	N	N	N	N
WP_027457579.1	arsenate reductase ArsC	N	N	N	N	N	N
WP_032957219.1	pilin	N	N	N	N	N	N
WP_032957905.1	glycosyltransferase family 2 protein	N	N	N	N	N	N
WP_032958497.1	GNAT family N-acetyltransferase	N	N	N	N	N	N
WP_032959298.1	SRPBCC family protein	N	N	N	N	N	N
WP_032962419.1	SDR family oxidoreductase	N	N	N	N	N	N
WP_033946869.1	methyltransferase domain-containing protein	N	N	N	N	N	N
WP_033946871.1	LysR substrate-binding domain-containing protein	N	N	N	N	N	N
WP_033946873.1	EexN family lipoprotein	N	N	N	N	N	N
WP_033946875.1	ribbon-helix-helix protein, CopG family	N	N	N	N	N	N
WP_033946878.1	TrbC/VirB2 family protein	N	N	N	N	N	N
WP_033946886.	P-type conjugative transfer protein TrbJ	N	N	N	N	N	N

1							
WP_033946891.1	P-type conjugative transfer protein TrbL	N	N	N	N	N	N
WP_033946893.1	conjugal transfer protein TrbF	N	N	N	N	N	N
WP_033946896.1	P-type conjugative transfer protein TrbG	N	N	N	N	N	N
WP_033946899.1	TrbI/VirB10 family protein	N	N	N	N	N	N
WP_039388932.1	DUF2958 domain-containing protein	N	N	N	N	N	N
WP_046986737.1	IS66 family insertion sequence element accessory protein TnpB	N	N	N	N	N	N
WP_048629390.1	P-type conjugative transfer protein TrbL	N	N	N	N	N	N
WP_048629391.1	conjugal transfer protein TrbF	N	N	N	N	N	N
WP_048629392.1	P-type conjugative transfer protein TrbG	N	N	N	N	N	N
WP_049404314.1	LysR family transcriptional regulator	N	N	N	N	N	N
WP_049404620.1	arsenate reductase ArsC	N	N	N	N	N	N
WP_049404642.1	heavy metal translocating P-type ATPase	N	N	N	N	N	N
WP_049404902.	type ISP restriction/modification enzyme	N	N	N	N	N	N

1							
WP_049405055.1	VWA domain-containing protein	N	N	N	N	N	N
WP_049405238.1	TrbI/VirB10 family protein	N	N	N	N	N	N
WP_049405254.1	ribbon-helix-helix protein, CopG family	N	N	N	N	N	N
WP_049405830.1	ferric reductase-like transmembrane domain-containing protein	N	N	N	N	N	N
WP_049405833.1	S26 family signal peptidase	N	N	N	N	N	N
WP_049405835.1	DUF2840 domain-containing protein	N	N	N	N	N	N
WP_049405837.1	ParA family partition ATPase	N	N	N	N	N	N
WP_049405840.1	replication initiator protein A	N	N	N	N	N	N
WP_049405843.1	DUF2285 domain-containing protein	N	N	N	N	N	N
WP_049405853.1	DUF736 domain-containing protein	N	N	N	N	N	N
WP_049405859.1	chromate efflux transporter	N	N	N	N	N	N
WP_049405865.1	ParB/RepB/Spo0J family partition protein	N	N	N	N	N	N
WP_049405868.	DUF932 domain-containing protein	N	N	N	N	N	N

1							
WP_049405870.1	DNA repair protein RadC	N	N	N	N	N	N
WP_049405874.1	site-specific integrase	N	N	N	N	N	N
WP_049405882.1	hypothetical protein	N	N	N	N	N	N
WP_049406852.1	TIGR03885 family FMN-dependent LLM class oxidoreductase	N	N	N	N	N	N
WP_049408461.1	3'-5' exonuclease	N	N	N	N	N	N
WP_049408511.1	hypothetical protein	N	N	N	N	N	N
WP_049408513.1	helix-turn-helix domain-containing protein	N	N	N	N	N	N
WP_049408515.1	LysR family transcriptional regulator	N	N	N	N	N	N
WP_049408519.1	heat resistance protein PsiE-GI	N	N	N	N	N	N
WP_049419566.1	trypsin-like peptidase domain-containing protein	N	N	N	N	N	N
WP_049419571.1	IS5 family transposase	N	N	N	N	N	N
WP_052758892.1	arsenate reductase (glutaredoxin)	N	N	N	N	N	N
WP_053091707.	aldehyde dehydrogenase family protein	N	N	N	N	N	N

1							
WP_080354599.1	heavy metal translocating P-type ATPase	N	N	NZ_CP040439.1-4	100	N	N
WP_087786465.1	CusA/CzcA family heavy metal efflux RND transporter	N	N	N	N	N	N
WP_087786553.1	S26 family signal peptidase	N	N	N	N	N	N
WP_087786554.1	DUF2840 domain-containing protein	N	N	N	N	N	N
WP_087786555.1	ParA family partition ATPase	N	N	NZ_CP028899.1-8	100	N	N
WP_087786556.1	IS21 family transposase	N	N	NZ_CP028899.1-8	100	N	N
WP_087786557.1	IS21-like element helper ATPase IstB	N	N	NZ_CP028899.1-8	100	N	N
WP_087786558.1	DUF2285 domain-containing protein	N	N	NZ_CP028899.1-8	100	N	N
WP_087786559.1	DUF2958 domain-containing protein	N	N	N	N	N	N
WP_087786561.1	DUF736 domain-containing protein	N	N	N	N	N	N
WP_087786563.1	ParB/RepB/Spo0J family partition protein	N	N	N	N	N	N
WP_087786564.1	DUF932 domain-containing protein	N	N	N	N	N	N
WP_087786570.	DNA repair protein RadC	N	N	N	N	N	N

	1							
	WP_087786571.1	PDDEXK nuclease domain-containing protein	N	N	N	N	N	N
	WP_087786572.1	site-specific integrase	N	N	N	N	N	N
	WP_087786576.1	chromosome partitioning protein ParB	N	N	N	N	N	N
	WP_087786578.1	IS66 family transposase	N	N	N	N	N	N
	WP_099475629.1	ABC transporter permease	N	N	N	N	N	N
	WP_197573146.1	SIR2 family protein	N	N	N	N	N	N
	WP_198421464.1	PadR family transcriptional regulator	N	N	N	N	N	N
	WP_199912530.1	diacylglycerol kinase	N	N	N	N	N	N
	WP_223277441.1	PadR family transcriptional regulator	N	N	N	N	N	N
	WP_234413153.1	GlxA family transcriptional regulator	N	N	N	N	N	N
	WP_003097544.1	IS21-like element ISPa36 family helper ATPase IstB	N	N	N	N	N	N
NZ_CP029773.1	WP_003097546.1	IS21-like element ISPa36 family transposase	N	N	N	N	N	N
	WP_006399830.	cytochrome o ubiquinol oxidase subunit	N	N	N	N	N	N

1	IV						
WP_008264806.1	hypothetical protein	N	N	N	N	N	N
WP_008264906.1	HupE/UreJ family protein	N	N	N	N	N	N
WP_008266162.1	CusA/CzcA family heavy metal efflux RND transporter	N	N	N	N	N	N
WP_008267238.1	MFS transporter	N	N	N	N	N	N
WP_032967187.1	metalloregulator ArsR/SmtB family transcription factor	N	N	N	N	N	N
WP_046430574.1	dihydrolipoyl dehydrogenase	N	N	N	N	N	N
WP_046430586.1	heavy metal translocating P-type ATPase	N	N	N	N	N	N
WP_046982950.1	PstS family phosphate ABC transporter substrate-binding protein	N	N	N	N	N	N
WP_049399243.1	MepB family protein	N	N	N	N	N	N
WP_049400072.1	DinB family protein	N	N	N	N	N	N
WP_049429062.1	GIY-YIG nuclease family protein	N	N	N	N	N	N
WP_049444104.1	GPW/gp25 family protein	N	N	N	N	N	N
WP_102789622.	phosphate ABC transporter permease	N	N	N	N	N	N

1	PstA						
WP_107379596.1	alcohol dehydrogenase catalytic domain-containing protein	N	N	N	N	N	N
WP_110710875.1	DNA (cytosine-5-)-methyltransferase	N	N	N	N	N	N
WP_110710968.1	ABC transporter ATP-binding protein	N	N	N	N	N	N
WP_110711253.1	integrase arm-type DNA-binding domain-containing protein	N	N	NZ_CP029773.1-3	100	N	N
WP_110711535.1	bifunctional helix-turn-helix transcriptional regulator/GNAT family N-acetyltransferase	N	N	N	N	N	N
WP_110711547.1	alpha/beta hydrolase	N	N	N	N	N	N
WP_110711813.1	NAD(P)H-dependent oxidoreductase	N	N	N	N	N	N
WP_110711814.1	LysR family transcriptional regulator	N	N	N	N	N	N
WP_110711957.1	redox-sensitive transcriptional activator SoxR	N	N	N	N	N	N
WP_110711958.1	MFS transporter	N	N	N	N	N	N
WP_110712036.1	restriction endonuclease	N	N	N	N	N	N
WP_110712037.1	class I SAM-dependent DNA methyltransferase	N	N	N	N	N	N

WP_110712039.1	type I restriction endonuclease subunit R	N	N	N	N	N	N
WP_110712085.1	NAD-dependent epimerase/dehydratase family protein	N	N	N	N	N	N
WP_110712196.1	PDDEXK nuclease domain-containing protein	N	N	N	N	N	N
WP_110712294.1	alpha/beta hydrolase	N	N	N	N	N	N
WP_110712296.1	LysR family transcriptional regulator	N	N	N	N	N	N
WP_110712299.1	VOC family protein	N	N	N	N	N	N
WP_110712302.1	SDR family oxidoreductase	N	N	N	N	N	N
WP_110712305.1	HlyD family secretion protein	N	N	N	N	N	N
WP_110712307.1	FAD-dependent oxidoreductase	N	N	N	N	N	N
WP_110712310.1	NAD(P)H-dependent oxidoreductase	NZ_CP029773.1-2	100	N	N	N	N
WP_110712312.1	LysR family transcriptional regulator	N	N	N	N	N	N
WP_110712313.1	pirin family protein	NZ_CP029773.1-2	100	N	N	N	N
WP_110712343.1	MBL fold metallo-hydrolase	N	N	N	N	N	N

WP_110712377.1	MFS transporter	N	N	N	N	N	N
WP_110712403.1	MerR family transcriptional regulator	N	N	N	N	N	N
WP_110712735.1	YjhX family toxin	N	N	N	N	N	N
WP_110712759.1	DNA methyltransferase	N	N	N	N	N	N
WP_110714012.1	DUF2185 domain-containing protein	N	N	N	N	N	N
WP_110714163.1	type VI secretion system contractile sheath small subunit	N	N	N	N	N	N
WP_110714317.1	AraC family transcriptional regulator	N	N	N	N	N	N
WP_174704372.1	phosphate ABC transporter permease subunit PstC	N	N	N	N	N	N
WP_197596969.1	vitamin K epoxide reductase family protein	N	N	N	N	N	N
WP_199849297.1	multidrug effflux MFS transporter	N	N	N	N	N	N
WP_204353095.1	recombinase family protein	N	N	N	N	N	N
WP_239496490.1	LysR family transcriptional regulator	N	N	N	N	N	N
WP_239496500.1	GrpB family protein	N	N	N	N	N	N

NZ_CP031058.1	WP_239496526.1	isocitrate lyase/PEP mutase family protein	N	N	N	N	N	N
	WP_239496566.1	oxidoreductase	N	N	N	N	N	N
	WP_239496569.1	phosphate ABC transporter ATP-binding protein PstB	N	N	N	N	N	N
	WP_000085131.1	ribbon-helix-helix protein, CopG family	N	N	N	N	N	N
	WP_000163574.1	tetracycline resistance transcriptional repressor TetR(G)	N	N	N	N	N	N
	WP_000251875.1	phosphoglucosamine mutase	N	N	N	N	N	N
	WP_000480968.1	aminoglycoside O-phosphotransferase APH(6)-Id	N	N	N	N	NZ_CP104863.1-4	100
	WP_000539741.1	multidrug efflux SMR transporter	N	N	N	N	N	N
	WP_000904906.1	recombinase family protein	N	N	N	N	N	N
	WP_001043260.1	sulfonamide-resistant dihydropteroate synthase Sul2	N	N	N	N	N	N
	WP_001082319.1	aminoglycoside O-phosphotransferase APH(3")-Ib	N	N	N	N	N	N
	WP_001138082.1	Tn3 family transposase	N	N	N	N	N	N
	WP_001255015.1	LysR family transcriptional regulator	N	N	N	N	N	N

WP_001257840.1	tetracycline efflux MFS transporter Tet(G)	N	N	N	N	N	N
WP_001447541.1	DUF3363 domain-containing protein	N	N	N	N	N	N
WP_003092277.1	P-type conjugative transfer protein TrbJ	N	N	N	N	N	N
WP_006399830.1	cytochrome o ubiquinol oxidase subunit IV	N	N	N	N	N	N
WP_008786730.1	glutaredoxin family protein	N	N	N	N	N	N
WP_009459729.1	AlpA family transcriptional regulator	N	N	N	N	N	N
WP_014603538.1	hypothetical protein	N	N	N	N	N	N
WP_019726333.1	hypothetical protein	N	N	N	N	N	N
WP_021156365.1	DUF1016 N-terminal domain-containing protein	N	N	N	N	N	N
WP_021156366.1	site-specific integrase	N	N	N	N	N	N
WP_021162757.1	S26 family signal peptidase	N	N	N	N	N	N
WP_021162758.1	DUF2840 domain-containing protein	N	N	N	N	N	N
WP_021162762.1	helix-turn-helix domain-containing protein	N	N	N	N	N	N

WP_021162763.1	DUF2285 domain-containing protein	N	N	N	N	N	N
WP_021162764.1	DUF2958 domain-containing protein	N	N	N	N	N	N
WP_021162766.1	DUF736 domain-containing protein	N	N	N	N	N	N
WP_021264217.1	TIGR03746 family integrating conjugative element protein	N	N	N	N	N	N
WP_021264218.1	TIGR03750 family conjugal transfer protein	N	N	N	N	N	N
WP_021264219.1	TIGR03745 family integrating conjugative element membrane protein	N	N	N	N	N	N
WP_021264220.1	TIGR03758 family integrating conjugative element protein	N	N	N	N	N	N
WP_021264221.1	TIGR03747 family integrating conjugative element membrane protein	N	N	N	N	N	N
WP_021264225.1	PilL N-terminal domain-containing protein	N	N	N	N	N	N
WP_021264227.1	helicase-related protein	N	N	N	N	N	N
WP_021264228.1	hypothetical protein	N	N	N	N	N	N
WP_021264230.1	DUF6094 domain-containing protein	N	N	N	N	N	N
WP_021264231.1	hypothetical protein	N	N	N	N	N	N

WP_021264233.1	hypothetical protein	N	N	N	N	N	N
WP_021264237.1	phosphoadenosine phosphosulfate reductase family protein	N	N	N	N	N	N
WP_021264238.1	DUF3085 domain-containing protein	N	N	N	N	N	N
WP_021264240.1	hypothetical protein	N	N	N	N	N	N
WP_021264242.1	hypothetical protein	N	N	N	N	N	N
WP_021264243.1	hypothetical protein	N	N	N	N	N	N
WP_021264249.1	DNA topoisomerase III	N	N	N	N	N	N
WP_021264250.1	single-stranded DNA-binding protein	N	N	N	N	N	N
WP_022579739.1	Hg(II)-responsive transcriptional regulator	N	N	N	N	N	N
WP_022579740.1	mercuric ion transporter MerT	N	N	N	N	N	N
WP_022579741.1	mercury resistance system periplasmic binding protein MerP	N	N	N	N	N	N
WP_022579743.1	mercury resistance co-regulator MerD	N	N	N	N	N	N
WP_022579749.1	P-type conjugative transfer protein TrbJ	N	N	N	N	N	N

WP_022579750.1	conjugal transfer transcriptional regulator TraJ	N	N	N	N	N	N
WP_022579752.1	replication protein C, IncQ-type	N	N	N	N	N	N
WP_022579753.1	helicase RepA family protein	N	N	N	N	N	N
WP_022579756.1	AlpA family phage regulatory protein	N	N	N	N	N	N
WP_022580324.1	DUF3577 domain-containing protein	N	N	N	N	N	N
WP_022580326.1	TIGR03759 family integrating conjugative element protein	N	N	N	N	N	N
WP_022580327.1	transglycosylase SLT domain-containing protein	N	N	N	N	N	N
WP_022580328.1	integrating conjugative element protein	N	N	N	N	N	N
WP_022580329.1	RAQPRD family integrative conjugative element protein	N	N	N	N	N	N
WP_022580330.1	TIGR03749 family integrating conjugative element protein	N	N	N	N	N	N
WP_022580331.1	TIGR03752 family integrating conjugative element protein	N	N	N	N	N	N
WP_022580333.1	DsbA family protein	N	N	N	N	N	N
WP_022580370.1	carboxymuconolactone decarboxylase family protein	N	N	N	N	N	N

WP_022580371.1	DUF302 domain-containing protein	N	N	N	N	N	N
WP_022580372.1	DoxX family protein	N	N	N	N	N	N
WP_022580375.1	DUF692 domain-containing protein	N	N	N	N	N	N
WP_022580377.1	RNA polymerase sigma factor	N	N	N	N	N	N
WP_022580381.1	thioredoxin family protein	N	N	N	N	N	N
WP_022580390.1	IS5-like element ISPa100 family transposase	N	N	N	N	N	N
WP_022580391.1	arsenate reductase ArsC	N	N	N	N	N	N
WP_022580560.1	Na+/H+ antiporter NhaA	N	N	N	N	N	N
WP_022580562.1	universal stress protein	N	N	N	N	N	N
WP_022580566.1	tyrosine-type recombinase/integrase	N	N	N	N	N	N
WP_022581019.1	TIGR03757 family integrating conjugative element protein	N	N	N	N	N	N
WP_022581021.1	integrating conjugative element protein	N	N	N	N	N	N
WP_022581022.1	hypothetical protein	N	N	N	N	N	N

WP_022581023.1	conjugal transfer protein TraG N-terminal domain-containing protein	N	N	N	N	N	N
WP_023082437.1	S8 family peptidase	N	N	N	N	N	N
WP_023093371.1	ATP-binding cassette domain-containing protein	N	N	N	N	N	N
WP_023093472.1	TIGR03761 family integrating conjugative element protein	N	N	N	N	N	N
WP_023093473.1	STY4528 family pathogenicity island replication protein	N	N	N	N	N	N
WP_023093474.1	DUF2857 domain-containing protein	N	N	N	N	N	N
WP_023093475.1	ParB family protein	N	N	N	N	N	N
WP_023093476.1	ParA family protein	N	N	N	N	N	N
WP_023093477.1	hypothetical protein	N	N	N	N	N	N
WP_023123965.1	DUF4917 family protein	N	N	N	N	N	N
WP_023123968.1	hypothetical protein	N	N	N	N	N	N
WP_023434798.1	DUF3275 family protein	N	N	N	N	N	N
WP_023435210.1	JAB domain-containing protein	N	N	N	N	N	N

WP_023435212.1	TIGR03751 family conjugal transfer lipoprotein	N	N	N	N	N	N
WP_023912020.1	helix-turn-helix transcriptional regulator	N	N	N	N	N	N
WP_023912021.1	cytochrome c biogenesis CcdA family protein	N	N	N	N	N	N
WP_023912026.1	permease	N	N	N	N	N	N
WP_023912031.1	helix-turn-helix domain-containing protein	N	N	N	N	N	N
WP_023912032.1	MobH family relaxase	N	N	N	N	N	N
WP_023980278.1	ATP-binding protein	N	N	N	N	N	N
WP_024008120.1	hypothetical protein	N	N	N	N	N	N
WP_031294484.1	mercury(II) reductase	N	N	N	N	N	N
WP_032127235.1	LysR substrate-binding domain-containing protein	N	N	N	N	N	N
WP_032127338.1	GNAT family N-acetyltransferase	N	N	N	N	N	N
WP_032127914.1	cupin domain-containing protein	N	N	N	N	N	N
WP_032129827.1	phage major capsid protein	N	N	NZ_CP031058.1-6	100	N	N

WP_034203400.1	helix-turn-helix transcriptional regulator	N	N	N	N	N	N
WP_049398866.1	isocitrate lyase/PEP mutase family protein	N	N	N	N	N	N
WP_049422288.1	MFS transporter	N	N	N	N	N	N
WP_049442707.1	type II toxin-antitoxin system RelE/ParE family toxin	N	N	N	N	N	N
WP_049462201.1	efflux transporter outer membrane subunit	N	N	N	N	N	N
WP_049462202.1	efflux RND transporter permease subunit	N	N	N	N	N	N
WP_049462203.1	efflux RND transporter periplasmic adaptor subunit	N	N	N	N	N	N
WP_049462207.1	metalloregulator ArsR/SmtB family transcription factor	N	N	N	N	N	N
WP_049462210.1	glutaredoxin	N	N	N	N	N	N
WP_050157749.1	LysR family transcriptional regulator	N	N	N	N	N	N
WP_050157758.1	TrbC/VirB2 family protein	N	N	N	N	N	N
WP_050157760.1	P-type conjugative transfer protein TrbL	N	N	N	N	N	N
WP_050157812.1	LysR family transcriptional regulator	N	N	N	N	N	N

WP_050597112.1	arsenite efflux transporter metallochaperone ArsD	N	N	N	N	N	N
WP_071557723.1	GDCCVxC domain-containing (seleno)protein	N	N	N	N	N	N
WP_071557724.1	permease	N	N	N	N	N	N
WP_071567713.1	EexN family lipoprotein	N	N	N	N	N	N
WP_132809718.1	XRE family transcriptional regulator	N	N	N	N	N	N
WP_134952144.1	metalloregulator ArsR/SmtB family transcription factor	N	N	N	N	N	N
WP_149908498.1	DHA2 family efflux MFS transporter permease subunit	N	N	N	N	N	N
WP_149909467.1	cation diffusion facilitator family transporter	N	N	N	N	N	N
WP_151608092.1	integrase domain-containing protein	N	N	N	N	N	N
WP_151608097.1	DUF4433 domain-containing protein	N	N	N	N	N	N
WP_151608098.1	macro domain-containing protein	N	N	N	N	N	N
WP_169708473.1	metal-sensing transcriptional repressor	N	N	N	N	N	N
WP_182267226.1	ABC transporter permease	N	N	N	N	N	N

WP_182267227.1	ABC transporter ATP-binding protein	N	N	N	N	N	N
WP_182333738.1	ABC transporter permease	N	N	N	N	N	N
WP_189719458.1	DNA repair exonuclease	N	N	N	N	N	N
WP_189719461.1	ATP-binding protein	N	N	N	N	N	N
WP_189719562.1	DUF1972 domain-containing protein	N	N	N	N	N	N
WP_189719679.1	MFS transporter	N	N	N	N	N	N
WP_189719680.1	redox-sensitive transcriptional activator SoxR	N	N	N	N	N	N
WP_189719745.1	DUF3158 family protein	N	N	N	N	N	N
WP_189719749.1	IS21-like element helper ATPase IstB	N	N	N	N	N	N
WP_189719750.1	IS21 family transposase	N	N	N	N	N	N
WP_189719794.1	hypothetical protein	N	N	NZ_CP031058.1-5	100	N	N
WP_189719795.1	DUF935 domain-containing protein	N	N	NZ_CP031058.1-5	100	N	N
WP_189719800.1	Mu-like prophage major head subunit gpT family protein	N	N	NZ_CP031058.1-5	100	N	N

WP_189719817.1	DMT family transporter	N	N	N	N	N	N
WP_189719889.1	Clp protease ClpP	N	N	NZ_CP031058.1-6	100	N	N
WP_189719891.1	terminase large subunit	N	N	NZ_CP031058.1-2, NZ_CP031058.1-6	100	N	N
WP_189720073.1	M24 family metallopeptidase	N	N	N	N	N	N
WP_189720089.1	DEAD/DEAH box helicase family protein	N	N	N	N	N	N
WP_189720091.1	SNF2-related protein	N	N	N	N	N	N
WP_189720092.1	WYL domain-containing protein	N	N	N	N	N	N
WP_189720346.1	AAA family ATPase	N	N	N	N	N	N
WP_189720350.1	MFS transporter	N	N	N	N	N	N
WP_189720351.1	MFS transporter	N	N	N	N	N	N
WP_189720447.1	Clp protease ClpP	N	N	NZ_CP031058.1-2	100	N	N
WP_189720448.1	phage major capsid protein	N	N	NZ_CP031058.1-2	100	N	N
WP_189721113.1	TonB-dependent receptor	N	N	N	N	N	N

	WP_189721177.1	NAD(P)-dependent alcohol dehydrogenase	N	N	N	N	N	N
	WP_223276810.1	LysR substrate-binding domain-containing protein	N	N	N	N	N	N
	WP_223276826.1	ABC transporter ATP-binding protein	N	N	N	N	N	N
	WP_223276833.1	DUF3164 family protein	N	N	NZ_CP031058.1-5	100	N	N
	WP_223276835.1	AraC family transcriptional regulator	N	N	N	N	N	N
	WP_223276836.1	AraC family ligand binding domain-containing protein	N	N	N	N	N	N
	WP_223276853.1	GrpB family protein	N	N	N	N	N	N
	WP_223276891.1	hypothetical protein	N	N	N	N	N	N
	WP_223276893.1	terminase TerL endonuclease subunit	N	N	N	N	N	N
	WP_223276903.1	IS3 family transposase	N	N	N	N	N	N
	WP_239498451.1	LysR family transcriptional regulator	N	N	N	N	N	N
	WP_262984416.1	terminase large subunit	N	N	NZ_CP031058.1-2, NZ_CP031058.1-6, NZ_CP067993.1-4	100	N	N
NZ_CP033586.1	WP_111197640.	UDP-N-acetylglucosamine 2-epimerase	N	N	N	N	N	N

	1	(non-hydrolyzing)						
	WP_133050666.1	GIY-YIG nuclease family protein	N	N	N	N	N	N
	WP_133050669.1	DNA methyltransferase	N	N	N	N	N	N
	WP_229298665.1	GlxA family transcriptional regulator	N	N	N	N	N	N
	WP_240791806.1	nuclear transport factor 2 family protein	N	N	N	N	N	N
	WP_240791831.1	SDR family oxidoreductase	N	N	N	N	N	N
	WP_005408332.1	GPW/gp25 family protein	N	N	N	N	N	N
	WP_024957746.1	transcriptional regulator	N	N	N	N	N	N
	WP_049449119.1	nuclear transport factor 2 family protein	N	N	N	N	N	N
NZ_CP033829.1	WP_049449966.1	ABC transporter ATP-binding protein	N	N	N	N	N	N
	WP_049449973.1	glycosyltransferase family 4 protein	N	N	N	N	N	N
	WP_049449986.1	WxcM-like domain-containing protein	N	N	N	N	N	N
	WP_049449988.1	glycosyltransferase family 2 protein	N	N	N	N	N	N
	WP_049449989.	DegT/DnrJ/EryC1/StrS family	N	N	N	N	N	N

1	aminotransferase						
WP_049450269.1	hypothetical protein	N	N	N	N	N	N
WP_088435499.1	BPL-N domain-containing protein	N	N	N	N	N	N
WP_088436191.1	DJ-1/PfpI family protein	N	N	N	N	N	N
WP_123958202.1	DegT/DnrJ/EryC1/StrS family aminotransferase	N	N	N	N	N	N
WP_123958559.1	SDR family oxidoreductase	N	N	N	N	N	N
WP_123958560.1	aldehyde dehydrogenase family protein	N	N	N	N	N	N
WP_123958561.1	AraC family transcriptional regulator	N	N	N	N	N	N
WP_123958632.1	amidohydrolase	N	N	N	N	N	N
WP_123958783.1	zincin-like metallopeptidase domain-containing protein	N	N	N	N	N	N
WP_123958900.1	Gp49 family protein	N	N	N	N	N	N
WP_123958901.1	lysozyme	N	N	N	N	N	N
WP_123958939.1	YqaJ viral recombinase family protein	N	N	N	N	N	N
WP_123959183.	cytochrome o ubiquinol oxidase subunit	N	N	N	N	N	N

	1	IV						
	WP_202151436.1	amidase family protein	N	N	N	N	N	N
	WP_216641836.1	DUF1629 domain-containing protein	N	N	N	N	N	N
	WP_234411807.1	Rid family hydrolase	N	N	N	N	N	N
	WP_253682435.1	SDR family oxidoreductase	N	N	N	N	N	N
	WP_253682450.1	very short patch repair endonuclease	N	N	N	N	N	N
	WP_005416649.1	RebB family R body protein	N	N	N	N	N	N
	WP_005416651.1	RebB family R body protein	N	N	N	N	N	N
	WP_006399830.1	cytochrome o ubiquinol oxidase subunit IV	N	N	N	N	N	N
NZ_CP033877.1	WP_006446865.1	RebB family R body protein	N	N	N	N	N	N
	WP_014037326.1	heavy metal response regulator transcription factor	N	N	N	N	N	N
	WP_049445059.1	RebB family R body protein	N	N	N	N	N	N
	WP_101766100.1	MepB family protein	N	N	N	N	N	N
	WP_164117654.	multidrug/biocide efflux PACE	N	N	N	N	N	N

1	transporter						
WP_188486473.1	metalloregulator ArsR/SmtB family transcription factor	N	N	N	N	N	N
WP_188486475.1	arsenate reductase ArsC	N	N	N	N	N	N
WP_188486477.1	arsenate reductase (glutaredoxin)	N	N	N	N	N	N
WP_188486481.1	arsenic transporter	N	N	N	N	N	N
WP_188486679.1	ParB-like protein	N	N	N	N	N	N
WP_188487926.1	VOC family protein	N	N	N	N	N	N
WP_188488787.1	Rrf2 family transcriptional regulator	N	N	N	N	N	N
WP_188488789.1	NAD(P)/FAD-dependent oxidoreductase	N	N	N	N	N	N
WP_188488823.1	class I SAM-dependent methyltransferase	N	N	N	N	N	N
WP_188488840.1	SRPBCC family protein	N	N	N	N	N	N
WP_188489180.1	helix-turn-helix domain-containing protein	N	N	N	N	N	N
WP_188489186.1	AAA family ATPase	N	N	N	N	N	N
WP_188489190.	restriction endonuclease	N	N	N	N	N	N

	1							
	WP_188489255.1	LysR family transcriptional regulator	N	N	N	N	N	N
	WP_188489479.1	alpha/beta fold hydrolase	N	N	N	N	N	N
	WP_188489683.1	FAD-dependent monooxygenase	N	N	N	N	N	N
	WP_188489685.1	LysR family transcriptional regulator	N	N	N	N	N	N
	WP_188489689.1	MFS transporter	N	N	N	N	N	N
	WP_188489691.1	aldo/keto reductase	N	N	N	N	N	N
	WP_223224474.1	LysR family transcriptional regulator	N	N	N	N	N	N
	WP_223224526.1	SDR family oxidoreductase	N	N	N	N	N	N
	WP_223224534.1	BPL-N domain-containing protein	N	N	N	N	N	N
	WP_005408332.1	GPW/gp25 family protein	N	N	N	N	N	N
NZ_CP037858.1	WP_005411473.1	cytochrome o ubiquinol oxidase subunit IV	N	N	N	N	N	N
	WP_005411609.1	acyl carrier protein	N	N	N	N	N	N
	WP_005416648.	RebB family R body protein	N	N	N	N	N	N

1							
WP_005416649.1	RebB family R body protein	N	N	N	N	N	N
WP_005416651.1	RebB family R body protein	N	N	N	N	N	N
WP_006425769.1	EamA family transporter	N	N	N	N	N	N
WP_006446865.1	RebB family R body protein	N	N	N	N	N	N
WP_012509968.1	ABC transporter ATP-binding protein	N	N	N	N	N	N
WP_019338970.1	DNA-binding transcriptional regulator	N	N	N	N	N	N
WP_046985218.1	transcriptional regulator	N	N	N	N	N	N
WP_049441719.1	sulfite exporter TauE/SafE family protein	N	N	N	N	N	N
WP_049441726.1	chromate efflux transporter	N	N	N	N	N	N
WP_049441864.1	ATP-binding protein	N	N	N	N	N	N
WP_049441866.1	metalloregulator ArsR/SmtB family transcription factor	N	N	N	N	N	N
WP_080355804.1	hypothetical protein	N	N	N	N	N	N
WP_099484974.	metalloregulator ArsR/SmtB family	N	N	N	N	N	N

1	transcription factor						
WP_099551046.1	DUF3649 domain-containing protein	N	N	N	N	N	N
WP_099551058.1	DUF3224 domain-containing protein	N	N	N	N	N	N
WP_099553200.1	glyoxalase	N	N	N	N	N	N
WP_099553449.1	ABC transporter ATP-binding protein	N	N	N	N	N	N
WP_099553451.1	iron ABC transporter permease	N	N	N	N	N	N
WP_099554013.1	MotA/TolQ/ExbB proton channel family protein	N	N	N	N	N	N
WP_111105525.1	ATP-binding protein	N	N	N	N	N	N
WP_114619015.1	arsenate reductase ArsC	N	N	N	N	N	N
WP_142805947.1	GNAT family N-acetyltransferase	N	N	N	N	N	N
WP_142806012.1	hypothetical protein	N	N	N	N	N	N
WP_142806022.1	NAD(P)-dependent oxidoreductase	N	N	N	N	N	N
WP_142806037.1	PepSY-associated TM helix domain-containing protein	N	N	N	N	N	N
WP_142806102.	cobaltochelatase subunit CobN	N	N	N	N	N	N

1							
WP_142806103.1	DUF2149 domain-containing protein	N	N	N	N	N	N
WP_142806248.1	helix-turn-helix domain-containing protein	N	N	N	N	N	N
WP_142806347.1	glutathione transferase GstA	N	N	N	N	N	N
WP_142806806.1	alpha/beta hydrolase	N	N	N	N	N	N
WP_142806847.1	TonB-dependent receptor	N	N	N	N	N	N
WP_142806848.1	alpha-D-ribose 1-methylphosphonate 5-triphosphate diphosphatase	N	N	N	N	N	N
WP_142806850.1	DUF5690 family protein	N	N	N	N	N	N
WP_142806972.1	L-dopachrome tautomerase-related protein	N	N	N	N	N	N
WP_142807192.1	UDP-glucose 4-epimerase GalE	N	N	N	N	N	N
WP_142807268.1	LysR family transcriptional regulator	N	N	N	N	N	N
WP_142807269.1	MFS transporter	N	N	N	N	N	N
WP_142807367.1	phage tail sheath subtilisin-like domain-containing protein	N	N	NZ_CP037858.1-1	100	N	N
WP_142807544.	response regulator	N	N	N	N	N	N

1							
WP_142807679.1	SRPBCC family protein	N	N	N	N	N	N
WP_142807702.1	TonB-dependent receptor	N	N	N	N	N	N
WP_142807792.1	DUF2075 domain-containing protein	N	N	N	N	N	N
WP_142807793.1	nucleotide pyrophosphohydrolase	N	N	N	N	N	N
WP_142807808.1	amino acid ABC transporter permease/ATP-binding protein	N	N	N	N	N	N
WP_142807813.1	LysR substrate-binding domain-containing protein	N	N	N	N	N	N
WP_142807822.1	GFA family protein	N	N	N	N	N	N
WP_142807978.1	TonB-dependent receptor	N	N	N	N	N	N
WP_142807979.1	MFS transporter	N	N	N	N	N	N
WP_221931195.1	hypothetical protein	N	N	N	N	N	N
WP_221931199.1	helix-turn-helix domain-containing protein	N	N	N	N	N	N
WP_221931201.1	cyanase	N	N	N	N	N	N
WP_239503745.	rhodanese-like domain-containing	N	N	N	N	N	N

	1	protein						
	WP_239503788.1	hypothetical protein	N	N	N	N	N	N
	WP_260678128.1	nuclear transport factor 2 family protein	N	N	N	N	N	N
	WP_260678178.1	ABC transporter ATP-binding protein	N	N	N	N	N	N
	WP_260678181.1	ABC transporter ATP-binding protein	N	N	N	N	N	N
	WP_260678183.1	helix-turn-helix transcriptional regulator	N	N	N	N	N	N
	WP_260678240.1	LysR family transcriptional regulator	N	N	N	N	N	N
	WP_260678405.1	TIGR03364 family FAD-dependent oxidoreductase	N	N	N	N	N	N
	WP_005411610.1	phosphopantetheine-binding protein	N	N	N	N	N	N
	WP_025873777.1	response regulator	N	N	N	N	N	N
NZ_CP040429.1	WP_025873780.1	EamA family transporter	N	N	N	N	N	N
	WP_025873781.1	DMT family transporter	N	N	N	N	N	N
	WP_025873919.1	helix-turn-helix transcriptional regulator	N	N	N	N	N	N
	WP_025873926.	GNAT family N-acetyltransferase	N	N	N	N	N	N

1							
WP_025874050.1	response regulator transcription factor	N	N	N	N	N	N
WP_025874094.1	LysR family transcriptional regulator	N	N	N	N	N	N
WP_025874351.1	nitrate/nitrite transporter	N	N	N	N	N	N
WP_025874361.1	Lrp/AsnC family transcriptional regulator	N	N	N	N	N	N
WP_025874382.1	biopolymer transporter ExbD	N	N	N	N	N	N
WP_025874390.1	LysR family transcriptional regulator	N	N	N	N	N	N
WP_025874693.1	response regulator transcription factor	N	N	N	N	N	N
WP_025874806.1	Cu(I)-responsive transcriptional regulator	N	N	N	N	N	N
WP_025875910.1	SDR family oxidoreductase	N	N	N	N	N	N
WP_025875914.1	Lrp/AsnC family transcriptional regulator	N	N	N	N	N	N
WP_025875920.1	Rid family hydrolase	N	N	N	N	N	N
WP_025876304.1	phosphotyrosine protein phosphatase	N	N	N	N	N	N
WP_025876351.	alpha/beta hydrolase	N	N	N	N	N	N

1							
WP_025876357.1	TetR/AcrR family transcriptional regulator	N	N	N	N	N	N
WP_025876674.1	UDP-forming cellulose synthase catalytic subunit	N	N	N	N	N	N
WP_025877085.1	VOC family protein	N	N	N	N	N	N
WP_025877180.1	LysR family transcriptional regulator	N	N	N	N	N	N
WP_025877422.1	cytochrome o ubiquinol oxidase subunit IV	N	N	N	N	N	N
WP_025878014.1	acyl carrier protein	N	N	N	N	N	N
WP_025878439.1	winged helix-turn-helix domain-containing protein	N	N	N	N	N	N
WP_025878554.1	MgtC/SapB family protein	N	N	N	N	N	N
WP_025878601.1	hypothetical protein	N	N	N	N	N	N
WP_025878951.1	transcriptional regulator BetI	N	N	N	N	N	N
WP_025879232.1	RidA family protein	N	N	N	N	N	N
WP_032951763.1	O-methyltransferase	N	N	N	N	N	N
WP_032974655.	aldehyde dehydrogenase iron-sulfur	N	N	N	N	N	N

1	subunit PaoA						
WP_032974735.1	AraC family transcriptional regulator	N	N	N	N	N	N
WP_032976023.1	aminotransferase class III-fold pyridoxal phosphate-dependent enzyme	N	N	N	N	N	N
WP_032976705.1	VOC family protein	N	N	N	N	N	N
WP_032976930.1	VWA domain-containing protein	N	N	N	N	N	N
WP_033830819.1	alpha/beta hydrolase	N	N	N	N	N	N
WP_033831116.1	LysR family transcriptional regulator	N	N	N	N	N	N
WP_033831150.1	cytochrome c	N	N	N	N	N	N
WP_033831258.1	type II secretion system major pseudopilin GspG	N	N	N	N	N	N
WP_033831264.1	AzlD family protein	N	N	N	N	N	N
WP_033832104.1	GNAT family N-acetyltransferase	N	N	N	N	N	N
WP_033832295.1	MBL fold metallo-hydrolase	N	N	N	N	N	N
WP_049464392.1	ABC transporter ATP-binding protein	N	N	N	N	N	N
WP_049466772.	cupin domain-containing protein	N	N	N	N	N	N

1							
WP_051585035.1	HAD family hydrolase	N	N	N	N	N	N
WP_051585047.1	LysR family transcriptional regulator [Stenotrophomonas sp. RIT309]	N	N	N	N	N	N
WP_059064113.1	BCCT family transporter	N	N	N	N	N	N
WP_059064684.1	sugar phosphate isomerase/epimerase family protein	N	N	N	N	N	N
WP_080148505.1	response regulator transcription factor	N	N	N	N	N	N
WP_080278337.1	cytochrome o ubiquinol oxidase subunit III	N	N	N	N	N	N
WP_088100665.1	NAD(P)H-dependent oxidoreductase	N	N	N	N	N	N
WP_099784100.1	carboxymuconolactone decarboxylase family protein	N	N	N	N	N	N
WP_099785660.1	PDDEXK nuclease domain-containing protein	N	N	N	N	N	N
WP_099843770.1	carboxymuconolactone decarboxylase family protein	N	N	N	N	N	N
WP_100436177.1	LysR family transcriptional regulator	N	N	N	N	N	N
WP_105164194.1	respiratory nitrate reductase subunit gamma [Stenotrophomonas sp. MYb57]	N	N	N	N	N	N
WP_105164648.	transcriptional regulator	N	N	N	N	N	N

1							
WP_134832138.1	SDR family oxidoreductase	N	N	N	N	N	N
WP_154879023.1	nuclear transport factor 2 family protein	N	N	N	N	N	N
WP_154879025.1	TetR/AcrR family transcriptional regulator	N	N	N	N	N	N
WP_154879035.1	VOC family protein	N	N	N	N	N	N
WP_154879108.1	tail assembly protein	NZ_CP040429.1-1	100	N	N	N	N
WP_154879125.1	type II toxin-antitoxin system RelE/ParE family toxin	N	N	N	N	N	N
WP_154879222.1	RNA 2'-phosphotransferase	N	N	N	N	N	N
WP_154879478.1	flavin reductase family protein	N	N	N	N	N	N
WP_154879677.1	GyrI-like domain-containing protein	N	N	N	N	N	N
WP_154879871.1	glycoside hydrolase family 104 protein	N	N	N	N	N	N
WP_154879878.1	DUF1064 domain-containing protein	N	N	N	N	N	N
WP_154879912.1	tail assembly protein	NZ_CP040429.1-2	100	N	N	N	N
WP_154880112.	nucleotide pyrophosphohydrolase	N	N	N	N	N	N

1							
WP_154880114.1	enoyl-CoA hydratase/isomerase family protein	N	N	N	N	N	N
WP_154880116.1	helix-turn-helix domain-containing protein	N	N	N	N	N	N
WP_154880148.1	DNA-binding protein	N	N	N	N	N	N
WP_154880154.1	TetR family transcriptional regulator	N	N	N	N	N	N
WP_154880348.1	aldo/keto reductase	N	N	N	N	N	N
WP_154880350.1	flavodoxin	N	N	N	N	N	N
WP_154880354.1	alpha/beta hydrolase	N	N	N	N	N	N
WP_154880356.1	alpha/beta fold hydrolase	N	N	N	N	N	N
WP_154880368.1	MFS transporter	N	N	N	N	N	N
WP_154880371.1	alpha/beta hydrolase	N	N	N	N	N	N
WP_154880409.1	NAD-dependent epimerase/dehydratase family protein	N	N	N	N	N	N
WP_154880411.1	type 1 glutamine amidotransferase domain-containing protein	N	N	N	N	N	N
WP_154880413.	TetR family transcriptional regulator	N	N	N	N	N	N

1							
WP_154880417.1	NADP-dependent oxidoreductase	N	N	N	N	N	N
WP_154880424.1	GMC family oxidoreductase	N	N	N	N	N	N
WP_154880482.1	AAA family ATPase	N	N	N	N	N	N
WP_154880537.1	TIGR03571 family LLM class oxidoreductase	N	N	N	N	N	N
WP_154880539.1	cyclase family protein	N	N	N	N	N	N
WP_154880543.1	aldo/keto reductase	N	N	N	N	N	N
WP_154880561.1	isochorismatase family protein	N	N	N	N	N	N
WP_154880610.1	NarK family nitrate/nitrite MFS transporter	N	N	N	N	N	N
WP_154880629.1	helix-turn-helix transcriptional regulator	N	N	N	N	N	N
WP_154880678.1	sigma-70 family RNA polymerase sigma factor	N	N	N	N	N	N
WP_154880797.1	FAD-dependent monooxygenase	N	N	N	N	N	N
WP_154881006.1	NADAR family protein	N	N	N	N	N	N
WP_154881015.	helix-turn-helix transcriptional regulator	N	N	N	N	N	N

1							
WP_154881078.1	LTA synthase family protein	N	N	N	N	N	N
WP_154881103.1	CmlA/FloR family chloramphenicol efflux MFS transporter	N	N	N	N	N	N
WP_154881105.1	Ohr family peroxiredoxin	N	N	N	N	N	N
WP_154881106.1	SDR family oxidoreductase	N	N	N	N	N	N
WP_154881108.1	RNA polymerase sigma-70 factor	N	N	N	N	N	N
WP_154881112.1	helix-turn-helix domain-containing protein [Stenotrophomonas sp. DR822]	N	N	N	N	N	N
WP_200866840.1	VOC family protein	N	N	N	N	N	N
WP_229655596.1	MFS transporter	N	N	N	N	N	N
WP_229655604.1	VOC family protein	N	N	N	N	N	N
WP_229655647.1	AMP-binding protein	N	N	N	N	N	N
WP_229655684.1	AraC family transcriptional regulator	N	N	N	N	N	N
WP_229655685.1	LLM class flavin-dependent oxidoreductase	N	N	N	N	N	N
WP_229655698.	very short patch repair endonuclease	N	N	N	N	N	N

	1							
	WP_005408332.1	GPW/gp25 family protein	N	N	N	N	N	N
	WP_005409664.1	heat resistance protein YfdX1	NZ_CP060026.1-2	100	N	N	NZ_CP060025.1-2, NZ_CP060027.1-4, NZ_CP102942.1-4, NZ_CP098483.1-4	100
	WP_005409665.1	small heat shock protein sHSP20-GI	N	N	N	N	N	N
	WP_016241869.1	hypothetical protein	N	N	N	N	N	N
	WP_021567627.1	heat resistance protein YfdX2	N	N	N	N	N	N
NZ_CP040430.1	WP_032961836.1	phage terminase large subunit	NZ_CM001824.1-1, NZ_CP014014.1-2, NZ_LR134324.1-1	100	NZ_AP021908.1-1, NZ_CP040431.1-2, NZ_CP040439.1-4, NZ_CP043578.1-1, NZ_CP056088.1-2, NZ_CP060027.1-3, NZ_LR134324.1-3, NZ_CP090418.1-2, NZ_CP104863.1-3, NZ_CP102942.1-3, NZ_CP098483.1-2	100	NZ_CP040430.1-2	100
	WP_033833415.1	3'-5' exonuclease	N	N	N	N	N	N

WP_033981790.1	heat resistance membrane protein HdeD-GI	N	N	N	N	N	N
WP_034000762.1	heat resistance protein YfdX2	N	N	N	N	N	N
WP_049399110.1	hypothetical protein	N	N	N	N	N	N
WP_049420578.1	helix-turn-helix domain-containing protein	N	N	N	N	N	N
WP_049420580.1	Hsp20/alpha crystallin family protein	N	N	N	N	N	N
WP_049429822.1	efflux RND transporter periplasmic adaptor subunit	N	N	N	N	N	N
WP_049429823.1	hypothetical protein	N	N	N	N	N	N
WP_049441491.1	cardiolipin synthase	N	N	N	N	N	N
WP_049449698.1	GNAT family N-acetyltransferase	N	N	N	N	N	N
WP_065426719.1	hypothetical protein	N	N	N	N	N	N
WP_087786704.1	ISL3 family transposase	N	N	N	N	N	N
WP_088429185.1	hypothetical protein	N	N	N	N	N	N
WP_088429662.1	alpha/beta hydrolase	N	N	N	N	N	N

WP_088430361.1	DJ-1/PfpI family protein	N	N	N	N	N	N
WP_088431037.1	hypothetical protein	N	N	N	N	N	N
WP_099526933.1	IS3-like element ISPa20 family transposase	N	N	N	N	N	N
WP_099527372.1	zinc metalloprotease HtpX	N	N	N	N	N	N
WP_099527865.1	small heat shock protein sHSP20-GI	N	N	N	N	N	N
WP_099540299.1	heat resistance membrane protein HdeD-GI	N	N	N	N	N	N
WP_154267672.1	McrC family protein	N	N	N	N	N	N
WP_154267703.1	DNA adenine methylase	N	N	N	N	N	N
WP_154267714.1	Abi family protein	N	N	N	N	N	N
WP_154267833.1	nucleotidyltransferase	N	N	N	N	N	N
WP_154267835.1	Mov34/MPN/PAD-1 family protein	N	N	N	N	N	N
WP_154267873.1	heat resistance system K+/H+ antiporter KefB-GI	N	N	N	N	N	N
WP_154267874.1	hypothetical protein	N	N	N	N	N	N

	WP_154267876.1	heat resistance protein YfdX1	N	N	N	N	N	N
	WP_154267951.1	ISL3 family transposase	N	N	N	N	N	N
	WP_196757859.1	hypothetical protein	N	N	N	N	N	N
	WP_217898830.1	amidase family protein	N	N	N	N	N	N
	WP_217898832.1	DNA methyltransferase	N	N	N	N	N	N
	WP_223846485.1	HNH endonuclease signature motif containing protein	N	N	N	N	N	N
	WP_229297634.1	SDR family oxidoreductase	N	N	N	N	N	N
	WP_229297701.1	site-specific DNA-methyltransferase	N	N	N	N	N	N
	WP_229297703.1	nuclear transport factor 2 family protein	N	N	N	N	N	N
NZ_CP040431.1	WP_005408332.1	GPW/gp25 family protein	N	N	N	N	N	N
	WP_005408904.1	nuclear transport factor 2 family protein	N	N	N	N	N	N
	WP_005409644.1	hypothetical protein	N	N	N	N	N	N
	WP_005409655.1	IS110 family transposase	N	N	NZ_CP088244.1-3	100	NZ_CP060025.1-2, NZ_CP060027.1-4,	100

						NZ_CP098483.1-4	
WP_005409659.1	heat resistance system K+/H+ antiporter KefB-GI	NZ_CP060026.1-2	100	NZ_CP040440.1-7, NZ_CP088244.1-3	100	NZ_CP060025.1-2, NZ_CP060027.1-4, NZ_CP098483.1-4	100
WP_005409661.1	hypothetical protein	NZ_CP060026.1-2	100	NZ_CP040440.1-7	100	NZ_CP060025.1-2, NZ_CP060027.1-4, NZ_CP098483.1-4	100
WP_005409662.1	HdeD family acid-resistance protein	NZ_CP060026.1-2	100	NZ_CP040440.1-7	100	NZ_CP060025.1-2, NZ_CP060027.1-4, NZ_CP098483.1-4	100
WP_005409663.1	heat resistance protein YfdX2	NZ_CP060026.1-2	100	NZ_CP040440.1-7	100	NZ_CP060025.1-2, NZ_CP060027.1-4, NZ_CP098483.1-4, NZ_CP102942.1-4	100
WP_005409664.1	heat resistance protein YfdX1	NZ_CP060026.1-2	100	NZ_CP040440.1-7	100	NZ_CP060025.1-2, NZ_CP060027.1-4, NZ_CP102942.1-4, NZ_CP098483.1-4	100
WP_005409665.1	small heat shock protein sHSP20-GI	N	N	N	N	N	N
WP_005409667.1	cardiolipin synthase	N	N	N	N	N	N
WP_005409669.1	Hsp20/alpha crystallin family protein	NZ_CP060026.1-2	100	NZ_CP040431.1-4, NZ_CP040433.1-2, NZ_CP040440.1-7, NZ_CP088244.1-4	100	NZ_CP060025.1-2, NZ_CP060027.1-4, NZ_CP102942.1-4, NZ_CP098483.1-4	100

WP_012479074.1	SMR family transporter	N	N	N	N	N	N
WP_012479075.1	TIM barrel protein	N	N	N	N	N	N
WP_012479077.1	FAD-dependent oxidoreductase	N	N	N	N	N	N
WP_012479078.1	glycosyltransferase	N	N	N	N	N	N
WP_024957346.1	glycosyltransferase family 2 protein	N	N	N	N	N	N
WP_032960048.1	site-specific integrase	N	N	N	N	N	N
WP_032961707.1	helix-turn-helix domain-containing protein	N	N	N	N	N	N
WP_032961714.1	heat resistance protein PsiE-GI	N	N	N	N	N	N
WP_032961836.1	phage terminase large subunit	NZ_CM001824.1-1, NZ_CP014014.1-2, NZ_LR134324.1-1	100	NZ_AP021908.1-1, NZ_CP040431.1-2, NZ_CP040439.1-4, NZ_CP043578.1-1, NZ_CP056088.1-2, NZ_CP060027.1-3, NZ_LR134324.1-3, NZ_CP090418.1-2, NZ_CP104863.1-3, NZ_CP102942.1-3,	100	NZ_CP040430.1-2	100

				NZ_CP098483.1-2			
WP_032965863.1	hypothetical protein	N	N	N	N	N	N
WP_049449467.1	site-specific integrase	NZ_CP088244.1-1, NZ_CP098483.1-1	100	N	N	NZ_CP040433.1-3, NZ_CP060025.1-3	100
WP_061478821.1	DNA cytosine methyltransferase	NZ_CP040431.1-1, NZ_CP060027.1-1	100	N	N	N	N
WP_065426719.1	hypothetical protein	N	N	N	N	N	N
WP_076738974.1	DUF1993 domain-containing protein	N	N	N	N	N	N
WP_154329194.1	AIPR family protein	N	N	N	N	N	N
WP_154329196.1	Z1 domain-containing protein	N	N	N	N	N	N
WP_154329370.1	ImmA/IrrE family metallo-endopeptidase	N	N	N	N	N	N
WP_154329545.1	N-6 DNA methylase	N	N	N	N	N	N
WP_185805886.1	metallohydrolase	N	N	N	N	N	N
WP_197599535.1	NAD(P)-dependent oxidoreductase	N	N	N	N	N	N
WP_227860296.1	ATP-binding protein	N	N	N	N	N	N
WP_229298564.	Mov34/MPN/PAD-1 family protein	N	N	N	N	N	N

	1							
	WP_229298666.1	SDR family oxidoreductase	N	N	N	N	N	N
	WP_229298737.1	very short patch repair endonuclease	N	N	N	N	N	N
	WP_229298741.1	TatD family hydrolase	N	N	N	N	N	N
	WP_000539741.1	multidrug efflux SMR transporter	N	N	N	N	N	N
	WP_001062689.1	site-specific integrase	N	N	N	N	N	N
	WP_001247107.1	helix-turn-helix domain-containing protein	N	N	N	N	N	N
	WP_001273857.1	DUF736 domain-containing protein	N	N	N	N	N	N
NZ_CP040432.1	WP_003092330.1	DNA repair protein RadC	N	N	N	N	N	N
	WP_003100847.1	recombinase family protein	N	N	N	N	N	N
	WP_003100872.1	DUF86 domain-containing protein	N	N	N	N	N	N
	WP_003108276.1	AbrB/MazE/SpoVT family DNA-binding domain-containing protein	N	N	N	N	N	N
	WP_003159185.1	recombinase family protein	N	N	N	N	N	N
	WP_003159186.	type II toxin-antitoxin system VapC	N	N	N	N	N	N

1	family toxin						
WP_004135208.1	LysR substrate-binding domain-containing protein	N	N	N	N	N	N
WP_004135210.1	carboxymuconolactone decarboxylase family protein	N	N	N	N	N	N
WP_004135212.1	tautomerase family protein	N	N	N	N	N	N
WP_004141632.1	NAD-dependent epimerase/dehydratase family protein	N	N	N	N	N	N
WP_004143363.1	AAA domain-containing protein	N	N	N	N	N	N
WP_004146420.1	BREX protein BrxB domain-containing protein	N	N	N	N	N	N
WP_004146422.1	BREX system P-loop protein BrxC	N	N	N	N	N	N
WP_004146424.1	hypothetical protein	N	N	N	N	N	N
WP_004146476.1	oleate hydratase	N	N	N	N	N	N
WP_004146585.1	tyrosine-type recombinase/integrase	N	N	N	N	N	N
WP_004146590.1	cyclophilin-like fold protein	N	N	N	N	N	N
WP_004146767.1	VWA domain-containing protein	N	N	N	N	NZ_CP040432.1-4	100
WP_004146768.	DUF475 domain-containing protein	N	N	N	N	NZ_CP040432.1-4	100

1							
WP_004146769.1	TerD family protein	N	N	N	N	NZ_CP040432.1-4	100
WP_004146770.1	TerD family protein	N	N	N	N	NZ_CP040432.1-4	
WP_004146832.1	cysteine protease StiP family protein	N	N	N	N	N	N
WP_004147795.1	helix-turn-helix domain-containing GNAT family N-acetyltransferase	N	N	N	N	N	N
WP_004147834.1	alpha/beta hydrolase	N	N	N	N	N	N
WP_004152370.1	YoeB-YefM toxin-antitoxin system antitoxin YefM	N	N	N	N	N	N
WP_004152375.1	conjugal transfer protein TraG N-terminal domain-containing protein	N	N	N	N	N	N
WP_004152410.1	integrase domain-containing protein	N	N	N	N	N	N
WP_005416651.1	RebB family R body protein	N	N	N	N	N	N
WP_006361971.1	DEAD/DEAH box helicase family protein	N	N	N	N	N	N
WP_006361972.1	N-6 DNA methylase	N	N	N	N	N	N
WP_006362647.1	LysR substrate-binding domain-containing protein	N	N	N	N	N	N
WP_006365086.	alpha/beta fold hydrolase	N	N	N	N	N	N

1							
WP_006367009.1	NAD(P)H-dependent oxidoreductase	N	N	N	N	N	N
WP_006367022.1	pirin family protein	N	N	N	N	N	N
WP_006367410.1	MFS transporter	N	N	N	N	N	N
WP_006367411.1	aldo/keto reductase	N	N	N	N	N	N
WP_006368766.1	RebB family R body protein	N	N	N	N	N	N
WP_006368769.1	RebB family R body protein	N	N	N	N	N	N
WP_006368771.1	RebB family R body protein	N	N	N	N	N	N
WP_006375482.1	DMT family transporter	N	N	N	N	N	N
WP_006375717.1	site-specific DNA-methyltransferase	N	N	N	N	N	N
WP_006378795.1	P-type conjugative transfer protein TrbL	N	N	N	N	N	N
WP_006378800.1	P-type conjugative transfer protein TrbJ	N	N	N	N	N	N
WP_006378802.1	VirB3 family type IV secretion system protein	N	N	N	N	N	N
WP_006378803.	TrbC/VirB2 family protein	N	N	N	N	N	N

1							
WP_006378865.1	CopG family transcriptional regulator	N	N	N	N	N	N
WP_006378991.1	LysR family transcriptional regulator	N	N	N	N	N	N
WP_006378994.1	hypothetical protein	N	N	N	N	N	N
WP_006378999.1	LysR family transcriptional regulator	N	N	N	N	N	N
WP_006379345.1	S26 family signal peptidase	N	N	N	N	N	N
WP_006379346.1	DUF2840 domain-containing protein	N	N	N	N	N	N
WP_006379424.1	DUF2285 domain-containing protein	N	N	N	N	N	N
WP_006379425.1	helix-turn-helix transcriptional regulator	N	N	N	N	N	N
WP_006379606.1	ParB/RepB/Spo0J family partition protein	N	N	N	N	N	N
WP_006387162.1	pilin	N	N	N	N	N	N
WP_006397453.1	RhuM family protein	N	N	N	N	N	N
WP_006399830.1	cytochrome o ubiquinol oxidase subunit IV	N	N	N	N	N	N
WP_012510145.	VOC family protein	N	N	N	N	N	N

1							
WP_012511031.1	LysR family transcriptional regulator	N	N	N	N	N	N
WP_012511690.1	metalloregulator ArsR/SmtB family transcription factor	N	N	N	N	N	N
WP_016487820.1	TrbI/VirB10 family protein	N	N	N	N	N	N
WP_016487821.1	P-type conjugative transfer protein TrbG	N	N	N	N	N	N
WP_016487824.1	EexN family lipoprotein	N	N	N	N	N	N
WP_016487838.1	DUF2958 domain-containing protein	N	N	N	N	N	N
WP_016487839.1	hypothetical protein	N	N	N	N	N	N
WP_022652187.1	hypothetical protein	N	N	N	N	N	N
WP_023093371.1	ATP-binding cassette domain-containing protein	N	N	N	N	N	N
WP_032968948.1	BREX system Lon protease-like protein BrxL	N	N	N	N	N	N
WP_032969047.1	TerD family protein	N	N	N	N	N	N
WP_032969062.1	helicase-related protein	N	N	N	N	N	N
WP_032969133.	GNAT family N-acetyltransferase	N	N	N	N	N	N

1							
WP_032969198.1	isocitrate lyase/PEP mutase family protein	N	N	N	N	N	N
WP_032969301.1	MFS transporter	N	N	N	N	N	N
WP_032971627.1	ABC transporter permease	N	N	N	N	N	N
WP_032972196.1	GrpB family protein	N	N	N	N	N	N
WP_087986239.1	IS3-like element ISStma9 family transposase	N	N	N	N	NZ_CP040432.1-4	100
WP_108400229.1	DUF86 domain-containing protein	N	N	N	N	N	N
WP_108400230.1	nucleotidyltransferase family protein	N	N	N	N	N	N
WP_141650943.1	TIGR00266 family protein	N	N	N	N	N	N
WP_154354630.1	VWA domain-containing protein	N	N	N	N	NZ_CP040432.1-4	100
WP_154354669.1	Tn3-like element ISPa38 family transposase	N	N	N	N	N	N
WP_154354670.1	recombinase family protein	N	N	N	N	N	N
WP_154354775.1	XRE family transcriptional regulator	N	N	N	N	N	N
WP_218054225.	lipopolysaccharide kinase InaA family	N	N	N	N	NZ_CP040432.1-4	100

	1	protein					
	WP_218054232.1	methyl-accepting chemotaxis protein	N	N	N	N	N	N
	WP_218054235.1	TolC family protein	N	N	N	N	N	N
	WP_223224815.1	WYL domain-containing protein	N	N	N	N	N	N
	WP_229298186.1	ABC transporter permease	N	N	N	N	N	N
	WP_229298187.1	ABC transporter ATP-binding protein	N	N	N	N	N	N
	WP_003140997.1	TIGR03758 family integrating conjugative element protein	N	N	N	N	N	N
	WP_003141001.1	helix-turn-helix domain-containing protein	N	N	N	N	N	N
	WP_003141015.1	integrating conjugative element protein	N	N	N	N	N	N
NZ_CP040433.1	WP_003141051.1	hypothetical protein	N	N	N	N	N	N
	WP_003292115.1	hypothetical protein	N	N	N	N	N	N
	WP_004362284.1	single-stranded DNA-binding protein	N	N	N	N	N	N
	WP_005409655.1	IS110 family transposase	N	N	NZ_CP088244.1-3	100	NZ_CP060025.1-2, NZ_CP060027.1-4, NZ_CP098483.1-4	100

WP_005409659.1	heat resistance system K+/H+ antiporter KefB-GI	NZ_CP060026.1-2	100	NZ_CP040440.1-7, NZ_CP088244.1-3	100	NZ_CP060025.1-2, NZ_CP060027.1-4, NZ_CP098483.1-4	100
WP_005409662.1	HdeD family acid-resistance protein	NZ_CP060026.1-2	100	N	N	NZ_CP060025.1-2, NZ_CP060027.1-4, NZ_CP098483.1-4	100
WP_005409663.1	heat resistance protein YfdX2	NZ_CP060026.1-2	100	N	N	NZ_CP060025.1-2, NZ_CP060027.1-4, NZ_CP098483.1-4, NZ_CP102942.1-4	100
WP_005409664.1	heat resistance protein YfdX1	NZ_CP060026.1-2	100	N	N	NZ_CP060025.1-2, NZ_CP060027.1-4, NZ_CP102942.1-4, NZ_CP098483.1-4	100
WP_005409665.1	small heat shock protein sHSP20-GI	N	N	N	N	N	N
WP_005409667.1	cardiolipin synthase	N	N	N	N	N	N
WP_005409669.1	Hsp20/alpha crystallin family protein	NZ_CP060026.1-2	100	NZ_CP040431.1-4, NZ_CP040433.1-2, NZ_CP040440.1-7, NZ_CP088244.1-4	100	NZ_CP060025.1-2, NZ_CP060027.1-4, NZ_CP102942.1-4, NZ_CP098483.1-4	100
WP_005414983.1	ATP-binding protein	N	N	N	N	N	N
WP_005414984.1	MFS transporter	N	N	N	N	N	N

WP_009618209.1	type II toxin-antitoxin system RelE/ParE family toxin	N	N	N	N	N	N
WP_009618210.1	type II toxin-antitoxin system RelB/DinJ family antitoxin	N	N	N	N	N	N
WP_011829925.1	AlpA family transcriptional regulator	N	N	N	N	N	N
WP_012478642.1	UvrD-helicase domain-containing protein	N	N	N	N	N	N
WP_013982110.1	ParA family protein	N	N	N	N	N	N
WP_013982111.1	type II toxin-antitoxin system HicA family toxin	N	N	N	N	N	N
WP_013982112.1	ParB family protein	N	N	N	N	N	N
WP_013982130.1	TIGR03759 family integrating conjugative element protein	N	N	N	N	N	N
WP_013982148.1	DUF3742 family protein	N	N	N	N	N	N
WP_017244834.1	TIGR03750 family conjugal transfer protein	N	N	N	N	N	N
WP_019484549.1	TIGR03745 family integrating conjugative element membrane protein	N	N	N	N	N	N
WP_019484551.1	TIGR03746 family integrating conjugative element protein	N	N	N	N	N	N
WP_019751268.1	STY4534 family ICE replication protein	N	N	N	N	N	N

WP_023083166.1	TIGR03751 family conjugal transfer lipoprotein	N	N	N	N	N	N
WP_023083168.1	TIGR03749 family integrating conjugative element protein	N	N	N	N	N	N
WP_023121122.1	type IV toxin-antitoxin system AbiEi family antitoxin domain-containing protein	N	N	N	N	N	N
WP_023657702.1	tyrosine-type recombinase/integrase	N	N	N	N	N	N
WP_023912240.1	DUF2857 domain-containing protein	N	N	N	N	N	N
WP_023912242.1	STY4528 family pathogenicity island replication protein	N	N	N	N	N	N
WP_023912244.1	TIGR03761 family integrating conjugative element protein	N	N	N	N	N	N
WP_023912246.1	DUF3158 family protein	N	N	N	N	N	N
WP_025297717.1	hypothetical protein	N	N	N	N	N	N
WP_031755001.1	DUF3275 family protein	N	N	N	N	N	N
WP_033873014.1	integrating conjugative element protein	N	N	N	N	N	N
WP_034021462.1	hypothetical protein	N	N	N	N	N	N
WP_043104882.	PilL N-terminal domain-containing	N	N	N	N	N	N

1	protein						
WP_045786220.1	DNA repair protein RadC	N	N	N	N	N	N
WP_049429336.1	BsuBI/PstI family type II restriction endonuclease	N	N	N	N	N	N
WP_049439817.1	type ISP restriction/modification enzyme	N	N	N	N	N	N
WP_061479192.1	nuclear transport factor 2 family protein	N	N	N	N	N	N
WP_061959005.1	thioredoxin domain-containing protein	N	N	N	N	N	N
WP_061959006.1	TIGR03757 family integrating conjugative element protein	N	N	N	N	N	N
WP_061960173.1	anti-sigma factor	N	N	N	N	N	N
WP_061960174.1	beta-propeller fold lactonase family protein	N	N	N	N	N	N
WP_062842676.1	hypothetical protein	N	N	N	N	N	N
WP_065178056.1	type II toxin-antitoxin system HigB family toxin	N	N	N	N	N	N
WP_085371671.1	DUF2958 domain-containing protein	N	N	N	N	N	N
WP_114618409.1	heat resistance protein PsiE-GI	N	N	N	N	N	N
WP_125866636.	ABC transporter substrate-binding	N	N	N	N	N	N

1	protein						
WP_128989844.1	DNA methyltransferase	N	N	N	N	N	N
WP_134969248.1	helix-turn-helix domain-containing GNAT family N-acetyltransferase	N	N	N	N	N	N
WP_134970267.1	sigma-54 dependent transcriptional regulator	N	N	N	N	N	N
WP_154350191.1	ABC transporter ATP-binding protein	N	N	N	N	N	N
WP_154350319.1	ATP-binding protein	N	N	N	N	N	N
WP_154350320.1	AAA family ATPase	N	N	N	N	N	N
WP_154350512.1	type II toxin-antitoxin system HipA family toxin	N	N	N	N	N	N
WP_154350595.1	AlpA family phage regulatory protein	N	N	N	N	N	N
WP_154350600.1	hypothetical protein	N	N	N	N	N	N
WP_154350601.1	DUF736 domain-containing protein	N	N	N	N	N	N
WP_154350604.1	helix-turn-helix transcriptional regulator	N	N	N	N	N	N
WP_154350605.1	DUF2285 domain-containing protein	N	N	N	N	N	N
WP_154350608.	chromosome partitioning protein ParB	N	N	N	N	N	N

1							
WP_154350609.1	DUF2840 domain-containing protein	N	N	N	N	N	N
WP_154350610.1	S26 family signal peptidase	N	N	N	N	N	N
WP_154350612.1	ATP-binding protein	N	N	N	N	N	N
WP_154350613.1	response regulator transcription factor	N	N	N	N	N	N
WP_154350618.1	LysR substrate-binding domain-containing protein	N	N	N	N	N	N
WP_154350619.1	entry exclusion lipoprotein TrbK	N	N	N	N	N	N
WP_154350623.1	TrbC/VirB2 family protein	N	N	N	N	N	N
WP_154350625.1	P-type conjugative transfer protein TrbJ	N	N	N	N	N	N
WP_154350626.1	hypothetical protein	N	N	N	N	N	N
WP_154350631.1	DUF3320 domain-containing protein	N	N	N	N	N	N
WP_154350632.1	type II toxin-antitoxin system HipA family toxin	N	N	N	N	N	N
WP_154350780.1	DnaJ C-terminal domain-containing protein	N	N	N	N	N	N
WP_154350799.	fimbria/pilus chaperone family protein	N	N	N	N	N	N

1							
WP_154350826.1	multidrug efflux RND transporter permease subunit	N	N	N	N	N	N
WP_154350827.1	MFS transporter	N	N	N	N	N	N
WP_154351305.1	GTPase	N	N	N	N	N	N
WP_154351306.1	DUF3085 domain-containing protein	N	N	N	N	N	N
WP_154351307.1	hypothetical protein	N	N	N	N	N	N
WP_154351308.1	DUF6094 domain-containing protein	N	N	N	N	N	N
WP_154351309.1	hypothetical protein	N	N	N	N	N	N
WP_154351310.1	lytic transglycosylase domain-containing protein	N	N	N	N	N	N
WP_154351312.1	TIGR03747 family integrating conjugative element membrane protein	N	N	N	N	N	N
WP_154351313.1	molybdopterin-dependent oxidoreductase	N	N	N	N	N	N
WP_154351314.1	nuclear transport factor 2 family protein	N	N	N	N	N	N
WP_154351315.1	RAQPRD family integrative conjugative element protein	N	N	N	N	N	N
WP_154351316.	TIGR03752 family integrating	N	N	N	N	N	N

1	conjugative element protein						
WP_154351319.1	conjugal transfer protein TraG N-terminal domain-containing protein	N	N	N	N	N	N
WP_154351320.1	nucleotidyl transferase AbiEii/AbiGii toxin family protein	N	N	N	N	N	N
WP_154351321.1	MobH family relaxase	N	N	N	N	N	N
WP_154351322.1	substrate-binding domain-containing protein	N	N	N	N	N	N
WP_154351554.1	hypothetical protein	N	N	N	N	N	N
WP_154351717.1	LysR substrate-binding domain-containing protein	N	N	N	N	N	N
WP_185807360.1	very short patch repair endonuclease	N	N	N	N	N	N
WP_185807475.1	LysR family transcriptional regulator	N	N	N	N	N	N
WP_185807476.1	LysR family transcriptional regulator	N	N	N	N	N	N
WP_185807478.1	RNA polymerase sigma factor	N	N	N	N	N	N
WP_197697522.1	YbhB/YbcL family Raf kinase inhibitor-like protein	N	N	N	N	N	N
WP_200899071.1	type II toxin-antitoxin system YhaV family toxin	N	N	N	N	N	N
WP_221892504.	ISL3 family transposase	N	N	N	N	N	N

	1							
	WP_221892513.1	efflux RND transporter periplasmic adaptor subunit	N	N	N	N	N	N
	WP_227200205.1	helix-turn-helix transcriptional regulator	N	N	N	N	N	N
	WP_229298442.1	efflux RND transporter periplasmic adaptor subunit	N	N	N	N	N	N
	WP_229298481.1	SNF2-related protein	N	N	N	N	N	N
	WP_229298486.1	chromate efflux transporter	N	N	N	N	N	N
	WP_229298541.1	efflux RND transporter permease subunit	N	N	N	N	N	N
	WP_229298548.1	bestrophin family protein	N	N	N	N	N	N
	WP_229298563.1	TatD family hydrolase	N	N	N	N	N	N
	WP_229298564.1	Mov34/MPN/PAD-1 family protein	N	N	N	N	N	N
	WP_229298574.1	DsbA family protein	N	N	N	N	N	N
	WP_003131974.1	mercuric ion transporter MerT	N	N	N	N	N	N
NZ_CP040434.1	WP_003131987.1	mercury resistance system periplasmic binding protein MerP	N	N	N	N	N	N
	WP_003156770.	mercury(II) reductase	N	N	N	N	N	N

1							
WP_004350822.1	LysR substrate-binding domain-containing protein	N	N	N	N	N	N
WP_005413387.1	Hg(II)-responsive transcriptional regulator	N	N	N	N	N	N
WP_005413400.1	Tn3-like element TnAs1 family transposase	N	N	N	N	NZ_CP043578.1-2	100
WP_005416649.1	RebB family R body protein	N	N	N	N	N	N
WP_005416651.1	RebB family R body protein	N	N	N	N	N	N
WP_006399830.1	cytochrome o ubiquinol oxidase subunit IV	N	N	N	N	N	N
WP_006446865.1	RebB family R body protein	N	N	N	N	N	N
WP_008264906.1	HupE/UreJ family protein	N	N	N	N	N	N
WP_009586987.1	permease	N	N	N	N	N	N
WP_010921730.1	recombinase family protein	N	N	N	N	N	N
WP_011716362.1	P-type conjugative transfer protein TrbJ	N	N	N	N	N	N
WP_011716365.1	conjugal transfer protein TrbF	N	N	N	N	N	N
WP_021204031.	VirB3 family type IV secretion system	N	N	N	N	N	N

1	protein						
WP_021204032.1	CopG family transcriptional regulator	N	N	N	N	N	N
WP_021204041.1	LLM class flavin-dependent oxidoreductase	N	N	N	N	N	N
WP_022960600.1	TrbC/VirB2 family protein	N	N	N	N	N	N
WP_022960607.1	ISL3 family transposase	N	N	N	N	N	N
WP_023047514.1	TetR/AcrR family transcriptional regulator	N	N	N	N	N	N
WP_024718240.1	EexN family lipoprotein	N	N	N	N	N	N
WP_032962419.1	SDR family oxidoreductase	N	N	N	N	N	N
WP_034019509.1	DUF736 domain-containing protein	N	N	N	N	N	N
WP_046427768.1	NADPH-dependent FMN reductase	N	N	N	N	N	N
WP_049453421.1	UvrD-helicase domain-containing protein	N	N	N	N	N	N
WP_049453423.1	ATP-binding protein	N	N	N	N	N	N
WP_053494789.1	ABC transporter permease	N	N	N	N	N	N
WP_058147580.	helix-turn-helix domain-containing	N	N	N	N	N	N

1	protein						
WP_066708478.1	IS5 family transposase	N	N	N	N	N	N
WP_080353442.1	arsenate reductase ArsC	N	N	N	N	N	N
WP_080353444.1	sulfite exporter TauE/SafE family protein	N	N	N	N	N	N
WP_111206078.1	CusA/CzcA family heavy metal efflux RND transporter	N	N	N	N	N	N
WP_111206079.1	MFS transporter	N	N	N	N	N	N
WP_111206081.1	transmembrane anchor protein	N	N	N	N	N	N
WP_111206503.1	VWA domain-containing protein	N	N	N	N	N	N
WP_111206835.1	TrbI/VirB10 family protein	N	N	N	N	N	N
WP_111207018.1	RebB family R body protein	N	N	N	N	N	N
WP_126870419.1	P-type conjugative transfer protein TrbL	N	N	N	N	N	N
WP_150989999.1	ParA family partition ATPase	N	N	N	N	N	N
WP_150990002.1	chromosome partitioning protein ParB	N	N	N	N	N	N
WP_150990006.	DUF2840 domain-containing protein	N	N	N	N	N	N

1							
WP_150990009.1	S26 family signal peptidase	N	N	N	N	N	N
WP_154344216.1	site-specific integrase	N	N	N	N	NZ_CP040434.1-2	100
WP_154344218.1	permease	N	N	N	N	N	N
WP_154344223.1	arsenical pump-driving ATPase	N	N	N	N	N	N
WP_154344224.1	arsenite efflux transporter metallochaperone ArsD	N	N	N	N	N	N
WP_154344226.1	helix-turn-helix transcriptional regulator	N	N	N	N	N	N
WP_154344227.1	DNA repair protein RadC	N	N	N	N	N	N
WP_154344232.1	helix-turn-helix transcriptional regulator	N	N	N	N	N	N
WP_154344233.1	replication initiator protein A	N	N	N	N	N	N
WP_154344253.1	DUF2958 domain-containing protein	N	N	N	N	N	N
WP_154344254.1	DUF2285 domain-containing protein	N	N	N	N	N	N
WP_168356115.1	HupE/UreJ family protein	N	N	N	N	N	N
WP_223224789.	SRPBCC domain-containing protein	N	N	N	N	N	N

	1							
	WP_229653713.1	thioredoxin family protein	N	N	N	N	N	N
	WP_229653766.1	GlxA family transcriptional regulator	N	N	N	N	N	N
	WP_229653768.1	very short patch repair endonuclease	N	N	N	N	N	N
	WP_005414983.1	ATP-binding protein	N	N	N	N	N	N
	WP_005414984.1	MFS transporter	N	N	N	N	N	N
	WP_010894225.1	integrase	N	N	N	N	N	N
	WP_017354994.1	heavy metal translocating P-type ATPase	NZ_CP040435.1-3	100	N	N	N	N
NZ_CP040435.1	WP_017357195.1	isoprenylcysteine carboxylmethyltransferase family protein	N	N	N	N	N	N
	WP_019337070.1	nuclear transport factor 2 family protein	N	N	N	N	N	N
	WP_021205172.1	DUF2857 domain-containing protein	N	N	N	N	N	N
	WP_033947958.1	hypothetical protein	N	N	N	N	N	N
	WP_033947959.1	AlpA family transcriptional regulator	N	N	N	N	N	N
	WP_033947962.	ParA family protein	N	N	N	N	N	N

1							
WP_033947964.1	hypothetical protein	N	N	N	N	N	N
WP_033947970.1	ParB N-terminal domain-containing protein	N	N	N	N	N	N
WP_033947976.1	STY4528 family pathogenicity island replication protein	N	N	N	N	N	N
WP_033947978.1	TIGR03761 family integrating conjugative element protein	N	N	N	N	N	N
WP_033947980.1	DUF3158 family protein	N	N	N	N	N	N
WP_033947983.1	single-stranded DNA-binding protein	N	N	N	N	N	N
WP_033947991.1	hypothetical protein	N	N	N	N	N	N
WP_033948001.1	calcium/sodium antiporter	N	N	N	N	N	N
WP_033948005.1	hypothetical protein	N	N	N	N	N	N
WP_033948009.1	DUF3085 domain-containing protein	N	N	N	N	N	N
WP_033948010.1	phosphoadenosine phosphosulfate reductase family protein	N	N	N	N	N	N
WP_033948012.1	hypothetical protein	N	N	N	N	N	N
WP_033948013.	DUF3577 domain-containing protein	N	N	N	N	N	N

1							
WP_033948016.1	DUF3275 family protein	N	N	N	N	N	N
WP_033948018.1	hypothetical protein	N	N	N	N	N	N
WP_033948020.1	hypothetical protein	N	N	N	N	N	N
WP_033948024.1	hypothetical protein	N	N	N	N	N	N
WP_033948026.1	DUF6094 domain-containing protein	N	N	N	N	N	N
WP_033948031.1	hypothetical protein	N	N	N	N	N	N
WP_033948037.1	PilL N-terminal domain-containing protein	N	N	N	N	N	N
WP_033948039.1	hypothetical protein	N	N	N	N	N	N
WP_033948041.1	TIGR03759 family integrating conjugative element protein	N	N	N	N	N	N
WP_033948045.1	transglycosylase SLT domain-containing protein	N	N	N	N	N	N
WP_033948047.1	integrating conjugative element protein	N	N	N	N	N	N
WP_033948054.1	TIGR03747 family integrating conjugative element membrane protein	N	N	N	N	N	N
WP_033948057.	RAQPRD family integrative conjugative	N	N	N	N	N	N

1	element protein						
WP_033948059.1	TIGR03758 family integrating conjugative element protein	N	N	N	N	N	N
WP_033948062.1	TIGR03745 family integrating conjugative element membrane protein	N	N	N	N	N	N
WP_033948065.1	TIGR03750 family conjugal transfer protein	N	N	N	N	N	N
WP_033948069.1	TIGR03749 family integrating conjugative element protein	N	N	N	N	N	N
WP_033948071.1	TIGR03752 family integrating conjugative element protein	N	N	N	N	N	N
WP_033948072.1	TIGR03751 family conjugal transfer lipoprotein	N	N	N	N	N	N
WP_033948077.1	DsbA family protein	N	N	N	N	N	N
WP_033948079.1	JAB domain-containing protein	N	N	N	N	N	N
WP_033948081.1	TIGR03757 family integrating conjugative element protein	N	N	N	N	N	N
WP_033948089.1	integrating conjugative element protein	N	N	N	N	N	N
WP_033948091.1	hypothetical protein	N	N	N	N	N	N
WP_033948094.1	DUF3742 family protein	N	N	N	N	N	N
WP_033948096.	RES family NAD+ phosphorylase	N	N	N	N	N	N

1							
WP_033948100.1	MobH family relaxase	N	N	N	N	N	N
WP_033948106.1	MnhB domain-containing protein	N	N	N	N	N	N
WP_033948108.1	NADH-quinone oxidoreductase subunit K	N	N	N	N	N	N
WP_033948109.1	proton-conducting transporter membrane subunit	N	N	N	N	N	N
WP_033948115.1	signal peptidase II	N	N	N	N	N	N
WP_033948116.1	LysR family transcriptional regulator	N	N	N	N	N	N
WP_033948117.1	integrase arm-type DNA-binding domain-containing protein	N	N	N	N	N	N
WP_042920626.1	helix-turn-helix transcriptional regulator	N	N	N	N	N	N
WP_042920638.1	hypothetical protein	N	N	N	N	N	N
WP_042920645.1	DNA cytosine methyltransferase	N	N	N	N	N	N
WP_049404642.1	heavy metal translocating P-type ATPase	N	N	N	N	N	N
WP_049431373.1	type II toxin-antitoxin system RelE/ParE family toxin	N	N	N	N	N	N
WP_049441866.	metalloregulator ArsR/SmtB family	N	N	N	N	N	N

1	transcription factor						
WP_049444104.1	GPW/gp25 family protein	N	N	N	N	N	N
WP_065195982.1	ATP-binding protein	N	N	N	N	N	N
WP_082316348.1	heavy metal translocating P-type ATPase	N	N	NZ_CP040439.1-4	100	N	N
WP_088479298.1	heavy metal response regulator transcription factor	N	N	N	N	N	N
WP_088480360.1	ABC transporter permease	N	N	N	N	N	N
WP_100442745.1	glycosyltransferase	N	N	N	N	N	N
WP_102947286.1	arsenate reductase ArsC	N	N	N	N	N	N
WP_102947288.1	hypothetical protein	N	N	N	N	N	N
WP_102947293.1	IS30 family transposase	N	N	N	N	N	N
WP_102947308.1	sulfite exporter TauE/SafE family protein	N	N	N	N	N	N
WP_154348283.1	UvrD-helicase domain-containing protein	N	N	N	N	N	N
WP_154348404.1	Eco29kI family restriction endonuclease	N	N	N	N	N	N
WP_154348405.	DNA cytosine methyltransferase	N	N	N	N	N	N

1							
WP_154348457.1	ABC transporter ATP-binding protein	N	N	N	N	N	N
WP_154348465.1	glycosyltransferase family 2 protein	N	N	N	N	N	N
WP_154348466.1	multidrug transporter	N	N	N	N	N	N
WP_154348468.1	pyridine nucleotide transhydrogenase	N	N	N	N	N	N
WP_154348469.1	FAD-dependent oxidoreductase	N	N	N	N	N	N
WP_154348470.1	glycosyltransferase	N	N	N	N	N	N
WP_154348775.1	rod shape-determining protein RodA	N	N	N	N	N	N
WP_154348908.1	class I SAM-dependent DNA methyltransferase	N	N	N	N	N	N
WP_154348910.1	DEAD/DEAH box helicase family protein	N	N	N	N	N	N
WP_154348911.1	DUF5655 domain-containing protein	N	N	N	N	N	N
WP_154348943.1	glycosyltransferase	N	N	N	N	N	N
WP_154349074.1	IS30 family transposase	N	N	N	N	N	N
WP_154349077.	zinc-binding dehydrogenase	N	N	N	N	N	N

	1							
	WP_154349788.1	IS30 family transposase	N	N	N	N	N	N
	WP_154349800.1	cytochrome o ubiquinol oxidase subunit IV	N	N	N	N	N	N
	WP_197596277.1	TIM barrel protein	N	N	N	N	N	N
	WP_200964917.1	hydrogen gas-evolving membrane-bound hydrogenase subunit E	N	N	N	N	N	N
	WP_229302222.1	recombinase family protein	N	N	N	N	N	N
	WP_229302303.1	LysR family transcriptional regulator	N	N	N	N	N	N
	WP_005411473.1	cytochrome o ubiquinol oxidase subunit IV	N	N	N	N	N	N
	WP_005995733.1	DUF305 domain-containing protein	N	N	N	N	N	N
	WP_010481480.1	DUF2188 domain-containing protein	N	N	N	N	N	N
NZ_CP040436.1	WP_010484073.1	type II toxin-antitoxin system RelE/ParE family toxin	N	N	N	N	N	N
	WP_019186249.1	metalloregulator ArsR/SmtB family transcription factor	N	N	N	N	N	N
	WP_049404106.1	Dam family site-specific DNA-(adenine-N6)-methyltransferase	N	N	N	N	N	N
	WP_074038336.	NAD(P)/FAD-dependent oxidoreductase	N	N	N	N	N	N

1							
WP_074038356.1	YegP family protein	N	N	N	N	N	N
WP_155028870.1	chemotaxis protein	N	N	N	N	N	N
WP_155028917.1	glycosyltransferase family 2 protein	N	N	N	N	N	N
WP_155028918.1	multidrug transporter	N	N	N	N	N	N
WP_155028919.1	TIM barrel protein	N	N	N	N	N	N
WP_155028920.1	NAD(P)-dependent oxidoreductase	N	N	N	N	N	N
WP_155028921.1	FAD-dependent oxidoreductase	N	N	N	N	N	N
WP_155029322.1	AAA family ATPase	N	N	N	N	N	N
WP_155029399.1	DUF87 domain-containing protein	N	N	N	N	N	N
WP_155029532.1	AAA family ATPase	N	N	N	N	N	N
WP_155029534.1	DNA repair exonuclease	N	N	N	N	N	N
WP_155030259.1	recombinase family protein	N	N	N	N	N	N
WP_195763072.	hypothetical protein	N	N	100		N	N

	1							
	WP_219627573.1	lysozyme	N	N	N	N	N	N
	WP_223846474.1	phage terminase large subunit	N	N	NZ_CP044092.1-1, NZ_CP040436.1-1, NZ_CP049956.1-4	100	N	N
	WP_225792080.1	DUF4423 domain-containing protein	N	N	N	N	N	N
	WP_229301534.1	adenylate/guanylate cyclase domain-containing protein	N	N	N	N	N	N
	WP_229301588.1	glycosyltransferase	N	N	N	N	N	N
	WP_229301595.1	restriction endonuclease	N	N	N	N	N	N
NZ_CP040437.1	WP_049469197.1	GPW/gp25 family protein	N	N	N	N	N	N
	WP_154329991.1	ABC transporter ATP-binding protein	N	N	N	N	N	N
	WP_154330184.1	flagellar biosynthesis protein FlhA	N	N	N	N	N	N
	WP_154330190.1	FliI/YscN family ATPase	N	N	N	N	N	N
	WP_154330194.1	flagellar type III secretion system pore protein FliP	N	N	N	N	N	N
	WP_154330253.1	VOC family protein	N	N	N	N	N	N

	WP_154330258.1	zinc-binding alcohol dehydrogenase family protein	N	N	N	N	N	N
	WP_154330573.1	alkyl sulfatase dimerization domain-containing protein	N	N	N	N	N	N
	WP_154330597.1	SDR family oxidoreductase	N	N	N	N	N	N
	WP_229301859.1	GlxA family transcriptional regulator	N	N	N	N	N	N
	WP_229301970.1	LLM class flavin-dependent oxidoreductase	N	N	N	N	N	N
	WP_229301973.1	3-oxoacyl-ACP reductase family protein	N	N	N	N	N	N
	WP_229301977.1	nuclease PIN	N	N	N	N	N	N
	WP_005413401.1	helix-turn-helix transcriptional regulator	N	N	N	N	NZ_CP043578.1-2	100
	WP_014645522.1	efflux RND transporter periplasmic adaptor subunit	N	N	N	N	N	N
	WP_021202108.1	metalloregulator ArsR/SmtB family transcription factor	N	N	N	N	N	N
NZ_CP040438.1	WP_031269107.1	DUF305 domain-containing protein	N	N	N	N	N	N
	WP_033835782.1	hypothetical protein	N	N	N	N	N	N
	WP_049453421.1	UvrD-helicase domain-containing protein	N	N	N	N	N	N

WP_049453423.1	ATP-binding protein	N	N	N	N	N	N
WP_053516512.1	Qat anti-phage system ATPase QatA	N	N	N	N	N	N
WP_054170770.1	AAA family ATPase	N	N	N	N	N	N
WP_076739061.1	Qat anti-phage system TatD family nuclease QatD	N	N	N	N	N	N
WP_100461253.1	alpha/beta fold hydrolase	N	N	N	N	N	N
WP_154262105.1	McrC family protein	N	N	N	N	N	N
WP_154262508.1	hypothetical protein	N	N	N	N	N	N
WP_154262672.1	flagellar biosynthesis protein FlhA	N	N	N	N	N	N
WP_154262696.1	flagellar type III secretion system pore protein FliP	N	N	N	N	N	N
WP_154263137.1	DUF1320 domain-containing protein	N	N	N	N	N	N
WP_154263140.1	Mu-like prophage major head subunit gpT family protein	N	N	NZ_CP040438.1-1	100	N	N
WP_154263147.1	DUF935 domain-containing protein	N	N	NZ_CP031058.1-5, NZ_CP040438.1-1	100	N	N
WP_154263148.1	hypothetical protein	N	N	NZ_CP040438.1-1	100	N	N

WP_154263287.1	helix-turn-helix domain-containing protein	N	N	N	N	N	N
WP_154263289.1	restriction endonuclease subunit S	N	N	N	N	N	N
WP_154263290.1	AAA family ATPase	N	N	N	N	N	N
WP_154263292.1	restriction endonuclease	N	N	N	N	N	N
WP_154263527.1	AAA family ATPase	N	N	N	N	N	N
WP_154263528.1	ATP-binding protein	N	N	N	N	N	N
WP_154263583.1	helix-turn-helix transcriptional regulator	N	N	N	N	N	N
WP_154263652.1	amidohydrolase	N	N	N	N	N	N
WP_154264118.1	ABC transporter permease	N	N	N	N	N	N
WP_154264119.1	hypothetical protein	N	N	N	N	N	N
WP_154264138.1	SDR family oxidoreductase	N	N	N	N	N	N
WP_154264256.1	NAD(P)/FAD-dependent oxidoreductase	N	N	N	N	N	N
WP_197467664.1	adenylate/guanylate cyclase domain-containing protein	N	N	N	N	N	N

NZ_CP040439.1	WP_204304015.1	ABC transporter ATP-binding protein	N	N	N	N	N	N
	WP_216349761.1	Qat anti-phage system associated protein QatB	N	N	N	N	N	N
	WP_229301101.1	GrpB family protein	N	N	N	N	N	N
	WP_229655248.1	MepB family protein	N	N	N	N	N	N
	WP_229655255.1	TIGR03364 family FAD-dependent oxidoreductase	N	N	N	N	N	N
	WP_229655278.1	flagellar basal body P-ring protein FlgI	N	N	N	N	N	N
	WP_005411473.1	cytochrome o ubiquinol oxidase subunit IV	N	N	N	N	N	N
	WP_008786730.1	glutaredoxin family protein	N	N	N	N	N	N
	WP_008786732.1	MFS transporter	N	N	N	N	N	N
	WP_008786734.1	efflux transporter outer membrane subunit	N	N	N	N	N	N
	WP_008786736.1	efflux RND transporter periplasmic adaptor subunit	N	N	N	N	N	N
	WP_008786737.1	metalloregulator ArsR/SmtB family transcription factor	N	N	N	N	N	N
	WP_008786738.1	cation diffusion facilitator family transporter	N	N	N	N	N	N

WP_008786741.1	metalloregulator ArsR/SmtB family transcription factor	N	N	N	N	N	N
WP_008786745.1	glutaredoxin	N	N	N	N	N	N
WP_008786747.1	LysR substrate-binding domain-containing protein	N	N	N	N	N	N
WP_008786748.1	EexN family lipoprotein	N	N	N	N	N	N
WP_008786751.1	CopG family transcriptional regulator	N	N	N	N	N	N
WP_008786753.1	TrbC/VirB2 family protein	N	N	N	N	N	N
WP_008786756.1	P-type conjugative transfer protein TrbJ	N	N	N	N	N	N
WP_008786757.1	hypothetical protein	N	N	N	N	N	N
WP_008786881.1	TrbI/VirB10 family protein	N	N	N	N	N	N
WP_008786882.1	P-type conjugative transfer protein TrbG	N	N	N	N	N	N
WP_017357195.1	isoprenylcysteine carboxylmethyltransferase family protein	N	N	N	N	N	N
WP_019396553.1	Lrp/AsnC ligand binding domain-containing protein	N	N	N	N	N	N
WP_021162751.1	MFS transporter	N	N	N	N	N	N

WP_021221531.1	P-type conjugative transfer protein TrbL	N	N	N	N	N	N
WP_023980278.1	ATP-binding protein	N	N	N	N	N	N
WP_026069907.1	helix-turn-helix domain-containing protein	N	N	N	N	N	N
WP_027080080.1	heavy metal translocating P-type ATPase	N	N	N	N	N	N
WP_032127356.1	HigA family addiction module antitoxin	N	N	N	N	N	N
WP_033946760.1	DUF736 domain-containing protein	N	N	N	N	N	N
WP_033946761.1	DUF2958 domain-containing protein	N	N	N	N	N	N
WP_033946763.1	DUF2285 domain-containing protein	N	N	N	N	N	N
WP_033946769.1	DUF2840 domain-containing protein	N	N	N	N	N	N
WP_033946771.1	S26 family signal peptidase	N	N	N	N	N	N
WP_033946790.1	hypothetical protein	N	N	N	N	N	N
WP_033948200.1	site-specific integrase	N	N	N	N	N	N
WP_033948212.1	S8 family peptidase	N	N	N	N	N	N

WP_033948218.1	hypothetical protein	N	N	N	N	N	N
WP_046984173.1	CusA/CzcA family heavy metal efflux RND transporter	N	N	N	N	N	N
WP_046984174.1	MFS transporter	N	N	N	N	N	N
WP_046984177.1	hypothetical protein	N	N	N	N	N	N
WP_049398866.1	isocitrate lyase/PEP mutase family protein	N	N	N	N	N	N
WP_049404642.1	heavy metal translocating P-type ATPase	N	N	N	N	N	N
WP_049406750.1	hypothetical protein	N	N	N	N	N	N
WP_049406765.1	tyrosine-type recombinase/integrase	N	N	N	N	N	N
WP_049407016.1	hypothetical protein	N	N	N	N	N	N
WP_049407018.1	BREX-3 system phosphatase PglZ	N	N	N	N	N	N
WP_049407022.1	DNA methyltransferase	N	N	N	N	N	N
WP_049407026.1	WYL domain-containing protein	N	N	N	N	N	N
WP_049407028.1	DUF6079 family protein	N	N	N	N	N	N

WP_049421310.1	LysR substrate-binding domain-containing protein	N	N	N	N	N	N
WP_052151340.1	DUF1016 N-terminal domain-containing protein	N	N	N	N	N	N
WP_053499309.1	Fic family protein	N	N	N	N	N	N
WP_053499319.1	type I restriction endonuclease subunit R	N	N	N	N	N	N
WP_053499322.1	SprT family zinc-dependent metalloprotease	N	N	N	N	N	N
WP_053499323.1	integrase domain-containing protein	N	N	N	N	N	N
WP_053506350.1	helix-turn-helix transcriptional regulator	N	N	N	N	N	N
WP_071540644.1	helix-turn-helix transcriptional regulator	N	N	N	N	N	N
WP_080356388.1	ADP-ribosylglycohydrolase family protein	N	N	N	N	N	N
WP_082316348.1	heavy metal translocating P-type ATPase	N	N	NZ_CP040439.1-4	100	N	N
WP_083536958.1	GDCCVxC domain-containing (seleno)protein	N	N	N	N	N	N
WP_099605285.1	P-type conjugative transfer protein TrbL	N	N	N	N	N	N
WP_099605287.1	P-type conjugative transfer protein TrbJ	N	N	N	N	N	N

WP_099605290.1	TrbC/VirB2 family protein	N	N	N	N	N	N
WP_099605292.1	ribbon-helix-helix protein, CopG family	N	N	N	N	N	N
WP_099605294.1	EexN family lipoprotein	N	N	N	N	N	N
WP_099605299.1	ATP-binding protein	N	N	N	N	N	N
WP_114618472.1	TIGR03758 family integrating conjugative element protein	N	N	N	N	N	N
WP_150359710.1	AlpA family phage regulatory protein	N	N	N	N	N	N
WP_150359735.1	hypothetical protein	N	N	N	N	N	N
WP_154263497.1	GNAT family N-acetyltransferase	N	N	N	N	N	N
WP_154263583.1	helix-turn-helix transcriptional regulator	N	N	N	N	N	N
WP_154351866.1	MFS transporter	N	N	N	N	N	N
WP_154351951.1	AIPR family protein	N	N	N	N	N	N
WP_154351953.1	Z1 domain-containing protein	N	N	N	N	N	N
WP_154352304.1	redox-sensitive transcriptional activator SoxR	N	N	N	N	N	N

WP_154352305.1	MFS transporter	N	N	N	N	N	N
WP_154352384.1	DNA repair protein RadC	N	N	N	N	N	N
WP_154352385.1	DUF2958 domain-containing protein	N	N	N	N	N	N
WP_154352389.1	hypothetical protein	N	N	N	N	N	N
WP_154352396.1	helix-turn-helix domain-containing protein	N	N	N	N	N	N
WP_154352398.1	DUF736 domain-containing protein	N	N	N	N	N	N
WP_154352399.1	DUF2958 domain-containing protein	N	N	N	N	N	N
WP_154352400.1	DUF2285 domain-containing protein	N	N	NZ_CP040439.1-8	100	N	N
WP_154352401.1	helix-turn-helix domain-containing protein	N	N	N	N	N	N
WP_154352404.1	chromosome partitioning protein ParB	N	N	N	N	N	N
WP_154352405.1	DUF2840 domain-containing protein	N	N	N	N	N	N
WP_154352406.1	S26 family signal peptidase	N	N	NZ_CP040439.1-8	100	N	N
WP_154352408.1	SNF2-related protein	N	N	N	N	N	N

WP_154352411.1	hypothetical protein	N	N	N	N	N	N
WP_154352412.1	DUF499 domain-containing protein	N	N	N	N	N	N
WP_154352418.1	P-type conjugative transfer protein TrbG	N	N	N	N	N	N
WP_154352419.1	TrbI/VirB10 family protein	N	N	N	N	N	N
WP_154352421.1	nucleotidyl transferase AbiEii/AbiGii toxin family protein	N	N	N	N	N	N
WP_154352518.1	type II toxin-antitoxin system RelE/ParE family toxin	N	N	N	N	N	N
WP_154352537.1	alpha/beta hydrolase	N	N	N	N	N	N
WP_154352539.1	AraC family transcriptional regulator	N	N	N	N	N	N
WP_154352540.1	LysR family transcriptional regulator	N	N	N	N	N	N
WP_154352542.1	NAD(P)-dependent alcohol dehydrogenase	N	N	N	N	N	N
WP_154352544.1	VOC family protein	N	N	N	N	N	N
WP_154352547.1	SDR family oxidoreductase	N	N	N	N	N	N
WP_154352551.1	HlyD family secretion protein	N	N	N	N	N	N

WP_154352576.1	heme-binding protein	N	N	N	N	N	N
WP_154352577.1	SDR family oxidoreductase	N	N	N	N	N	N
WP_154352579.1	oxidoreductase	N	N	N	N	N	N
WP_154352581.1	pirin family protein	N	N	N	N	N	N
WP_154352582.1	LysR family transcriptional regulator	N	N	N	N	N	N
WP_154352584.1	NAD(P)H-dependent oxidoreductase	N	N	N	N	N	N
WP_154352596.1	efflux transporter outer membrane subunit	N	N	N	N	N	N
WP_154352597.1	MFS transporter	N	N	N	N	N	N
WP_154352598.1	HlyD family secretion protein	N	N	N	N	N	N
WP_154352599.1	glycosyltransferase	N	N	N	N	N	N
WP_154352601.1	hypothetical protein	N	N	N	N	N	N
WP_154353036.1	metalloregulator ArsR/SmtB family transcription factor	N	N	N	N	N	N
WP_154353282.1	type II toxin-antitoxin system RelE/ParE family toxin	N	N	N	N	N	N

WP_154353368.1	M24 family metallopeptidase	N	N	N	N	N	N
WP_154353611.1	LysR family transcriptional regulator	N	N	N	N	N	N
WP_169708473.1	metal-sensing transcriptional repressor	N	N	N	N	N	N
WP_204315338.1	DUF1156 domain-containing protein	N	N	N	N	N	N
WP_204315339.1	hypothetical protein	N	N	N	N	N	N
WP_207384764.1	class I SAM-dependent DNA methyltransferase	N	N	N	N	N	N
WP_223224739.1	IS3 family transposase	N	N	N	N	N	N
WP_229300960.1	SDR family NAD(P)-dependent oxidoreductase	N	N	N	N	N	N
WP_229300962.1	FAD-dependent oxidoreductase	N	N	N	N	N	N
WP_229300966.1	hypothetical protein	NZ_CP040439.1-2	100	N	N	N	N
WP_229300977.1	MBL fold metallo-hydrolase	N	N	N	N	N	N
WP_229301049.1	hypothetical protein	N	N	N	N	N	N
WP_229301101.1	GrpB family protein	N	N	N	N	N	N

	WP_229301157.1	ATP-binding protein	N	N	N	N	N	N
	WP_229301169.1	helicase-related protein	N	N	N	N	N	N
	WP_229301194.1	ABC transporter ATP-binding protein	N	N	N	N	N	N
	WP_229301213.1	type IV toxin-antitoxin system AbiEi family antitoxin	N	N	N	N	N	N
	WP_229301217.1	integrase core domain-containing protein	N	N	N	N	N	N
	WP_229301224.1	very short patch repair endonuclease	N	N	N	N	N	N
	WP_005408332.1	GPW/gp25 family protein	N	N	N	N	N	N
	WP_005409659.1	heat resistance system K+/H+ antiporter KefB-GI	NZ_CP060026.1-2	100	NZ_CP040440.1-7, NZ_CP088244.1-3	100	NZ_CP060025.1-2, NZ_CP060027.1-4, NZ_CP098483.1-4	100
NZ_CP040440.1	WP_005409661.1	hypothetical protein	NZ_CP060026.1-2	100	N	N	NZ_CP060025.1-2, NZ_CP060027.1-4, NZ_CP098483.1-4	100
	WP_005409662.1	HdeD family acid-resistance protein	NZ_CP060026.1-2	100	N	N	NZ_CP060025.1-2, NZ_CP060027.1-4, NZ_CP098483.1-4	100
	WP_005409663.1	heat resistance protein YfdX2	NZ_CP060026.1-2	100	N	N	NZ_CP060025.1-2, NZ_CP060027.1-4, NZ_CP098483.1-4,	100

						NZ_CP102942.1-4	
WP_005409664.1	heat resistance protein YfdX1	NZ_CP060026.1-2	100	N	N	NZ_CP060025.1-2,<br>NZ_CP060027.1-4,<br>NZ_CP102942.1-4,<br>NZ_CP098483.1-4	100
WP_005409665.1	small heat shock protein sHSP20-GI	N	N	N	N	N	N
WP_005409667.1	cardiolipin synthase	N	N	N	N	N	N
WP_005409669.1	Hsp20/alpha crystallin family protein	NZ_CP060026.1-2	100	NZ_CP040431.1-4,<br>NZ_CP040433.1-2,<br>NZ_CP040440.1-7,<br>NZ_CP088244.1-4	100	NZ_CP060025.1-2,<br>NZ_CP060027.1-4,<br>NZ_CP102942.1-4,<br>NZ_CP098483.1-4	100
WP_011517532.1	cytochrome b	N	N	N	N	N	N
WP_012480368.1	TIGR03885 family FMN-dependent LLM class oxidoreductase	N	N	N	N	N	N
WP_012481497.1	cytochrome o ubiquinol oxidase subunit IV	N	N	N	N	N	N
WP_014859386.1	DUF2840 domain-containing protein	N	N	N	N	N	N
WP_014859387.1	hypothetical protein	N	N	N	N	N	N
WP_014859391.1	helix-turn-helix domain-containing protein	N	N	N	N	N	N
WP_014859392.	DUF2285 domain-containing protein	N	N	NZ_CP040440.1-8,	100		

1				NZ_CP060026.1-4			
WP_014859394.1	DUF2958 domain-containing protein	N	N	N	N	N	N
WP_014859395.1	hypothetical protein	N	N	N	N	N	N
WP_014861255.1	DNA repair protein RadC	N	N	N	N	N	N
WP_014861256.1	PDDEXK nuclease domain-containing protein	N	N	NZ_CP040440.1-8, NZ_CP060026.1-4	100	N	N
WP_014861257.1	site-specific integrase	N	N	NZ_CP040440.1-8, NZ_CP060026.1-4	100	N	N
WP_024001347.1	ferric reductase-like transmembrane domain-containing protein	N	N	N	N	N	N
WP_024537630.1	S26 family signal peptidase	N	N	NZ_CP040440.1-8, NZ_CP060026.1-4	100	N	N
WP_024956907.1	LysR substrate-binding domain-containing protein	N	N	N	N	N	N
WP_032961714.1	heat resistance protein PsiE-GI	N	N	N	N	N	N
WP_034400679.1	diacylglycerol kinase	N	N	N	N	N	N
WP_043033545.1	GNAT family N-acetyltransferase	N	N	N	N	N	N
WP_043371530.1	TrbC/VirB2 family protein	N	N	N	N	N	N
WP_049395161.	histidine utilization repressor	N	N	N	N	N	N

1							
WP_049395165.1	DUF305 domain-containing protein	N	N	N	N	N	N
WP_049395288.1	helix-turn-helix transcriptional regulator	N	N	N	N	N	N
WP_049395575.1	P-type conjugative transfer protein TrbL	N	N	N	N	N	N
WP_049395576.1	hypothetical protein	N	N	N	N	N	N
WP_049395578.1	P-type conjugative transfer protein TrbJ	N	N	N	N	N	N
WP_049395585.1	LysR family transcriptional regulator	N	N	N	N	N	N
WP_049396030.1	TM0106 family RecB-like putative nuclease	NZ_CP060026.1-2		NZ_CP040440.1-7		N	N
WP_049396036.1	helix-turn-helix domain-containing protein	N	N	N	N	N	N
WP_049396984.1	helix-turn-helix domain-containing protein	N	N	N	N	N	N
WP_049396991.1	group II intron reverse transcriptase/maturase	N	N	N	N	N	N
WP_049397307.1	IS110 family transposase	NZ_CP060026.1-2	100	NZ_CP040440.1-7	100	NZ_CP102942.1-4	100
WP_049397460.1	LysR family transcriptional regulator	N	N	N	N	N	N
WP_049397550.	ABC transporter ATP-binding protein	N	N	N	N	N	N

1							
WP_049397866.1	phage terminase large subunit	NZ_CP040440.1-2	100	NZ_CP060026.1-5	100	N	N
WP_049406765.1	tyrosine-type recombinase/integrase	N	N	N	N	N	N
WP_049406835.1	phosphoadenosine phosphosulfate reductase family protein	N	N	N	N	N	N
WP_049407014.1	DUF262 domain-containing protein	N	N	N	N	N	N
WP_049407016.1	hypothetical protein	N	N	N	N	N	N
WP_049407018.1	BREX-3 system phosphatase PglZ	N	N	N	N	N	N
WP_049407022.1	DNA methyltransferase	N	N	N	N	N	N
WP_049407025.1	BREX-3 system P-loop-containing protein BrxF	N	N	N	N	N	N
WP_049407026.1	WYL domain-containing protein	N	N	N	N	N	N
WP_049407028.1	DUF6079 family protein	N	N	N	N	N	N
WP_062166963.1	nucleotide-binding protein	N	N	NZ_CP040440.1-8, NZ_CP060026.1-4		N	N
WP_079390835.1	helix-turn-helix transcriptional regulator	N	N	N	N	N	N
WP_080240930.	ABC transporter permease	N	N	N	N	N	N

1							
WP_080356388.1	ADP-ribosylglycohydrolase family protein	N	N	N	N	N	N
WP_088611174.1	tyrosine-type recombinase/integrase	NZ_CP040440.1-5, NZ_CP060026.1-3	100	N	N	NZ_CP022053.2-8	100
WP_099540247.1	DNA cytosine methyltransferase	NZ_CP040440.1-1, NZ_CP060026.1-1	100	N	N	N	N
WP_154344303.1	GPW/gp25 family protein	N	N	N	N	N	N
WP_154344436.1	terminase	N	N	N	N	N	N
WP_162838283.1	DEAD/DEAH box helicase family protein	N	N	N	N	N	N
WP_197594234.1	helicase-related protein	N	N	N	N	N	N
WP_201022396.1	IS3 family transposase	N	N	N	N	N	N
WP_219627573.1	lysozyme	N	N	N	N	N	N
WP_229298563.1	TatD family hydrolase	N	N	N	N	N	N
WP_229298564.1	Mov34/MPN/PAD-1 family protein	N	N	N	N	N	N
WP_229302578.1	formimidoylglutamase	N	N	N	N	N	N
WP_229302592.	type II toxin-antitoxin system RelE/ParE	N	N	N	N	N	N

	1	family toxin						
	WP_229302610.1	NAD(P)H-dependent oxidoreductase	N	N	N	N	N	N
	WP_229302632.1	nuclear transport factor 2 family protein	N	N	N	N	N	N
	WP_229302653.1	amidohydrolase	N	N	N	N	N	N
	WP_000058717.1	EamA family transporter	N	N	N	N	NZ_CP043578.1-2	100
	WP_000164043.1	tetracycline resistance transcriptional repressor TetR(A)	N	N	N	N	NZ_CP043578.1-2	100
	WP_000179844.1	DDE-type integrase/transposase/recombinase	N	N	N	N	NZ_CP043578.1-2	100
	WP_000804064.1	tetracycline efflux MFS transporter Tet(A)	N	N	N	N	N	N
NZ_CP043578.1	WP_000995360.1	mercury resistance co-regulator MerD	N	N	N	N	N	N
	WP_003131974.1	mercuric ion transporter MerT	N	N	N	N	N	N
	WP_003131987.1	mercury resistance system periplasmic binding protein MerP	N	N	N	N	N	N
	WP_003155746.1	TniB family NTP-binding protein	N	N	N	N	N	N
	WP_003159191.1	aminoglycoside N-acetyltransferase AAC(6')-Ib4	N	N	N	N	N	N
	WP_003462990.	N-6 DNA methylase	N	N	N	N	N	N

1							
WP_003462995.1	type II toxin-antitoxin system RelE/ParE family toxin	N	N	N	N	N	N
WP_003462997.1	site-specific integrase	N	N	N	N	N	N
WP_004217865.1	class 1 integron integrase IntI1	N	N	N	N	N	N
WP_005413387.1	Hg(II)-responsive transcriptional regulator	N	N	N	N	N	N
WP_005413400.1	Tn3-like element TnAs1 family transposase	N	N	N	N	NZ_CP043578.1-2	100
WP_006122485.1	TniQ family protein	N	N	N	N	N	N
WP_006399830.1	cytochrome o ubiquinol oxidase subunit IV	N	N	N	N	N	N
WP_008264806.1	hypothetical protein	N	N	N	N	N	N
WP_008264906.1	HupE/UreJ family protein	N	N	N	N	N	N
WP_008266162.1	CusA/CzcA family heavy metal efflux RND transporter	N	N	N	N	N	N
WP_008266446.1	SDR family oxidoreductase	N	N	N	N	N	N
WP_008267238.1	MFS transporter	N	N	N	N	N	N
WP_014859391.	helix-turn-helix domain-containing	N	N	N	N	N	N

1	protein						
WP_021202108.1	metalloregulator ArsR/SmtB family transcription factor	N	N	N	N	N	N
WP_027016815.1	DNA-binding transcriptional regulator	N	N	N	N	N	N
WP_032127338.1	GNAT family N-acetyltransferase	N	N	N	N	N	N
WP_032128664.1	metalloregulator ArsR/SmtB family transcription factor	N	N	N	N	N	N
WP_044314809.1	PadR family transcriptional regulator	N	N	N	N	N	N
WP_044314813.1	LysR substrate-binding domain-containing protein	N	N	N	N	N	N
WP_044314814.1	EexN family lipoprotein	N	N	N	N	N	N
WP_049398866.1	isocitrate lyase/PEP mutase family protein	N	N	N	N	N	N
WP_049399243.1	MepB family protein	N	N	N	N	N	N
WP_049422655.1	type II toxin-antitoxin system RelE/ParE family toxin	N	N	N	N	N	N
WP_049423593.1	redox-sensitive transcriptional activator SoxR	N	N	N	N	N	N
WP_049453421.1	UvrD-helicase domain-containing protein	N	N	N	N	N	N
WP_049453423.	ATP-binding protein	N	N	N	N	N	N

1							
WP_080353442.1	arsenate reductase ArsC	N	N	N	N	N	N
WP_080353444.1	sulfite exporter TauE/SafE family protein	N	N	N	N	N	N
WP_086009122.1	IS3-like element ISPa39 family transposase	N	N	N	N	N	N
WP_105029020.1	VirB3 family type IV secretion system protein	N	N	N	N	N	N
WP_108497791.1	hypothetical protein	N	N	N	N	N	N
WP_108497792.1	P-type conjugative transfer protein TrbJ	N	N	N	N	N	N
WP_108497794.1	TrbC/VirB2 family protein	N	N	N	N	N	N
WP_108497957.1	P-type conjugative transfer protein TrbG	N	N	N	N	N	N
WP_122379238.1	P-type conjugative transfer protein TrbL	N	N	N	N	N	N
WP_122379239.1	TrbI/VirB10 family protein	N	N	N	N	N	N
WP_175223965.1	helix-turn-helix transcriptional regulator	N	N	N	N	N	N
WP_180876981.1	Hcp family type VI secretion system effector	N	N	N	N	N	N
WP_180885796.	mercury(II) reductase	N	N	N	N	NZ_CP043578.1-2	100

1							
WP_182266109.1	NAD(P)-dependent alcohol dehydrogenase	N	N	N	N	N	N
WP_182266110.1	LysR substrate-binding domain-containing protein	N	N	N	N	N	N
WP_182266192.1	CopG family transcriptional regulator	N	N	N	N	N	N
WP_182266193.1	methyltransferase domain-containing protein	N	N	N	N	N	N
WP_182266194.1	S26 family signal peptidase	N	N	N	N	N	N
WP_182266195.1	DUF2840 domain-containing protein	N	N	N	N	N	N
WP_182266196.1	chromosome partitioning protein ParB	N	N	N	N	N	N
WP_182266199.1	DUF2285 domain-containing protein	N	N	N	N	N	N
WP_182266200.1	DUF2958 domain-containing protein	N	N	N	N	N	N
WP_182266201.1	nucleotide-binding protein	N	N	N	N	N	N
WP_182266202.1	DUF736 domain-containing protein	N	N	N	N	N	N
WP_182266203.1	hypothetical protein	N	N	N	N	N	N
WP_182266204.	ParB/RepB/Spo0J family partition	N	N	N	N	N	N

1	protein						
WP_182266692.1	DUF3304 domain-containing protein	N	N	N	N	N	N
WP_182266797.1	virulence RhuM family protein	N	N	N	N	N	N
WP_182266895.1	SIR2 family protein	N	N	N	N	N	N
WP_182266944.1	DUF3304 domain-containing protein	N	N	N	N	N	N
WP_182266994.1	MFS transporter	N	N	N	N	N	N
WP_182267025.1	McrC family protein	N	N	N	N	N	N
WP_182267027.1	N-6 DNA methylase	N	N	N	N	N	N
WP_182267140.1	DNA cytosine methyltransferase	N	N	N	N	N	N
WP_182267141.1	endonuclease	N	N	N	N	N	N
WP_182267167.1	type VI secretion system contractile sheath small subunit	N	N	N	N	N	N
WP_182267173.1	type VI secretion system baseplate subunit TssK	N	N	N	N	N	N
WP_182267175.1	type VI secretion system ATPase TssH	N	N	N	N	N	N
WP_182267226.	ABC transporter permease	N	N	N	N	N	N

	1							
	WP_182267227.1	ABC transporter ATP-binding protein	N	N	N	N	N	N
	WP_182267616.1	MFS transporter	N	N	N	N	N	N
	WP_182267677.1	LysR substrate-binding domain-containing protein	N	N	N	N	N	N
	WP_182267823.1	phage integrase N-terminal SAM-like domain-containing protein	N	N	N	N	N	N
	WP_182267824.1	recombinase family protein	N	N	N	N	N	N
	WP_198422072.1	type VI secretion system baseplate subunit TssF	N	N	N	N	N	N
	WP_223224789.1	SRPBCC domain-containing protein	N	N	N	N	N	N
	WP_232527984.1	IS3 family transposase	N	N	N	N	N	N
	WP_232528068.1	IS3 family transposase	N	N	N	N	N	N
	WP_232528088.1	GrpB family protein	N	N	N	N	N	N
	WP_232528363.1	helix-turn-helix transcriptional regulator	N	N	N	N	N	N
NZ_CP044029.1	WP_000483554.1	peptide-methionine (R)-S-oxide reductase MsrB	N	N	N	N	N	N
	WP_005408332.	GPW/gp25 family protein	N	N	N	N	N	N

1							
WP_025467194.1	glutaredoxin family protein	N	N	N	N	N	N
WP_043033961.1	hypothetical protein	N	N	N	N	N	N
WP_047290287.1	calcium/sodium antiporter	N	N	N	N	N	N
WP_047290288.1	MnhB domain-containing protein	N	N	N	N	N	N
WP_047290294.1	hypothetical protein	N	N	N	N	N	N
WP_049396379.1	nuclear transport factor 2 family protein	N	N	N	N	N	N
WP_049397868.1	lysozyme	N	N	N	N	N	N
WP_049404266.1	lysozyme	N	N	N	N	N	N
WP_049431274.1	site-specific integrase	N	N	N	N	N	N
WP_053506162.1	nucleotidyl transferase AbiEii/AbiGii toxin family protein	N	N	N	N	N	N
WP_062607032.1	nucleoside triphosphatase NudI	N	N	N	N	N	N
WP_099605294.1	EexN family lipoprotein	N	N	N	N	N	N
WP_099605299.	ATP-binding protein	N	N	N	N	N	N

1							
WP_111111829.1	SMR family transporter	N	N	N	N	N	N
WP_111118248.1	ISL3 family transposase	N	N	N	N	N	N
WP_121335901.1	IS5 family transposase	N	N	N	N	N	N
WP_150359622.1	adenine methyltransferase	N	N	NZ_CP044092.1-3	100	N	N
WP_150359627.1	YqaJ viral recombinase family protein	N	N	NZ_CP044092.1-3	100	N	N
WP_150359630.1	hypothetical protein	N	N	N	N	N	N
WP_150359645.1	DUF2280 domain-containing protein	N	N	N	N	N	N
WP_150359694.1	type ISP restriction/modification enzyme	N	N	NZ_CP044092.1-5	100	N	N
WP_150359711.1	DUF2958 domain-containing protein	N	N	N	N	N	N
WP_150359712.1	hypothetical protein	N	N	NZ_CP044092.1-6	100	N	N
WP_150359714.1	hypothetical protein	N	N	N	N	N	N
WP_150359715.1	AAA family ATPase	N	N	NZ_CP044092.1-6	100	N	N
WP_150359716.	UvrD-helicase domain-containing	N	N	NZ_CP044092.1-6	100	N	N

1	protein						
WP_150359717.1	Tn3-like element IS1071 family transposase	N	N	NZ_CP044092.1-6, NZ_CP044092.1-7	100	N	N
WP_150359720.1	helix-turn-helix domain-containing protein	N	N	N	N	N	N
WP_150359726.1	S26 family signal peptidase	N	N	N	N	N	N
WP_150359729.1	hypothetical protein	N	N	N	N	N	N
WP_150359730.1	DUF1156 domain-containing protein	N	N	N	N	N	N
WP_150359732.1	hypothetical protein	N	N	N	N	N	N
WP_150359733.1	DUF499 domain-containing protein	N	N	N	N	N	N
WP_150359739.1	magnesium and cobalt transport protein CorA	N	N	N	N	N	N
WP_150359743.1	IS5 family transposase	N	N	N	N	N	N
WP_190953015.1	SNF2-related protein	N	N	N	N	N	N
WP_199522034.1	type IV toxin-antitoxin system AbiEi family antitoxin	N	N	N	N	N	N
WP_223846388.1	hypothetical protein	N	N	N	N	N	N
WP_223846390.	putative metallopeptidase	N	N	N	N	N	N

	1							
	WP_223846408.1	SDR family oxidoreductase	N	N	N	N	N	N
	WP_223846474.1	phage terminase large subunit	N	N	NZ_CP044092.1-1, NZ_CP040436.1-1, NZ_CP049956.1-4	100	N	N
	WP_223846491.1	transposase	N	N	N	N	N	N
	WP_004136660.1	putative addiction module antidote protein	N	N	N	N	N	N
	WP_005409501.1	transcriptional regulator BetI	N	N	N	N	N	N
	WP_005409726.1	ABC transporter permease	N	N	N	N	N	N
	WP_005409874.1	biopolymer transporter ExbD	N	N	N	N	N	N
NZ_CP047310.1	WP_005413654.1	Lrp/AsnC family transcriptional regulator	N	N	N	N	N	N
	WP_005416613.1	ABC transporter ATP-binding protein	N	N	N	N	N	N
	WP_006402206.1	acyl carrier protein	N	N	N	N	N	N
	WP_006425764.1	aminotransferase class III-fold pyridoxal phosphate-dependent enzyme	N	N	N	N	N	N
	WP_010481563.1	response regulator transcription factor	N	N	N	N	N	N

WP_010482111.1	cytochrome o ubiquinol oxidase subunit III	N	N	N	N	N	N
WP_010482114.1	ubiquinol oxidase subunit II	N	N	N	N	N	N
WP_010482305.1	hypothetical protein	N	N	N	N	N	N
WP_010482365.1	DNA polymerase III subunit epsilon	N	N	N	N	N	N
WP_010482574.1	Cu(I)-responsive transcriptional regulator	N	N	N	N	N	N
WP_010483185.1	DMT family transporter	N	N	N	N	N	N
WP_010483441.1	alpha/beta hydrolase	N	N	N	N	N	N
WP_010483459.1	helix-turn-helix transcriptional regulator	N	N	N	N	N	N
WP_010483493.1	LysR family transcriptional regulator	N	N	N	N	N	N
WP_010483737.1	glutathione S-transferase family protein	N	N	N	N	N	N
WP_010484041.1	formate dehydrogenase subunit gamma	N	N	N	N	N	N
WP_010484073.1	type II toxin-antitoxin system RelE/ParE family toxin	N	N	N	N	N	N
WP_010484092.1	winged helix-turn-helix domain-containing protein	N	N	N	N	N	N

WP_010484895.1	helix-turn-helix domain-containing protein	N	N	N	N	N	N
WP_010484993.1	Rid family hydrolase	N	N	N	N	N	N
WP_010485178.1	sigma-70 family RNA polymerase sigma factor	N	N	N	N	N	N
WP_010485179.1	VOC family protein	N	N	N	N	N	N
WP_010485313.1	respiratory nitrate reductase subunit gamma	N	N	N	N	N	N
WP_010485323.1	AzlD family protein	N	N	N	N	N	N
WP_010485668.1	LysR family transcriptional regulator	N	N	N	N	N	N
WP_010485778.1	response regulator	N	N	N	N	N	N
WP_010486459.1	VOC family protein	N	N	N	N	N	N
WP_010486561.1	alpha/beta hydrolase	N	N	N	N	N	N
WP_010487140.1	VOC family protein	N	N	N	N	N	N
WP_019336271.1	hypothetical protein	N	N	N	N	N	N
WP_019336516.1	carboxymuconolactone decarboxylase family protein	N	N	N	N	N	N

WP_019336795.1	polysaccharide biosynthesis tyrosine autokinase	N	N	N	N	N	N
WP_019337583.1	response regulator	N	N	N	N	N	N
WP_019337795.1	type II toxin-antitoxin system RelE/ParE family toxin	N	N	N	N	N	N
WP_019337810.1	NarK family nitrate/nitrite MFS transporter	N	N	N	N	N	N
WP_019338001.1	class I SAM-dependent methyltransferase	N	N	N	N	N	N
WP_019338179.1	response regulator transcription factor	N	N	N	N	N	N
WP_019338302.1	GNAT family N-acetyltransferase	N	N	N	N	N	N
WP_019338770.1	formate dehydrogenase subunit beta	N	N	N	N	N	N
WP_019338970.1	DNA-binding transcriptional regulator	N	N	N	N	N	N
WP_019659837.1	winged helix-turn-helix domain-containing protein	N	N	N	N	N	N
WP_019660267.1	DUF2239 family protein	N	N	N	N	N	N
WP_019660556.1	metalloregulator ArsR/SmtB family transcription factor	N	N	N	N	N	N
WP_020424480.1	response regulator	N	N	N	N	N	N

WP_021202349.1	NAD(P)-dependent oxidoreductase	N	N	N	N	N	N
WP_021203687.1	HigA family addiction module antitoxin	N	N	N	N	N	N
WP_029550852.1	low molecular weight protein-tyrosine-phosphatase	N	N	N	N	N	N
WP_043395953.1	ATP-binding cassette domain-containing protein	N	N	N	N	N	N
WP_043396137.1	TIGR03571 family LLM class oxidoreductase	N	N	N	N	N	N
WP_043396245.1	type II secretion system major pseudopilin GspG	N	N	N	N	N	N
WP_043397376.1	hypothetical protein	N	N	N	N	N	N
WP_043397553.1	VOC family protein	N	N	N	N	N	N
WP_043397864.1	UDP-N-acetylglucosamine 2-epimerase (non-hydrolyzing)	N	N	N	N	N	N
WP_043398268.1	LysR substrate-binding domain-containing protein	N	N	N	N	N	N
WP_043399281.1	protein deglycase HchA	N	N	N	N	N	N
WP_043399287.1	alpha/beta hydrolase	N	N	N	N	N	N
WP_043399309.1	GNAT family N-acetyltransferase	N	N	N	N	N	N

WP_043400854.1	SDR family oxidoreductase	N	N	N	N	N	N
WP_043401378.1	helix-turn-helix domain-containing protein	N	N	N	N	N	N
WP_043402402.1	alpha/beta hydrolase	N	N	N	N	N	N
WP_049409424.1	helix-turn-helix transcriptional regulator	N	N	N	N	N	N
WP_049410845.1	SDR family oxidoreductase	N	N	N	N	N	N
WP_049412996.1	helix-turn-helix domain-containing protein	N	N	N	N	N	N
WP_049413046.1	response regulator transcription factor	N	N	N	N	N	N
WP_049434910.1	SRPBCC family protein	N	N	N	N	N	N
WP_049437693.1	MerR family transcriptional regulator	N	N	N	N	N	N
WP_049453529.1	BCCT family transporter	N	N	N	N	N	N
WP_049455405.1	MerR family transcriptional regulator	N	N	N	N	N	N
WP_049481079.1	ATP-binding protein	N	N	N	N	N	N
WP_049482036.1	MFS transporter	N	N	N	N	N	N

WP_049482252.1	TetR/AcrR family transcriptional regulator	N	N	N	N	N	N
WP_052148783.1	diacylglycerol kinase	N	N	N	N	N	N
WP_053443265.1	LysR substrate-binding domain-containing protein	N	N	N	N	N	N
WP_054171045.1	LysR family transcriptional regulator	N	N	N	N	N	N
WP_071305268.1	response regulator	N	N	N	N	N	N
WP_099559651.1	methylated-DNA--[protein]-cysteine S-methyltransferase	N	N	N	N	N	N
WP_099559933.1	CmlA/FloR family chloramphenicol efflux MFS transporter	N	N	N	N	N	N
WP_109814228.1	heavy metal response regulator transcription factor	N	N	N	N	N	N
WP_109815165.1	SMR family transporter	N	N	N	N	N	N
WP_125437589.1	GNAT family N-acetyltransferase	N	N	N	N	N	N
WP_126560114.1	TonB-dependent receptor	N	N	N	N	N	N
WP_159359682.1	TrbI/VirB10 family protein	N	N	N	N	N	N
WP_159359760.1	ABC transporter permease [Stenotrophomonas sp. GD03958]	N	N	N	N	N	N

WP_159359850.1	tautomerase family protein	N	N	N	N	N	N
WP_159359859.1	response regulator transcription factor	N	N	N	N	N	N
WP_159359912.1	GyrI-like domain-containing protein	N	N	N	N	N	N
WP_159359917.1	cellulase family glycosylhydrolase	N	N	N	N	N	N
WP_159359924.1	EamA family transporter	N	N	N	N	N	N
WP_159359966.1	carboxymuconolactone decarboxylase family protein	N	N	N	N	N	N
WP_159359978.1	ABC transporter ATP-binding protein	N	N	N	N	N	N
WP_159359990.1	thioesterase family protein	N	N	N	N	N	N
WP_159359992.1	ATP-binding protein	N	N	N	N	N	N
WP_159360053.1	SRPBCC domain-containing protein	N	N	N	N	N	N
WP_159360066.1	dihydrodipicolinate synthase family protein	N	N	N	N	N	N
WP_159360147.1	aldehyde oxidoreductase molybdenum-binding subunit PaoC	N	N	N	N	N	N
WP_159360149.1	aldehyde dehydrogenase iron-sulfur subunit PaoA	N	N	N	N	N	N

WP_159360150.1	nuclear transport factor 2 family protein	N	N	N	N	N	N
WP_159360151.1	TetR family transcriptional regulator	N	N	N	N	N	N
WP_159360188.1	hypothetical protein	N	N	N	N	N	N
WP_159360194.1	ABC transporter six-transmembrane domain-containing protein	N	N	N	N	N	N
WP_159360196.1	TonB-dependent siderophore receptor	N	N	N	N	N	N
WP_159360229.1	2,5-didehydrogluconate reductase DkgB	N	N	N	N	N	N
WP_159360230.1	cyclase family protein	N	N	N	N	N	N
WP_159360255.1	nitrate/nitrite transporter	N	N	N	N	N	N
WP_159360258.1	biotin/lipoyl-binding protein	N	N	N	N	N	N
WP_159360274.1	MgtC/SapB family protein	N	N	N	N	N	N
WP_159360325.1	NADH:flavin oxidoreductase/NADH oxidase family protein	N	N	N	N	N	N
WP_159360328.1	FAD-dependent monooxygenase	N	N	N	N	N	N
WP_159360351.1	agmatine deiminase family protein	N	N	N	N	N	N

WP_159360401.1	sugar phosphate isomerase/epimerase family protein	N	N	N	N	N	N
WP_159360450.1	VOC family protein	N	N	N	N	N	N
WP_159360533.1	L-seryl-tRNA(Sec) selenium transferase	N	N	N	N	N	N
WP_159360534.1	selenocysteine-specific translation elongation factor	N	N	N	N	N	N
WP_159360535.1	selenide, water dikinase SelD	N	N	N	N	N	N
WP_159360570.1	LysR family transcriptional regulator	N	N	N	N	N	N
WP_159360575.1	hydrolase	N	N	N	N	N	N
WP_159360596.1	Gfo/Idh/MocA family oxidoreductase	N	N	N	N	N	N
WP_159360615.1	MFS transporter	N	N	N	N	N	N
WP_159360692.1	L-dopachrome tautomerase-related protein	N	N	N	N	N	N
WP_159360783.1	ATP-binding protein	N	N	N	N	N	N
WP_201448452.1	hypothetical protein	N	N	N	N	N	N
WP_201448461.1	nucleoside triphosphatase NudI	N	N	N	N	N	N

	WP_219625843.1	DoxX family protein	N	N	N	N	N	N
	WP_227200592.1	LysR family transcriptional regulator	N	N	N	N	N	N
	WP_235652565.1	hypothetical protein	N	N	N	N	N	N
	WP_236565129.1	HipA N-terminal domain-containing protein	N	N	N	N	N	N
	WP_025874361.1	Lrp/AsnC family transcriptional regulator	N	N	N	N	N	N
	WP_025876304.1	phosphotyrosine protein phosphatase	N	N	N	N	N	N
	WP_025877422.1	cytochrome o ubiquinol oxidase subunit IV	N	N	N	N	N	N
	WP_025878014.1	acyl carrier protein	N	N	N	N	N	N
NZ_CP049368.1	WP_025878601.1	hypothetical protein	N	N	N	N	N	N
	WP_033831264.1	AzlD family protein	N	N	N	N	N	N
	WP_089235668.1	ABC transporter permease	N	N	N	N	N	N
	WP_089235818.1	biopolymer transporter ExbD	N	N	N	N	N	N
	WP_089235844.1	respiratory nitrate reductase subunit gamma	N	N	N	N	N	N

WP_089235909.1	MgtC/SapB family protein	N	N	N	N	N	N
WP_089236876.1	transcriptional regulator	N	N	N	N	N	N
WP_089237406.1	PDDEXK nuclease domain-containing protein	N	N	N	N	N	N
WP_089238596.1	ABC transporter ATP-binding protein	N	N	N	N	N	N
WP_089239702.1	EamA family transporter	N	N	N	N	N	N
WP_089240303.1	alpha/beta hydrolase	N	N	N	N	N	N
WP_099818458.1	LysR family transcriptional regulator	N	N	N	N	N	N
WP_100462647.1	LysR family transcriptional regulator	N	N	N	N	N	N
WP_100464099.1	response regulator	N	N	N	N	N	N
WP_100465559.1	MotA/TolQ/ExbB proton channel family protein	N	N	N	N	N	N
WP_100552412.1	Cu(I)-responsive transcriptional regulator	N	N	N	N	N	N
WP_108265102.1	type II toxin-antitoxin system RelE/ParE family toxin	N	N	N	N	N	N
WP_165375951.1	VOC family protein	N	N	N	N	N	N

WP_165376120.1	RNA 2'-phosphotransferase	N	N	N	N	N	N
WP_165376150.1	MFS transporter	N	N	N	N	N	N
WP_165376181.1	winged helix-turn-helix domain-containing protein	N	N	N	N	N	N
WP_165376197.1	UDP-glucose 4-epimerase GalE	N	N	N	N	N	N
WP_165376374.1	flavin reductase family protein	N	N	N	N	N	N
WP_165376408.1	phage tail sheath subtilisin-like domain-containing protein	N	N	NZ_CP049368.1-2	100	N	N
WP_165376515.1	GNAT family N-acetyltransferase	N	N	N	N	N	N
WP_165376518.1	helix-turn-helix transcriptional regulator	N	N	N	N	N	N
WP_165376577.1	GyrI-like domain-containing protein	N	N	N	N	N	N
WP_165376633.1	aminotransferase class III-fold pyridoxal phosphate-dependent enzyme	N	N	N	N	N	N
WP_165376636.1	DMT family transporter	N	N	N	N	N	N
WP_165376781.1	ABC transporter ATP-binding protein	N	N	N	N	N	N
WP_165376787.1	ATP-binding protein	N	N	N	N	N	N

WP_165376880.1	GNAT family N-acetyltransferase	N	N	N	N	N	N
WP_165376953.1	LysR family transcriptional regulator	N	N	N	N	N	N
WP_165376954.1	hypothetical protein	N	N	N	N	N	N
WP_165376957.1	TetR family transcriptional regulator	N	N	N	N	N	N
WP_165376958.1	O-methyltransferase	N	N	N	N	N	N
WP_165376961.1	metalloregulator ArsR/SmtB family transcription factor	N	N	N	N	N	N
WP_165376962.1	DUF2938 domain-containing protein	N	N	N	N	N	N
WP_165377046.1	transcriptional regulator BetI	N	N	N	N	N	N
WP_165377047.1	BCCT family transporter	N	N	N	N	N	N
WP_165377137.1	ABC transporter ATP-binding protein	N	N	N	N	N	N
WP_165377145.1	NADPH-dependent FMN reductase	N	N	N	N	N	N
WP_165377150.1	beta-ketoacyl-ACP synthase II	N	N	N	N	N	N
WP_165377155.1	cyclophilin-like fold protein	N	N	N	N	N	N

WP_165377158.1	LysR family transcriptional regulator	N	N	N	N	N	N
WP_165377159.1	TetR family transcriptional regulator	N	N	N	N	N	N
WP_165377162.1	NADP-dependent oxidoreductase	N	N	N	N	N	N
WP_165377167.1	MFS transporter	N	N	N	N	N	N
WP_165377168.1	AraC family transcriptional regulator	N	N	N	N	N	N
WP_165377177.1	aldo/keto reductase	N	N	N	N	N	N
WP_165377178.1	LysR family transcriptional regulator	N	N	N	N	N	N
WP_165377187.1	ATP-binding protein	N	N	N	N	N	N
WP_165377209.1	cytochrome c	N	N	N	N	N	N
WP_165377210.1	GMC family oxidoreductase	N	N	N	N	N	N
WP_165377220.1	hypothetical protein	N	N	N	N	N	N
WP_165377243.1	PepSY-associated TM helix domain-containing protein	N	N	N	N	N	N
WP_165377294.1	DUF2149 domain-containing protein	N	N	N	N	N	N

WP_165377295.1	LysR family transcriptional regulator	N	N	N	N	N	N
WP_165377314.1	type II secretion system major pseudopilin GspG	N	N	N	N	N	N
WP_165377325.1	nitrate/nitrite transporter [Stenotrophomonas lactitubi]	N	N	N	N	N	N
WP_165377328.1	NarK family nitrate/nitrite MFS transporter	N	N	N	N	N	N
WP_165377339.1	helix-turn-helix transcriptional regulator	N	N	N	N	N	N
WP_165377374.1	VOC family protein	N	N	N	N	N	N
WP_165377448.1	RidA family protein	N	N	N	N	N	N
WP_165377470.1	DUF2239 family protein	N	N	N	N	N	N
WP_165377495.1	FAD-dependent monooxygenase	N	N	N	N	N	N
WP_165377498.1	response regulator transcription factor	N	N	N	N	N	N
WP_165377548.1	agmatine deiminase family protein	N	N	N	N	N	N
WP_165377549.1	LysR family transcriptional regulator	N	N	N	N	N	N
WP_165377560.1	LysR family transcriptional regulator	N	N	N	N	N	N

WP_165377561.1	zinc-dependent alcohol dehydrogenase family protein	N	N	N	N	N	N
WP_165377681.1	TetR/AcrR family transcriptional regulator	N	N	N	N	N	N
WP_165377683.1	alpha/beta hydrolase	N	N	N	N	N	N
WP_165377917.1	response regulator transcription factor	N	N	N	N	N	N
WP_165377985.1	alpha/beta hydrolase	N	N	N	N	N	N
WP_165377986.1	helix-turn-helix transcriptional regulator	N	N	N	N	N	N
WP_165378123.1	LTA synthase family protein	N	N	N	N	N	N
WP_165378128.1	alcohol dehydrogenase catalytic domain-containing protein	N	N	N	N	N	N
WP_165378149.1	DHA2 family efflux MFS transporter permease subunit	N	N	N	N	N	N
WP_165378181.1	CmlA/FloR family chloramphenicol efflux MFS transporter	N	N	N	N	N	N
WP_165378183.1	Ohr family peroxiredoxin	N	N	N	N	N	N
WP_165378184.1	SDR family oxidoreductase	N	N	N	N	N	N
WP_165378185.1	LysR family transcriptional regulator	N	N	N	N	N	N

WP_165378187.1	carboxymuconolactone decarboxylase family protein	N	N	N	N	N	N
WP_165378188.1	cupin domain-containing protein	N	N	N	N	N	N
WP_165378189.1	RNA polymerase sigma-70 factor	N	N	N	N	N	N
WP_165378191.1	MBL fold metallo-hydrolase	N	N	N	N	N	N
WP_165378200.1	LysR family transcriptional regulator	N	N	N	N	N	N
WP_165378231.1	LLM class oxidoreductase	N	N	N	N	N	N
WP_165378232.1	glutathione S-transferase family protein	N	N	N	N	N	N
WP_165378233.1	LysR substrate-binding domain-containing protein	N	N	N	N	N	N
WP_165378345.1	cytochrome o ubiquinol oxidase subunit III	N	N	N	N	N	N
WP_165378575.1	DNA-binding protein	N	N	N	N	N	N
WP_207233999.1	helix-turn-helix transcriptional regulator	N	N	N	N	N	N
WP_207234007.1	AMP-binding protein	N	N	N	N	N	N
WP_207234017.1	glycosyltransferase family 2 protein	N	N	N	N	N	N

	WP_207234036.1	LLM class flavin-dependent oxidoreductase	N	N	N	N	N	N
	WP_242629851.1	alpha/beta fold hydrolase	N	N	N	N	N	N
	WP_242629908.1	NADAR family protein	N	N	N	N	N	N
	WP_242629914.1	LysR family transcriptional regulator	N	N	N	N	N	N
	WP_242629976.1	helix-turn-helix domain-containing protein	N	N	N	N	N	N
	WP_242629979.1	cellulase family glycosylhydrolase	N	N	N	N	N	N
	WP_242630000.1	NAD(P)H-dependent oxidoreductase	N	N	N	N	N	N
	WP_000483554.1	peptide-methionine (R)-S-oxide reductase MsrB	N	N	N	N	N	N
	WP_003050225.1	TIGR03758 family integrating conjugative element protein	N	N	N	N	N	N
	WP_003050245.1	helix-turn-helix domain-containing protein	N	N	N	N	N	N
NZ_CP049956.1	WP_003050256.1	AAA family ATPase	N	N	N	N	N	N
	WP_003050273.1	CBASS effector endonuclease NucC	N	N	N	N	N	N
	WP_003050422.1	hypothetical protein	N	N	N	N	N	N

WP_003090093.1	AlpA family transcriptional regulator	N	N	N	N	N	N
WP_003090097.1	DUF2857 domain-containing protein	N	N	N	N	N	N
WP_003090159.1	type III CBASS phage resistance system CD-NTase-associated protein Cap7	N	N	N	N	N	N
WP_003090167.1	RAQPRD family integrative conjugative element protein	N	N	N	N	N	N
WP_003098886.1	TIGR03759 family integrating conjugative element protein	N	N	N	N	N	N
WP_003098890.1	PilL N-terminal domain-containing protein	N	N	N	N	N	N
WP_003098923.1	hypothetical protein	N	N	N	N	N	N
WP_003098932.1	mercury(II) reductase	N	N	N	N	N	N
WP_003098939.1	Hg(II)-responsive transcriptional regulator	N	N	N	N	N	N
WP_003098941.1	hypothetical protein	N	N	N	N	N	N
WP_003098955.1	signal peptidase II	N	N	N	N	N	N
WP_003098961.1	heavy metal translocating P-type ATPase	N	N	N	N	N	N
WP_003098965.1	Cd(II)/Pb(II)-responsive transcriptional regulator	N	N	N	N	N	N

WP_003098972.1	cation transporter	N	N	N	N	N	N
WP_003098976.1	single-stranded DNA-binding protein	N	N	N	N	N	N
WP_003098978.1	DUF3158 family protein	N	N	N	N	N	N
WP_003098988.1	ParA family protein	NZ_CP060023.1-2	100	N	N	N	N
WP_003098996.1	DUF305 domain-containing protein	N	N	N	N	N	N
WP_003109779.1	integrating conjugative element protein	N	N	N	N	N	N
WP_003109780.1	transglycosylase SLT domain-containing protein	N	N	N	N	N	N
WP_003116819.1	DUF6094 domain-containing protein	N	N	N	N	N	N
WP_003116820.1	hypothetical protein	N	N	N	N	N	N
WP_003116821.1	hypothetical protein	N	N	N	N	N	N
WP_003116823.1	DUF3275 family protein	N	N	N	N	N	N
WP_003116824.1	DUF932 domain-containing protein	N	N	N	N	N	N
WP_003116825.1	DUF3577 domain-containing protein	N	N	N	N	N	N

WP_003116826.1	mercury resistance system periplasmic binding protein MerP	N	N	N	N	N	N
WP_003116827.1	mercuric ion transporter MerT	N	N	N	N	N	N
WP_003116829.1	hypothetical protein	N	N	N	N	N	N
WP_003116832.1	hypothetical protein	N	N	N	N	N	N
WP_003116836.1	TIGR03761 family integrating conjugative element protein	N	N	N	N	N	N
WP_003116837.1	STY4528 family pathogenicity island replication protein	N	N	N	N	N	N
WP_003153638.1	type III CBASS phage resistance system CD-NTase-associated protein Cap8	N	N	N	N	N	N
WP_003153640.1	CBASS oligonucleotide cyclase	N	N	N	N	N	N
WP_003153642.1	AAA family ATPase	N	N	N	N	N	N
WP_003153644.1	TIGR03747 family integrating conjugative element membrane protein	N	N	N	N	N	N
WP_003464969.1	glutathione S-transferase N-terminal domain-containing protein	N	N	N	N	N	N
WP_003821113.1	TIGR03752 family integrating conjugative element protein	N	N	N	N	N	N
WP_003821118.1	JAB domain-containing protein	N	N	N	N	N	N

WP_003821119.1	TIGR03757 family integrating conjugative element protein	N	N	N	N	N	N
WP_003821123.1	integrating conjugative element protein	N	N	N	N	N	N
WP_003821125.1	hypothetical protein	N	N	N	N	N	N
WP_003821130.1	MobH family relaxase	N	N	N	N	N	N
WP_003821132.1	glutaredoxin family protein	N	N	N	N	N	N
WP_003821135.1	FadR/GntR family transcriptional regulator	N	N	N	N	N	N
WP_003821152.1	metalloregulator ArsR/SmtB family transcription factor	N	N	N	N	N	N
WP_003821153.1	cation diffusion facilitator family transporter	N	N	N	N	N	N
WP_003821155.1	MFS transporter	N	N	N	N	N	N
WP_003821156.1	metal-sensing transcriptional repressor	N	N	N	N	N	N
WP_003821159.1	HlyD family efflux transporter periplasmic adaptor subunit	N	N	N	N	N	N
WP_004350508.1	ParB family protein	N	N	N	N	N	N
WP_005408112.1	LysR family transcriptional regulator	N	N	N	N	N	N

WP_005411473.1	cytochrome o ubiquinol oxidase subunit IV	N	N	N	N	N	N
WP_012479055.1	ABC transporter permease	N	N	N	N	N	N
WP_012479056.1	ABC transporter ATP-binding protein	N	N	N	N	N	N
WP_012479078.1	glycosyltransferase	N	N	N	N	N	N
WP_012480368.1	TIGR03885 family FMN-dependent LLM class oxidoreductase	N	N	N	N	N	N
WP_012614046.1	tyrosine-type recombinase/integrase	N	N	N	N	N	N
WP_012614048.1	LysR family transcriptional regulator	N	N	N	N	N	N
WP_012614056.1	efflux RND transporter permease subunit	N	N	N	N	N	N
WP_012614060.1	heavy metal translocating P-type ATPase	N	N	N	N	N	N
WP_012614066.1	RES family NAD+ phosphorylase	N	N	N	N	N	N
WP_012614069.1	thioredoxin domain-containing protein	N	N	N	N	N	N
WP_012614071.1	TIGR03746 family integrating conjugative element protein	N	N	N	N	N	N
WP_015912826.1	metalloregulator ArsR/SmtB family transcription factor	N	N	N	N	N	N

WP_015912828.1	hypothetical protein	N	N	N	N	N	N
WP_019416316.1	PIN domain-containing protein	N	N	N	N	N	N
WP_021204989.1	MFS transporter	N	N	N	N	N	N
WP_023103964.1	DUF736 domain-containing protein	N	N	N	N	N	N
WP_023127547.1	EexN family lipoprotein	N	N	N	N	N	N
WP_023127548.1	LysR family transcriptional regulator	N	N	N	N	N	N
WP_023127558.1	S26 family signal peptidase	N	N	N	N	N	N
WP_023127560.1	hypothetical protein	N	N	N	N	N	N
WP_023127562.1	helix-turn-helix domain-containing protein	N	N	N	N	N	N
WP_024082527.1	helix-turn-helix transcriptional regulator	N	N	N	N	N	N
WP_024956822.1	helix-turn-helix domain-containing protein	N	N	N	N	N	N
WP_024956907.1	LysR substrate-binding domain-containing protein	N	N	N	N	N	N
WP_031629745.1	DUF3085 domain-containing protein	N	N	N	N	N	N

WP_033979860.1	P-type conjugative transfer protein TrbL	N	N	N	N	N	N
WP_033979861.1	hypothetical protein	N	N	N	N	N	N
WP_033979862.1	P-type conjugative transfer protein TrbJ	N	N	N	N	N	N
WP_033979867.1	TrbC/VirB2 family protein	N	N	N	N	N	N
WP_047289083.1	hypothetical protein	N	N	N	N	N	N
WP_047289092.1	DUF2958 domain-containing protein	N	N	N	N	N	N
WP_047289094.1	DNA repair protein RadC	N	N	N	N	N	N
WP_047289095.1	AlpA family phage regulatory protein	N	N	N	N	N	N
WP_047290279.1	TrbI/VirB10 family protein	N	N	N	N	N	N
WP_047290294.1	hypothetical protein	N	N	N	N	N	N
WP_053092058.1	Clp protease ClpP	NZ_CP049956.1-2	100	N	N	N	N
WP_053506162.1	nucleotidyl transferase AbiEii/AbiGii toxin family protein	N	N	N	N	N	N
WP_053506169.1	hypothetical protein	N	N	N	N	N	N

WP_053506340.1	DUF2958 domain-containing protein	N	N	N	N	N	N
WP_053506343.1	SNF2-related protein	N	N	N	N	N	N
WP_053506344.1	hypothetical protein	N	N	N	N	N	N
WP_053506350.1	helix-turn-helix transcriptional regulator	N	N	N	N	N	N
WP_062607032.1	nucleoside triphosphatase NudI	N	N	N	N	N	N
WP_069953511.1	bleomycin binding protein Ble-MBL	N	N	N	N	N	N
WP_079859790.1	phosphoribosylanthranilate isomerase	N	N	N	N	N	N
WP_099485177.1	amino acid ABC transporter permease/ATP-binding protein	N	N	N	N	N	N
WP_121335901.1	IS5 family transposase	N	N	N	N	N	N
WP_137138279.1	DUF1156 domain-containing protein	N	N	N	N	N	N
WP_150359739.1	magnesium and cobalt transport protein CorA	N	N	N	N	N	N
WP_150359743.1	IS5 family transposase	N	N	N	N	N	N
WP_165710232.1	GPW/gp25 family protein	N	N	N	N	N	N

WP_165710334.1	TIGR03751 family conjugal transfer lipoprotein	N	N	N	N	N	N
WP_165710335.1	TIGR03749 family integrating conjugative element protein	N	N	N	N	N	N
WP_165710336.1	TIGR03750 family conjugal transfer protein	N	N	N	N	N	N
WP_165710337.1	TIGR03745 family integrating conjugative element membrane protein	N	N	N	N	N	N
WP_165710338.1	hypothetical protein	N	N	N	N	N	N
WP_165710372.1	DUF2285 domain-containing protein	N	N	N	N	N	N
WP_165710373.1	DUF2840 domain-containing protein	N	N	N	N	N	N
WP_165710374.1	DUF499 domain-containing protein	N	N	N	N	N	N
WP_165710376.1	calcium/sodium antiporter	N	N	N	N	N	N
WP_165710383.1	HsdR family type I site-specific deoxyribonuclease	N	N	N	N	N	N
WP_165710457.1	SIR2 family protein	NZ_CP049956.1-1	100	N	N	N	N
WP_165710571.1	phage major capsid protein	NZ_CP049956.1-2	100	N	N	N	N
WP_165711208.1	DUF488 domain-containing protein	N	N	N	N	N	N

	WP_165711248.1	glycosyltransferase family 2 protein	N	N	N	N	N	N
	WP_165711249.1	SMR family transporter	N	N	N	N	N	N
	WP_165711251.1	FAD-dependent oxidoreductase	N	N	N	N	N	N
	WP_172694150.1	transposase	N	N	N	N	N	N
	WP_197599535.1	NAD(P)-dependent oxidoreductase	N	N	N	N	N	N
	WP_199522034.1	type IV toxin-antitoxin system AbiEi family antitoxin	N	N	N	N	N	N
	WP_219627573.1	lysozyme	N	N	N	N	N	N
	WP_223656233.1	hypothetical protein	N	N	N	N	N	N
	WP_223846491.1	transposase	N	N	N	N	N	N
	WP_227415191.1	nuclear transport factor 2 family protein	N	N	N	N	N	N
	WP_231914652.1	SDR family oxidoreductase	N	N	N	N	N	N
NZ_CP051467.1	WP_000761850.1	organomercurial lyase MerB	N	N	N	N	N	N
	WP_000995361.1	mercury resistance co-regulator MerD	N	N	N	N	N	N

WP_003830788.1	DDE-type integrase/transposase/recombinase	N	N	N	N	N	N
WP_003830789.1	TniB family NTP-binding protein	N	N	N	N	N	N
WP_005408332.1	GPW/gp25 family protein	N	N	N	N	N	N
WP_005409675.1	AraC family transcriptional regulator	N	N	N	N	N	N
WP_005411473.1	cytochrome o ubiquinol oxidase subunit IV	N	N	N	N	N	N
WP_005412377.1	BPL-N domain-containing protein	N	N	N	N	N	N
WP_005413073.1	P-type conjugative transfer protein TrbL	N	N	N	N	N	N
WP_005413074.1	hypothetical protein	N	N	N	N	N	N
WP_005413075.1	P-type conjugative transfer protein TrbJ	N	N	N	N	N	N
WP_005413084.1	SDR family oxidoreductase	N	N	N	N	N	N
WP_005413088.1	S26 family signal peptidase	N	N	N	N	N	N
WP_005413093.1	helix-turn-helix domain-containing protein	N	N	N	N	N	N
WP_005413094.1	DUF2285 domain-containing protein	N	N	N	N	N	N

WP_005413100.1	DUF2958 domain-containing protein	N	N	N	N	N	N
WP_005413102.1	hypothetical protein	N	N	N	N	N	N
WP_005413103.1	DEAD/DEAH box helicase	N	N	N	N	N	N
WP_005413104.1	Qat anti-phage system ATPase QatA	N	N	N	N	N	N
WP_005413106.1	Qat anti-phage system QueC-like protein QatC	N	N	N	N	N	N
WP_005413389.1	TniQ family protein	N	N	N	N	N	N
WP_005413391.1	DUF3330 domain-containing protein	N	N	N	N	N	N
WP_005413392.1	broad-spectrum mercury transporter MerE	N	N	N	N	N	N
WP_005413394.1	organomercurial transporter MerC	N	N	N	N	N	N
WP_005413396.1	mercury resistance system periplasmic binding protein MerP	N	N	N	N	N	N
WP_005413401.1	helix-turn-helix transcriptional regulator	N	N	N	N	NZ_CP043578.1-2	100
WP_005413453.1	hypothetical protein	N	N	N	N	N	N
WP_005413455.1	SDR family oxidoreductase	N	N	N	N	N	N

WP_005414259.1	hypothetical protein	N	N	N	N	N	N
WP_005414736.1	DUF1629 domain-containing protein	N	N	N	N	N	N
WP_005414746.1	HlyD family efflux transporter periplasmic adaptor subunit	N	N	N	N	N	N
WP_005414747.1	ATP-binding cassette domain-containing protein	N	N	N	N	N	N
WP_017355541.1	MFS transporter	N	N	N	N	N	N
WP_017355546.1	efflux RND transporter periplasmic adaptor subunit	N	N	N	N	N	N
WP_026070691.1	XRE family transcriptional regulator	N	N	N	N	N	N
WP_031269107.1	DUF305 domain-containing protein	N	N	N	N	N	N
WP_032966054.1	helix-turn-helix domain-containing protein	N	N	N	N	N	N
WP_032966379.1	DNA cytosine methyltransferase	N	N	N	N	N	N
WP_032966429.1	nucleotidyl transferase AbiEii/AbiGii toxin family protein	N	N	N	N	N	N
WP_032966544.1	nucleotide pyrophosphohydrolase	N	N	N	N	N	N
WP_032966653.1	hypothetical protein	N	N	N	N	N	N

WP_032966655.1	GIY-YIG nuclease family protein	N	N	N	N	N	N
WP_032966658.1	hypothetical protein	N	N	N	N	N	N
WP_032966666.1	EexN family lipoprotein	N	N	N	N	N	N
WP_032966671.1	DoxX family protein	N	N	N	N	N	N
WP_032966672.1	DoxX family protein	N	N	N	N	N	N
WP_032966674.1	NAD(P)H-binding protein	N	N	N	N	N	N
WP_032966675.1	helix-turn-helix domain-containing protein	N	N	N	N	N	N
WP_032966679.1	hypothetical protein	N	N	N	N	N	N
WP_032966680.1	Qat anti-phage system associated protein QatB	N	N	N	N	N	N
WP_032966681.1	Qat anti-phage system TatD family nuclease QatD	N	N	N	N	N	N
WP_032966791.1	SDR family oxidoreductase	N	N	N	N	N	N
WP_032966794.1	aldehyde dehydrogenase family protein	N	N	N	N	N	N
WP_032967187.1	metalloregulator ArsR/SmtB family transcription factor	N	N	N	N	N	N

WP_032967276.1	hypothetical protein	N	N	N	N	N	N
WP_046272807.1	universal stress protein	NZ_CP051467.1-3	100	N	N	N	N
WP_050557873.1	MBL fold metallo-hydrolase	N	N	N	N	N	N
WP_061201506.1	phage exclusion protein Lit family protein	N	N	N	N	N	N
WP_061201507.1	hypothetical protein	N	N	N	N	N	N
WP_080101779.1	very short patch repair endonuclease	N	N	N	N	N	N
WP_080101801.1	helix-turn-helix transcriptional regulator	N	N	N	N	N	N
WP_088470467.1	hypothetical protein	N	N	N	N	N	N
WP_099497959.1	DnaT-like ssDNA-binding protein	N	N	N	N	N	N
WP_099497963.1	DUF1073 domain-containing protein	NZ_CP051467.1-1	100	N	N	N	N
WP_099497969.1	hypothetical protein	N	N	N	N	N	N
WP_099497990.1	DUF1643 domain-containing protein	N	N	N	N	N	N
WP_100442899.1	AAA family ATPase	N	N	N	N	N	N

	WP_168759593.1	DUF2184 domain-containing protein	NZ_CP051467.1-1	100	N	N	N	N
	WP_168759691.1	hypothetical protein	N	N	N	N	N	N
	WP_168759711.1	phage head closure protein	N	N	N	N	N	N
	WP_168759717.1	HK97 family phage prohead protease	NZ_CP051467.1-4	100	N	N	N	N
	WP_168759723.1	Gp49 family protein	N	N	N	N	N	N
	WP_168759728.1	RusA family crossover junction endodeoxyribonuclease	N	N	N	N	N	N
	WP_168759740.1	YqaJ viral recombinase family protein	N	N	N	N	N	N
	WP_232418880.1	SDR family oxidoreductase	N	N	N	N	N	N
	WP_232418907.1	Rid family hydrolase	N	N	N	N	N	N
	WP_232418980.1	AlpA family phage regulatory protein	N	N	N	N	N	N
	WP_241898613.1	phage terminase large subunit	NZ_CP051467.1-1	100	N	N	N	N
	WP_247650426.1	helicase-related protein	N	N	N	N	N	N
NZ_CP052863.1	WP_005408332.1	GPW/gp25 family protein	N	N	N	N	N	N

WP_019338230.1	SDR family oxidoreductase	N	N	N	N	N	N
WP_019338236.1	thioredoxin	N	N	N	N	N	N
WP_049449119.1	nuclear transport factor 2 family protein	N	N	N	N	N	N
WP_049449966.1	ABC transporter ATP-binding protein	N	N	N	N	N	N
WP_049449975.1	glycosyltransferase family 4 protein	N	N	N	N	N	N
WP_049449986.1	WxcM-like domain-containing protein	N	N	N	N	N	N
WP_049449988.1	glycosyltransferase family 2 protein	N	N	N	N	N	N
WP_049449989.1	DegT/DnrJ/EryC1/StrS family aminotransferase	N	N	N	N	N	N
WP_049450033.1	carboxymuconolactone decarboxylase family protein	N	N	N	N	N	N
WP_049450035.1	TetR/AcrR family transcriptional regulator	N	N	N	N	N	N
WP_059033628.1	OsmC family protein	N	N	N	N	N	N
WP_134708996.1	hypothetical protein	N	N	N	N	N	N
WP_169448303.1	DNA N-6-adenine-methyltransferase	N	N	N	N	N	N

	WP_169448459.1	amidohydrolase	N	N	N	N	N	N
	WP_169448603.1	AraC family transcriptional regulator	N	N	N	N	N	N
	WP_169448604.1	aldehyde dehydrogenase family protein	N	N	N	N	N	N
	WP_169448606.1	SDR family oxidoreductase	N	N	N	N	N	N
	WP_169448822.1	DUF1993 domain-containing protein	N	N	N	N	N	N
	WP_212577443.1	substrate binding domain-containing protein	N	N	N	N	N	N
	WP_212577444.1	hypothetical protein	N	N	N	N	N	N
	WP_212577448.1	integrase	NZ_CP052863.1-1	100	N	N	N	N
	WP_249335710.1	SDR family oxidoreductase	N	N	N	N	N	N
	WP_249335742.1	hypothetical protein	N	N	N	N	N	N
	WP_249335743.1	glycosyltransferase family 4 protein	N	N	N	N	N	N
	WP_283241139.1	DegT/DnrJ/EryC1/StrS family aminotransferase	N	N	N	N	N	N
NZ_CP056088.1	WP_003131974.1	mercuric ion transporter MerT	N	N	N	N	N	N

WP_003131987.1	mercury resistance system periplasmic binding protein MerP	N	N	N	N	N	N
WP_003156770.1	mercury(II) reductase	N	N	N	N	N	N
WP_005413072.1	conjugal transfer protein TrbF	N	N	N	N	N	N
WP_005413387.1	Hg(II)-responsive transcriptional regulator	N	N	N	N	N	N
WP_005413400.1	Tn3-like element TnAs1 family transposase	N	N	N	N	NZ_CP043578.1-2	100
WP_007182542.1	antibiotic biosynthesis monooxygenase	N	N	N	N	N	N
WP_008264906.1	HupE/UreJ family protein	N	N	N	N	N	N
WP_008266446.1	SDR family oxidoreductase	N	N	N	N	N	N
WP_008267238.1	MFS transporter	N	N	N	N	N	N
WP_010921730.1	recombinase family protein	N	N	N	N	N	N
WP_017514199.1	helix-turn-helix transcriptional regulator	N	N	N	N	N	N
WP_017514200.1	DUF736 domain-containing protein	N	N	N	N	N	N
WP_017514233.1	LysR family transcriptional regulator	N	N	N	N	N	N

WP_017514237.1	DUF190 domain-containing protein	N	N	N	N	N	N
WP_017514238.1	fluoride efflux transporter CrcB	N	N	N	N	N	N
WP_017514534.1	P-type conjugative transfer protein TrbG	N	N	N	N	N	N
WP_019726269.1	site-specific integrase	N	N	NZ_CP056088.1-3	100	N	N
WP_019726271.1	DNA repair protein RadC	N	N	N	N	N	N
WP_019726272.1	DUF2958 domain-containing protein	N	N	N	N	N	N
WP_019726276.1	ParB/RepB/Spo0J family partition protein	N	N	NZ_CP056088.1-3	100	N	N
WP_019726277.1	hypothetical protein	N	N	N	N	N	N
WP_019726278.1	DUF736 domain-containing protein	N	N	N	N	N	N
WP_019726279.1	helix-turn-helix transcriptional regulator	N	N	N	N	N	N
WP_019726280.1	DUF2958 domain-containing protein	N	N	N	N	N	N
WP_019726283.1	DUF2285 domain-containing protein	N	N	NZ_CP056088.1-3	100	N	N
WP_019726284.1	helix-turn-helix domain-containing protein	N	N	N	N	N	N

WP_019726285.1	replication initiator protein A	N	N	NZ_CP056088.1-3	100	N	N
WP_019726286.1	ParA family partition ATPase	N	N	NZ_CP056088.1-3	100	N	N
WP_019726289.1	S26 family signal peptidase	N	N	N	N	N	N
WP_019726306.1	P-type conjugative transfer protein TrbJ	N	N	N	N	N	N
WP_019726308.1	P-type conjugative transfer protein TrbL	N	N	N	N	N	N
WP_019726309.1	conjugal transfer protein TrbF	N	N	N	N	N	N
WP_019726310.1	P-type conjugative transfer protein TrbG	N	N	N	N	N	N
WP_019726311.1	TrbI/VirB10 family protein	N	N	N	N	N	N
WP_024569782.1	VirB3 family type IV secretion system protein	N	N	N	N	N	N
WP_024569787.1	Chromate resistance protein ChrB	N	N	N	N	N	N
WP_024957611.1	hypothetical protein	N	N	N	N	N	N
WP_024957960.1	VOC family protein	N	N	N	N	N	N
WP_024957961.1	Na+/H+ antiporter NhaA	N	N	N	N	N	N

WP_025981540.1	hypothetical protein	N	N	N	N	N	N
WP_025981541.1	DUF2840 domain-containing protein	N	N	N	N	N	N
WP_032959298.1	SRPBCC family protein	N	N	N	N	N	N
WP_034112276.1	VirB3 family type IV secretion system protein	N	N	N	N	N	N
WP_034112278.1	TrbC/VirB2 family protein	N	N	N	N	N	N
WP_034112282.1	CopG family transcriptional regulator	N	N	N	N	N	N
WP_034112290.1	LysR substrate-binding domain-containing protein	N	N	N	N	N	N
WP_034112294.1	cytochrome c biogenesis CcdA family protein	N	N	N	N	N	N
WP_034112296.1	carboxymuconolactone decarboxylase family protein	N	N	N	N	N	N
WP_034112300.1	mercury(II) reductase	N	N	N	N	N	N
WP_034112301.1	organomercurial transporter MerC	N	N	N	N	N	N
WP_034112302.1	mercury resistance system periplasmic binding protein MerP	N	N	N	N	N	N
WP_034112303.1	mercuric ion transporter MerT	N	N	N	N	N	N

WP_034112304.1	Hg(II)-responsive transcriptional regulator	N	N	N	N	N	N
WP_034112305.1	DUF302 domain-containing protein	N	N	N	N	N	N
WP_034112311.1	sigma-70 family RNA polymerase sigma factor	N	N	N	N	N	N
WP_035882038.1	Qat anti-phage system TatD family nuclease QatD	N	N	N	N	N	N
WP_035882039.1	Qat anti-phage system QueC-like protein QatC	N	N	N	N	N	N
WP_035882041.1	Qat anti-phage system ATPase QatA	N	N	N	N	N	N
WP_035882043.1	hypothetical protein	N	N	N	N	N	N
WP_035882045.1	DUF2958 domain-containing protein	N	N	N	N	N	N
WP_035882049.1	ParB/RepB/Spo0J family partition protein	N	N	N	N	N	N
WP_035882050.1	DUF2285 domain-containing protein	N	N	N	N	N	N
WP_035882051.1	helix-turn-helix domain-containing protein	N	N	N	N	N	N
WP_035882054.1	ParA family partition ATPase	N	N	N	N	N	N
WP_035882055.1	hypothetical protein	N	N	N	N	N	N

WP_035882056.1	DUF2840 domain-containing protein	N	N	N	N	N	N
WP_035882057.1	S26 family signal peptidase	N	N	N	N	N	N
WP_035882059.1	relaxase/mobilization nuclease and DUF3363 domain-containing protein	N	N	N	N	N	N
WP_035882061.1	voltage-gated chloride channel family protein	N	N	N	N	N	N
WP_035882064.1	phosphopyruvate hydratase	N	N	N	N	N	N
WP_035882067.1	EexN family lipoprotein	N	N	N	N	N	N
WP_035882069.1	ribbon-helix-helix protein, CopG family	N	N	N	N	N	N
WP_035882072.1	TrbC/VirB2 family protein	N	N	N	N	N	N
WP_035882074.1	P-type conjugative transfer protein TrbJ	N	N	N	N	N	N
WP_035882077.1	P-type conjugative transfer protein TrbL	N	N	N	N	N	N
WP_035882078.1	TrbI/VirB10 family protein	N	N	N	N	N	N
WP_035882154.1	DUF2958 domain-containing protein	N	N	N	N	N	N
WP_049431373.1	type II toxin-antitoxin system RelE/ParE family toxin	N	N	N	N	N	N

WP_049460453.1	helix-turn-helix domain-containing protein	N	N	N	N	N	N
WP_052229528.1	DoxX family protein	N	N	N	N	N	N
WP_052229529.1	DUF692 domain-containing protein	N	N	N	N	N	N
WP_072167338.1	arsenate reductase ArsC	N	N	N	N	N	N
WP_088479174.1	TonB-dependent receptor	N	N	N	N	N	N
WP_088479176.1	flavodoxin	N	N	N	N	N	N
WP_088479179.1	alpha/beta fold hydrolase	N	N	N	N	N	N
WP_088479182.1	divalent metal cation transporter	N	N	N	N	N	N
WP_088479202.1	YeeE/YedE family protein	N	N	N	N	N	N
WP_088479298.1	heavy metal response regulator transcription factor	N	N	N	N	N	N
WP_088479299.1	multidrug efflux RND transporter permease subunit	N	N	N	N	N	N
WP_088479334.1	sulfite exporter TauE/SafE family protein	N	N	N	N	N	N
WP_088479472.1	transmembrane anchor protein	N	N	N	N	N	N

WP_088479473.1	CusA/CzcA family heavy metal efflux RND transporter	N	N	N	N	N	N
WP_088479522.1	cytochrome o ubiquinol oxidase subunit IV	N	N	N	N	N	N
WP_088479546.1	phosphoadenosine phosphosulfate reductase family protein	N	N	N	N	N	N
WP_088479556.1	tyrosine-type recombinase/integrase	N	N	N	N	N	N
WP_088480104.1	IS3 family transposase	N	N	N	N	N	N
WP_088480360.1	ABC transporter permease	N	N	N	N	N	N
WP_088480428.1	DEAD/DEAH box helicase family protein	N	N	N	N	N	N
WP_088480608.1	alkene reductase	N	N	N	N	N	N
WP_088480852.1	glycosyltransferase	N	N	N	N	N	N
WP_088481019.1	phage tail sheath subtilisin-like domain-containing protein	NZ_CP056088.1-1	100	N	N	N	N
WP_100442933.1	SDR family oxidoreductase	N	N	N	N	N	N
WP_164084146.1	integrase core domain-containing protein	N	N	N	N	N	N
WP_164171609.1	AAA family ATPase	N	N	NZ_CP056088.1-4	100	N	N

	WP_164171613.1	DUF3164 family protein	N	N	NZ_CP056088.1-4	100	N	N
	WP_171013289.1	replication initiator protein A	N	N	N	N	N	N
	WP_223224753.1	SDR family oxidoreductase	N	N	N	N	N	N
	WP_223224789.1	SRPBCC domain-containing protein	N	N	N	N	N	N
	WP_236253126.1	DUF3422 family protein	N	N	N	N	N	N
	WP_236253127.1	IS3 family transposase	N	N	N	N	N	N
	WP_236253170.1	helicase-related protein	N	N	N	N	N	N
	WP_005407702.1	type II toxin-antitoxin system RelE/ParE family toxin	N	N	N	N	N	N
	WP_005407703.1	putative addiction module antidote protein	N	N	N	N	N	N
	WP_005407950.1	winged helix-turn-helix domain-containing protein	N	N	N	N	N	N
NZ_CP060021.1	WP_005408112.1	LysR family transcriptional regulator	N	N	N	N	N	N
	WP_005408494.1	helix-turn-helix transcriptional regulator	N	N	N	N	N	N
	WP_005408666.1	DMT family transporter	N	N	N	N	N	N

WP_005408850.1	carboxymuconolactone decarboxylase family protein	N	N	N	N	N	N
WP_005408917.1	SRPBCC family protein	N	N	N	N	N	N
WP_005409098.1	SRPBCC family protein	N	N	N	N	N	N
WP_005409356.1	metalloregulator ArsR/SmtB family transcription factor	N	N	N	N	N	N
WP_005409440.1	Cu(I)-responsive transcriptional regulator	N	N	N	N	N	N
WP_005409501.1	transcriptional regulator BetI	N	N	N	N	N	N
WP_005409502.1	BCCT family transporter	N	N	N	N	N	N
WP_005409726.1	ABC transporter permease	N	N	N	N	N	N
WP_005409730.1	LysR family transcriptional regulator	N	N	N	N	N	N
WP_005409793.1	sensory rhodopsin transducer	N	N	N	N	N	N
WP_005409850.1	response regulator	N	N	N	N	N	N
WP_005409874.1	biopolymer transporter ExbD	N	N	N	N	N	N
WP_005409898.1	AzlD family protein	N	N	N	N	N	N

WP_005409958.1	tetratricopeptide repeat protein	N	N	N	N	N	N
WP_005409984.1	MgtC/SapB family protein	N	N	N	N	N	N
WP_005410081.1	putative quinol monooxygenase	N	N	N	N	N	N
WP_005410104.1	helix-turn-helix domain-containing protein	N	N	N	N	N	N
WP_005410131.1	MerR family transcriptional regulator	N	N	N	N	N	N
WP_005410145.1	helix-turn-helix domain-containing protein	N	N	N	N	N	N
WP_005410442.1	VOC family protein	N	N	N	N	N	N
WP_005410881.1	formate dehydrogenase subunit beta	N	N	N	N	N	N
WP_005410882.1	formate dehydrogenase subunit gamma	N	N	N	N	N	N
WP_005411002.1	LysR family transcriptional regulator	N	N	N	N	N	N
WP_005411161.1	VOC family protein	N	N	N	N	N	N
WP_005411280.1	hypothetical protein	N	N	N	N	N	N
WP_005411594.1	hypothetical protein	N	N	N	N	N	N

WP_005411609.1	acyl carrier protein	N	N	N	N	N	N
WP_005411640.1	DNA polymerase III subunit epsilon	N	N	N	N	N	N
WP_005412840.1	LysR family transcriptional regulator	N	N	N	N	N	N
WP_005413494.1	ABC transporter ATP-binding protein	N	N	N	N	N	N
WP_005413603.1	TIGR03571 family LLM class oxidoreductase	N	N	N	N	N	N
WP_005413654.1	Lrp/AsnC family transcriptional regulator	N	N	N	N	N	N
WP_005414055.1	SMR family transporter	N	N	N	N	N	N
WP_005414661.1	DNA-binding transcriptional regulator	N	N	N	N	N	N
WP_010485313.1	respiratory nitrate reductase subunit gamma	N	N	N	N	N	N
WP_012478754.1	VOC family protein	N	N	N	N	N	N
WP_012479055.1	ABC transporter permease	N	N	N	N	N	N
WP_012479264.1	flavin reductase family protein	N	N	N	N	N	N
WP_012479298.1	phage tail sheath subtilisin-like domain-containing protein	NZ_CP022053.2-3	100	NZ_CP040431.1-3, NZ_CP044092.1-2,	100	NC_010943.1-6	100

				NZ_CP060021.1-2, NZ_CP060024.1-5, NZ_CP060027.1-2, NZ_CP098483.1-3, NZ_CP104290.1-1			
WP_012479302.1	baseplate J/gp47 family protein	N	N	N	N	N	N
WP_012479306.1	glycoside hydrolase family 104 protein	N	N	N	N	N	N
WP_012479322.1	LysR substrate-binding domain-containing protein	N	N	N	N	N	N
WP_012479522.1	GyrI-like domain-containing protein	N	N	N	N	N	N
WP_012479691.1	ABC transporter ATP-binding protein	N	N	N	N	N	N
WP_012479990.1	DNA-binding protein	N	N	N	N	N	N
WP_012480472.1	NarK family nitrate/nitrite MFS transporter	N	N	N	N	N	N
WP_012480491.1	helix-turn-helix transcriptional regulator	N	N	N	N	N	N
WP_012480618.1	DUF2239 family protein	N	N	N	N	N	N
WP_012481498.1	cytochrome o ubiquinol oxidase subunit III	N	N	N	N	N	N
WP_019659676.	response regulator	N	N	N	N	N	N

1							
WP_024957346.1	glycosyltransferase family 2 protein	N	N	N	N	N	N
WP_038646246.1	type II toxin-antitoxin system HipA family toxin	N	N	N	N	N	N
WP_043033286.1	nitrate/nitrite transporter	N	N	N	N	N	N
WP_043033321.1	hydrolase	N	N	N	N	N	N
WP_043033322.1	LysR family transcriptional regulator	N	N	N	N	N	N
WP_043033528.1	nucleoside triphosphatase NudI	N	N	N	N	N	N
WP_043034610.1	LysR substrate-binding domain-containing protein	N	N	N	N	N	N
WP_043035115.1	response regulator transcription factor	N	N	N	N	N	N
WP_043035594.1	metalloregulator ArsR/SmtB family transcription factor	N	N	N	N	N	N
WP_043035603.1	SDR family oxidoreductase	N	N	N	N	N	N
WP_044569589.1	glutathione S-transferase family protein	N	N	N	N	N	N
WP_049395605.1	PDDEXK nuclease domain-containing protein	N	N	N	N	N	N
WP_049396433.	MFS transporter	N	N	N	N	N	N

1							
WP_049396604.1	FAD-dependent monooxygenase	N	N	N	N	N	N
WP_049396610.1	NADH:flavin oxidoreductase/NADH oxidase family protein	N	N	N	N	N	N
WP_049450670.1	nuclear transport factor 2 family protein	N	N	N	N	N	N
WP_049450798.1	response regulator	N	N	N	N	N	N
WP_049459636.1	alpha/beta hydrolase	N	N	N	N	N	N
WP_060380234.1	aldehyde oxidoreductase molybdenum-binding subunit PaoC	N	N	N	N	N	N
WP_060381221.1	TetR family transcriptional regulator	N	N	N	N	N	N
WP_061201610.1	aldehyde dehydrogenase iron-sulfur subunit PaoA	NZ_CM001824.1-4	100	N	N	N	N
WP_062606330.1	DUF2938 domain-containing protein	N	N	N	N	N	N
WP_065183429.1	VOC family protein	N	N	N	N	N	N
WP_076738953.1	hypothetical protein	N	N	N	N	N	N
WP_076739576.1	response regulator	N	N	N	N	N	N
WP_081284579.	cyclase family protein	N	N	N	N	N	N

1							
WP_099473637.1	ATP-binding protein	N	N	N	N	N	N
WP_099474045.1	ATP-binding protein	N	N	N	N	N	N
WP_099474433.1	TIGR03885 family FMN-dependent LLM class oxidoreductase	N	N	N	N	N	N
WP_099484354.1	Gfo/Idh/MocA family oxidoreductase	N	N	N	N	N	N
WP_099523560.1	L-dopachrome tautomerase-related protein	N	N	N	N	N	N
WP_099523568.1	hypothetical protein	N	N	N	N	N	N
WP_099523646.1	type II secretion system major pseudopilin GspG	N	N	N	N	N	N
WP_099523681.1	sigma-70 family RNA polymerase sigma factor	N	N	N	N	N	N
WP_099523683.1	VOC family protein	N	N	N	N	N	N
WP_099523717.1	biotin/lipoyl-binding protein	N	N	N	N	N	N
WP_099523788.1	MFS transporter	N	N	N	N	N	N
WP_099524045.1	GNAT family N-acetyltransferase	N	N	N	N	N	N
WP_099524157.	UDP-N-acetylglucosamine 2-epimerase	N	N	N	N	N	N

1	(non-hydrolyzing)						
WP_099524647.1	LysE family transporter	N	N	N	N	N	N
WP_099524650.1	AraC family transcriptional regulator	N	N	N	N	N	N
WP_099524676.1	hypothetical protein	N	N	N	N	N	N
WP_099524798.1	Rid family hydrolase	N	N	N	N	N	N
WP_099524873.1	aminotransferase class III-fold pyridoxal phosphate-dependent enzyme	N	N	N	N	N	N
WP_099524875.1	EamA family transporter	N	N	N	N	N	N
WP_100438731.1	agmatine deiminase family protein	N	N	N	N	N	N
WP_111193373.1	L-seryl-tRNA(Sec) selenium transferase	N	N	N	N	N	N
WP_111193374.1	selenocysteine-specific translation elongation factor	N	N	N	N	N	N
WP_164105487.1	MFS transporter	N	N	N	N	N	N
WP_185807095.1	DNA adenine methylase	NZ_CP060021.1-1	100	N	N	N	N
WP_231347490.1	nuclear transport factor 2 family protein	N	N	N	N	N	N
WP_239502325.	GlxA family transcriptional regulator	N	N	N	N	N	N

	1							
	WP_239504153.1	helix-turn-helix domain-containing protein	N	N	N	N	N	N
	WP_239504154.1	NmrA family NAD(P)-binding protein	N	N	N	N	N	N
	WP_239504156.1	SDR family oxidoreductase	N	N	N	N	N	N
	WP_239504158.1	metalloregulator ArsR/SmtB family transcription factor	N	N	N	N	N	N
	WP_260618751.1	LysR substrate-binding domain-containing protein	N	N	N	N	N	N
	WP_003050225.1	TIGR03758 family integrating conjugative element protein	N	N	N	N	N	N
	WP_003050245.1	helix-turn-helix domain-containing protein	N	N	N	N	N	N
	WP_003050273.1	CBASS effector endonuclease NucC	N	N	N	N	N	N
NZ_CP060022.1	WP_003050422.1	hypothetical protein	N	N	N	N	N	N
	WP_003090093.1	AlpA family transcriptional regulator	N	N	N	N	N	N
	WP_003090097.1	DUF2857 domain-containing protein	N	N	N	N	N	N
	WP_003090159.1	type III CBASS phage resistance system CD-NTase-associated protein Cap7	N	N	N	N	N	N
	WP_003090173.	TIGR03745 family integrating	N	N	N	N	N	N

1	conjugative element membrane protein						
WP_003090202.1	thioredoxin domain-containing protein	N	N	N	N	N	N
WP_003090203.1	JAB domain-containing protein	N	N	N	N	N	N
WP_003090212.1	hypothetical protein	N	N	N	N	N	N
WP_003090216.1	DUF3742 family protein	N	N	N	N	N	N
WP_003090219.1	RES family NAD+ phosphorylase	N	N	N	N	N	N
WP_003090319.1	copper homeostasis periplasmic binding protein CopC	N	N	N	N	N	N
WP_003097498.1	Cu(+)/Ag(+) sensor histidine kinase	N	N	N	N	N	N
WP_003097522.1	copper homeostasis membrane protein CopD	N	N	N	N	N	N
WP_003097524.1	LysR family transcriptional regulator	N	N	N	N	N	N
WP_003097526.1	helix-turn-helix transcriptional regulator	N	N	N	N	N	N
WP_003098886.1	TIGR03759 family integrating conjugative element protein	N	N	N	N	N	N
WP_003098888.1	hypothetical protein	N	N	N	N	N	N
WP_003098890.	PilL N-terminal domain-containing	N	N	N	N	N	N

1	protein						
WP_003098947.1	hypothetical protein	N	N	N	N	N	N
WP_003098949.1	hypothetical protein	N	N	N	N	N	N
WP_003098955.1	signal peptidase II	N	N	N	N	N	N
WP_003098965.1	Cd(II)/Pb(II)-responsive transcriptional regulator	N	N	N	N	N	N
WP_003098972.1	cation transporter	N	N	N	N	N	N
WP_003098976.1	single-stranded DNA-binding protein	N	N	N	N	N	N
WP_003098978.1	DUF3158 family protein	N	N	N	N	N	N
WP_003098981.1	TIGR03761 family integrating conjugative element protein	N	N	N	N	N	N
WP_003098985.1	ParB family protein	NZ_CP060023.1-2	100	N	N	N	N
WP_003098988.1	ParA family protein	NZ_CP060023.1-2	100	N	N	N	N
WP_003098991.1	hypothetical protein	NZ_CP060023.1-2	100	N	N	N	N
WP_003098996.1	DUF305 domain-containing protein	N	N	N	N	N	N
WP_003105624.	CBASS oligonucleotide cyclase	N	N	N	N	N	N

1							
WP_003105626.1	type III CBASS phage resistance system CD-NTase-associated protein Cap8	N	N	N	N	N	N
WP_003105629.1	AAA family ATPase	N	N	N	N	N	N
WP_003105635.1	RAQPRD family integrative conjugative element protein	N	N	N	N	N	N
WP_003105639.1	TIGR03750 family conjugal transfer protein	N	N	N	N	N	N
WP_003105643.1	TIGR03749 family integrating conjugative element protein	N	N	N	N	N	N
WP_003107243.1	ArsI/CadI family heavy metal resistance metalloenzyme	N	N	N	N	N	N
WP_003108989.1	hypothetical protein	N	N	N	N	N	N
WP_003109689.1	efflux RND transporter periplasmic adaptor subunit	N	N	N	N	N	N
WP_003109690.1	TolC family protein	N	N	N	N	N	N
WP_003109695.1	arsenate reductase ArsC	N	N	N	N	N	N
WP_003109768.1	MobH family relaxase	N	N	N	N	N	N
WP_003109769.1	hypothetical protein	N	N	N	N	N	N
WP_003109772.	TIGR03757 family integrating	N	N	N	N	N	N

1	conjugative element protein						
WP_003109775.1	TIGR03751 family conjugal transfer lipoprotein	N	N	N	N	N	N
WP_003109777.1	PIN domain-containing protein	N	N	N	N	N	N
WP_003109778.1	TIGR03747 family integrating conjugative element membrane protein	N	N	N	N	N	N
WP_003109779.1	integrating conjugative element protein	N	N	N	N	N	N
WP_003109780.1	transglycosylase SLT domain-containing protein	N	N	N	N	N	N
WP_003116799.1	helix-turn-helix domain-containing protein	N	N	N	N	N	N
WP_003116812.1	AAA family ATPase	N	N	N	N	N	N
WP_005304583.1	helix-turn-helix transcriptional regulator	N	N	N	N	N	N
WP_005414984.1	MFS transporter	N	N	N	N	N	N
WP_005416648.1	RebB family R body protein	N	N	N	N	N	N
WP_005416649.1	RebB family R body protein	N	N	N	N	N	N
WP_005416651.1	RebB family R body protein	N	N	N	N	N	N
WP_006226009.	hypothetical protein	N	N	N	N	N	N

1							
WP_006226010.1	DUF3085 domain-containing protein	N	N	N	N	N	N
WP_006226011.1	DUF3577 domain-containing protein	N	N	N	N	N	N
WP_006226012.1	DUF3275 family protein	N	N	N	N	N	N
WP_006226013.1	hypothetical protein	N	N	N	N	N	N
WP_006226014.1	hypothetical protein	N	N	N	N	N	N
WP_006226241.1	DUF411 domain-containing protein	N	N	N	N	N	N
WP_006375900.1	zincin-like metallopeptidase domain-containing protein	N	N	N	N	N	N
WP_006399830.1	cytochrome o ubiquinol oxidase subunit IV	N	N	N	N	N	N
WP_006446865.1	RebB family R body protein	N	N	N	N	N	N
WP_008264646.1	STY4528 family pathogenicity island replication protein	N	N	N	N	N	N
WP_008266552.1	hypothetical protein	N	N	N	N	N	N
WP_012478642.1	UvrD-helicase domain-containing protein	N	N	N	N	N	N
WP_015014846.	PadR family transcriptional regulator	N	N	N	N	N	N

1							
WP_020200523.1	DUF6127 family protein	N	N	N	N	N	N
WP_023086160.1	copper resistance protein B	N	N	N	N	N	N
WP_023657320.1	copper resistance system multicopper oxidase	N	N	N	N	N	N
WP_024956711.1	DUF2958 domain-containing protein	N	N	N	N	N	N
WP_024956712.1	DUF2285 domain-containing protein	N	N	NZ_CP060022.1-9, NZ_CP060023.1-9, NZ_CP065965.1-5	100	N	N
WP_024956713.1	helix-turn-helix domain-containing protein	N	N	N	N	N	N
WP_024956717.1	DUF2840 domain-containing protein	N	N	NZ_CP060022.1-9, NZ_CP060023.1-9, NZ_CP065965.1-5	100	N	N
WP_024956718.1	S26 family signal peptidase	N	N	NZ_CP060022.1-9, NZ_CP060023.1-9, NZ_CP065965.1-5	100	N	N
WP_025566605.1	small heat shock protein sHSP20-GI	N	N	N	N	N	N
WP_031688306.1	hypothetical protein	N	N	N	N	N	N
WP_032957905.1	glycosyltransferase family 2 protein	N	N	N	N	N	N

WP_032963389.1	hypothetical protein	N	N	N	N	N	N
WP_034022740.1	hypothetical protein	NZ_CP060022.1-5, NZ_CP060023.1-3	100	N	N	N	N
WP_034022744.1	hypothetical protein	N	N	N	N	N	N
WP_034022749.1	major capsid protein	NZ_CP060022.1-5, NZ_CP060023.1-3	100	NZ_CP060022.1-6	100	N	N
WP_034022752.1	head decoration protein	N	N	N	N	N	N
WP_034022755.1	phage portal protein	NZ_CP060022.1-5, NZ_CP060023.1-3	100	NZ_CP060022.1-6, NZ_CP060023.1-8	100	N	N
WP_034022757.1	hypothetical protein	N	N	N	N	N	N
WP_034022758.1	hypothetical protein	N	N	N	N	N	N
WP_034022759.1	hypothetical protein	N	N	N	N	N	N
WP_034022762.1	DUF3489 domain-containing protein	N	N	NZ_CP060022.1-6, NZ_CP060023.1-8	100	N	N
WP_034022764.1	hypothetical protein	N	N	N	N	N	N
WP_034022766.1	hypothetical protein	N	N	N	N	N	N
WP_034022770.1	site-specific DNA-methyltransferase	N	N	NZ_CP060022.1-6, NZ_CP060023.1-8	100	N	N

WP_034022774.1	DUF6362 family protein	N	N	N	N	N	N
WP_034022775.1	hypothetical protein	N	N	N	N	N	N
WP_034022778.1	phage/plasmid primase, P4 family	N	N	NZ_CP060022.1-6, NZ_CP060023.1-8	100	N	N
WP_034022781.1	DUF6511 domain-containing protein	N	N	N	N	N	N
WP_034022783.1	hypothetical protein	N	N	NZ_CP060022.1-6, NZ_CP060023.1-8	100	N	N
WP_034022786.1	ATP-binding protein	N	N	NZ_CP060022.1-6, NZ_CP060023.1-8	100	N	N
WP_034022794.1	hypothetical protein	N	N	N	N	N	N
WP_034022798.1	DUF2924 domain-containing protein	N	N	N	N	N	N
WP_034022805.1	helix-turn-helix transcriptional regulator	N	N	N	N	N	N
WP_034022807.1	hypothetical protein	N	N	N	N	N	N
WP_034022810.1	hypothetical protein	N	N	N	N	N	N
WP_034022813.1	DUF2188 domain-containing protein	N	N	N	N	N	N
WP_034022819.1	phage terminase large subunit family protein	NZ_CP060022.1-5, NZ_CP060023.1-3	100	NZ_CP060022.1-6, NZ_CP060023.1-8	100	N	N

WP_034067943.1	Bro-N domain-containing protein	N	N	NZ_CP060022.1-6, NZ_CP060023.1-8	100	N	N
WP_034067946.1	isoleucyl-tRNA synthetase	N	N	NZ_CP060022.1-6, NZ_CP060023.1-8	100	N	N
WP_046427768.1	NADPH-dependent FMN reductase	N	N	N	N	N	N
WP_046428099.1	type II toxin-antitoxin system RelE/ParE family toxin [Stenotrophomonas sp. GD04064]	N	N	N	N	N	N
WP_046428102.1	HigA family addiction module antitoxin	N	N	N	N	N	N
WP_046428496.1	McrC family protein	N	N	N	N	N	N
WP_046428716.1	ABC transporter ATP-binding protein	N	N	N	N	N	N
WP_046430205.1	HsdR family type I site-specific deoxyribonuclease	N	N	N	N	N	N
WP_046430206.1	SprT family zinc-dependent metalloprotease	N	N	N	N	N	N
WP_046430563.1	metalloregulator ArsR/SmtB family transcription factor	N	N	N	N	N	N
WP_046430578.1	sodium:proton exchanger	N	N	N	N	N	N
WP_046430586.1	heavy metal translocating P-type ATPase	N	N	N	N	N	N
WP_046430608.	Tn3 family transposase	N	N	N	N	N	N

1							
WP_046432106.1	XRE family transcriptional regulator	N	N	N	N	N	N
WP_046432108.1	type IV toxin-antitoxin system AbiEi family antitoxin domain-containing protein	N	N	N	N	N	N
WP_046432110.1	nucleotidyl transferase AbiEii/AbiGii toxin family protein	N	N	N	N	N	N
WP_046432112.1	recombinase family protein	N	N	N	N	N	N
WP_046432114.1	ParB/RepB/Spo0J family partition protein	N	N	N	N	N	N
WP_046432116.1	plasmid partitioning protein RepB C-terminal domain-containing protein	N	N	N	N	N	N
WP_046432121.1	lysozyme	N	N	N	N	N	N
WP_046432123.1	hypothetical protein	N	N	N	N	N	N
WP_046432125.1	DUF2793 domain-containing protein	N	N	N	N	N	N
WP_046432152.1	recombinase family protein	N	N	NZ_CP060022.1-6, NZ_CP060023.1-8	100	N	N
WP_046432185.1	hypothetical protein	N	N	N	N	N	N
WP_046432224.1	zeta toxin family protein	N	N	N	N	N	N

WP_049244394.1	hypothetical protein	N	N	N	N	N	N
WP_049408527.1	heat resistance protein YfdX2	N	N	N	N	N	N
WP_049408528.1	heat resistance protein YfdX1	N	N	N	N	N	N
WP_049420578.1	helix-turn-helix domain-containing protein	N	N	N	N	N	N
WP_049420580.1	Hsp20/alpha crystallin family protein	N	N	N	N	N	N
WP_049420584.1	cardiolipin synthase	N	N	N	N	N	N
WP_049420590.1	HdeD family acid-resistance protein	N	N	N	N	N	N
WP_049420592.1	hypothetical protein	N	N	N	N	N	N
WP_072167338.1	arsenate reductase ArsC	N	N	N	N	N	N
WP_087786704.1	ISL3 family transposase	N	N	N	N	N	N
WP_087944428.1	IS3-like element ISStma9 family transposase	NC_010943.1-1	100	NZ_CP060023.1-10, NZ_CP060024.1-6, NZ_CP060022.1-10	100	N	N
WP_116825096.1	OST-HTH/LOTUS domain-containing protein	N	N	NZ_CP060022.1-6, NZ_CP060023.1-8	100	N	N
WP_141098655.	ABC transporter permease	N	N	N	N	N	N

1							
WP_143568593.1	hypothetical protein	N	N	N	N	N	N
WP_180839888.1	site-specific DNA-methyltransferase	N	N	N	N	N	N
WP_180839947.1	TIGR03885 family FMN-dependent LLM class oxidoreductase	N	N	N	N	N	N
WP_180839963.1	TIGR03752 family integrating conjugative element protein	N	N	N	N	N	N
WP_180839965.1	heavy metal translocating P-type ATPase	N	N	N	N	N	N
WP_180840055.1	very short patch repair endonuclease [Stenotrophomonas maltophilia group sp. msm4]	N	N	N	N	N	N
WP_180840060.1	DNA cytosine methyltransferase [Stenotrophomonas maltophilia group sp. msm4]	N	N	N	N	N	N
WP_180840062.1	GIY-YIG nuclease family protein [Stenotrophomonas maltophilia group sp. msm4]	N	N	N	N	N	N
WP_180840131.1	heat resistance system K+/H+ antiporter KefB-GI	N	N	N	N	N	N
WP_197596969.1	vitamin K epoxide reductase family protein	N	N	N	N	N	N
WP_197651614.1	ImmA/IrrE family metallo-endopeptidase	N	N	N	N	N	N

	WP_234600102.1	elements of external origin	N	N	N	N	N	N
	WP_254916320.1	DUF6094 domain-containing protein	N	N	N	N	N	N
	WP_258008290.1	ATP-binding protein	N	N	N	N	N	N
	WP_003050225.1	TIGR03758 family integrating conjugative element protein	N	N	N	N	N	N
	WP_003050245.1	helix-turn-helix domain-containing protein	N	N	N	N	N	N
	WP_003050273.1	CBASS effector endonuclease NucC	N	N	N	N	N	N
	WP_003050422.1	hypothetical protein	N	N	N	N	N	N
	WP_003090093.1	AlpA family transcriptional regulator	N	N	N	N	N	N
NZ_CP060023.1	WP_003090097.1	DUF2857 domain-containing protein	N	N	N	N	N	N
	WP_003090159.1	type III CBASS phage resistance system CD-NTase-associated protein Cap7	N	N	N	N	N	N
	WP_003090173.1	TIGR03745 family integrating conjugative element membrane protein	N	N	N	N	N	N
	WP_003090202.1	thioredoxin domain-containing protein	N	N	N	N	N	N
	WP_003090203.1	JAB domain-containing protein	N	N	N	N	N	N

WP_003090212.1	hypothetical protein	N	N	N	N	N	N
WP_003090216.1	DUF3742 family protein	N	N	N	N	N	N
WP_003090219.1	RES family NAD+ phosphorylase	N	N	N	N	N	N
WP_003090319.1	copper homeostasis periplasmic binding protein CopC	N	N	N	N	N	N
WP_003097498.1	Cu(+)/Ag(+) sensor histidine kinase	N	N	N	N	N	N
WP_003097522.1	copper homeostasis membrane protein CopD	N	N	N	N	N	N
WP_003097524.1	LysR family transcriptional regulator	N	N	N	N	N	N
WP_003097526.1	helix-turn-helix transcriptional regulator	N	N	N	N	N	N
WP_003098886.1	TIGR03759 family integrating conjugative element protein	N	N	N	N	N	N
WP_003098888.1	hypothetical protein	N	N	N	N	N	N
WP_003098890.1	PilL N-terminal domain-containing protein	N	N	N	N	N	N
WP_003098947.1	hypothetical protein	N	N	N	N	N	N
WP_003098949.1	hypothetical protein	N	N	N	N	N	N

WP_003098955.1	signal peptidase II	N	N	N	N	N	N
WP_003098965.1	Cd(II)/Pb(II)-responsive transcriptional regulator	N	N	N	N	N	N
WP_003098972.1	cation transporter	N	N	N	N	N	N
WP_003098976.1	single-stranded DNA-binding protein	N	N	N	N	N	N
WP_003098978.1	DUF3158 family protein	N	N	N	N	N	N
WP_003098981.1	TIGR03761 family integrating conjugative element protein	N	N	N	N	N	N
WP_003098985.1	ParB family protein	NZ_CP060023.1-2	100	N	N	N	N
WP_003098988.1	ParA family protein	NZ_CP060023.1-2	100	N	N	N	N
WP_003098991.1	hypothetical protein	NZ_CP060023.1-2	100	N	N	N	N
WP_003098996.1	DUF305 domain-containing protein	N	N	N	N	N	N
WP_003105624.1	CBASS oligonucleotide cyclase	N	N	N	N	N	N
WP_003105626.1	type III CBASS phage resistance system CD-NTase-associated protein Cap8	N	N	N	N	N	N
WP_003105629.1	AAA family ATPase	N	N	N	N	N	N

WP_003105635.1	RAQPRD family integrative conjugative element protein	N	N	N	N	N	N
WP_003105639.1	TIGR03750 family conjugal transfer protein	N	N	N	N	N	N
WP_003105643.1	TIGR03749 family integrating conjugative element protein	N	N	N	N	N	N
WP_003107243.1	ArsI/CadI family heavy metal resistance metalloenzyme	N	N	N	N	N	N
WP_003108989.1	hypothetical protein	N	N	N	N	N	N
WP_003109689.1	efflux RND transporter periplasmic adaptor subunit	N	N	N	N	N	N
WP_003109690.1	TolC family protein	N	N	N	N	N	N
WP_003109695.1	arsenate reductase ArsC	N	N	N	N	N	N
WP_003109768.1	MobH family relaxase	N	N	N	N	N	N
WP_003109769.1	hypothetical protein	N	N	N	N	N	N
WP_003109772.1	TIGR03757 family integrating conjugative element protein	N	N	N	N	N	N
WP_003109775.1	TIGR03751 family conjugal transfer lipoprotein	N	N	N	N	N	N
WP_003109777.1	PIN domain-containing protein	N	N	N	N	N	N

WP_003109778.1	TIGR03747 family integrating conjugative element membrane protein	N	N	N	N	N	N
WP_003109779.1	integrating conjugative element protein	N	N	N	N	N	N
WP_003109780.1	transglycosylase SLT domain-containing protein	N	N	N	N	N	N
WP_003116799.1	helix-turn-helix domain-containing protein	N	N	N	N	N	N
WP_003116812.1	AAA family ATPase	N	N	N	N	N	N
WP_005304583.1	helix-turn-helix transcriptional regulator	N	N	N	N	N	N
WP_005414984.1	MFS transporter	N	N	N	N	N	N
WP_005416648.1	RebB family R body protein	N	N	N	N	N	N
WP_005416649.1	RebB family R body protein	N	N	N	N	N	N
WP_005416651.1	RebB family R body protein	N	N	N	N	N	N
WP_006226009.1	hypothetical protein	N	N	N	N	N	N
WP_006226010.1	DUF3085 domain-containing protein	N	N	N	N	N	N
WP_006226011.1	DUF3577 domain-containing protein	N	N	N	N	N	N

WP_006226012.1	DUF3275 family protein	N	N	N	N	N	N
WP_006226013.1	hypothetical protein	N	N	N	N	N	N
WP_006226014.1	hypothetical protein	N	N	N	N	N	N
WP_006226241.1	DUF411 domain-containing protein	N	N	N	N	N	N
WP_006375900.1	zincin-like metallopeptidase domain-containing protein	N	N	N	N	N	N
WP_006399830.1	cytochrome o ubiquinol oxidase subunit IV	N	N	N	N	N	N
WP_006446865.1	RebB family R body protein	N	N	N	N	N	N
WP_008264646.1	STY4528 family pathogenicity island replication protein	N	N	N	N	N	N
WP_008266552.1	hypothetical protein	N	N	N	N	N	N
WP_012478642.1	UvrD-helicase domain-containing protein	N	N	N	N	N	N
WP_015014846.1	PadR family transcriptional regulator	N	N	N	N	N	N
WP_020200523.1	DUF6127 family protein	N	N	N	N	N	N
WP_023086160.1	copper resistance protein B	N	N	N	N	N	N

WP_023657320.1	copper resistance system multicopper oxidase	N	N	N	N	N	N
WP_024956711.1	DUF2958 domain-containing protein	N	N	N	N	N	N
WP_024956712.1	DUF2285 domain-containing protein	N	N	NZ_CP060022.1-9, NZ_CP060023.1-9, NZ_CP065965.1-5	100	N	N
WP_024956713.1	helix-turn-helix domain-containing protein	N	N	N	N	N	N
WP_024956717.1	DUF2840 domain-containing protein	N	N	NZ_CP060022.1-9, NZ_CP060023.1-9, NZ_CP065965.1-5	100	N	N
WP_024956718.1	S26 family signal peptidase	N	N	NZ_CP060022.1-9, NZ_CP060023.1-9, NZ_CP065965.1-5	100	N	N
WP_025566605.1	small heat shock protein sHSP20-GI	N	N	N	N	N	N
WP_031688306.1	hypothetical protein	N	N	N	N	N	N
WP_032957905.1	glycosyltransferase family 2 protein	N	N	N	N	N	N
WP_032963389.1	hypothetical protein	N	N	N	N	N	N
WP_034022735.1	DUF6441 family protein	NZ_CP060022.1-5, NZ_CP060023.1-3	100	N	N	N	N
WP_034022740.	hypothetical protein	NZ_CP060022.1-5,	100	N	N	N	N

1		NZ_CP060023.1-3					
WP_034022744.1	hypothetical protein	N	N	N	N	N	N
WP_034022747.1	hypothetical protein	N	N	N	N	N	N
WP_034022749.1	major capsid protein	NZ_CP060022.1-5, NZ_CP060023.1-3	100	NZ_CP060022.1-6	100	N	N
WP_034022752.1	head decoration protein	N	N	N	N	N	N
WP_034022755.1	phage portal protein	NZ_CP060022.1-5, NZ_CP060023.1-3	100	NZ_CP060022.1-6, NZ_CP060023.1-8	100	N	N
WP_034022757.1	hypothetical protein	N	N	N	N	N	N
WP_034022758.1	hypothetical protein	N	N	N	N	N	N
WP_034022759.1	hypothetical protein	N	N	N	N	N	N
WP_034022762.1	DUF3489 domain-containing protein	N	N	NZ_CP060022.1-6, NZ_CP060023.1-8	100	N	N
WP_034022764.1	hypothetical protein	N	N	N	N	N	N
WP_034022766.1	hypothetical protein	N	N	N	N	N	N
WP_034022770.1	site-specific DNA-methyltransferase	N	N	NZ_CP060022.1-6, NZ_CP060023.1-8	100	N	N
WP_034022774.	DUF6362 family protein	N	N	N	N	N	N

1							
WP_034022775.1	hypothetical protein	N	N	N	N	N	N
WP_034022778.1	phage/plasmid primase, P4 family	N	N	NZ_CP060022.1-6, NZ_CP060023.1-8	100	N	N
WP_034022781.1	DUF6511 domain-containing protein	N	N	N	N	N	N
WP_034022783.1	hypothetical protein	N	N	NZ_CP060022.1-6, NZ_CP060023.1-8	100	N	N
WP_034022786.1	ATP-binding protein	N	N	NZ_CP060022.1-6, NZ_CP060023.1-8	100	N	N
WP_034022794.1	hypothetical protein	N	N	N	N	N	N
WP_034022798.1	DUF2924 domain-containing protein	N	N	N	N	N	N
WP_034022805.1	helix-turn-helix transcriptional regulator	N	N	N	N	N	N
WP_034022807.1	hypothetical protein	N	N	N	N	N	N
WP_034022810.1	hypothetical protein	N	N	N	N	N	N
WP_034022813.1	DUF2188 domain-containing protein	N	N	N	N	N	N
WP_034022819.1	phage terminase large subunit family protein	NZ_CP060022.1-5, NZ_CP060023.1-3	100	NZ_CP060022.1-6, NZ_CP060023.1-8	100	N	N
WP_034067943.	Bro-N domain-containing protein	N	N	NZ_CP060022.1-6,	100	N	N

1				NZ_CP060023.1-8			
WP_034067946.1	isoleucyl-tRNA synthetase	N	N	NZ_CP060022.1-6, NZ_CP060023.1-8	100	N	N
WP_046427768.1	NADPH-dependent FMN reductase	N	N	N	N	N	N
WP_046428099.1	type II toxin-antitoxin system RelE/ParE family toxin [Stenotrophomonas sp. GD04064]	N	N	N	N	N	N
WP_046428102.1	HigA family addiction module antitoxin	N	N	N	N	N	N
WP_046428496.1	McrC family protein	N	N	N	N	N	N
WP_046428716.1	ABC transporter ATP-binding protein	N	N	N	N	N	N
WP_046430205.1	HsdR family type I site-specific deoxyribonuclease	N	N	N	N	N	N
WP_046430206.1	SprT family zinc-dependent metalloprotease	N	N	N	N	N	N
WP_046430563.1	metalloregulator ArsR/SmtB family transcription factor	N	N	N	N	N	N
WP_046430578.1	sodium:proton exchanger	N	N	N	N	N	N
WP_046430586.1	heavy metal translocating P-type ATPase	N	N	N	N	N	N
WP_046430608.1	Tn3 family transposase	N	N	N	N	N	N

WP_046432106.1	XRE family transcriptional regulator	N	N	N	N	N	N
WP_046432108.1	type IV toxin-antitoxin system AbiEi family antitoxin domain-containing protein	N	N	N	N	N	N
WP_046432110.1	nucleotidyl transferase AbiEii/AbiGii toxin family protein	N	N	N	N	N	N
WP_046432112.1	recombinase family protein	N	N	N	N	N	N
WP_046432114.1	ParB/RepB/Spo0J family partition protein	N	N	N	N	N	N
WP_046432116.1	plasmid partitioning protein RepB C-terminal domain-containing protein	N	N	N	N	N	N
WP_046432121.1	lysozyme	N	N	N	N	N	N
WP_046432123.1	hypothetical protein	N	N	N	N	N	N
WP_046432125.1	DUF2793 domain-containing protein	N	N	N	N	N	N
WP_046432152.1	recombinase family protein	N	N	NZ_CP060022.1-6, NZ_CP060023.1-8	100	N	N
WP_046432185.1	hypothetical protein	N	N	N	N	N	N
WP_046432224.1	zeta toxin family protein	N	N	N	N	N	N
WP_049244394.	hypothetical protein	N	N	N	N	N	N

1							
WP_049408527.1	heat resistance protein YfdX2	N	N	N	N	N	N
WP_049408528.1	heat resistance protein YfdX1	N	N	N	N	N	N
WP_049420578.1	helix-turn-helix domain-containing protein	N	N	N	N	N	N
WP_049420580.1	Hsp20/alpha crystallin family protein	N	N	N	N	N	N
WP_049420584.1	cardiolipin synthase	N	N	N	N	N	N
WP_049420590.1	HdeD family acid-resistance protein	N	N	N	N	N	N
WP_049420592.1	hypothetical protein	N	N	N	N	N	N
WP_072167338.1	arsenate reductase ArsC	N	N	N	N	N	N
WP_087786704.1	ISL3 family transposase	N	N	N	N	N	N
WP_087944428.1	IS3-like element ISStma9 family transposase	NC_010943.1-1	100	NZ_CP060023.1-10, NZ_CP060024.1-6, NZ_CP060022.1-10	100	N	N
WP_116825096.1	OST-HTH/LOTUS domain-containing protein	N	N	NZ_CP060022.1-6, NZ_CP060023.1-8	100	N	N
WP_141098655.1	ABC transporter permease	N	N	N	N	N	N

WP_143568593.1	hypothetical protein	N	N	N	N	N	N
WP_180839888.1	site-specific DNA-methyltransferase	N	N	N	N	N	N
WP_180839947.1	TIGR03885 family FMN-dependent LLM class oxidoreductase	N	N	N	N	N	N
WP_180839963.1	TIGR03752 family integrating conjugative element protein	N	N	N	N	N	N
WP_180839965.1	heavy metal translocating P-type ATPase	N	N	N	N	N	N
WP_180840055.1	very short patch repair endonuclease [Stenotrophomonas maltophilia group sp. msm4]	N	N	N	N	N	N
WP_180840060.1	DNA cytosine methyltransferase [Stenotrophomonas maltophilia group sp. msm4]	N	N	N	N	N	N
WP_180840062.1	GIY-YIG nuclease family protein [Stenotrophomonas maltophilia group sp. msm4]	N	N	N	N	N	N
WP_180840131.1	heat resistance system K+/H+ antiporter KefB-GI	N	N	N	N	N	N
WP_197596969.1	vitamin K epoxide reductase family protein	N	N	N	N	N	N
WP_197651614.1	ImmA/IrrE family metallo-endopeptidase	N	N	N	N	N	N
WP_234600102.	elements of external origin	N	N	N	N	N	N

	1						
	WP_254916320.1	DUF6094 domain-containing protein	N	N	N	N	N	N
	WP_258008290.1	ATP-binding protein	N	N	N	N	N	N
	WP_000761850.1	organomercurial lyase MerB	N	N	N	N	N	N
	WP_001138070.1	Tn3 family transposase	NZ_CP060024.1-1	100	N	N	N	N
	WP_003124096.1	recombinase family protein	N	N	N	N	N	N
	WP_003132004.1	broad-spectrum mercury transporter MerE	N	N	N	N	N	N
	WP_004153231.1	DEAD/DEAH box helicase family protein	N	N	N	N	N	N
NZ_CP060024.1	WP_005408332.1	GPW/gp25 family protein	N	N	N	N	N	N
	WP_005413325.1	alpha/beta hydrolase	N	N	N	N	N	N
	WP_005413329.1	hypothetical protein	N	N	N	N	N	N
	WP_005414259.1	hypothetical protein	N	N	N	N	N	N
	WP_005995733.1	DUF305 domain-containing protein	N	N	N	N	N	N
	WP_006375900.	zincin-like metallopeptidase	N	N	N	N	N	N

1	domain-containing protein						
WP_012478973.1	McrC family protein	N	N	N	N	N	N
WP_012479075.1	TIM barrel protein	N	N	N	N	N	N
WP_012479078.1	glycosyltransferase	N	N	N	N	N	N
WP_012479702.1	hypothetical protein, partial	N	N	N	N	N	N
WP_012480196.1	NAD(P)/FAD-dependent oxidoreductase	N	N	N	N	N	N
WP_012480197.1	metalloregulator ArsR/SmtB family transcription factor	N	N	N	N	N	N
WP_012480664.1	DUF3320 domain-containing protein	N	N	N	N	N	N
WP_012481497.1	cytochrome o ubiquinol oxidase subunit IV	N	N	N	N	N	N
WP_021202108.1	metalloregulator ArsR/SmtB family transcription factor	N	N	N	N	N	N
WP_021204017.1	AAA family ATPase	N	N	N	N	N	N
WP_024957346.1	glycosyltransferase family 2 protein	N	N	N	N	N	N
WP_024957931.1	DUF1629 domain-containing protein	N	N	N	N	N	N
WP_032966722.	hypothetical protein	N	N	N	N	N	N

	1							
	WP_033690515.1	DUF3330 domain-containing protein	N	N	N	N	N	N
	WP_065178072.1	zincin-like metallopeptidase domain-containing protein	N	N	N	N	N	N
	WP_072167333.1	YeeE/YedE thiosulfate transporter family protein	N	N	N	N	N	N
	WP_185812492.1	mercury resistance co-regulator MerD	N	N	N	N	N	N
	WP_185812597.1	ABC transporter ATP-binding protein	N	N	N	N	N	N
	WP_185812599.1	SMR family transporter	N	N	N	N	N	N
	WP_221892764.1	site-specific DNA-methyltransferase	N	N	N	N	N	N
	WP_229297698.1	SMI1/KNR4 family protein	N	N	N	N	N	N
	WP_003140997.1	TIGR03758 family integrating conjugative element protein	N	N	N	N	N	N
	WP_003141001.1	helix-turn-helix domain-containing protein	N	N	N	N	N	N
NZ_CP060025.1	WP_003141015.1	integrating conjugative element protein	N	N	N	N	N	N
	WP_003141051.1	hypothetical protein	N	N	N	N	N	N
	WP_003292115.	hypothetical protein	N	N	N	N	N	N

1							
WP_004362284.1	single-stranded DNA-binding protein	N	N	N	N	N	N
WP_005409655.1	IS110 family transposase	N	N	NZ_CP088244.1-3	100	NZ_CP060025.1-2, NZ_CP060027.1-4, NZ_CP098483.1-4	100
WP_005409659.1	heat resistance system K+/H+ antiporter KefB-GI	NZ_CP060026.1-2	100	NZ_CP040440.1-7, NZ_CP088244.1-3	100	NZ_CP060025.1-2, NZ_CP060027.1-4, NZ_CP098483.1-4	100
WP_005409662.1	HdeD family acid-resistance protein	NZ_CP060026.1-2	100	N	N	NZ_CP060025.1-2, NZ_CP060027.1-4, NZ_CP098483.1-4	100
WP_005409663.1	heat resistance protein YfdX2	NZ_CP060026.1-2	100	N	N	NZ_CP060025.1-2, NZ_CP060027.1-4, NZ_CP098483.1-4, NZ_CP102942.1-4	100
WP_005409664.1	heat resistance protein YfdX1	NZ_CP060026.1-2	100	N	N	NZ_CP060025.1-2, NZ_CP060027.1-4, NZ_CP102942.1-4, NZ_CP098483.1-4	100
WP_005409665.1	small heat shock protein sHSP20-GI	N	N	N	N	N	N
WP_005409667.1	cardiolipin synthase	N	N	N	N	N	N
WP_005409669.1	Hsp20/alpha crystallin family protein	NZ_CP060026.1-2	100	NZ_CP040431.1-4, NZ_CP040433.1-2,	100	NZ_CP060025.1-2, NZ_CP060027.1-4,	100

				NZ_CP040440.1-7, NZ_CP088244.1-4		NZ_CP102942.1-4, NZ_CP098483.1-4	
WP_005414983.1	ATP-binding protein	N	N	N	N	N	N
WP_005414984.1	MFS transporter	N	N	N	N	N	N
WP_009618209.1	type II toxin-antitoxin system RelE/ParE family toxin	N	N	N	N	N	N
WP_009618210.1	type II toxin-antitoxin system RelB/DinJ family antitoxin	N	N	N	N	N	N
WP_011829925.1	AlpA family transcriptional regulator	N	N	N	N	N	N
WP_012478642.1	UvrD-helicase domain-containing protein	N	N	N	N	N	N
WP_013982110.1	ParA family protein	N	N	N	N	N	N
WP_013982111.1	type II toxin-antitoxin system HicA family toxin	N	N	N	N	N	N
WP_013982112.1	ParB family protein	N	N	N	N	N	N
WP_013982130.1	TIGR03759 family integrating conjugative element protein	N	N	N	N	N	N
WP_013982148.1	DUF3742 family protein	N	N	N	N	N	N
WP_017244834.1	TIGR03750 family conjugal transfer protein	N	N	N	N	N	N

WP_019484549.1	TIGR03745 family integrating conjugative element membrane protein	N	N	N	N	N	N
WP_019484551.1	TIGR03746 family integrating conjugative element protein	N	N	N	N	N	N
WP_019751268.1	STY4534 family ICE replication protein	N	N	N	N	N	N
WP_023083166.1	TIGR03751 family conjugal transfer lipoprotein	N	N	N	N	N	N
WP_023083168.1	TIGR03749 family integrating conjugative element protein	N	N	N	N	N	N
WP_023121122.1	type IV toxin-antitoxin system AbiEi family antitoxin domain-containing protein	N	N	N	N	N	N
WP_023657702.1	tyrosine-type recombinase/integrase	N	N	N	N	N	N
WP_023912240.1	DUF2857 domain-containing protein	N	N	N	N	N	N
WP_023912242.1	STY4528 family pathogenicity island replication protein	N	N	N	N	N	N
WP_023912244.1	TIGR03761 family integrating conjugative element protein	N	N	N	N	N	N
WP_023912246.1	DUF3158 family protein	N	N	N	N	N	N
WP_025297717.1	hypothetical protein	N	N	N	N	N	N
WP_031755001.	DUF3275 family protein	N	N	N	N	N	N

1							
WP_033873014.1	integrating conjugative element protein	N	N	N	N	N	N
WP_034021462.1	hypothetical protein	N	N	N	N	N	N
WP_043104882.1	PilL N-terminal domain-containing protein	N	N	N	N	N	N
WP_045786220.1	DNA repair protein RadC	N	N	N	N	N	N
WP_049429336.1	BsuBI/PstI family type II restriction endonuclease	N	N	N	N	N	N
WP_049439817.1	type ISP restriction/modification enzyme	N	N	N	N	N	N
WP_061479192.1	nuclear transport factor 2 family protein	N	N	N	N	N	N
WP_061959005.1	thioredoxin domain-containing protein	N	N	N	N	N	N
WP_061959006.1	TIGR03757 family integrating conjugative element protein	N	N	N	N	N	N
WP_061960173.1	anti-sigma factor	N	N	N	N	N	N
WP_061960174.1	beta-propeller fold lactonase family protein	N	N	N	N	N	N
WP_062842676.1	hypothetical protein	N	N	N	N	N	N
WP_065178056.	type II toxin-antitoxin system HigB	N	N	N	N	N	N

1	family toxin						
WP_080052373.1	TatD family hydrolase	N	N	N	N	N	N
WP_085371671.1	DUF2958 domain-containing protein	N	N	N	N	N	N
WP_114618409.1	heat resistance protein PsiE-GI	N	N	N	N	N	N
WP_125866636.1	ABC transporter substrate-binding protein	N	N	N	N	N	N
WP_128989844.1	DNA methyltransferase	N	N	N	N	N	N
WP_134969248.1	helix-turn-helix domain-containing GNAT family N-acetyltransferase	N	N	N	N	N	N
WP_134970267.1	sigma-54 dependent transcriptional regulator	N	N	N	N	N	N
WP_154350134.1	DNA (cytosine-5-)-methyltransferase	N	N	N	N	N	N
WP_154350191.1	ABC transporter ATP-binding protein	N	N	N	N	N	N
WP_154350319.1	ATP-binding protein	N	N	N	N	N	N
WP_154350320.1	AAA family ATPase	N	N	N	N	N	N
WP_154350512.1	type II toxin-antitoxin system HipA family toxin	N	N	N	N	N	N
WP_154350595.	AlpA family phage regulatory protein	N	N	N	N	N	N

1							
WP_154350600.1	hypothetical protein	N	N	N	N	N	N
WP_154350601.1	DUF736 domain-containing protein	N	N	N	N	N	N
WP_154350603.1	TIR domain-containing protein	N	N	N	N	N	N
WP_154350604.1	helix-turn-helix transcriptional regulator	N	N	N	N	N	N
WP_154350605.1	DUF2285 domain-containing protein	N	N	N	N	N	N
WP_154350608.1	chromosome partitioning protein ParB	N	N	N	N	N	N
WP_154350609.1	DUF2840 domain-containing protein	N	N	N	N	N	N
WP_154350610.1	S26 family signal peptidase	N	N	N	N	N	N
WP_154350612.1	ATP-binding protein	N	N	N	N	N	N
WP_154350613.1	response regulator transcription factor	N	N	N	N	N	N
WP_154350618.1	LysR substrate-binding domain-containing protein	N	N	N	N	N	N
WP_154350619.1	entry exclusion lipoprotein TrbK	N	N	N	N	N	N
WP_154350623.	TrbC/VirB2 family protein	N	N	N	N	N	N

1							
WP_154350625.1	P-type conjugative transfer protein TrbJ	N	N	N	N	N	N
WP_154350626.1	hypothetical protein	N	N	N	N	N	N
WP_154350631.1	DUF3320 domain-containing protein	N	N	N	N	N	N
WP_154350780.1	DnaJ C-terminal domain-containing protein	N	N	N	N	N	N
WP_154350799.1	fimbria/pilus chaperone family protein	N	N	N	N	N	N
WP_154350826.1	multidrug efflux RND transporter permease subunit	N	N	N	N	N	N
WP_154350827.1	MFS transporter	N	N	N	N	N	N
WP_154350897.1	TIGR03885 family FMN-dependent LLM class oxidoreductase	N	N	N	N	N	N
WP_154351305.1	GTPase	N	N	N	N	N	N
WP_154351306.1	DUF3085 domain-containing protein	N	N	N	N	N	N
WP_154351307.1	hypothetical protein	N	N	N	N	N	N
WP_154351308.1	DUF6094 domain-containing protein	N	N	N	N	N	N
WP_154351309.	hypothetical protein	N	N	N	N	N	N

1							
WP_154351310.1	lytic transglycosylase domain-containing protein	N	N	N	N	N	N
WP_154351312.1	TIGR03747 family integrating conjugative element membrane protein	N	N	N	N	N	N
WP_154351313.1	molybdopterin-dependent oxidoreductase	N	N	N	N	N	N
WP_154351314.1	nuclear transport factor 2 family protein	N	N	N	N	N	N
WP_154351315.1	RAQPRD family integrative conjugative element protein	N	N	N	N	N	N
WP_154351316.1	TIGR03752 family integrating conjugative element protein	N	N	N	N	N	N
WP_154351319.1	conjugal transfer protein TraG N-terminal domain-containing protein	N	N	N	N	N	N
WP_154351320.1	nucleotidyl transferase AbiEii/AbiGii toxin family protein	N	N	N	N	N	N
WP_154351321.1	MobH family relaxase	N	N	N	N	N	N
WP_154351322.1	substrate-binding domain-containing protein	N	N	N	N	N	N
WP_154351554.1	hypothetical protein	N	N	N	N	N	N
WP_154351717.1	LysR substrate-binding domain-containing protein	N	N	N	N	N	N
WP_185807360.	very short patch repair endonuclease	N	N	N	N	N	N

1							
WP_185807475.1	LysR family transcriptional regulator	N	N	N	N	N	N
WP_185807476.1	LysR family transcriptional regulator	N	N	N	N	N	N
WP_185807478.1	RNA polymerase sigma factor	N	N	N	N	N	N
WP_197697522.1	YbhB/YbcL family Raf kinase inhibitor-like protein	N	N	N	N	N	N
WP_221892504.1	ISL3 family transposase	N	N	N	N	N	N
WP_221892513.1	efflux RND transporter periplasmic adaptor subunit	N	N	N	N	N	N
WP_227200205.1	helix-turn-helix transcriptional regulator	N	N	N	N	N	N
WP_229298442.1	efflux RND transporter periplasmic adaptor subunit	N	N	N	N	N	N
WP_229298481.1	SNF2-related protein	N	N	N	N	N	N
WP_229298486.1	chromate efflux transporter	N	N	N	N	N	N
WP_229298541.1	efflux RND transporter permease subunit	N	N	N	N	N	N
WP_229298548.1	bestrophin family protein	N	N	N	N	N	N
WP_229298574.	DsbA family protein	N	N	N	N	N	N

	1							
	WP_260619835.1	HipA N-terminal domain-containing protein, partial	N	N	N	N	N	N
	WP_005408332.1	GPW/gp25 family protein	N	N	N	N	N	N
	WP_005409659.1	heat resistance system K+/H+ antiporter KefB-GI	NZ_CP060026.1-2	100	NZ_CP040440.1-7, NZ_CP088244.1-3	100	NZ_CP060025.1-2, NZ_CP060027.1-4, NZ_CP098483.1-4	100
	WP_005409661.1	hypothetical protein	NZ_CP060026.1-2	100	N	N	NZ_CP060025.1-2, NZ_CP060027.1-4, NZ_CP098483.1-4	100
	WP_005409662.1	HdeD family acid-resistance protein	NZ_CP060026.1-2	100	N	N	NZ_CP060025.1-2, NZ_CP060027.1-4, NZ_CP098483.1-4	100
NZ_CP060026.1	WP_005409663.1	heat resistance protein YfdX2	NZ_CP060026.1-2	100	N	N	NZ_CP060025.1-2, NZ_CP060027.1-4, NZ_CP098483.1-4, NZ_CP102942.1-4	100
	WP_005409664.1	heat resistance protein YfdX1	NZ_CP060026.1-2	100	N	N	NZ_CP060025.1-2, NZ_CP060027.1-4, NZ_CP102942.1-4, NZ_CP098483.1-4	100
	WP_005409665.1	small heat shock protein sHSP20-GI	N	N	N	N	N	N
	WP_005409667.1	cardiolipin synthase	N	N	N	N	N	N

WP_005409669.1	Hsp20/alpha crystallin family protein	NZ_CP060026.1-2	100	NZ_CP040431.1-4,<br>NZ_CP040433.1-2,<br>NZ_CP040440.1-7,<br>NZ_CP088244.1-4	100	NZ_CP060025.1-2,<br>NZ_CP060027.1-4,<br>NZ_CP102942.1-4,<br>NZ_CP098483.1-4	100
WP_011517532.1	cytochrome b	N	N	N	N	N	N
WP_012480368.1	TIGR03885 family FMN-dependent LLM class oxidoreductase	N	N	N	N	N	N
WP_012481497.1	cytochrome o ubiquinol oxidase subunit IV	N	N	N	N	N	N
WP_014859386.1	DUF2840 domain-containing protein	N	N	N	N	N	N
WP_014859387.1	hypothetical protein	N	N	N	N	N	N
WP_014859391.1	helix-turn-helix domain-containing protein	N	N	N	N	N	N
WP_014859392.1	DUF2285 domain-containing protein	N	N	NZ_CP040440.1-8,<br>NZ_CP060026.1-4	100		N
WP_014859394.1	DUF2958 domain-containing protein	N	N	N	N	N	N
WP_014859395.1	hypothetical protein	N	N	N	N	N	N
WP_014861255.1	DNA repair protein RadC	N	N	N	N	N	N
WP_014861256.1	PDDEXK nuclease domain-containing protein	N	N	NZ_CP040440.1-8,<br>NZ_CP060026.1-4	100	N	N

WP_014861257.1	site-specific integrase	N	N	NZ_CP040440.1-8, NZ_CP060026.1-4	100	N	N
WP_024001347.1	ferric reductase-like transmembrane domain-containing protein	N	N	N	N	N	N
WP_024537630.1	S26 family signal peptidase	N	N	NZ_CP040440.1-8, NZ_CP060026.1-4	100	N	N
WP_024956907.1	LysR substrate-binding domain-containing protein	N	N	N	N	N	N
WP_032961714.1	heat resistance protein PsiE-GI	N	N	N	N	N	N
WP_034400679.1	diacylglycerol kinase	N	N	N	N	N	N
WP_043371530.1	TrbC/VirB2 family protein	N	N	N	N	N	N
WP_049395161.1	histidine utilization repressor	N	N	N	N	N	N
WP_049395165.1	DUF305 domain-containing protein	N	N	N	N	N	N
WP_049395288.1	helix-turn-helix transcriptional regulator	N	N	N	N	N	N
WP_049395575.1	P-type conjugative transfer protein TrbL	N	N	N	N	N	N
WP_049395576.1	hypothetical protein	N	N	N	N	N	N
WP_049395578.1	P-type conjugative transfer protein TrbJ	N	N	N	N	N	N

WP_049395585.1	LysR family transcriptional regulator	N	N	N	N	N	N
WP_049396030.1	TM0106 family RecB-like putative nuclease	NZ_CP060026.1-2	100	NZ_CP040440.1-7	100	N	N
WP_049396984.1	helix-turn-helix domain-containing protein	N	N	N	N	N	N
WP_049396991.1	group II intron reverse transcriptase/maturase	N	N	N	N	N	N
WP_049397307.1	IS110 family transposase	NZ_CP060026.1-2	100	NZ_CP040440.1-7	100	NZ_CP102942.1-4	100
WP_049397460.1	LysR family transcriptional regulator	N	N	N	N	N	N
WP_049397550.1	ABC transporter ATP-binding protein	N	N	N	N	N	N
WP_049397866.1	phage terminase large subunit	NZ_CP040440.1-2	100	NZ_CP060026.1-5	100	N	N
WP_049397868.1	lysozyme	N	N	N	N	N	N
WP_049406765.1	tyrosine-type recombinase/integrase	N	N	N	N	N	N
WP_049406835.1	phosphoadenosine phosphosulfate reductase family protein	N	N	N	N	N	N
WP_049407014.1	DUF262 domain-containing protein	N	N	N	N	N	N
WP_049407016.1	hypothetical protein	N	N	N	N	N	N

WP_049407018.1	BREX-3 system phosphatase PglZ	N	N	N	N	N	N
WP_049407022.1	DNA methyltransferase	N	N	N	N	N	N
WP_049407025.1	BREX-3 system P-loop-containing protein BrxF	N	N	N	N	N	N
WP_049407026.1	WYL domain-containing protein	N	N	N	N	N	N
WP_049407028.1	DUF6079 family protein	N	N	N	N	N	N
WP_062166963.1	nucleotide-binding protein	N	N	NZ_CP040440.1-8, NZ_CP060026.1-4	100	N	N
WP_079390835.1	helix-turn-helix transcriptional regulator	N	N	N	N	N	N
WP_080052373.1	TatD family hydrolase	N	N	N	N	N	N
WP_080240930.1	ABC transporter permease	N	N	N	N	N	N
WP_080354090.1	helix-turn-helix domain-containing protein	N	N	N	N	N	N
WP_080354103.1	amino acid ABC transporter permease/ATP-binding protein	N	N	N	N	N	N
WP_088611174.1	tyrosine-type recombinase/integrase	NZ_CP040440.1-5, NZ_CP060026.1-3	100	N	N	NZ_CP022053.2-8	100
WP_099540247.1	DNA cytosine methyltransferase	NZ_CP040440.1-1, NZ_CP060026.1-1	100	N	N	N	N

	WP_154344303.1	GPW/gp25 family protein	N	N	N	N	N	N
	WP_154344436.1	terminase	N	N	N	N	N	N
	WP_154344446.1	DNA N-6-adenine-methyltransferase	NZ_CP040440.1-5, NZ_CP060026.1-3	100	N	N	N	N
	WP_162838283.1	DEAD/DEAH box helicase family protein	N	N	N	N	N	N
	WP_197594234.1	helicase-related protein	N	N	N	N	N	N
	WP_221892533.1	amidohydrolase, partial	N	N	N	N	N	N
	WP_221892535.1	ADP-ribosylglycohydrolase family protein, partial	N	N	N	N	N	N
	WP_224481980.1	GNAT family N-acetyltransferase	N	N	N	N	N	N
	WP_229302592.1	type II toxin-antitoxin system RelE/ParE family toxin	N	N	N	N	N	N
	WP_231347490.1	nuclear transport factor 2 family protein	N	N	N	N	N	N
	WP_306766115.1	Mov34/MPN/PAD-1 family protein	N	N	N	N	N	N
NZ_CP060027.1	WP_005408332.1	GPW/gp25 family protein	N	N	N	N	N	N
	WP_005408904.1	nuclear transport factor 2 family protein	N	N	N	N	N	N

WP_005409644.1	hypothetical protein	N	N	N	N	N	N
WP_005409655.1	IS110 family transposase	N	N	NZ_CP088244.1-3	100	NZ_CP060025.1-2, NZ_CP060027.1-4, NZ_CP098483.1-4	100
WP_005409659.1	heat resistance system K+/H+ antiporter KefB-GI	NZ_CP060026.1-2	100	NZ_CP040440.1-7, NZ_CP088244.1-3	100	NZ_CP060025.1-2, NZ_CP060027.1-4, NZ_CP098483.1-4	100
WP_005409661.1	hypothetical protein	NZ_CP060026.1-2	100	N	N	NZ_CP060025.1-2, NZ_CP060027.1-4, NZ_CP098483.1-4	100
WP_005409662.1	HdeD family acid-resistance protein	NZ_CP060026.1-2	100	N	N	NZ_CP060025.1-2, NZ_CP060027.1-4, NZ_CP098483.1-4	100
WP_005409663.1	heat resistance protein YfdX2	NZ_CP060026.1-2	100	N	N	NZ_CP060025.1-2, NZ_CP060027.1-4, NZ_CP098483.1-4, NZ_CP102942.1-4	100
WP_005409664.1	heat resistance protein YfdX1	NZ_CP060026.1-2	100	N	N	NZ_CP060025.1-2, NZ_CP060027.1-4, NZ_CP102942.1-4, NZ_CP098483.1-4	100
WP_005409665.1	small heat shock protein sHSP20-GI	N	N	N	N	N	N
WP_005409667.1	cardiolipin synthase	N	N	N	N	N	N

WP_005409669.1	Hsp20/alpha crystallin family protein	NZ_CP060026.1-2	100	NZ_CP040431.1-4, NZ_CP040433.1-2, NZ_CP040440.1-7, NZ_CP088244.1-4	100	NZ_CP060025.1-2, NZ_CP060027.1-4, NZ_CP102942.1-4, NZ_CP098483.1-4	100
WP_012479074.1	SMR family transporter	N	N	N	N	N	N
WP_012479075.1	TIM barrel protein	N	N	N	N	N	N
WP_012479077.1	FAD-dependent oxidoreductase	N	N	N	N	N	N
WP_012479078.1	glycosyltransferase	N	N	N	N	N	N
WP_024957346.1	glycosyltransferase family 2 protein	N	N	N	N	N	N
WP_032960048.1	site-specific integrase	N	N	N	N	N	N
WP_032961707.1	helix-turn-helix domain-containing protein	N	N	N	N	N	N
WP_032961714.1	heat resistance protein PsiE-GI	N	N	N	N	N	N
WP_032961836.1	phage terminase large subunit	NZ_CM001824.1-1, NZ_CP014014.1-2, NZ_LR134324.1-1	100	NZ_AP021908.1-1, NZ_CP040431.1-2, NZ_CP040439.1-4, NZ_CP043578.1-1, NZ_CP056088.1-2, NZ_CP060027.1-3,	100	NZ_CP040430.1-2	100

				NZ_LR134324.1-3,<br>NZ_CP090418.1-2,<br>NZ_CP104863.1-3,<br>NZ_CP102942.1-3,<br>NZ_CP098483.1-2			
WP_032965863.1	hypothetical protein	N	N	N	N	N	N
WP_049449467.1	site-specific integrase	NZ_CP088244.1-1,<br>NZ_CP098483.1-1	100	N	N	NZ_CP040433.1-3,<br>NZ_CP060025.1-3	100
WP_061478821.1	DNA cytosine methyltransferase	NZ_CP040431.1-1,<br>NZ_CP060027.1-1	100	N	N	N	N
WP_065426719.1	hypothetical protein	N	N	N	N	N	N
WP_076738974.1	DUF1993 domain-containing protein	N	N	N	N	N	N
WP_154329194.1	AIPR family protein	N	N	N	N	N	N
WP_154329196.1	Z1 domain-containing protein	N	N	N	N	N	N
WP_154329200.1	very short patch repair endonuclease	N	N	N	N	N	N
WP_154329370.1	ImmA/IrrE family metallo-endopeptidase	N	N	N	N	N	N
WP_154329545.1	N-6 DNA methylase	N	N	N	N	N	N
WP_154329687.	TatD family hydrolase	N	N	N	N	N	N

	1							
	WP_185805886. 1	metallohydrolase	N	N	N	N	N	N
	WP_197599535. 1	NAD(P)-dependent oxidoreductase	N	N	N	N	N	N
	WP_227860296. 1	ATP-binding protein	N	N	N	N	N	N
	WP_229298666. 1	SDR family oxidoreductase	N	N	N	N	N	N
	WP_306766115. 1	Mov34/MPN/PAD-1 family protein	N	N	N	N	N	N
	WP_005408320. 1	tautomerase family protein	N	N	N	N	N	N
	WP_005409501. 1	transcriptional regulator BetI	N	N	N	N	N	N
	WP_005409726. 1	ABC transporter permease	N	N	N	N	N	N
NZ_CP060259.1	WP_005409874. 1	biopolymer transporter ExbD	N	N	N	N	N	N
	WP_005409984. 1	MgtC/SapB family protein	N	N	N	N	N	N
	WP_005413654. 1	Lrp/AsnC family transcriptional regulator	N	N	N	N	N	N
	WP_006402206. 1	acyl carrier protein	N	N	N	N	N	N
	WP_010480868.	LysR family transcriptional regulator	N	N	N	N	N	N

1							
WP_010481131.1	response regulator transcription factor	N	N	N	N	N	N
WP_010481875.1	LysR family transcriptional regulator	N	N	N	N	N	N
WP_010482305.1	hypothetical protein	N	N	N	N	N	N
WP_010483459.1	helix-turn-helix transcriptional regulator	N	N	N	N	N	N
WP_010483717.1	response regulator transcription factor	N	N	N	N	N	N
WP_010483779.1	baseplate J/gp47 family protein	N	N	N	N	N	N
WP_010483787.1	phage tail sheath subtilisin-like domain-containing protein	NZ_CP011305.1-1, NZ_CP011306.1-1, NZ_CP060259.1-2, NZ_CP077679.1-1, NZ_CP104169.1-1, NZ_OU943334.1-1	100	N	N	N	N
WP_010484073.1	type II toxin-antitoxin system RelE/ParE family toxin	N	N	N	N	N	N
WP_010484074.1	HigA family addiction module antitoxin	N	N	N	N	N	N
WP_010484092.1	winged helix-turn-helix domain-containing protein	N	N	N	N	N	N
WP_010484425.	protein deglycase HchA	N	N	N	N	N	N

1							
WP_010484895.1	helix-turn-helix domain-containing protein	N	N	N	N	N	N
WP_010485313.1	respiratory nitrate reductase subunit gamma	N	N	N	N	N	N
WP_010485315.1	nitrate/nitrite transporter	N	N	N	N	N	N
WP_010485465.1	DUF3224 domain-containing protein	N	N	N	N	N	N
WP_010485668.1	LysR family transcriptional regulator	N	N	N	N	N	N
WP_010485778.1	response regulator	N	N	N	N	N	N
WP_010486459.1	VOC family protein	N	N	N	N	N	N
WP_010486485.1	metalloregulator ArsR/SmtB family transcription factor	N	N	N	N	N	N
WP_010486984.1	type II toxin-antitoxin system RelE/ParE family toxin	N	N	N	N	N	N
WP_012479558.1	EamA family transporter	N	N	N	N	N	N
WP_019336271.1	hypothetical protein	N	N	N	N	N	N
WP_019336320.1	ubiquinol oxidase subunit II	N	N	N	N	N	N
WP_019336516.	carboxymuconolactone decarboxylase	N	N	N	N	N	N

1	family protein						
WP_019337581.1	DMT family transporter	N	N	N	N	N	N
WP_019337583.1	response regulator	N	N	N	N	N	N
WP_019337773.1	type II secretion system major pseudopilin GspG	N	N	N	N	N	N
WP_019337810.1	NarK family nitrate/nitrite MFS transporter	N	N	N	N	N	N
WP_019338001.1	class I SAM-dependent methyltransferase	N	N	N	N	N	N
WP_019338302.1	GNAT family N-acetyltransferase	N	N	N	N	N	N
WP_019338357.1	alpha/beta hydrolase	N	N	N	N	N	N
WP_019338771.1	formate dehydrogenase subunit gamma	N	N	N	N	N	N
WP_019338970.1	DNA-binding transcriptional regulator	N	N	N	N	N	N
WP_019660267.1	DUF2239 family protein	N	N	N	N	N	N
WP_019661429.1	Cu(I)-responsive transcriptional regulator	N	N	N	N	N	N
WP_020424480.1	response regulator	N	N	N	N	N	N
WP_029380003.	biotin/lipoyl-binding protein	N	N	N	N	N	N

1							
WP_029380112.1	putative addiction module antidote protein	N	N	N	N	N	N
WP_032959372.1	Rid family hydrolase	N	N	N	N	N	N
WP_043396034.1	NAD(P)-dependent oxidoreductase	N	N	N	N	N	N
WP_043396259.1	AzlD family protein	N	N	N	N	N	N
WP_043396696.1	GyrI-like domain-containing protein	N	N	N	N	N	N
WP_043397376.1	hypothetical protein	N	N	N	N	N	N
WP_043397553.1	VOC family protein	N	N	N	N	N	N
WP_043400854.1	SDR family oxidoreductase	N	N	N	N	N	N
WP_043402041.1	Rid family hydrolase	N	N	N	N	N	N
WP_049410042.1	VOC family protein	N	N	N	N	N	N
WP_049410845.1	SDR family oxidoreductase	N	N	N	N	N	N
WP_049411723.1	hypothetical protein	N	N	N	N	N	N
WP_049412996.	helix-turn-helix domain-containing	N	N	N	N	N	N

1	protein						
WP_049413006.1	response regulator transcription factor	N	N	N	N	N	N
WP_049413021.1	NADH:flavin oxidoreductase/NADH oxidase family protein	N	N	N	N	N	N
WP_049426760.1	helix-turn-helix transcriptional regulator	N	N	N	N	N	N
WP_049434138.1	SMR family transporter	N	N	N	N	N	N
WP_049434910.1	SRPBCC family protein	N	N	N	N	N	N
WP_049437620.1	response regulator transcription factor	N	N	N	N	N	N
WP_049437693.1	MerR family transcriptional regulator	N	N	N	N	N	N
WP_049453529.1	BCCT family transporter	N	N	N	N	N	N
WP_049455337.1	TonB-dependent receptor	N	N	N	N	N	N
WP_049455405.1	MerR family transcriptional regulator	N	N	N	N	N	N
WP_049455462.1	zinc-dependent alcohol dehydrogenase family protein	N	N	N	N	N	N
WP_049481079.1	ATP-binding protein	N	N	N	N	N	N
WP_049482241.	methylated-DNA--[protein]-cysteine	N	N	N	N	N	N

1	S-methyltransferase						
WP_049482249.1	sugar phosphate isomerase/epimerase family protein	N	N	N	N	N	N
WP_049482252.1	TetR/AcrR family transcriptional regulator	N	N	N	N	N	N
WP_049483138.1	LysE family transporter [Stenotrophomonas sp. NY11291]	N	N	N	N	N	N
WP_053442503.1	UDP-N-acetylglucosamine 2-epimerase (non-hydrolyzing)	N	N	N	N	N	N
WP_053443171.1	diacylglycerol kinase	N	N	N	N	N	N
WP_053443191.1	VOC family protein	N	N	N	N	N	N
WP_057503330.1	LysR family transcriptional regulator	N	N	N	N	N	N
WP_071305687.1	cellulase family glycosylhydrolase	N	N	N	N	N	N
WP_099559268.1	alpha/beta hydrolase	N	N	N	N	N	N
WP_100050633.1	ATP-binding protein	N	N	N	N	N	N
WP_109814228.1	heavy metal response regulator transcription factor	N	N	N	N	N	N
WP_109814876.1	VOC family protein	N	N	N	N	N	N
WP_126561042.	selenocysteine-specific translation	N	N	N	N	N	N

1	elongation factor						
WP_126561141.1	hypothetical protein	N	N	N	N	N	N
WP_143994368.1	ATP-binding cassette domain-containing protein	N	N	N	N	N	N
WP_154352241.1	glycoside hydrolase family 104 protein	N	N	N	N	N	N
WP_159360194.1	ABC transporter six-transmembrane domain-containing protein	N	N	N	N	N	N
WP_171953774.1	TetR family transcriptional regulator	N	N	N	N	N	N
WP_191850845.1	alpha/beta hydrolase	N	N	N	N	N	N
WP_197602112.1	LysR substrate-binding domain-containing protein	N	N	N	N	N	N
WP_224328161.1	ABC transporter ATP-binding protein	N	N	N	N	N	N
WP_224328188.1	response regulator	N	N	N	N	N	N
WP_224328189.1	TonB-dependent siderophore receptor	N	N	N	N	N	N
WP_224328203.1	response regulator transcription factor	N	N	N	N	N	N
WP_224328239.1	sigma-70 family RNA polymerase sigma factor [Stenotrophomonas sp. NY11291]	N	N	N	N	N	N
WP_224328285.	FAD-dependent monooxygenase	N	N	N	N	N	N

1							
WP_224328294.<br>1	agmatine deiminase family protein	N	N	N	N	N	N
WP_224328295.<br>1	LysR substrate-binding<br>domain-containing protein	N	N	N	N	N	N
WP_224328332.<br>1	alpha/beta fold hydrolase	N	N	N	N	N	N
WP_224328333.<br>1	alpha/beta hydrolase	N	N	N	N	N	N
WP_224328439.<br>1	response regulator transcription factor	N	N	N	N	N	N
WP_224328516.<br>1	L-seryl-tRNA(Sec) selenium transferase	N	N	N	N	N	N
WP_224328592.<br>1	Gfo/Idh/MocA family oxidoreductase	N	N	N	N	N	N
WP_224328617.<br>1	MFS transporter	N	N	N	N	N	N
WP_224328621.<br>1	CmlA/FloR family chloramphenicol<br>efflux MFS transporter	N	N	N	N	N	N
WP_224328693.<br>1	helix-turn-helix domain-containing<br>protein	N	N	N	N	N	N
WP_224328694.<br>1	MBL fold metallo-hydrolase	N	N	N	N	N	N
WP_224328700.<br>1	IS3 family transposase	N	N	N	N	N	N
WP_224328702.	DUF6691 family protein	N	N	N	N	N	N

1							
WP_224328703.1	sulfite exporter TauE/SafE family protein	N	N	N	N	N	N
WP_224328756.1	cytochrome o ubiquinol oxidase subunit III	N	N	N	N	N	N
WP_224328793.1	DNA polymerase III subunit epsilon	N	N	N	N	N	N
WP_224328847.1	SDR family oxidoreductase	N	N	N	N	N	N
WP_224328848.1	carboxymuconolactone decarboxylase family protein	N	N	N	N	N	N
WP_224328852.1	alkene reductase	N	N	N	N	N	N
WP_224328853.1	thioredoxin	N	N	N	N	N	N
WP_224328855.1	dihydrolipoyl dehydrogenase	N	N	N	N	N	N
WP_224328931.1	MFS transporter	N	N	N	N	N	N
WP_224328932.1	MFS transporter	N	N	N	N	N	N
WP_224328943.1	ABC transporter permease	N	N	N	N	N	N
WP_224328983.1	MFS transporter	N	N	N	N	N	N
WP_224329035.	DUF1064 domain-containing protein	N	N	N	N	N	N

1							
WP_224329068.1	glutathione S-transferase family protein	N	N	N	N	N	N
WP_224329103.1	LysR family transcriptional regulator	N	N	N	N	N	N
WP_224329130.1	aminotransferase class III-fold pyridoxal phosphate-dependent enzyme	N	N	N	N	N	N
WP_224329168.1	carboxymuconolactone decarboxylase family protein	N	N	N	N	N	N
WP_224329174.1	ABC transporter ATP-binding protein	N	N	N	N	N	N
WP_224329182.1	thioesterase family protein	N	N	N	N	N	N
WP_224329184.1	ATP-binding protein	N	N	N	N	N	N
WP_224329229.1	AraC family transcriptional regulator	N	N	N	N	N	N
WP_224329259.1	YjbH domain-containing protein	N	N	N	N	N	N
WP_224329266.1	phage major capsid protein	N	N	N	N	N	N
WP_224329278.1	dihydrodipicolinate synthase family protein	N	N	N	N	N	N
WP_224329279.1	GNAT family N-acetyltransferase	N	N	N	N	N	N
WP_224329335.	GNAT family N-acetyltransferase	N	N	N	N	N	N

	1							
	WP_224329343.1	nucleoside triphosphatase NudI	N	N	N	N	N	N
	WP_224329354.1	aldehyde oxidoreductase molybdenum-binding subunit PaoC	N	N	N	N	N	N
	WP_224329357.1	nuclear transport factor 2 family protein	N	N	N	N	N	N
	WP_224329358.1	TetR family transcriptional regulator	N	N	N	N	N	N
	WP_224329422.1	superoxide dismutase	N	N	N	N	N	N
	WP_224329429.1	heparan-alpha-glucosaminide N-acetyltransferase domain-containing protein	N	N	N	N	N	N
	WP_265096486.1	Rid family hydrolase	N	N	N	N	N	N
	WP_265096487.1	major royal jelly family protein	N	N	N	N	N	N
	WP_265096488.1	major royal jelly family protein	N	N	N	N	N	N
	WP_004146420.1	BREX protein BrxB domain-containing protein	N	N	N	N	N	N
NZ_CP065965.1	WP_004153231.1	DEAD/DEAH box helicase family protein	N	N	N	N	N	N
	WP_005304583.1	helix-turn-helix transcriptional regulator	N	N	N	N	N	N

WP_005408112.1	LysR family transcriptional regulator	N	N	N	N	N	N
WP_005408332.1	GPW/gp25 family protein	N	N	N	N	N	N
WP_005408904.1	nuclear transport factor 2 family protein	N	N	N	N	N	N
WP_005411473.1	cytochrome o ubiquinol oxidase subunit IV	N	N	N	N	N	N
WP_006375900.1	zincin-like metallopeptidase domain-containing protein	N	N	N	N	N	N
WP_012478672.1	helix-turn-helix transcriptional regulator	N	N	N	N	N	N
WP_012479056.1	ABC transporter ATP-binding protein	N	N	N	N	N	N
WP_012479074.1	SMR family transporter	N	N	N	N	N	N
WP_012479075.1	TIM barrel protein	N	N	N	N	N	N
WP_012479076.1	NAD(P)-dependent oxidoreductase	N	N	N	N	N	N
WP_012479077.1	FAD-dependent oxidoreductase	N	N	N	N	N	N
WP_012479078.1	glycosyltransferase	N	N	N	N	N	N
WP_020200523.1	DUF6127 family protein	N	N	N	N	N	N

WP_020200576.1	helix-turn-helix transcriptional regulator	N	N	N	N	N	N
WP_024956190.1	GPW/gp25 family protein	N	N	N	N	N	N
WP_024956193.1	phage tail assembly protein	N	N	N	N	N	N
WP_024956393.1	Hg(II)-responsive transcriptional regulator	N	N	N	N	N	N
WP_024956394.1	mercuric transporter MerT family protein	N	N	N	N	N	N
WP_024956395.1	mercury resistance system periplasmic binding protein MerP	N	N	N	N	N	N
WP_024956414.1	hypothetical protein	N	N	N	N	N	N
WP_024956557.1	alpha/beta hydrolase	N	N	N	N	N	N
WP_024956711.1	DUF2958 domain-containing protein	N	N	N	N	N	N
WP_024956712.1	DUF2285 domain-containing protein	N	N	NZ_CP060022.1-9, NZ_CP060023.1-9, NZ_CP065965.1-5	100	N	N
WP_024956713.1	helix-turn-helix domain-containing protein	N	N	N	N	N	N
WP_024956717.1	DUF2840 domain-containing protein	N	N	NZ_CP060022.1-9, NZ_CP060023.1-9, NZ_CP065965.1-5	100	N	N

WP_024956864.1	adenylate/guanylate cyclase domain-containing protein	N	N	N	N	N	N
WP_024957346.1	glycosyltransferase family 2 protein	N	N	N	N	N	N
WP_024957400.1	DUF305 domain-containing protein	N	N	N	N	N	N
WP_024957601.1	AAA family ATPase	NZ_CP008838.1-2, NZ_LT906480.1-2	100	N	N	NZ_CP065965.1-10	100
WP_024957611.1	hypothetical protein	N	N	N	N	N	N
WP_024957613.1	hypothetical protein	N	N	N	N	N	N
WP_024957746.1	transcriptional regulator	N	N	N	N	N	N
WP_024957931.1	DUF1629 domain-containing protein	N	N	N	N	N	N
WP_024957933.1	metalloregulator ArsR/SmtB family transcription factor	N	N	N	N	N	N
WP_024957960.1	VOC family protein	N	N	N	N	N	N
WP_024958019.1	XRE family transcriptional regulator	N	N	N	N	N	N
WP_024958021.1	DUF3322 and DUF2220 domain-containing protein	N	N	N	N	N	N
WP_024958026.1	BrxE family protein	N	N	N	N	N	N

WP_024958027.1	DUF1819 family protein	N	N	N	N	N	N
WP_024958028.1	BREX system P-loop protein BrxC	N	N	N	N	N	N
WP_024958029.1	N-6 DNA methylase	N	N	N	N	N	N
WP_024958031.1	DUF488 domain-containing protein	N	N	N	N	N	N
WP_024958032.1	PglZ domain-containing protein	NZ_CP008838.1-1	100	N	N	N	N
WP_024958033.1	BREX system Lon protease-like protein BrxL	NZ_CP008838.1-1	100	N	N	N	N
WP_024958034.1	hypothetical protein	N	N	N	N	N	N
WP_024958035.1	DUF2793 domain-containing protein	N	N	N	N	N	N
WP_024958036.1	glycosyltransferase family 2 protein	NZ_CP008838.1-1, NZ_CP065965.1-1, NZ_LT906480.1-5	100	N	N	N	N
WP_024958037.1	phage tail protein	NZ_CP008838.1-1, NZ_CP065965.1-1, NZ_LT906480.1-5	100	N	N	N	N
WP_024958039.1	DUF2163 domain-containing protein	NZ_CP008838.1-1, NZ_CP065965.1-1, NZ_LT906480.1-5	100	N	N	N	N
WP_024958040.	hypothetical protein	NZ_CP008838.1-1,	100	N	N	N	N

1		NZ_CP065965.1-1, NZ_LT906480.1-5					
WP_024958041.1	hypothetical protein	NZ_CP008838.1-1, NZ_CP065965.1-1, NZ_LT906480.1-5	100	N	N	N	N
WP_024958042.1	tail protein	NZ_CP008838.1-1, NZ_CP065965.1-1, NZ_LT906480.1-5	100	N	N	N	N
WP_024958043.1	DUF6441 family protein	NZ_CP008838.1-1, NZ_CP065965.1-1, NZ_LT906480.1-5	100	N	N	N	N
WP_024958044.1	hypothetical protein	N	N	N	N	N	N
WP_024958045.1	hypothetical protein	NZ_CP008838.1-1, NZ_CP065965.1-1, NZ_LT906480.1-5	100	N	N	N	N
WP_024958046.1	hypothetical protein	N	N	N	N	N	N
WP_024958047.1	hypothetical protein	N	N	N	N	N	N
WP_024958049.1	head decoration protein	N	N	N	N	N	N
WP_024958050.1	S49 family peptidase	NZ_CP008838.1-1, NZ_CP065965.1-1, NZ_LT906480.1-5	100	NZ_LT906480.1-8	100	N	N
WP_024958052.	hypothetical protein	N	N	N	N	N	N

1							
WP_024958053.1	hypothetical protein	N	N	N	N	N	N
WP_024958054.1	hypothetical protein	N	N	N	N	N	N
WP_024958058.1	DUF3489 domain-containing protein	NZ_CP008838.1-1, NZ_CP065965.1-1	100	NZ_LT906480.1-8	100	N	N
WP_024958059.1	hypothetical protein	N	N	N	N	N	N
WP_024958060.1	hypothetical protein	N	N	N	N	N	N
WP_024958063.1	DUF6362 family protein	N	N	N	N	N	N
WP_024958064.1	hypothetical protein	N	N	N	N	N	N
WP_024958066.1	phage/plasmid primase, P4 family	NZ_CP008838.1-1, NZ_CP065965.1-1	100			N	N
WP_024958069.1	DUF6511 domain-containing protein	N	N	N	N	N	N
WP_024958070.1	hypothetical protein	NZ_CP008838.1-1, NZ_CP065965.1-1	100			N	N
WP_024958074.1	hypothetical protein	N	N	N	N	N	N
WP_024958075.1	hypothetical protein	N	N	N	N	N	N
WP_024958076.	recombinase family protein	NZ_CP008838.1-1,	100			N	N

1		NZ_CP065965.1-1					
WP_024958077.1	DUF2924 domain-containing protein	N	N	N	N	N	N
WP_024958080.1	ImmA/IrrE family metallo-endopeptidase	N	N	N	N	N	N
WP_024958081.1	hypothetical protein	NZ_CP008838.1-1	100	N	N	N	N
WP_024958082.1	hypothetical protein	NZ_CP008838.1-1	100	N	N	N	N
WP_024958541.1	YafY family protein	N	N	N	N	N	N
WP_024958791.1	ISL3 family transposase	N	N	N	N	N	N
WP_032963337.1	IS3 family transposase	N	N	N	N	N	N
WP_032964292.1	DUF488 domain-containing protein	N	N	N	N	N	N
WP_032964294.1	lysozyme	N	N	N	N	N	N
WP_032964308.1	site-specific DNA-methyltransferase	N	N	N	N	N	N
WP_076738308.1	ATP-binding protein	N	N	N	N	N	N
WP_076738309.1	3'-5' exonuclease	N	N	N	N	N	N
WP_076738391.	DNA cytosine methyltransferase	N	N	N	N	N	N

1							
WP_076738392.1	DUF262 domain-containing protein	N	N	N	N	N	N
WP_143568593.1	hypothetical protein	N	N	N	N	N	N
WP_197697519.1	DNA adenine methylase	N	N	N	N	N	N
WP_197697522.1	YbhB/YbcL family Raf kinase inhibitor-like protein	N	N	N	N	N	N
WP_197697523.1	CBASS oligonucleotide cyclase	N	N	N	N	N	N
WP_223224752.1	DUF3422 family protein	N	N	N	N	N	N
WP_224119399.1	SMI1/KNR4 family protein	N	N	N	N	N	N
WP_231910752.1	SDR family oxidoreductase	N	N	N	N	N	N
WP_231910796.1	OST-HTH/LOTUS domain-containing protein	N	N	N	N	N	N
WP_231910800.1	site-specific DNA-methyltransferase	N	N	N	N	N	N
WP_231910802.1	elements of external origin	N	N	N	N	N	N
WP_231910804.1	phage portal protein	NZ_CP008838.1-1, NZ_CP065965.1-1, NZ_LT906480.1-5	100	NZ_LT906480.1-8	100	N	N

	WP_231910805.1	major capsid protein	NZ_CP008838.1-1, NZ_CP065965.1-1, NZ_LT906480.1-5	100	N	N	N	N
	WP_231910821.1	SDR family oxidoreductase	N	N	N	N	N	N
	WP_231910825.1	Na+/H+ antiporter NhaA	N	N	N	N	N	N
	WP_231910850.1	type II toxin-antitoxin system RelE/ParE family toxin	N	N	N	N	N	N
	WP_231910863.1	NgoMIV family type II restriction endonuclease	N	N	N	N	N	N
	WP_231910988.1	substrate binding domain-containing protein	N	N	N	N	N	N
	WP_232622053.1	AbiEi antitoxin N-terminal domain-containing protein	N	N	N	N	N	N
	WP_269458352.1	replication-associated recombination protein A	N	N	N	N	N	N
NZ_CP067993.1	WP_012510131.1	transcriptional regulator	N	N	N	N	N	N
	WP_032127338.1	GNAT family N-acetyltransferase	N	N	N	N	N	N
	WP_049398403.1	WxcM-like domain-containing protein	N	N	N	N	N	N
	WP_049398405.1	DegT/DnrJ/EryC1/StrS family aminotransferase	N	N	N	N	N	N
	WP_049398866.	isocitrate lyase/PEP mutase family	N	N	N	N	N	N

1	protein						
WP_049423757.1	M24 family metallopeptidase	N	N	N	N	N	N
WP_049424895.1	glycosyltransferase family 2 protein	N	N	N	N	N	N
WP_121504516.1	phage major capsid protein	N	N	NZ_CP067993.1-3		N	N
WP_125892879.1	glucose-1-phosphate cytidylyltransferase	N	N	N	N	N	N
WP_125892883.1	dTDP-4-dehydrorhamnose 3,5-epimerase	N	N	N	N	N	N
WP_126928434.1	metalloregulator ArsR/SmtB family transcription factor	N	N	N	N	N	N
WP_180853761.1	hypothetical protein	N	N	N	N	N	N
WP_201116140.1	LysR family transcriptional regulator	N	N	N	N	N	N
WP_201116626.1	ATP-binding protein	N	N	N	N	N	N
WP_201116654.1	phage major capsid protein	N	N	NZ_CP067993.1-4	100	N	N
WP_201116662.1	phage portal protein	N	N	NZ_CP067993.1-4	100	N	N
WP_201116978.1	hypothetical protein	N	N	N	N	N	N
WP_201116999.	site-specific DNA-methyltransferase	NZ_CP067993.1-2	100	N	N	N	N

1							
WP_201117162.1	ISL3 family transposase	N	N	N	N	N	N
WP_201117808.1	HipA domain-containing protein	N	N	N	N	N	N
WP_201118377.1	LysR family transcriptional regulator	N	N	N	N	N	N
WP_201118663.1	Rrf2 family transcriptional regulator	N	N	N	N	N	N
WP_201118777.1	MFS transporter	N	N	N	N	N	N
WP_201118835.1	DEAD/DEAH box helicase family protein	N	N	N	N	N	N
WP_201118836.1	endonuclease NucS	N	N	N	N	N	N
WP_201118892.1	Gfo/Idh/MocA family oxidoreductase	N	N	N	N	N	N
WP_201118903.1	CDP-glucose 4,6-dehydratase	N	N	N	N	N	N
WP_201118937.1	phage portal protein	N	N	NZ_CP067993.1-3	100	N	N
WP_201119165.1	TlpA disulfide reductase family protein	N	N	N	N	N	N
WP_201119169.1	response regulator	N	N	N	N	N	N
WP_201119171.	ATP-binding protein	N	N	N	N	N	N

	1							
	WP_201119256.1	helix-turn-helix transcriptional regulator	N	N	N	N	N	N
	WP_236494339.1	NAD(P)-dependent alcohol dehydrogenase	N	N	N	N	N	N
	WP_236494343.1	outer membrane beta-barrel protein	N	N	N	N	N	N
	WP_236494366.1	recombinase family protein	N	N	N	N	N	N
	WP_236494391.1	hypothetical protein	N	N	N	N	N	N
	WP_236494403.1	IS3 family transposase	N	N	N	N	N	N
	WP_236494418.1	class I SAM-dependent DNA methyltransferase	N	N	N	N	N	N
	WP_236494422.1	glycosyltransferase	N	N	N	N	N	N
	WP_236494428.1	terminase TerL endonuclease subunit	N	N	NZ_CP067993.1-3	100	N	N
	WP_236494439.1	protein-disulfide reductase DsbD	N	N	N	N	N	N
	WP_236494442.1	cyclophilin-like fold protein	N	N	N	N	N	N
NZ_CP077679.1	WP_004136660.1	putative addiction module antidote protein	N	N	N	N	N	N
	WP_004152082.	carboxymuconolactone decarboxylase	N	N	N	N	N	N

1	family protein						
WP_005408320.1	tautomerase family protein	N	N	N	N	N	N
WP_005408779.1	response regulator	N	N	N	N	N	N
WP_005409726.1	ABC transporter permease	N	N	N	N	N	N
WP_005409874.1	biopolymer transporter ExbD	N	N	N	N	N	N
WP_005411610.1	phosphopantetheine-binding protein	N	N	N	N	N	N
WP_005413654.1	Lrp/AsnC family transcriptional regulator	N	N	N	N	N	N
WP_006402206.1	acyl carrier protein	N	N	N	N	N	N
WP_006425764.1	aminotransferase class III-fold pyridoxal phosphate-dependent enzyme	N	N	N	N	N	N
WP_010481265.1	VOC family protein	N	N	N	N	N	N
WP_010482305.1	hypothetical protein	N	N	N	N	N	N
WP_010482807.1	VOC family protein	N	N	N	N	N	N
WP_010483441.1	alpha/beta hydrolase	N	N	N	N	N	N
WP_010483459.	helix-turn-helix transcriptional regulator	N	N	N	N	N	N

1							
WP_010483779.1	baseplate J/gp47 family protein	N	N	N	N	N	N
WP_010484092.1	winged helix-turn-helix domain-containing protein	N	N	N	N	N	N
WP_010484993.1	Rid family hydrolase	N	N	N	N	N	N
WP_010485179.1	VOC family protein	N	N	N	N	N	N
WP_010485209.1	hypothetical protein	N	N	N	N	N	N
WP_010485313.1	respiratory nitrate reductase subunit gamma	N	N	N	N	N	N
WP_010485668.1	LysR family transcriptional regulator	N	N	N	N	N	N
WP_012479558.1	EamA family transporter	N	N	N	N	N	N
WP_019337795.1	type II toxin-antitoxin system RelE/ParE family toxin	N	N	N	N	N	N
WP_019337810.1	NarK family nitrate/nitrite MFS transporter	N	N	N	N	N	N
WP_019338357.1	alpha/beta hydrolase	N	N	N	N	N	N
WP_019338592.1	PDDEXK nuclease domain-containing protein	N	N	N	N	N	N
WP_019659640.	HigA family addiction module antitoxin	N	N	N	N	N	N

1							
WP_024957423.1	MgtC/SapB family protein	N	N	N	N	N	N
WP_029550852.1	low molecular weight protein-tyrosine-phosphatase	N	N	N	N	N	N
WP_043396259.1	AzlD family protein	N	N	N	N	N	N
WP_043397376.1	hypothetical protein	N	N	N	N	N	N
WP_043397870.1	glycosyl transferase family protein	N	N	N	N	N	N
WP_043398633.1	helix-turn-helix domain-containing protein [Stenotrophomonas sp. NY11291]	N	N	N	N	N	N
WP_043400508.1	response regulator transcription factor	N	N	N	N	N	N
WP_043401935.1	VOC family protein	N	N	N	N	N	N
WP_049409535.1	heavy metal response regulator transcription factor	N	N	N	N	N	N
WP_049433742.1	ABC transporter ATP-binding protein	N	N	N	N	N	N
WP_049433895.1	response regulator	N	N	N	N	N	N
WP_049433897.1	DMT family transporter	N	N	N	N	N	N

WP_049434627.1	helix-turn-helix domain-containing protein	N	N	N	N	N	N
WP_049434910.1	SRPBCC family protein	N	N	N	N	N	N
WP_049434925.1	protein deglycase HchA	N	N	N	N	N	N
WP_049434952.1	ABC transporter ATP-binding protein	N	N	N	N	N	N
WP_049435351.1	LysR family transcriptional regulator	N	N	N	N	N	N
WP_049435431.1	VOC family protein	N	N	N	N	N	N
WP_049435746.1	ubiquinol oxidase subunit II	N	N	N	N	N	N
WP_049435905.1	type II toxin-antitoxin system RelE/ParE family toxin	N	N	N	N	N	N
WP_049435907.1	HigA family addiction module antitoxin	N	N	N	N	N	N
WP_049436013.1	DNA polymerase III subunit epsilon	N	N	N	N	N	N
WP_049436300.1	metalloregulator ArsR/SmtB family transcription factor	N	N	N	N	N	N
WP_049436334.1	LysR family transcriptional regulator	N	N	N	N	N	N
WP_049436417.1	response regulator	N	N	N	N	N	N

WP_049436428.1	type II toxin-antitoxin system RelE/ParE family toxin	N	N	N	N	N	N
WP_049436621.1	DUF2239 family protein	N	N	N	N	N	N
WP_049436683.1	response regulator transcription factor	N	N	N	N	N	N
WP_049436734.1	zinc-dependent alcohol dehydrogenase family protein	N	N	N	N	N	N
WP_049436913.1	alpha/beta hydrolase	N	N	N	N	N	N
WP_049436931.1	MFS transporter	N	N	N	N	N	N
WP_049437383.1	alpha/beta hydrolase	N	N	N	N	N	N
WP_049437387.1	TetR/AcrR family transcriptional regulator	N	N	N	N	N	N
WP_049437620.1	response regulator transcription factor	N	N	N	N	N	N
WP_049437693.1	MerR family transcriptional regulator	N	N	N	N	N	N
WP_049455305.1	ABC transporter ATP-binding protein	N	N	N	N	N	N
WP_053442610.1	GNAT family N-acetyltransferase	N	N	N	N	N	N
WP_053443132.1	response regulator transcription factor	N	N	N	N	N	N

WP_054171045.1	LysR family transcriptional regulator	N	N	N	N	N	N
WP_054171575.1	GNAT family N-acetyltransferase	N	N	N	N	N	N
WP_054171744.1	nucleoside triphosphatase NudI	N	N	N	N	N	N
WP_054171932.1	sigma-70 family RNA polymerase sigma factor	N	N	N	N	N	N
WP_054172022.1	MerR family transcriptional regulator	N	N	N	N	N	N
WP_054172027.1	FAD-dependent monooxygenase	N	N	N	N	N	N
WP_054172101.1	LysR substrate-binding domain-containing protein	N	N	N	N	N	N
WP_054172215.1	SMR family transporter	N	N	N	N	N	N
WP_054172495.1	hypothetical protein	N	N	N	N	N	N
WP_071305278.1	EamA family transporter	N	N	N	N	N	N
WP_071305768.1	SRPBCC domain-containing protein	N	N	N	N	N	N
WP_082783343.1	cytochrome o ubiquinol oxidase subunit III	N	N	N	N	N	N
WP_088023180.1	HigA family addiction module antitoxin	N	N	N	N	N	N

WP_088023182.1	type II toxin-antitoxin system RelE/ParE family toxin	N	N	N	N	N	N
WP_100064753.1	ATP-binding protein	N	N	N	N	N	N
WP_100445944.1	biotin/lipoyl-binding protein	N	N	N	N	N	N
WP_111202554.1	class I SAM-dependent methyltransferase	N	N	N	N	N	N
WP_125436937.1	ATP-binding cassette domain-containing protein	N	N	N	N	N	N
WP_126561141.1	hypothetical protein	N	N	N	N	N	N
WP_134959345.1	type II secretion system major pseudopilin GspG	N	N	N	N	N	N
WP_180836344.1	response regulator	N	N	N	N	N	N
WP_197579149.1	GNAT family N-acetyltransferase	N	N	N	N	N	N
WP_197643862.1	thioesterase family protein	N	N	N	N	N	N
WP_197643866.1	ATP-binding protein	N	N	N	N	N	N
WP_197644018.1	MFS transporter	N	N	N	N	N	N
WP_197644202.1	CmlA/FloR family chloramphenicol efflux MFS transporter	N	N	N	N	N	N

WP_197644223.1	DoxX family protein	N	N	N	N	N	N
WP_197645348.1	diacylglycerol kinase	N	N	N	N	N	N
WP_197645651.1	LysR family transcriptional regulator	N	N	N	N	N	N
WP_197645899.1	Gfo/Idh/MocA family oxidoreductase	N	N	N	N	N	N
WP_197646261.1	dihydrodipicolinate synthase family protein	N	N	N	N	N	N
WP_197646477.1	sugar phosphate isomerase/epimerase family protein	N	N	N	N	N	N
WP_197646870.1	formate dehydrogenase subunit gamma	N	N	N	N	N	N
WP_217013410.1	hypothetical protein	N	N	N	N	N	N
WP_217013431.1	response regulator	N	N	N	N	N	N
WP_217013432.1	ATP-binding protein	N	N	N	N	N	N
WP_217013434.1	ABC transporter six-transmembrane domain-containing protein	N	N	N	N	N	N
WP_217013438.1	TonB-dependent siderophore receptor	N	N	N	N	N	N
WP_217013440.1	PepSY-associated TM helix domain-containing protein	N	N	N	N	N	N

WP_217013444.1	TonB-dependent siderophore receptor	N	N	N	N	N	N
WP_217013490.1	nitrate/nitrite transporter	N	N	N	N	N	N
WP_217013643.1	NADH:flavin oxidoreductase/NADH oxidase family protein	N	N	N	N	N	N
WP_217013697.1	agmatine deiminase family protein	N	N	N	N	N	N
WP_217013841.1	SDR family oxidoreductase	N	N	N	N	N	N
WP_217013844.1	methylated-DNA--[protein]-cysteine S-methyltransferase	N	N	N	N	N	N
WP_217014143.1	L-seryl-tRNA(Sec) selenium transferase	N	N	N	N	N	N
WP_217014145.1	selenocysteine-specific translation elongation factor	N	N	N	N	N	N
WP_217014147.1	selenide, water dikinase SelD	N	N	N	N	N	N
WP_217014527.1	L-dopachrome tautomerase-related protein	N	N	N	N	N	N
WP_217014530.1	LysR family transcriptional regulator	N	N	N	N	N	N
WP_217015106.1	UDP-N-acetylglucosamine 2-epimerase (non-hydrolyzing)	N	N	N	N	N	N
WP_217015308.1	response regulator transcription factor	N	N	N	N	N	N

WP_217015454.1	GyrI-like domain-containing protein	N	N	N	N	N	N
WP_217015614.1	TonB-dependent receptor	N	N	N	N	N	N
WP_217015627.1	alpha/beta hydrolase	N	N	N	N	N	N
WP_217015699.1	SDR family oxidoreductase	N	N	N	N	N	N
WP_217015700.1	DUF2938 domain-containing protein	N	N	N	N	N	N
WP_217015724.1	Cu(I)-responsive transcriptional regulator	N	N	N	N	N	N
WP_217015747.1	transcriptional regulator BetI	N	N	N	N	N	N
WP_217015748.1	BCCT family transporter	N	N	N	N	N	N
WP_217015802.1	aldehyde oxidoreductase molybdenum-binding subunit PaoC	N	N	N	N	N	N
WP_217015804.1	nuclear transport factor 2 family protein	N	N	N	N	N	N
WP_217015805.1	TetR family transcriptional regulator	N	N	N	N	N	N
WP_241888353.1	GNAT family N-acetyltransferase	N	N	N	N	N	N
WP_254113502.1	DUF3224 domain-containing protein	N	N	N	N	N	N

	WP_254113579.1	alpha/beta hydrolase	N	N	N	N	N	N
	WP_254113627.1	MFS transporter	N	N	N	N	N	N
	WP_254113660.1	cellulase family glycosylhydrolase	N	N	N	N	N	N
	WP_275539821.1	helix-turn-helix transcriptional regulator	N	N	N	N	N	N
	WP_302054304.1	NAD(P)-dependent oxidoreductase	N	N	N	N	N	N
	WP_005408332.1	GPW/gp25 family protein	N	N	N	N	N	N
	WP_006399830.1	cytochrome o ubiquinol oxidase subunit IV	N	N	N	N	N	N
	WP_032128280.1	redox-sensitive transcriptional activator SoxR	N	N	N	N	N	N
	WP_042358872.1	metalloregulator ArsR/SmtB family transcription factor	N	N	N	N	N	N
NZ_CP080573.1	WP_049399243.1	MepB family protein	N	N	N	N	N	N
	WP_049400072.1	DinB family protein	N	N	N	N	N	N
	WP_093999576.1	type II toxin-antitoxin system RelE/ParE family toxin	N	N	N	N	N	N
	WP_110711813.1	NAD(P)H-dependent oxidoreductase	N	N	N	N	N	N

WP_110712296.1	LysR family transcriptional regulator	N	N	N	N	N	N
WP_110712305.1	HlyD family secretion protein	N	N	N	N	N	N
WP_110712735.1	YjhX family toxin	N	N	N	N	N	N
WP_153855110.1	MFS transporter	N	N	N	N	N	N
WP_153855337.1	alpha/beta fold hydrolase	N	N	N	N	N	N
WP_153856238.1	acyl-protein synthetase	N	N	N	N	N	N
WP_153856421.1	bifunctional helix-turn-helix transcriptional regulator/GNAT family N-acetyltransferase	N	N	N	N	N	N
WP_164160373.1	helix-turn-helix transcriptional regulator	N	N	N	N	N	N
WP_164161638.1	alpha/beta hydrolase	N	N	N	N	N	N
WP_180846634.1	aromatic acid/H+ symport family MFS transporter	N	N	N	N	N	N
WP_180854402.1	SDR family oxidoreductase	N	N	N	N	N	N
WP_180854405.1	VOC family protein	N	N	N	N	N	N
WP_180854723.	DUF1972 domain-containing protein	N	N	N	N	N	N

1							
WP_180859242.1	NAD(P)-dependent alcohol dehydrogenase	N	N	N	N	N	N
WP_204303192.1	EcsC family protein	N	N	N	N	N	N
WP_248858021.1	LysR family transcriptional regulator	N	N	N	N	N	N
WP_248858286.1	GrpB family protein	N	N	N	N	N	N
WP_248858318.1	M24 family metallopeptidase	N	N	N	N	N	N
WP_248858328.1	nucleotide pyrophosphohydrolase	N	N	N	N	N	N
WP_248859607.1	isocitrate lyase/PEP mutase family protein	N	N	N	N	N	N
WP_248860168.1	LysR family transcriptional regulator	N	N	N	N	N	N
WP_248860379.1	DUF2958 domain-containing protein	N	N	N	N	N	N
WP_248860380.1	SRPBCC family protein	N	N	N	N	N	N
WP_248860516.1	alpha/beta hydrolase	N	N	N	N	N	N
WP_248860517.1	cupin domain-containing protein	N	N	N	N	N	N
WP_248860519.	oxidoreductase	N	N	N	N	N	N

	1							
	WP_248860524.1	FAD-dependent oxidoreductase	N	N	N	N	N	N
	WP_248860525.1	NAD(P)H-dependent oxidoreductase	N	N	N	N	N	N
	WP_248860526.1	LysR family transcriptional regulator	N	N	N	N	N	N
	WP_248860527.1	pirin family protein	N	N	N	N	N	N
	WP_248860530.1	maleylacetoacetate isomerase	N	N	N	N	N	N
	WP_248860531.1	gentisate 1,2-dioxygenase	N	N	N	N	N	N
	WP_248860563.1	multidrug/biocide efflux PACE transporter	N	N	N	N	N	N
	WP_248860565.1	FAD-dependent monooxygenase	N	N	N	N	N	N
	WP_248860566.1	LysR family transcriptional regulator	N	N	N	N	N	N
	WP_248860572.1	LysR family transcriptional regulator	N	N	N	N	N	N
	WP_248860573.1	MFS transporter	N	N	N	N	N	N
	WP_283102588.1	DNA (cytosine-5-)-methyltransferase	N	N	N	N	N	N
NZ_CP083454.1	WP_005407702.	type II toxin-antitoxin system RelE/ParE	N	N	N	N	N	N

NZ_CP088240.1	1	family toxin						
	WP_005407703.1	putative addiction module antidote protein	N	N	N	N	N	N
	WP_005407950.1	winged helix-turn-helix domain-containing protein	N	N	N	N	N	N
	WP_005408042.1	UDP-N-acetylglucosamine 2-epimerase (non-hydrolyzing)	N	N	N	N	N	N
	WP_005408666.1	DMT family transporter	N	N	N	N	N	N
	WP_005408670.1	response regulator	N	N	N	N	N	N
	WP_005408904.1	nuclear transport factor 2 family protein	N	N	N	N	N	N
	WP_005408917.1	SRPBCC family protein	N	N	N	N	N	N
	WP_005409356.1	metalloregulator ArsR/SmtB family transcription factor	N	N	N	N	N	N
	WP_005409401.1	GNAT family N-acetyltransferase	N	N	N	N	N	N
	WP_005409440.1	Cu(I)-responsive transcriptional regulator	N	N	N	N	N	N
	WP_005409501.1	transcriptional regulator BetI	N	N	N	N	N	N
	WP_005409502.1	BCCT family transporter	N	N	N	N	N	N
	WP_005409726.	ABC transporter permease	N	N	N	N	N	N

1							
WP_005409730.1	LysR family transcriptional regulator	N	N	N	N	N	N
WP_005409793.1	sensory rhodopsin transducer	N	N	N	N	N	N
WP_005409813.1	DUF3224 domain-containing protein	N	N	N	N	N	N
WP_005409874.1	biopolymer transporter ExbD	N	N	N	N	N	N
WP_005409958.1	tetratricopeptide repeat protein	N	N	N	N	N	N
WP_005409975.1	sigma-70 family RNA polymerase sigma factor	N	N	N	N	N	N
WP_005410131.1	MerR family transcriptional regulator	N	N	N	N	N	N
WP_005410145.1	helix-turn-helix domain-containing protein	N	N	N	N	N	N
WP_005410442.1	VOC family protein	N	N	N	N	N	N
WP_005410881.1	formate dehydrogenase subunit beta	N	N	N	N	N	N
WP_005410882.1	formate dehydrogenase subunit gamma	N	N	N	N	N	N
WP_005411002.1	LysR family transcriptional regulator	N	N	N	N	N	N
WP_005411280.	hypothetical protein	N	N	N	N	N	N

1							
WP_005411609.1	acyl carrier protein	N	N	N	N	N	N
WP_005411610.1	phosphopantetheine-binding protein	N	N	N	N	N	N
WP_005411640.1	DNA polymerase III subunit epsilon	N	N	N	N	N	N
WP_005413494.1	ABC transporter ATP-binding protein	N	N	N	N	N	N
WP_005413537.1	hypothetical protein	N	N	N	N	N	N
WP_005413562.1	ATP-binding protein	N	N	N	N	N	N
WP_005413603.1	TIGR03571 family LLM class oxidoreductase	N	N	N	N	N	N
WP_005413654.1	Lrp/AsnC family transcriptional regulator	N	N	N	N	N	N
WP_005413725.1	VOC family protein	N	N	N	N	N	N
WP_005413733.1	MerR family transcriptional regulator	N	N	N	N	N	N
WP_005413799.1	RidA family protein	N	N	N	N	N	N
WP_005413886.1	LysR substrate-binding domain-containing protein	N	N	N	N	N	N
WP_005414055.	SMR family transporter	N	N	N	N	N	N

1							
WP_010481646.1	phage major tail tube protein	N	N	N	N	N	N
WP_010481648.1	GPW/gp25 family protein	N	N	N	N	N	N
WP_010483441.1	alpha/beta hydrolase	N	N	N	N	N	N
WP_012479036.1	class I SAM-dependent methyltransferase	N	N	N	N	N	N
WP_012479264.1	flavin reductase family protein	N	N	N	N	N	N
WP_012479990.1	DNA-binding protein	N	N	N	N	N	N
WP_012479994.1	LysR substrate-binding domain-containing protein	N	N	N	N	N	N
WP_012479996.1	SDR family oxidoreductase	N	N	N	N	N	N
WP_012480374.1	ABC transporter six-transmembrane domain-containing protein	N	N	N	N	N	N
WP_012480385.1	EamA family transporter	N	N	N	N	N	N
WP_012480412.1	heavy metal response regulator transcription factor	N	N	N	N	N	N
WP_012480455.1	AzlD family protein	N	N	N	N	N	N
WP_012480468.	respiratory nitrate reductase subunit	N	N	N	N	N	N

1	gamma						
WP_012480472.1	NarK family nitrate/nitrite MFS transporter	N	N	N	N	N	N
WP_012480491.1	helix-turn-helix transcriptional regulator	N	N	N	N	N	N
WP_019659022.1	carboxymuconolactone decarboxylase family protein	N	N	N	N	N	N
WP_019659676.1	response regulator	N	N	N	N	N	N
WP_024956137.1	AraC family transcriptional regulator	N	N	N	N	N	N
WP_024956985.1	helix-turn-helix transcriptional regulator	N	N	N	N	N	N
WP_032963270.1	response regulator	N	N	N	N	N	N
WP_032963456.1	aromatic alcohol reductase	N	N	N	N	N	N
WP_033833493.1	phage tail sheath protein	NZ_CP083454.1-1	100	N	N	N	N
WP_038645740.1	EamA family transporter	N	N	N	N	N	N
WP_038646246.1	type II toxin-antitoxin system HipA family toxin	N	N	N	N	N	N
WP_043033308.1	type II secretion system major pseudopilin GspG	N	N	N	N	N	N
WP_043033321.	hydrolase	N	N	N	N	N	N

1							
WP_043033322.1	LysR family transcriptional regulator	N	N	N	N	N	N
WP_043033528.1	nucleoside triphosphatase NudI	N	N	N	N	N	N
WP_043033545.1	GNAT family N-acetyltransferase	N	N	N	N	N	N
WP_043035115.1	response regulator transcription factor	N	N	N	N	N	N
WP_043035379.1	LysR family transcriptional regulator	N	N	N	N	N	N
WP_043035380.1	MFS transporter	N	N	N	N	N	N
WP_044571374.1	helix-turn-helix domain-containing protein	N	N	N	N	N	N
WP_049395563.1	SDR family oxidoreductase	N	N	N	N	N	N
WP_049395564.1	methylated-DNA--[protein]-cysteine S-methyltransferase	N	N	N	N	N	N
WP_065183849.1	biotin/lipoyl-binding protein	N	N	N	N	N	N
WP_065184103.1	DUF2938 domain-containing protein	N	N	N	N	N	N
WP_072167338.1	arsenate reductase ArsC	N	N	N	N	N	N
WP_076739970.	LysR family transcriptional regulator	N	N	N	N	N	N

1							
WP_076739977.1	ABC transporter ATP-binding protein	N	N	N	N	N	N
WP_080281857.1	cytochrome o ubiquinol oxidase subunit III	N	N	N	N	N	N
WP_080353444.1	sulfite exporter TauE/SafE family protein	N	N	N	N	N	N
WP_099472679.1	ubiquinol oxidase subunit II	N	N	N	N	N	N
WP_099472756.1	VOC family protein	N	N	N	N	N	N
WP_099473012.1	LysE family translocator	N	N	N	N	N	N
WP_099473492.1	DNA-binding transcriptional regulator	N	N	N	N	N	N
WP_099484848.1	selenocysteine-specific translation elongation factor	N	N	N	N	N	N
WP_099527753.1	L-dopachrome tautomerase-related protein	N	N	N	N	N	N
WP_099527806.1	VOC family protein	N	N	N	N	N	N
WP_100437960.1	glutathione S-transferase family protein	N	N	N	N	N	N
WP_100438079.1	L-seryl-tRNA(Sec) selenium transferase	N	N	N	N	N	N
WP_100438143.	Gfo/Idh/MocA family oxidoreductase	N	N	N	N	N	N

1							
WP_100438306.1	DNA cytosine methyltransferase	N	N	N	N	N	N
WP_100438436.1	SRPBCC family protein	N	N	N	N	N	N
WP_100438608.1	MgtC/SapB family protein	N	N	N	N	N	N
WP_100438686.1	putative quinol monooxygenase	N	N	N	N	N	N
WP_100438694.1	helix-turn-helix domain-containing protein	N	N	N	N	N	N
WP_100438697.1	NADH:flavin oxidoreductase/NADH oxidase family protein	N	N	N	N	N	N
WP_100438731.1	agmatine deiminase family protein	N	N	N	N	N	N
WP_100441034.1	ArsO family NAD(P)H-dependent flavin-containing monooxygenase	N	N	N	N	N	N
WP_100441041.1	YeeE/YedE family protein	N	N	N	N	N	N
WP_111722894.1	GyrI-like domain-containing protein	N	N	N	N	N	N
WP_111722918.1	ATP-binding protein	N	N	N	N	N	N
WP_111722972.1	hypothetical protein	N	N	N	N	N	N
WP_111722996.	cyclase family protein	N	N	N	N	N	N

1							
WP_111723013.1	DUF2239 family protein	N	N	N	N	N	N
WP_172476233.1	tautomerase family protein	N	N	N	N	N	N
WP_199849323.1	MBL fold metallo-hydrolase	N	N	N	N	N	N
WP_224119214.1	TIGR03885 family FMN-dependent LLM class oxidoreductase	N	N	N	N	N	N
WP_224119233.1	FAD-dependent monooxygenase	N	N	N	N	N	N
WP_224119264.1	MFS transporter	N	N	N	N	N	N
WP_224119270.1	phage portal protein	NZ_CP083454.1-1	100	N	N	N	N
WP_224119272.1	phage major capsid protein, P2 family	NZ_CP083454.1-1	100	N	N	N	N
WP_224119335.1	MFS transporter	N	N	N	N	N	N
WP_224119349.1	LysR substrate-binding domain-containing protein	N	N	N	N	N	N
WP_224119407.1	metalloregulator ArsR/SmtB family transcription factor	N	N	N	N	N	N
WP_005407935.1	class I SAM-dependent methyltransferase	N	N	N	N	N	N
WP_005408042.	UDP-N-acetylglucosamine 2-epimerase	N	N	N	N	N	N

1	(non-hydrolyzing)						
WP_005408320.1	tautomerase family protein	N	N	N	N	N	N
WP_005408373.1	GNAT family N-acetyltransferase	N	N	N	N	N	N
WP_005408494.1	helix-turn-helix transcriptional regulator	N	N	N	N	N	N
WP_005408666.1	DMT family transporter	N	N	N	N	N	N
WP_005408670.1	response regulator	N	N	N	N	N	N
WP_005408779.1	response regulator	N	N	N	N	N	N
WP_005408917.1	SRPBCC family protein	N	N	N	N	N	N
WP_005409356.1	metalloregulator ArsR/SmtB family transcription factor	N	N	N	N	N	N
WP_005409440.1	Cu(I)-responsive transcriptional regulator	N	N	N	N	N	N
WP_005409501.1	transcriptional regulator BetI	N	N	N	N	N	N
WP_005409502.1	BCCT family transporter	N	N	N	N	N	N
WP_005409726.1	ABC transporter permease	N	N	N	N	N	N
WP_005409793.	sensory rhodopsin transducer	N	N	N	N	N	N

1							
WP_005409868.1	hydrolase	N	N	N	N	N	N
WP_005409874.1	biopolymer transporter ExbD	N	N	N	N	N	N
WP_005409890.1	type II secretion system major pseudopilin GspG	N	N	N	N	N	N
WP_005409898.1	AzlD family protein	N	N	N	N	N	N
WP_005409958.1	tetratricopeptide repeat protein	N	N	N	N	N	N
WP_005410065.1	Rid family hydrolase	N	N	N	N	N	N
WP_005410081.1	putative quinol monooxygenase	N	N	N	N	N	N
WP_005410104.1	helix-turn-helix domain-containing protein	N	N	N	N	N	N
WP_005410131.1	MerR family transcriptional regulator	N	N	N	N	N	N
WP_005410145.1	helix-turn-helix domain-containing protein	N	N	N	N	N	N
WP_005410442.1	VOC family protein	N	N	N	N	N	N
WP_005410882.1	formate dehydrogenase subunit gamma	N	N	N	N	N	N
WP_005411002.	LysR family transcriptional regulator	N	N	N	N	N	N

1							
WP_005411014.1	hypothetical protein	N	N	N	N	N	N
WP_005411161.1	VOC family protein	N	N	N	N	N	N
WP_005411280.1	hypothetical protein	N	N	N	N	N	N
WP_005411609.1	acyl carrier protein	N	N	N	N	N	N
WP_005412462.1	baseplate J/gp47 family protein	N	N	N	N	N	N
WP_005413116.1	LysE family transporter	N	N	N	N	N	N
WP_005413133.1	SDR family oxidoreductase	N	N	N	N	N	N
WP_005413196.1	response regulator	N	N	N	N	N	N
WP_005413494.1	ABC transporter ATP-binding protein	N	N	N	N	N	N
WP_005413604.1	cyclase family protein	N	N	N	N	N	N
WP_005413615.1	response regulator	N	N	N	N	N	N
WP_005413654.1	Lrp/AsnC family transcriptional regulator	N	N	N	N	N	N
WP_005413725.	VOC family protein	N	N	N	N	N	N

1							
WP_005413886.1	LysR substrate-binding domain-containing protein	N	N	N	N	N	N
WP_005414287.1	response regulator transcription factor	N	N	N	N	N	N
WP_005414661.1	DNA-binding transcriptional regulator	N	N	N	N	N	N
WP_005416840.1	respiratory nitrate reductase subunit gamma	N	N	N	N	N	N
WP_012479558.1	EamA family transporter	N	N	N	N	N	N
WP_012479691.1	ABC transporter ATP-binding protein	N	N	N	N	N	N
WP_012479952.1	AraC family transcriptional regulator	N	N	N	N	N	N
WP_012479990.1	DNA-binding protein	N	N	N	N	N	N
WP_012480266.1	Ohr family peroxiredoxin	N	N	N	N	N	N
WP_012480267.1	SDR family oxidoreductase	N	N	N	N	N	N
WP_012480268.1	LysR family transcriptional regulator	N	N	N	N	N	N
WP_012480270.1	carboxymuconolactone decarboxylase family protein	N	N	N	N	N	N
WP_012480271.	cupin domain-containing protein	N	N	N	N	N	N

1							
WP_012480272.1	RNA polymerase sigma-70 factor	N	N	N	N	N	N
WP_012480273.1	MBL fold metallo-hydrolase	N	N	N	N	N	N
WP_012480305.1	fasciclin domain-containing protein	N	N	N	N	N	N
WP_012480412.1	heavy metal response regulator transcription factor	N	N	N	N	N	N
WP_012480466.1	nitrate/nitrite transporter	N	N	N	N	N	N
WP_012480472.1	NarK family nitrate/nitrite MFS transporter	N	N	N	N	N	N
WP_012480499.1	diacylglycerol kinase	N	N	N	N	N	N
WP_019659676.1	response regulator	N	N	N	N	N	N
WP_024956419.1	SRPBCC family protein	N	N	N	N	N	N
WP_032962486.1	response regulator transcription factor	N	N	N	N	N	N
WP_033833148.1	DUF2239 family protein	N	N	N	N	N	N
WP_043033392.1	hypothetical protein	N	N	N	N	N	N
WP_043035013.	alpha/beta hydrolase	N	N	N	N	N	N

1							
WP_044570411.1	MerR family transcriptional regulator	N	N	N	N	N	N
WP_049397448.1	MFS transporter	N	N	N	N	N	N
WP_049397460.1	LysR family transcriptional regulator	N	N	N	N	N	N
WP_049397746.1	MgtC/SapB family protein	N	N	N	N	N	N
WP_049459412.1	glycoside hydrolase family 104 protein	N	N	N	N	N	N
WP_049459486.1	LysR family transcriptional regulator	N	N	N	N	N	N
WP_049460141.1	response regulator	N	N	N	N	N	N
WP_049461019.1	glycosyl transferase family protein	N	N	N	N	N	N
WP_059034103.1	protein deglycase HchA	N	N	NZ_CP088240.1-2	100	N	N
WP_059034228.1	SRPBCC domain-containing protein	N	N	N	N	N	N
WP_060380287.1	glutathione S-transferase family protein	N	N	N	N	N	N
WP_060380553.1	LysR substrate-binding domain-containing protein	N	N	N	N	N	N
WP_062605322.	DNA polymerase III subunit epsilon	N	N	N	N	N	N

1							
WP_062605454.1	ubiquinol oxidase subunit II	N	N	N	N	N	N
WP_062605567.1	DoxX family protein	N	N	N	N	N	N
WP_062605583.1	MFS transporter	N	N	N	N	N	N
WP_062605716.1	selenide, water dikinase SelD	N	N	N	N	N	N
WP_062605718.1	L-seryl-tRNA(Sec) selenium transferase	N	N	N	N	N	N
WP_062605833.1	L-dopachrome tautomerase-related protein	N	N	N	N	N	N
WP_062605862.1	selenocysteine-specific translation elongation factor	N	N	N	N	N	N
WP_062606223.1	sigma-70 family RNA polymerase sigma factor	N	N	N	N	N	N
WP_062606366.1	VOC family protein	N	N	N	N	N	N
WP_062606476.1	SMR family transporter	N	N	N	N	N	N
WP_062606486.1	methylated-DNA--[protein]-cysteine S-methyltransferase	N	N	N	N	N	N
WP_062606487.1	SDR family oxidoreductase	N	N	N	N	N	N
WP_062606494.	alpha/beta hydrolase	N	N	N	N	N	N

1							
WP_062606496.1	sugar phosphate isomerase/epimerase family protein	N	N	N	N	N	N
WP_062606498.1	TetR/AcrR family transcriptional regulator	N	N	N	N	N	N
WP_062606561.1	biotin/lipoyl-binding protein	N	N	N	N	N	N
WP_062606609.1	LysR substrate-binding domain-containing protein	N	N	N	N	N	N
WP_062606698.1	alpha/beta hydrolase	N	N	N	N	N	N
WP_062606751.1	GyrI-like domain-containing protein	N	N	N	N	N	N
WP_062606837.1	aminotransferase class III-fold pyridoxal phosphate-dependent enzyme	N	N	N	N	N	N
WP_062607032.1	nucleoside triphosphatase NudI	N	N	N	N	N	N
WP_062607048.1	aldehyde oxidoreductase molybdenum-binding subunit PaoC	N	N	N	N	N	N
WP_062607069.1	aromatic alcohol reductase	N	N	N	N	N	N
WP_062607108.1	glucose 1-dehydrogenase	N	N	N	N	N	N
WP_062607154.1	TIGR03885 family FMN-dependent LLM class oxidoreductase	N	N	N	N	N	N
WP_062607160.	ATP-binding protein	N	N	N	N	N	N

1							
WP_062607162.1	TonB-dependent siderophore receptor	N	N	N	N	N	N
WP_062607183.1	2,5-didehydrogluconate reductase DkgB	N	N	N	N	N	N
WP_062607185.1	TIGR03571 family LLM class oxidoreductase	N	N	N	N	N	N
WP_062607218.1	LysR family transcriptional regulator	N	N	N	N	N	N
WP_062607309.1	NADH:flavin oxidoreductase/NADH oxidase family protein	N	N	N	N	N	N
WP_062607352.1	agmatine deiminase family protein	N	N	N	N	N	N
WP_062607893.1	VOC family protein	N	N	N	N	N	N
WP_062608072.1	TetR family transcriptional regulator	N	N	N	N	N	N
WP_062608074.1	MFS transporter	N	N	N	N	N	N
WP_062608076.1	MFS transporter	N	N	N	N	N	N
WP_062608101.1	winged helix-turn-helix domain-containing protein	N	N	N	N	N	N
WP_062608115.1	UDP-glucose 4-epimerase GalE	N	N	N	N	N	N
WP_080281857.	cytochrome o ubiquinol oxidase subunit	N	N	N	N	N	N

1	III						
WP_080398666.1	glycosyltransferase family 2 protein	N	N	N	N	N	N
WP_099473637.1	ATP-binding protein	N	N	N	N	N	N
WP_099561649.1	carboxymuconolactone decarboxylase family protein	N	N	N	N	N	N
WP_154267843.1	hypothetical protein	N	N	N	N	N	N
WP_223846393.1	metalloregulator ArsR/SmtB family transcription factor	N	N	N	N	N	N
WP_229298673.1	ABC transporter six-transmembrane domain-containing protein	N	N	N	N	N	N
WP_231098231.1	helix-turn-helix domain-containing protein	N	N	N	N	N	N
WP_231336597.1	FAD-dependent monooxygenase	N	N	N	N	N	N
WP_231336640.1	Gfo/Idh/MocA family oxidoreductase	N	N	N	N	N	N
WP_231336678.1	type II toxin-antitoxin system HipA family toxin	N	N	N	N	N	N
WP_231336769.1	phage tail sheath subtilisin-like domain-containing protein	NZ_CP088240.1-1	100	N	N	N	N
WP_231336793.1	nuclear transport factor 2 family protein	N	N	N	N	N	N
WP_231336795.	LysR family transcriptional regulator	N	N	N	N	N	N

	1							
	WP_231336822.1	DUF2938 domain-containing protein	N	N	N	N	N	N
	WP_267257587.1	very short patch repair endonuclease	N	N	N	N	N	N
	WP_269216952.1	helix-turn-helix transcriptional regulator	N	N	N	N	N	N
	WP_269216968.1	DNA (cytosine-5-)-methyltransferase	N	N	N	N	N	N
	WP_005411449.1	arsenate reductase (glutaredoxin)	N	N	N	N	N	N
	WP_005411473.1	cytochrome o ubiquinol oxidase subunit IV	N	N	N	N	N	N
	WP_005413341.1	replication-associated recombination protein A	N	N	N	N	N	N
	WP_017354485.1	excinuclease ABC subunit UvrB	N	N	N	N	N	N
NZ_CP088241.1	WP_017355006.1	AAA family ATPase	N	N	N	N	N	N
	WP_024956822.1	helix-turn-helix domain-containing protein	N	N	N	N	N	N
	WP_026070508.1	NAD(P)/FAD-dependent oxidoreductase	N	N	N	N	N	N
	WP_026070509.1	DUF3422 family protein	N	N	N	N	N	N
	WP_026070510.	sigma-54 dependent transcriptional	N	N	N	N	N	N

1	regulator						
WP_026070607.1	DUF6602 domain-containing protein	N	N	N	N	N	N
WP_026070608.1	hypothetical protein	N	N	N	N	N	N
WP_026070609.1	CBASS oligonucleotide cyclase	N	N	N	N	N	N
WP_037590709.1	NAD(P)/FAD-dependent oxidoreductase	N	N	N	N	N	N
WP_049396673.1	GIY-YIG nuclease family protein	N	N	N	N	N	N
WP_062607032.1	nucleoside triphosphatase NudI	N	N	N	N	N	N
WP_080356717.1	7-carboxy-7-deazaguanine synthase	N	N	NZ_CP088241.1-4	100	N	N
WP_099484974.1	metalloregulator ArsR/SmtB family transcription factor	N	N	N	N	N	N
WP_099527074.1	acyl-protein synthetase	N	N	N	N	N	N
WP_099596820.1	7-cyano-7-deazaguanine synthase QueC	N	N	NZ_CP088241.1-4	100	N	N
WP_111118423.1	acyl carrier protein	N	N	N	N	N	N
WP_114618472.1	TIGR03758 family integrating conjugative element protein	N	N	N	N	N	N
WP_114618930.	DNA cytosine methyltransferase	N	N	N	N	N	N

	1							
	WP_114618931.1	endonuclease	N	N	N	N	N	N
	WP_229298665.1	GlxA family transcriptional regulator	N	N	N	N	N	N
	WP_231347366.1	hypothetical protein	N	N	N	N	N	N
	WP_231347408.1	DNA methyltransferase	N	N	N	N	N	N
	WP_231347455.1	SDR family oxidoreductase	N	N	N	N	N	N
	WP_231347490.1	nuclear transport factor 2 family protein	N	N	N	N	N	N
	WP_231347513.1	arsenic transporter	N	N	N	N	N	N
	WP_231347522.1	GPW/gp25 family protein	N	N	N	N	N	N
	WP_003132004.1	broad-spectrum mercury transporter MerE	N	N	N	N	N	N
	WP_010921730.1	recombinase family protein	N	N	N	N	N	N
NZ_CP088242.1	WP_012509968.1	ABC transporter ATP-binding protein	N	N	N	N	N	N
	WP_021201829.1	helix-turn-helix transcriptional regulator	N	N	N	N	N	N
	WP_031269107.	DUF305 domain-containing protein	N	N	N	N	N	N

1							
WP_043033500.1	hypothetical protein	N	N	N	N	N	N
WP_045890798.1	DUF3330 domain-containing protein	N	N	N	N	N	N
WP_049465767.1	transcriptional regulator	N	N	N	N	N	N
WP_049465972.1	helix-turn-helix domain-containing protein	N	N	N	N	N	N
WP_049469197.1	GPW/gp25 family protein	N	N	N	N	N	N
WP_049470268.1	glycosyltransferase family 2 protein	N	N	N	N	N	N
WP_130768164.1	DUF262 domain-containing protein	N	N	N	N	N	N
WP_130768699.1	AAA family ATPase	N	N	N	N	N	N
WP_130768704.1	helix-turn-helix transcriptional regulator	N	N	N	N	N	N
WP_148239545.1	GPW/gp25 family protein	N	N	N	N	N	N
WP_150827605.1	BPL-N domain-containing protein	N	N	N	N	N	N
WP_188252521.1	DNA cytosine methyltransferase	NZ_CP088242.1-1, NZ_CP088243.1-1	100	N	N	N	N
WP_197585579.	YbhB/YbcL family Raf kinase	N	N	N	N	N	N

	1	inhibitor-like protein					
	WP_231353005.1	Dam family site-specific DNA-(adenine-N6)-methyltransferase	N	N	N	N	N	N
	WP_231353006.1	DUF87 domain-containing protein	N	N	N	N	N	N
	WP_231353045.1	AAA family ATPase	N	N	N	N	N	N
	WP_231353159.1	ATP-dependent helicase	N	N	N	N	N	N
	WP_003132004.1	broad-spectrum mercury transporter MerE	N	N	N	N	N	N
	WP_010921730.1	recombinase family protein	N	N	N	N	N	N
	WP_012509968.1	ABC transporter ATP-binding protein	N	N	N	N	N	N
	WP_021201829.1	helix-turn-helix transcriptional regulator	N	N	N	N	N	N
NZ_CP088243.1	WP_031269107.1	DUF305 domain-containing protein	N	N	N	N	N	N
	WP_043033500.1	hypothetical protein	N	N	N	N	N	N
	WP_045890798.1	DUF3330 domain-containing protein	N	N	N	N	N	N
	WP_049465767.1	transcriptional regulator	N	N	N	N	N	N
	WP_049465972.	helix-turn-helix domain-containing	N	N	N	N	N	N

1	protein						
WP_049469197.1	GPW/gp25 family protein	N	N	N	N	N	N
WP_049470268.1	glycosyltransferase family 2 protein	N	N	N	N	N	N
WP_130768164.1	DUF262 domain-containing protein	N	N	N	N	N	N
WP_130768699.1	AAA family ATPase	N	N	N	N	N	N
WP_130768704.1	helix-turn-helix transcriptional regulator	N	N	N	N	N	N
WP_148239545.1	GPW/gp25 family protein	N	N	N	N	N	N
WP_150827605.1	BPL-N domain-containing protein	N	N	N	N	N	N
WP_188252521.1	DNA cytosine methyltransferase	NZ_CP088242.1-1, NZ_CP088243.1-1	100	N	N	N	N
WP_197585579.1	YbhB/YbcL family Raf kinase inhibitor-like protein	N	N	N	N	N	N
WP_231353005.1	Dam family site-specific DNA-(adenine-N6)-methyltransferase	N	N	N	N	N	N
WP_231353006.1	DUF87 domain-containing protein	N	N	N	N	N	N
WP_231353045.1	AAA family ATPase	N	N	N	N	N	N
WP_231353159.	ATP-dependent helicase	N	N	N	N	N	N

	1							
	WP_000995360.1	mercury resistance co-regulator MerD	N	N	N	N	N	N
	WP_003132004.1	broad-spectrum mercury transporter MerE	N	N	N	N	N	N
	WP_005409644.1	hypothetical protein	N	N	N	N	N	N
	WP_005409655.1	IS110 family transposase	N	N	NZ_CP088244.1-3	100	NZ_CP060025.1-2, NZ_CP060027.1-4, NZ_CP098483.1-4	100
	WP_005409659.1	heat resistance system K+/H+ antiporter KefB-GI	NZ_CP060026.1-2	100	NZ_CP040440.1-7, NZ_CP088244.1-3	100	NZ_CP060025.1-2, NZ_CP060027.1-4, NZ_CP098483.1-4	100
NZ_CP088244.1	WP_005409663.1	heat resistance protein YfdX2	NZ_CP060026.1-2	100	N	N	NZ_CP060025.1-2, NZ_CP060027.1-4, NZ_CP098483.1-4, NZ_CP102942.1-4	100
	WP_005409664.1	heat resistance protein YfdX1	NZ_CP060026.1-2	100	N	N	NZ_CP060025.1-2, NZ_CP060027.1-4, NZ_CP102942.1-4, NZ_CP098483.1-4	100
	WP_005409665.1	small heat shock protein sHSP20-GI	N	N	N	N	N	N
	WP_005409669.1	Hsp20/alpha crystallin family protein	NZ_CP060026.1-2	100	NZ_CP040431.1-4, NZ_CP040433.1-2, NZ_CP040440.1-7,	100	NZ_CP060025.1-2, NZ_CP060027.1-4, NZ_CP102942.1-4,	100

				NZ_CP088244.1-4		NZ_CP098483.1-4	
WP_005411786.1	efflux RND transporter periplasmic adaptor subunit	N	N	N	N	N	N
WP_005413401.1	helix-turn-helix transcriptional regulator	N	N	N	N	NZ_CP043578.1-2	100
WP_005414983.1	ATP-binding protein	N	N	N	N	N	N
WP_010921730.1	recombinase family protein	N	N	N	N	N	N
WP_012478642.1	UvrD-helicase domain-containing protein	N	N	N	N	N	N
WP_021202108.1	metalloregulator ArsR/SmtB family transcription factor	N	N	N	N	N	N
WP_031269107.1	DUF305 domain-containing protein	N	N	N	N	N	N
WP_032960046.1	hypothetical protein	N	N	N	N	N	N
WP_032960048.1	site-specific integrase	N	N	N	N	N	N
WP_032961707.1	helix-turn-helix domain-containing protein	N	N	N	N	N	N
WP_032961710.1	HdeD family acid-resistance protein	N	N	N	N	N	N
WP_043033500.1	hypothetical protein	N	N	N	N	N	N
WP_045890798.	DUF3330 domain-containing protein	N	N	N	N	N	N

1							
WP_049449467.1	site-specific integrase	NZ_CP088244.1-1, NZ_CP098483.1-1	100	N	N	NZ_CP040433.1-3, NZ_CP060025.1-3	100
WP_061480426.1	YafY family protein	N	N	N	N	N	N
WP_065186635.1	NotI family restriction endonuclease	N	N	N	N	N	N
WP_088496878.1	glycosyltransferase family 2 protein	N	N	N	N	N	N
WP_111191079.1	DNA cytosine methyltransferase	N	N	N	N	N	N
WP_134967688.1	fimbria/pilus chaperone family protein	N	N	N	N	N	N
WP_134967700.1	outer membrane beta-barrel protein	N	N	N	N	N	N
WP_134967712.1	DnaJ C-terminal domain-containing protein	N	N	N	N	N	N
WP_134968111.1	chromate efflux transporter	N	N	N	N	N	N
WP_134968210.1	hypothetical protein	N	N	N	N	N	N
WP_134968438.1	Hachiman antiphage defense system protein HamA	N	N	N	N	N	N
WP_134968439.1	UvrD-helicase domain-containing protein	N	N	N	N	N	N
WP_134968440.	ATP-dependent endonuclease	N	N	N	N	N	N

	1							
	WP_227200205.1	helix-turn-helix transcriptional regulator	N	N	N	N	N	N
	WP_229298564.1	Mov34/MPN/PAD-1 family protein	N	N	N	N	N	N
	WP_231352087.1	hypothetical protein	N	N	N	N	N	N
	WP_231352088.1	cardiolipin synthase	N	N	N	N	N	N
	WP_231352098.1	MFS transporter	N	N	N	N	N	N
	WP_231352269.1	group II intron reverse transcriptase/maturase	N	N	N	N	N	N
	WP_231352312.1	macro domain-containing protein	N	N	NZ_CP088244.1-7	100	N	N
	WP_231352336.1	site-specific DNA-methyltransferase	N	N	N	N	N	N
	WP_231352618.1	hypothetical protein	N	N	N	N	N	N
	WP_231352683.1	DEAD/DEAH box helicase	N	N	N	N	N	N
	WP_231352686.1	hypothetical protein	N	N	N	N	N	N
	WP_231352721.1	bestrophin family protein	N	N	N	N	N	N
NZ_CP090418.1	WP_005408332.	GPW/gp25 family protein	N	N	N	N	N	N

1							
WP_005411473.1	cytochrome o ubiquinol oxidase subunit IV	N	N	N	N	N	N
WP_005413341.1	replication-associated recombination protein A	N	N	N	N	N	N
WP_008786730.1	glutaredoxin family protein	N	N	N	N	N	N
WP_008786732.1	MFS transporter	N	N	N	N	N	N
WP_008786734.1	efflux transporter outer membrane subunit	N	N	N	N	N	N
WP_008786735.1	efflux RND transporter permease subunit	N	N	N	N	N	N
WP_008786736.1	efflux RND transporter periplasmic adaptor subunit	N	N	N	N	N	N
WP_008786737.1	metalloregulator ArsR/SmtB family transcription factor	N	N	N	N	N	N
WP_008786738.1	cation diffusion facilitator family transporter	N	N	N	N	N	N
WP_008786740.1	MFS transporter	N	N	N	N	N	N
WP_008786741.1	metalloregulator ArsR/SmtB family transcription factor	N	N	N	N	N	N
WP_008786745.1	glutaredoxin	N	N	N	N	N	N
WP_008786746.	Lrp/AsnC ligand binding	N	N	N	N	N	N

1	domain-containing protein						
WP_008786747.1	LysR substrate-binding domain-containing protein	N	N	N	N	N	N
WP_008786751.1	CopG family transcriptional regulator	N	N	N	N	N	N
WP_008786753.1	TrbC/VirB2 family protein	N	N	N	N	N	N
WP_008786754.1	VirB3 family type IV secretion system protein	N	N	N	N	N	N
WP_008786756.1	P-type conjugative transfer protein TrbJ	N	N	N	N	N	N
WP_008786757.1	hypothetical protein	N	N	N	N	N	N
WP_008786881.1	TrbI/VirB10 family protein	N	N	N	N	N	N
WP_008786882.1	P-type conjugative transfer protein TrbG	N	N	N	N	N	N
WP_013721771.1	helix-turn-helix domain-containing protein	N	N	N	N	N	N
WP_017354485.1	excinuclease ABC subunit UvrB	N	N	N	N	N	N
WP_017355006.1	AAA family ATPase	N	N	N	N	N	N
WP_017357195.1	isoprenylcysteine carboxylmethyltransferase family protein	N	N	N	N	N	N
WP_019396544.	DUF2840 domain-containing protein	N	N	N	N	N	N

1							
WP_019396545.1	S26 family signal peptidase	N	N	N	N	N	N
WP_021221531.1	P-type conjugative transfer protein TrbL	N	N	N	N	N	N
WP_023087439.1	DUF2958 domain-containing protein	N	N	N	N	N	N
WP_023087440.1	helix-turn-helix transcriptional regulator	N	N	N	N	N	N
WP_023087441.1	DUF736 domain-containing protein	N	N	N	N	N	N
WP_024889339.1	arsenate reductase ArsC	N	N	N	N	N	N
WP_024889345.1	site-specific integrase	N	N	NZ_CP090418.1-3	100	N	N
WP_024957611.1	hypothetical protein	N	N	N	N	N	N
WP_025986882.1	hypothetical protein	N	N	N	N	N	N
WP_026070508.1	NAD(P)/FAD-dependent oxidoreductase	N	N	N	N	N	N
WP_026070510.1	sigma-54 dependent transcriptional regulator	N	N	N	N	N	N
WP_026070607.1	DUF6602 domain-containing protein	N	N	N	N	N	N
WP_026070608.	hypothetical protein	N	N	N	N	N	N

1							
WP_026070609.1	CBASS oligonucleotide cyclase	N	N	N	N	N	N
WP_037590709.1	NAD(P)/FAD-dependent oxidoreductase	N	N	N	N	N	N
WP_049398866.1	isocitrate lyase/PEP mutase family protein	N	N	N	N	N	N
WP_049399935.1	helix-turn-helix transcriptional regulator	N	N	N	N	N	N
WP_049403072.1	DUF3164 family protein	N	N	N	N	NZ_CP090418.1-5	100
WP_049404642.1	heavy metal translocating P-type ATPase	N	N	N	N	N	N
WP_082316348.1	heavy metal translocating P-type ATPase	N	N	NZ_CP040439.1-4	100	N	N
WP_087923870.1	helix-turn-helix domain-containing protein	N	N	N	N	N	N
WP_088025918.1	HigA family addiction module antitoxin	N	N	N	N	N	N
WP_100471313.1	LysR family transcriptional regulator	N	N	N	N	N	N
WP_106467629.1	NAD(P)H-dependent oxidoreductase	N	N	N	N	N	N
WP_106467630.1	LysR family transcriptional regulator	N	N	N	N	N	N
WP_106468708.	metalloregulator ArsR/SmtB family	N	N	N	N	N	N

1	transcription factor						
WP_106469160.1	MepB family protein	N	N	N	N	N	N
WP_125284378.1	chromate efflux transporter	N	N	N	N	N	N
WP_134300639.1	PaeR7I family type II restriction endonuclease	N	N	N	N	N	N
WP_164088554.1	YegP family protein	N	N	N	N	N	N
WP_164089362.1	TIR domain-containing protein	N	N	N	N	N	N
WP_164089365.1	uracil-DNA glycosylase	N	N	N	N	N	N
WP_164089513.1	type II toxin-antitoxin system HipA family toxin	N	N	N	N	N	N
WP_164089849.1	DUF5690 family protein	N	N	N	N	N	N
WP_164090115.1	type II toxin-antitoxin system RelE/ParE family toxin	N	N	N	N	N	N
WP_164090128.1	GDP-mannose 4,6-dehydratase	N	N	N	N	N	N
WP_164090226.1	MBL fold metallo-hydrolase	N	N	N	N	N	N
WP_164090388.1	alpha/beta hydrolase	N	N	N	N	N	N
WP_169708473.	metal-sensing transcriptional repressor	N	N	N	N	N	N

1							
WP_191985840.1	TaqI-like C-terminal specificity domain-containing protein	N	N	N	N	N	N
WP_197597043.1	AAA family ATPase	N	N	N	N	NZ_CP090418.1-5	100
WP_219626381.1	TIGR03364 family FAD-dependent oxidoreductase	N	N	N	N	N	N
WP_234026142.1	tyrosine-type recombinase/integrase	N	N	N	N	N	N
WP_234500054.1	DEAD/DEAH box helicase family protein	N	N	N	N	N	N
WP_234500056.1	class I SAM-dependent DNA methyltransferase	N	N	N	N	N	N
WP_234500123.1	DUF3304 domain-containing protein	N	N	N	N	N	N
WP_234500162.1	type I restriction endonuclease	N	N	N	N	N	N
WP_234500163.1	DEAD/DEAH box helicase family protein	N	N	N	N	N	N
WP_234500179.1	EexN family lipoprotein	N	N	N	N	N	N
WP_234500180.1	DUF2285 domain-containing protein	N	N	N	N	N	N
WP_234500201.1	DUF3422 family protein	N	N	N	N	N	N
WP_234500209.	helix-turn-helix domain-containing	N	N	N	N	N	N

	1	protein						
	WP_003097544.1	IS21-like element ISPa36 family helper ATPase IstB	N	N	N	N	N	N
	WP_003097546.1	IS21-like element ISPa36 family transposase	N	N	N	N	N	N
	WP_003131974.1	mercuric ion transporter MerT	N	N	N	N	N	N
	WP_003131987.1	mercury resistance system periplasmic binding protein MerP	N	N	N	N	N	N
	WP_003156770.1	mercury(II) reductase	N	N	N	N	N	N
	WP_005413341.1	replication-associated recombination protein A	N	N	N	N	N	N
NZ_CP090423.1	WP_005413387.1	Hg(II)-responsive transcriptional regulator	N	N	N	N	N	N
	WP_005416648.1	RebB family R body protein	N	N	N	N	N	N
	WP_005416649.1	RebB family R body protein	N	N	N	N	N	N
	WP_005416651.1	RebB family R body protein	N	N	N	N	N	N
	WP_006375717.1	site-specific DNA-methyltransferase	N	N	N	N	N	N
	WP_010921730.1	recombinase family protein	N	N	N	N	N	N
	WP_017354485.	excinuclease ABC subunit UvrB	N	N	N	N	N	N

1							
WP_017355006.1	AAA family ATPase	N	N	N	N	N	N
WP_026070508.1	NAD(P)/FAD-dependent oxidoreductase	N	N	N	N	N	N
WP_026070509.1	DUF3422 family protein	N	N	N	N	N	N
WP_026070510.1	sigma-54 dependent transcriptional regulator	N	N	N	N	N	N
WP_026070607.1	DUF6602 domain-containing protein	N	N	N	N	N	N
WP_026070608.1	hypothetical protein	N	N	N	N	N	N
WP_026070609.1	CBASS oligonucleotide cyclase	N	N	N	N	N	N
WP_037590709.1	NAD(P)/FAD-dependent oxidoreductase	N	N	N	N	N	N
WP_046984174.1	MFS transporter	N	N	N	N	N	N
WP_053518720.1	DUF2846 domain-containing protein	N	N	N	N	N	N
WP_071227708.1	CusA/CzcA family heavy metal efflux RND transporter	N	N	N	N	N	N
WP_071229638.1	group II intron reverse transcriptase/maturase	NZ_CP090423.1-3	100	N	N	N	N
WP_102947207.	IS5 family transposase	N	N	N	N	N	N

1							
WP_102947323.1	antibiotic biosynthesis monooxygenase	N	N	N	N	N	N
WP_107230625.1	RebB family R body protein	N	N	N	N	N	N
WP_107230645.1	heavy metal response regulator transcription factor	N	N	N	N	N	N
WP_249842003.1	acyl-protein synthetase	N	N	N	N	N	N
WP_249843131.1	helix-turn-helix domain-containing protein	N	N	N	N	N	N
WP_249843379.1	phage terminase large subunit	N	N	NZ_CP090423.1-4	100	N	N
WP_249843534.1	LysR family transcriptional regulator	N	N	N	N	N	N
WP_249843632.1	YjhX family toxin	N	N	N	N	N	N
WP_249844023.1	Tn3-like element TnAs1 family transposase	N	N	N	N	N	N
WP_249844218.1	NAD-dependent epimerase/dehydratase family protein	N	N	N	N	N	N
WP_249844420.1	DUF2280 domain-containing protein	N	N	N	N	N	N
WP_249844421.1	putative metallopeptidase	NZ_CP090423.1-2	100	N	N	N	N
WP_249844751.	type I secretion system permease/ATPase	N	N	N	N	N	N

	1							
	WP_000995360.1	mercury resistance co-regulator MerD	N	N	N	N	N	N
	WP_003132004.1	broad-spectrum mercury transporter MerE	N	N	N	N	N	N
	WP_005408332.1	GPW/gp25 family protein	N	N	N	N	N	N
	WP_005409644.1	hypothetical protein	N	N	N	N	N	N
	WP_005409655.1	IS110 family transposase	N	N	NZ_CP088244.1-3	100	NZ_CP060025.1-2, NZ_CP060027.1-4, NZ_CP098483.1-4	100
NZ_CP098483.1	WP_005409659.1	heat resistance system K+/H+ antiporter KefB-GI	NZ_CP060026.1-2	100	NZ_CP040440.1-7, NZ_CP088244.1-3	100	NZ_CP060025.1-2, NZ_CP060027.1-4, NZ_CP098483.1-4	100
	WP_005409661.1	hypothetical protein	NZ_CP060026.1-2	100	N	N	NZ_CP060025.1-2, NZ_CP060027.1-4, NZ_CP098483.1-4	100
	WP_005409662.1	HdeD family acid-resistance protein	NZ_CP060026.1-2	100	N	N	NZ_CP060025.1-2, NZ_CP060027.1-4, NZ_CP098483.1-4	100
	WP_005409663.1	heat resistance protein YfdX2	NZ_CP060026.1-2	100	N		NZ_CP060025.1-2, NZ_CP060027.1-4, NZ_CP098483.1-4, NZ_CP102942.1-4	100
	WP_005409664.	heat resistance protein YfdX1	NZ_CP060026.1-2	100	N		NZ_CP060025.1-2,	100

1						NZ_CP060027.1-4, NZ_CP102942.1-4, NZ_CP098483.1-4	
WP_005409665.1	small heat shock protein sHSP20-GI	N	N	N	N	N	N
WP_005409667.1	cardiolipin synthase	N	N	N	N	N	N
WP_005409669.1	Hsp20/alpha crystallin family protein	NZ_CP060026.1-2	100	NZ_CP040431.1-4, NZ_CP040433.1-2, NZ_CP040440.1-7, NZ_CP088244.1-4	100	NZ_CP060025.1-2, NZ_CP060027.1-4, NZ_CP102942.1-4, NZ_CP098483.1-4	100
WP_005411473.1	cytochrome o ubiquinol oxidase subunit IV	N	N	N	N	N	N
WP_005413401.1	helix-turn-helix transcriptional regulator	N	N	N	N	NZ_CP043578.1-2	100
WP_010921730.1	recombinase family protein	N	N	N	N	N	N
WP_012509968.1	ABC transporter ATP-binding protein	N	N	N	N	N	N
WP_014645522.1	efflux RND transporter periplasmic adaptor subunit	N	N	N	N	N	N
WP_024956864.1	adenylate/guanylate cyclase domain-containing protein	N	N	N	N	N	N
WP_024957931.1	DUF1629 domain-containing protein	N	N	N	N	N	N
WP_031269107.	DUF305 domain-containing protein	N	N	N	N	N	N

1							
WP_032960048.1	site-specific integrase	N	N	N	N	N	N
WP_032961707.1	helix-turn-helix domain-containing protein	N	N	N	N	N	N
WP_032961836.1	phage terminase large subunit	NZ_CM001824.1-1, NZ_CP014014.1-2, NZ_LR134324.1-1	100	NZ_AP021908.1-1 NZ_CP040431.1-2, NZ_CP040439.1-4, NZ_CP043578.1-1, NZ_CP056088.1-2, NZ_CP060027.1-3, NZ_LR134324.1-3, NZ_CP090418.1-2, NZ_CP104863.1-3, NZ_CP102942.1-3, NZ_CP098483.1-2	100	NZ_CP040430.1-2	100
WP_032965863.1	hypothetical protein	N	N	N	N	N	N
WP_033835782.1	hypothetical protein	N	N	N	N	N	N
WP_043033500.1	hypothetical protein	N	N	N	N	N	N
WP_043033545.1	GNAT family N-acetyltransferase	N	N	N	N	N	N
WP_045890798.1	DUF3330 domain-containing protein	N	N	N	N	N	N

WP_049431274.1	site-specific integrase	N	N	N	N	N	N
WP_049449467.1	site-specific integrase	NZ_CP088244.1-1, NZ_CP098483.1-1	100	N	N	NZ_CP040433.1-3, NZ_CP060025.1-3	100
WP_049455418.1	adenylosuccinate synthetase	N	N	N	N	N	N
WP_065184007.1	hypothetical protein	N	N	N	N	N	N
WP_065426719.1	hypothetical protein	N	N	N	N	N	N
WP_076738943.1	hypothetical protein	N	N	N	N	N	N
WP_076738974.1	DUF1993 domain-containing protein	N	N	N	N	N	N
WP_076739213.1	DUF87 domain-containing protein	N	N	N	N	N	N
WP_076739345.1	zincin-like metallopeptidase domain-containing protein	N	N	N	N	N	N
WP_076739574.1	hypothetical protein	N	N	N	N	N	N
WP_076739824.1	DNA adenine methylase	N	N	N	N	N	N
WP_088429179.1	toll/interleukin-1 receptor domain-containing protein	N	N	N	N	N	N
WP_099473939.1	GtrA family protein	N	N	N	N	N	N

	WP_099562672.1	MazG nucleotide pyrophosphohydrolase domain-containing protein	N	N	N	N	N	N
	WP_183153619.1	ATP-binding protein	N	N	N	N	N	N
	WP_197572742.1	restriction endonuclease	N	N	N	N	N	N
	WP_199175310.1	SIR2 family protein	N	N	N	N	N	N
	WP_224481897.1	nuclease PIN	N	N	N	N	N	N
	WP_229298563.1	TatD family hydrolase	N	N	N	N	N	N
	WP_229298564.1	Mov34/MPN/PAD-1 family protein	N	N	N	N	N	N
	WP_251101610.1	anti-phage ATPase IteA	N	N	N	N	N	N
	WP_251101613.1	VWA domain-containing protein	N	N	N	N	N	N
	WP_005407935.1	class I SAM-dependent methyltransferase	N	N	N	N	N	N
	WP_005408042.1	UDP-N-acetylglucosamine 2-epimerase (non-hydrolyzing)	N	N	N	N	N	N
NZ_CP101622.1	WP_005408320.1	tautomerase family protein	N	N	N	N	N	N
	WP_005408373.1	GNAT family N-acetyltransferase	N	N	N	N	N	N

WP_005408494.1	helix-turn-helix transcriptional regulator	N	N	N	N	N	N
WP_005408666.1	DMT family transporter	N	N	N	N	N	N
WP_005408670.1	response regulator	N	N	N	N	N	N
WP_005408779.1	response regulator	N	N	N	N	N	N
WP_005408850.1	carboxymuconolactone decarboxylase family protein	N	N	N	N	N	N
WP_005408917.1	SRPBCC family protein	N	N	N	N	N	N
WP_005409356.1	metalloregulator ArsR/SmtB family transcription factor	N	N	N	N	N	N
WP_005409440.1	Cu(I)-responsive transcriptional regulator	N	N	N	N	N	N
WP_005409501.1	transcriptional regulator BetI	N	N	N	N	N	N
WP_005409502.1	BCCT family transporter	N	N	N	N	N	N
WP_005409726.1	ABC transporter permease	N	N	N	N	N	N
WP_005409793.1	sensory rhodopsin transducer	N	N	N	N	N	N
WP_005409868.1	hydrolase	N	N	N	N	N	N

WP_005409874.1	biopolymer transporter ExbD	N	N	N	N	N	N
WP_005409890.1	type II secretion system major pseudopilin GspG	N	N	N	N	N	N
WP_005409898.1	AzlD family protein	N	N	N	N	N	N
WP_005409958.1	tetratricopeptide repeat protein	N	N	N	N	N	N
WP_005410065.1	Rid family hydrolase	N	N	N	N	N	N
WP_005410081.1	putative quinol monooxygenase	N	N	N	N	N	N
WP_005410104.1	helix-turn-helix domain-containing protein	N	N	N	N	N	N
WP_005410131.1	MerR family transcriptional regulator	N	N	N	N	N	N
WP_005410145.1	helix-turn-helix domain-containing protein	N	N	N	N	N	N
WP_005410442.1	VOC family protein	N	N	N	N	N	N
WP_005410881.1	formate dehydrogenase subunit beta	N	N	N	N	N	N
WP_005410882.1	formate dehydrogenase subunit gamma	N	N	N	N	N	N
WP_005411002.1	LysR family transcriptional regulator	N	N	N	N	N	N

WP_005411014.1	hypothetical protein	N	N	N	N	N	N
WP_005411161.1	VOC family protein	N	N	N	N	N	N
WP_005411280.1	hypothetical protein	N	N	N	N	N	N
WP_005411594.1	hypothetical protein	N	N	N	N	N	N
WP_005411609.1	acyl carrier protein	N	N	N	N	N	N
WP_005412462.1	baseplate J/gp47 family protein	N	N	N	N	N	N
WP_005412840.1	LysR family transcriptional regulator	N	N	N	N	N	N
WP_005413116.1	LysE family transporter	N	N	N	N	N	N
WP_005413133.1	SDR family oxidoreductase	N	N	N	N	N	N
WP_005413196.1	response regulator	N	N	N	N	N	N
WP_005413494.1	ABC transporter ATP-binding protein	N	N	N	N	N	N
WP_005413604.1	cyclase family protein	N	N	N	N	N	N
WP_005413615.1	response regulator	N	N	N	N	N	N

WP_005413654.1	Lrp/AsnC family transcriptional regulator	N	N	N	N	N	N
WP_005413725.1	VOC family protein	N	N	N	N	N	N
WP_005413886.1	LysR substrate-binding domain-containing protein	N	N	N	N	N	N
WP_005414287.1	response regulator transcription factor	N	N	N	N	N	N
WP_005414661.1	DNA-binding transcriptional regulator	N	N	N	N	N	N
WP_005416840.1	respiratory nitrate reductase subunit gamma	N	N	N	N	N	N
WP_012479558.1	EamA family transporter	N	N	N	N	N	N
WP_012479691.1	ABC transporter ATP-binding protein	N	N	N	N	N	N
WP_012479952.1	AraC family transcriptional regulator	N	N	N	N	N	N
WP_012479990.1	DNA-binding protein	N	N	N	N	N	N
WP_012480266.1	Ohr family peroxiredoxin	N	N	N	N	N	N
WP_012480267.1	SDR family oxidoreductase	N	N	N	N	N	N
WP_012480268.1	LysR family transcriptional regulator	N	N	N	N	N	N

WP_012480270.1	carboxymuconolactone decarboxylase family protein	N	N	N	N	N	N
WP_012480271.1	cupin domain-containing protein	N	N	N	N	N	N
WP_012480272.1	RNA polymerase sigma-70 factor	N	N	N	N	N	N
WP_012480273.1	MBL fold metallo-hydrolase	N	N	N	N	N	N
WP_012480412.1	heavy metal response regulator transcription factor	N	N	N	N	N	N
WP_012480466.1	nitrate/nitrite transporter	N	N	N	N	N	N
WP_012480472.1	NarK family nitrate/nitrite MFS transporter	N	N	N	N	N	N
WP_012480499.1	diacylglycerol kinase	N	N	N	N	N	N
WP_019659676.1	response regulator	N	N	N	N	N	N
WP_024956419.1	SRPBCC family protein	N	N	N	N	N	N
WP_032962486.1	response regulator transcription factor	N	N	N	N	N	N
WP_033833148.1	DUF2239 family protein	N	N	N	N	N	N
WP_038646246.1	type II toxin-antitoxin system HipA family toxin	N	N	N	N	N	N

WP_043033392.1	hypothetical protein	N	N	N	N	N	N
WP_043035013.1	alpha/beta hydrolase	N	N	N	N	N	N
WP_044570411.1	MerR family transcriptional regulator	N	N	N	N	N	N
WP_049397448.1	MFS transporter	N	N	N	N	N	N
WP_049397460.1	LysR family transcriptional regulator	N	N	N	N	N	N
WP_049397746.1	MgtC/SapB family protein	N	N	N	N	N	N
WP_049459412.1	glycoside hydrolase family 104 protein	N	N	N	N	N	N
WP_049459486.1	LysR family transcriptional regulator	N	N	N	N	N	N
WP_049460141.1	response regulator	N	N	N	N	N	N
WP_049461019.1	glycosyl transferase family protein	N	N	N	N	N	N
WP_059034103.1	protein deglycase HchA	N	N	NZ_CP088240.1-2	100	N	N
WP_059034228.1	SRPBCC domain-containing protein	N	N	N	N	N	N
WP_060380287.1	glutathione S-transferase family protein	N	N	N	N	N	N

WP_060380553.1	LysR substrate-binding domain-containing protein	N	N	N	N	N	N
WP_062605322.1	DNA polymerase III subunit epsilon	N	N	N	N	N	N
WP_062605454.1	ubiquinol oxidase subunit II	N	N	N	N	N	N
WP_062605567.1	DoxX family protein	N	N	N	N	N	N
WP_062605583.1	MFS transporter	N	N	N	N	N	N
WP_062605716.1	selenide, water dikinase SelD	N	N	N	N	N	N
WP_062605718.1	L-seryl-tRNA(Sec) selenium transferase	N	N	N	N	N	N
WP_062605854.1	Gfo/Idh/MocA family oxidoreductase	N	N	N	N	N	N
WP_062605862.1	selenocysteine-specific translation elongation factor	N	N	N	N	N	N
WP_062606223.1	sigma-70 family RNA polymerase sigma factor	N	N	N	N	N	N
WP_062606366.1	VOC family protein	N	N	N	N	N	N
WP_062606476.1	SMR family transporter	N	N	N	N	N	N
WP_062606486.1	methylated-DNA--[protein]-cysteine S-methyltransferase	N	N	N	N	N	N

WP_062606487.1	SDR family oxidoreductase	N	N	N	N	N	N
WP_062606494.1	alpha/beta hydrolase	N	N	N	N	N	N
WP_062606496.1	sugar phosphate isomerase/epimerase family protein	N	N	N	N	N	N
WP_062606498.1	TetR/AcrR family transcriptional regulator	N	N	N	N	N	N
WP_062606557.1	helix-turn-helix transcriptional regulator	N	N	N	N	N	N
WP_062606593.1	phage tail sheath subtilisin-like domain-containing protein	CP078102.1-1, NZ_CP101622.1-2	100	N	N	N	N
WP_062606609.1	LysR substrate-binding domain-containing protein	N	N	N	N	N	N
WP_062606698.1	alpha/beta hydrolase	N	N	N	N	N	N
WP_062606751.1	GyrI-like domain-containing protein	N	N	N	N	N	N
WP_062606837.1	aminotransferase class III-fold pyridoxal phosphate-dependent enzyme	N	N	N	N	N	N
WP_062607032.1	nucleoside triphosphatase NudI	N	N	N	N	N	N
WP_062607048.1	aldehyde oxidoreductase molybdenum-binding subunit PaoC	N	N	N	N	N	N
WP_062607108.1	glucose 1-dehydrogenase	N	N	N	N	N	N

WP_062607154.1	TIGR03885 family FMN-dependent LLM class oxidoreductase	N	N	N	N	N	N
WP_062607160.1	ATP-binding protein	N	N	N	N	N	N
WP_062607183.1	2,5-didehydrogluconate reductase DkgB	N	N	N	N	N	N
WP_062607185.1	TIGR03571 family LLM class oxidoreductase	N	N	N	N	N	N
WP_062607218.1	LysR family transcriptional regulator	N	N	N	N	N	N
WP_062607309.1	NADH:flavin oxidoreductase/NADH oxidase family protein	N	N	N	N	N	N
WP_062607352.1	agmatine deiminase family protein	N	N	N	N	N	N
WP_062608072.1	TetR family transcriptional regulator	N	N	N	N	N	N
WP_062608074.1	MFS transporter	N	N	N	N	N	N
WP_062608076.1	MFS transporter	N	N	N	N	N	N
WP_062608101.1	winged helix-turn-helix domain-containing protein	N	N	N	N	N	N
WP_062608115.1	UDP-glucose 4-epimerase GalE	N	N	N	N	N	N
WP_080281857.1	cytochrome o ubiquinol oxidase subunit III	N	N	N	N	N	N

WP_080398666.1	glycosyltransferase family 2 protein	N	N	N	N	N	N
WP_087802826.1	ISL3 family transposase	N	N	CP078102.1-3	100	N	N
WP_099473467.1	ISL3 family transposase	N	N	N	N	N	N
WP_099473637.1	ATP-binding protein	N	N	N	N	N	N
WP_154267843.1	hypothetical protein	N	N	N	N	N	N
WP_197584338.1	fasciclin domain-containing protein	N	N	N	N	N	N
WP_223846393.1	metalloregulator ArsR/SmtB family transcription factor	N	N	N	N	N	N
WP_229295581.1	NmrA family NAD(P)-binding protein	N	N	N	N	N	N
WP_229298673.1	ABC transporter six-transmembrane domain-containing protein	N	N	N	N	N	N
WP_231098209.1	DUF2938 domain-containing protein	N	N	N	N	N	N
WP_231098231.1	helix-turn-helix domain-containing protein	N	N	N	N	N	N
WP_231336597.1	FAD-dependent monooxygenase	N	N	N	N	N	N
WP_231336793.1	nuclear transport factor 2 family protein	N	N	N	N	N	N

WP_267257546.1	DUF1064 domain-containing protein	N	N	N	N	N	N
WP_267257549.1	glycoside hydrolase family 104 protein	N	N	N	N	N	N
WP_267257587.1	very short patch repair endonuclease	N	N	N	N	N	N
WP_267257622.1	L-dopachrome tautomerase-related protein	N	N	N	N	N	N
WP_267257653.1	phage late control D family protein	NZ_CP101622.1-1	100	N	N	N	N
WP_267257657.1	phage tail assembly protein	N	N	N	N	N	N
WP_267257659.1	phage tail sheath protein	NZ_CP101622.1-1	100	N	N	N	N
WP_267257671.1	phage major capsid protein, P2 family	NZ_CP101622.1-1	100	N	N	N	N
WP_267257673.1	terminase ATPase subunit family protein	N	N	N	N	N	N
WP_267257674.1	phage portal protein	NZ_CP101622.1-1	100	N	N	N	N
WP_267257689.1	VOC family protein	N	N	N	N	N	N
WP_267257721.1	efflux RND transporter periplasmic adaptor subunit	N	N	N	N	N	N
WP_267257725.1	TonB-dependent siderophore receptor	N	N	N	N	N	N

	WP_005408332.1	GPW/gp25 family protein	N	N	N	N	N	N
	WP_005409659.1	heat resistance system K+/H+ antiporter KefB-GI	NZ_CP060026.1-2	100	NZ_CP040440.1-7, NZ_CP088244.1-3	100	NZ_CP060025.1-2, NZ_CP060027.1-4, NZ_CP098483.1-4	100
	WP_005409661.1	hypothetical protein	NZ_CP060026.1-2	100	N	N	NZ_CP060025.1-2, NZ_CP060027.1-4, NZ_CP098483.1-4	100
	WP_005409662.1	HdeD family acid-resistance protein	NZ_CP060026.1-2	100	N	N	NZ_CP060025.1-2, NZ_CP060027.1-4, NZ_CP098483.1-4	100
NZ_CP102942.1	WP_005409663.1	heat resistance protein YfdX2	NZ_CP060026.1-2	100	N	N	NZ_CP060025.1-2, NZ_CP060027.1-4, NZ_CP098483.1-4, NZ_CP102942.1-4	100
	WP_005409664.1	heat resistance protein YfdX1	NZ_CP060026.1-2	100	N	N	NZ_CP060025.1-2, NZ_CP060027.1-4, NZ_CP102942.1-4, NZ_CP098483.1-4	100
	WP_005409665.1	small heat shock protein sHSP20-GI	N	N	N	N	N	N
	WP_005409669.1	Hsp20/alpha crystallin family protein	NZ_CP060026.1-2	100	NZ_CP040431.1-4, NZ_CP040433.1-2, NZ_CP040440.1-7, NZ_CP088244.1-4	100	NZ_CP060025.1-2, NZ_CP060027.1-4, NZ_CP102942.1-4, NZ_CP098483.1-4	100
	WP_008267133.	hypothetical protein	N	N	N	N	N	N

1							
WP_019184226.1	hypothetical protein	N	N	N	N	N	N
WP_032961707.1	helix-turn-helix domain-containing protein	N	N	N	N	N	N
WP_032961836.1	phage terminase large subunit	NZ_CM001824.1-1, NZ_CP014014.1-2, NZ_LR134324.1-1	100	NZ_AP021908.1-1, NZ_CP040431.1-2, NZ_CP040439.1-4, NZ_CP043578.1-1, NZ_CP056088.1-2, NZ_CP060027.1-3, NZ_LR134324.1-3, NZ_CP090418.1-2, NZ_CP104863.1-3, NZ_CP102942.1-3, NZ_CP098483.1-2	100	NZ_CP040430.1-2	100
WP_040006894.1	efflux RND transporter periplasmic adaptor subunit	N	N	N	N	N	N
WP_049395161.1	histidine utilization repressor	N	N	N	N	N	N
WP_049395164.1	formimidoylglutamase	N	N	N	N	N	N
WP_049395165.1	DUF305 domain-containing protein	N	N	N	N	N	N
WP_049396030.1	TM0106 family RecB-like putative nuclease	NZ_CP060026.1-2	100	NZ_CP040440.1-7	100	N	N

WP_049396086.1	amidohydrolase	N	N	N	N	N	N
WP_049396991.1	group II intron reverse transcriptase/maturase	N	N	N	N	N	N
WP_049397307.1	IS110 family transposase	NZ_CP060026.1-2	100	NZ_CP040440.1-7	100	NZ_CP102942.1-4	100
WP_049417537.1	gamma-mobile-trio protein GmtX	N	N	N	N	N	N
WP_049417539.1	integrase family protein	N	N	N	N	N	N
WP_049417540.1	gamma-mobile-trio recombinase GmtY	N	N	N	N	N	N
WP_049417866.1	McrC family protein	N	N	N	N	N	N
WP_049418569.1	hypothetical protein	N	N	N	N	N	N
WP_049431274.1	site-specific integrase	N	N	N	N	N	N
WP_049452914.1	hypothetical protein	N	N	N	N	N	N
WP_049452916.1	Dyp-type peroxidase	N	N	N	N	N	N
WP_065426719.1	hypothetical protein	N	N	N	N	N	N
WP_070470375.1	catalase family protein	N	N	N	N	N	N

WP_080052373.1	TatD family hydrolase	N	N	N	N	N	N
WP_080354259.1	SDR family oxidoreductase	N	N	N	N	N	N
WP_087786704.1	ISL3 family transposase	N	N	N	N	N	N
WP_088024719.1	DEAD/DEAH box helicase family protein	N	N	N	N	N	N
WP_088024721.1	DUF262 domain-containing protein	N	N	N	N	N	N
WP_099470480.1	DNA adenine methylase	N	N	N	N	N	N
WP_111128681.1	type II toxin-antitoxin system RelE/ParE family toxin	N	N	N	N	N	N
WP_111128695.1	P-type DNA transfer protein VirB5	N	N	N	N	N	N
WP_111128734.1	antitoxin	N	N	N	N	N	N
WP_197578521.1	TrkH family potassium uptake protein	N	N	N	N	N	N
WP_197578522.1	Trk system potassium transporter TrkA	N	N	N	N	N	N
WP_197579804.1	helix-turn-helix transcriptional regulator	N	N	N	N	N	N
WP_197579806.1	ImmA/IrrE family metallo-endopeptidase	N	N	N	N	N	N

	WP_197579955.1	sigma-70 family RNA polymerase sigma factor	N	N	N	N	N	N
	WP_197697522.1	YbhB/YbcL family Raf kinase inhibitor-like protein	N	N	N	N	N	N
	WP_229298564.1	Mov34/MPN/PAD-1 family protein	N	N	N	N	N	N
	WP_229302632.1	nuclear transport factor 2 family protein	N	N	N	N	N	N
	WP_258389974.1	cardiolipin synthase	N	N	N	N	N	N
	WP_258390003.1	SNF2-related protein	N	N	N	N	N	N
	WP_258390004.1	DNA methyltransferase	N	N	N	N	N	N
	WP_309252304.1	hypothetical protein	N	N	N	N	N	N
	WP_053500298.1	asparaginase	N	N	N	N	N	N
	WP_099560548.1	OmpA family protein	N	N	N	N	N	N
NZ_CP104071.1	WP_171952632.1	nitrilase family protein	N	N	N	N	N	N
	WP_171953748.1	Eco29kI family restriction endonuclease	N	N	N	N	N	N
	WP_171953749.1	DNA cytosine methyltransferase	N	N	N	N	N	N

WP_197654786.1	ATP-binding protein	N	N	N	N	N	N
WP_198422644.1	hypothetical protein	N	N	N	N	N	N
WP_227844139.1	toll/interleukin-1 receptor domain-containing protein	N	N	N	N	N	N
WP_227844547.1	cytochrome c	N	N	N	N	N	N
WP_227844548.1	GMC family oxidoreductase	N	N	N	N	N	N
WP_227844549.1	gluconate 2-dehydrogenase subunit 3 family protein	N	N	N	N	N	N
WP_227844552.1	ATP-binding cassette domain-containing protein	N	N	N	N	N	N
WP_227844553.1	iron chelate uptake ABC transporter family permease subunit	N	N	N	N	N	N
WP_227844557.1	fumarylacetoacetate hydrolase family protein	N	N	N	N	N	N
WP_227844561.1	transcriptional regulator GcvA	N	N	N	N	N	N
WP_227844562.1	carboxymuconolactone decarboxylase family protein	N	N	N	N	N	N
WP_227844565.1	cupin domain-containing protein	N	N	N	N	N	N
WP_227844818.1	terminase TerL endonuclease subunit	N	N	NZ_CP104071.1-2	100	N	N

NZ_CP104169.1	WP_227844821.1	phage major capsid protein	N	N	NZ_CP104071.1-2	100	N	N
	WP_227844886.1	type II toxin-antitoxin system RelE/ParE family toxin	N	N	N	N	N	N
	WP_260833100.1	GDP-mannose 4,6-dehydratase	N	N	N	N	N	N
	WP_004152082.1	carboxymuconolactone decarboxylase family protein	N	N	N	N	N	N
	WP_005408320.1	tautomerase family protein	N	N	N	N	N	N
	WP_005408663.1	aminotransferase class III-fold pyridoxal phosphate-dependent enzyme	N	N	N	N	N	N
	WP_005409726.1	ABC transporter permease	N	N	N	N	N	N
	WP_005409874.1	biopolymer transporter ExbD	N	N	N	N	N	N
	WP_005409898.1	AzlD family protein	N	N	N	N	N	N
	WP_005410423.1	SMR family transporter	N	N	N	N	N	N
	WP_005411610.1	phosphopantetheine-binding protein	N	N	N	N	N	N
	WP_005413654.1	Lrp/AsnC family transcriptional regulator	N	N	N	N	N	N
	WP_006402206.1	acyl carrier protein	N	N	N	N	N	N

WP_010481563.1	response regulator transcription factor	N	N	N	N	N	N
WP_010482111.1	cytochrome o ubiquinol oxidase subunit III	N	N	N	N	N	N
WP_010482305.1	hypothetical protein	N	N	N	N	N	N
WP_010482574.1	Cu(I)-responsive transcriptional regulator	N	N	N	N	N	N
WP_010483185.1	DMT family transporter	N	N	N	N	N	N
WP_010483441.1	alpha/beta hydrolase	N	N	N	N	N	N
WP_010483459.1	helix-turn-helix transcriptional regulator	N	N	N	N	N	N
WP_010483717.1	response regulator transcription factor	N	N	N	N	N	N
WP_010483779.1	baseplate J/gp47 family protein	N	N	N	N	N	N
WP_010483787.1	phage tail sheath subtilisin-like domain-containing protein	NZ_CP011305.1-1, NZ_CP011306.1-1, NZ_CP060259.1-2, NZ_CP077679.1-1, NZ_CP104169.1-1, NZ_OU943334.1-1	100	N	N	N	N
WP_010484073.1	type II toxin-antitoxin system RelE/ParE family toxin	N	N	N	N	N	N

WP_010484074.1	HigA family addiction module antitoxin	N	N	N	N	N	N
WP_010484092.1	winged helix-turn-helix domain-containing protein	N	N	N	N	N	N
WP_010484895.1	helix-turn-helix domain-containing protein	N	N	N	N	N	N
WP_010485179.1	VOC family protein	N	N	N	N	N	N
WP_010485313.1	respiratory nitrate reductase subunit gamma	N	N	N	N	N	N
WP_010485668.1	LysR family transcriptional regulator	N	N	N	N	N	N
WP_010486459.1	VOC family protein	N	N	N	N	N	N
WP_010486485.1	metalloregulator ArsR/SmtB family transcription factor	N	N	N	N	N	N
WP_010486561.1	alpha/beta hydrolase	N	N	N	N	N	N
WP_010486565.1	TetR/AcrR family transcriptional regulator	N	N	N	N	N	N
WP_019336271.1	hypothetical protein	N	N	N	N	N	N
WP_019337245.1	response regulator	N	N	N	N	N	N
WP_019337410.1	hypothetical protein	N	N	N	N	N	N

WP_019337583.1	response regulator	N	N	N	N	N	N
WP_019337773.1	type II secretion system major pseudopilin GspG	N	N	N	N	N	N
WP_019338179.1	response regulator transcription factor	N	N	N	N	N	N
WP_019338970.1	DNA-binding transcriptional regulator	N	N	N	N	N	N
WP_019660267.1	DUF2239 family protein	N	N	N	N	N	N
WP_024957423.1	MgtC/SapB family protein	N	N	N	N	N	N
WP_043396137.1	TIGR03571 family LLM class oxidoreductase	N	N	N	N	N	N
WP_043397376.1	hypothetical protein	N	N	N	N	N	N
WP_043398268.1	LysR substrate-binding domain-containing protein	N	N	N	N	N	N
WP_043400330.1	formate dehydrogenase subunit gamma	N	N	N	N	N	N
WP_043400854.1	SDR family oxidoreductase	N	N	N	N	N	N
WP_043401018.1	Gfo/Idh/MocA family oxidoreductase	N	N	N	N	N	N
WP_049411723.1	hypothetical protein	N	N	N	N	N	N

WP_049412419.1	L-dopachrome tautomerase-related protein	N	N	N	N	N	N
WP_049413071.1	alpha/beta hydrolase	N	N	N	N	N	N
WP_049433742.1	ABC transporter ATP-binding protein	N	N	N	N	N	N
WP_049435351.1	LysR family transcriptional regulator	N	N	N	N	N	N
WP_049452277.1	GPW/gp25 family protein	N	N	N	N	N	N
WP_049454007.1	ubiquinol oxidase subunit II	N	N	N	N	N	N
WP_049455314.1	ATP-binding protein	N	N	N	N	N	N
WP_054172884.1	low molecular weight protein-tyrosine-phosphatase	N	N	N	N	N	N
WP_054172886.1	NAD-dependent epimerase/dehydratase family protein	N	N	N	N	N	N
WP_057502338.1	transcriptional regulator BetI	N	N	N	N	N	N
WP_057502339.1	BCCT family transporter	N	N	N	N	N	N
WP_057502822.1	response regulator	N	N	N	N	N	N
WP_057503530.1	LysR substrate-binding domain-containing protein	N	N	N	N	N	N

WP_071305687.1	cellulase family glycosylhydrolase	N	N	N	N	N	N
WP_099559578.1	winged helix-turn-helix transcriptional regulator	N	N	N	N	N	N
WP_099559651.1	methylated-DNA--[protein]-cysteine S-methyltransferase	N	N	N	N	N	N
WP_100448379.1	LysE family translocator	N	N	N	N	N	N
WP_134957686.1	ATP-binding protein	N	N	N	N	N	N
WP_134957753.1	ABC transporter ATP-binding protein	N	N	N	N	N	N
WP_134958821.1	UDP-N-acetylglucosamine 2-epimerase (non-hydrolyzing)	N	N	N	N	N	N
WP_164191154.1	DUF3224 domain-containing protein	N	N	N	N	N	N
WP_164191783.1	NarK family nitrate/nitrite MFS transporter	N	N	N	N	N	N
WP_164191957.1	ATP-binding protein	N	N	N	N	N	N
WP_164194091.1	class I SAM-dependent methyltransferase	N	N	N	N	N	N
WP_171954223.1	AraC family transcriptional regulator	N	N	N	N	N	N
WP_224329182.1	thioesterase family protein	N	N	N	N	N	N

WP_241877262.1	Rid family hydrolase	N	N	N	N	N	N
WP_260362393.1	MerR family transcriptional regulator	N	N	N	N	N	N
WP_260362394.1	NADH:flavin oxidoreductase/NADH oxidase family protein	N	N	N	N	N	N
WP_260362421.1	agmatine deiminase family protein	N	N	N	N	N	N
WP_260362422.1	LysR family transcriptional regulator	N	N	N	N	N	N
WP_260362428.1	LysR family transcriptional regulator	N	N	N	N	N	N
WP_260362429.1	zinc-dependent alcohol dehydrogenase family protein	N	N	N	N	N	N
WP_260362673.1	sugar phosphate isomerase/epimerase family protein	N	N	N	N	N	N
WP_260363462.1	VOC family protein	N	N	N	N	N	N
WP_260363564.1	alpha/beta hydrolase	N	N	N	N	N	N
WP_260363565.1	helix-turn-helix transcriptional regulator	N	N	N	N	N	N
WP_260363569.1	L-seryl-tRNA(Sec) selenium transferase	N	N	N	N	N	N
WP_260363570.1	selenocysteine-specific translation elongation factor	N	N	N	N	N	N

WP_260363667.1	MFS transporter	N	N	N	N	N	N
WP_260363670.1	CmlA/FloR family chloramphenicol efflux MFS transporter	N	N	N	N	N	N
WP_260363789.1	DNA polymerase III subunit epsilon	N	N	N	N	N	N
WP_260363835.1	type II toxin-antitoxin system HipA family toxin	N	N	N	N	N	N
WP_260363849.1	VOC family protein	N	N	N	N	N	N
WP_260363888.1	phage tail protein	N	N	N	N	N	N
WP_260363893.1	phage major tail tube protein	N	N	N	N	N	N
WP_260363894.1	phage tail sheath protein	NZ_CP104169.1-2	100	N	N	N	N
WP_260363905.1	phage major capsid protein, P2 family	NZ_CP104169.1-2	100	N	N	N	N
WP_260363908.1	phage portal protein	NZ_CP104169.1-2	100	N	N	N	N
WP_260363992.1	MFS transporter	N	N	N	N	N	N
WP_260363993.1	MFS transporter	N	N	N	N	N	N
WP_260364146.1	glutathione S-transferase family protein	N	N	N	N	N	N

WP_260364199.1	class I SAM-dependent DNA methyltransferase	N	N	N	N	N	N
WP_260364233.1	GyrI-like domain-containing protein	N	N	N	N	N	N
WP_260364258.1	EamA family transporter	N	N	N	N	N	N
WP_260364322.1	ABC transporter ATP-binding protein	N	N	N	N	N	N
WP_260364345.1	TonB-dependent receptor	N	N	N	N	N	N
WP_260364382.1	SRPBCC family protein	N	N	N	N	N	N
WP_260364388.1	alpha/beta hydrolase	N	N	N	N	N	N
WP_260364393.1	GNAT family N-acetyltransferase	N	N	N	N	N	N
WP_260364454.1	SDR family oxidoreductase	N	N	N	N	N	N
WP_260364455.1	GNAT family N-acetyltransferase	N	N	N	N	N	N
WP_260364456.1	metalloregulator ArsR/SmtB family transcription factor	N	N	N	N	N	N
WP_260364457.1	DUF2938 domain-containing protein	N	N	N	N	N	N
WP_260364473.1	polysaccharide biosynthesis tyrosine autokinase	N	N	N	N	N	N

WP_260364484.1	YjbH domain-containing protein	N	N	N	N	N	N
WP_260364496.1	dihydrodipicolinate synthase family protein	N	N	N	N	N	N
WP_260364497.1	GNAT family N-acetyltransferase	N	N	N	N	N	N
WP_260364682.1	aldehyde dehydrogenase	N	N	N	N	N	N
WP_260364684.1	GNAT family N-acetyltransferase	N	N	N	N	N	N
WP_260364695.1	nucleoside triphosphatase NudI	N	N	N	N	N	N
WP_260364718.1	nuclear transport factor 2 family protein	N	N	N	N	N	N
WP_260364719.1	TetR family transcriptional regulator	N	N	N	N	N	N
WP_260364766.1	TonB-dependent siderophore receptor	N	N	N	N	N	N
WP_260364768.1	PepSY-associated TM helix domain-containing protein	N	N	N	N	N	N
WP_260364782.1	cyclase family protein	N	N	N	N	N	N
WP_260364784.1	heavy metal response regulator transcription factor	N	N	N	N	N	N
WP_260364791.1	winged helix-turn-helix domain-containing protein	N	N	N	N	N	N

NZ_CP104286.1	WP_260364799.1	LysR family transcriptional regulator	N	N	N	N	N	N
	WP_260364846.1	sigma-70 family RNA polymerase sigma factor	N	N	N	N	N	N
	WP_005408332.1	GPW/gp25 family protein	N	N	N	N	N	N
	WP_005408904.1	nuclear transport factor 2 family protein	N	N	N	N	N	N
	WP_005413341.1	replication-associated recombination protein A	N	N	N	N	N	N
	WP_017355006.1	AAA family ATPase	N	N	N	N	N	N
	WP_021202108.1	metalloregulator ArsR/SmtB family transcription factor	N	N	N	N	N	N
	WP_021204017.1	AAA family ATPase	N	N	N	N	N	N
	WP_026070508.1	NAD(P)/FAD-dependent oxidoreductase	N	N	N	N	N	N
	WP_026070509.1	DUF3422 family protein	N	N	N	N	N	N
	WP_026070510.1	sigma-54 dependent transcriptional regulator	N	N	N	N	N	N
	WP_026070607.1	DUF6602 domain-containing protein	N	N	N	N	N	N
	WP_026070608.1	hypothetical protein	N	N	N	N	N	N

WP_026070609.1	CBASS oligonucleotide cyclase	N	N	N	N	N	N
WP_037590709.1	NAD(P)/FAD-dependent oxidoreductase	N	N	N	N	N	N
WP_080354691.1	EcsC family protein	N	N	N	N	N	N
WP_099479294.1	ATP-binding protein	N	N	N	N	N	N
WP_099479298.1	class I SAM-dependent DNA methyltransferase	N	N	N	N	N	N
WP_099481364.1	hypothetical protein	N	N	N	N	N	N
WP_099481365.1	cupin domain-containing protein	N	N	N	N	N	N
WP_099481367.1	transcriptional regulator GcvA	N	N	N	N	N	N
WP_102947207.1	IS5 family transposase	N	N	N	N	N	N
WP_102947208.1	glutathione-independent formaldehyde dehydrogenase	N	N	N	N	N	N
WP_102947209.1	DUF427 domain-containing protein	N	N	N	N	N	N
WP_102947323.1	antibiotic biosynthesis monooxygenase	N	N	N	N	N	N
WP_227832773.1	restriction endonuclease	N	N	N	N	N	N

	WP_227832889.1	tyrosine-type recombinase/integrase	N	N	N	N	N	N
	WP_227832956.1	DEAD/DEAH box helicase family protein	N	N	N	N	N	N
	WP_227832957.1	helicase-related protein	N	N	N	N	N	N
	WP_227832962.1	MFS transporter	N	N	N	N	N	N
	WP_227832963.1	SDR family oxidoreductase	N	N	N	N	N	N
	WP_227832964.1	SDR family oxidoreductase	N	N	N	N	N	N
	WP_260813481.1	zinc-binding dehydrogenase	N	N	N	N	N	N
	WP_260813483.1	GlxA family transcriptional regulator	N	N	N	N	N	N
	WP_005408494.1	helix-turn-helix transcriptional regulator	N	N	N	N	N	N
	WP_005409501.1	transcriptional regulator BetI	N	N	N	N	N	N
NZ_CP104287.1	WP_005409726.1	ABC transporter permease	N	N	N	N	N	N
	WP_005409874.1	biopolymer transporter ExbD	N	N	N	N	N	N
	WP_005409984.1	MgtC/SapB family protein	N	N	N	N	N	N

WP_005411610.1	phosphopantetheine-binding protein	N	N	N	N	N	N
WP_005413133.1	SDR family oxidoreductase	N	N	N	N	N	N
WP_005413654.1	Lrp/AsnC family transcriptional regulator	N	N	N	N	N	N
WP_005416517.1	response regulator	N	N	N	N	N	N
WP_005416799.1	type II secretion system major pseudopilin GspG	N	N	N	N	N	N
WP_005419760.1	hypothetical protein	N	N	N	N	N	N
WP_005420037.1	ubiquinol oxidase subunit II	N	N	N	N	N	N
WP_005420284.1	hypothetical protein	N	N	N	N	N	N
WP_006402206.1	acyl carrier protein	N	N	N	N	N	N
WP_010482545.1	response regulator	N	N	N	N	N	N
WP_010484073.1	type II toxin-antitoxin system RelE/ParE family toxin	N	N	N	N	N	N
WP_010484895.1	helix-turn-helix domain-containing protein	N	N	N	N	N	N
WP_010485313.1	respiratory nitrate reductase subunit gamma	N	N	N	N	N	N

WP_010485668.1	LysR family transcriptional regulator	N	N	N	N	N	N
WP_012480466.1	nitrate/nitrite transporter	N	N	N	N	N	N
WP_014647218.1	ABC transporter ATP-binding protein	N	N	N	N	N	N
WP_019659002.1	response regulator transcription factor	N	N	N	N	N	N
WP_019659022.1	carboxymuconolactone decarboxylase family protein	N	N	N	N	N	N
WP_019659087.1	response regulator transcription factor	N	N	N	N	N	N
WP_019659189.1	response regulator	N	N	N	N	N	N
WP_019659193.1	DMT family transporter	N	N	N	N	N	N
WP_019659640.1	HigA family addiction module antitoxin	N	N	N	N	N	N
WP_019659950.1	MerR family transcriptional regulator	N	N	N	N	N	N
WP_019660148.1	response regulator transcription factor	N	N	N	N	N	N
WP_019660241.1	RidA family protein	N	N	N	N	N	N
WP_019660307.1	winged helix-turn-helix transcriptional regulator	N	N	N	N	N	N

WP_019660932.1	class I SAM-dependent methyltransferase	N	N	N	N	N	N
WP_019661429.1	Cu(I)-responsive transcriptional regulator	N	N	N	N	N	N
WP_021202181.1	metalloregulator ArsR/SmtB family transcription factor	N	N	N	N	N	N
WP_021202184.1	protein-tyrosine-phosphatase	N	N	N	N	N	N
WP_021202287.1	hydrolase	N	N	N	N	N	N
WP_021202308.1	cyclase family protein	N	N	N	N	N	N
WP_021202430.1	TetR family transcriptional regulator	N	N	N	N	N	N
WP_021202431.1	nuclear transport factor 2 family protein	N	N	N	N	N	N
WP_021202492.1	aminotransferase class III-fold pyridoxal phosphate-dependent enzyme	N	N	N	N	N	N
WP_021202495.1	EamA family transporter	N	N	N	N	N	N
WP_021203081.1	alpha/beta hydrolase	N	N	N	N	N	N
WP_021203382.1	winged helix-turn-helix domain-containing protein	N	N	N	N	N	N
WP_021203522.1	DNA polymerase III subunit epsilon	N	N	N	N	N	N

WP_021203687.1	HigA family addiction module antitoxin	N	N	N	N	N	N
WP_021203900.1	Gfo/Idh/MocA family oxidoreductase	N	N	N	N	N	N
WP_021204101.1	VOC family protein	N	N	N	N	N	N
WP_021204238.1	diacylglycerol kinase	N	N	N	N	N	N
WP_021204259.1	tetratricopeptide repeat protein	N	N	N	N	N	N
WP_021204273.1	VOC family protein	N	N	N	N	N	N
WP_021204330.1	UDP-forming cellulose synthase catalytic subunit	N	N	N	N	N	N
WP_026347179.1	low molecular weight protein-tyrosine-phosphatase	N	N	N	N	N	N
WP_031269378.1	methylated-DNA--[protein]-cysteine S-methyltransferase [Stenotrophomonas maltophilia group sp. msm1]	N	N	N	N	N	N
WP_031270539.1	selenocysteine-specific translation elongation factor	N	N	N	N	N	N
WP_049396154.1	formate dehydrogenase subunit gamma	N	N	N	N	N	N
WP_058979789.1	putative addiction module antidote protein	N	N	N	N	N	N
WP_058982857.	sensory rhodopsin transducer	N	N	N	N	N	N

1							
WP_103283119.1	tautomerase family protein	N	N	N	N	N	N
WP_111096227.1	NarK family nitrate/nitrite MFS transporter	N	N	N	N	N	N
WP_111097688.1	type II toxin-antitoxin system RelE/ParE family toxin	N	N	N	N	N	N
WP_111098301.1	SDR family oxidoreductase	N	N	N	N	N	N
WP_111098529.1	winged helix-turn-helix domain-containing protein	N	N	N	N	N	N
WP_111098566.1	DUF4287 domain-containing protein	N	N	N	N	N	N
WP_111102396.1	biotin/lipoyl-binding protein	N	N	N	N	N	N
WP_111102601.1	AzlD family protein	N	N	N	N	N	N
WP_134729068.1	LysR family transcriptional regulator [Stenotrophomonas maltophilia group sp. msm1]	N	N	N	N	N	N
WP_134729194.1	hypothetical protein	N	N	N	N	N	N
WP_134730024.1	CmlA/FloR family chloramphenicol efflux MFS transporter	N	N	N	N	N	N
WP_134730337.1	GyrI-like domain-containing protein	N	N	N	N	N	N

WP_134731702.1	BCCT family transporter	N	N	N	N	N	N
WP_134735365.1	SRPBCC domain-containing protein	N	N	N	N	N	N
WP_194430608.1	DoxX family protein	N	N	N	N	N	N
WP_197596157.1	YegP family protein	N	N	N	N	N	N
WP_197605150.1	Clp protease ClpP	N	N	NZ_CP104323.1-3, NZ_CP104287.1-1, NZ_CP104323.1-2	100	N	N
WP_207050033.1	hypothetical protein	N	N	N	N	N	N
WP_227844649.1	SDR family oxidoreductase	N	N	N	N	N	N
WP_227853528.1	SMR family transporter	N	N	N	N	N	N
WP_227853601.1	DUF1064 domain-containing protein	N	N	N	N	N	N
WP_227853617.1	Rid family hydrolase	N	N	N	N	N	N
WP_227853635.1	DUF2239 family protein	N	N	N	N	N	N
WP_227853759.1	cytochrome o ubiquinol oxidase subunit III	N	N	N	N	N	N
WP_227853863.	ABC transporter ATP-binding protein	N	N	N	N	N	N

1							
WP_227853908.1	alpha/beta hydrolase	N	N	N	N	N	N
WP_227854034.1	L-seryl-tRNA(Sec) selenium transferase	N	N	N	N	N	N
WP_227854035.1	selenide, water dikinase SelD	N	N	N	N	N	N
WP_227854191.1	TIGR03885 family FMN-dependent LLM class oxidoreductase	N	N	N	N	N	N
WP_227854194.1	TonB-dependent siderophore receptor	N	N	N	N	N	N
WP_227854204.1	TIGR03571 family LLM class oxidoreductase	N	N	N	N	N	N
WP_227854221.1	VOC family protein	N	N	N	N	N	N
WP_227854246.1	aldehyde oxidoreductase molybdenum-binding subunit PaoC	N	N	N	N	N	N
WP_227854279.1	hydrolase	N	N	N	N	N	N
WP_227854310.1	DEAD/DEAH box helicase family protein	N	N	N	N	N	N
WP_227854412.1	protein deglycase HchA	N	N	N	N	N	N
WP_227854433.1	type II toxin-antitoxin system RelE/ParE family toxin	N	N	N	N	N	N
WP_258009861.	NAD-binding protein	N	N	N	N	N	N

	1							
	WP_275942789.1	helix-turn-helix transcriptional regulator	N	N	N	N	N	N
	WP_005408332.1	GPW/gp25 family protein	N	N	N	N	N	N
	WP_005408904.1	nuclear transport factor 2 family protein	N	N	N	N	N	N
	WP_043033528.1	nucleoside triphosphatase NudI	N	N	N	N	N	N
	WP_043034950.1	McrC family protein	N	N	N	N	N	N
	WP_043034954.1	type I restriction-modification system endonuclease	N	N	N	N	N	N
NZ_CP104288.1	WP_154329194.1	AIPR family protein	N	N	N	N	N	N
	WP_154329196.1	Z1 domain-containing protein	N	N	N	N	N	N
	WP_227859976.1	SDR family oxidoreductase	N	N	N	N	N	N
	WP_227860027.1	cytochrome c	N	N	N	N	N	N
	WP_227860028.1	GMC family oxidoreductase	N	N	N	N	N	N
	WP_227860029.1	gluconate 2-dehydrogenase subunit 3 family protein	N	N	N	N	N	N
	WP_227860033.	ATP-binding cassette domain-containing	N	N	N	N	N	N

	1	protein						
	WP_227860035.<br>1	iron chelate uptake ABC transporter family permease subunit	N	N	N	N	N	N
	WP_227860039.<br>1	fumarylacetoacetate hydrolase family protein	N	N	N	N	N	N
	WP_227860042.<br>1	transcriptional regulator GcvA	N	N	N	N	N	N
	WP_227860043.<br>1	carboxymuconolactone decarboxylase family protein	N	N	N	N	N	N
	WP_227860044.<br>1	cupin domain-containing protein	N	N	N	N	N	N
	WP_227860049.<br>1	metallohydrolase	N	N	N	N	N	N
	WP_227860296.<br>1	ATP-binding protein	N	N	N	N	N	N
	WP_229298737.<br>1	very short patch repair endonuclease	N	N	N	N	N	N
	WP_005413341.<br>1	replication-associated recombination protein A	N	N	N	N	N	N
	WP_017354485.<br>1	excinuclease ABC subunit UvrB	N	N	N	N	N	N
NZ_CP104289.1	WP_017355006.<br>1	AAA family ATPase	N	N	N	N	N	N
	WP_026070508.<br>1	NAD(P)/FAD-dependent oxidoreductase	N	N	N	N	N	N
	WP_026070509.	DUF3422 family protein	N	N	N	N	N	N

1							
WP_026070510.1	sigma-54 dependent transcriptional regulator	N	N	N	N	N	N
WP_026070607.1	DUF6602 domain-containing protein	N	N	N	N	N	N
WP_026070608.1	hypothetical protein	N	N	N	N	N	N
WP_026070609.1	CBASS oligonucleotide cyclase	N	N	N	N	N	N
WP_037590709.1	NAD(P)/FAD-dependent oxidoreductase	N	N	N	N	N	N
WP_053501247.1	helix-turn-helix transcriptional regulator	N	N	N	N	N	N
WP_065199099.1	ThiF family adenylyltransferase	N	N	N	N	N	N
WP_065199100.1	multiubiquitin domain-containing protein	N	N	N	N	N	N
WP_065199104.1	helix-turn-helix transcriptional regulator	N	N	N	N	N	N
WP_088497116.1	Gp49 family protein	N	N	N	N	N	N
WP_100438305.1	Eco29kI family restriction endonuclease	N	N	N	N	N	N
WP_102947207.1	IS5 family transposase	N	N	N	N	N	N
WP_102947323.	antibiotic biosynthesis monooxygenase	N	N	N	N	N	N

	1							
	WP_111007945.1	UvrD-helicase domain-containing protein	N	N	N	N	N	N
	WP_227832955.1	bestrophin family protein	N	N	N	N	N	N
	WP_227836612.1	DEAD/DEAH box helicase family protein	N	N	N	N	N	N
	WP_227836614.1	class I SAM-dependent DNA methyltransferase	N	N	N	N	N	N
	WP_227837157.1	type II toxin-antitoxin system RelE/ParE family toxin	N	N	N	N	N	N
	WP_227837432.1	DNA adenine methylase	N	N	N	N	N	N
	WP_227837798.1	hypothetical protein	N	N	N	N	N	N
	WP_227838122.1	DNA cytosine methyltransferase	N	N	N	N	N	N
	WP_227838186.1	AAA domain-containing protein	N	N	N	N	N	N
	WP_227838310.1	DUF6708 domain-containing protein	N	N	N	N	N	N
	WP_111189775.1	TIR domain-containing protein	N	N	N	N	N	N
NZ_CP104290.1	WP_111189776.1	hypothetical protein	N	N	N	N	N	N
	WP_227415721.	site-specific DNA-methyltransferase	N	N	N	N	N	N

	1							
	WP_227415921.1	hypothetical protein	N	N	N	N	N	N
	WP_227416002.1	cupin domain-containing protein	N	N	N	N	N	N
	WP_227416005.1	carboxymuconolactone decarboxylase family protein	N	N	N	N	N	N
	WP_227416010.1	fumarylacetoacetate hydrolase family protein	N	N	N	N	N	N
	WP_227416014.1	iron chelate uptake ABC transporter family permease subunit	N	N	N	N	N	N
	WP_227416021.1	gluconate 2-dehydrogenase subunit 3 family protein	N	N	N	N	N	N
	WP_227416022.1	GMC family oxidoreductase	N	N	N	N	N	N
	WP_227416023.1	c-type cytochrome	N	N	N	N	N	N
	WP_261280074.1	recombinase family protein	N	N	N	N	N	N
	WP_261280091.1	ATP-binding protein	N	N	N	N	N	N
	WP_000063280.1	major capsid protein	N	N	N	N	N	N
NZ_CP104292.1	WP_000065374.1	DUF2528 family protein	N	N	N	N	N	N
	WP_000092275.	prophage endopeptidase RzpD	N	N	N	N	N	N

1							
WP_000100844.1	phage recombination protein Bet	N	N	N	N	N	N
WP_000104864.1	LexA family transcriptional regulator	N	N	N	N	N	N
WP_000113775.1	NinE family protein	N	N	N	N	N	N
WP_000123343.1	S49 family peptidase	N	N	N	N	N	N
WP_000145933.1	protein ren	N	N	N	N	N	N
WP_000149542.1	DUF1317 domain-containing protein	N	N	N	N	N	N
WP_000158919.1	DNA-packaging protein FI	N	N	N	N	N	N
WP_000185505.1	replication protein	N	N	N	N	N	N
WP_000186853.1	YqaJ viral recombinase family protein	N	N	N	N	N	N
WP_000194780.1	C40 family peptidase	N	N	N	N	N	N
WP_000251069.1	lambda phage CII family protein	N	N	N	N	N	N
WP_000453580.1	DNA-packaging protein	N	N	N	N	N	N
WP_000459457.	phage tail assembly protein T	N	N	N	N	N	N

1							
WP_000479153.1	phage minor tail protein G	N	N	N	N	N	N
WP_000480968.1	aminoglycoside O-phosphotransferase APH(6)-Id	N	N	N	N	N	N
WP_000515495.1	host specificity protein J	N	N	N	N	N	N
WP_000533640.1	tyrosine-type recombinase/integrase	N	N	N	N	N	N
WP_000611491.1	phosphoadenosine phosphosulfate reductase family protein	N	N	N	N	N	N
WP_000683105.1	phage minor tail U family protein	N	N	N	N	N	N
WP_000736903.1	recombination protein NinB	N	N	N	N	N	N
WP_000738492.1	serum resistance lipoprotein Bor	N	N	N	N	N	N
WP_000752979.1	head-tail joining protein	N	N	N	N	N	N
WP_000763367.1	TraR/DksA family transcriptional regulator	N	N	N	N	N	N
WP_000788910.1	replication protein P	N	N	N	N	N	N
WP_000840207.1	phage tail tape measure protein	N	N	N	N	N	N
WP_000847379.	phage tail protein	N	N	N	N	N	N

1							
WP_000975070.1	phage tail protein	N	N	N	N	N	N
WP_000995451.1	host-nuclease inhibitor Gam family protein	N	N	N	N	N	N
WP_001027292.1	phage terminase large subunit family protein	N	N	N	N	N	N
WP_001043260.1	sulfonamide-resistant dihydropteroate synthase Sul2	N	N	N	N	N	N
WP_001082319.1	aminoglycoside O-phosphotransferase APH(3")-Ib	N	N	N	N	N	N
WP_001120888.1	IS91-like element ISVsa3 family transposase	N	N	N	N	N	N
WP_001152639.1	phage minor tail protein L	N	N	N	N	N	N
WP_001255015.1	LysR family transcriptional regulator	N	N	N	N	N	N
WP_001271136.1	serine/threonine protein phosphatase	N	N	N	N	N	N
WP_001297109.1	head decoration protein	N	N	N	N	N	N
WP_001349920.1	phage tail protein	N	N	N	N	N	N
WP_001359455.1	phage portal protein	N	N	N	N	N	N
WP_001407644.	tail assembly protein	N	N	N	N	N	N

1							
WP_001447541.1	DUF3363 domain-containing protein	N	N	N	N	N	N
WP_009873364.1	tetracycline resistance transcriptional repressor TetR(C)	N	N	N	N	N	N
WP_009873365.1	IS200/IS605 family transposase	N	N	N	N	N	N
WP_015979258.1	tail fiber assembly protein	N	N	N	N	N	N
WP_015979259.1	AAA family ATPase	N	N	N	N	N	N
WP_015979261.1	recombination protein NinG	N	N	N	N	N	N
WP_026347113.1	SIR2 family protein	N	N	N	N	N	N
WP_104938957.1	CopG family transcriptional regulator	N	N	N	N	N	N
WP_111111829.1	SMR family transporter	N	N	N	N	N	N
WP_162109697.1	AlpA family phage regulatory protein	N	N	N	N	N	N
WP_216653153.1	replication initiator protein A	N	N	N	N	N	N
WP_227843259.1	hypothetical protein	N	N	N	N	N	N
WP_227843811.	hypothetical protein	N	N	N	N	N	N

	1						
	WP_227843841.1	LuxR family transcriptional regulator	N	N	N	N	N	N
	WP_261263886.1	helix-turn-helix domain-containing protein	N	N	N	N	N	N
	WP_261263951.1	hypothetical protein	N	N	N	N	N	N
	WP_005409440.1	Cu(I)-responsive transcriptional regulator	N	N	N	N	N	N
	WP_005409726.1	ABC transporter permease	N	N	N	N	N	N
	WP_005411609.1	acyl carrier protein	N	N	N	N	N	N
	WP_005413654.1	Lrp/AsnC family transcriptional regulator	N	N	N	N	N	N
NZ_CP104323.1	WP_005420598.1	putative addiction module antidote protein	N	N	N	N	N	N
	WP_006399830.1	cytochrome o ubiquinol oxidase subunit IV	N	N	N	N	N	N
	WP_010485668.1	LysR family transcriptional regulator	N	N	N	N	N	N
	WP_012479411.1	helix-turn-helix transcriptional regulator	N	N	N	N	N	N
	WP_014037677.1	helix-turn-helix domain-containing protein	N	N	N	N	N	N
	WP_014645786.	TetR family transcriptional regulator	N	N	N	N	N	N

1							
WP_014645788.1	MFS transporter	N	N	N	N	N	N
WP_014645816.1	class I SAM-dependent methyltransferase	N	N	N	N	N	N
WP_014645829.1	winged helix-turn-helix domain-containing protein	N	N	N	N	N	N
WP_014645907.1	UDP-N-acetylglucosamine 2-epimerase (non-hydrolyzing)	N	N	N	N	N	N
WP_014646094.1	flavin reductase family protein	N	N	N	N	N	N
WP_014646324.1	alpha/beta hydrolase	N	N	N	N	N	N
WP_014646430.1	EamA family transporter	N	N	N	N	N	N
WP_014646433.1	response regulator	N	N	N	N	N	N
WP_014646778.1	SRPBCC family protein	N	N	N	N	N	N
WP_014646978.1	response regulator	N	N	N	N	N	N
WP_014647012.1	BCCT family transporter	N	N	N	N	N	N
WP_014647135.1	response regulator	N	N	N	N	N	N
WP_014647218.	ABC transporter ATP-binding protein	N	N	N	N	N	N

1							
WP_014647247.1	hypothetical protein	N	N	N	N	N	N
WP_014647273.1	sensory rhodopsin transducer	N	N	N	N	N	N
WP_014647350.1	LysR family transcriptional regulator	N	N	N	N	N	N
WP_014647351.1	pirin family protein	N	N	N	N	N	N
WP_014647352.1	hydrolase	N	N	N	N	N	N
WP_014647378.1	AzlD family protein	N	N	N	N	N	N
WP_014647407.1	response regulator transcription factor	N	N	N	N	N	N
WP_014647409.1	diacylglycerol kinase	N	N	N	N	N	N
WP_014647429.1	tetratricopeptide repeat protein	N	N	N	N	N	N
WP_014647448.1	MgtC/SapB family protein	N	N	N	N	N	N
WP_014647585.1	NADH:flavin oxidoreductase/NADH oxidase family protein	N	N	N	N	N	N
WP_014647822.1	SDR family oxidoreductase	N	N	N	N	N	N
WP_014647841.	SMR family transporter	N	N	N	N	N	N

1							
WP_014647846.1	hypothetical protein	N	N	N	N	N	N
WP_014648178.1	response regulator transcription factor	N	N	N	N	N	N
WP_014648247.1	HigA family addiction module antitoxin	N	N	N	N	N	N
WP_014648460.1	Gfo/Idh/MocA family oxidoreductase	N	N	N	N	N	N
WP_014648590.1	response regulator transcription factor	N	N	N	N	N	N
WP_014648639.1	hypothetical protein	N	N	N	N	N	N
WP_014648729.1	cytochrome o ubiquinol oxidase subunit III	N	N	N	N	N	N
WP_014648871.1	DNA polymerase III subunit epsilon	N	N	N	N	N	N
WP_032957727.1	alpha/beta hydrolase	N	N	N	N	N	N
WP_032958324.1	DoxX family protein	N	N	N	N	N	N
WP_032958352.1	VOC family protein	N	N	N	N	N	N
WP_032958520.1	glutathione S-transferase family protein	N	N	N	N	N	N
WP_032959997.	metalloregulator ArsR/SmtB family	N	N	N	N	N	N

1	transcription factor						
WP_032962018.1	LysR family transcriptional regulator	N	N	N	N	N	N
WP_032962148.1	type II toxin-antitoxin system RelE/ParE family toxin	N	N	N	N	N	N
WP_032962610.1	winged helix-turn-helix domain-containing protein	N	N	N	N	N	N
WP_041863210.1	response regulator	N	N	N	N	N	N
WP_046431075.1	VOC family protein	N	N	N	N	N	N
WP_046431094.1	MerR family transcriptional regulator	N	N	N	N	N	N
WP_046431272.1	putative quinol monooxygenase	N	N	N	N	N	N
WP_046431502.1	zinc-dependent alcohol dehydrogenase family protein	N	N	N	N	N	N
WP_049404958.1	LysR family transcriptional regulator	N	N	N	N	N	N
WP_049405454.1	tail assembly protein	NZ_CP028899.1-1, NZ_CP104323.1-1	100	N	N	N	N
WP_049406437.1	SDR family oxidoreductase	N	N	N	N	N	N
WP_049408320.1	methylated-DNA--[protein]-cysteine S-methyltransferase	N	N	N	N	N	N
WP_049439232.	MFS transporter	N	N	N	N	N	N

1							
WP_049439571.1	EamA family transporter	N	N	N	N	N	N
WP_049439746.1	TetR family transcriptional regulator	N	N	N	N	N	N
WP_049439912.1	response regulator	N	N	N	N	N	N
WP_049440661.1	hypothetical protein	N	N	N	N	N	N
WP_049441504.1	DMT family transporter	N	N	N	N	N	N
WP_049441669.1	helix-turn-helix domain-containing protein	N	N	N	N	N	N
WP_049442064.1	hypothetical protein	N	N	N	N	N	N
WP_053494450.1	DUF2938 domain-containing protein	N	N	N	N	N	N
WP_074726395.1	tautomerase family protein	N	N	N	N	N	N
WP_088475915.1	LysR family transcriptional regulator	N	N	N	N	N	N
WP_100448254.1	Rid family hydrolase	N	N	N	N	N	N
WP_111205971.1	ATP-binding protein	N	N	N	N	N	N
WP_126411319.	selenocysteine-specific translation	N	N	N	N	N	N

1	elongation factor						
WP_126412339.1	DUF3224 domain-containing protein	N	N	N	N	N	N
WP_134808466.1	MFS transporter	N	N	N	N	N	N
WP_134809166.1	VOC family protein	N	N	N	N	N	N
WP_141098655.1	ABC transporter permease	N	N	N	N	N	N
WP_142234422.1	GyrI-like domain-containing protein	N	N	N	N	N	N
WP_142235221.1	L-seryl-tRNA(Sec) selenium transferase	N	N	N	N	N	N
WP_164167130.1	ABC transporter ATP-binding protein	N	N	N	N	N	N
WP_169411882.1	alpha/beta hydrolase	N	N	N	N	N	N
WP_197575533.1	HigA family addiction module antitoxin	N	N	N	N	N	N
WP_197605150.1	Clp protease ClpP	N	N	NZ_CP104323.1-3, NZ_CP104287.1-1, NZ_CP104323.1-2	100	N	N
WP_197605687.1	VOC family protein	N	N	N	N	N	N
WP_197606300.1	VOC family protein	N	N	N	N	N	N

WP_227863647.1	aminotransferase class III-fold pyridoxal phosphate-dependent enzyme	N	N	N	N	N	N
WP_227863703.1	ubiquinol oxidase subunit II	N	N	N	N	N	N
WP_227863724.1	TonB-dependent siderophore receptor	N	N	N	N	N	N
WP_227863726.1	ABC transporter six-transmembrane domain-containing protein	N	N	N	N	N	N
WP_227863727.1	ATP-binding protein	N	N	N	N	N	N
WP_227863729.1	TIGR03885 family FMN-dependent LLM class oxidoreductase	N	N	N	N	N	N
WP_227863779.1	SDR family oxidoreductase	N	N	N	N	N	N
WP_227863780.1	GlxA family transcriptional regulator	N	N	N	N	N	N
WP_227863782.1	MFS transporter	N	N	N	N	N	N
WP_227863783.1	cyclophilin-like fold protein	N	N	N	N	N	N
WP_227863784.1	carboxymuconolactone decarboxylase family protein	N	N	N	N	N	N
WP_227863787.1	aldehyde oxidoreductase molybdenum-binding subunit PaoC	N	N	N	N	N	N
WP_227863819.1	carboxymuconolactone decarboxylase family protein	N	N	N	N	N	N

WP_227863872.1	type II toxin-antitoxin system RelE/ParE family toxin	N	N	N	N	N	N
WP_227863888.1	DUF2239 family protein	N	N	N	N	N	N
WP_227863902.1	MerR family transcriptional regulator	N	N	N	N	N	N
WP_227863904.1	FAD-dependent monooxygenase	N	N	N	N	N	N
WP_227863951.1	GIY-YIG nuclease family protein	N	N	N	N	N	N
WP_227863960.1	hypothetical protein	N	N	N	N	N	N
WP_227863963.1	SRPBCC domain-containing protein	N	N	N	N	N	N
WP_227864000.1	glycoside hydrolase family 104 protein	N	N	N	N	N	N
WP_227864004.1	DUF1064 domain-containing protein	N	N	N	N	N	N
WP_227864030.1	helix-turn-helix domain-containing protein	N	N	N	N	N	N
WP_227864031.1	aromatic alcohol reductase	N	N	N	N	N	N
WP_227864032.1	GNAT family N-acetyltransferase	N	N	N	N	N	N
WP_227864274.1	LysR substrate-binding domain-containing protein	N	N	N	N	N	N

WP_227864379.1	formate dehydrogenase subunit beta	N	N	N	N	N	N
WP_227864380.1	formate dehydrogenase subunit gamma	N	N	N	N	N	N
WP_227864381.1	selenide, water dikinase SelD	N	N	N	N	N	N
WP_227864422.1	transcriptional regulator BetI	N	N	N	N	N	N
WP_227864473.1	glycosyl transferase family protein	N	N	N	N	N	N
WP_227864501.1	metalloregulator ArsR/SmtB family transcription factor	N	N	N	N	N	N
WP_227864504.1	protein deglycase HchA	N	N	N	N	N	N
WP_227864513.1	Rid family hydrolase	N	N	N	N	N	N
WP_227864564.1	LysR family transcriptional regulator	N	N	N	N	N	N
WP_227864602.1	biotin/lipoyl-binding protein	N	N	N	N	N	N
WP_227864680.1	cyclase family protein	N	N	N	N	N	N
WP_227864681.1	TIGR03571 family LLM class oxidoreductase	N	N	N	N	N	N
WP_227864682.1	2,5-didehydrogluconate reductase DkgB	N	N	N	N	N	N

NZ_CP104324.1	WP_269194971.1	helix-turn-helix transcriptional regulator	N	N	N	N	N	N
	WP_005411473.1	cytochrome o ubiquinol oxidase subunit IV	N	N	N	N	N	N
	WP_010484073.1	type II toxin-antitoxin system RelE/ParE family toxin	N	N	N	N	N	N
	WP_017354485.1	excinuclease ABC subunit UvrB	N	N	N	N	N	N
	WP_017355006.1	AAA family ATPase	N	N	N	N	N	N
	WP_021202858.1	DUF1972 domain-containing protein	N	N	N	N	N	N
	WP_026070508.1	NAD(P)/FAD-dependent oxidoreductase	N	N	N	N	N	N
	WP_026070509.1	DUF3422 family protein	N	N	N	N	N	N
	WP_026070510.1	sigma-54 dependent transcriptional regulator	N	N	N	N	N	N
	WP_026070607.1	DUF6602 domain-containing protein	N	N	N	N	N	N
	WP_026070608.1	hypothetical protein	N	N	N	N	N	N
	WP_026070609.1	CBASS oligonucleotide cyclase	N	N	N	N	N	N
	WP_037590709.1	NAD(P)/FAD-dependent oxidoreductase	N	N	N	N	N	N

WP_053496918.1	AAA family ATPase	N	N	N	N	N	N
WP_057502224.1	GIY-YIG nuclease family protein	N	N	N	N	N	N
WP_102947207.1	IS5 family transposase	N	N	N	N	N	N
WP_102947323.1	antibiotic biosynthesis monooxygenase	N	N	N	N	N	N
WP_102947324.1	cyclic nucleotide-binding domain-containing thioredoxin-disulfide reductase	N	N	N	N	N	N
WP_111103805.1	acyl-protein synthetase	N	N	N	N	N	N
WP_164106453.1	group II intron reverse transcriptase/maturase	N	N	N	N	N	N
WP_227832955.1	bestrophin family protein	N	N	N	N	N	N
WP_227851967.1	DNA methyltransferase	N	N	N	N	N	N
WP_227851997.1	AAA family ATPase	N	N	N	N	N	N
WP_227852406.1	phage major capsid protein	N	N	NZ_CP104324.1-4	100	N	N
WP_227852407.1	Clp protease ClpP	N	N	NZ_CP104324.1-4	100	N	N
WP_227852408.	terminase TerL endonuclease subunit	N	N	NZ_CP104324.1-4	100	N	N

	1							
	WP_227852419.1	acyl carrier protein	N	N	N	N	N	N
	WP_227852570.1	outer membrane beta-barrel protein	N	N	N	N	N	N
	WP_227852699.1	metalloregulator ArsR/SmtB family transcription factor	N	N	N	N	N	N
	WP_227852774.1	phage head closure protein	N	N	N	N	N	N
	WP_227852777.1	DUF3168 domain-containing protein	N	N	N	N	N	N
	WP_227852781.1	phage tail assembly chaperone family protein, TAC	N	N	N	N	N	N
	WP_227852787.1	DUF1833 family protein	N	N	N	N	N	N
	WP_227852880.1	ATP-binding protein	NZ_CP088241.1-1	100	N	N	NZ_CP090418.1-4	100
	WP_227852882.1	replication-associated recombination protein A	N	N	N	N	N	N
	WP_260833009.1	YbhB/YbcL family Raf kinase inhibitor-like protein	N	N	N	N	N	N
	WP_260833015.1	Clp protease ClpP	NZ_CP104324.1-1	100	N	N	N	N
	WP_260833016.1	very short patch repair endonuclease	N	N	N	N	N	N
NZ_CP104863.1	WP_000043569.	hypothetical protein	N	N	N	N	N	N

1							
WP_000063629.1	ABC transporter permease	N	N	N	N	N	N
WP_000085131.1	ribbon-helix-helix protein, CopG family	N	N	N	N	N	N
WP_000147048.1	hypothetical protein	N	N	N	N	N	N
WP_000150010.1	TrbC/VirB2 family protein	N	N	N	N	N	N
WP_000405670.1	LysR family transcriptional regulator	N	N	N	N	N	N
WP_000480968.1	aminoglycoside O-phosphotransferase APH(6)-Id	N	N	N	N	NZ_CP104863.1-4	100
WP_000539741.1	multidrug efflux SMR transporter	N	N	N	N	N	N
WP_000679427.1	quaternary ammonium compound efflux SMR transporter QacE delta 1	N	N	N	N	N	N
WP_000717310.1	EexN family lipoprotein	N	N	N	N	N	N
WP_001000746.1	P-type conjugative transfer protein TrbL	N	N	N	N	N	N
WP_001062689.1	site-specific integrase	N	N	N	N	N	N
WP_001067855.1	IS6-like element IS26 family transposase	N	N	N	N	NZ_CP104863.1-4	100
WP_001082319.	aminoglycoside O-phosphotransferase	N	N	N	N	N	N

1	APH(3")-Ib						
WP_001138082.1	Tn3 family transposase	N	N	N	N	N	N
WP_001247107.1	helix-turn-helix domain-containing protein	N	N	N	N	N	N
WP_001273857.1	DUF736 domain-containing protein	N	N	N	N	N	N
WP_003092277.1	P-type conjugative transfer protein TrbJ	N	N	N	N	N	N
WP_003092280.1	VirB3 family type IV secretion system protein	N	N	N	N	N	N
WP_003092304.1	S26 family signal peptidase	N	N	N	N	N	N
WP_003092318.1	DUF2285 domain-containing protein	N	N	N	N	N	N
WP_003092330.1	DNA repair protein RadC	N	N	N	N	N	N
WP_003830719.1	quaternary ammonium compound efflux SMR transporter QacG2	N	N	N	N	N	N
WP_005408112.1	LysR family transcriptional regulator	N	N	N	N	N	N
WP_005408332.1	GPW/gp25 family protein	N	N	N	N	N	N
WP_005408917.1	SRPBCC family protein	N	N	N	N	N	N
WP_005411449.	arsenate reductase (glutaredoxin)	N	N	N	N	N	N

1							
WP_005411451.1	arsenic transporter	N	N	N	N	N	N
WP_005411473.1	cytochrome o ubiquinol oxidase subunit IV	N	N	N	N	N	N
WP_006379425.1	helix-turn-helix transcriptional regulator	N	N	N	N	N	N
WP_009459591.1	helix-turn-helix transcriptional regulator	N	N	N	N	N	N
WP_009459672.1	TIGR03758 family integrating conjugative element protein	N	N	N	N	N	N
WP_009459699.1	DUF3085 domain-containing protein	N	N	N	N	N	N
WP_009459729.1	AlpA family transcriptional regulator	N	N	N	N	N	N
WP_014603538.1	hypothetical protein	N	N	N	N	N	N
WP_016487838.1	DUF2958 domain-containing protein	N	N	N	N	N	N
WP_017512401.1	hypothetical protein	N	N	N	N	N	N
WP_017512403.1	hypothetical protein	N	N	N	N	N	N
WP_017514119.1	TIGR03757 family integrating conjugative element protein	N	N	N	N	N	N
WP_017515583.	TIGR03752 family integrating	N	N	N	N	N	N

1	conjugative element protein						
WP_017515759.1	integrating conjugative element protein	N	N	N	N	N	N
WP_017516006.1	hypothetical protein	N	N	N	N	N	N
WP_019726333.1	hypothetical protein	N	N	N	N	N	N
WP_020924940.1	Dyp-type peroxidase	N	N	N	N	N	N
WP_022580212.1	hypothetical protein	N	N	N	N	N	N
WP_023093368.1	LysR family transcriptional regulator	N	N	N	N	N	N
WP_023093371.1	ATP-binding cassette domain-containing protein	N	N	N	N	N	N
WP_023093372.1	DUF2840 domain-containing protein	N	N	N	N	N	N
WP_023093474.1	DUF2857 domain-containing protein	N	N	N	N	N	N
WP_023103875.1	integrase arm-type DNA-binding domain-containing protein	N	N	N	N	N	N
WP_023103885.1	VOC family protein	N	N	N	N	N	N
WP_023103896.1	integrating conjugative element protein	N	N	N	N	N	N
WP_023103898.	DNA repair protein RadC	N	N	N	N	N	N

1							
WP_023103901.1	TIGR03751 family conjugal transfer lipoprotein	N	N	N	N	N	N
WP_023103903.1	TIGR03749 family integrating conjugative element protein	N	N	N	N	N	N
WP_023103905.1	TIGR03750 family conjugal transfer protein	N	N	N	N	N	N
WP_023103906.1	TIGR03745 family integrating conjugative element membrane protein	N	N	N	N	N	N
WP_023103908.1	TIGR03747 family integrating conjugative element membrane protein	N	N	N	N	N	N
WP_023103919.1	hypothetical protein	N	N	N	N	N	N
WP_023103921.1	hypothetical protein	N	N	N	N	N	N
WP_023103923.1	DUF3275 family protein	N	N	N	N	N	N
WP_023103924.1	DUF3577 domain-containing protein	N	N	N	N	N	N
WP_023103925.1	hypothetical protein	N	N	N	N	N	N
WP_023464298.1	IS6-like element IS6100 family transposase	N	N	N	N	NZ_CP104863.1-4	100
WP_023464791.1	DUF3422 family protein	N	N	N	N	N	N
WP_023835768.	DUF488 domain-containing protein	N	N	N	N	N	N

1							
WP_024086516.1	IS5-like element ISPa26 family transposase	N	N	N	N	N	N
WP_024956907.1	LysR substrate-binding domain-containing protein	N	N	N	N	N	N
WP_028690943.1	excinuclease ABC subunit UvrB	N	N	N	N	N	N
WP_028690947.1	NAD(P)/FAD-dependent oxidoreductase	N	N	N	N	N	N
WP_028690948.1	replication-associated recombination protein A	N	N	N	N	N	N
WP_028690949.1	Na+/H+ antiporter NhaA	N	N	N	N	N	N
WP_028690951.1	MobH family relaxase	N	N	N	N	N	N
WP_028690954.1	DsbA family protein	N	N	N	N	N	N
WP_028690955.1	transglycosylase SLT domain-containing protein	N	N	N	N	N	N
WP_028690956.1	PilL N-terminal domain-containing protein	N	N	N	N	N	N
WP_028690958.1	helicase-related protein	N	N	N	N	N	N
WP_028690960.1	DNA topoisomerase III	N	N	N	N	N	N
WP_028690961.	single-stranded DNA-binding protein	N	N	N	N	N	N

1							
WP_028690962.1	DUF3158 family protein	N	N	N	N	N	N
WP_028690963.1	TIGR03761 family integrating conjugative element protein	N	N	N	N	N	N
WP_028690964.1	STY4528 family pathogenicity island replication protein	N	N	N	N	N	N
WP_028690965.1	ParB family protein	N	N	N	N	N	N
WP_028690966.1	ParA family protein	N	N	N	N	N	N
WP_028698647.1	conjugal transfer protein TraG N-terminal domain-containing protein	N	N	N	N	N	N
WP_042862846.1	TIGR03759 family integrating conjugative element protein	N	N	N	N	N	N
WP_043033828.1	acyl-CoA dehydrogenase family protein	N	N	N	N	N	N
WP_099484974.1	metalloregulator ArsR/SmtB family transcription factor	N	N	N	N	N	N
WP_114619015.1	arsenate reductase ArsC	N	N	N	N	N	N
WP_197596600.1	nucleotide sugar dehydrogenase	N	N	N	N	N	N
WP_227859975.1	GlxA family transcriptional regulator	N	N	N	N	N	N
WP_228756277.	alcohol dehydrogenase catalytic	N	N	N	N	N	N

1	domain-containing protein						
WP_240791806.1	nuclear transport factor 2 family protein	N	N	N	N	N	N
WP_254912518.1	DUF6094 domain-containing protein	N	N	N	N	N	N
WP_261727878.1	TIGR03746 family integrating conjugative element protein	N	N	N	N	N	N
WP_261727937.1	class I SAM-dependent DNA methyltransferase	N	N	N	N	N	N
WP_261727938.1	DEAD/DEAH box helicase family protein	N	N	N	N	N	N
WP_261728061.1	GIY-YIG nuclease family protein	N	N	N	N	N	N
WP_261728063.1	DNA methyltransferase	N	N	N	N	N	N
WP_261728091.1	SDR family oxidoreductase	N	N	N	N	N	N
WP_261728104.1	tyrosine-type recombinase/integrase	N	N	N	N	N	N
WP_261728169.1	HlyD family efflux transporter periplasmic adaptor subunit	N	N	N	N	N	N
WP_261728178.1	phosphoadenosine phosphosulfate reductase family protein	N	N	N	N	N	N
WP_261728201.1	DEAD/DEAH box helicase family protein	N	N	N	N	N	N
WP_261728213.	AadA family aminoglycoside	N	N	N	N	N	N

	1	3''-O-nucleotidyltransferase						
	WP_261728217.1	TrbI/VirB10 family protein	N	N	N	N	N	N
	WP_261728259.1	metalloregulator ArsR/SmtB family transcription factor	N	N	N	N	N	N
	WP_261728309.1	DNA adenine methylase	NZ_CP104863.1-1		N	N	N	N
	WP_261728478.1	SDR family oxidoreductase	N	N	N	N	N	N
	WP_261728491.1	transposase	N	N	N	N	N	N
	WP_315968189.1	helix-turn-helix domain-containing protein	N	N	N	N	N	N
NZ_CP106759.1	WP_005414746.1	HlyD family efflux transporter periplasmic adaptor subunit	N	N	N	N	N	N
	WP_053515824.1	FadR/GntR family transcriptional regulator	N	N	N	N	N	N
	WP_053515919.1	efflux RND transporter periplasmic adaptor subunit	N	N	N	N	N	N
	WP_053515927.1	TetR/AcrR family transcriptional regulator	N	N	N	N	N	N
	WP_053518143.1	NUDIX domain-containing protein	N	N	N	N	N	N
	WP_065174462.1	nuclear transport factor 2 family protein	N	N	N	N	N	N
	WP_065174626.	VWA domain-containing protein	N	N	N	N	N	N

1							
WP_065181626.1	helix-turn-helix domain-containing GNAT family N-acetyltransferase	N	N	N	N	N	N
WP_111185940.1	type I secretion system permease/ATPase	N	N	N	N	N	N
WP_111187015.1	helix-turn-helix transcriptional regulator	N	N	N	N	N	N
WP_197600006.1	glycoside hydrolase family 43 protein	N	N	N	N	N	N
WP_227255387.1	nucleotide pyrophosphohydrolase	N	N	N	N	N	N
WP_239519805.1	TolC family protein	N	N	N	N	N	N
WP_253118489.1	MDR family oxidoreductase	N	N	N	N	N	N
WP_253118553.1	LysR family transcriptional regulator	N	N	N	N	N	N
WP_263174235.1	TonB-dependent siderophore receptor	N	N	N	N	N	N
WP_263174238.1	hypothetical protein	N	N	N	N	N	N
WP_263174239.1	hypothetical protein	N	N	N	N	N	N
WP_263174254.1	LysR substrate-binding domain-containing protein	N	N	N	N	N	N
WP_263174303.	SMP-30/gluconolactonase/LRE family	N	N	N	N	N	N

1	protein						
WP_263174505.1	MFS transporter	N	N	N	N	N	N
WP_263174615.1	Ig-like domain-containing protein	N	N	N	N	N	N
WP_263174781.1	DUF1833 domain-containing protein	N	N	N	N	N	N
WP_263174790.1	phage tail assembly chaperone family protein, TAC	N	N	N	N	N	N
WP_263174795.1	DUF3168 domain-containing protein	N	N	N	N	N	N
WP_263174800.1	phage head closure protein	N	N	N	N	N	N
WP_263174806.1	head-tail connector protein	N	N	N	N	N	N
WP_263174811.1	phage major capsid protein	NZ_CP106759.1-2	100	N	N	N	N
WP_263174813.1	HK97 family phage prohead protease	NZ_CP106759.1-2	100	N	N	N	N
WP_263174815.1	phage portal protein	NZ_CP106759.1-2	100	N	N	N	N
WP_263174818.1	terminase TerL endonuclease subunit	N	N	N	N	N	N
WP_263174827.1	glycoside hydrolase family 19 protein	NZ_CP106759.1-2	100	N	N	N	N
WP_263174856.	hypothetical protein	N	N	N	N	N	N

	1							
	WP_263177152.1	DUF262 domain-containing protein	N	N	N	N	N	N
	WP_263177156.1	NgoMIV family type II restriction endonuclease	N	N	N	N	N	N
	WP_263177158.1	DNA cytosine methyltransferase	N	N	N	N	N	N
	WP_263177843.1	DUF4276 family protein	N	N	N	N	N	N
	WP_263177844.1	AAA family ATPase	N	N	N	N	N	N
	WP_263178026.1	DapH/DapD/GlmU-related protein	N	N	N	N	N	N
	WP_263178030.1	phenylphosphate carboxylase subunit delta	N	N	N	N	N	N
	WP_263178056.1	hypothetical protein	N	N	N	N	N	N
	WP_263178282.1	CGNR zinc finger domain-containing protein	N	N	N	N	N	N
NZ_LR134301.1	WP_057499537.1	YqaJ viral recombinase family protein	N	N	NZ_LR134301.1-3	100	N	N
	WP_057499538.1	recombinase RecT	N	N	NZ_LR134301.1-3	100	N	N
	WP_057499574.1	lysozyme	N	N	N	N	N	N
	WP_057499752.	flagellar type III secretion system pore	N	N	N	N	N	N

	1	protein FliP						
	WP_057499769.1	flagellar biosynthesis protein FlhA	N	N	N	N	N	N
	WP_057499934.1	Eco29kI family restriction endonuclease	N	N	N	N	N	N
	WP_057499935.1	DNA cytosine methyltransferase	N	N	N	N	N	N
	WP_057500949.1	glycosyltransferase	N	N	N	N	N	N
	WP_057501327.1	hypothetical protein	N	N	N	N	N	N
	WP_197718339.1	glycosyltransferase	N	N	N	N	N	N
	WP_232015467.1	flagellar basal body P-ring protein FlgI	N	N	N	N	N	N
	WP_003131974.1	mercuric ion transporter MerT	N	N	N	N	N	N
	WP_003131987.1	mercury resistance system periplasmic binding protein MerP	N	N	N	N	N	N
NZ_LR134324.1	WP_003156770.1	mercury(II) reductase	N	N	N	N	N	N
	WP_005413387.1	Hg(II)-responsive transcriptional regulator	N	N	N	N	N	N
	WP_005413400.1	Tn3-like element TnAs1 family transposase	N	N	N	N	NZ_CP043578.1-2	100
	WP_005416648.	RebB family R body protein	N	N	N	N	N	N

1							
WP_005416649.1	RebB family R body protein	N	N	N	N	N	N
WP_005416651.1	RebB family R body protein	N	N	N	N	N	N
WP_006399830.1	cytochrome o ubiquinol oxidase subunit IV	N	N	N	N	N	N
WP_006446865.1	RebB family R body protein	N	N	N	N	N	N
WP_010921730.1	recombinase family protein	N	N	N	N	N	N
WP_032962419.1	SDR family oxidoreductase	N	N	N	N	N	N
WP_049438932.1	7-cyano-7-deazaguanine synthase QueC	N	N	N	N	N	N
WP_049438934.1	7-carboxy-7-deazaguanine synthase	N	N	N	N	N	N
WP_053494789.1	ABC transporter permease	N	N	N	N	N	N
WP_126411527.1	type I restriction-modification system endonuclease	N	N	N	N	N	N
WP_126411528.1	Fic family protein	N	N	N	N	N	N
WP_126411529.1	N-6 DNA methylase	N	N	N	N	N	N
WP_126411531.	DUF262 domain-containing protein	N	N	N	N	N	N

	1							
	WP_126411959.1	type ISP restriction/modification enzyme	N	N	N	N	N	N
	WP_172601375.1	DUF3304 domain-containing protein	N	N	N	N	N	N
	WP_223224789.1	SRPBCC domain-containing protein	N	N	N	N	N	N
	WP_229653766.1	GlxA family transcriptional regulator	N	N	N	N	N	N
	WP_005407702.1	type II toxin-antitoxin system RelE/ParE family toxin	N	N	N	N	N	N
	WP_005407703.1	putative addiction module antidote protein	N	N	N	N	N	N
	WP_005407950.1	winged helix-turn-helix domain-containing protein	N	N	N	N	N	N
	WP_005408112.1	LysR family transcriptional regulator	N	N	N	N	N	N
NZ_LS483377.1	WP_005408494.1	helix-turn-helix transcriptional regulator	N	N	N	N	N	N
	WP_005408580.1	GyrI-like domain-containing protein	N	N	N	N	N	N
	WP_005408663.1	aminotransferase class III-fold pyridoxal phosphate-dependent enzyme	N	N	N	N	N	N
	WP_005408666.1	DMT family transporter	N	N	N	N	N	N
	WP_005408670.	response regulator	N	N	N	N	N	N

1							
WP_005408850.1	carboxymuconolactone decarboxylase family protein	N	N	N	N	N	N
WP_005408904.1	nuclear transport factor 2 family protein	N	N	N	N	N	N
WP_005408915.1	LysR family transcriptional regulator	N	N	N	N	N	N
WP_005409356.1	metalloregulator ArsR/SmtB family transcription factor	N	N	N	N	N	N
WP_005409501.1	transcriptional regulator BetI	N	N	N	N	N	N
WP_005409502.1	BCCT family transporter	N	N	N	N	N	N
WP_005409633.1	response regulator	N	N	N	N	N	N
WP_005409716.1	aromatic alcohol reductase	N	N	N	N	N	N
WP_005409726.1	ABC transporter permease	N	N	N	N	N	N
WP_005409730.1	LysR family transcriptional regulator	N	N	N	N	N	N
WP_005409813.1	DUF3224 domain-containing protein	N	N	N	N	N	N
WP_005409868.1	hydrolase	N	N	N	N	N	N
WP_005409874.	biopolymer transporter ExbD	N	N	N	N	N	N

1							
WP_005409890.1	type II secretion system major pseudopilin GspG	N	N	N	N	N	N
WP_005409984.1	MgtC/SapB family protein	N	N	N	N	N	N
WP_005410065.1	Rid family hydrolase	N	N	N	N	N	N
WP_005410081.1	putative quinol monooxygenase	N	N	N	N	N	N
WP_005410131.1	MerR family transcriptional regulator	N	N	N	N	N	N
WP_005410145.1	helix-turn-helix domain-containing protein	N	N	N	N	N	N
WP_005410442.1	VOC family protein	N	N	N	N	N	N
WP_005410881.1	formate dehydrogenase subunit beta	N	N	N	N	N	N
WP_005410882.1	formate dehydrogenase subunit gamma	N	N	N	N	N	N
WP_005411280.1	hypothetical protein	N	N	N	N	N	N
WP_005411473.1	cytochrome o ubiquinol oxidase subunit IV	N	N	N	N	N	N
WP_005411594.1	hypothetical protein	N	N	N	N	N	N
WP_005411609.	acyl carrier protein	N	N	N	N	N	N

1							
WP_005412458.1	phage tail sheath subtilisin-like domain-containing protein	NZ_CM001824.1-2, NZ_CP033829.1-1	100	NZ_CP033586.1-2, NZ_LS483377.1-2, NZ_CP051467.1-6, NZ_CP104863.1-2	100	N	N
WP_005413494.1	ABC transporter ATP-binding protein	N	N	N	N	N	N
WP_005413537.1	hypothetical protein	N	N	N	N	N	N
WP_005413562.1	ATP-binding protein	N	N	N	N	N	N
WP_005413615.1	response regulator	N	N	N	N	N	N
WP_005413630.1	LysR family transcriptional regulator	N	N	N	N	N	N
WP_005413654.1	Lrp/AsnC family transcriptional regulator	N	N	N	N	N	N
WP_005413725.1	VOC family protein	N	N	N	N	N	N
WP_005414287.1	response regulator transcription factor	N	N	N	N	N	N
WP_005414661.1	DNA-binding transcriptional regulator	N	N	N	N	N	N
WP_012478717.1	type II toxin-antitoxin system HipA family toxin	N	N	N	N	N	N
WP_012479558.	EamA family transporter	N	N	N	N	N	N

1							
WP_012479691.1	ABC transporter ATP-binding protein	N	N	N	N	N	N
WP_012479990.1	DNA-binding protein	N	N	N	N	N	N
WP_012480385.1	EamA family transporter	N	N	N	N	N	N
WP_012480982.1	VOC family protein	N	N	N	N	N	N
WP_024956137.1	AraC family transcriptional regulator	N	N	N	N	N	N
WP_024956419.1	SRPBCC family protein	N	N	N	N	N	N
WP_024956907.1	LysR substrate-binding domain-containing protein	N	N	N	N	N	N
WP_024957582.1	alpha/beta hydrolase	N	N	N	N	N	N
WP_033833882.1	PDDEXK nuclease domain-containing protein	N	N	N	N	N	N
WP_033835282.1	LysR substrate-binding domain-containing protein	N	N	N	N	N	N
WP_043033649.1	glutathione S-transferase family protein	N	N	N	N	N	N
WP_049429325.1	sugar phosphate isomerase/epimerase family protein	N	N	N	N	N	N
WP_049437387.	TetR/AcrR family transcriptional	N	N	N	N	N	N

1	regulator						
WP_049452801.1	SRPBCC family protein	N	N	N	N	N	N
WP_049459296.1	MFS transporter	N	N	N	N	N	N
WP_060380169.1	DUF2239 family protein	N	N	N	N	N	N
WP_065183880.1	L-seryl-tRNA(Sec) selenium transferase	N	N	N	N	N	N
WP_076738953.1	hypothetical protein	N	N	N	N	N	N
WP_088429930.1	TIGR03571 family LLM class oxidoreductase	N	N	N	N	N	N
WP_088431955.1	L-dopachrome tautomerase-related protein	N	N	N	N	N	N
WP_099490305.1	alpha/beta hydrolase	N	N	N	N	N	N
WP_099490484.1	FAD-dependent monooxygenase	N	N	N	N	N	N
WP_099605019.1	DUF2185 domain-containing protein	N	N	N	N	N	N
WP_108269768.1	AzlD family protein	N	N	N	N	N	N
WP_111118069.1	TIGR03885 family FMN-dependent LLM class oxidoreductase	N	N	N	N	N	N
WP_111118274.	heavy metal response regulator	N	N	N	N	N	N

1	transcription factor						
WP_111151274.1	cytochrome o ubiquinol oxidase subunit III	N	N	N	N	N	N
WP_111197492.1	baseplate J/gp47 family protein	N	N	N	N	N	N
WP_111197640.1	UDP-N-acetylglucosamine 2-epimerase (non-hydrolyzing)	N	N	N	N	N	N
WP_111686856.1	UDP-N-acetylglucosamine 2-epimerase (non-hydrolyzing)	N	N	N	N	N	N
WP_111686893.1	MFS transporter	N	N	N	N	N	N
WP_111687087.1	ATP-binding protein	N	N	N	N	N	N
WP_111687147.1	LysE family translocator	N	N	N	N	N	N
WP_111687156.1	hypothetical protein	N	N	N	N	N	N
WP_111687174.1	DUF2938 domain-containing protein	N	N	N	N	N	N
WP_111687185.1	glycoside hydrolase family 104 protein	N	N	N	N	N	N
WP_111687201.1	GNAT family N-acetyltransferase	N	N	N	N	N	N
WP_111687215.1	Cu(I)-responsive transcriptional regulator	N	N	N	N	N	N
WP_111687278.	nuclear transport factor 2 family protein	N	N	N	N	N	N

1							
WP_111687279.1	TetR family transcriptional regulator	NZ_LS483377.1-1	100	N	N	N	N
WP_111687306.1	sensory rhodopsin transducer	N	N	N	N	N	N
WP_111687314.1	multidrug efflux RND transporter permease subunit	N	N	N	N	N	N
WP_111687335.1	heavy metal response regulator transcription factor	N	N	N	N	N	N
WP_111687375.1	biotin/lipoyl-binding protein	N	N	N	N	N	N
WP_111687399.1	sigma-70 family RNA polymerase sigma factor	N	N	N	N	N	N
WP_111687439.1	helix-turn-helix domain-containing protein	N	N	N	N	N	N
WP_111687443.1	NADH:flavin oxidoreductase/NADH oxidase family protein	N	N	N	N	N	N
WP_111687462.1	agmatine deiminase family protein	N	N	N	N	N	N
WP_111687508.1	SDR family oxidoreductase	N	N	N	N	N	N
WP_111687514.1	SMR family transporter	N	N	N	N	N	N
WP_111687669.1	LysR family transcriptional regulator	N	N	N	N	N	N
WP_111687712.	MFS transporter	N	N	N	N	N	N

	1							
	WP_111687713.1	VOC family protein	N	N	N	N	N	N
	WP_111687774.1	IS481-like element ISStma12 family transposase	N	N	N	N	N	N
	WP_111687926.1	Gfo/Idh/MocA family oxidoreductase	N	N	N	N	N	N
	WP_223846407.1	GlxA family transcriptional regulator	N	N	N	N	N	N
	WP_231914654.1	helix-turn-helix domain-containing protein	N	N	N	N	N	N
	WP_231914697.1	LysR substrate-binding domain-containing protein	N	N	N	N	N	N
	WP_231914703.1	metalloregulator ArsR/SmtB family transcription factor	N	N	N	N	N	N
	WP_231914709.1	DUF1064 domain-containing protein	N	N	N	N	N	N
	WP_269460180.1	helix-turn-helix transcriptional regulator	N	N	N	N	N	N
	WP_004146420.1	BREX protein BrxB domain-containing protein	N	N	N	N	N	N
NZ_LT906480.1	WP_004153231.1	DEAD/DEAH box helicase family protein	N	N	N	N	N	N
	WP_005304583.1	helix-turn-helix transcriptional regulator	N	N	N	N	N	N
	WP_005408112.	LysR family transcriptional regulator	N	N	N	N	N	N

1							
WP_005408332.1	GPW/gp25 family protein	N	N	N	N	N	N
WP_005408904.1	nuclear transport factor 2 family protein	N	N	N	N	N	N
WP_005411473.1	cytochrome o ubiquinol oxidase subunit IV	N	N	N	N	N	N
WP_006375900.1	zincin-like metallopeptidase domain-containing protein	N	N	N	N	N	N
WP_012478672.1	helix-turn-helix transcriptional regulator	N	N	N	N	N	N
WP_012479056.1	ABC transporter ATP-binding protein	N	N	N	N	N	N
WP_012479074.1	SMR family transporter	N	N	N	N	N	N
WP_012479075.1	TIM barrel protein	N	N	N	N	N	N
WP_012479076.1	NAD(P)-dependent oxidoreductase	N	N	N	N	N	N
WP_012479077.1	FAD-dependent oxidoreductase	N	N	N	N	N	N
WP_012479078.1	glycosyltransferase	N	N	N	N	N	N
WP_020200523.1	DUF6127 family protein	N	N	N	N	N	N
WP_020200576.	helix-turn-helix transcriptional regulator	N	N	N	N	N	N

1							
WP_024956190.1	GPW/gp25 family protein	N	N	N	N	N	N
WP_024956193.1	phage tail assembly protein	N	N	N	N	N	N
WP_024956393.1	Hg(II)-responsive transcriptional regulator	N	N	N	N	N	N
WP_024956394.1	mercuric transporter MerT family protein	N	N	N	N	N	N
WP_024956395.1	mercury resistance system periplasmic binding protein MerP	N	N	N	N	N	N
WP_024956414.1	hypothetical protein	N	N	N	N	N	N
WP_024956557.1	alpha/beta hydrolase	N	N	N	N	N	N
WP_024956711.1	DUF2958 domain-containing protein	N	N	N	N	N	N
WP_024956712.1	DUF2285 domain-containing protein	N	N	NZ_CP060022.1-9, NZ_CP060023.1-9, NZ_CP065965.1-5	100	N	N
WP_024956713.1	helix-turn-helix domain-containing protein	N	N	N	N	N	N
WP_024956717.1	DUF2840 domain-containing protein	N	N	NZ_CP060022.1-9, NZ_CP060023.1-9, NZ_CP065965.1-5	100	N	N
WP_024956864.	adenylate/guanylate cyclase	N	N	N	N	N	N

1	domain-containing protein						
WP_024957346.<br>1	glycosyltransferase family 2 protein	N	N	N	N	N	N
WP_024957400.<br>1	DUF305 domain-containing protein	N	N	N	N	N	N
WP_024957601.<br>1	AAA family ATPase	NZ_CP008838.1-2,<br>NZ_LT906480.1-2	100	N	N	NZ_CP065965.1-10	100
WP_024957611.<br>1	hypothetical protein	N	N	N	N	N	N
WP_024957613.<br>1	hypothetical protein	N	N	N	N	N	N
WP_024957746.<br>1	transcriptional regulator	N	N	N	N	N	N
WP_024957931.<br>1	DUF1629 domain-containing protein	N	N	N	N	N	N
WP_024957933.<br>1	metalloregulator ArsR/SmtB family transcription factor	N	N	N	N	N	N
WP_024957960.<br>1	VOC family protein	N	N	N	N	N	N
WP_024957961.<br>1	Na+/H+ antiporter NhaA	N	N	N	N	N	N
WP_024958018.<br>1	AbiEi antitoxin N-terminal domain-containing protein	N	N	N	N	N	N
WP_024958019.<br>1	XRE family transcriptional regulator	N	N	N	N	N	N
WP_024958021.	DUF3322 and DUF2220	N	N	N	N	N	N

1	domain-containing protein						
WP_024958026.1	BrxE family protein	N	N	N	N	N	N
WP_024958027.1	DUF1819 family protein	N	N	N	N	N	N
WP_024958028.1	BREX system P-loop protein BrxC	N	N	N	N	N	N
WP_024958029.1	N-6 DNA methylase	N	N	N	N	N	N
WP_024958031.1	DUF488 domain-containing protein	N	N	N	N	N	N
WP_024958032.1	PglZ domain-containing protein	NZ_CP008838.1-1	100	N	N	N	N
WP_024958033.1	BREX system Lon protease-like protein BrxL	NZ_CP008838.1-1	100	N	N	N	N
WP_024958034.1	hypothetical protein	N	N	N	N	N	N
WP_024958035.1	DUF2793 domain-containing protein	N	N	N	N	N	N
WP_024958036.1	glycosyltransferase family 2 protein	NZ_CP008838.1-1, NZ_CP065965.1-1, NZ_LT906480.1-5	100	N	N	N	N
WP_024958037.1	phage tail protein	NZ_CP008838.1-1, NZ_CP065965.1-1, NZ_LT906480.1-5	100	N	N	N	N
WP_024958039.	DUF2163 domain-containing protein	NZ_CP008838.1-1,	100	N	N	N	N

1		NZ_CP065965.1-1,<br>NZ_LT906480.1-5					
WP_024958040.<br>1	hypothetical protein	NZ_CP008838.1-1,<br>NZ_CP065965.1-1,<br>NZ_LT906480.1-5	100	N	N	N	N
WP_024958041.<br>1	hypothetical protein	NZ_CP008838.1-1,<br>NZ_CP065965.1-1,<br>NZ_LT906480.1-5	100	N	N	N	N
WP_024958042.<br>1	tail protein	NZ_CP008838.1-1,<br>NZ_CP065965.1-1,<br>NZ_LT906480.1-5	100	N	N	N	N
WP_024958043.<br>1	DUF6441 family protein	NZ_CP008838.1-1,<br>NZ_CP065965.1-1,<br>NZ_LT906480.1-5	100	N	N	N	N
WP_024958044.<br>1	hypothetical protein	N	N	N	N	N	N
WP_024958045.<br>1	hypothetical protein	NZ_CP008838.1-1,<br>NZ_CP065965.1-1,<br>NZ_LT906480.1-5	100	N	N	N	N
WP_024958046.<br>1	hypothetical protein	N	N	N	N	N	N
WP_024958047.<br>1	hypothetical protein	N	N	N	N	N	N
WP_024958049.<br>1	head decoration protein	N	N	N	N	N	N
WP_024958050.	S49 family peptidase	NZ_CP008838.1-1,	100	NZ_LT906480.1-8	100	N	N

1		NZ_CP065965.1-1, NZ_LT906480.1-5					
WP_024958052.1	hypothetical protein	N	N	N	N	N	N
WP_024958053.1	hypothetical protein	N	N	N	N	N	N
WP_024958054.1	hypothetical protein	N	N	N	N	N	N
WP_024958058.1	DUF3489 domain-containing protein	NZ_CP008838.1-1, NZ_CP065965.1-1	100	NZ_LT906480.1-8	100	N	N
WP_024958059.1	hypothetical protein	N	N	N	N	N	N
WP_024958060.1	hypothetical protein	N	N	N	N	N	N
WP_024958062.1	site-specific DNA-methyltransferase	NZ_CP008838.1-1, NZ_CP065965.1-1	100	NZ_LT906480.1-8	100	N	N
WP_024958063.1	DUF6362 family protein	N	N	N	N	N	N
WP_024958064.1	hypothetical protein	N	N	N	N	N	N
WP_024958066.1	phage/plasmid primase, P4 family	NZ_CP008838.1-1, NZ_CP065965.1-1	100	NZ_LT906480.1-8	100	N	N
WP_024958069.1	DUF6511 domain-containing protein	N	N	N	N	N	N
WP_024958070.1	hypothetical protein	NZ_CP008838.1-1, NZ_CP065965.1-1	100	NZ_LT906480.1-8	100	N	N

WP_024958074.1	hypothetical protein	N	N	N	N	N	N
WP_024958075.1	hypothetical protein	N	N	N	N	N	N
WP_024958076.1	recombinase family protein	NZ_CP008838.1-1, NZ_CP065965.1-1	100	NZ_LT906480.1-8	100	N	N
WP_024958077.1	DUF2924 domain-containing protein	N	N	N	N	N	N
WP_024958080.1	ImmA/IrrE family metallo-endopeptidase	N	N	N	N	N	N
WP_024958081.1	hypothetical protein	NZ_CP008838.1-1	100	N	N	N	N
WP_024958082.1	hypothetical protein	NZ_CP008838.1-1	100	N	N	N	N
WP_024958541.1	YafY family protein	N	N	N	N	N	N
WP_024958791.1	ISL3 family transposase	N	N	N	N	N	N
WP_032963337.1	IS3 family transposase	N	N	N	N	N	N
WP_032964292.1	DUF488 domain-containing protein	N	N	N	N	N	N
WP_032964294.1	lysozyme	N	N	N	N	N	N
WP_032964308.1	site-specific DNA-methyltransferase	N	N	N	N	N	N

WP_076738308.1	ATP-binding protein	N	N	N	N	N	N
WP_076738309.1	3'-5' exonuclease	N	N	N	N	N	N
WP_076738391.1	DNA cytosine methyltransferase	N	N	N	N	N	N
WP_076738392.1	DUF262 domain-containing protein	N	N	N	N	N	N
WP_143568593.1	hypothetical protein	N	N	N	N	N	N
WP_197697519.1	DNA adenine methylase	N	N	N	N	N	N
WP_197697522.1	YbhB/YbcL family Raf kinase inhibitor-like protein	N	N	N	N	N	N
WP_197697523.1	CBASS oligonucleotide cyclase	N	N	N	N	N	N
WP_223224752.1	DUF3422 family protein	N	N	N	N	N	N
WP_224119399.1	SMI1/KNR4 family protein	N	N	N	N	N	N
WP_231910752.1	SDR family oxidoreductase	N	N	N	N	N	N
WP_231910796.1	OST-HTH/LOTUS domain-containing protein	N	N	N	N	N	N
WP_231910802.1	elements of external origin	N	N	N	N	N	N

	WP_231910804.1	phage portal protein	NZ_CP008838.1-1, NZ_CP065965.1-1, NZ_LT906480.1-5	100	NZ_LT906480.1-8	100	N	N
	WP_231910805.1	major capsid protein	NZ_CP008838.1-1, NZ_CP065965.1-1, NZ_LT906480.1-5	100	N	N	N	N
	WP_231910821.1	SDR family oxidoreductase	N	N	N	N	N	N
	WP_231910850.1	type II toxin-antitoxin system RelE/ParE family toxin	N	N	N	N	N	N
	WP_231910863.1	NgoMIV family type II restriction endonuclease	N	N	N	N	N	N
	WP_231910988.1	substrate binding domain-containing protein	N	N	N	N	N	N
	WP_269458352.1	replication-associated recombination protein A	N	N	N	N	N	N
NZ_OU943334.1	WP_003050225.1	TIGR03758 family integrating conjugative element protein	N	N	N	N	N	N
	WP_003050245.1	helix-turn-helix domain-containing protein	N	N	N	N	N	N
	WP_003050273.1	CBASS effector endonuclease NucC	N	N	N	N	N	N
	WP_003050422.1	hypothetical protein	N	N	N	N	N	N
	WP_003090093.1	AlpA family transcriptional regulator	N	N	N	N	N	N

WP_003090097.1	DUF2857 domain-containing protein	N	N	N	N	N	N
WP_003090159.1	type III CBASS phage resistance system CD-NTase-associated protein Cap7	N	N	N	N	N	N
WP_003090173.1	TIGR03745 family integrating conjugative element membrane protein	N	N	N	N	N	N
WP_003090202.1	thioredoxin domain-containing protein	N	N	N	N	N	N
WP_003090203.1	JAB domain-containing protein	N	N	N	N	N	N
WP_003090212.1	hypothetical protein	N	N	N	N	N	N
WP_003090214.1	conjugal transfer protein TraG N-terminal domain-containing protein	N	N	N	N	N	N
WP_003090303.1	DUF411 domain-containing protein	N	N	N	N	N	N
WP_003090319.1	copper homeostasis periplasmic binding protein CopC	N	N	N	N	N	N
WP_003097515.1	copper resistance system multicopper oxidase	N	N	N	N	N	N
WP_003097522.1	copper homeostasis membrane protein CopD	N	N	N	N	N	N
WP_003097544.1	IS21-like element ISPa36 family helper ATPase IstB	N	N	N	N	N	N
WP_003097546.1	IS21-like element ISPa36 family transposase	N	N	N	N	N	N

WP_003097548.1	integrase arm-type DNA-binding domain-containing protein	N	N	N	N	N	N
WP_003098886.1	TIGR03759 family integrating conjugative element protein	N	N	N	N	N	N
WP_003098890.1	PilL N-terminal domain-containing protein	N	N	N	N	N	N
WP_003098893.1	helicase-related protein	N	N	N	N	N	N
WP_003098899.1	DUF6094 domain-containing protein	N	N	N	N	N	N
WP_003098901.1	hypothetical protein	N	N	N	N	N	N
WP_003098908.1	hypothetical protein	N	N	N	N	N	N
WP_003098913.1	DUF3275 family protein	N	N	N	N	N	N
WP_003098915.1	DUF932 domain-containing protein	N	N	N	N	N	N
WP_003098919.1	DUF3577 domain-containing protein	N	N	N	N	N	N
WP_003098932.1	mercury(II) reductase	N	N	N	N	N	N
WP_003098934.1	mercury resistance system periplasmic binding protein MerP	N	N	N	N	N	N
WP_003098936.1	mercuric ion transporter MerT	N	N	N	N	N	N

WP_003098939.1	Hg(II)-responsive transcriptional regulator	N	N	N	N	N	N
WP_003098941.1	hypothetical protein	N	N	N	N	N	N
WP_003098947.1	hypothetical protein	N	N	N	N	N	N
WP_003098949.1	hypothetical protein	N	N	N	N	N	N
WP_003098955.1	signal peptidase II	N	N	N	N	N	N
WP_003098961.1	heavy metal translocating P-type ATPase	N	N	N	N	N	N
WP_003098965.1	Cd(II)/Pb(II)-responsive transcriptional regulator	N	N	N	N	N	N
WP_003098972.1	cation transporter	N	N	N	N	N	N
WP_003098976.1	single-stranded DNA-binding protein	N	N	N	N	N	N
WP_003098978.1	DUF3158 family protein	N	N	N	N	N	N
WP_003098981.1	TIGR03761 family integrating conjugative element protein	N	N	N	N	N	N
WP_003098985.1	ParB family protein	NZ_CP060023.1-2	100	N	N	N	N
WP_003098988.1	ParA family protein	NZ_CP060023.1-2	100	N	N	N	N

WP_003098996.1	DUF305 domain-containing protein	N	N	N	N	N	N
WP_003105079.1	conjugative transfer ATPase	N	N	N	N	N	N
WP_003105620.1	TIGR03747 family integrating conjugative element membrane protein	N	N	N	N	N	N
WP_003105624.1	CBASS oligonucleotide cyclase	N	N	N	N	N	N
WP_003105639.1	TIGR03750 family conjugal transfer protein	N	N	N	N	N	N
WP_003105641.1	TIGR03746 family integrating conjugative element protein	N	N	N	N	N	N
WP_003105643.1	TIGR03749 family integrating conjugative element protein	N	N	N	N	N	N
WP_003107233.1	chromate efflux transporter	N	N	N	N	N	N
WP_003107241.1	arsenate reductase ArsC	N	N	N	N	N	N
WP_003107243.1	ArsI/CadI family heavy metal resistance metalloenzyme	N	N	N	N	N	N
WP_003109768.1	MobH family relaxase	N	N	N	N	N	N
WP_003109770.1	integrating conjugative element protein	N	N	N	N	N	N
WP_003109772.1	TIGR03757 family integrating conjugative element protein	N	N	N	N	N	N

WP_003109775.1	TIGR03751 family conjugal transfer lipoprotein	N	N	N	N	N	N
WP_003109776.1	TIGR03752 family integrating conjugative element protein	N	N	N	N	N	N
WP_003109779.1	integrating conjugative element protein	N	N	N	N	N	N
WP_003109780.1	transglycosylase SLT domain-containing protein	N	N	N	N	N	N
WP_003116799.1	helix-turn-helix domain-containing protein	N	N	N	N	N	N
WP_003116813.1	type IV conjugative transfer system coupling protein TraD	N	N	N	N	N	N
WP_005408883.1	copper resistance protein B	N	N	N	N	N	N
WP_005408896.1	STY4528 family pathogenicity island replication protein	N	N	N	N	N	N
WP_005411473.1	cytochrome o ubiquinol oxidase subunit IV	N	N	N	N	N	N
WP_008786730.1	glutaredoxin family protein	N	N	N	N	N	N
WP_008786732.1	MFS transporter	N	N	N	N	N	N
WP_008786734.1	efflux transporter outer membrane subunit	N	N	N	N	N	N
WP_008786735.1	efflux RND transporter permease subunit	N	N	N	N	N	N

WP_008786737.1	metalloregulator ArsR/SmtB family transcription factor	N	N	N	N	N	N
WP_008786738.1	cation diffusion facilitator family transporter	N	N	N	N	N	N
WP_008786740.1	MFS transporter	N	N	N	N	N	N
WP_008786741.1	metalloregulator ArsR/SmtB family transcription factor	N	N	N	N	N	N
WP_008786745.1	glutaredoxin	N	N	N	N	N	N
WP_008786747.1	LysR substrate-binding domain-containing protein	N	N	N	N	N	N
WP_008786748.1	EexN family lipoprotein	N	N	N	N	N	N
WP_008786751.1	CopG family transcriptional regulator	N	N	N	N	N	N
WP_008786753.1	TrbC/VirB2 family protein	N	N	N	N	N	N
WP_008786754.1	VirB3 family type IV secretion system protein	N	N	N	N	N	N
WP_008786756.1	P-type conjugative transfer protein TrbJ	N	N	N	N	N	N
WP_008786757.1	hypothetical protein	N	N	N	N	N	N
WP_008786881.1	TrbI/VirB10 family protein	N	N	N	N	N	N

WP_008786882.1	P-type conjugative transfer protein TrbG	N	N	N	N	N	N
WP_008786883.1	conjugal transfer protein TrbF	N	N	N	N	N	N
WP_010483777.1	GPW/gp25 family protein	N	N	N	N	N	N
WP_014747691.1	PIN domain-containing protein	N	N	N	N	N	N
WP_015014846.1	PadR family transcriptional regulator	N	N	N	N	N	N
WP_019396553.1	Lrp/AsnC ligand binding domain-containing protein	N	N	N	N	N	N
WP_021162752.1	efflux RND transporter periplasmic adaptor subunit	N	N	N	N	N	N
WP_021202108.1	metalloregulator ArsR/SmtB family transcription factor	N	N	N	N	N	N
WP_021221531.1	P-type conjugative transfer protein TrbL	N	N	N	N	N	N
WP_023123028.1	S26 family signal peptidase	N	N	N	N	N	N
WP_023123029.1	DUF2840 domain-containing protein	N	N	N	N	N	N
WP_023123030.1	hypothetical protein	N	N	N	N	N	N
WP_023123034.1	helix-turn-helix domain-containing protein	N	N	N	N	N	N

WP_023123035.1	DUF2285 domain-containing protein	N	N	N	N	N	N
WP_023123036.1	DUF2958 domain-containing protein	N	N	N	N	N	N
WP_023123037.1	helix-turn-helix transcriptional regulator	N	N	N	N	N	N
WP_023123040.1	abortive infection family protein	N	N	N	N	N	N
WP_023123041.1	DNA repair protein RadC	N	N	N	N	N	N
WP_023123042.1	AAA family ATPase	N	N	N	N	N	N
WP_023123043.1	PDDEXK nuclease domain-containing protein	N	N	N	N	N	N
WP_023123044.1	site-specific integrase	N	N	N	N	N	N
WP_024957611.1	hypothetical protein	N	N	N	N	N	N
WP_029885645.1	replication initiator protein A	N	N	N	N	N	N
WP_031629745.1	DUF3085 domain-containing protein	N	N	N	N	N	N
WP_049461335.1	ABC transporter permease	N	N	N	N	N	N
WP_049461337.1	ABC transporter ATP-binding protein	N	N	N	N	N	N

WP_057502819.1	nucleoside triphosphatase NudI	N	N	N	N	N	N
WP_057503924.1	NADPH-dependent FMN reductase	N	N	N	N	N	N
WP_057504370.1	metalloregulator ArsR/SmtB family transcription factor	N	N	N	N	N	N
WP_081280899.1	arsenate reductase ArsC	N	N	N	N	N	N
WP_164191178.1	TIGR03885 family FMN-dependent LLM class oxidoreductase	N	N	N	N	N	N
WP_164192588.1	SRPBCC family protein	N	N	N	N	N	N
WP_169708473.1	metal-sensing transcriptional repressor	N	N	N	N	N	N
WP_216655222.1	uracil-DNA glycosylase	N	N	N	N	N	N
WP_223224739.1	IS3 family transposase	N	N	N	N	N	N
WP_228354833.1	alpha/beta hydrolase	N	N	NZ_OU943334.1-3	100	N	N
WP_228354903.1	WecB/TagA/CpsF family glycosyltransferase	N	N	N	N	N	N
WP_228355053.1	DUF3164 family protein	N	N	N	N	NZ_OU943334.1-4	100
WP_228355059.1	AAA family ATPase	N	N	N	N	NZ_OU943334.1-4	100

WP_228355208.1	AAA family ATPase	N	N	N	N	N	N
WP_228355209.1	DNA topoisomerase III	N	N	N	N	N	N
WP_228355239.1	DUF5655 domain-containing protein	N	N	N	N	N	N
WP_228355242.1	ATP-dependent DNA helicase	N	N	N	N	N	N
WP_228355244.1	TnsA endonuclease N-terminal domain-containing protein	N	N	N	N	N	N
WP_228355325.1	GNAT family N-acetyltransferase	N	N	N	N	N	N
WP_228355386.1	sulfite exporter TauE/SafE family protein	N	N	N	N	N	N
WP_228777907.1	heavy metal translocating P-type ATPase	N	N	N	N	N	N

Table S7: Prediction of CRISPR-Cas and CRISPR spacers targeted *S. maltophilia* prophages carried by *S. maltophilia* and known phages in public database, N stands for no.

Genome GenBank	Spacer number	Spacer OTUs	Spacer sequence	Spacer source	Match incomplete prophage	Match intact prophage	Match questionable prophage	Match known phage	Identity (%)
CP050452.1	3	1	TTTGCGATGAGGGCTGTTGCCGGCCAGCGGCCGGCACTACCGGAGCG	NZ_CP049956.1	NZ_LT906480.1-6, NZ_CP065965.1-9, NZ_CP065965.1-7, NZ_CP049956.1-7, NZ_CP049368.1-3, NZ_CP040437.1-4, NZ_CP014014.1-7, NC_010943.1-3, NZ_CP104292.1-2	NZ_LT906480.1-1	N	N	100
CP071784.1	2	2	TTGGTGGGTGCCAACCTTGGTTGGCACCGATGCTGCCCCGGTAGCC	NZ_CP040438.1	N	N	N	N	N
CP078102.1	14	3	TTGCGATGCGCAAGCGGGAAAAGGTCACGCGCGGTGC	NZ_CP052863.1	N	N	N	N	N
CP091781.1	11	4	TTCGCGGCCAGTAGATCCACGCCATGCGTGGATGCGCCGTGGCCTG	NZ_CP056088.1	NZ_CP040432.1-2	N	N	N	100

			CGCTTTCT						
NC_010943.1	12	5	TTCGATGGATGTTCATCCACGCATGGCGTGGATCTACCGGTG	NZ_CP018756.1	N	N	N	N	N
NC_011071.1	11	6	TTCATCGGCCTGGCAGTAGATCCACGCCATGCGTGGATGTGACGTCAG	NZ_CP051467.1	N	N	N	N	N
NC_015947.1	13	7	TTCATCGGCCCGGCAGTAGATCCACGCCATGCGTGGATGTGACGGCTG	NZ_CP104863.1	N	N	N	N	N
NC_017671.1	7	8	TGTGGTGGGCCAGGCGTCCGGTAAGTCGAGCATGGCTCGACTCTACAAG	NC_015947.1	N	N	N	N	N
NZ_AP021867.1	17	9	TGTCGGAGTCGTCCCAGTAGAGCCACGCCATGCGTGGATGAATTCAT	NZ_CP018756.1	N	N	N	N	N
NZ_AP021908.	8	10	TCGATATCTGACTGGTGCTCGCCC	NZ_CP008838.1	N	N	N	N	N

1			GCTGCGATGATGC							
NZ_CM001824.1	12	11	TGGTGAAGCGGGTAGGTGCCGACCGTTGGTCGGCACACCGGTTATTGCATG	NZ_CP098483.1	N	N	N	N	N	
NZ_CP008838.1	15	12	TGGTAGATCCGCGCCGTGCACGGATGGGAAA	NZ_CP104286.1	N	N	N	N	N	
NZ_CP011010.1	9	13	TGGGCCTCTGGTGGGTGAGGACCTTGGTCCACGCCGCTCTTCGGTGGGAA	NZ_CP049368.1	N	N	N	N	N	
NZ_CP011305.1	2	14	TGGGCAGAGGGAGCCTGGTAGAGGCCGACCTTGGTCGGCGCATACGGTGTGCGTGC	NZ_AP021687.1	NZ_CP049368.1-2, NZ_CP049956.1-6, NZ_CP044092.1-6, NZ_CP040439.1-7, NZ_CP104288.1-4	N	N	N	100	
NZ_CP014014.1	9	15	TGGCGCGCGTGGGCGGACAAGATCATGGGCGAT	NZ_CP052863.1	N	N	N	N	N	
NZ_CP015612.1	12	16	TGGCCGTGCCGACTTTGGTGGGTGCCGACCGTTG	NZ_CP037858.1	N	NZ_CP022053.2-4, NZ_CP022053.2-3	N	N	100	

			GTCGGCACGGA AGCCCGTGCCTC T						
NZ_CP018756.1	31	17	TGGCACCTACCA GGAGCGAGTCA TCAGC	NZ_CP022053.2	N	N	N	N	N
NZ_CP022053.2	9	18	TGGCACCCACC GGAAGTGATCG CTCTCGGTGGGT GCGGACCGTTG GTCCGCACTGTC T	NZ_CP104287.1	N	N	N	N	N
NZ_CP025298.1	8	19	TGGCACAACTCC CACCGACGCCA TGACCTT	NZ_LR134301.1	N	N	N	N	N
NZ_CP027562.1	9	20	TGGAAGTGTCTG GAAGTTCATCCA CGCATGGCGTG GATCTGCTGGA AGTGTCTGGAA	NZ_CP040429.1	N	N	N	N	N
NZ_CP028358.1	11	21	TGCTTGATCTGC CGGCCAGCGGC CGGCACTACCC GATCATTGCCTG GTGCT	NZ_CP040432.1	NZ_CP049956.1-9, NZ_CP031058.1-2	NZ_CP060026.1-3, NZ_CP040440.1-5, NZ_CP028358.1-3, NZ_CP101622.1-1	NZ_CP022053.2-8	N	100
NZ_CP028899.	8	22	TGCTCCTGGTAG	NZ_CP043578.1	N	N	N	N	N

1			GTGCCGAGCTTG<br>CTCGGCACGCTC<br>TCCATCGGTGAT						
NZ_CP029773.1	9	23	CGCACGCTGCAT<br>TGCGCCGGTGA<br>AGAAGGCTGCC<br>GCCAAGCCAGT<br>TGCCGACCGTTG	NZ_CP033586.1	N	N	N	N	N
NZ_CP031058.1	7	24	GTCGGCACGCC<br>GTTAAAGCCGC<br>CCGACCAACGG<br>TCGGGCGCTACC<br>GGTCGGTT	NZ_CP018756.1	N	N	N	N	N
NZ_CP033586.1	14	25	TGCCCTGCCGGC<br>CAGCGGCCGGC<br>ACTACCGCATCC<br>CCGGTG	NZ_CP037858.1	NC_010943.1 -3	N	N	N	100
NZ_CP033829.1	16	26	TGCCCCCATGGC<br>CGTTCATCCAAG<br>CATGGCGTGGA<br>TCTACTGGAGAT<br>GGCGG	NZ_CP040429.1	N	N	N	N	N
NZ_CP033877.1	12	27	TGCCATGCATGG<br>GCGCCCACCAA<br>GGTGGGCATCT<br>ACCAGAGCGCT	NZ_LR134301.1	N	N	N	N	N

			GTACCGC						
NZ_CP037858.1	7	28	TGATCCAATCCCGATCAGAATCGGTGCCTGCCGCTCC	NZ_CP011305.1	N	N	N	N	N
NZ_CP040429.1	13	29	TGATCCAATCCCGACCAGAATCGGTGCCTGCCGCTCC	NZ_OU943334.1	N	N	N	N	N
NZ_CP040430.1	15	30	TGAGGCTGCTCTGGTGGGTGCCGACCGTTGGTCGGCACTGCGCCAGCCCCGC	NZ_CP067993.1	NZ_CP049368.1-2	N	N	N	100
NZ_CP040431.1	9	31	TGAATCGGTTGGGCACAAGCCCTGGTAGCGCCGGGCATG	NZ_CP040429.1	N	N	N	N	N
NZ_CP040432.1	16	32	TCTTTGCTGCTTTGGTGGGTGCCGACCTTGGTCGGCGCGCCACAAATCTCC	NC_015947.1	N	N	N	N	N
NZ_CP040433.1	0	33	TCTTTCCCGCATCCACGCGTGGCGTGGATCTACC	NZ_CP015612.1	N	N	N	N	N

NZ_CP040434.1	8	34	TCTGCCTGGGTCTGGTAGTTGCCAACCTTGGTTGGCA	NZ_CP040432.1	N	N	N	N	N
NZ_CP040435.1	4	35	TCTGATCTGCCGGCCAGCGGCCGGCACTACCGATCACGGCCTGCCGC	NZ_CP029773.1	N	N	N	N	N
NZ_CP040436.1	4	36	TCGTGTATGGGAATGCATTGCGCGCCGCCGGGCATCGGTCCGTGACCTTATGAATC	NZ_CP106759.1	N	N	N	N	N
NZ_CP040437.1	5	37	TCGCGATCATCCACACATGGCGCGGATCTACTGAAGGAGCCTAGCTCTGGTGGG	NZ_CP049368.1	N	N	N	N	N
NZ_CP040438.1	15	38	TCGCAACCCGCTTTGGTGGGGGCCGACCGTTGGTCGGCACGGATGT	NZ_CP104292.1	N	N	N	N	N
NZ_CP040439.1	9	39	CTGTGCCTCTCGTCCGGTGACTGGACGGGCTTCGGT	NZ_CP018756.1	N	N	N	N	N

			AGATGCCAACC TTGGTTGGCCCC C						
NZ_CP040440.1	9	40	TCCGGTAGTGCC GGCCGCTGGCC GGCAACAGCCC TCGTCGCAAA	NZ_CP104863.1	NZ_LT906480.1-6, NZ_CP088241.1-5, NZ_CP065965.1-9, NZ_CP065965.1-7, NZ_CP049956.1-7, NZ_CP049956.1-6, NZ_CP049368.1-3, NZ_CP040437.1-4, NZ_CP014014.1-7, NC_010943.1-3, NZ_CP104292.1-2	NZ_LT906480.1-1	N	N	100
NZ_CP043578.1	16	41	TCCGGCGTGGTG GGTGCCGACCG TTGGTCGGTACA CCCCTGATGTCG	NZ_CP015612.1	N	N	N	N	N
NZ_CP044092.1	17	42	TCCAGGTGATGC TTCAATCGTTCA	NZ_CP104863.1	N	N	N	N	N
NZ_CP047310.1	3	43	TCCAACGGTTCG CACCCACCCGA GTCAGACC	NZ_CP040430.1	N	N	N	N	N
NZ_CP049368.1	8	44	TCAGCACGGTTG GAATGTGCCAA CCAACAGTT	NZ_CP028899.1	N	N	N	N	N
NZ_CP049956.1	12	45	TCACATCAGCCG GTAGGTGCCAA CCTTGGTTGGCA CGCTCACCTCAC CCGGT	NZ_CP056088.1	N	NZ_CP031058.1-1	N	N	100

NZ_CP051467.1	11	46	TATCGGCGCGCCCCCTTGAAACGCAGGCGCCGTTGGCGCCGGCGTTCCG	NZ_CP022053.2	N	N	N	N	N
NZ_CP052863.1	16	47	TATCGCGCAGCGCGGGCCTGCCCCCAGATCGA	NZ_CP018756.1	N	N	N	N	N
NZ_CP056088.1	9	48	TAGGTGCCAACCTTGGTTGGCACGGATGTGTCAGC	NZ_CP043578.1	N	N	NZ_CP022053.2-8, NZ_CP060026.1-10, CP050452.1-2	N	100
NZ_CP060021.1	8	49	TACTGACCTCTGGTGGGTGCCGACCGTTGGTCGGCACTGCC	NZ_CP025298.1	NZ_CP049368.1-2	N	N	N	100
NZ_CP060022.1	6	50	TACCCTCAATCGACAGAGATTCATCCACCGACAGAT	NZ_CP049368.1	N	N	N	N	N
NZ_CP060023.1	6	51	TACCCGCGTGGGACAAGGCGTGCCGACCAACGGTCGGCACCTACAGCGTCAA	NZ_CP067993.1	N	N	N	N	N
NZ_CP060024.	1	52	TACCCACCAATG	NZ_CP104289.1	N	N	N	N	N

1			TGGTAGTGCCG<br>GCCGCTGGCCG<br>GCAAA						
NZ_CP060025.<br>1	0	53	TACCAGGTCGG<br>GTCGAGCGTGC<br>CGACCAAGGTT<br>GGCACCTACCA<br>GGCTGGGTCG	NZ_CP031058.1	N	N	N	N	N
NZ_CP060026.<br>1	10	54	TACCAGGGCCA<br>AGACCCGCTGC<br>GGTAAATCCAC<br>GCCATGCGTGG<br>ATGCAT	NZ_CP080573.1	N	N	N	N	N
NZ_CP060027.<br>1	12	55	TACCAGAGCCG<br>TGTGCTGCCAGG<br>CGTCATGCG	NZ_CP077679.1	N	N	N	N	N
NZ_CP060259.<br>1	4	56	TACCACAGCAC<br>ACCGGTAGCGC<br>CGGGCCATGCC<br>CGGCGGAG	NZ_CP018756.1	NZ_CP049368.1-2	N	N	N	100
NZ_CP065965.<br>1	16	57	TACCAAAGCCG<br>TGTGCTGCCAGG<br>CGTCAAGCCCG<br>CCGACGG	NZ_LR134324.1	N	N	N	N	N
NZ_CP067993.<br>1	10	58	NCCGGNCACCC<br>AGAGCAGCCGG	NZ_CP060023.1	N	N	N	N	N

			CCNGCGGCCGG CTCTACCGGGGC CAATCGAC						
NZ_CP077679.1	2	59	NACCAAAGCCG TGTGCTGCCAGG CGTCAAGCCCG CCGACGG	NZ_CP060023.1	N	N	N	N	N
NZ_CP080573.1	9	60	GTTGCGCGATA GAGCTTCATCCA CGCATGGCGTG GATCTACTGACA CCATGATTCT	NZ_CP040438.1	N	N	N	N	N
NZ_CP083454.1	8	61	GTTAAGGTGGA ATGCGCGGAAC GCCGGCGCCCA AGGCGCCTGCG TTTCAAGGGGG CGCGCCGACAG GCGGGGGGGTA AGGTGGAATGC GCGGAACGCT	NZ_CP043578.1	N	N	N	N	N
NZ_CP088240.1	14	62	GTGTGGCATTGC GGCTGATGAAG GGGCGGTTCCG CGTGGTCA	NC_015947.1	N	N	N	N	N
NZ_CP088241.	14	63	GTGTGGCAATG	NZ_CP025298.1	N	N	N	N	N

1			CGGTTGATGAA GGTGCGGTTCCG CGTGGTCAT						
NZ_CP088242.1	8	64	GTGCCGCTGCGC TTGCCGGGCATT GGCCCGACGCT ACCGATGCGGG ATCTGACTTGGT GT	CP071784.1	N	N	N	N	N
NZ_CP088243.1	8	65	GTGCCACACCCC CCGGTAGCGCC GGGCCATGCCC GGCGGATGTG	NZ_CP018756.1	NZ_CP037858.1-1	N	N	N	100
NZ_CP088244.1	2	66	GTGCCAAGCCC CCGGTAGGTGTC GACCTTGGTCGA CACGCATTACAC GGC	NZ_LR134301.1	N	N	N	N	N
NZ_CP090418.1	10	67	GTAGAGCCGAG CCCATGCTCGGC TGATCGCCGGA	NZ_CP018756.1	N	N	N	N	N
NZ_CP090423.1	6	68	GTAGAGCCACG CCACGCGTGGA TGCATTTCGCGC	NZ_CP018756.1	N	N	N	N	N
NZ_CP098483.	7	69	ACGGCCAGAG GGTTTCCACGCC	NZ_CP040429.1	N	N	N	N	N

1			TTCGCCGACACG GAT						
NZ_CP101622.1	13	70	GGTGGGTGCCG ACCGTTGGTCGG CAGTGGGCCAG CCCCGC	NZ_CP015612.1	N	N	N	N	N
NZ_CP102942.1	10	71	GGTCGACTATCG CGCGCAGTGCG GGGTTTCGCTAC CTGATCGAAGA GCAGTCGACTG ACAGTCGACTCT ACCCCGGTCGA CCATCGCGCGC AGCGCGGGGGT	NZ_CP104288.1	N	N	N	N	N
NZ_CP104071.1	3	72	GGTCGAAGCGC GCGGCCAGGCG CGGATCGGCCTT GGCTTCCTCGCG GCTGATGCG	NZ_CP090423.1	N	N	N	N	N
NZ_CP104169.1	1	73	GGTCCGGCTTGG TGGGTGCCGAC CGTTGGTCGGCA CACCTTTCAAT	NC_015947.1	NZ_CP049956.1-6, NZ_CP044092.1-6, NZ_CP040439.1-7	N	N	N	100
NZ_CP104286.1	12	74	GGTCAGTCGGG GGCCCTGGCCG	NZ_CP040439.1	NZ_OU943334.1-2, NZ_CP104289.1-3, NZ_CP088241.1-5, NZ_CP049956.1-9,	NZ_CP060026.1-3,NZ_CP040440.1-5, NZ_CP028358.1-3,NZ_CP101622.1-1,	CP050452.1-2,NZ_CP022053.2-8,NZ_ CP060026.1-10	N	100

			GCATCGCGCCGT<br>GCAGGACGTCA<br>GGGGGTTGCCG<br>GCCAGCGGCCG<br>GCACTACCCGTA<br>AATCGGGGGCT<br>CTGGCCGGCATT		NZ_CP049956.1-7, NZ_CP040437.1-4,<br>NZ_CP088243.1-4, NZ_CP088242.1-4,<br>NZ_CP088241.1-2, NZ_CP065965.1-6,<br>NZ_CP051467.1-5, NZ_CP049368.1-3,<br>NZ_CP044092.1-5, NZ_CP040438.1-2,<br>NZ_CP040436.1-3, NZ_CP040435.1-5,<br>NZ_CP037858.1-2, NZ_CP008838.1-5,<br>NZ_CP101622.1-3, NZ_CP080573.1-5	NZ_LT906480.1-1,NZ_CP049368.1-1,<br>NZ_CP022053.2-3,NZ_CP022053.2-2			
NZ_CP104287.<br>1	8	75	GGTAGTGCCGG<br>CCGCTGGCCGG<br>CATTCCCATTCG	NZ_CP014014.1	N	N	N	N	N
NZ_CP104288.<br>1	13	76	GGTAGATCCAC<br>GCCATGCGTGG<br>ATGGGTGATCC<br>GTCA	NZ_CP018756.1	N	N	N	N	N
NZ_CP104289.<br>1	5	77	GGGTGTGCCGA<br>CCGGATGCA	NZ_CP052863.1	N	N	N	N	N
NZ_CP104290.<br>1	12	78	GGGTGGTGATTC<br>ACCTGCCACCTT<br>GGGC	NZ_CP101622.1	N	N	N	N	N
NZ_CP104292.<br>1	8	79	GGGTGATGTGA<br>GCGTGCCGACC<br>AAGGTCGGCAC<br>CTACCGGGTGAT<br>GTGAGCA	NZ_AP021687.1	N	N	N	N	N
NZ_CP104323.	7	80	GGGGTCTGTGA	NZ_CP015612.1	N	N	N	N	N

1			GGCATGGTAGA GGCCGACCTTG GTC						
NZ_CP104324.1	3	81	GGGGGCGCCAA CCAAGGTTGGC GGCTACCAGGG CGTCGGTGCCGC GATATCCGG	NZ_CP049368.1	N	N	N	N	N
NZ_CP104863.1	14	82	GGGGCTGTGTG GTGCCGACCAA GGTCGGCGACT ACCGGGGCTGT GTGGT	NZ_CP028358.1	N	N	N	N	N
NZ_CP106759.1	1	83	GGGGCCCACGA GACATGGTAGA GGCCGACCTTG GTCGGCGCTTGC CGATTGT	NZ_CP040439.1	N	N	N	N	N
NZ_LR134301.1	5	84	GGGGAATAGGA AGAATGCGCGG GCAACCCAGCG CGCCAACGGCG GGCTGGGTTGC	NC_015947.1	N	N	N	N	N
NZ_LR134324.1	6	85	GGGCTTGGATCT ACCGACCTCTGG TGGGTACTGACC	NZ_CP044092.1	N	N	N	N	N

			TTTGGTGGGGGCCGACCG						
NZ_LS483377.1	11	86	GGGCGGCGCGCCGTGCGTCCGTTGATCGTTCGCGCGGCGGGTGAAGGGTGC	NZ_CP022053.2	N	N	N	N	N
NZ_LS483406.1	8	87	GGGCGCCGTTGATGTCCTGCGTGCCAACCAAGGTTGGCACCTACCAAAAGCGCCG	NZ_CP040430.1	NZ_CP049956.1-6	N	N	N	100
NZ_LT906480.1	16	88	GGGCGATGTGAGCGTGCCGACCAAGGTCGGCACCTACCGGGCGATGTGAGCG	NZ_CP040432.1	N	N	N	N	N
NZ_OU943334.1	4	89	GGGCCTGCGCGGCGCTGCTTTGGTAGCTGCCCACCTTGGTGGGCACCGATGGAAGCCA	NC_011071.1	N	N	N	N	N
		90	GGGCCTGAGCGGTGTTGCTTTGGTAGATGCCCACCTTGGTGGGCG	NZ_AP021687.1	N	N	N	N	N

91	GGCGGTTTCGGT GGTGAAGGCCG TGGT	NZ_CP011010.1	N	N	N	N	N
92	GGCGGGCCAGG ACATCCGCAGC CACCCATGGGG TGGCTCTACTG	NZ_CP049368.1	N	N	N	N	N
93	GGCCTTCTTCAC CGCCTTCTTGGC CG	NZ_CP090423.1	N	N	N	N	N
94	GGCCCACGGCC GGCGGATTCCG GTAGATGCCAA CCTTGGTTGGCA CCGTTGATGTTC C	NZ_CP025298.1	N	N	N	N	N
95	GGCACTTCGCA GGCCAACCAAG GTTGGCCACTAC CGGTTCATGCAG GGCCGGAACGC T	NZ_CP028358.1	N	N	N	N	N
96	GGATGATCCGC GGACGCACCGT GCATCGCTTTGG TGGGTGCCGAC	CP050452.1	N	N	N	N	N

	CGTTGGCG GGATCCGCGCG TGGTGGTTGCAT						
97	CTGGTAGGTGC GGGCCTTGGTCC GCACGCT	NZ_CP040434.1	N	N	N	N	N
98	GGATCACCGCA GCGGGCGAGCA CCA	NZ_CP014014.1	N	N	N	N	N
99	GGACGGTTTGGT TGCCGGCCAGC GGCCGGCACTA CCGAAGCCAAT CCACGCATGAC CG	NC_015947.1	NZ_CP104292.1-2, NZ_CP088241.1-5, NZ_CP049956.1-7, NZ_LT906480.1-6, NZ_CP065965.1-9, NC_010943.1-3, NZ_CP088241.1-2, NZ_CP065965.1-7, NZ_CP065965.1-6, NZ_CP051467.1-5, NZ_CP049956.1-6, NZ_CP049368.1-3, NZ_CP044092.1-5, NZ_CP040437.1-4, NZ_CP040432.1-2, NZ_CP031058.1-2, NZ_CP014014.1-7, NZ_CP101622.1-3	NZ_LT906480.1-1, NZ_CP060026.1-1, NZ_CP040440.1-1, NZ_CP022053.2-2	NZ_CP060026.1-10	N	100
100	GGAAGCATGGA GTGTCGCGATCT G	NZ_CP028358.1	N	N	N	N	N
101	GCTTCGGTGAAC CTGCAGCGAGG CATGGCCTGATC CT	NZ_CP088244.1	N	N	N	N	N
102	GCTGTTCTGGAT	NZ_CP031058.1	N	N	N	N	N

	GCATGCCCACC<br>AAGGTGGGCAT<br>CTACCAGAGCG<br>CTGTACCCC						
103	GCTGCGTGTCGA<br>CCAAGGTCGAC<br>ACCTACCGGATC<br>GGATCAGCGCT<br>GCGCA	NZ_CP040429.1	N	N	N	N	N
104	GCTGCGGGCTTG<br>ACGACC	NZ_CP044092.1	N	N	N	N	N
105	GCTCTACAGAA<br>GAGCGAACAGC<br>AGCCGAGCATG<br>GCTCGGATCTAC<br>AGAAGAG	NZ_CP056088.1	N	N	N	N	N
106	GCTATCGGCCCT<br>CATCCGCGCATG<br>GCGTGGATCTAC<br>CGCAGCCT	NZ_CP040439.1	N	N	N	N	N
107	GCTACAGGGCC<br>TGAGCGCTGTTG<br>CTTTGGTAGCTG<br>CCCACCTTGGTG<br>GGCACCGGTG	NZ_CP067993.1	N	N	N	N	N
108	GCGTTCAGTAG	NZ_CP033877.1	N	N	N	N	N

	ATCCACGCCAG GCGTGGATGGG ATGCCGGCAGA AGCA						
109	GCGTGGCAGAC CGGGGTCTGGT GGCGGCCGACC TTGGTTGGCCCC CAAACCCAGC	NZ_CP033877.1	N	N	N	N	N
110	GCGGTACAGCG CTCTGGTAGATG CCCACCTTGGTG GGCGCCGATGC ATGGCA	NZ_CP040435.1	N	N	N	N	N
111	GCGGCCGGCAC TACCGAATCCAC GCGTGGTGGT	NZ_CP043578.1	N	N	N	N	N
112	GCGCCCGATCC GTGCTGTATCTC TCC	NZ_CP031058.1	N	N	N	N	N
113	GCGCCAGGAGG TTGCCGGCCAGC GGCCGGCACTA CCCGTAAATCG GGGGCCC	NZ_CP043578.1	NZ_OU943334.1-2, NZ_CP031058.1-2, NZ_CP104289.1-3, NZ_CP049956.1-9, NZ_CP088241.1-2, NZ_CP065965.1-6, NZ_CP051467.1-5, NZ_CP049368.1-3, NZ_CP044092.1-5, NZ_CP040438.1-2, NZ_CP040436.1-3, NZ_CP040435.1-5,	NZ_CP060026.1-3, NZ_CP040440.1-5, NZ_CP028358.1-3, NZ_CP101622.1-1, NZ_CP022053.2-3, NZ_CP022053.2-2 ,NZ_LR134324.1-,	N	N	100

			NZ_CP037858.1-2, NZ_CP008838.1-5, NZ_CP104288.1-4, NZ_CP101622.1-3, NZ_CP080573.1-5, NZ_LT906480.1-6, NZ_CP065965.1-9, NZ_CP014014.1-7, NC_010943.1-3	NZ_CP060026.1-1, NZ_CP060023.1-4, NZ_CP060022.1-2, NZ_CP040440.1-1, NZ_CP040434.1-1, NZ_CP025298.1-1, NC_017671.1-1, NZ_CP104323.1-1, NZ_CP040429.1-1, NZ_CP049368.1-1			
114	GCGCATGCCCA GTAGAGCCACG CCATGCGTGGAT GAATTTCAT	NZ_CP018756.1	N	N	N	N	N
115	GCGCACGCGGC TGCCGACCAGG CCGAT	NZ_CP040429.1	N	N	N	N	N
116	GCGATGTGAGC GTGCCAACCAG GGTTGGCACCTA CCGGGTTATGTG AGCGC	NZ_CP104323.1	N	N	N	N	N
117	GCCTGACTGAA ATGCCGGCCAG CGGCCGGCACT ACCCCTCGCCGC CTGACTGA	NZ_CP033829.1	N	N	N	N	N
118	GCCTCCTGACGC	NZ_CP101622.1	N	N	N	N	N

	CCTGCGCGGCG CGATGCCGGCC AGGGCCCCCGA TTTACGG						
119	GCCTCATTGAAA GGCAAACAAAA AAGCCCGGGTG TCTGCACACCGG GCTTCT	CP078102.1	N	N	N	N	N
120	GCCTCATTGAAA GGCAAACAAAA AAGCCCGGGTG TCTGCACACCCG GGCTTCT	NZ_CP037858.1	N	N	N	N	N
121	GCCTCATTGAAA GGCAAACAAAA AAAGCCCGGGT GTCTGCACACCC GGGCTACT	NZ_CP104286.1	N	N	N	N	N
122	GCCGCACTGGC CTTCGGCCACCT TGGCCTTGCCGG CGGGCTTGCTGC TGGCAGCCTTGT TGGCA	NZ_CP018756.1	N	N	N	N	N
123	GCCGAGGCCCG	NC_010943.1	N	N	N	N	N

	GCGGGGGCGGC<br>AGGCG						
124	GCCCCATCCACT<br>ATCGATACAGG<br>CCCCTGCCT	NZ_CP031058.1	N	N	N	N	N
125	GCCCCACGAAC<br>CCTGGTAGTGCC<br>GGTCGCTGGCC<br>GGGCAGCCCCA<br>CGAACCCT	NZ_CP101622.1	N	N	N	N	N
126	GCCCCACGAAC<br>CCTGGTAGTGCC<br>GGCCGCCGGCC<br>GCCGGCCGGGC<br>AGCCCCACGAA<br>CCT	NZ_CP104863.1	N	N	N	N	N
127	GCCCCAAACCC<br>AGCGCCGACCA<br>AGGTCGGCATCT<br>ACCGTAGCGTG	NZ_CP043578.1	N	N	N	N	N
128	GCCCAAGGTGG<br>CAGGTGAATCA<br>CCATG	NC_010943.1	N	N	N	N	N
129	GCCAACCAAGG<br>TTGGCCTCTACC<br>AGAGCCGGGCC	NZ_CP037858.1	N	N	N	N	N

130	ATGCATGTGAA CGCAGG GCCAACCAAGG TTGGCATCTACC AGAGCCGGGTC ACGCATGTACC GGCCGGTCAGG CA	NZ_CP067993.1	N	N	N	N	N
131	GCATCCACGCGT GGCCGCTGCATC GGGTAGGTGCA GGTCCTGGTCCG CGCGCG	NZ_CP104286.1	N	N	N	N	N
132	GCATCCGCGTGT GGCCGCTGCATC GGGTAGGTGCG GGCCCTGGTCCG CACGCG	NZ_CP014014.1	N	N	N	N	N
133	GCATCCACGCGT GGCCGCTGCATC GGGTAGGTGCG GGCCTTGGTCCG CACGCG	CP078102.1	N	N	N	N	N
134	GCAGTTGCCTGG AGGCCAGGGAA GCCACGCATGG	NZ_CP040438.1	N	N	N	N	N

	CGTGGCTCTACT GCCTCTCCCCTA G						
135	GCAGGACGTCA GGAGGTTGCCG GCCAGCGGCCG GCACTACCCGTA AATCGGG	NZ_CP080573.1	NZ_CP031058.1-2, NZ_OU943334.1-2, NZ_CP104289.1-3, NZ_CP049956.1-9, NZ_CP088241.1-2, NZ_CP065965.1-6, NZ_CP051467.1-5, NZ_CP049368.1-3, NZ_CP044092.1-5, NZ_CP040438.1-2, NZ_CP040436.1-3, NZ_CP040435.1-5, NZ_CP037858.1-2, NZ_CP008838.1-5, NZ_CP104288.1-4, NZ_CP101622.1-3, NZ_CP080573.1-5, NZ_LT906480.1-6, NZ_CP065965.1-9, NZ_CP014014.1-7, NC_010943.1-3	NZ_CP060026.1-3, NZ_CP040440.1-5, NZ_CP028358.1-3, NZ_CP101622.1-1, NZ_CP022053.2-3, NZ_CP022053.2-2, NZ_LR134324.1-2, NZ_CP060026.1-1, NZ_CP060023.1-4, NZ_CP060022.1-2, NZ_CP040440.1-1, NZ_CP040434.1-1, NZ_CP025298.1-1, NC_017671.1-1, NZ_CP104323.1-1, NZ_CP040429.1-1, NZ_CP049368.1-1	NZ_CP022053.2-8, NZ_CP060026.1-10, CP050452.1-2	N	100
136	GCACGGTGCTTT CCACTGCCATGA C	NZ_CP051467.1	N	N	N	N	N
137	GCACCCTCCACC CATCGCGCACC GT	NC_010943.1	N	N	N	N	N
138	GCACCCCAGCC CGCCGTTGGCGC	NZ_CP056088.1	N	N	N	N	N

	GCTGGGGTGCCCG					
139	GCAATGCGCCGGGGGCCGCCACACCTGCTCT	NZ_CP090423.1	N	N	N	N	N
140	GCAACCCGTTTTGTTGGGTGCTGACCTTGGTCGGCACGAATGCCTCCTGCACGCGCGACC	NZ_OU943334.1	N	N	N	N	N
141	GCAACCCGCTTTGGTGGGTGCCGACCGTTGGTCGGCACGGATGCCTGTGCCTGTCGATC	NZ_CP098483.1	N	N	N	N	N
142	GCAACCCGCTTTGGTGGGTGCCGACCGTTGGTCGGCACGGATGCCTGTGCCTCCCGATC	NZ_CP033829.1	N	N	N	N	N
143	GCAACCCGCTTTGGTGGGGGCCGACCGTTTGGTCG	NZ_CP040430.1	N	N	N	N	N

	GCACGGATGCC<br>TGTGCCTCTCGA<br>TC						
144	GCAACCCAGCG<br>CGCCAACGGCG<br>GGCTGGGTTGC	NZ_CP104288.1	N	N	N	N	N
145	GATTCCAGGCA<br>AAGAAAAAGCG<br>GGCCGAAGCCC<br>GCTTTTTCATCT<br>GCGAAAGCGTG	NZ_CP040432.1	N	N	N	N	N
146	GATTCCAGGCA<br>AAAAAAAAGCG<br>GGCCGAAGCCC<br>GCTTTTTCATCT<br>GCGAAAGCGTG	NC_011071.1	N	N	N	N	N
147	GATGCTTCTCGC<br>TGCCATGACCTG	NZ_CP060027.1	N	N	N	N	N
148	GATCGAGAGGC<br>ACAGGCATCCG<br>TGCCGACCAAC<br>GGTCGGCACCC<br>ACCAAAGCGGG<br>TTGC	NZ_CP052863.1	N	N	N	N	N
149	GATCCACCCACC<br>ACCAGCCGCAA	NZ_CP018756.1	N	N	N	N	N

	TCCGG						
150	GAGTCAGACCTT CCCGGTAGCGTC GGGCCGTGCCC GGCGAAGAGC	NZ_CP040432.1	N	N	N	N	N
151	GACTATGGATTG CCGGCCAGCGG CCGGCACTACC GGGGCCATGGA TTGT	NZ_CP104286.1	NZ_CP104288.1-4, NZ_CP040439.1-7, NZ_CP014014.1-7, NZ_CP104292.1-2	NZ_CP022053.2-3	N	N	100
152	GACCCTGTAGA GCCGAGCCATG CTCGGCTGTAAA ACGCAA	NZ_CP018756.1	NZ_CP040436.1-3, CP071784.1-1	N	N	N	100
153	GACCCCAATGC CCCGGTGGGTG CCAACCTTGGTT GTCACTCTTTAC TGCCCT	NZ_CP104286.1	N	N	N	N	N
154	GAATGAATTCAT CCACGCATGGC GTGGATCTACTG GCAACTGCGGC TGT	NZ_CP018756.1	NZ_CP049368.1-3, NZ_CP040432.1-2	N	NZ_OU943334.1-4	N	100
155	GAATCCATGCGT GGTGGTTGCATC	NZ_CP031058.1	N	N	N	N	N

	GGGTAGGTGCG GACCTTGGTCCG CACGC						
156	GAATCCACGTGT GGCGGCTACAT CGGGTAGGTGC GGACCCTGGTCC GCACGCG	NZ_LS483377.1	N	N	N	N	N
157	GAAGGCGGATG CGGTGGTAGAT CCACGCCATGC GTGGATGATTTT TCACGGGCGCC GAA	NZ_CP040438.1	N	N	N	N	N
158	GAAGCGTTGGT GGGTGCCAACC GTGGGTTGGCAT CGATGCTGCCCC TGGTAGCC	NZ_AP021687.1	N	N	N	N	N
159	GAAGCCAGCGC CAACCAAGGTT GGCGGCTACCG GGCCTGAGCGG CGTTGCTTT	NZ_CP040432.1	N	N	N	N	N
160	GAAGATCGCGG GATTGCCGGCC	NZ_LS483377.1	N	NZ_CP022053.2-3	N	N	100

	AGCGGCCGGCACTACCGAAGATCACGGGG						
161	GAACACCCGTGGCCAGGCTGGCGGCCTGGCAGCAGAG	NZ_CP008838.1	N	N	N	N	N
162	GAACACCCGTGGCCAGGCTGGCGGCCTGGCAGCAG	NZ_CP022053.2	N	N	N	N	N
163	GAAACCGCCGACCAAGGTCGGCTGCTACCAGAGCGTGCCATTGCCTCTTC	NZ_CP040438.1	N	N	N	N	N
164	GAAACCGCCGACCAAGGTCGGCCACTACCAGAGCGTGCCATTGCCTCTTC	NZ_CP067993.1	N	N	N	N	N
165	CTTGCCTTCGCCACACTTGCCTTCG	NZ_CP040439.1	N	N	N	N	N
166	CTGCTGCCAGGCCGCCAGCCTGG	NZ_CP098483.1	N	N	N	N	N

167	CCACGGGTGTTC CTCCGTGCCGAC CAAGGTCGGCA CCCACCAAGAG CGGCAA	NZ_CP015612.1	N	N	N	N	N
168	CTCATCCACGCA TGGCGTGAATCT ACTGACCTCTGG TGGGGGCTGAC CTTTGGTGGG	NZ_CP040437.1	N	N	N	N	N
169	CTATCGAAATGC CTGACGGGCTC GGCTTTGGTAG	NZ_CP011010.1	N	N	N	N	N
170	CTACTGACACCA TGATTCTGGTGG GTGCCAACCTTG GTTGGCACGCGT TGCGCGATAA	NZ_CP015612.1	N	N	N	N	N
171	CTACCGGATGG GGTGGTGGTAG CGCGGGACCGT GCCCCGCGAGC GCGCAGCGCGG TGT	NZ_CP018756.1	N	N	N	N	N
172	CGTTGAAACGA AGAACCCCCGG	NZ_CP040429.1	N	N	N	N	N

	TGAGAGCCGGG GGTTTTTTGTGG TGCCGATGATGC CT						
173	CGTGCGCGCAC GATCGGCGACC TTGAC	NZ_CP040429.1	N	N	N	N	N
174	AGATGCGGGAG CAACAGTCCTCG TTTGCGATGAGG GCT	NZ_AP021908.1	N	N	N	N	N
175	CGGTGCCGCTTC CTTCTTCACCG	NZ_CP090423.1	N	N	N	N	N
176	CGGTCGGCCCC ACCAAAGGTCA GAACCCACCAA AGGTCAGTACC CACCAGAGGTC	NZ_CP052863.1	N	N	N	N	N
177	CGGTCACGCCA CCCGGTAGTCGC CCACCTTGGTGG GCGCTGTTGACC TCCCGT	NZ_CP080573.1	N	N	N	N	N
178	CGGTAGATCCA CGCCGTGCGTG GATGGGAAA	NZ_LS483377.1	N	N	N	N	N

179	CGGGATGTCAG CCCTGGCGGAC GTCGACCTTGGT CGACGAACGAG GTGCCAGCCCT	NZ_CP037858.1	N	N	N	N	N
180	CGGCGTGTCCG ATCCCGGTTGTA GTTGCCCTAC	NZ_CP080573.1	N	N	N	N	N
181	CGGCGTGTCCG AGCCCGGTTGTA GTTGCCCTAC	NZ_CP029773.1	N	N	N	N	N
182	CGGCGTGGATCT ACTACCGGGCT GCGCCCTGGTG GGTGCCGACCG TTGGTCGGCACT GAC	NZ_CP018756.1	NZ_CP008838.1-5	N	N	N	100
183	CGGCAGATCCC GCATCGGTAAC GCCGGGCCATG CCCGGCGAGT	NZ_OU943334.1	N	N	N	N	N
184	CGGCAGAGTAA ACCGCCCCCGCC CGCCA	NZ_CP043578.1	N	N	N	N	N
185	CGGCAGAATCA GCCGTCGCCCGC	NZ_CP040430.1	N	N	N	N	N

	CA						
186	CGGATGCACGCCAACCAAGGTTGGCGACTACCGGGGCATGGCGCTCT	NZ_CP029773.1	N	N	N	N	N
187	CGCTGAACATTGAATGATGGTGCCGGCCGCTGGCCGGCATCACATCCAGGCT	NZ_CP104287.1	N	N	N	N	N
188	CGCGTGCGGACCAGGGCCCGCACCTACCCGATGTAGCCGCCACACGTGGATGG	NZ_CP033586.1	N	N	N	N	N
189	CGCGTGCGGACCAGGGCCCGCACCTACCCGATGCAGCGGCCACGCGTGGATGC	NZ_CP101622.1	N	N	N	N	N
190	CGCGCAGCGCGGGGTTTTCGGGGGGCTGAACGAAGAGCAGTCGACTGACAGTT	NZ_CP033586.1	N	N	N	N	N

191	CGATGGCACATCCACTGGTGGGTACCAACTGTTGGTTGGCACAGATGACTGCTTC	NC_015947.1	N	N	N	N	N
192	CGAGTGAGGAAACATCGTGTCGACCAAGGTCGACATCTACCGGGGGCTGGATAG	NZ_CP040438.1	N	N	N	N	N
193	CGACCGCCAACACACCCG	NZ_CP033877.1	N	N	N	N	100
194	CGAACACCTGCAGCCAGGCATGGCCTGGCTCT	NZ_CP014014.1	N	N	N	N	N
195	CCTGGTGGGTGCCGACCTTGGTCGGCACTGAGGCTGGCCCAGC	NZ_CM001824.1	N	N	N	N	N
196	CCTACCGGGTGCATGATGGCCTGCGGAACTGACGGACTGCTTTGGTG	NZ_CP056088.1	N	N	N	N	N
197	CCTACCGATGGTTTTTCGCGAGTGT	NZ_CP025298.1	N	N	N	N	N

198	CCGTACGGCAA<br>GCCGAAGGGTC<br>CGGGCGCA	NZ_CP011010.1	N	N	N	N	N
199	CCGTAAATCGG<br>GGGCTCTGGCC<br>GGCATCGCGCC<br>GTGCAGAGGTC<br>AGGAG	NZ_CP104286.1	N	N	N	N	N
200	CCGTAAATCGG<br>GGCCCTGGCCG<br>GCATCGCGCCG<br>CGCAGGGCGTC<br>AGGAGGC	CP078102.1	N	N	N	N	N
201	CCGGTAGCGCC<br>GGGCCATGCCC<br>GGCGGACAGTG<br>CCAACCAAGGT<br>GG	NC_011071.1	N	N	N	N	N
202	CCGGCGGCCGA<br>TGCCGCCGCCCC<br>GGCCACTACCG<br>AGCAGGCCGCC<br>A	NZ_CP043578.1	N	N	N	N	N
203	CCGGCGGCCGA<br>TGCCGCCGCCCC<br>GGCCACCACCG	NC_015947.1	N	N	N	N	N

204	AGCAGGCCGCCACCGGCCAGATCCACCGCAGAAGCATCCACGCATGGCATCGCCCGGGCACCCCGCA	NZ_CP025298.1	N	N	N	N	N
205	CCGGCAATGCCAACCAAGGTTGGCAGCTACCAGAGCCGG	NZ_CP043578.1	N	N	N	N	N
206	CCGCGGTACAGCGCTCTGGTAGATGCCCACCTTGGTGGGCGCCGATGCATGGCA	NZ_CP028358.1	N	N	N	N	N
207	CCGCGATGCATCGGTGCCCACCAAGGTGGGCAGCTACCAAAGCCGCGCCGCTCAAGCCC	NZ_CP031058.1	N	N	N	N	N
208	CCGAGCAGGAGCAGTGCCAGGCGAGCCTGGCACCCACCAGAACA	NZ_CP056088.1	N	N	N	N	N

	GTAGAA						
209	CCGAGCAGGAG CAGGAGCAGGA GCAGTGCCAGG CGATCCTGGCAC CCACCAGTAGA A	NZ_LR134301.1	N	N	N	N	N
210	CCGACACCTACC AAAGCGAATGT GCG	CP091781.1	N	N	N	N	N
211	CCCTCAGTGCCG TGGGTGCGGCC AGCCGCTGCTGC AGGTC	NZ_CP011010.1	N	N	N	N	N
212	CCCGGCGCTACC GCCCTGGTAGGT GCCAACCTTGA ACCC	NZ_CP040429.1	N	N	N	N	N
213	CCCGGCAAAGT CAACCGCCCCC GCCCGTCAGTA	NZ_CP104289.1	N	N	N	N	N
214	CCCGCTGTTGGT AGGTGCCGACC GTTGGTCGGCAC AATCTGTCGGCA CCC	NZ_CP104323.1	N	N	N	N	N

215	CCCCTGACGCCC TGCACGGCGCG ATGCCGGCCAG GGCCCCCGATTT ACGG	NZ_CP008838.1	NZ_LT906480.1-6, NZ_CP065965.1-9	N	N	N	100
216	CCCCATCCGATC AGGTCATGAAC CC	NZ_CP040430.1	N	N	N	N	N
217	CCCATGCATCGC GTCATCCACGCA TGGCGTGGATA CACCCACCA	NZ_CP043578.1	N	N	N	N	N
218	CCCATCCACGCA TGGCGGCTGCA CTTGGAAGGTG CGGACCTTGGTC CGCACGCC	NZ_CP011010.1	N	N	N	N	N
219	CCATGCGCAGTT CTGGTAGCGGC CGACCTTGGCCG GCGCGGTGAAT GCGTGTCGACC AG	NZ_CP104286.1	N	N	N	N	N
220	CCATCCGCGCGT GGTGGCTGCAG CGGGTAGGTGC	NZ_CP104289.1	N	N	N	N	N

221	GGTCCCTGGTCC GCACGCCT CCAGGTGCCGG CTCTGGTAGGCG TCGACCTTGGTC GCCGAAGGGCA TTTCAGCAG	NZ_CP014014.1	N	N	N	N	N
222	CCACGAGACAT GGTAGAGGCCG ACCTTGGTCGGC GCTTGCCGATTG T	NZ_CP067993.1	N	N	N	N	N
223	CCACCCACCAA CGCGGTAGTGC CGGCCGCTGGC CGGCAACCTCAT GCAT	NZ_CP040438.1	NZ_CP049956.1-6, NZ_LT906480.1-6, NZ_CP065965.1-9, NC_010943.1-3, NZ_CP088241.1-2, NZ_CP065965.1-6, NZ_CP051467.1-5, NZ_CP049368.1-3, NZ_CP044092.1-5, NZ_CP040436.1-3, NZ_CP040435.1-5, NZ_CP037858.1-2, NZ_CP014014.1-7, NZ_CP101622.1-3, NZ_CP080573.1-5, NZ_CP088243.1-4, NZ_CP088242.1-4, NZ_CP065965.1-7, NZ_CP040437.1-4, NZ_CP040432.1-2, NZ_CP104292.1-2	NZ_LT906480.1-1, NZ_LR134324.1-2, NZ_CP060026.1-1, NZ_CP060023.1-4, NZ_CP060022.1-2, NZ_CP040440.1-1, NZ_CP040434.1-1, NZ_CP025298.1-1, NC_017671.1-1, NZ_CP104323.1-1, NZ_CP040429.1-1, NZ_CP022053.2-2	NZ_CP060026.1-10	N	100
224	CCACACAGCCA TCCCGTCGCGGC GTTTCAGCTACG	NZ_CP033877.1	N	N	N	N	N

225	CCGCTTCGGT CATGTGCCTTGG TAGAGTCGACC GTTGGTCGACCA TGCGCCTT	NZ_CP018756.1	N	N	N	N	N
226	CATGGAACTCTC AGTCGCATCGAT GCTCGAGGGCT CCTGCTCCCGGT A	NZ_CP018756.1	N	N	N	N	N
227	CATGCATCCGTG CCAACCAAGGT TGGCACCTACCG AGCGATGGCGA ATC	NZ_CP104289.1	N	N	N	N	N
228	CATGCATCCGTG CCAACCAAGGT TGGCACCTACCA GGGCAGCGGCC AGTTGCA	NZ_CP049368.1	NZ_CP040439.1-7	N	N	N	100
229	CATGCATCAGTG CCAACCAAGGT TGGCACCCACC AGGCCCACC	NZ_CP027562.1	NZ_CP033829.1-3	NZ_CP052863.1-1, NZ_CP101622.1-1	N	N	100
230	CATGCATCAGTG CCAACCAAGGT	NZ_CP090418.1	NZ_CP033829.1-3	NZ_CP052863.1-1, NZ_CP101622.1-1	N	N	100

	TGGCACCCACC AGGCCCA						
231	CATCCCCGGCG GATCCCGCGAC CCGTCCA	NZ_CP040437.1	N	N	N	N	N
232	CATCCACGAAA CCGCGCCAGCA CCCGCAGCCGC CGCAACGACCG GCCC	NZ_CP090423.1	N	N	N	N	N
233	CATCCAATGGG GTCAGATNCGTT TTNTGCAGNAA AACGGATCTGA CCCCAAGAGCC GTTC	NZ_CP060022.1	N	N	N	N	N
234	CATCAGGCTCTG GTAGGTGCCGA CCTTGGTCGGCA CGCCATCAAGCT CTGGT	NZ_CP077679.1	N	N	N	N	N
235	CAGTAGATCCA CGCCATGCGTG GATGCAC	NZ_CP015612.1	N	N	N	N	N
236	CAGGCCGCCAG CCTGGCCACGG	NZ_CP104290.1	N	N	N	N	N

	GTGTTCTGGTAG GTG						
237	CAGGATCGCCG CCTCTGTAGAGT CGAGCCGT	NZ_AP021687.1	N	N	N	N	N
238	CAGCCCTCGTCG CAAGCGAGGAC TGTTGCTCCCGC ATCCTCGCTTGA TCCTGCTGCT	NZ_CP022053.2	N	N	N	N	N
239	CAGCAGCAATA CCAACCAGGTT GGCACCTACCA CACCAGCAATA	NZ_LR134301.1	N	N	N	N	N
240	CAGCACTACCG CAAGGCGTGCC GGTCAAAGCTG ACCCAC	NZ_CP040429.1	N	N	N	N	N
241	CAGAGCAGTCA GTGCCAACCAA GGTTGGCAACT ACCAGGGCAGT CAA	NZ_CP028899.1	N	N	N	N	N
242	CAGAAGGGGTA GTGCCGGCCGCT GGCCGGCAGTG	NZ_CP033586.1	NZ_CP031058.1-2	N	N	N	100

	TCGACCAAGGT CGACAC					
243	CACTTCCCCTGT CCTGGTTCT	NZ_CP104287.1	N	N	N	N	N
244	CACTCCGCTTTG ACTA	NZ_CP104287.1	N	N	N	N	N
245	CACGGCTGCCG GGCATGCGGTA GATCCACGCCAT GCGTGGATGCG CCGGGTCCGGA TACACCTGT	NZ_CP033877.1	NZ_CP037858.1-1	N	N	N	100
246	CACCTGACCTAA GAGCAGCCGAG CGTGGGCTCGG CTCTACAAGGA GCGCGGTCCCC GCCT	NZ_CP040432.1	NZ_CP104071.1-1	N	N	N	100
247	CACCGGGACGA ATGGGGCCTGG TAGAGGCCGAC CTTGGTCGGCGC TTGCCGATTGT	NZ_CP028899.1	N	N	N	N	N
248	CACCGGAGTTG ATGCCGGCCAA CGGTCGGCACCT	NZ_CP060026.1	N	N	N	N	N

249	GCCGAATTGAT CACCGCGCCGC CCGCGACGGCG GCCTGCACGTCG ATGGCGAGGAC GTGAACAT	NZ_CP018756.1	N	N	N	N	N
250	CACCCAATCGCC CACCAAGGTGG GCGACTACCAG AGCGATCCGAC GGCGGCCATGA AC	NZ_CP090423.1	N	N	N	N	N
251	CACCAGAGCCG GGCCACGCAGG TGAACGCAGGC GGGCGCAGGCG GGCGC	CP091781.1	N	N	N	N	N
252	CACCAACGTGG TAGTGCCGGCC GCTGGCCGGCA ACCTCATGCATC GC	NZ_CP037858.1	NZ_CP049956.1-6, NZ_CP088241.1-2, NZ_CP065965.1-6, NZ_CP051467.1-5, NZ_CP049368.1-3, NZ_CP044092.1-5, NZ_CP040438.1-2, NZ_CP040436.1-3, NZ_CP040435.1-5, NZ_CP037858.1-2, NZ_CP101622.1-3, NZ_CP080573.1-5, NZ_LT906480.1-6, NZ_CP065965.1-9, NZ_CP014014.1-7, NC_010943.1 -3,NZ_CP088241.1-5, NZ_CP049956.1-7,	NZ_CP022053.2-2	NZ_CP060026.1-10	N	100

			NZ_CP040439.1-7				
253	CACACACCCGG TAGCGCCGGGC CATGCCC	NZ_CP033877.1	N	N	N	N	N
254	CACAAACACCT ACAGCGTCAAA TGATCCGATCCA GA	NZ_CP018756.1	N	N	N	N	N
255	CAAATGCAGAA CGCGGGTACCG ACCGTTGGTCGG TACGCCTCAAA GC	NZ_CP018756.1	N	N	N	N	N
256	ATTGGGATCCGT GCGGACCAACG GTCCGCACCTAC CGACGCCGATG CCT	NZ_CP011010.1	N	N	N	N	N
257	ATTCAACGCGC GCGGACACGCC CGTGCCAACCG ATGGGTCCAGG	NZ_CP018756.1	N	N	N	N	N
258	ACGGCGCCAAC CAAGGTTGGCG ACTACCGCGGT ACAGCGCTCT	NZ_CM001824.1	N	N	N	N	N

259	ATGGGCGTGGTTCATGACCCGATCGGA	NZ_CP051467.1	N	N	N	N	N
260	ATGGCATCGTCCAGCGATCCGAACC	NZ_AP021687.1	N	N	N	N	N
261	ATGGATCCAGGACGGCGCCAACCAAGGTTGGCGACTATCGCGGTACAGCGCTCT	NZ_LS483377.1	N	N	N	N	N
262	ATGGATCCAGGACGGCGCCAACCAAGGTTGGCAACTACCGGGGTACAGCGC	NZ_CP040438.1	N	N	N	N	N
263	ATGCTGGTGGTGGCATTCATTGGCAGCGCCGGGCCGTGCTT	NZ_CP015612.1	N	N	N	N	N
264	ATGCTGCTCTGCCGGCCAGCGGCCGGCACACTACCGCTGCCCTGACCCATGGCTCC	NZ_CP049368.1	N	N	N	N	N
265	ATGCGGGATCT	NZ_CP056088.1	N	N	N	N	N

	GACTTGGTGGGTGCGAACCTTGTTTCGCACCGTTCTTCCCGCCGCAAAC						
266	ATGCCGGGATTGACGGTAGAGCGCCAACCAAGGTTGGCATCTACCAAGTC	NZ_CP011010.1	N	N	N	N	N
267	ATGCACCACGGCGCCAACCAAGGTTGGCGACTACCGGGCCTGAGCGGCGTTGCTTT	NZ_CP080573.1	N	N	N	N	N
268	ATGATCCGCGGGCGCACCGTGCATCGCCTCGGCACGCCCACCGATGCG	NZ_CP040430.1	N	N	N	N	N
269	ATGAATTCATCCACGCATGGCGTGGATCTACCGTTCGCCG	NZ_CP018756.1	N	N	N	N	N
270	ATGAATCTGCCCTGGTGGGTGCC	NZ_CP028358.1	N	N	N	N	N

	GACCTTGGTCGGCACATGAATCTGCC						
271	ATCGGCGCGTGGTGGCCGCACTTGGTAGGTGCGGACCTTGGTCCGCACGCT	NZ_CP040437.1	N	N	N	N	N
272	ATCGCGGAAAAGATCGGATGGACAAC	NZ_CP040429.1	N	N	N	N	N
273	ATCCGATCGGGTCATGAACCACGCCCAT	NZ_CM001824.1	N	N	N	N	N
274	ATAGGAATTCACCTTGAACTGCCTGGGTG	NZ_CP015612.1	N	N	N	N	N
275	AGTTCCGCAGGCCATCATGCACCCGGTAGGTGTCGACCTTGGTCGACACG	NZ_CP015612.1	N	N	N	N	N
276	AGTCGACCGTTGGTCGACTGCCCTTCCATCCG	NZ_CP018756.1	N	N	N	N	N
277	AGTAGATCCAC	NZ_CP018756.1	NZ_CP049956.1-6,NZ_CP044092.1-6,NZ_CP0	N	N	N	100

	GCCATGCGTGG ATAAGACCCGG CACAGTGCGGA CCAACGGTCCG CACCCACCA		40439.1-7				
278	AGGTTCGTGGG GCTGCCCGGC	NZ_CP104288.1	N	N	N	N	N
279	AGGGTTCGTGG GGTTGCCGGCC AGCGGCCGGCA TTACCAGGGTTC GTGGGG	NZ_CP040436.1	N	N	N	N	N
280	AGGGTTCGTGG GGCTGCCCGGC CGGCACTACCA GGGTTCGTGGG GC	NZ_CP014014.1	N	N	N	N	N
281	AGGGTTAAGGC GGTATGCGCGG GCAAAACCGCA GAGCGGTTTGCT	NZ_CP028358.1	N	N	N	N	N
282	AGGGTCCACGC GGTTGCCGGCC AACGGCCGGCA CTACCAGGGTCC ATGCGA	NZ_CP080573.1	N	NZ_CP033877.1-1	N	N	100

283	AGGGCTGCGTC GTCGAAGGCGC GAGGCCCGACG GGCTCGTCGCA GACGAGGACGG C	NZ_CP040429.1	N	N	N	N	N
284	AGGGCCAATCG ACGCCATGATCC GCT	NZ_CP060027.1	N	N	N	N	N
285	AGGGCAGTAAA GAGTGCCAACC AAGGTTGGCAC CCACCGGGGCA TTGGGGTC	NZ_CP033586.1	N	N	N	N	N
286	AGGCATGAGGC CTGTATCGATCC TGGGCGGCC	NZ_CP033877.1	N	N	N	N	N
287	AGGCAGGGGCC TGTATCGATCGT GGATGGGGCT	NZ_CP015612.1	N	N	N	N	N
288	AGGCAGGGGCC TGTATCGATAGT GGATGGGGC	NZ_CP067993.1	N	N	N	N	N
289	AGGCAGGGGCC TGTATCGATAAT GGATGG	NZ_CP043578.1	N	N	N	N	N

290	AGGAGGGTGGT GACACCCATTG GCAGCGCCAGG CCATGGCT	NZ_LS483377.1	N	N	N	N	N
291	AGCGGCCGGCA CTACCCCCATCC ACGCATGGTAG G	NZ_CP029773.1	N	N	N	N	N
292	AGCGGCAGGAT CAAGCGAGGAT GCGGGAGCAAC AGTCCTCGCTTG CGACGAGGGCT	NZ_CP104292.1	N	N	N	N	N
293	AGCGAACACCT GCAGCCAGGCA TGGCCTGGCTCT ACGACGGTTGC CTGGGG	NZ_CP033877.1	N	N	N	N	N
294	AGCCCTCGTCGC AAACGAGGTCT GTTGCCCCCGCA TCCTCCCCTGAT CCTGCT	CP071784.1	N	N	N	N	N
295	AGCCCTCGTCGC AAACGAGGACT GTTGCCCCCGCA	NZ_CP060027.1	NZ_LT906480.1-6, NZ_CP065965.1-9, NZ_CP014014.1-7, NC_010943.1-3, NZ_CP088241.1-5, NZ_CP065965.1-7,	NZ_LT906480.1-1	N	N	100

	TCCTCGCTTGAT CCTGCTGCCCCG GTAGTGCCGGC CGCTGGCCGGC AACAGCCCTCGT CGCAAGCGAGG ACTGTTGCT		NZ_CP049956.1-7, NZ_CP049956.1-6, NZ_CP049368.1-3, NZ_CP040439.1-7, NZ_CP104292.1-2				
296	AGCCATGGCCT GGCGCTGCCAA TGGGTGTCACCA CCCTCCTGGTGG GT	NZ_CP088241.1	N	N	N	N	N
297	AGCCATGGCCT GGCGCTGCCAA TAGGTGTCACCA CCCTCCT	NZ_CP060021.1	N	N	N	N	N
298	AGCAGGATCAG GGGAGGATGCG GGGGCAACAGA CCTCGTTTGCGA CGAGGGCT	NZ_CP040436.1	N	N	N	N	N
299	AGCAGCAGGAT CAGGGGAGGAT GCGGGGGCAAC AGACCTCGTTTG CGACGAGGGCT	NZ_CP104287.1	N	N	N	N	N

300	AGCACTACCCCT<br>TCTGCCAGGCCG<br>CCAGCCTGGCC<br>ACGGGTGTTCTG<br>GTAGGC	NZ_CP049956.1	N	N	N	N	N
301	AGCAACAGTTCT<br>CGTTTGCGATGA<br>GGGCTGTTGCCG<br>GCCAGCGGCCG<br>GCACTACCGGG<br>GCAGCAGGATC<br>AAGCGAGGATG<br>CGGGGGCAACA<br>GTCCTCGTTTGC<br>GACGAGGGCT	NZ_CP104286.1	NZ_CP014014.1-7, NZ_LT906480.1-6, NZ_CP065965.1-9, NC_010943.1-3, NZ_CP088241.1-5, NZ_CP065965.1-7, NZ_CP049956.1-7, NZ_CP049956.1-6, NZ_CP049368.1-3, NZ_CP040439.1-7, NZ_CP104292.1-2	NZ_LT906480.1-1	N	N	100
302	AGATCGCGGGG<br>TTGCCGGCCGGT<br>GGCCGGCACTA<br>CCGTAGATCGC<br>GGGGTTA	NC_015947.1	N	N	N	N	N
303	AGATACAGGCA<br>TCTGCTCTGGTA<br>GGTGTCGACCTT<br>GGTCGACACGA<br>ATGCC	NZ_CP033877.1	NC_010943.1 -4	N	N	N	100
304	AGAGCTTTGCGT	NZ_CP049368.1	N	N	N	N	N

	CGTCGAAGGCT<br>CGGGGGCAGAC<br>GGGTTCGTTGCA<br>GAGGC						
305	AGAGCGTGCCA<br>TTGCCTCTTCGG<br>TAGGTGCCAAC<br>CTTGGTTGGCAC<br>AGAAG	NZ_CP031058.1	NC_010943.1 -4	N	N	N	100
306	AGAGCAACGCC<br>GCTCAGGCCTG<br>GTAGCCGCCAA<br>CCTTGGTTGGCG<br>CTGGCTTCCAT	NZ_CP052863.1	N	N	N	N	N
307	AGAATGGCCGG<br>TCAGCCGAGCTT<br>GGCTCGGCTCTA<br>CAGAATGGCCG<br>GTC	CP050452.1	N	N	N	N	N
308	AGAACCGCCGA<br>CCAAGGTCGGC<br>GACTACCGGAG<br>CGTGCCATTGCC<br>TCTCC	NZ_CP033877.1	N	N	N	N	N
309	AGAAAGCGCAG<br>GCCACGGCGCA	NZ_CP028358.1	NZ_CP040432.1-2	N	N	N	100

	TCCACGCATGGC<br>GTGGATCTACTG<br>GCCGCGAA						
310	ACTGGCTTGGCG<br>GCAGCCTTCTTC<br>ACCGGCGCA	NZ_CP044092.1	N	N	N	N	N
311	ACTACCATCAG<br>ATGCAACCATTC<br>ATCGCCTGAGC<br>GTCGCGGAACT	NZ_CP029773.1	N	N	N	N	N
312	ACGTCTGGGCTG<br>CCCTACACAA	NZ_CP101622.1	N	N	N	N	N
313	ACGGTAGTGCC<br>GGCCGCTGGCC<br>GGCAACTTCATG<br>ATCCGGGTGCG<br>GATCTGCT	NC_015947.1	NZ_CP088243.1-4, NZ_CP088242.1-4,<br>NZ_CP088241.1-2, NZ_CP065965.1-7,<br>NZ_CP065965.1-6, NZ_CP044092.1-5,<br>NZ_CP040437.1-4, NZ_CP040432.1-2,<br>NZ_CP101622.1-3	NZ_LT906480.1-1,<br>NZ_CP060026.1-1,<br>NZ_CP040440.1-1, NZ_CP022053.2-2	NZ_CP060026.1-10	N	100
314	ACGGCGCCGGC<br>TCTGGTGGGTGC<br>CGACCGTTGGTC<br>GGCACTGATGC<br>CTGC	NZ_AP021687.1	N	N	N	N	N
315	ACGGCGCCGGC<br>TCTGGTGGGTGC<br>CGACCGTTGGTC<br>GGCACTGATGC	NZ_CP040432.1	N	N	N	N	N

	CT						
316	ACGGACCGCCA ACCAAGGTTGG CGGCAACCGGG CAGCGGGCAGC GGACCT	NZ_CP040438.1	N	N	N	N	N
317	ACGCGGAGCGA TACGACCCAGG TCGACACCTACC TGAGCGGGGCG AT	NZ_AP021687.1	N	N	N	N	N
318	ACCTTGGTCCGC ACGCTTTTGCCG GCCAGCGGCCG GCACTACCGG	NZ_LR134324.1	NZ_CP040439.1-7, NC_010943.1 -3	N	N	N	100
319	ACCTCAGTCGA GCAAGCTCGAC TCTACGCCTACC TCG	NZ_CP011010.1	N	N	N	N	N
320	ACCGTGTCGACC AAGGTCGACAC CTACCCAGACG GAGACACCTCG GCATTGCT	NZ_CP033829.1	N	N	N	N	N
321	ACCGTCGGTAG AGTCGACTGTTA	NZ_CP104071.1	NC_010943.1 -4	N	N	N	100

	GTCGACTGCTCT TCCTTCAACGC ACCGGGGCGGT TTCCATGCGGAC						
322	CAACGGTCCGC ACCTACCGGGG CGGTTTC	NZ_CP018756.1	N	N	N	N	N
323	ACCGGCGCCTTC TTGCTGGCAGCG GCAGGCTTGGC CTTG	NZ_CP044092.1	N	N	N	N	N
324	ACCGGAGCTTG AAGGCGTGCCG ACCAAGGTCGG CACCTACCGGG CTTGATG	NZ_CP056088.1	N	N	N	N	N
325	ACCGCCTTCGGC GCC	NZ_CP044092.1	N	N	N	NC_008296.2	100
326	ACCGCCGACCA AGGTCGGCCAC TACCAGAGCGT GCCATTGCCTCT TCGGT	NZ_OU943334.1	N	N	N	N	N
327	ACCCGTCCGCTC TGGTAGTGCCG GCCGCTGGCCG	NZ_CP104863.1	NZ_CP040439.1-7, NZ_CP088243.1-4, NZ_CP088242.1-4, NZ_CP088241.1-5, NZ_CP049956.1-7, NZ_LT906480.1-6,	N	CP050452.1-2	N	100

	GCAACCCATCC GCTC		NZ_CP065965.1-9, NZ_CP065965.1-7, NZ_CP049956.1-6, NZ_CP049368.1-3, NZ_CP040437.1-4, NZ_CP040432.1-2, NC_010943.1-3, NZ_CP104292.1-2				
328	ACCCAGAAGAT CAGCGCCAAGG ACATGGCCGAC CTCGG	NZ_CP018756.1	N	N	N	N	N
329	ACCATCCAATCC GCTAGGTACCG NCCGTTGGTCGG TACGGAACCAA CCAATCCGG	NZ_CP060027.1	N	N	N	N	N
330	ACCAGGGCAAA GACCCGCTGCA GTAGATACACG CCATGCGTGGAT GCATGCGT	NZ_CP028899.1	N	N	N	N	N
331	ACCAGGAGATG TGAGCGTGCCA ACCAGGGTTGG CACCTACCGGG AGATGTGT	NZ_CP104290.1	N	N	N	N	N
332	ACATGCTTCAGT GCCAACCAAGG TTGGCACCTACC	NZ_CP033877.1	NZ_CP049956.1-6, NZ_CP044092.1-6	N	N	N	100

	AGGGCAACGAC CC					
333	ACAGGTTGCCTG GGAGGTAGTGC CGGCCGCTGGC CGGCAATCTCGC GAA	NC_015947.1	N	N	N	N	N
334	ACACAGGGCCA ATCGACGCCAT GAT	NZ_CP051467.1	N	N	N	N	N
335	ACAATCGGCAA GCGCCGACCAA GGTCGGCCTCTA CCAGGCCCCATT CGCCCCGGC	NZ_CP060022.1	N	N	N	N	N
336	ACAACACGGTA GTGCCGGCCGCT GGCCGGCAACT TCGTGATCCGGG TGCGGATCTGCT	NC_017671.1	NZ_CP049956.1-6, NZ_CP088241.1-2, NZ_CP065965.1-6, NZ_CP044092.1-5, NZ_CP040437.1-4, NZ_CP101622.1-3, NZ_CP088243.1-4, NZ_CP088242.1-4, NZ_CP065965.1-7	NZ_CP060026.1-1, NZ_CP040440.1-1, NZ_CP022053.2-2	NZ_CP060026.1-10	N	100
337	AATGTGTCAGCC GCGCGCGCCCA AACGGTCGCGC AGGCACCA	NZ_CP052863.1	N	N	N	N	N
338	AATGGCGGCGG ACGCGGTACGG	NZ_CP043578.1	N	N	N	N	N

339	GCGGCCGGGGC TGCG AATCCACGCGT GGCGTGGATCT ACTGGACGACT C	NZ_CP040437.1	N	N	N	N	N
340	AAGTCAGATCC CGCATCGGTAG CGTCGGGCCAT GCCCGGCAAGC GCAGCGGCACG AG	NZ_CP028358.1	N	N	N	N	N
341	AAGGTTCATCCA CGCATGGCGCG GCACTGCATCCA CGCATGGCGTG GATCTACTG	NZ_CP018756.1	N	N	N	N	N
342	AAGGTCGACAC CTACCGAGGGG CCTAGCGCGCA GCGGTCGCCAT GCGCGGCCCT	NZ_CP037858.1	N	N	N	N	N
343	AAGGGGTAGTG CTGGCCGCTGGT CGGCAGTGTCG ACCAAGGTCGA	NZ_CP088241.1	N	N	N	N	N

	CA						
344	AAGGCCTCCCG CCCCGGTAGATC CACGCCATGCGT GGATGAAGGCA TTC	NZ_CP011010.1	N	N	N	N	N
345	AAGGCAGGCTT CGGACACATTC GTGCCAACCAA	NZ_CP025298.1	N	N	N	N	N
346	AACCTTGGTTGG CACATTCCAACC GTGCNGNGGAG	NZ_CP060027.1	N	N	N	N	N
347	AACCTCGCGAA CTCGGCTATGCT C	NZ_CP088244.1	N	N	N	N	N
348	AACCATGGTAG TGCCGGCCGCTG GCCGGCGCGCG CCGGGCATGGC	CP050452.1	N	N	N	N	N
349	AACAGGCCGGG CTGGCTCCTGGT GGGTGCC	NZ_CP018756.1	N	N	N	N	N
350	AACAGCGCAGA GCAGCCGACCG GTAGAGTCGAC CGTTGGTCGACT	NZ_CP018756.1	N	N	N	N	N

	GGCGAGAT						
351	AAACGCGCGGA CGGCCCGGCGC CAACCAAGGCG GCATCTACCCTG CACGGCATC	NZ_CP040429.1	N	N	N	N	N
352	AAAAGGGATTC ACCCTGAACTGT CTGGGTG	NZ_AP021687.1	N	N	N	N	N
353	TGTTTTCCCGCA GCGGCCCACGC GCC	NZ_CP060259.1	N	N	N	N	N
354	AAAGCGGAGGC GCATTGGTGCGC TCTGGTAGGTGC CAACCAG	NZ_CP040434.1	N	N	N	N	N
355	AAAGACCAGCT GCTGTAGATCCA CGCCATGCGTG GATGCGTGCC	NZ_CP014014.1	N	N	N	N	N
356	TCTCCACCCGCG CCGGTAGGTGC CAACCTTGGTTG GCACGCTCGATC CGCCCC	NZ_CP104323.1	N	NZ_CP031058.1-1	N	N	100
357	TCTCCACCCGCG	NZ_CP028899.1	N	N	N	N	N

	CCGGTAGCTGCC AACCTTGGTTGG CACGCTCGATCC GCCCC						
358	GAAACCGCCGA CCAAGGTCGGC TACTACCAGAG CGTGCCATTGCC TCTTC	NZ_CP040439.1	N	N	N	N	N
359	TCTTTCCAGAAG TTTCTGCAGCCG CCTTGATCTGTC TTT	NZ_CP028899.1	N	N	N	N	N
360	GCAACCCGCTTT GGTGGGGGCCG ACCGTTGGTCGG CACGGATGCCT GTGCCTGTCGAT C	NZ_CP104286.1	N	N	N	N	N
361	AGAGCGCTGTA CCGCGGTAGTC GCCAACCTTGGT TGGCGCCGTCCT GGACCCAT	NZ_CP051467.1	N	N	N	N	N
362	ACTGGAAAGGG CCACGCATGGA	NZ_CP033877.1	N	N	N	N	N

	TGTGCGTGACCT CGTCATCGGCCA GGCCTCATG						
363	ACTGGAAAGGG CCACGCATGGA TATGCGTGACCT CGTCATCGGCCT GGCCTCATG	NZ_CP025298.1	N	N	N	N	N
364	ACAGAGCGCGA CGGGGTCGTTGT GG	NZ_CP104289.1	N	N	N	N	N
365	ACAGAGCGCCA CGGGGTCGTTGT GG	NZ_CP088244.1	N	N	N	N	N
366	AAAGGCATTTAT CAACAAAGGGC CCGGTCACGAT GGCTTGGTCGGT CGCCCCAAGCTT G	NC_015947.1	N	N	N	N	N
367	AAAAGGGATTC ACCCTGAACTGT CTG	NC_011071.1,NZ_ CP040432.1	N	N	N	N	N
368	TTTCCCATCCAC GCATGGCGCGG ATCTACCG	NZ_CP022053.2,N Z_CP060026.1,NZ _CP060027.1,NZ_	N	N	N	N	N

369	TTGTGTAGGGCA GCCCAGACGT	CP101622.1,NZ_CP088241.1,NZ_CP104863.1 NZ_LS483377.1,CP078102.1,NZ_CP088240.1	N	N	N	N	N
370	TTCTGATCTGCC GGCCAGCGGCC GGCACTACTGGT TCCTGTGGCCTG GCG	NZ_CP040439.1,NZ_CP067993.1	N	N	N	N	N
371	TTCTACTGTTCT GGTGGGTGCCA GGCTCGCCTGGC ACTGCTCCTGCT CGG	NZ_CP040435.1,NZ_CP028358.1	N	N	N	N	N
372	TTCTACTGTTCT GGTGGGTGCCA AGCTTGCTTGGC ACTGGGTCTGG GTCTGCTCTG	NZ_CP015612.1,NZ_CP040438.1,NZ_CP043578.1	N	N	N	N	N
373	TTCTACAGGTAG GTGCCAACCTTG GTTGGCACGGA TGTGTCAGCGC	NZ_CP040439.1,NZ_CP040438.1	N	N	N	N	N
374	TTCCGCCGGGCA	NZ_CP022053.2,N	NZ_CP049368.1-2	N	N	N	100

	TGGCCCGGCGCTACCGGTAGCTGCTGACCTCTTCCCG	Z_CP051467.1,NZ_CP008838.1					
375	TTCCACTGTCCTTGTTCTGGTAGGTGCCAAGCTTGCTTGGCACGGGGTCTGGGTCTGCTC	NZ_LR134324.1,NC_017671.1,NZ_CP040434.1	N	N	N	N	N
376	TTCCACTGTCCTCGTTCTGGTAGGTACCAAGCTTGCTTGGCACGGGGTCTGCTCTG	NZ_CP040440.1,NZ_CP060026.1,NZ_CP102942.1,NC_010943.1,NZ_CP104288.1,CP078102.1,NZ_CP088240.1	N	N	N	N	N
377	TGGGCCAGTGAACGCCACGGTAGTG	NZ_CP101622.1,NZ_CP033586.1,NZ_CP104863.1	N	N	N	N	N
378	TGGAAATCCATCAGTAGAGCCAGGCCATGCCTGGCTGACGCCATATCGATCCACCAACAG	CP091781.1,NZ_CP033829.1	N	N	N	N	N
379	TGAGCTTGCTGT	NZ_CP027562.1,N	N	N	N	N	N

	CGCCACTGCGG CCCGCC	Z_CP090418.1					
380	TGACCTCTGGTG GGGGCTGACCTT TGGTGGGTGCC GGCCTTGGTCGG CACCGTCTC	NZ_CP088242.1,NZ_CP088243.1	N	N	N	N	N
381	TCTTTCCAGAAG TTTCTGCAGCCG CCTTGACCTGTC TTT	NZ_CP104323.1,NZ_CP040434.1	N	N	N	N	N
382	TCTTTACTGCGT TGGTGGGTGCC AACCTTGGTTGG CACTCTTTACTG CCCT	NZ_CP098483.1,NZ_AP021908.1,NZ_CP040440.1,NZ_CP060026.1,NZ_CP102942.1,NZ_CM001824.1,NZ_CP104290.1,NZ_CP104288.1	N	N	N	N	N
383	TCTCCACCCGCG CCGGTAGGTGC CAACCTTGGCTG GCACGCTCGATC CGCCCC	NZ_CP060022.1,NZ_CP060023.1,NC_017671.1,NZ_CP040434.1	N	N	N	N	N
384	TCGCCGGCGATT GCACACCGCAT	NZ_CP029773.1,NZ_CP080573.1	NZ_CP037858.1-1	N	N	N	100

	CCACGCATGGC GTGGATCTACCG T						
385	TCGAATCGGCC AGTAGATCCAC GCCACGCG	NZ_CP051467.1,NZ_CP033586.1	N	N	N	N	N
386	TCAGGGTGAGT GGGGCCTGGTA GAGGCCGACCT TGGTCGGCGCTT GCCGATTGC	NZ_CP104292.1,NZ_CP040431.1,NZ_CP040440.1,NZ_CP060026.1,NZ_CP088240.1,NZ_CP040430.1	N	N	N	N	N
387	TACGGAGCCAA CCGCATCCACGC ATGGCGTGGAT CTACCGACCGC AATG	NC_011071.1,NZ_CP040432.1,NZ_AP021687.1	N	N	N	N	N
388	GTTTTGCCCCGA AATCTGGCT	NZ_CP102942.1,NZ_CP104288.1	N	N	N	N	N
389	GTGCGCGTCCCC ATGATGGGCAT CAGCATGGTGC GCGTCAGCGGC GTGGTGA	NZ_CP027562.1,NZ_CP090418.1	N	N	N	N	N
390	GTGATGCCAGG TGCGGCGTTGCC	NZ_CP088242.1,NZ_CP088243.1	N	NZ_LT906480.1-1	N	N	100

	GGCCAGCGGCCGGCACTACCGAGATGCCAGGTACA						
391	GTGATCCTGGGATGCCGGCCAGCGGCCGGCACTACGTGATCCTGGGA	NZ_CP040439.1,NZ_CP067993.1	N	N	N	N	N
392	GTCATGGCAGTGGAAAGCACCGTGC	NZ_CM001824.1, NZ_CP040430.1	N	N	N	N	N
393	GTCAGAACACGCTGTCTTTGAAACTGGTTTCCAACTTTCCCGGGCAGCGACAGAT	NZ_CP090418.1,NZ_CP008838.1,NZ_CP065965.1,NZ_LT906480.1	N	NZ_LT906480.1-2, NZ_CP008838.1-2, NZ_CP028358.1-1	NZ_CP065965.1-10, NZ_CP090418.1-5	N	100
394	GTAGATCCACGCCATGCGTGGATGTTCTTCCGAATTCGCT	NZ_CP047310.1,NZ_CP060259.1	NZ_CP104071.1-1	N	N	N	100
395	GGTTGATCTGTCCTTATCCGATCC	NC_011071.1,NZ_CP040432.1,NZ_AP021687.1	N	N	N	N	N
396	GGTGGCAGGTGAATCACCATCCG	NZ_CP060022.1,NC_017671.1,NZ_C	N	N	N	N	N

	GTA	P104323.1,NZ_CP028899.1					
397	GGTCGCCAAGCCCGCAGC	NZ_CP033586.1,NZ_CP088241.1,NZ_CP052863.1	N	N	N	N	N
398	GGGTGGGTGCCGACCGTTGGTCGGCACGATCCTTCCGGGCCATGAACCCCGGTATCGTC	NZ_CP025298.1,NC_015947.1	CP078102.1-3	N	N	N	100
399	GGGTGCCGACAGATTGTGCCGACCAACGGTCGGCACCCACCAGGAGCGGG	NZ_CP060022.1,NZ_CP060023.1	NZ_CP049368.1-2, NZ_CP049956.1-6, NZ_CP044092.1-6, NZ_CP040439.1-7	N	N	N	100
400	GGGCATGTTCCAGTAGATCCACGCCATGCGTGGATGGCGGTGAA	NZ_CP040435.1,NZ_CP028358.1	NZ_CP014014.1-7	N	N	N	100
401	GGGCAGGTCTGACTCTGGTGGGTGCGAACCG	NZ_CP088241.1,NZ_CP104863.1	N	N	N	N	N
402	GGCGCGCAGCGGTTGCCATGCGCGGCTTCGGTGGC	NZ_CP008838.1,NZ_CP065965.1,NZ_LT906480.1	N	N		N	N

	GGCCGACCTTG GTCGGCGCCAT G						
403	GGCGCCGAAGG CGGT	NZ_CP033586.1,NZ_CP088241.1,NZ_CP052863.1	N	N	N	NC_008296.2	100
404	GGAGCGGATGG GTTGCCGGCCA GCGGCCGGCAC TACCAGAGCGG ACGGGT	NZ_CP008838.1,NZ_CP040430.1,NZ_CP065965.1,NZ_LT906480.1	NZ_CP040439.1-7, NZ_CP088243.1-4, NZ_CP088242.1-4, NZ_CP088241.1-5, NZ_CP049956.1-7, NZ_LT906480.1-6, NZ_CP065965.1-9, NZ_CP065965.1-7, NZ_CP049956.1-6, NZ_CP049368.1-3, NZ_CP040437.1-4, NZ_CP040432.1-2, NC_010943.1-3, NZ_CP104292.1-2	N	CP050452.1-2	N	100
405	GCTTCCCGTAGG TGCCGACCTTGG TCGGCACGCCC AGCTTCCCGTAG GT	NZ_LR134324.1,NZ_CP040434.1	N	N	N	N	N
406	GCTACCGGTCG GGTGGGTGCCG ACTGTTGGTCGG CACATCCCATTC CACGATCTACCT CCGATCTGGTGG GTGCCAACC	NZ_CP033586.1, NZ_CP088241.1	N	N	N	N	N
407	GCCTCATTGAAA	CP091781.1,	N	N	N	N	N

	GGCAAACAGAA AAGCCCGGGTG TCTGCACACCCG GGCTTC	NZ_LS483377.1, NZ_CP033829.1, NZ_CP052863.1					
408	GCCTCATTGAAA GGCAAACAAAA AAAGCCCGGGT GTCTGCACACCC GGGCTTCT	NZ_CP098483.1, NZ_AP021908.1, NZ_CP040431.1, NZ_CP040440.1, NZ_CP060026.1, NZ_CP102942.1, NC_010943.1,NZ_ CM001824.1, NZ_CP060027.1, NZ_CP088240.1, NZ_CP044092.1	N	N	N	N	N
409	GCCGGTTGGAT ACGCGGGAATG GCCCCATCCACG CATGGCGTGGA TCTACTGAACCC C	NZ_CP027562.1,N Z_CP090418.1	N	N	N	N	N
410	GCCGGGGCGAA TGGGGCCTGGT AGAGGCCGACC TTGGTCGGCGCT TGCCGATTGT	NZ_CP060023.1, NC_017671.1	N	N	N	N	N

411	GCCCAAGGTGG CAGGTGAATCA CCACCC	NZ_CP098483.1, CP078102.1, NZ_CP088240.1	N	N	N	N	N
412	GCCAACCAAGG TTGGCATCCACC AGAGCCGGGCC ATGCATGTGAA CGCAGG	NC_010943.1, NZ_CP049956.1	N	N	N	N	N
413	GCATCCACGCGT GGCCGCTGCATC GGGTAGGTGCG GGCCCTGGTCCG CGCGCG	NZ_CP104288.1, NZ_CP033829.1, NZ_CP052863.1	N	N	N	N	N
414	GCATCCACGCGT GGCCGCTGCATC GGGTAGGTGCG GGCCCTGGTCCG CACGCG	NZ_CP102942.1,N Z_CP088240.1	N	N	N	N	N
415	GCATCATCGCA GCGGGCGAGCA CCA	NZ_CP065965.1, NZ_LT906480.1	N	N	N	N	N
416	GCAGAAGCCCG TATCGACCGTGG ATGG	NZ_CP104292.1, NZ_CP049956.1	N	N	N	N	N
417	GCAATCGGCAA GCGCCGACCAA	NZ_CP060021.1, NZ_CP022053.2,	N	N	N	N	N

	GGTCGGCCTCTA CCAGGCCCCACT CACCCTGA	NZ_CP060027.1					
418	GCAACCCGTTTT GGTGGGTGCTG ACCTTGGTCGGC ACGAATGCCTCC TGCACGCGCGA CC	NZ_CP047310.1, NZ_CP060259.1	N	N	N	N	N
419	GCAACCCGCTTT GGTGGGTGCCG ACCGTTGGTCGG CACGGATGCCT GTGCCTCTCGAT	NZ_CP083454.1,NZ_LS483406.1	N	N	N	N	N
420	GCAACCCGCTTT GGTGGGGGCCG ACCGTTGGTCGG CACGGATGCCT GTGCCTCTCGAT C	NZ_CP040431.1, NZ_CM001824.1, NZ_CP049956.1, NZ_CP104290.1, NZ_CP104288.1	N	N	N	N	N
421	GCAACCCGCTTT GGTGGGGGCCG ACCGTTGGTCGG CACGGATGTCTG TGCCTCTCGAT	NZ_AP021908.1, NZ_CP044092.1	N	N	N	N	N
422	GCAACCCGCTTT	NZ_CP008838.1,N	N	N	N	N	N

	GGTGGGGGCCG ACCGTTGGCCG GCACGGATGCC TGTGCCTGTCGA TC	Z_CP065965.1,NZ _LT906480.1					
423	GATGGTGCCGTT GAGGTTGCCGTT GAGCAGGTT	NZ_CP104071.1, NZ_CP040436.1	N	N	N	N	N
424	GATCGAGAGGC ACAGGCATCCG TGCCGACCAAC GGTCGGCCCCC ACCAAAGCGGG TTGC	NZ_CP060021.1, NZ_CP051467.1, NZ_CP104863.1, NZ_CP060027.1	N	N	N	N	N
425	GAGCCCGGGAA CAATGAGG	NZ_CP040438.1, NZ_CP043578.1	N	N	N	N	N
426	GAGCAGACCCA GACCCCGTGCC AAGCAAGCTTG GCACCTACCAG AACAAGGACAG TGGAA	NZ_CP060022.1, NZ_CP060023.1	N	N	N	N	N
427	GACCTCTGGTGG GTACTGACCTTT GGTGGGTTCTGA CCTTTGGTGGGG	CP091781.1, NZ_CP033829.1	N	N	N	N	N

	CCGACCG						
428	GACACGGTAGT GCCGGCCGCTG GCCGGCAACTTC GTGATCCGGGT GCGGATCTGCT	NZ_CP040440.1, NZ_CP102942.1, NZ_CP049956.1, CP078102.1, NZ_CP088240.1	N	NZ_CP060026.1-1, NZ_CP040440.1-1, NZ_CP022053.2-2	N	N	100
429	GACACGGCAGT GCCGGCCGCTG GCCGGCAACTTC GTGATCCGGGT GCGGATCTGCT	NC_010943.1,NZ_ CP104290.1	N	N	N	N	N
430	GAATGCAGTAG ATCCACGCCATG CGTGGATACGC GCTATAGGGC	NC_017671.1, NZ_CP104323.1,N Z_CP040434.1	N	N	N	N	N
431	GACCCCAATGC CCCGGTGGGTG CCAACCTTGGTT GGCACTCTTTAC TGCCCT	NZ_CP083454.1, NZ_CP104292.1, NZ_LS483406.1, NZ_CP040431.1, NC_010943.1,NZ_ CP049956.1, NZ_CP040430.1, NZ_CP052863.1, NZ_CP044092.1	N	N	N	N	N
432	GACCCCAACGC CCCGGTGGGTG	CP091781.1, NZ_CP033829.1	NZ_CP049956.1-6, NZ_CP088241.1-2, NZ_CP065965.1-6, NZ_CP044092.1-5,	N	N	N	100

	CCAACCTTGGTTGGCACTCTTTACTGCCCT		NZ_CP040437.1-4, NZ_CP101622.1-3, NZ_CP088243.1-4, NZ_CP088242.1-4, NZ_CP065965.1-7, NZ_CP040438.1-2				
433	GACACGGTAGTGCCGGCCGCTGGCCGGCAACTTCGTGATCGGGGTGCGGATCTGCT	NZ_CP060021.1, NZ_CP044092.1	N	NZ_CP060026.1-1, NZ_CP040440.1-1, NZ_CP022053.2-2	NZ_CP060026.1-10	N	100
434	GAATCATCAGATCGCGATGCTGCA	NZ_CP104286.1, NZ_CP033829.1	N	N	N	N	N
435	GAAGCGGGTAGGTGCCGACCGTTGGTCGGCACACCGGTTATTGCATG	NZ_CP083454.1, NZ_LS483406.1, NZ_CP014014.1, CP091781.1, NZ_CP049956.1, NZ_CP104286.1, NZ_CP104290.1, CP078102.1, NZ_CP040430.1, NZ_CP033829.1, NZ_CP033829.1, NZ_CP065965.1, NZ_LT906480.1	N	N	N	N	N
436	GAAGACGTGCCTTGGTAGAGGC	NZ_LR134324.1, NC_017671.1,	N	N	N	N	N

	CGACCTTGGTCG GCGCTTTT	NZ_CP028899.1, NZ_CP040434.1					
437	GAAACGGGTAG GTGCCGACCGTT GGTCGGCACAC CGGTTATTGCAT G	NZ_AP021908.1, NZ_CP040440.1, NZ_CP060026.1, NZ_CM001824.1, NZ_CP088240.1, NZ_CP044092.1	N	N	N	N	N
438	CTTTCGTTAGGA ACCCGTTACGTT TAGGCA	NZ_CP102942.1, NZ_CP104288.1	NZ_CP104288.1-4	NZ_CP102942.1-1	N	N	100
439	CTGCAGCCAGG CATGGCCTGGCT CTACTGCCGGTT GCCTGG	NZ_CP029773.1, NC_011071.1, NZ_CP040438.1, NZ_CP040432.1, NZ_AP021687.1	N	N	N	N	N
440	CTGACGTCACAT CCACGCATGGC GTGGATCTACTG CCAGGCCGATG AA	NZ_CP083454.1, NZ_LS483406.1, NZ_CM001824.1, NZ_CP049956.1	N	N	N	N	N
441	CTCTTCGCGGGA ACCACGCGTGG CGTGGATCTACT GCCGGTCAGCCT GCCGTGGGTC	NZ_CP040432.1, NZ_AP021687.1	N	N	N	N	N

442	CTCTGCCTGGGTCTGGTAGTTGCCAACCTTGGTTGGCAT	NZ_CP029773.1, NZ_CP080573.1	N	N	N	N	N
443	CTCTGCCTGGGTCTGGTAGTTGCCAACCTTGGTTGGCA	NC_011071.1, NZ_CP040438.1, NZ_CP043578.1, NZ_AP021687.1	N	N	N	N	N
444	CTCTACCGGAATGGCGGCGGATGCGGTACGGGCGGCCGGGGCTGCGCC	NZ_CP040439.1, NZ_CP040438.1	N	N	N	N	N
445	CGTCGGTAGAGTCGACTGTTAGTCGACTGCTCTTCCTTCAACGC	NZ_CP104324.1, NZ_CP104287.1	NC_010943.1 -4	N	N	N	100
446	CGGTAGATCCGCGCCGTGCGTGGATGGGAAA	NZ_CP083454.1, NZ_LS483406.1, NZ_CP044092.1	N	N	N	N	N
447	CGGTAGATCCGCGCCATGCGTGGATGGGAAA	NZ_CP098483.1, NZ_CP060021.1, NZ_CP104292.1, NZ_CP040431.1, NZ_CP040440.1, NZ_CP102942.1,	N	N	N	N	N

		NC_010943.1,NZ_CP049956.1, NZ_CP104290.1, NZ_CP104288.1, CP078102.1					
448	CGGGCACCCCAGCGCGCCAACGGCGGGCTGGGGTGC	NZ_CP040435.1, NZ_CP028358.1	N	N	N	N	N
449	CGGGAAGAGGTCAGCAGCTACCGGTAGCGCCGGGCCATGCCCGGCGGAA	NZ_CM001824.1, NZ_CP104290.1, NZ_CP065965.1, NZ_LT906480.1	NZ_CP049368.1-2	N	N	N	100
450	CGGCCTGCAGCCGTCGCACGTCGATGCCACTG	NZ_CP027562.1, NZ_CP090418.1	N	N	N	N	N
451	CGGATGCCTCCATGGCCGTTCATCCACGCATGGCGTGGATCTACTG	NZ_CP027562.1, NZ_CP090418.1	NZ_CP049368.1-3, NZ_CP090418.1-3	N	N	N	100
452	CGCGCCGGTGAAGAAGGCTGCCGCCAAGCCAGT	NZ_CP088241.1, NZ_CP052863.1	N	N	NZ_OU943334.1-4	N	100
453	CGCCAACCAAGGTTGGTATCTAC	NZ_CP040440.1, NZ_CP060026.1,	N	N	N	N	N

	CAGAGCCGGGC CATGCATGTGA ACGCAGG	NZ_CP102942.1					
454	CGCCAACCAAG GTTGGCATCCAC CAGAGCCGGGC CATGCATGTGA ACGCAGG	NZ_LS483377.1, NZ_CP104288.1, NZ_CP040430.1, NZ_CP065965.1, NZ_LT906480.1, NZ_CP044092.1	N	N	N	N	N
455	CGCCAAATGGT AGTGCCGGCCG CTGGCCGGCAA CCTCATGCATCG CGT	NZ_CP088242.1, NZ_CP088243.1	NZ_CP049956.1-6, NZ_OU943334.1-2, NZ_CP088241.1-5, NZ_CP088241.1-2, NZ_CP065965.1-6, NZ_CP051467.1-5, NZ_CP049956.1-7, NZ_CP049368.1-3, NZ_CP044092.1-5, NZ_CP040438.1-2, NZ_CP040436.1-3, NZ_CP040435.1-5, NZ_CP037858.1-2, NZ_CP104289.1-3, NZ_CP101622.1-3, NZ_CP080573.1-5, NZ_LT906480.1-6, NZ_CP065965.1-9, NZ_CP049956.1-9, NZ_CP014014.1-7, NC_010943.1-3, NZ_CP040439.1-7, NZ_CP040432.1-2	NZ_LT906480.1-1, NZ_LR134324.1-2, NZ_CP060026.1-1, NZ_CP060023.1-4, NZ_CP060022.1-2, NZ_CP040440.1-1, NZ_CP040434.1-1, NZ_CP025298.1-1, NC_017671.1-1, NZ_CP104323.1-1, NZ_CP040429.1-1, NZ_CP022053.2-2, NZ_CP060026.1-3, NZ_CP040440.1-5, NZ_CP028358.1-3, NZ_CP101622.1-1	NZ_CP060026.1-10, NZ_CP022053.2-8	N	100
456	CGAAGCGGCAT TTCCGTGGCCTG GCCGG	NZ_CP088242.1,NZ_CP088243.1	N	N	N	N	N

457	CCTGCCGGGGC GAATGGGGCCT GGTAGAGGCCG ACCTTGGTCGGC GCTTGCCGAT	NZ_LR134324.1,NZ_CP104323.1	N	N	N	N	N
458	CCGTCTGGCAA GGGTAGTGCCG TCTTCCAGCAGC CACAGCGCACC GTCGGATGG	NZ_CP088242.1, NZ_CP088243.1	N	N	N	N	N
459	CCGTAAATCGG GGGCCCTGGCC GGCATCGCGCC GTGCAGGGCGT CAGGGG	NZ_CP065965.1, NZ_LT906480.1	NZ_LT906480.1-6, NZ_CP065965.1-9	N	N	N	100
460	CCGTAAATCGG GGGCCCTGGCC GGCATCGCGCC GCGCAGGGCGT CAGGAGGC	NZ_LS483377.1, NZ_CP088240.1	N	N	N	N	N
461	CCGGCTGCCGAT GCCGCCGCCCC GGCCACCACCG AGCAGGCCGCC A	NZ_CP011305.1, NZ_CP060259.1	N	N	N	N	N
462	CCGGCCGCCGA	NZ_CP104169.1,	N	N	N	N	N

	TGCCGCCGCCCC GGCCACCACCG AGCAGGCCGCC A	NZ_CP047310.1					
463	CCGGAGAGCAG TCGAGCATGGCT CGACTCTACAG GGGCGCTGCAC CCGTGTTTGGTA	NZ_CP067993.1, NZ_CP043578.1	NZ_OU943334.1-2, NZ_CP090418.1-3	NZ_CP028358.1-1	N	N	100
464	CCCAAGGACCC GGCAGCCGGCC AGCGGCCGGCT CTACCCATCCAT GCATGGGCCAC GCAA	CP091781.1, NZ_CP033829.1	N	N	N	N	N
465	CCATCCACGTGT GGCGGCTACAT CGGGTAGGTGC GGGCCCTGGTCC GCACGCG	NZ_CP104292.1, NZ_CP044092.1	N	N	N	N	N
466	CCATCCACGCGT GGCCGCTGCATC GGGTAGGTGCG GGCCCTGGTCCG CGCGCG	NZ_CP008838.1, NZ_CP065965.1, NZ_LT906480.1	N	N	N	N	N
467	CATGCGTCAGTG	NZ_CP088242.1,	N	N	N	N	N

	CCAACCAAGGT CTGCACCCACCA GGGCAAAGACC C	NZ_CP088243.1					
468	CATGCAATAAC CGGTGTGCCGA CCAACGGTCGG CACCTACCCGTT TC	NZ_CP051467.1, NZ_CP033586.1, NZ_CP088241.1	N	N	N	N	N
469	CAGGTCATGGC AGTGGAAAGCA TCGTGC	NZ_CP049956.1, NZ_CP044092.1	N	N	N	N	N
470	CAGGTCATGGC AGCGAGAAGCA TC	NZ_CP083454.1, NZ_LS483406.1, NZ_CP040431.1, NZ_CP104290.1	N	N	N	N	N
471	CAGCAGGATCA AGCGAGGATGC GGGGGCAACAG TCCTCGCTTGCG ACGAGGGCT	CP091781.1, NZ_CP052863.1, NZ_CP052863.1	N	N	N	N	N
472	CAGAGCAGACC CCGTGCCAAGC AAGCTTGGTACC TACCAGAACGA GGACAGTGGAA	NZ_CP060024.1, NZ_CP101622.1, NZ_CP033586.1,N Z_CP104863.1	N	N	N	N	N

	G						
473	CAGCCTATTGCC CCGGTAGGTGC CC	NC_011071.1,NZ_ CP040432.1,	N	N	N	N	N
474	CACTACCGTGGC GTTCACTGGCCC A	NZ_CP104292.1, NZ_CP104288.1, CP078102.1, NZ_CP088240.1, NZ_CP044092.1	N	N	N	N	N
475	CACCAGGGCAG TAAAGAGTGCC AACCAAGGTTG GCACCCACCGG GGCATTGG	NZ_CP060021.1, NZ_CP022053.2, NZ_CP060027.1, NZ_CP088241.1	N	N	N	N	N
476	CAAGGCCAAGC CTGCCGCTGCCA GCAAGAAGGCG CCGGT	NZ_CP033586.1, NZ_CP088241.1, NZ_CP052863.1	N	N	N	N	N
477	ATTTTGGCGATC TGGACGAAGAG CAGTCGACCAA	NZ_CP008838.1, NZ_CP065965.1, NZ_LT906480.1	N	N	N	N	N
478	ATGGATCCAGG ACGGCGCCAAC CAAGGTTGGCG ACTACCGCGGT ACAGCGCTCT	NZ_AP021908.1, NZ_CP040431.1, CP091781.1, NC_010943.1, NZ_CP040430.1,	N	N	N	N	N

		NZ_CP033829.1					
479	ATGGAAGCCAG CGCCAACCAAG GTTGGCGGCTAC CAGGCCTGAGC GGCGTTGCTCT	NZ_CP014014.1, CP091781.1, NZ_CP033829.1	N	N	N	N	N
480	ATGCATCCGTGC CAACCAAGGTT GGCACCTACCG AGCGATGGCAA ATCGCG	NZ_CP104324.1, NZ_CP104287.1	N	N	N	N	N
481	ATGAGTCTGCCT TGGTGGGTGCC GACCTTGGTCGG CACATGAATCTG CC	NZ_CP104286.1, NZ_CP033829.1	N	N	N	N	N
482	ATGAACGATTG AAGCATCACCT GGA	NZ_CP008838.1, NZ_CP040430.1, NZ_CP065965.1, NZ_LT906480.1	N	N	N	N	N
483	ATCGTGCACCGC TTGGTAGGTGCC GACCTGGGTCG GCACAGACCTG G	NZ_CP040432.1,NZ_AP021687.1	N	N	N	N	N
484	ATCGCGCGCAG	NC_011071.1,	N	N	N	N	N

	CGCAGAATGTGTGGTCCC	NZ_AP021687.1					
485	ATCGAGAGGCACAGGCATCCGTGCCGACCAACGGTCGGCACCCACCAAAGCGGGTTGT	NZ_CP101622.1, NZ_CP088241.1	N	N	N	N	N
486	ATCATGGCGTCGATTGGCCCTGTGT	NZ_LS483377.1, NZ_CM001824.1, NZ_CP040430.1	N	N	N	N	N
487	AGGTTCATGGCGTCGGCGGAATAGGTGC	NC_011071.1, NZ_CP040432.1, NZ_AP021687.1	N	N	N	N	N
488	AGGGCAGTAAAGAGTGCCAACCAAGGTTGGCACCCACCAACGCAGTAAAGA	NZ_CP051467.1, NZ_CP104863.1	N	N	N	N	N
489	AGGATCATGGCGTCGATTGGCCCTGTGT	NZ_CP049956.1, NZ_CP044092.1	N	N	N	N	N
490	AGCGGATCATGGCGTCGATTGGCCCT	NZ_CP083454.1, NZ_LS483406.1, NZ_CP040431.1, NZ_CP104290.1	N	N	N	N	N

491	AGCCAGCCCAT GGCAGCGTCTC AGGAACGTTGC TTCGGTGC	NZ_CP104290.1, CP078102.1, NZ_CP088240.1, NZ_CP008838.1, NZ_CP065965.1, NZ_LT906480.1	N	N	N	N	N
492	AGCAGCAGGAT CAGGCGAGGAT GCGGGGGCAAC AGTCCTCGTTTG CGACGAGGGCT GTG	NZ_CP040437.1, NZ_CP088242.1, NZ_CP088243.1	N	N	N	N	N
493	AGCAGATCCGC ACCCGGATCAC GAAGTTGCCGG CCAGCGGCCGG CACTACCGTGTC	NZ_CP060026.1, NZ_CP101622.1	NZ_CP049956.1-6, NZ_CP088241.1-2, NZ_CP065965.1-6, NZ_CP044092.1-5, NZ_CP040437.1-4, NZ_CP101622.1-3, NZ_CP088243.1-3, NZ_CP088242.1-4, NZ_CP065965.1-7	NZ_CP060026.1-1, NZ_CP040440.1-1, NZ_CP022053.2-2	NZ_CP060026.1-10	N	100
494	AGCAACAGTTCT CGTTTGCGATGA GGGCTGTTGCCG GCCAGCGGCCG GCACTACCGGG GCAGCAGGATC AAGCGAGGATG CGGGGGCAACA GTCCTCGTTTGC	NZ_CM001824.1, CP078102.1, NZ_CP088240.1	NZ_LT906480.1-6, NZ_CP065965.1-9, NZ_CP014014.1-7, NC_010943.1-3, NZ_CP088241.1-5, NZ_CP065965.1-7, NZ_CP049956.1-7, NZ_CP049956.1-6, NZ_CP049368.1-3, NZ_CP040439.1-7, NZ_CP104292.1-2	NZ_LT906480.1-1	N	N	100

495	GACGAGGGCTG AGATCGGAGGT AGATCGTGGAA TGGGATGTGCC GACCAACAGTC GGCACCCACCC GACCGGTAGCG CCGGCCGCTGG CCGGCG	NZ_CP065965.1, NZ_LT906480.1	N	N	N	N	N
496	AGAGCGCTGTA CCGCGGTAGTC GCCAACCTTGGT TGGCGCCGTCCT GGATCCAT	NZ_CP060021.1, NZ_CP056088.1, NZ_CP060027.1, NZ_CP104863.1	N	N	N	N	N
497	AGAGCCAGGCC ATGCCTGGCTGC AGGT	NZ_CP101622.1, NZ_CP104863.1, NZ_CP008838.1	N	N	N	N	N
498	AGAAGGCGGTT CGCATCCGCCG AAGTGGAACAG TAGATCCACGCC GTGCGTGGATG C	NZ_CP027562.1, NZ_CP090418.1	N	N	N	N	N
499	AGAAGCCCGGG TGTGCAGACAC CCGGGCTTTTTT	NZ_CP051467.1, NZ_CP101622.1	N	N	N	N	N

500	TGTTTGCCTTTC AATGAGGC AGAAAGCGGGA GCGCGGTAGAT CCATGCCACGC GTGGATGATTTT TCCCGAGTGTCG AA	NZ_CP027562.1, NZ_CP090418.1	N	N	N	N	N
501	ACGCATGCATCC ACGCATGGTGT GGATCTACAGC AGCTGGTCTTT	NZ_CP060027.1, NZ_CP088241.1, NZ_CP104863.1, NZ_CP008838.1	N	N	N	N	N
502	ACCTGCAGCCA GGCATGGCCTG GCTCTACTGCCG GTTGCC	NZ_CP088242.1, NZ_CP088243.1	N	N	N	N	N
503	ACCTGCAGCCA GGCATGGCCTG GCTCT	NZ_CP102942.1, NZ_CP104290.1, CP078102.1, NZ_CP088240.1, NZ_CP033829.1, NZ_CP065965.1, NZ_LT906480.1	N	N	N	N	N
504	ACCGGGGGGCC TGGCGCGCAGC GGTCGCCATGC	NZ_CP040440.1, NZ_CP060026.1	N	N	N	N	N

505	GCGGCTCTGGTA ACAACCCGCTTT GGTGGGTGCCG ACCGTTGGTCGG CACGGATGCCT GTGCCTCTCGAT	NC_010943.1, CP078102.1, NZ_CP088240.1	N	N	N	N	N
506	AAGTCAGATCC CGCATCGGTAG CGTCGGGCCAA TGCCCGGCAAG CGCAGCGGCAC	NZ_CP104071.1, NZ_CP040436.1	N	N	N	N	N
507	CGTAAATCGGG GGCCCTGGCCG GCATCGATCGC GCCGTGCACGA CGTCAGGAG	NZ_AP021908.1, NZ_CP104288.1	N	N	N	N	N
508	AAGCCCGCTCTG GTGGGTGCCGA CCTTGGTCGGCA CTGAAGCTGGC CCAGC	NZ_AP021908.1, NC_010943.1	N	N	N	N	N
509	AAGCCAGATCC CGCATCGGTAG CGTCGGGCCAA TGCCCGGCAAG CGCAGCGGCAC	NZ_CP104324.1, NZ_CP104287.1	N	N	N	N	N

510	AAAGGCATTTATCAACAAAGGGCCCGGTCACGATGGCTTGGTCGGTCGCTCCAAGCTTG	NZ_CP029773.1, NZ_CP080573.1	N	N	N	N	N
511	AAAGACCAGCTGCTGTAGATCCACACCATGCGTGGATGCATGCGT	NZ_CP083454.1, NZ_LS483406.1, NZ_CP040431.1, NZ_LS483377.1, NZ_CP065965.1, NZ_LT906480.1, NZ_CP044092.1	N	N	N	N	N
512	TCACATCCAACTTCGGCGTCTGCAGGCGT	NZ_CP027562.1,NZ_CP090418.1	N	N	N	N	N
513	AGCAGATCCGCACCCCGATCACGAAGTTGCCGGCCAGCGGCCGGCACTACCGTGTC	NZ_CP033586.1, NZ_CP088241.1	N	NZ_CP060026.1-1, NZ_CP040440.1-1, NZ_CP022053.2-2	N	N	100
514	CATGCAATAACCGGTGTGCCGACCAACGGTCGGCACCTACCCGCTTC	NZ_CP060021.1, NZ_CP101622.1, NZ_CP008838.1	N	N	N	N	N
